# Polyphenol extract of *Syzygium brachythyrsum* mitigates atherosclerosis in high-fat diet induced ApoE^-/-^ mice by regulating ROS/Keap1/Nrf2 pathway

**DOI:** 10.1371/journal.pone.0347758

**Published:** 2026-05-05

**Authors:** He-Ping Liu, Pei-Wen He, Kai-Jing Yu, Qiang Shang, Wen Xu, Xin-Fu Cai

**Affiliations:** 1 Sichuan Engineering Research Center of Antiviral Traditional Chinese Medicine Industrialization, Pengzhou, China; 2 State Key Laboratory of Traditional Chinese Medicine Syndrome, The Second Affiliated Hospital of Guangzhou University of Chinese Medicine, Guangzhou, China; 3 Chinese Medicine Guangdong Laboratory, Guangdong, Hengqin, China; Emory University School of Medicine, UNITED STATES OF AMERICA

## Abstract

*Syzygium brachythyrsum* (SB) is an ethnic herb widely used by the Dai ethnic minority in Yunnan province of China. Our previous studies have shown that the polyphenol extract of SB (PSB) exhibits antioxidant and anti-inflammatory effects, and inhibits the formation of macrophage-derived foam cells by reducing lipid uptake and inflammation, suppressing reactive oxygen species (ROS), and downregulating the NF-κB and MAPK signaling pathways. This study aims to investigate the effects of PSB on suppressing atherosclerosis in vivo and to explore the underlying mechanisms.A high-fat diet was used to induce the development of atherosclerosis in ApoE^-/-^ mice for 12 weeks. Mice were treated with three doses of PSB and atorvastatin. Morphological characteristics of aorta bulk, aortic root and liver were examined using Oil Red O and H&E staining. ELISA, immunofluorescence and fluorescence staining were used to assess lipid level, pro-inflammatory cytokines, oxidative indexes, ROS levels and CD36 protein expression in the aortic root. In vitro, an ox-LDL-induced THP-1 macrophage-derived foam cell model was established. ROS fluorescence and Western blot analyses were performed to investigate the involvement of the ROS/Keap1/Nrf2 signaling pathway, with or without the Nrf2 inhibitor ML385. The Results suggested that PSB significantly reduced lipid plaques in the aorta, and hepatic lipid accumulation. PSB also improved serum and hepatic lipid profiles and inflammatory levels, alleviated oxidative stress and reduced CD36 expression in vivo. Furthermore PSB inhibited ox-LDL induced foam cell formation, alleviated the stimulation of oxidative stress in THP-1 foam cells by activating the ROS/Keap1/Nrf2 signaling pathway. These effects were blocked by the Nrf2 inhibitor ML385.For the first time, our study revealed the anti-atherosclerotic effects of polyphenol extract of *S. brachythyrsum*. It offers insight into the potential mechanism by which PSB mitigates the early onset of AS via ROS/Keap1/Nrf2 pathway. In summary, the current study suggests that PSB, as a complementary therapy, may have a beneficial effects on cardiovascular diseases and deserves further investigation.

## Introduction

Atherosclerosis (AS), a chronic -inflammatory condition of blood vessels, is a major contributor to severe cardiovascular conditions such as stroke, heart disease, and peripheral artery disease [1,2]. The pathogenesis of AS is complex and multifactorial, primarily involving lipid accumulation, inflammation, oxidative stress, and genetics [3]. The crosstalk between oxidative stress and inflammation plays a crucial role in the formation of AS, from the development of lipid plaque to plaque rupture, causing damage to blood vessel lining cells, stimulating smooth muscle cell proliferation, and attracting macrophage migration [4–6]. Excessive oxidative stress can be triggered by various internal or external stimuli, resulting in the overproduction of reactive oxygen species (ROS) in body. During the process of atherosclerosis (AS), this surplus ROS levels damage endothelial cells, triggering the release of inflammatory mediators such as adhesion molecules and chemokines. This, in turn, attracts more inflammatory cells to adhere and infiltrate into the affected area, thereby further intensifying the inflammatory response and accelerating the progression of AS [7–9]. Additionally oxidative stress induces lipid peroxidation, a process in which low-density lipoprotein (LDL) is oxidized to form oxidized LDL (ox-LDL). Both macrophages and vascular endothelial cells possess specific recognition mechanisms for ox-LDL, which initiate the uptake of lipids and their subsequent transformation into foam cells [10].

Plant polyphenols represent a major class of natural antioxidants and anti-inflammatory ingredients [11]. Previously reported studies strongly support the role of polyphenols in preventing degenerative diseases, particularly cardiovascular diseases. For instance, paeonol has been shown to inhibit endothelial cells apoptosis, inflammation, and oxidative stress induced by ox-LDL [12]. Chlorogenic acid reduces serum levels of total cholesterol, triglycerides, and LDL in the blood of ApoE^-/-^ mice on a high-fat diet, while also reducing inflammation [13]. In the current study we focus on an ethnomedicinal plant *Syzygium brachythyrsum* Merr. & L.M.Perry (SB)*.* The fruit and leaves, known as Dongqing fruit, have long been utilized as a medicinal and edible resource in the Dai ethnic group in Yunnan province of China to treat hyperglycemia, cardiodynia and tracheitis for many years [14,15]. Our previous phytochemical investigation revealed that *S. brachythyrsum* rich in polyphenols, with over 50 polyphenols identified. Additionally, several new polyphenols were purified and elucidated by means of semi-preparative HPLC and NMR [16]. We have also developed a straightforward method for extracting total polyphenols from *S. brachythyrsum*. Chemical investigation revealed that the main components include bergenin, ellagic acid and flavonoid derivatives [17]. Pharmacological studies have demonstrated that the polyphenol extract from *S. brachythyrsum* exhibits promising antioxidant and anti-inflammatory effects in vitro. It inhibits the formation of macrophage-derived foam cells induced by oxidized LDL (ox-LDL) through mechanisms that involve the reduction of lipid uptake and inflammation, suppression of reactive oxygen species (ROS), promotion of cholesterol excretion, and downregulation of the NF-κB and MAPK signaling pathways [18]. Moreover, our preliminary in vivo findings suggest that *S. brachythyrsum* may protect against atherosclerosis in a high-fat diet-induced ApoE-/- mouse model.

In the present study, we established an AS model of ApoE^-/-^ mice induced by long term high-fat diet to investigate the anti-atherosclerotic effect of the polyphenol extract of *S. brachythyrsum* (PSB). Additionally, we explored its mechanism of action using a THP-1 macrophage cell model. This research provides a scientific foundation for the further development and utilization of *S. brachythyrsum*.

## Result

### Compositional analysis of PSB

**Fig 1:**
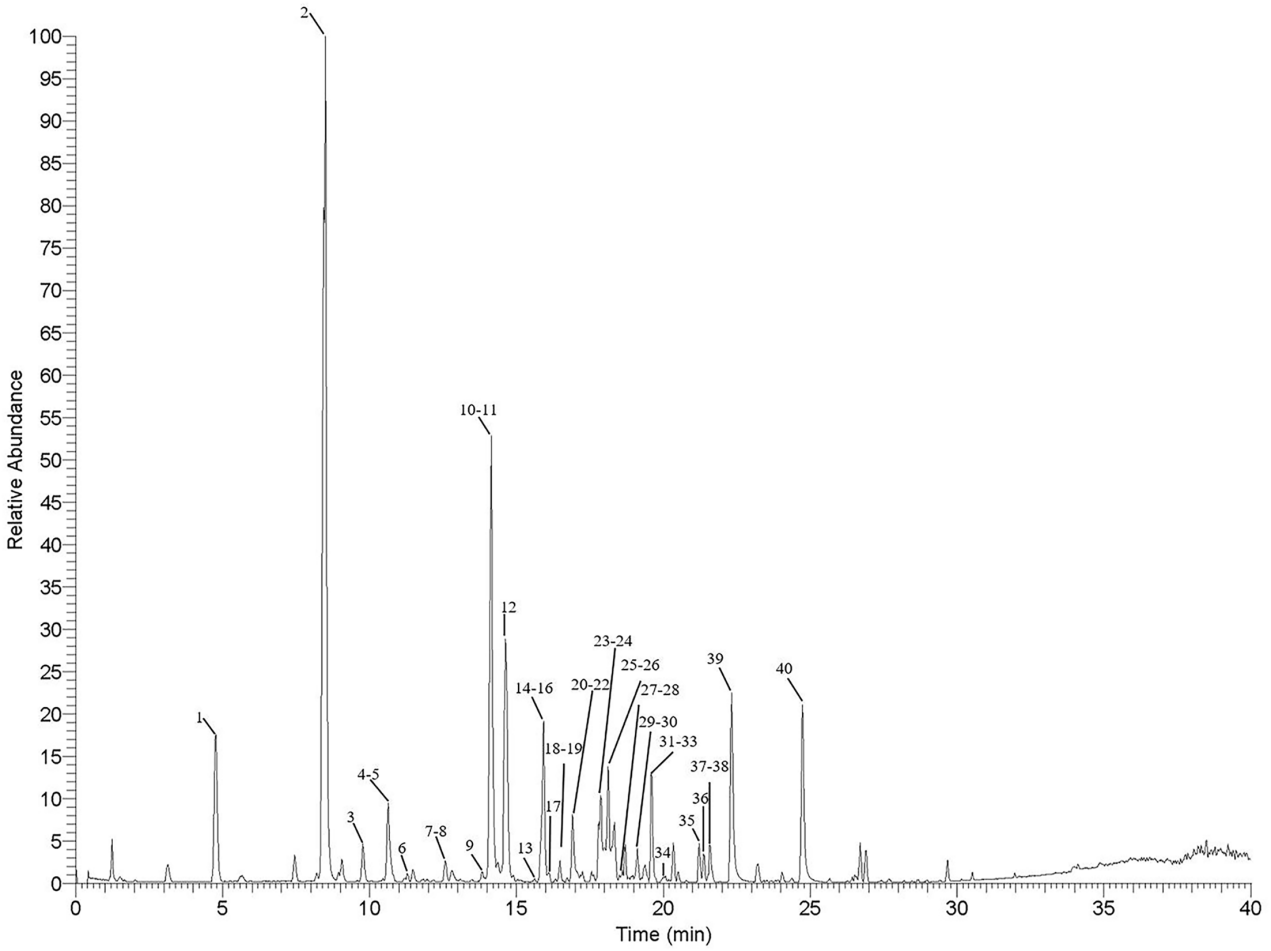
The Base Peak Chromatogram (BPC) of PSB in UHPLC-Q-Exactive Orbitrap MS. (Tested in negative ion mode).

The LC-MS of PSB (Fig 1) was analyzed using UHPLC-Q-Exactive Orbitrap MS in negative ion mode. Based on our previous study, a total of 40 major chromatographic peaks were elucidated [16] and all 40 peaks were identified as polyphenols in the PSB. These comprise 13 bergenin derivatives, 9 norbergenin derivatives, 6 brachythol B derivatives, and 12 flavonoids and phenolic acids. Detailed information on the PSB components, such as names, retention times, molecular formulas, precursor ions, mass deviations, and fragment ions, is listed in S1 Table in S1 File.

### PSB had an ameliorative effect on AS symptoms in AS mice

**Fig 2:**
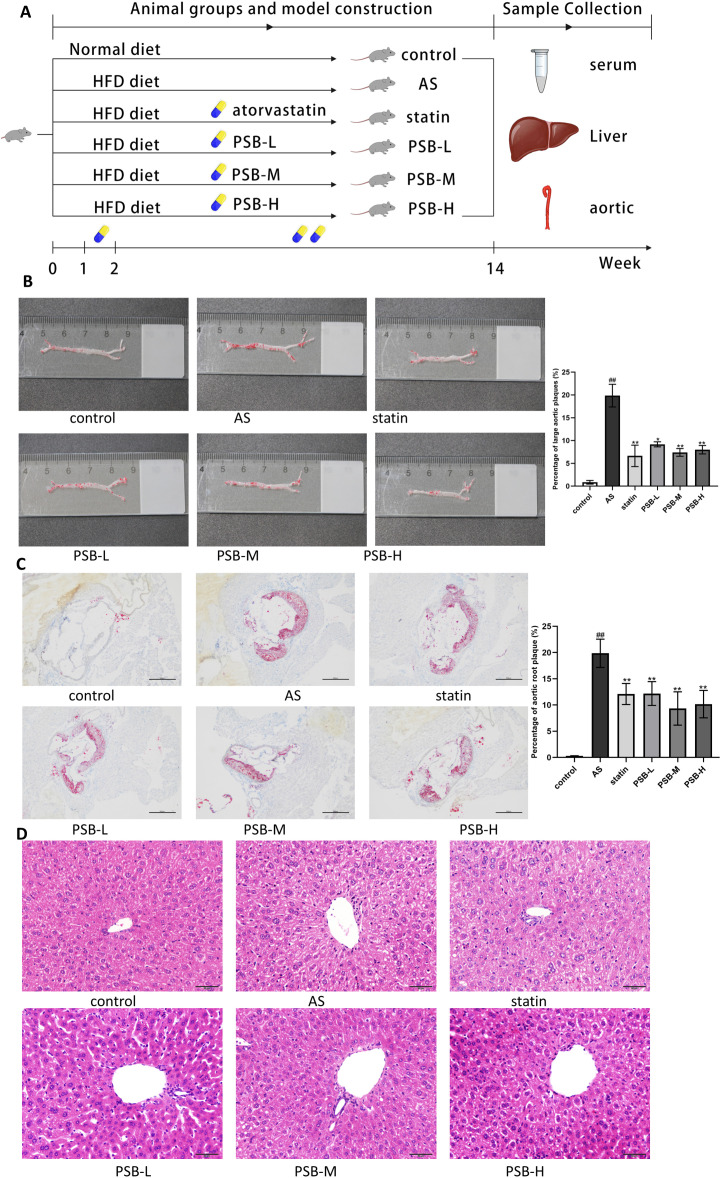
PSB improved HFD-induced AS in mice. **(A)** Experimental design, HFD: high-fat diet. **(B)** Oil red O staining of aortic bulk. **(C)** Oil red O staining of the aortic root. **(D)** H&E staining of the liver. Each group n = 4. Data are expressed as mean ± SD. ^#^*P* < 0.05 or ^##^*P* < 0.01 vs. control group, *P < 0.05 or **P < 0.01 vs. AS group.

Fig 2A presents a schematic representation of the animal modeling and dosing schedule, outlining the key steps and group arrangements utilized in the animal experiment. ApoE^-/-^ mice were subjected to a high fat diet (HFD) for 14 weeks to induce modeling. Upon completion of the experiment, liver and aorta tissues from all mice were stained to assess structural changes. Blood serum was collected to measure the levels of inflammatory cytokines/molecules and lipids.

First, we analyzed the lipid content in the aorta using Oil Red O staining. As illustrated in Fig 2B, the aortic surfaces of mice in the control group displayed minimal lipid deposits, with most of the aorta appearing transparent. In contrast, mice in the AS group exhibited pronounced lipid deposition, predominantly localized in the thoracic aorta. These findings confirmed the successful establishment of the AS mouse model. Both atorvastatin and PSB treatments demonstrated protective effects against AS-related damage, leading to a reduction in aortic lipid plaques in mice.

The aortic root was further examined using Oil Red O staining. As illustrated in Fig 2C, the aortic roots of mice in the control group exhibited no significant lipid deposition, appearing smooth and without any noticeable narrowing of the aortic lumen. In contrast, the aortic lumens of mice in the AS group were narrowed, with considerable lipid accumulation on the aortic walls, resulting in irregularities. Treatment with PSB and atorvastatin alleviated lesions in the aortic roots of AS mice, leading to a reduction in the lipid deposition area.

A long-term high-fat diet can induce pathological changes in liver tissue, contributing to fatty liver, which is closely associated with atherosclerosis. Liver tissue pathological changes were assessed using H&E staining. As shown in Fig 2D, the control group displayed normal lobule architecture and cellular shape and size. In contrast, the AS group exhibited disordered liver cell arrangement, with a marked increase in the number and size of fatty vacuoles. Notably, PSB treatment improved hepatic cell organization, reducing the number of fatty vacuoles and macrovesicular steatosis. Taken together, these results demonstrate that PSB significantly improves lipid deposition in both the aorta and liver of HFD-induced ApoE^-/-^ AS mice.

### PSB improved serum and hepatic lipid and inflammatory levels in AS mice

**Fig 3:**
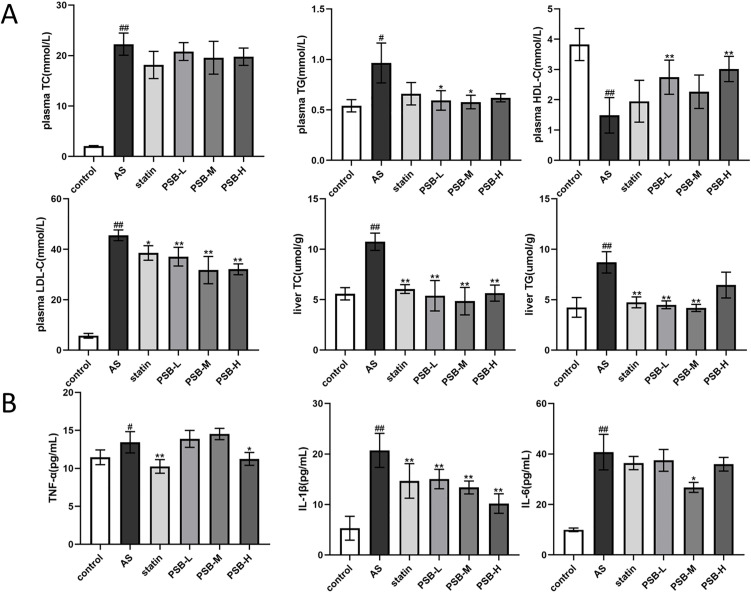
PSB improved lipid levels and reduced inflammatory factor levels in the serum of AS mice. **(A)** Lipid levels in serum and liver. **(B)** Inflammatory factor levels. Each group n = 6. Data are expressed as mean ± SD. ^#^*P* < 0.05 or ^##^*P* < 0.01 vs. control group, *P < 0.05 or **P < 0.01 vs. AS group.

Lipid metabolism abnormalities are an important risk factor in the development of AS. As shown in Fig 3A, compared with the control group, mice in the AS group had increased serum and hepatic TG and TC contents as well as increased serum LDL-C, and a decreased serum HDL-C level. PSB treatment reduced serum TG, LDL-C levels, as well as liver TG, and TC levels, and increased the serum HDL-C level to varying degrees. However, there was no significant reduction in serum TC levels in mice.

The determination of inflammatory factors in mouse serum is shown in Fig 3B. Compared with the control group, mice in the AS group had increased serum levels of TNF-α, IL-6, IL-1β. After PSB treatment, serum levels of TNF-α, IL-6, IL-1β decreased in mice. Among them, IL-1β showed dose dependence. These results demonstrated that PSB treatment improved serum and liver lipid profiles and reduced systemic inflammation.

### PSB alleviated oxidative stress and reduced CD36 expression in AS mice

**Fig 4:**
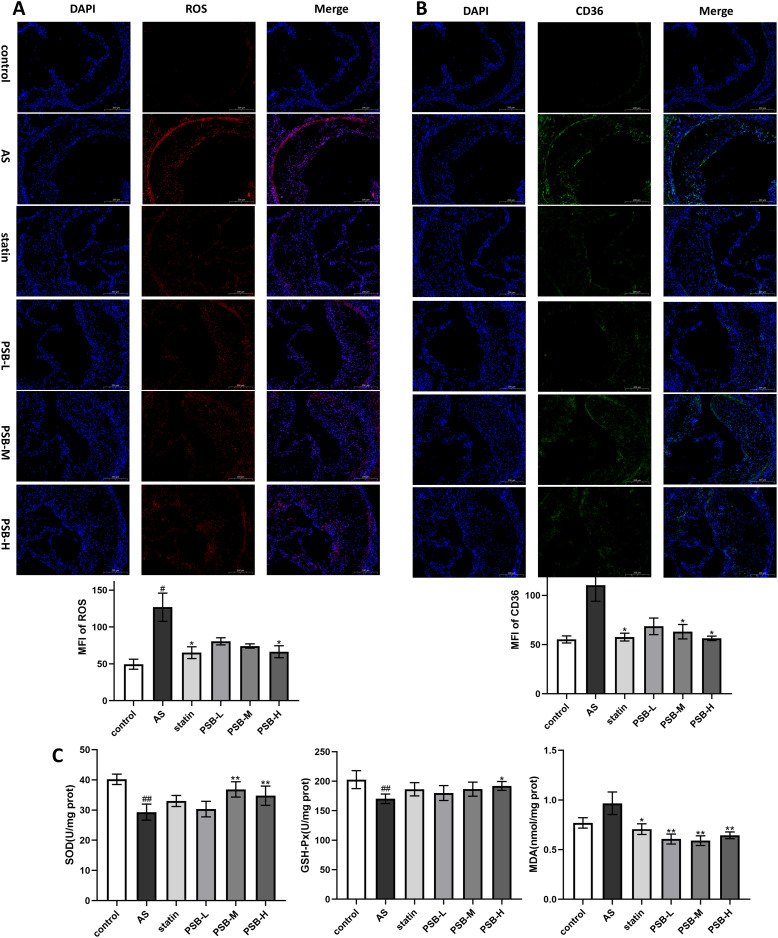
Effects of PSB to relieved oxidative stress. **(A)** Fluorescence staining of ROS by DHE in the aortic root (n = 4); (B) immunofluorescence of CD36 in the aortic root (n = 4); **(C)** Liver oxidative stress markers: GSH-Px, MDA and SOD levels (n = 6). Data are expressed as mean ± SD. ^#^*P* < 0.05 or ^##^*P* < 0.01 vs. control group, *P < 0.05 or **P < 0.01 vs. AS group.

Oxidative stress plays a significant role throughout the entire AS progression from foam cell formation to plaque rupture. In the context of oxidative stress, excessive ROS causes oxidative modification of lipids. Modified lipoproteins such as Ox-LDL stimulate the expression of CD36, promoting lipid endocytosis, leading to foam cell formation. Herein, immunofluorescence and fluorescence staining of ROS and CD36 in the aortic root of AS mice(Fig 4B and 4C) demonstrated increased expression of ROS and CD36 compared with the control group (*P* < 0.05), confirming severe oxidative stress in AS mice. After PSB treatment, there was a dose-dependent decrease in CD36 and ROS expression in the aortic root of mice (*P* < 0.05). Oxidative stress indicators such as SOD, GSH-Px and MDA were further tested by ELISA method. As shown in Fig 4C, AS group showed significantly reduced antioxidant enzyme activities of SOD and GSH-Px, while MDA levels were elevated. In contrast, PSB reduced the production of MDA, one of the major final products of lipid peroxidation, and also increased the expression of the two antioxidant enzymes SOD and GSH-Px.

### PSB inhibited lipid and reduced NO release in vitro

**Fig 5:**
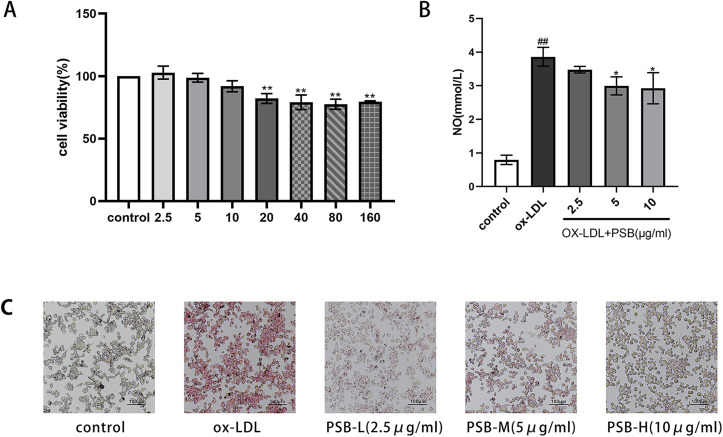
PSB inhibited lipid and reduced NO release in ox-LDL-induced THP-1 macrophages. **(A)** Determination of THP-1 cell viability by MTT assay. **(B)** THP-1 foamy cell NO release. **(C)** Oil red O staining of THP-1 foam cells. Each group n = 3. Data are expressed as mean ± SD. ^#^*P* < 0.05 or ^##^*P* < 0.01 vs. control group, *P < 0.05 or **P < 0.01 vs. ox-LDL group.

Our above study demonstrated that the anti- atherosclerotic effect of the polyphenolic extract may be attributed to the strong antioxidant property in vivo. To further explore its potential antioxidation mechanism, we employed a foam cell model using PMA-induced THP-1 macrophages. This foam cell model was successfully established through ox-LDL treatment. Initially, MTT assay was conducted to determine the cytotoxic concentration of PSB. Cell viability began to decline at PSB concentrations over 40 μg/mL, with approximately 80% viability observed at 160 μg/mL. No significant differences in cell viability were noted at concentrations below 10 μg/mL compared with the control group. Consequently, we selected low dose (2.5 μg/mL), medium dose (5 μg/mL), and high dose (10 μg/mL) for the subsequent experiments (Fig 5A).

NO measurement experiment showed an increase in intracellular NO content after 24 hours of ox-LDL induction, whereas it decreased after PSB administration (Fig 5B) in a dose dependent manner. Oil Red O staining of the cells confirmed successful modeling of the foam cells, and after PSB treatment, a reduction in lipid content stained by Oil Red O was observed (Fig 5C). These results indicate that PSB can reduce lipid accumulation and NO production, and thus, inhibiting foam cell formation triggered by Ox-LDL.

The nitric oxide (NO) measurement experiments indicated an increase in intracellular NO levels following 24 hours of ox-LDL induction, which was reduced after PSB administration (Fig 5B) in a dose-dependent manner. Oil Red O staining confirmed the successful modeling of the foam cells, and after PSB treatment, a decrease in lipid content was observed, as indicated by the Oil Red O staining (Fig 5C). These findings suggest that PSB effectively reduces both lipid accumulation and NO production, thereby inhibiting the formation of foam cells induced by ox-LDL.

### PSB alleviated the stimulation of oxidative stress in THP-1 foam cells by activating the Keap1/Nrf2 signaling pathway

**Fig 6:**
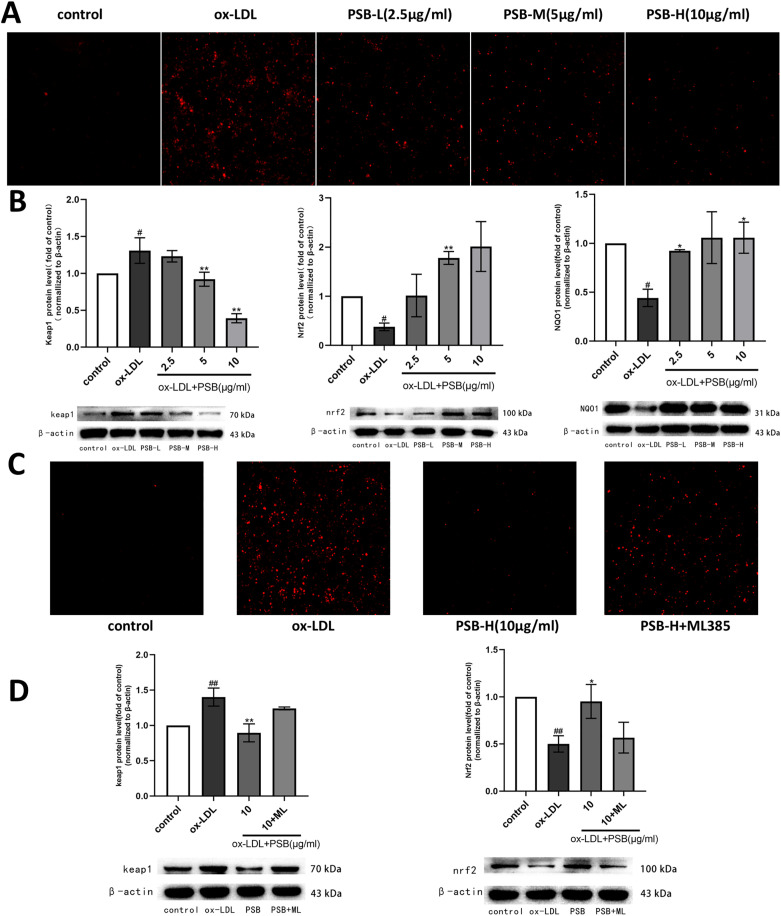
PSB attenuated the effects of stimulation by oxidative stress in THP-1 foam cells by activating the Keap1/Nrf2 signaling pathway. **(A)** ROS fluorogram of THP-1 foam cells. **(B)** Western blot images and quantitative analysis plots of Keap1, Nrf2 and NQO1. **(C)** ROS fluorogram of THP-1 foam cells under ML385 inhibitor. **(D)** Western blot images and quantitative analysis plots of Keap1 and Nrf2 under ML385 inhibitor. Each group n = 3. Data are expressed as mean ± SD. ^#^*P* < 0.05 or ^##^*P* < 0.01 vs. control group, *P < 0.05 or **P < 0.01 vs. ox-LDL group.

The anti-oxidant mechanism was further investigated using the THP-1 foam cell model. Fluorescence staining of cellular reactive oxygen species (ROS) showed that ox-LDL triggered an increase in ROS fluorescence intensity, which could be reversed by PSB administration (Fig 6A). Additionally, PSB treatment led to a reduction in the expression of Keap1 protein and an increase in the expression of Nrf2 and NQO1 proteins (Fig 6B). To determine whether PSB mitigates oxidative stress and inhibits foam cell formation through modulation of the Keap1/Nrf2 pathway, we introduced the Nrf2 inhibitor ML385 to the cell model. The presence of ML385 significantly reduced the efficacy of PSB, resulting in elevated ROS fluorescence intensity (Fig 6C). Furthermore, no significant differences in Keap1 and Nrf2 protein expression were observed compared to the ox-LDL group, indicating a diminished effectiveness of PSB in this context (Fig 6D). These results suggest that PSB can effectively reduce oxidative stress by modulating the Keap1/Nrf2 pathway, thereby inhibiting the formation of foam cells.

## Discussion

Statins are widely used for the prevention of AS due to their ability to inhibit cholesterol synthesis in the liver, promote myocardial remodeling, inhibit thrombus formation, and stabilize atherosclerotic plaques [19]. However, long-term use of statins can lead to adverse effects, including elevated liver enzymes and muscle pain. In recent years, traditional herb medicine has gained attention for its potential applications in the prevention and treatment of AS [20,21]. *Syzygium brachythyrsum* (SB), called Dongqing fruit, is used as folk medicinal-edible resource by Dai ethnic group in Yunnan province. In the present study, we enriched the total polyphenols extract (PSB) using a straightforward method. Our previous research indicated that PSB had lipid-lowering and anti-inflammatory effects, achieved through pathways such as reducing NO release, decreasing macrophage uptake of ox-LDL, promoting cholesterol excretion, and inhibiting inflammation [18]. Herein, we proved that PSB could alleviate the pathological features of aorta atherosis in HDF-induced AS ApoE^-/-^ mice. Combining the above studies, it’s suggested that PSB has great potential for preventing and treating AS.

**Fig 7:**
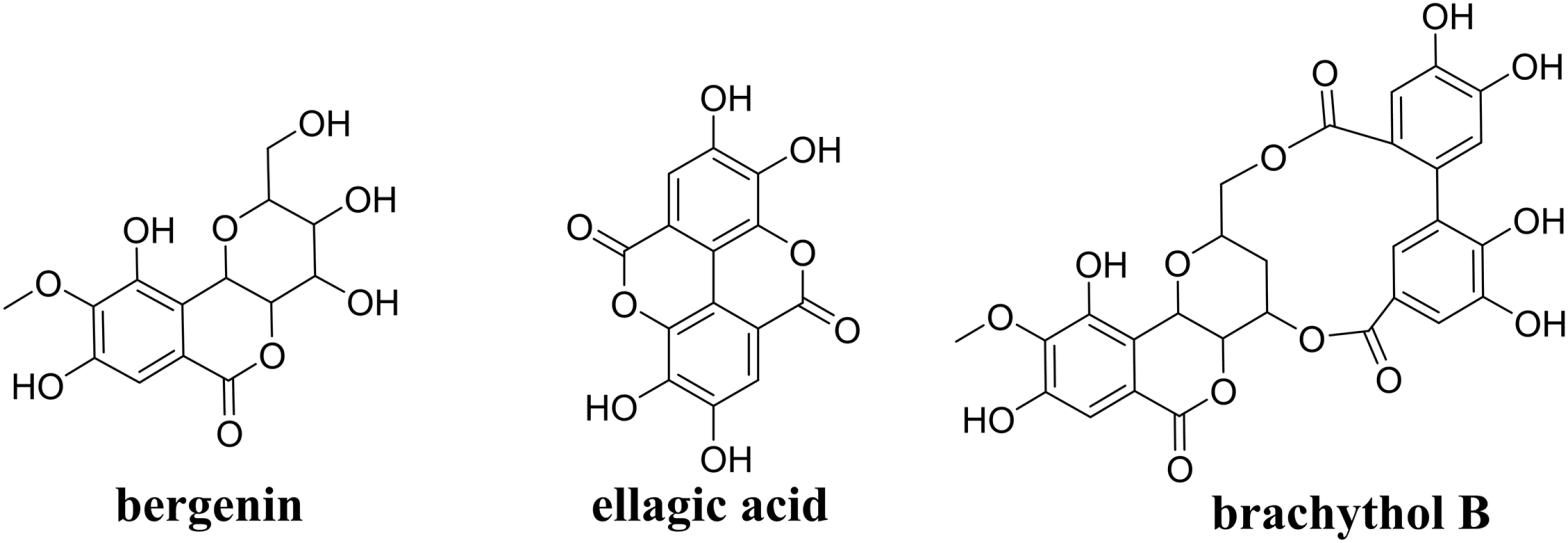
The structures of bergenin, ellagic acid and brachythol B.

Our chemical works clearly showed that PSB contains large contents of tannic acid-type polyphenolics such as bergenin, ellagic acid and Brachythol B derivatives [17]. Several studies have demonstrated that bergenin [22] and ellagic acid [23] exert anti-AS potency by inhibiting oxidative stress via Nrf2 activation and other mechanism. Brachythol B is a new compound isolated from PSB in our lab. Structurally, it is composed of ellagic acid and bergenin modules linked via two ester bonds (Fig 7). Our previous study demonstrated brachythol B possess significant antioxidant and anti-inflammatory activities in vitro. Further investigation of the anti-AS effect of these individual compounds should be conducted, which may lead to new chemical and biological discoveries.

After engulfing large amounts of lipids, macrophages form foam-like cells rich in fats, a process known as macrophage foam cell formation. Driven by excessive ROS, LDL undergoes lipid peroxidation, producing Ox-LDL, which plays a key role during this process [24]. In response to ROS-induced damage, the body produces protective proteins to mitigate this damage through the activation of Keap1-Nrf2/ARE pathway, inducing expression of antioxidant enzymes such as NQO1, HO-1, SOD, GPX, etc., thereby scavenging ROS and counteracting excessive oxidative stress [25–27]. Previous reports have demonstrated that the main components in PSB such as quercetin, bergenin and ellagic acid ameliorates oxidative damage through upregulating NRF2 [28–30]. This suggests that activating the classical Keap1-Nrf2 signaling pathway may contribute to PSB’s beneficial effects against AS. To support this hypothesis, we added the Nrf2 inhibitor ML385 and test phosphorylation levels of Nrf2, Keap1, and intracellular fluorescence ROS in THP-1 macrophages. Our study results show that ML385 could block the effects of PSB on the ROS/Keap1/Nrf2 pathway (Fig 6).

To summarize, our study suggests that PSB plays a role in combating early-stage atherosclerosis by improving lipid metabolism, mitigate oxidative stress, suppress inflammation, and demonstrated its potential as an anti-AS drug. However, there are limitations in the current research, that require further experimental investigation. This study found that PSB can reduce CD36 expression and the levels of lipid peroxidation end product malondialdehyde. A targeted lipidomics analysis should be done to profile those oxidated lipids that may be involved in the underlying mechanism of foam cell formation. Further exploration of the crosstalk between oxidative stress and inflammation on the AS development, specifically, the ROS/NF-κB signaling pathway is planned to examine whether excessive ROS activates NF-κB, thereby promoting the expression of macrophage pro-inflammatory factors such as TNF-α and IL-6.

## Materials and methods

### Chemicals and reagents

Atorvastatin calcium tablets were provided by Pfizer Inc. (USA). A 4% paraformaldehyde solution (KJ30089) was obtained from Crystalgen Biotech (Guangzhou, China). DHE stain (37291) and PMA (Phorbol 12-myristate 13-acetate, 524400) were purchased from Sigma-Aldrich (USA). DAPI (C1005), anti-fade mounting medium (P0126), BCA protein concentration assay kit (P0011), and reactive oxygen species assay kit (S0033S) were provided by Beyotime Biotechnology (Shanghai, China). Saturated Oil Red O staining solution (P0047), FITC-conjugated goat anti-rabbit secondary antibody (PN0045), CY3-conjugated goat anti-rabbit secondary antibody (PN0051), primary antibody dilution buffer, and citrate repair solution were supplied by PinoPharm (Wuhan, China). Triton X-100 (T8200) was provided by Solarbio (Beijing, China). CD36 (P16671) was supplied by Wuhan Sanying Biotechnology (Wuhan, China). HE staining was provided by Raygene Biotech (Beijing, China). Triglyceride assay kit (A110-1–1), total cholesterol assay kit (A111-1–1), high-density lipoprotein cholesterol assay kit (A112-1–1), low-density lipoprotein cholesterol assay kit (A113-1–1), glutathione peroxidase assay kit (A005-1–2), total superoxide dismutase assay kit (A001-3–2), and malondialdehyde assay kit (A003-1–2) were obtained from Nanjing Jiancheng Bioengineering Institute (Nanjing, China). Mouse interleukin 6 ELISA assay kit (JLW20268-96T), mouse tumor necrosis factor α ELISA assay kit (JLW10484-96T), and mouse interleukin 1 β ELISA assay kit (JLW18442-96T) were provided by Jianglai Bio (Shanghai, China). Oxidized low-density lipoprotein (L34357) was provided by Guangzhou Yiyuan Biotech Co., Ltd. (Guangzhou, China). South American fetal bovine serum (C04001) was provided by Vivacell (Shanghai, China). Secondary antibodies (31460) were provided by Gibco (USA). Immobilon Western HRP Substrate was provided by Millipore (USA). Nrf2 Antibody (AF0639), Keap1 Antibody (AF5266), NQO1 Antibody (DF6437), and beta Actin Antibody (AF7018) were purchased from Affinity Biosciences (Jiangsu, China).

### Preparation of PSB

The leaves of *S. brachythyrsum* were collected in Xishuangbanna Dai Nationality Autonomous Prefecture, Yunnan Province, China. Permissions for field access were granted verbally by the private landowners, and no formal permits were required from governmental or institutional authorities as the collection activities did not involve protected areas or regulated species. The plant was authenticated by Prof. Hua-gu Ye, a senior botanist at the South Botanic Garden of Chinese Academy of Science. A voucher specimen (specimen number: GD220502) was deposited in the State Key Laboratory of Traditional Chinese Medicine Syndrome, Guangzhou University of Chinese Medicine.

The sample preparation was carried out according to previous research work [17] with slight modification. Briefly, the dried leaves of SB were roughly powdered and extracted by maceration with ten times anhydrous ethanol four times, each for 48 hours. The extract was filtered, combined and concentrated under vacuum. A total of 900 g of the crude extract was suspended in distilled water (1.5 L) and further partitioned orderly with petroleum ether, dichloromethane, and ethyl acetate three times each. The ethyl acetate fraction was vacuum concentrated to obtain the polyphenol extract of *Syzygium brachythyrsum* (PSB, 78.95g). PSB was suspended in distilled water by ultrasonic treatment (5 min at room temperature) prior to administration.

### Composition analysis of PSB

The LC-MS analysis was performed using a Thermo Fisher ultra-high performance liquid chromatography-mass spectrometry (UHPLC-MS) system, consisting of an UltiMate 3000 UHPLC coupled with a Q-Exactive Orbitrap Plus mass spectrometer. Chromatographic separation was carried out on a Waters ACQUITY UPLCHSS T3 (1.8 μm, 2.1 × 100 mm), at a flow rate of 0.2 mL/min. Gradient elution was conducted using a solvent system of 0.1% formic acid in water (A) and acetonitrile (B). The gradient program was as follows: 5%−13% B over 0−8 minutes; 13%−17% B over 8−12 minutes; 17%−45% B over 12−25 minutes; 45%−70% B over 25−30 minutes; 70%−90% B over 30−35 minutes; 90%−99% B over 35−40 minutes; and 99%−5% B from 40-40.5 minutes. The injection volume was 2 μL.

For mass spectrometry (MS) analysis, the spray voltage was set at 3.7 kV and the full scan range was m/z 120–1450. The sheath gas pressure was maintained at 35 psi, auxiliary gas at 5 psi, capillary temperature at 350 °C, and probe heater temperature at 320 °C. Chromatographic data were recorded and integrated using Xcalibur software (Thermo Fisher Scientific). Full scan mass spectrometry and tandem mass spectrometry (MS/MS) data were utilized for compound identification.

### Animal

Male SPF-grade ApoE^-/-^ mice were purchased from Guangdong Yaokang Biotechnology Co., Ltd. (License No.: SCXK (Guangdong) 2020−0054), weighing 22−26 g, aged 7−8 weeks, and maintained under controlled conditions at a temperature of 22 ± 2 °C and humidity 55 ± 10%, with a 12-hour light/dark cycle. Throughout the experiment, the animals were provided with free access to food and water and were housed in the SPF animal facility of Guangdong Provincial Hospital of Traditional Chinese Medicine (Animal Experiment Facility License No.: SYXK (Guangdong) 2018−0094). The animal study was approved by the Institutional Animal Care and Use Committee of Guangdong Provincial Hospital of Traditional Chinese Medicine (No.2022021). All efforts were made to minimize the number of animals used and their suffering. For blood collection, animals were anesthetized with an intraperitoneal injection of ketamine (80 mg/kg) and xylazine (10 mg/kg). At the end of the experiments, animals were euthanized by cervical dislocation under deep anesthesia.

### Animal groups and model construction

ApoE^-/-^ male mice were randomly divided into six groups, each consisting of 10 mice: a control group (Blank group), a model group (AS group), an positive control group (atorvastatin, statin group), and low-dose PSB (PSB-L group), medium-dose PSB (PSB-M group), and high-dose PSB (PSB-H group) groups. During the experiment, the positive control (statin group) received 5 mg/kg/day of atorvastatin. The PSB-L, PSB-M, and PSB-H groups received doses of 150, 300, and 600 mg/kg/day respectively. After one week of acclimatization with a normal diet, all groups except the control group were fed a high-fat diet to induce atherosclerosis. The treatment groups (including the atorvastatin, PSB-L, PSB-M, and PSB-H groups) received oral gavage administration, while mice in the control and AS groups received an equivalent volume of 0.9% saline by oral gavage. PSB was administered by oral gavage according to a two-phase dosing schedule: twice weekly during the first week to allow gradual adaptation to the experimental procedures and minimize stress-related confounding effects, followed by once daily from week 2 until the end of the experiment (week 14) to maintain sustained therapeutic exposure.

### Sample collection

After anesthetizing the mice, the blood was collected by retro-orbital sampling method and transferred into centrifuge tubes, which were left at room temperature for 30 minutes. The tubes were then centrifuged at 3000 rpm for 10 minutes to separate the serum. The supernatant serum was carefully transferred into new centrifuge tubes and stored at −80 °C. Immediately after blood collection, the liver and aorta tissues were dissected and stored either at −80 °C or fixed in a fixative solution for subsequent experiment. For histological evaluation, tissue samples from 4 mice in each group were randomly selected for analysis.

### Biochemical parameters determination

Serum levels of total cholesterol (TC), triglycerides (TG), high-density lipoprotein cholesterol (HDL-C), and low-density lipoprotein cholesterol (LDL-C), as well as hepatic levels of TC, TG, superoxide dismutase (SOD), glutathione peroxidase (GSH-Px), and malondialdehyde (MDA) were determined using commercial assay kits purchased from Nanjing Jiancheng Bioengineering Institute.

Additionally, enzyme-linked immunosorbent assay (ELISA) kits were used to detect the levels of tumor necrosis factor-alpha (TNF-α), interleukin-6 (IL-6), and interleukin-1 beta (IL-1β) in mouse serum, purchased from Jianglai Biosciences.

### Liver Hematoxylin and Eosin (HE) Staining

Liver samples were removed from the fixative solution, placed in embedding cassettes, and dehydrated using an automatic dehydrator. After dehydration, the samples were embedded in paraffin and then sectioned. The sections were deparaffinized with xylene and rehydrated through a graded series of ethanol solutions.. They were then stained with HE stain. Finally, the stained sections were observed and photographed under a light microscope.

### Oil red O staining of aorta and aortic Root

The aorta was removed from the fixative, washed with PBS, and then stained with Oil Red O reagent. The vessel wall and plaques were eventually stained white and red, respectively. The heart and upper part of the aortic root were embedded in optimal cutting temperature compound and frozen at −80 °C Frozen sections of 10 μm were cut from the tip of the aortic valve and stained with Oil Red O to assess lesions. The Image-Pro Plus software was used to assess the areas of atherosclerotic lesions in the aorta. The percentage of plaque area was calculated as (Oil Red O-positive plaque area/ total aortic area) × 100%.

### Immunofluorescence and fluorescence staining

ROS: The frozen sections of aortic roots were thawed to room temperature, and the tissue areas were circled with a hydrophobic pen. The sections were then incubated with freshly prepared DHE working solution (diluted in PBS) at 37°C in the dark for 30 minutes. After incubation, the slides were immersed in PBS and washed on a shaker in the dark. The sections were briefly drained, and DAPI working solution was applied to stain the nuclei for 10 minutes at room temperature in the dark. Subsequently, the slides were washed again in PBS on a shaker in the dark, drained briefly, and mounted with anti-fade mounting medium before covering with coverslips. Images were captured under an inverted fluorescence microscope, and average fluorescence intensity per group was calculated using Image J software.

CD36: Frozen sections were thawed to room temperature, fixed with 4% paraformaldehyde for 30 minutes, washed with PBS, and incubated with serum blocking at 37 °C for 60 minutes. After removal of the blocking solution, the sections were incubated with the primary antibody overnight at 4 °C. Following primary antibody incubation, sections were washed with PBS and then incubated with secondary antibody at 37 °C for 1 hour. Subsequently, DAPI working solution was added and incubated at room temperature for 5 minutes. After washing with PBS, the sections were mounted using anti-fade mounting medium The slides were stored at 4 °C in the dark. Fluorescence images were observed and captured under an inverted fluorescence microscope, and the average fluorescence intensity per group was calculated using Image J software.

### Cell culture and experimental conditions

The THP-1 cells were cultured in RPMI-1640 medium (Gibco) supplemented with 10% fetal bovine serum (FBS; standard South American origin) and 1% penicillin-streptomycin, and they were incubated at 37 °C in a 5% CO_2_ atmosphere. The cells were treated with phorbol myristate acetate (PMA: 160 nmol/L, Sigma) for 24 hours to induce differentiation of monocytes into macrophages. Subsequently, the macrophages were incubated with oxidized low-density lipoprotein (oxLDL: 80 μg/mL) for 24 hours to transform them into foam cells.

### Cell viability assay

THP-1 macrophages were seeded in a 96-well plate and treated with varying concentrations of PSB for 24 hours. Cell viability was assessed using the MTT assay, and the absorbance was measured at 490 nm. Cell viability was calculated as the percentage of the absorbance in the treated group compared to the control group.

### Cell oil red O staining

After administration, the culture medium was discarded, and cells were fixed with 4% paraformaldehyde at room temperature for 30 minutes. The cells were then stained with Oil Red O solution at room temperature for 30 minutes. Finally, intracellular lipid deposition was observed and photographed under an inverted microscope.

### NO measurement

After administration, the cell culture supernatant was transferred to centrifuge tubes and centrifuged at 4°C, 3000 rpm for 15 minutes to collect the supernatant. Nitric oxide (NO) levels were measured using a Griess assay kit according to the manufacturer’s instructions.

### ROS fluorescence

After treatment, the culture medium was discarded, and the cells were washed with PBS. The cells were then incubated with PBS containing 10 μM DCFH-DA fluorescent probe at 37 °C for 20 minutes in the dark. After washing with PBS, the cells were observed under a fluorescence microscope to evaluate intracellular reactive oxygen species (ROS) levels by measuring red DCF fluorescence.

### Western blot

After treatment, the culture medium was discarded, and the cells were lysed to extract total proteins. Protein concentration was determined, and western blot analysis was performed. Briefly, proteins were separated by SDS-PAGE, transferred to PVDF membranes, blocked with skim milk, incubated with primary and secondary antibodies, and visualized using a chemiluminescence detection system. Protein band intensities were quantified using Image J software.

### Statistical analysis

Statistical analysis was performed using SPSS 26 software. Data were first assessed for normality using the Shapiro-Wilk test. For normally distributed data, one-way analysis of variance (ANOVA) was conducted, followed by Bonferroni post-hoc test for homogeneous variances (P > 0.05) or Dunnett’s T3 post-hoc test for inhomogeneous variances (P < 0.05). Graphs were generated using GraphPad Prism 8 software.

## Supporting information

S1 File Table S1. Mass spectrum information of the major components in the PSB. (DOCX)

S2 File Raw images of FIG6B and 6D. (ZIP)

S3 File Raw images of Oil red O staining of aortic Root. (ZIP)

S4 File Raw images of Oil red O staining of aorta bulk. (ZIP)

S5 File Raw images of Liver H&E staining. (ZIP)

S6 File Raw images of cell Oil red O staining. (ZIP)

S7 File Raw images of cell ROS fluorescence staining. (ZIP)

S8 File Raw images of cell ROS fluorescence staining with ML inhibitor. (ZIP)

S9 File Raw images of CD36 of aorta immunofluorescence. (ZIP)

S10 File Raw images of aorta ROS fluorescence staining. (ZIP)

## References

[1] Libby P. The changing landscape of atherosclerosis. Nature. 2021;592(7855):524–33. doi: 10.1038/s41586-021-03392-8 33883728

[2] Libby P, Buring JE, Badimon L, Hansson GK, Deanfield J, Bittencourt MS, et al. Atherosclerosis. Nat Rev Dis Primers. 2019;5(1):56. doi: 10.1038/s41572-019-0106-z 31420554

[3] Björkegren JLM, Lusis AJ. Atherosclerosis: Recent developments. Cell. 2022;185(10):1630–45. doi: 10.1016/j.cell.2022.04.004 35504280 PMC9119695

[4] Chistiakov DA, Melnichenko AA, Grechko AV, Myasoedova VA, Orekhov AN. Potential of anti-inflammatory agents for treatment of atherosclerosis. Exp Mol Pathol. 2018;104(2):114–24. doi: 10.1016/j.yexmp.2018.01.008 29378168

[5] Kattoor AJ, Pothineni NVK, Palagiri D, Mehta JL. Oxidative Stress in Atherosclerosis. Curr Atheroscler Rep. 2017;19(11):42. doi: 10.1007/s11883-017-0678-6 28921056

[6] Vogel A, Brunner JS, Hajto A, Sharif O, Schabbauer G. Lipid scavenging macrophages and inflammation. Biochim Biophys Acta Mol Cell Biol Lipids. 2022;1867(1):159066. doi: 10.1016/j.bbalip.2021.159066 34626791

[7] Forteza MJ, Ketelhuth DFJ. Metabolism in atherosclerotic plaques: immunoregulatory mechanisms in the arterial wall. Clin Sci (Lond). 2022;136(6):435–54. doi: 10.1042/CS20201293 35348183 PMC8965849

[8] Trus E, Basta S, Gee K. Who’s in charge here? Macrophage colony stimulating factor and granulocyte macrophage colony stimulating factor: Competing factors in macrophage polarization. Cytokine. 2020;127:154939. doi: 10.1016/j.cyto.2019.154939 31786501

[9] Wang W, Kang PM. Oxidative Stress and Antioxidant Treatments in Cardiovascular Diseases. Antioxidants (Basel). 2020;9(12):1292. doi: 10.3390/antiox9121292 33348578 PMC7766219

[10] Tabas I, García-Cardeña G, Owens GK. Recent insights into the cellular biology of atherosclerosis. J Cell Biol. 2015;209(1):13–22. doi: 10.1083/jcb.201412052 25869663 PMC4395483

[11] Ahmadi A, Jamialahmadi T, Sahebkar A. Polyphenols and atherosclerosis: A critical review of clinical effects on LDL oxidation. Pharmacol Res. 2022;184:106414. doi: 10.1016/j.phrs.2022.106414 36028188

[12] Vellasamy S, Murugan D, Abas R, Alias A, Seng WY, Woon CK. Biological activities of paeonol in cardiovascular diseases: a review. Molecules. 2021;26(16). doi: 10.3390/molecules26164976 PMC8400614 34443563

[13] Amato A, Caldara G-F, Nuzzo D, Baldassano S, Picone P, Rizzo M, et al. NAFLD and Atherosclerosis Are Prevented by a Natural Dietary Supplement Containing Curcumin, Silymarin, Guggul, Chlorogenic Acid and Inulin in Mice Fed a High-Fat Diet. Nutrients. 2017;9(5):492. doi: 10.3390/nu9050492 28505074 PMC5452222

[14] Guo GH. Dictionary of Clinical Chinese Medicine. 2nd ed. Hunan Science and Technology Press. 2007.

[15] Liu DW. Yunnan Chinese Herbal Medicine. 40th anniversary ed. The People’s Press of Yunnan. 2000.

[16] Tu HS, Qiu JQ, Xu Y, Zhang J, Qiu SH, Huang ZH. Rapid discovery of new derivatives of short-sequence bushy-leaved albicans based on UHPLC-Q-Exactive-MS technique. Anal Test. 2022;41(07):988–97. doi: 10.19969/j.fxcsxb.21110405

[17] Qiu J, Chen XL, Liang PL, Zhang L, Xu Y, Gong M. Integrating approach to discover novel bergenin derivatives and phenolics with antioxidant and anti-inflammatory activities from bio-active fraction of Syzygium brachythyrsum. Arab J Chem. 2022;15:103507. doi: 10.1016/j.arabjc.2021.103507

[18] Chen X-L, Liang P-L, Gong M-J, Xu Y, Zhang L, Qiu X-H, et al. Polyphenolics from Syzygium brachythyrsum Inhibits Oxidized Low-Density Lipoprotein-Induced Macrophage-Derived Foam Cell Formation and Inflammation. Foods. 2022;11(21):3543. doi: 10.3390/foods11213543 36360156 PMC9656637

[19] Bence KK, Birnbaum MJ. Metabolic drivers of non-alcoholic fatty liver disease. Mol Metab. 2021;50:101143. doi: 10.1016/j.molmet.2020.101143 33346069 PMC8324696

[20] Ma L, Dai J, Chen J, Cai H-W, Li J-Y, Li X-Y, et al. Research Progress of Angiogenesis in Atherosclerotic Plaque in Chinese Medicine and Western Medicine. Chin J Integr Med. 2018;24(12):950–5. doi: 10.1007/s11655-018-2569-2 30178090

[21] Zhi W, Liu Y, Wang X, Zhang H. Recent advances of traditional Chinese medicine for the prevention and treatment of atherosclerosis. J Ethnopharmacol. 2023;301:115749. doi: 10.1016/j.jep.2022.115749 36181983

[22] Liang L, Yang W. Bergenin ameliorates the progression of atherosclerosis by inhibiting oxidative stress, inflammation, and monocytes adhesion in human umbilical vein endothelial cells. Mol Cell Toxicol. 2024. doi: 10.1007/s13273-024-00428-8

[23] Ding Y, Zhang B, Zhou K, Chen M, Wang M, Jia Y, et al. Dietary ellagic acid improves oxidant-induced endothelial dysfunction and atherosclerosis: role of Nrf2 activation. Int J Cardiol. 2014;175(3):508–14. doi: 10.1016/j.ijcard.2014.06.045 25017906

[24] Maguire EM, Pearce SWA, Xiao Q. Foam cell formation: A new target for fighting atherosclerosis and cardiovascular disease. Vascul Pharmacol. 2019;112:54–71. doi: 10.1016/j.vph.2018.08.002 30115528

[25] Baird L, Yamamoto M. The Molecular Mechanisms Regulating the KEAP1-NRF2 Pathway. Mol Cell Biol. 2020;40(13):e00099–20. doi: 10.1128/MCB.00099-20 32284348 PMC7296212

[26] Martin-Hurtado A, Martin-Morales R, Robledinos-Antón N, Blanco R, Palacios-Blanco I, Lastres-Becker I, et al. NRF2-dependent gene expression promotes ciliogenesis and Hedgehog signaling. Sci Rep. 2019;9(1):13896. doi: 10.1038/s41598-019-50356-0 31554934 PMC6761261

[27] Pajares M, Rojo AI, Arias E, Díaz-Carretero A, Cuervo AM, Cuadrado A. Transcription factor NFE2L2/NRF2 modulates chaperone-mediated autophagy through the regulation of LAMP2A. Autophagy. 2018;14(8):1310–22. doi: 10.1080/15548627.2018.1474992 29950142 PMC6103698

[28] Alanazi ST, Harisa GI, Salama SA. Modulating SIRT1, Nrf2, and NF-κB signaling pathways by bergenin ameliorates the cadmium-induced nephrotoxicity in rats. Chem Biol Interact. 2024;387:110797. doi: 10.1016/j.cbi.2023.110797 37949422

[29] Luo X, Weng X, Bao X, Bai X, Lv Y, Zhang S, et al. A novel anti-atherosclerotic mechanism of quercetin: Competitive binding to KEAP1 via Arg483 to inhibit macrophage pyroptosis. Redox Biol. 2022;57:102511. doi: 10.1016/j.redox.2022.102511 36274522 PMC9596875

[30] Naghibi N, Sadeghi A, Movahedinia S, Rahimi Naiini M, Rajizadeh MA, Bahri F, et al. Ellagic acid ameliorates aging-induced renal oxidative damage through upregulating SIRT1 and NRF2. BMC Complement Med Ther. 2023;23(1):77. doi: 10.1186/s12906-023-03907-y 36899375 PMC9999491

