# Supplementary material for: Polyphenol extract of Syzygium brachythyrsum mitigates atherosclerosis in high-fat diet induced ApoE-/- mice by regulating ROS/Keap1/Nrf2 pathway
Source: PLoS One. 2026 May 5;21(5):e0347758. doi: 10.1371/journal.pone.0347758 (PMC13143111; doi:10.1371/journal.pone.0347758)
Supplement: S1 File — (DOCX) [file pone.0347758.s001.docx]

Table S1. Mass spectrum information of the major components in the PSB

| No. | Name | RT  (min) | Molecular formula | [M-H]^-^ | Error  (ppm) | Fragment[negative] |
| --- | --- | --- | --- | --- | --- | --- |
|  | **Bergenin derivatives** |  |  |  |  |  |
| 2 | Bergenin | 8.5 | C_14_H_16_O_9_ | 327.07196 | -0.6 | 312.0483，249.0403，234.0163，207.0288，192.0054 |
| 12 | Brachythol A | 14.6 | C_21_H_20_O_13_ | 479.08350 | 0.8 | 327.0713，312.0480，271.0460，249.0401，234.0162，207.0291，169.0131 |
| 23 | Di-galloyl-*O*-bergenin | 17.8 | C_28_H_24_O_17_ | 631.09528 | 0.2 | 479.0837，327.0724，249.0401，207.0292，193.0132，169.0132 |
| 26 | Methxygalloyl-*O*-bergenin | 18.2 | C_22_H_22_O_13_ | 493.09927 | 1.0 | 327.0709，207.0289，249.0400，192.0054 |
| 28 | Di-galloyl-*O*-bergenin | 18.7 | C_28_H_24_O_17_ | 631.09412 | 0.1 | 479.0832，327.0720，249.0400，207.0288，193.0132，169.0131 |
| 30 | Di-galloyl-*O*-bergenin | 19.1 | C_28_H_24_O_17_ | 631.09418 | 0.2 | 479.0826，327.0721，249.0401，207.0289，193.0132，169.0132 |
| 31 | Hydroxybenzoyl-*O*-bergenin | 19.5 | C_21_H_30_O_11_ | 447.09344 | 0.3 | 327.0715，249.0400，234.0163，207.0289，192.0054 |
| 32 | Caffeoyl-*O*-bergenin | 19.6 | C_23_H_22_O_12_ | 489.10422 | 0.8 | 327.0712，313.0558，249.0400，207.0289 |
| 34 | Syringyl-*O*-bergenin | 20.0 | C_23_H_24_O_13_ | 507.11465 | 0.5 | 327.0699，299.0722，249.0401，234.0165，271.0463，207.0290，192.0054 |
| 35 | Coumaroyl-*O*-bergenin | 21.2 | C_23_H_22_O_11_ | 473.10928 | 0.7 | 327.0721，249.0403，312.0488，234.0162，207.0289，192.0054 |
| 36 | Sinapoyl-*O*-bergenin | 21.4 | C_25_H_26_O_13_ | 533.13031 | 0.5 | 327.0716，312.0487，249.0403，234.0164，207.0289，192.0054 |
| 37 | Feruloyl-*O*-bergenin | 21.6 | C_24_H_24_O_12_ | 503.11987 | 0.7 | 327.0724，249.0401，234.0164，207.0289，192.0053 |
| 38 | Bergenin derivative | 21.7 | C_26_H_32_O_13_ | 551.17737 | 0.6 | 327.0717，249.0403，234.0163，207.0289，193.0132 |
|  | **Norbergenin derivatives** |  |  |  |  |  |
| 1 | Norbergenin | 4.8 | C_13_H_14_O_9_ | 313.05673 | 0.7 | 235.0241，207.0291，193.0132 |
| 5 | Galloyl-*O*-norbergenin | 10.6 | C_20_H_18_O_13_ | 465.06744 | -0.1 | 313.0563，295.0453，271.0469，235.0242，207.0288，193.0131，169.0131 |
| 7 | Galloyl-*O*-norbergenin | 12.6 | C_20_H_18_O_13_ | 465.06769 | 0.5 | 313.0563，295.0457，271.0460，235.0243，207.0288，193.0133，169.0133 |
| 15 | Methylgalloyl-*O*-norbergenin | 15.9 | C_21_H_20_O_13_ | 479.08356 | 0.9 | 313.0558，271.0457，235.0241，207.0289，193.0132，169.0132 |
| 17 | Norbergenin derivative | 16.1 | C_26_H_34_O_12_ | 537.19806 | 0.6 | 313.0556，271.0463，169.0131 |
| 18 | Salicylic acid-*O*-norbergenin | 16.5 | C_20_H_18_O_11_ | 433.07764 | 0.0 | 313.0563，235.0243，193.0133 |
| 19 | Norbergenin derivative | 16.5 | C_26_H_34_O_12_ | 537.19806 | 0.6 | 313.0564，271.0459，169.0132 |
| 22 | Caffeoyl-*O*-norbergenin | 16.9 | C_22_H_20_O_12_ | 475.08875 | 1.2 | 313.0563，235.0230，193.0131，207.0288，161.0231 |
| 33 | Feruloyl-*O*-norbergenin | 19.6 | C_23_H_22_O_12_ | 489.10422 | 0.8 | 313.0558，235.0238，207.0289，193.0132 |
|  | **Brachythol B derivatives** |  |  |  |  |  |
| 4 | Galloyl-HHDP-hexose | 10.5 | C_27_H_22_O_18_ | 633.07336 | 0.0 | 300.9987，275.0198，249.0407，169.0128 |
| 11 | Brachythol B | 14.2 | C_28_H_22_O_17_ | 629.07837 | -0.1 | 300.9988，275.0202 |
| 14 | HHDP-*O*-bergenin | 15.8 | C_28_H_22_O_17_ | 629.07855 | 0.2 | 300.9988，275.0197，249.0396 |
| 20 | Methyl galloyl-HHDP-hexose | 16.9 | C_28_H_24_O_18_ | 647.08911 | 0.2 | 300.9987，275.0199，257.0089 |
| 25 | Galloyl-*O*-brachythol B | 18.1 | C_35_H_26_O_21_ | 781.08954 | 0.2 | 300.9987，275.0199，249.0408，479.0783 |
| 29 | Methoxyl brachythol B | 19.0 | C_29_H_24_O_18_ | 659.08936 | 0.6 | 300.9991，249.0402 |
|  | **Flavonoids and phenolic acids** |  |  |  |  |  |
| 3 | Chlorogenic acid | 9.8 | C_16_H_18_O_9_ | 353.08746 | -1.0 | 191.0550，179.0340 |
| 6 | Methylethyl hexoside-*O*-gallate | 11.3 | C_16_H_22_O_10_ | 373.11362 | -1.1 | 169.0132，151.0024 |
| 8 | Coumaroyl-*O*-quinic acid | 12.6 | C_16_H_18_O_8_ | 337.09274 | -0.4 | 191.0551，173.0444，163.0390 |
| 9 | Feruloylquinic acid | 13.8 | C_17_H_20_O_9_ | 367.10321 | -0.7 | 191.0551，173.0445 |
| 10 | Methyl hexose-tri-*O*-gallate | 14.1 | C_28_H_26_O_18_ | 649.10474 | 0.2 | 497.0928，345.0819，271.0460，211.0238，169.0131 |
| 13 | Feruloylquinic acid | 15.5 | C_17_H_20_O_9_ | 367.10309 | -1.0 | 191.0551，173.0446 |
| 16 | Galloyl-*O*-di-methylgallate | 15.9 | C_23_H_18_O_13_ | 501.06555 | -3.8 | 486.0418，271.0461，211.0239，169.0132 |
| 21 | Ellagic acid | 16.9 | C_14_H_6_O_8_ | 300.99902 | 0.1 | 257.0093，229.0131 |
| 24 | Quercetin-*O*-pantose | 17.8 | C_20_H_18_O_11_ | 433.07755 | -0.2 | 301.0345 |
| 27 | Quercetin-*O*-rhamnoside | 18.5 | C_21_H_20_O_11_ | 447.09332 | 0.1 | 315.0145，284.0328 |
| 39 | Quercetin | 22.3 | C_15_H_10_O_7_ | 301.03555 | 0.6 | 273.0402，257.0452，178.9975，151.0025 |
| 40 | Kaempferol | 24.7 | C_15_H_10_O_6_ | 285.04059 | 0.5 | 257.0455，151.0025 |

Table S2. Data statistical result for oil red O staining of aortic bulk（‾$x\pm s$,n=4）

| Groups | normality test, *P* | HOV test, *P* | Proportion of gross plaque in aorta (%) | *F，P* | *P'* |
| --- | --- | --- | --- | --- | --- |
| control | 0.411 | 0.009 | 0.849±0.395 | 67.142,0.000 | 0.003^##^ |
| AS | 0.406 |  | 19.841±2.490 |  | — |
| statin | 0.466 |  | 6.636±2.350 |  | 0.003** |
| PT-L | 0.646 |  | 9.167±0.554 |  | 0.017* |
| PT-M | 0.744 |  | 7.396±0.844 |  | 0.008** |
| PT-H | 0.984 |  | 7.984±0.925 |  | 0.008** |

Table S3. Data statistical result for oil red O staining of aortic root

（‾$x\pm s$,n=4）

| Groups | normality test, *P* | HOV test, *P* | Proportion of gross plaque in aorta root (%) | *F，P* | *P'* |
| --- | --- | --- | --- | --- | --- |
| control | 0.481 | 0.060 | 0.299±0.030 | 28.572,0.000 | 0.000^###^ |
| AS | 0.347 |  | 19.865±2.695 |  | — |
| statin | 0.292 |  | 12.102±2.000 |  | 0.003** |
| PT-L | 0.955 |  | 12.188±2.270 |  | 0.003** |
| PT-M | 0.093 |  | 9.339±3.174 |  | 0.000*** |
| PT-H | 0.850 |  | 10.165±2.619 |  | 0.000*** |

Table S4. Data statistical result for lipid levels in serum of AS mice（‾$x\pm s$,n=6）

| Lipids | Groups | normality test, *P* | HOV test, *P* | lipid levels (mmol/L) | *F，P* | *P'* |
| --- | --- | --- | --- | --- | --- | --- |
| TG | control | 0.312 | 0.004 | 0.541±0.061 | 12.164,0.000 | 0.025^#^ |
|  | AS | 0.213 |  | 0.965±0.198 |  | — |
|  | statin | 0.775 |  | 0.660±0.112 |  | 0.114 |
|  | PT-L | 0.185 |  | 0.594±0.097 |  | 0.043* |
|  | PT-M | 0.386 |  | 0.578±0.067 |  | 0.037* |
|  | PT-H | 0.656 |  | 0.619±0.040 |  | 0.064 |
| TC | control | 0.155 | 0.006 | 2.046±0.100 | 70.501,0.000 | 0.000^###^ |
|  | AS | 0.336 |  | 22.257±2.194 |  | — |
|  | statin | 0.310 |  | 18.145±2.692 |  | 0.169 |
|  | PT-L | 0.583 |  | 20.804±1.770 |  | 0.938 |
|  | PT-M | 0.433 |  | 19.573±3.253 |  | 0.747 |
|  | PT-H | 0.612 |  | 19.777±1.715 |  | 0.450 |
| HDL-C | control | 0.297 | 0.822 | 3.826±0.530 | 13.077,0.000 | 0.000^###^ |
|  | AS | 0.579 |  | 1.486±0.587 |  | — |
|  | statin | 0.728 |  | 1.950±0.691 |  | 1.000 |
|  | PT-L | 0.205 |  | 2.744±0.564 |  | 0.008** |
|  | PT-M | 0.086 |  | 2.266±0.554 |  | 0.343 |
|  | PT-H | 0.437 |  | 3.016±0.417 |  | 0.001** |
| LDL-C | control | 0.466 | 0.074 | 5.715±0.933 | 110.932,0.000 | 0.000^###^ |
|  | AS | 0.884 |  | 45.521±2.104 |  | — |
|  | statin | 0.713 |  | 38.529±2.882 |  | 0.010* |
|  | PT-L | 0.191 |  | 37.058±3.729 |  | 0.001** |
|  | PT-M | 0.226 |  | 31.749±5.384 |  | 0.000*** |
|  | PT-H | 0.181 |  | 32.059±2.151 |  | 0.000*** |

Table S5. Data statistical result for lipid levels in liver of AS mice（‾$x\pm s$,n=6）

| Lipids | Groups | normality test, *P* | HOV test, *P* | lipid levels (mmol/L) | *F，P* | *P'* |
| --- | --- | --- | --- | --- | --- | --- |
| TG | control | 0.521 | 0.010 | 4.235±0.976 | 27.094,0.000 | 0.000^###^ |
|  | AS | 0.648 |  | 8.701±1.054 |  | — |
|  | statin | 0.873 |  | 4.734±0.540 |  | 0.001** |
|  | PT-L | 0.656 |  | 4.494±0.388 |  | 0.001** |
|  | PT-M | 0.480 |  | 4.184±0.347 |  | 0.001** |
|  | PT-H | 0.994 |  | 6.451±1.283 |  | 0.090 |
| TC | control | 0.600 | 0.112 | 5.575±0.608 | 28.073,0.000 | 0.000^###^ |
|  | AS | 0.117 |  | 10.742±0.854 |  | — |
|  | statin | 0.409 |  | 6.050±0.437 |  | 0.000*** |
|  | PT-L | 0.808 |  | 5.389±1.508 |  | 0.000*** |
|  | PT-M | 0.536 |  | 4.849±1.365 |  | 0.000*** |
|  | PT-H | 0.090 |  | 5.645±0.798 |  | 0.000*** |

Table S6. Data statistical result for Inflammatory factor levels in serum.（‾$x\pm s$,n=6）

| Inflammatory factors | Groups | normality test, *P* | HOV test, *P* | levels (mmol/L) | *F，P* | *P'* |
| --- | --- | --- | --- | --- | --- | --- |
| IL-1β | control | 0.487 | 0.056 | 5.300±2.366 | 25.416,0.000 | 0.000^###^ |
|  | AS | 0.239 |  | 20.707±3.371 |  | — |
|  | statin | 0.486 |  | 14.661±3.423 |  | 0.004** |
|  | PT-L | 0.411 |  | 15.035±1.925 |  | 0.007** |
|  | PT-M | 0.294 |  | 13.369±1.292 |  | 0.000*** |
|  | PT-H | 0.394 |  | 10.180±1.933 |  | 0.000*** |
| IL-6 | control | 0.995 | 0.000 | 9.946±0.727 | 53.860,0.000 | 0.001^##^ |
|  | AS | 0.159 |  | 40.732±7.052 |  | — |
|  | statin | 0.244 |  | 36.395±2.633 |  | 0.872 |
|  | PT-L | 0.561 |  | 37.455±4.318 |  | 0.989 |
|  | PT-M | 0.118 |  | 26.752±1.963 |  | 0.036* |
|  | PT-H | 0.206 |  | 35.928±2.703 |  | 0.805 |
| TNF-α | control | 0.501 | 0.639 | 11.456±0.970 | 16.939,0.000 | 0.031^#^ |
|  | AS | 0.417 |  | 13.433±1.398 |  | — |
|  | statin | 0.530 |  | 10.260±0.895 |  | 0.000*** |
|  | PT-L | 0.199 |  | 13.881±1.107 |  | 1.000 |
|  | PT-M | 0.573 |  | 14.523±0.743 |  | 1.000 |
|  | PT-H | 0.533 |  | 11.242±0.844 |  | 0.012* |

Table S7. Data statistical result for Fluorescence staining of ROS and immunofluorescence of CD36 in the aortic root（‾$x\pm s$,n=4）

|  | Groups | normality test, *P* | HOV test, *P* | Mean Fluorescence Intensity | *F，P* | *P'* |
| --- | --- | --- | --- | --- | --- | --- |
| CD36 | control | 0.131 | 0.008 | 55.191±3.562 | 25.067,0.000 | 0.035^#^ |
|  | AS | 0.830 |  | 110.247±16.279 |  | — |
|  | statin | 0.106 |  | 57.609±3.967 |  | 0.039* |
|  | PT-L | 0.086 |  | 68.538±8.465 |  | 0.063 |
|  | PT-M | 0.147 |  | 63.089±7.363 |  | 0.043* |
|  | PT-H | 0.457 |  | 56.312±2.252 |  | 0.040* |
| ROS | control | 0.948 | 0.017 | 49.518±6.738 | 29.364,0.000 | 0.015^#^ |
|  | AS | 0.855 |  | 126.792±19.123 |  | — |
|  | statin | 0.086 |  | 65.162±8.011 |  | 0.030* |
|  | PT-L | 0.894 |  | 80.561±4.931 |  | 0.090 |
|  | PT-M | 0.180 |  | 74.182±2.868 |  | 0.067 |
|  | PT-H | 0.571 |  | 66.405±8.096 |  | 0.032* |

Table S8. Data statistical result for Liver oxidative stress markers of GSH-Px, SOD, and MDA（‾$x\pm s$,n=6）

|  | Groups | normality test, *P* | HOV test, *P* | GSH-Px、SOD、MDA | *F，P* | *P'* |
| --- | --- | --- | --- | --- | --- | --- |
| GSH-Px  （U/mg prot） | control | 0.886 | 0.452 | 202.705±15.196 | 5.492,0.001 | 0.000^###^ |
|  | AS | 0.757 |  | 170.367±8.050 |  | — |
|  | statin | 0.573 |  | 186.390±11.208 |  | 0.317 |
|  | PT-L | 0.449 |  | 180.036±12.632 |  | 1.000 |
|  | PT-M | 0.875 |  | 186.620±11.932 |  | 0.293 |
|  | PT-H | 0.184 |  | 192.059±7.575 |  | 0.038* |
| SOD  （U/mg prot） | control | 0.492 | 0.377 | 40.188±1.731 | 16.425,0.000 | 0.000^###^ |
|  | AS | 0.425 |  | 29.315±2.659 |  | — |
|  | statin | 0.172 |  | 33.025±1.829 |  | 0.213 |
|  | PT-L | 0.092 |  | 30.319±2.570 |  | 1.000 |
|  | PT-M | 0.155 |  | 36.844±2.537 |  | 0.000*** |
|  | PT-H | 0.437 |  | 34.759±3.177 |  | 0.009** |
| MDA  （nmol/mg prot） | control | 0.958 | 0.040 | 0.769±0.052 | 29.057,0.000 | 0.061 |
|  | AS | 0.531 |  | 0.967±0.113 |  | — |
|  | statin | 0.274 |  | 0.706±0.053 |  | 0.015* |
|  | PT-L | 0.455 |  | 0.606±0.050 |  | 0.002** |
|  | PT-M | 0.284 |  | 0.590±0.048 |  | 0.002** |
|  | PT-H | 0.506 |  | 0.643±0.034 |  | 0.006** |

Table S9. Data statistical result for THP-1 cell viability by MTT assay（‾$x\pm s$,n=3）

| Groups | normality test, *P* | HOV test, *P* | cell viability（%） | *F，P* | *P'* |
| --- | --- | --- | --- | --- | --- |
| control | — | 0.057 | 100.000±0.000 | 21.487,0.000 | — |
| PT-2.5 μg/mL | 0.936 |  | 102.817±5.202 |  | 1.000 |
| PT-5 μg/mL | 0.220 |  | 98.732±3.494 |  | 1.000 |
| PT-10 μg/mL | 0.421 |  | 91.901±4.367 |  | 0.649 |
| PT-20 μg/mL | 0.073 |  | 82.088±3.911 |  | 0.001** |
| PT-40 μg/mL | 0.360 |  | 79.059±5.867 |  | 0.000*** |
| PT-80 μg/mL | 0.378 |  | 77.529±4.054 |  | 0.000*** |
| PT-160 μg/mL | 0.843 |  | 79.577±0.701 |  | 0.000*** |

Table S10. Data statistical result for NO release（‾$x\pm s$,n=3）

| Groups | normality test, *P* | HOV test, *P* | NO  (mmol/L) | *F，P* | *P'* |
| --- | --- | --- | --- | --- | --- |
| control | 0.795 | 0.057 | 0.795±0.140 | 53.700,0.000 | 0.000^###^ |
| ox-LDL | 0.230 |  | 3.863±0.280 |  | — |
| PT-L | 0.290 |  | 3.478±0.099 |  | 1.000 |
| PT-M | 0.315 |  | 2.995±0.269 |  | 0.036* |
| PT-H | 0.320 |  | 2.926±0.464 |  | 0.022* |

Table S11. Data statistical result for quantitative analysis plots of Keap1, Nrf2 and NQO1（‾$x\pm s$,n=3）

| Index | Group | normality test, *P* | HOV test, *P* | Protein expression level | *F，P* | *P'* |
| --- | --- | --- | --- | --- | --- | --- |
| Keap1 | control | — | 0.112 | 1.000±0.000 | 39.848,0.000 | 0.034^#^ |
|  | ox-LDL | 0.818 |  | 1.309±0.174 |  | — |
|  | PT-L | 0.994 |  | 1.231±0.077 |  | 1.000 |
|  | PT-M | 0.197 |  | 0.920±0.095 |  | 0.007** |
|  | PT-H | 0.762 |  | 0.392±0.062 |  | 0.000*** |
| Nrf2 | control | — | 0.048 | 1.000±0.000 | 13.992,0.000 | 0.019^#^ |
|  | ox-LDL | 0.614 |  | 0.376±0.077 |  | — |
|  | PT-L | 0.229 |  | 1.014±0.431 |  | 0.413 |
|  | PT-M | 0.470 |  | 1.778±0.130 |  | 0.002** |
|  | PT-H | 0.959 |  | 2.010±0.507 |  | 0.108 |
| NQO1 | control | — | 0.022 | 1.000±0.000 | 9.817,0.002 | 0.031^#^ |
|  | ox-LDL | 0.079 |  | 0.441±0.088 |  | — |
|  | PT-L | 0.269 |  | 0.923±0.012 |  | 0.039* |
|  | PT-M | 0.510 |  | 1.057±0.264 |  | 0.183 |
|  | PT-H | 0.947 |  | 1.056±0.159 |  | 0.045* |

Table S12. Data statistical result for quantitative analysis plots of Keap1, Nrf2 and NQO1 under ML385 inhibitor.（‾$x\pm s$,n=3）

| Index | Group | normality test, *P* | HOV test, *P* | Protein expression level | *F，P* | *P'* |
| --- | --- | --- | --- | --- | --- | --- |
| Keap1 | control | — | 0.052 | 1.000±0.000 | 19.110,0.001 | 0.004^##^ |
|  | ox-LDL | 0.190 |  | 1.401±0.128 |  | — |
|  | PT | 0.990 |  | 0.894±0.127 |  | 0.001** |
|  | PT+ML | 0.974 |  | 1.240±0.019 |  | 0.374 |
| Nrf2 | control | — | 0.162 | 1.000±0.000 | 11.978,0.003 | 0.009^##^ |
|  | ox-LDL | 0.987 |  | 0.500±0.087 |  | — |
|  | PT | 0.674 |  | 0.951±0.180 |  | 0.016* |
|  | PT+ML | 0.978 |  | 0.567±0.163 |  | 1.000 |

Note. All the data were normally distributed, and ANOVA was used for analysis. The homogeneity of variance test (HOV) yielded a *P*>0.05, and Bonferroni’s method was applied for multiple comparisons between groups. Compared with the control group, ^#^indicates *P*<0.05, ^##^indicates *P*<0.01, ^###^indicates *P*<0.001. Compared with the model group, * indicates *P*<0.05，** indicates *P*<0.01，*** indicates *P*<0.001.
