# Supplementary figures and images for: Polyphenol extract of Syzygium brachythyrsum mitigates atherosclerosis in high-fat diet induced ApoE-/- mice by regulating ROS/Keap1/Nrf2 pathway (part 1 of 4)
Source: PLoS One. 2026 May 5;21(5):e0347758. doi: 10.1371/journal.pone.0347758 (PMC13143111; doi:10.1371/journal.pone.0347758)

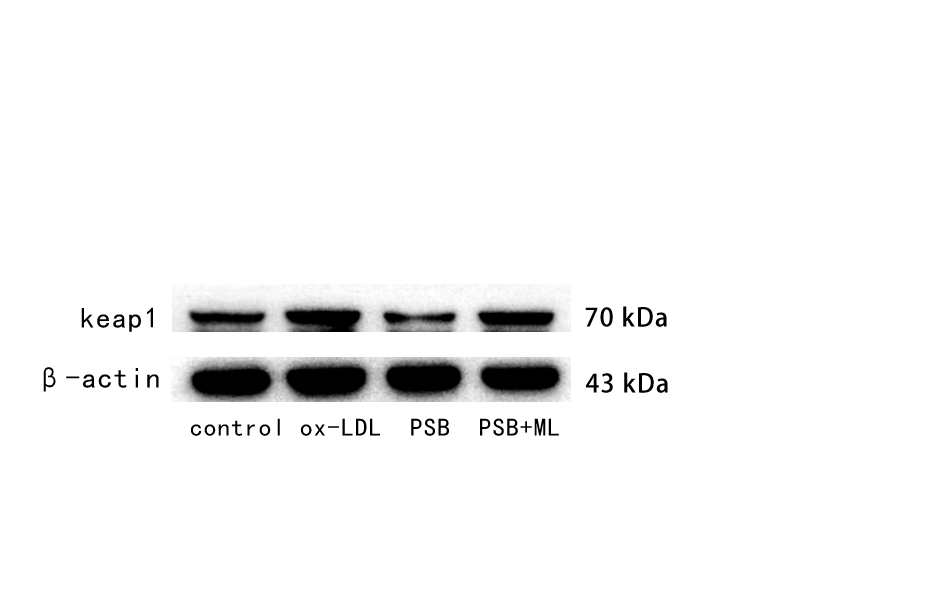

Supplement: S2 File — (ZIP) [file pone.0347758.s002.zip › FIG6B/keap1/0110-4 拷贝.tif]

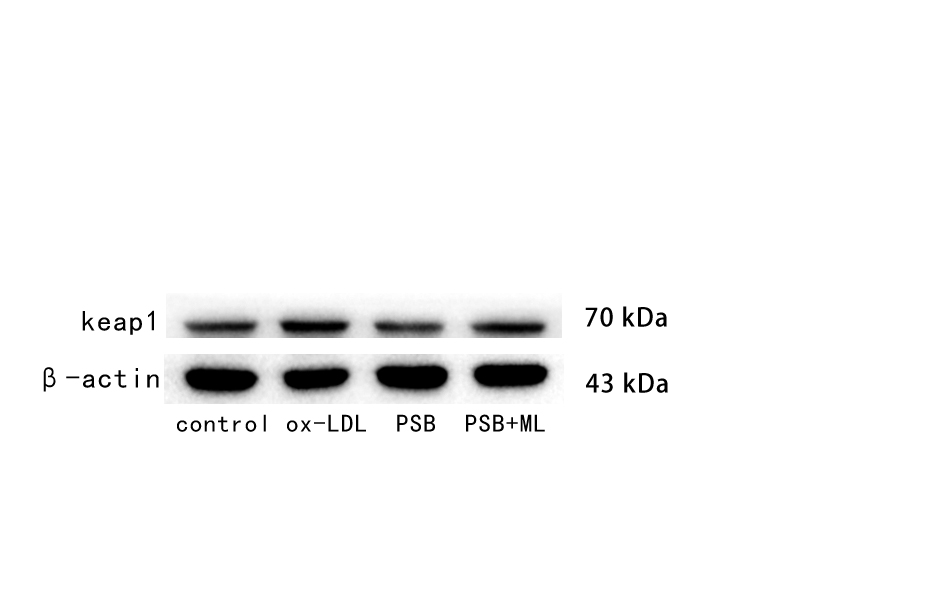

Supplement: S2 File — (ZIP) [file pone.0347758.s002.zip › FIG6B/keap1/0114-2 拷贝 2.tif]

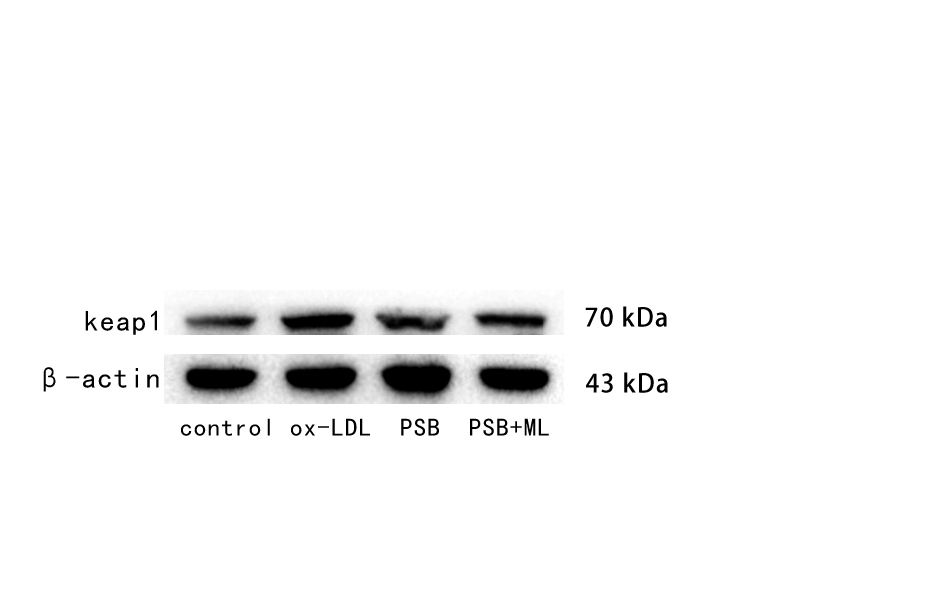

Supplement: S2 File — (ZIP) [file pone.0347758.s002.zip › FIG6B/keap1/0114-4 拷贝 2.tif]

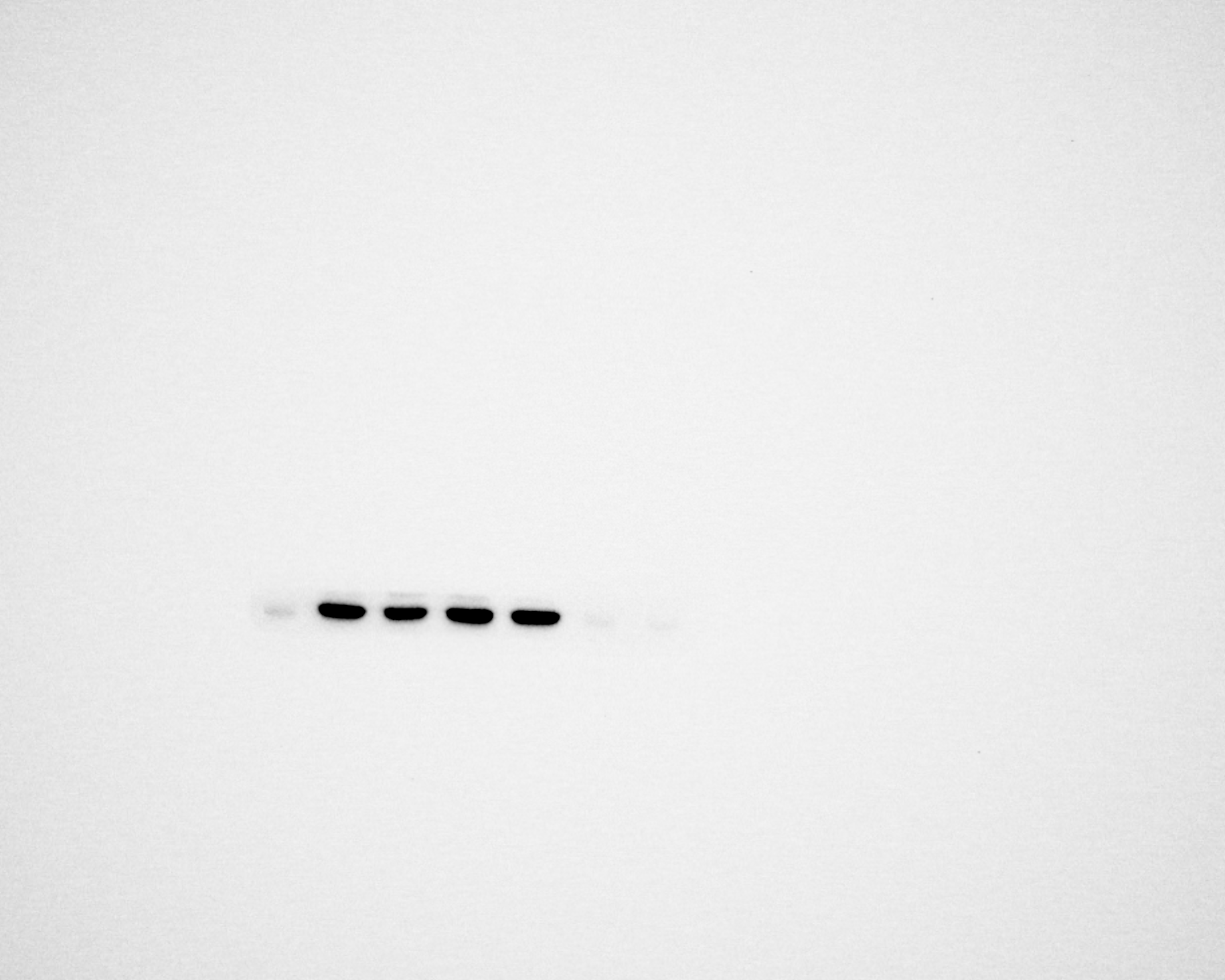

Supplement: S2 File — (ZIP) [file pone.0347758.s002.zip › FIG6B/keap1/2-actin-0114_2(Chemiluminescence).tif]

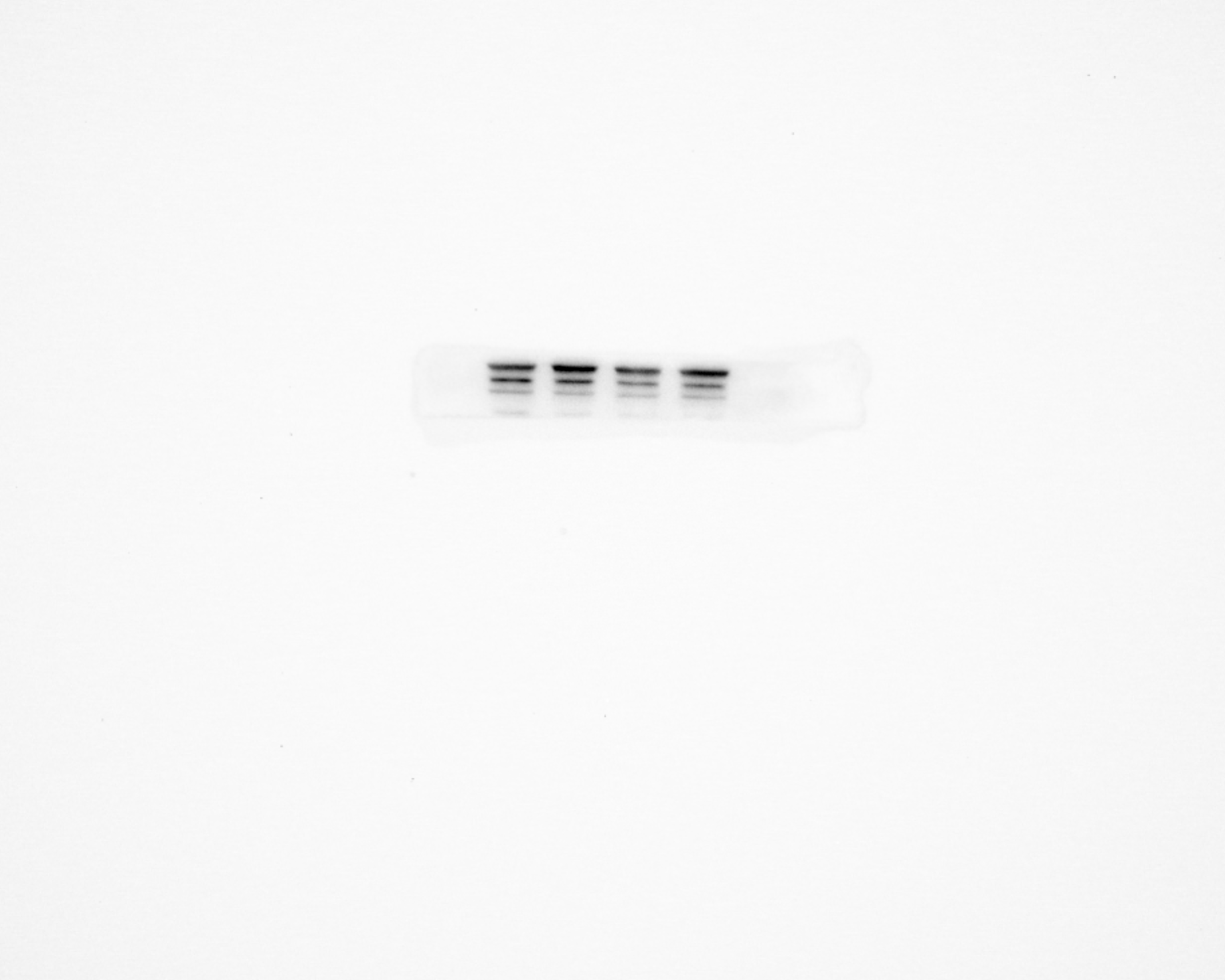

Supplement: S2 File — (ZIP) [file pone.0347758.s002.zip › FIG6B/keap1/2-keap-0114_3(Chemiluminescence).tif]

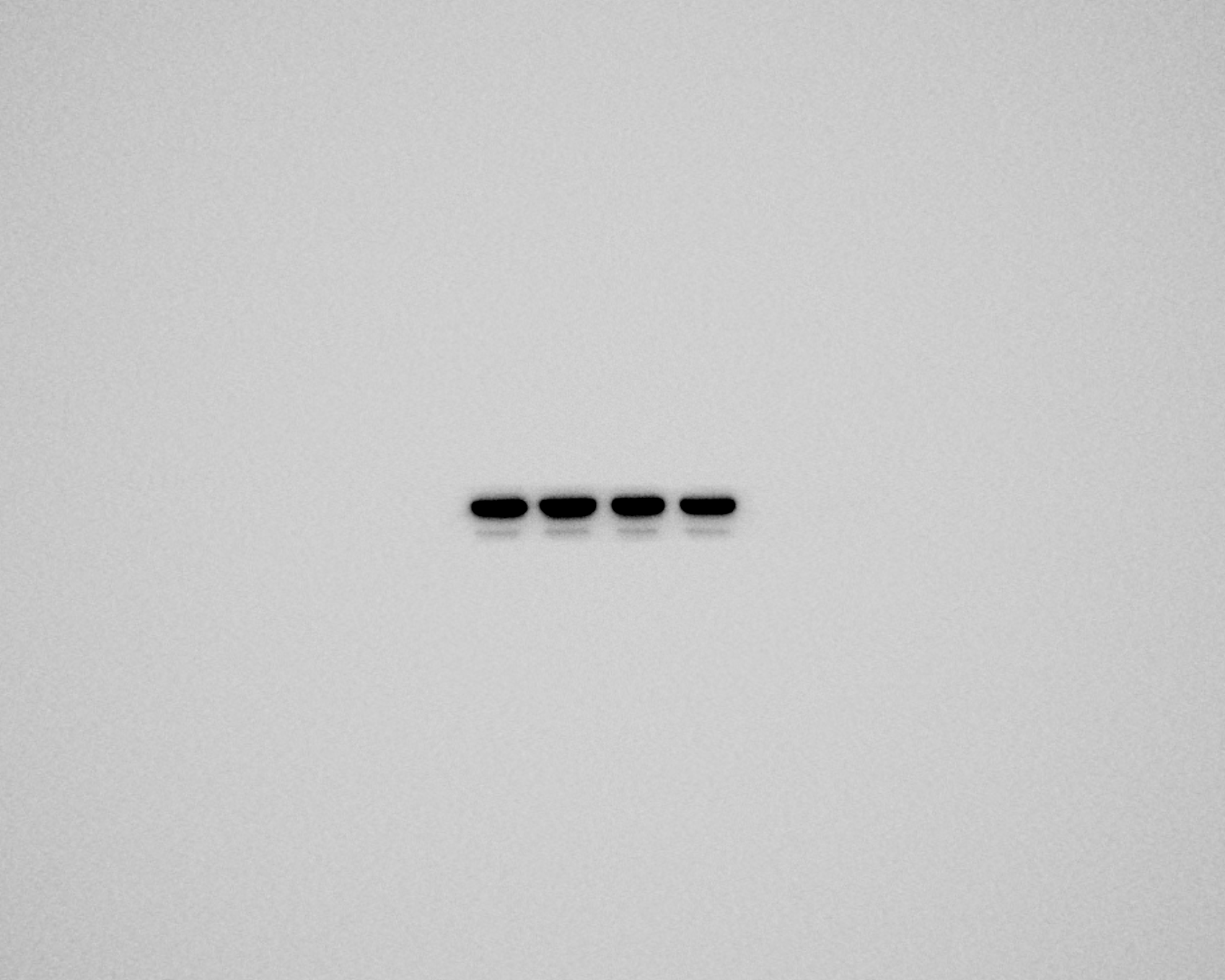

Supplement: S2 File — (ZIP) [file pone.0347758.s002.zip › FIG6B/keap1/4-actin-0110_1(Chemiluminescence).tif]

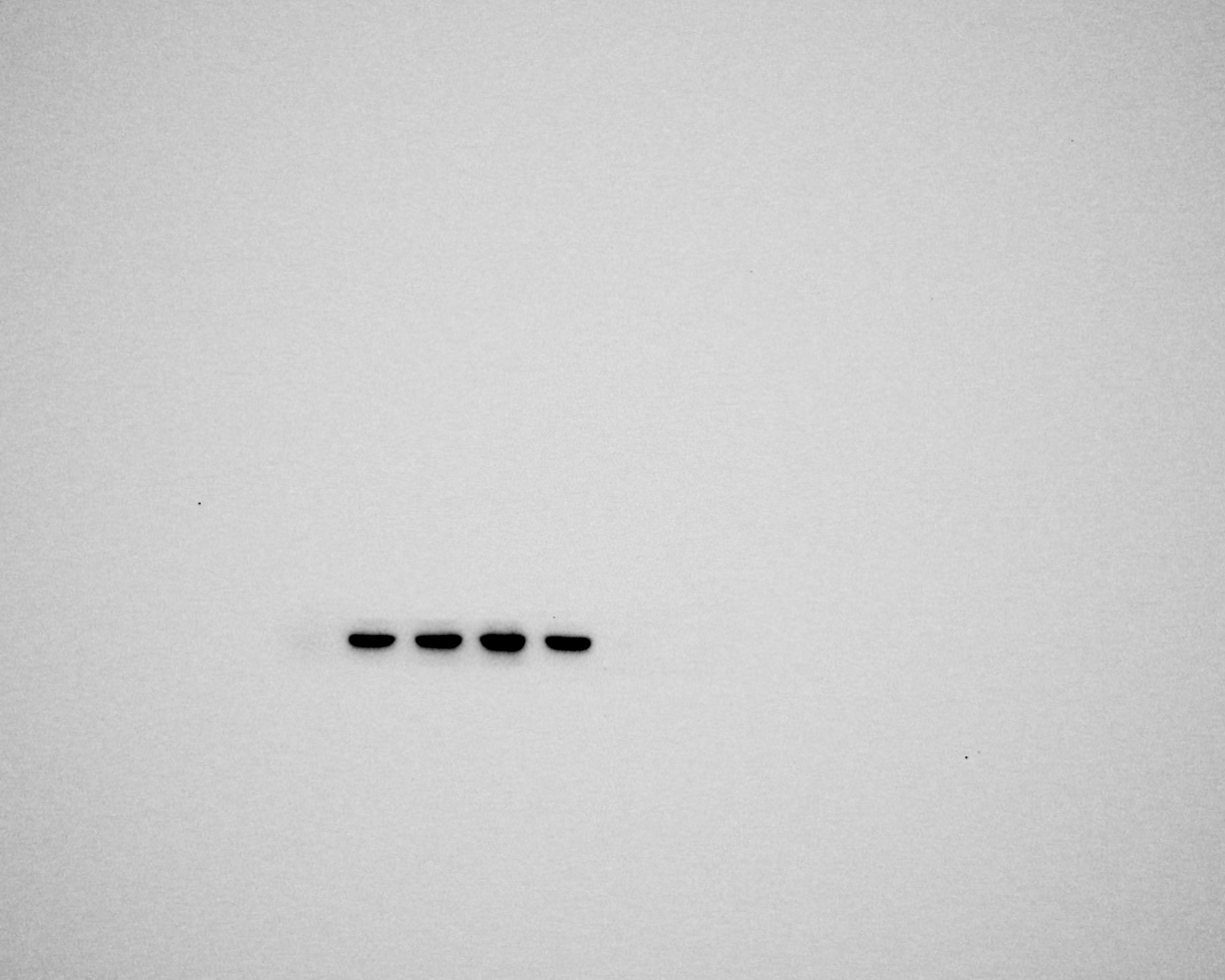

Supplement: S2 File — (ZIP) [file pone.0347758.s002.zip › FIG6B/keap1/4-actin-0114_1(Chemiluminescence).tif]

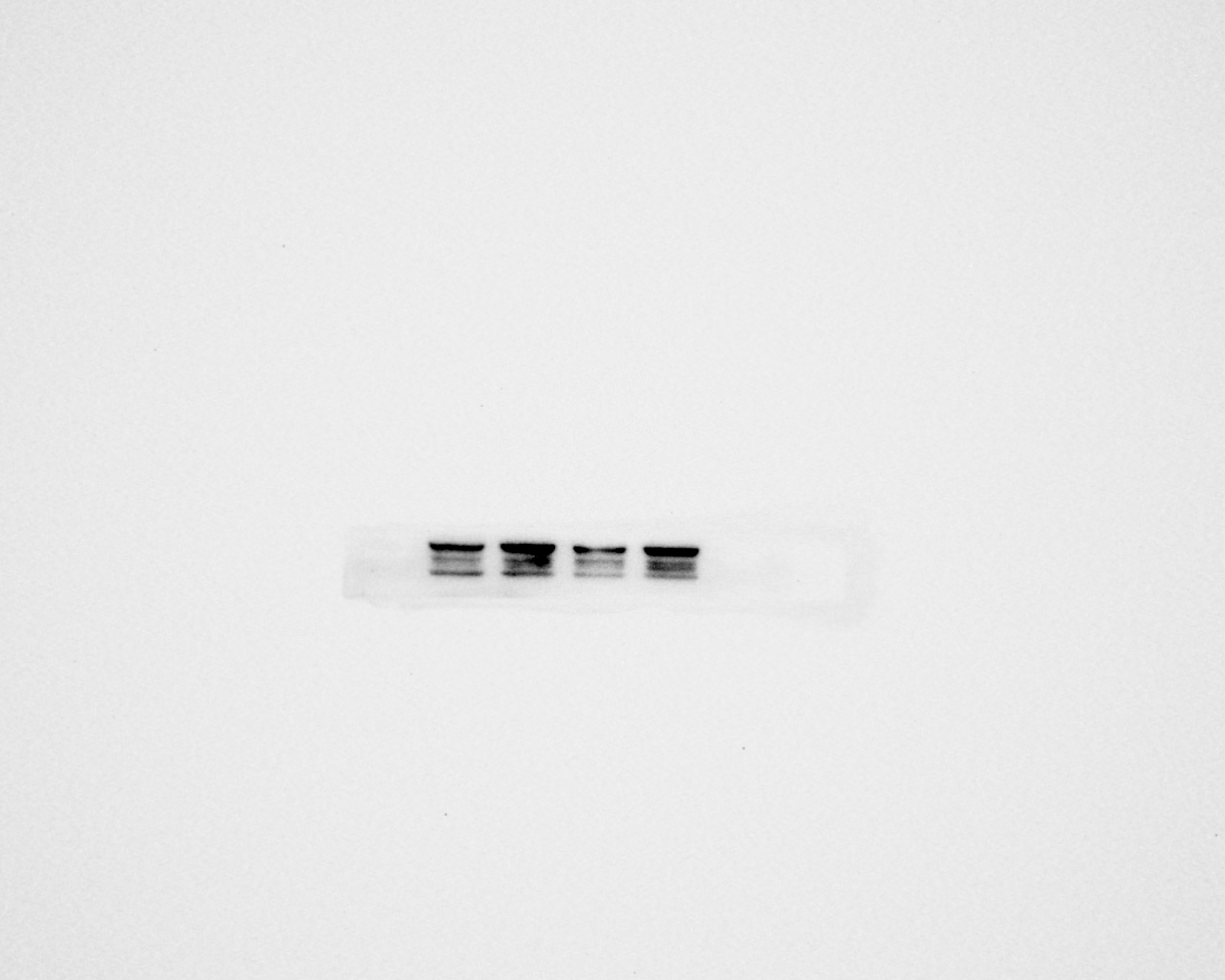

Supplement: S2 File — (ZIP) [file pone.0347758.s002.zip › FIG6B/keap1/4-keap-0110_2(Chemiluminescence).tif]

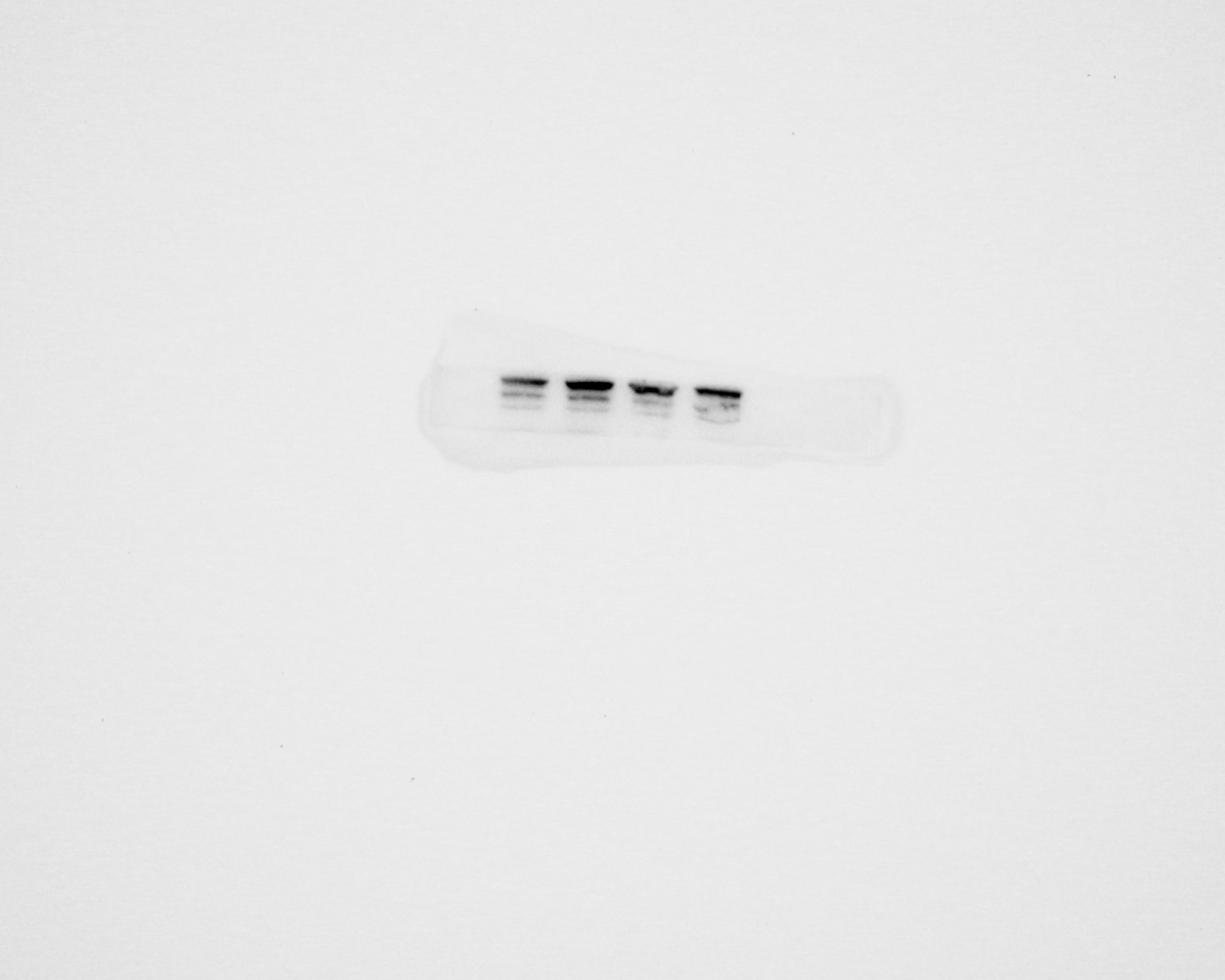

Supplement: S2 File — (ZIP) [file pone.0347758.s002.zip › FIG6B/keap1/4-keap-0114_3(Chemiluminescence).tif]

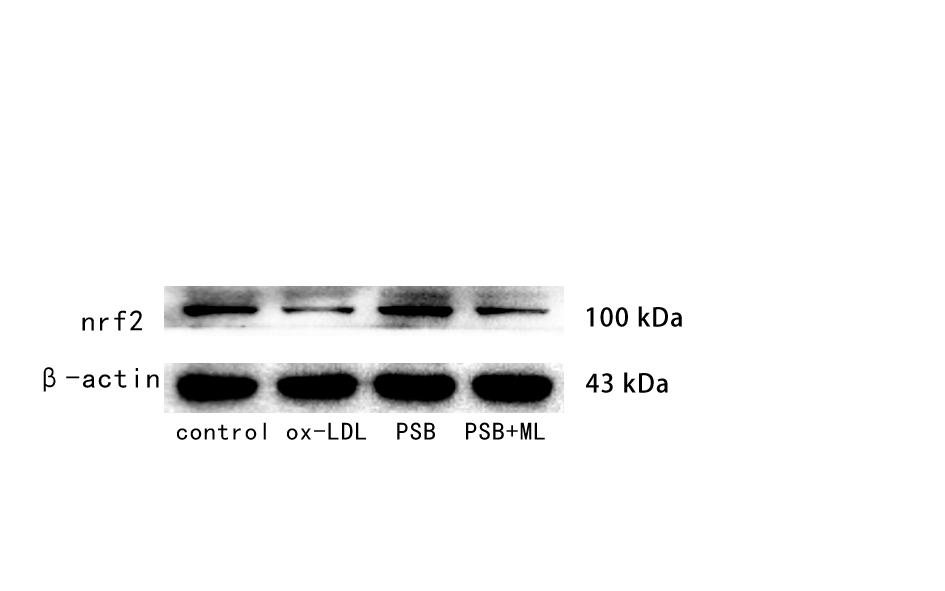

Supplement: S2 File — (ZIP) [file pone.0347758.s002.zip › FIG6B/nrf2/0116-2 拷贝 2.tif]

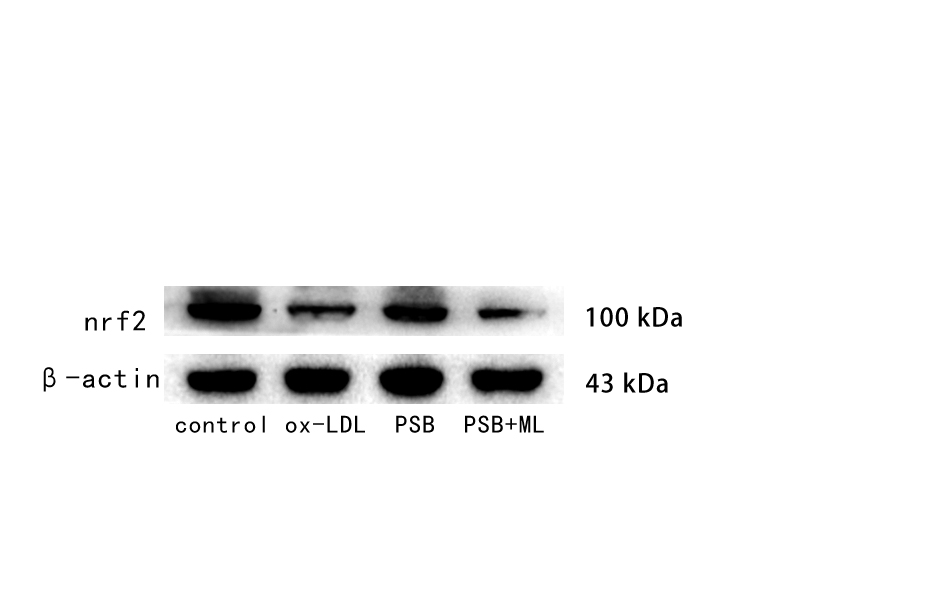

Supplement: S2 File — (ZIP) [file pone.0347758.s002.zip › FIG6B/nrf2/0118-1 拷贝 2.tif]

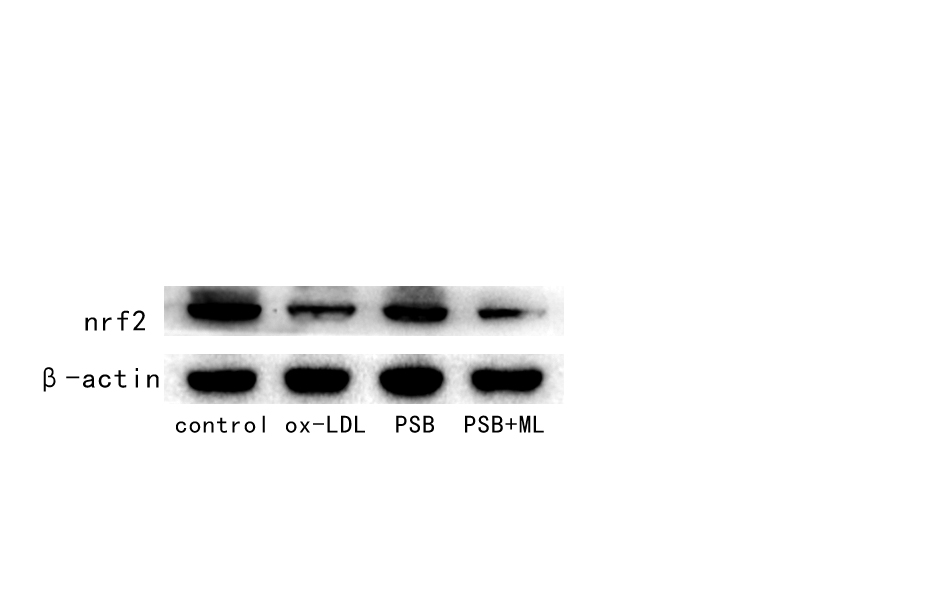

Supplement: S2 File — (ZIP) [file pone.0347758.s002.zip › FIG6B/nrf2/0118-1 拷贝.jpg]

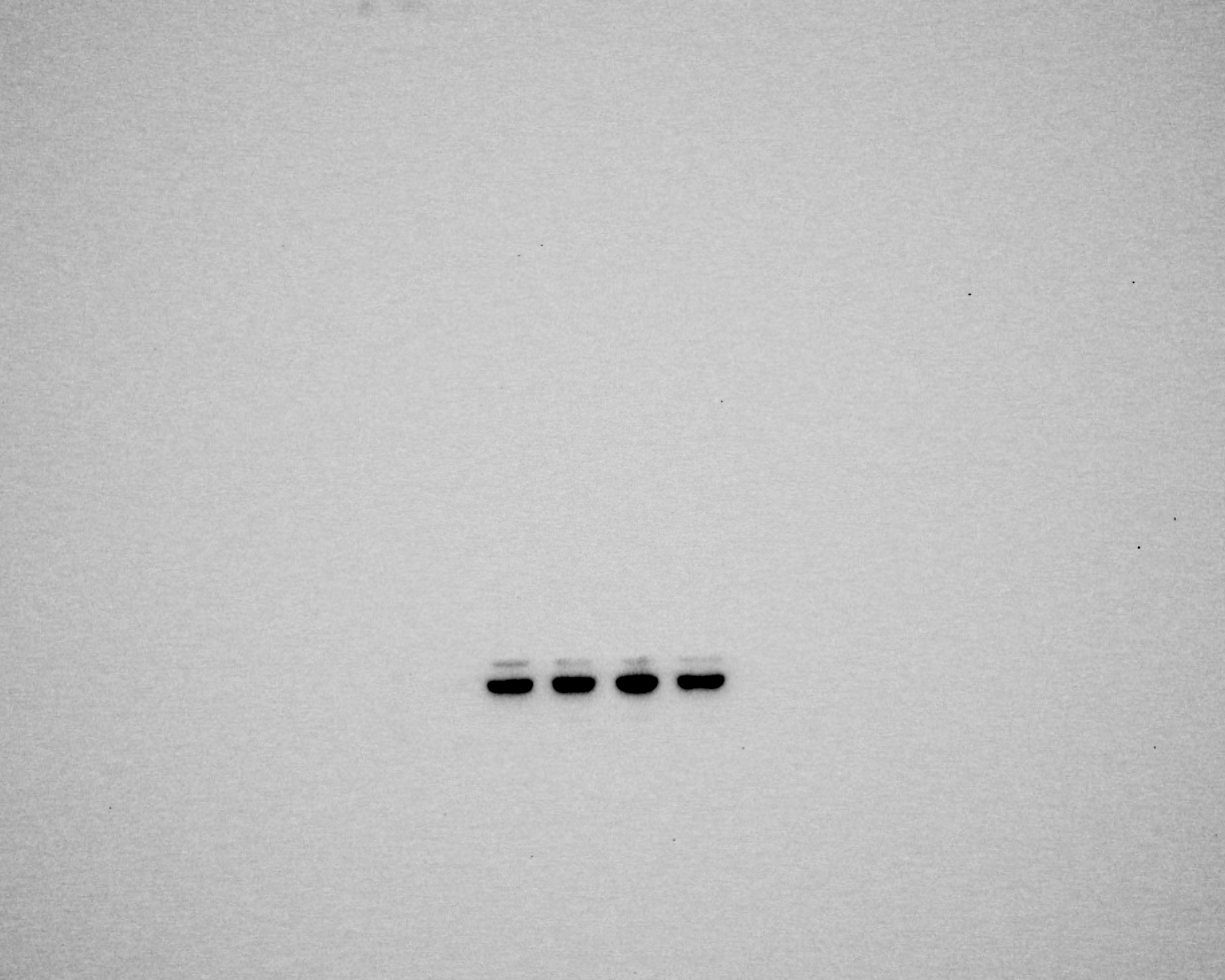

Supplement: S2 File — (ZIP) [file pone.0347758.s002.zip › FIG6B/nrf2/1-actin-0118_2(Chemiluminescence).tif]

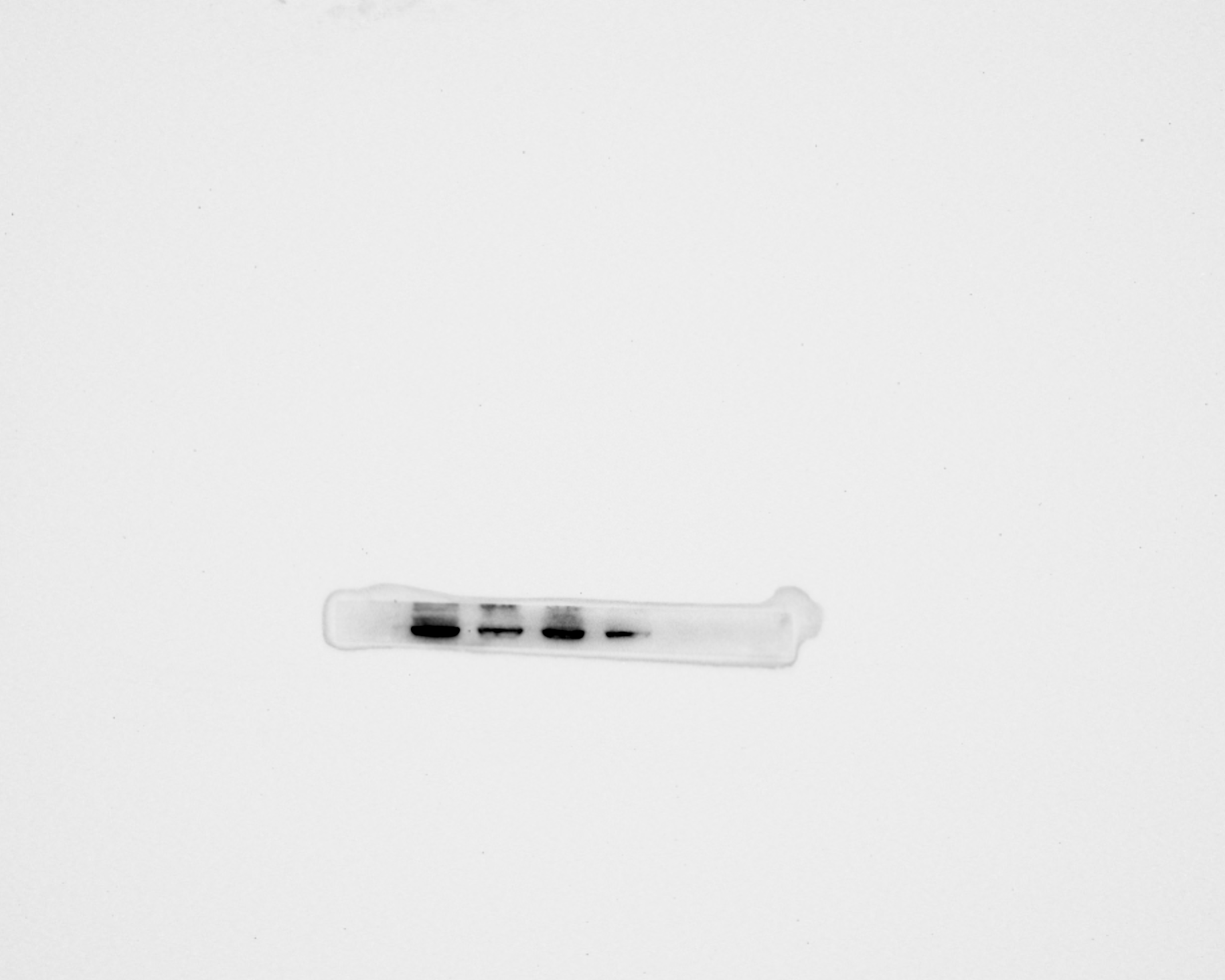

Supplement: S2 File — (ZIP) [file pone.0347758.s002.zip › FIG6B/nrf2/1-nrf-0118_1(Chemiluminescence).tif]

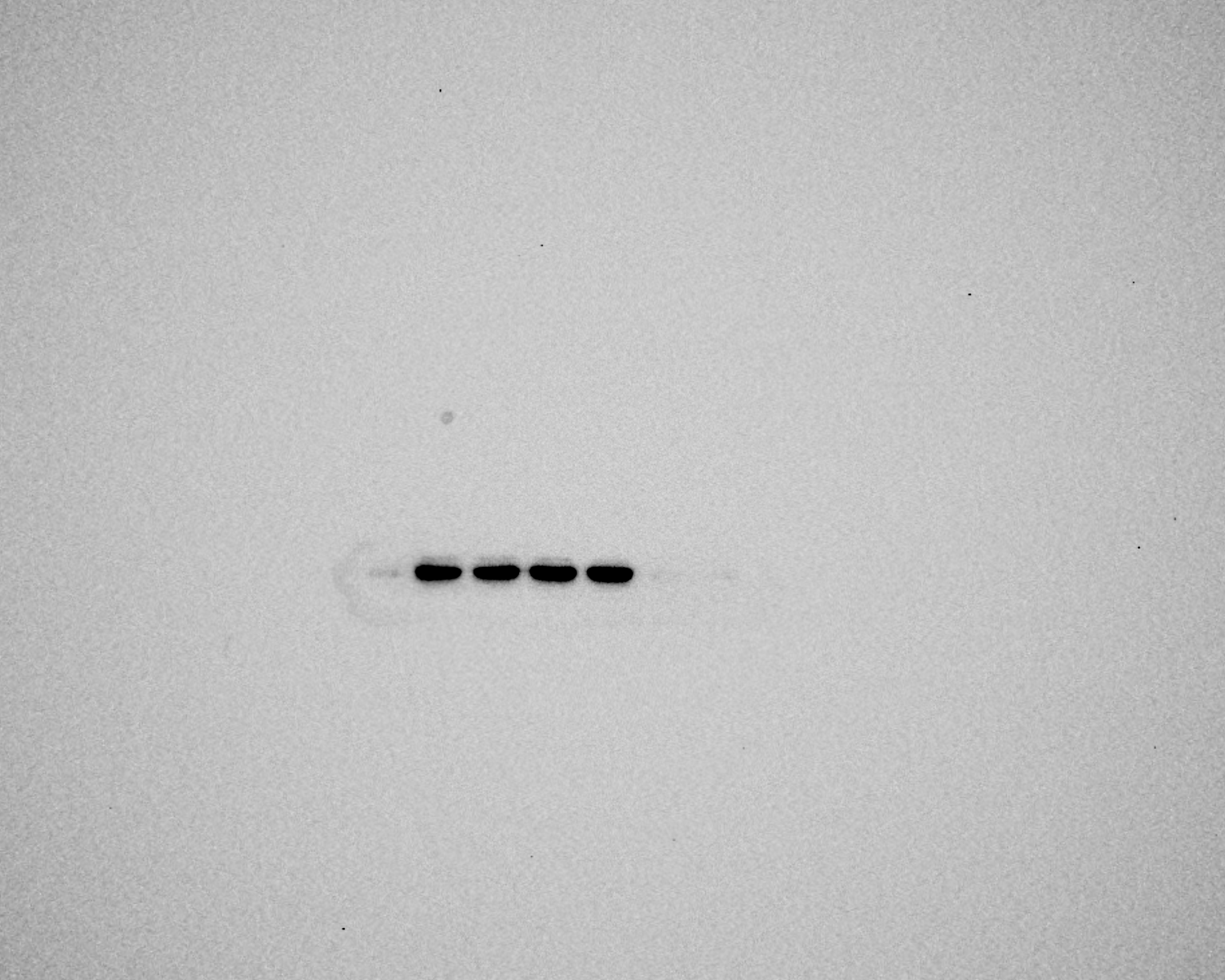

Supplement: S2 File — (ZIP) [file pone.0347758.s002.zip › FIG6B/nrf2/2-actin-0116_2(Chemiluminescence).tif]

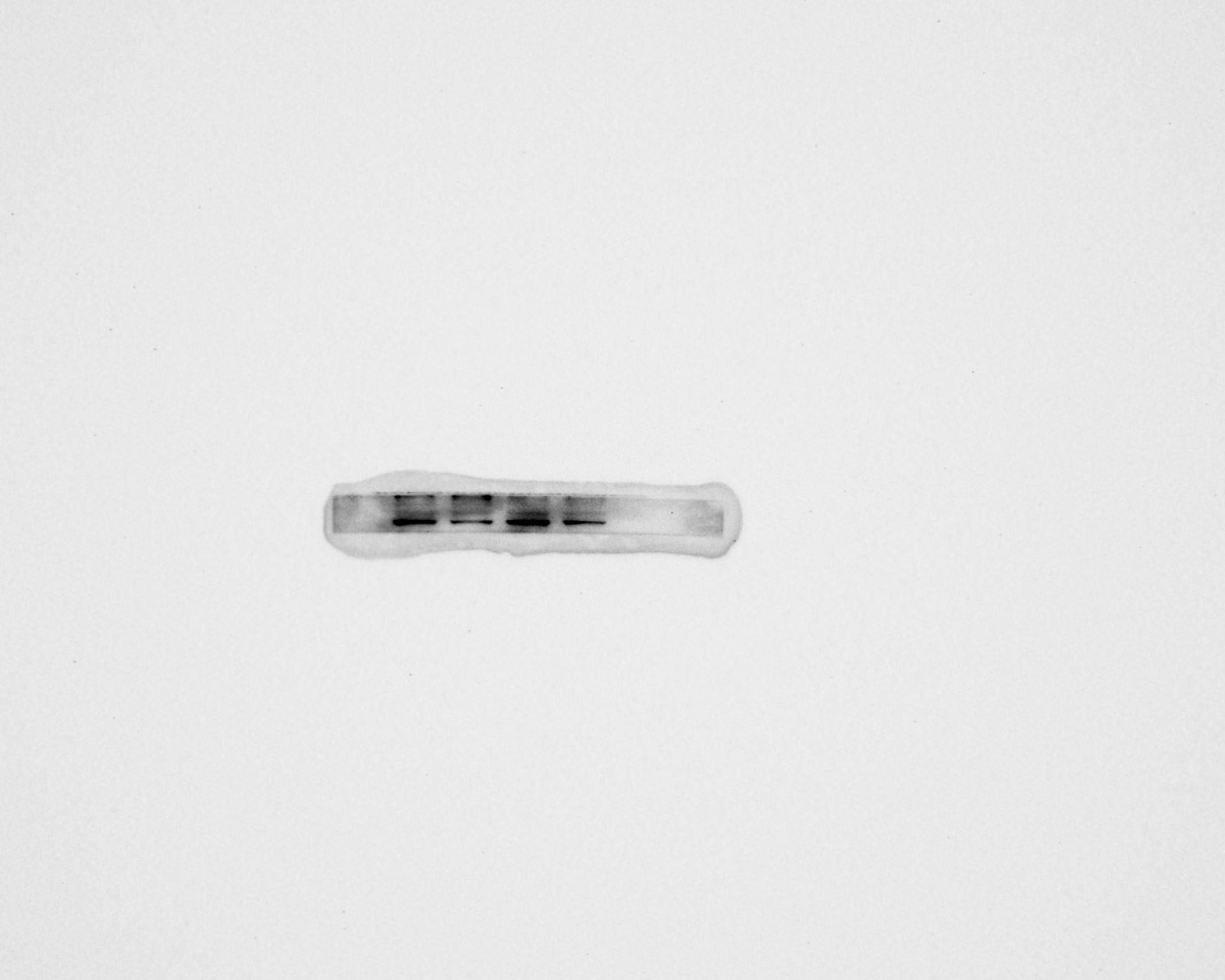

Supplement: S2 File — (ZIP) [file pone.0347758.s002.zip › FIG6B/nrf2/2-nrf-0116_3(Chemiluminescence).tif]

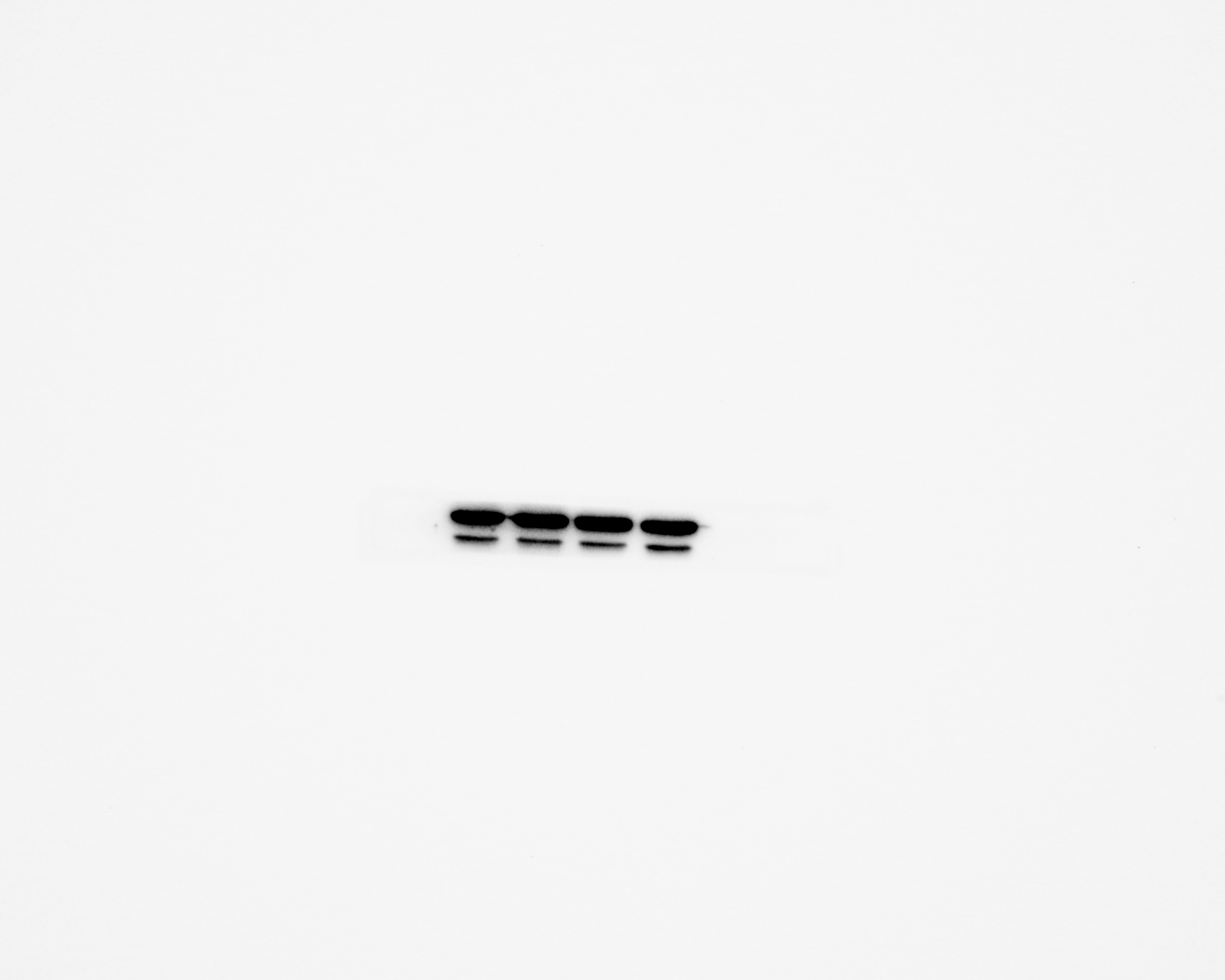

Supplement: S2 File — (ZIP) [file pone.0347758.s002.zip › FIG6B/nrf2/3-actin-0112_1(Chemiluminescence).tif]

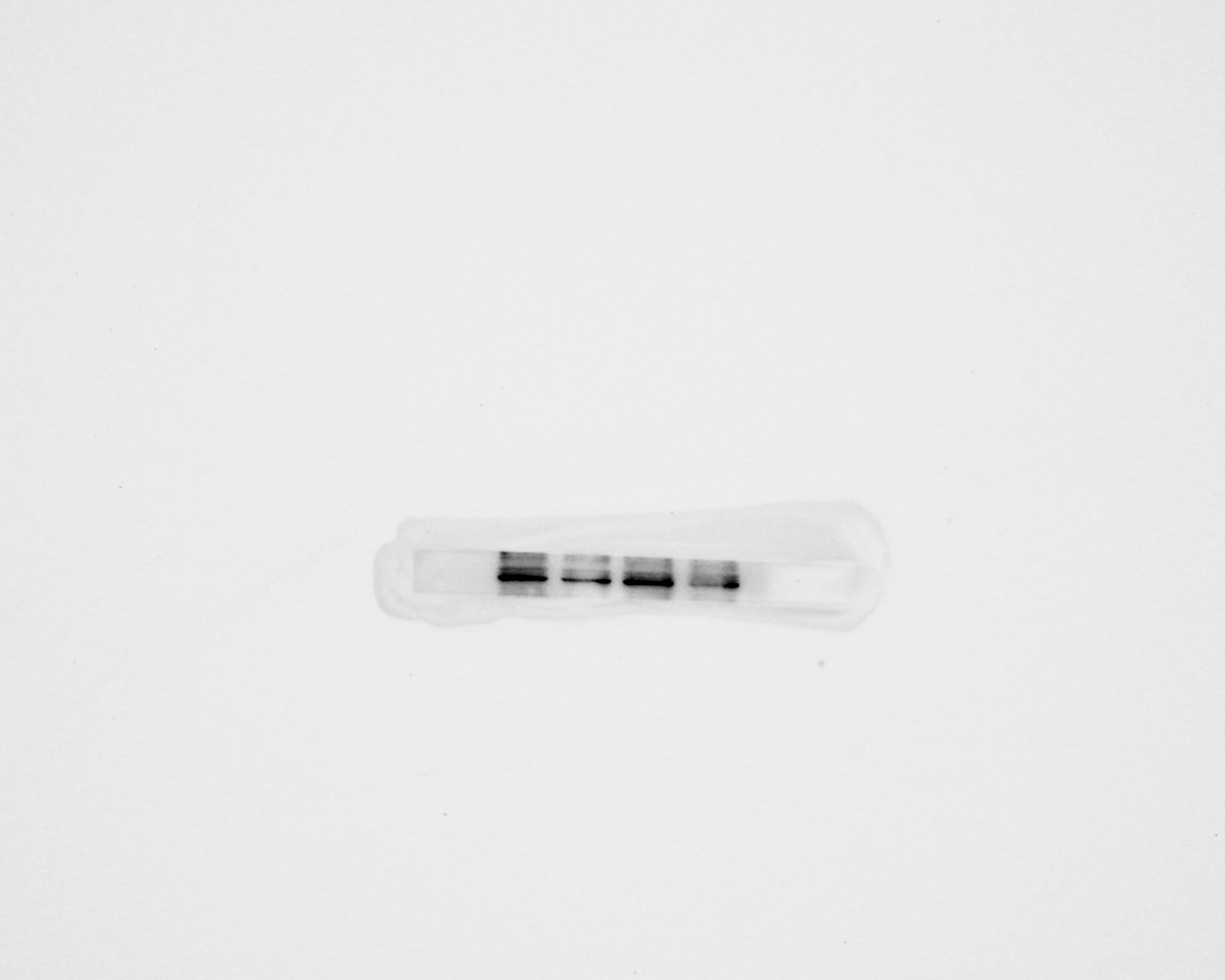

Supplement: S2 File — (ZIP) [file pone.0347758.s002.zip › FIG6B/nrf2/3-nrf-0112_3(Chemiluminescence).tif]

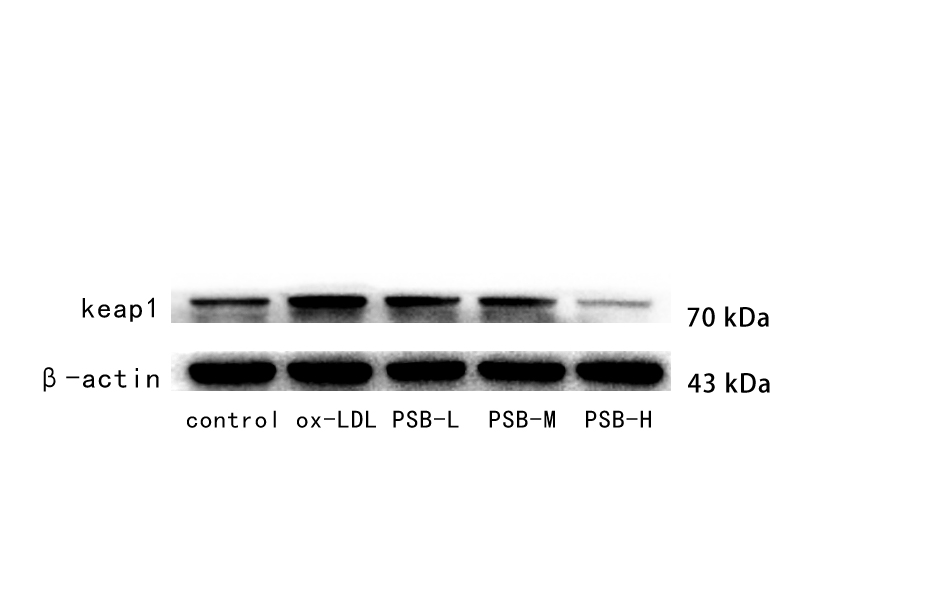

Supplement: S2 File — (ZIP) [file pone.0347758.s002.zip › FIG6D/keap1/0106-keap1-2 拷贝 2.tif]

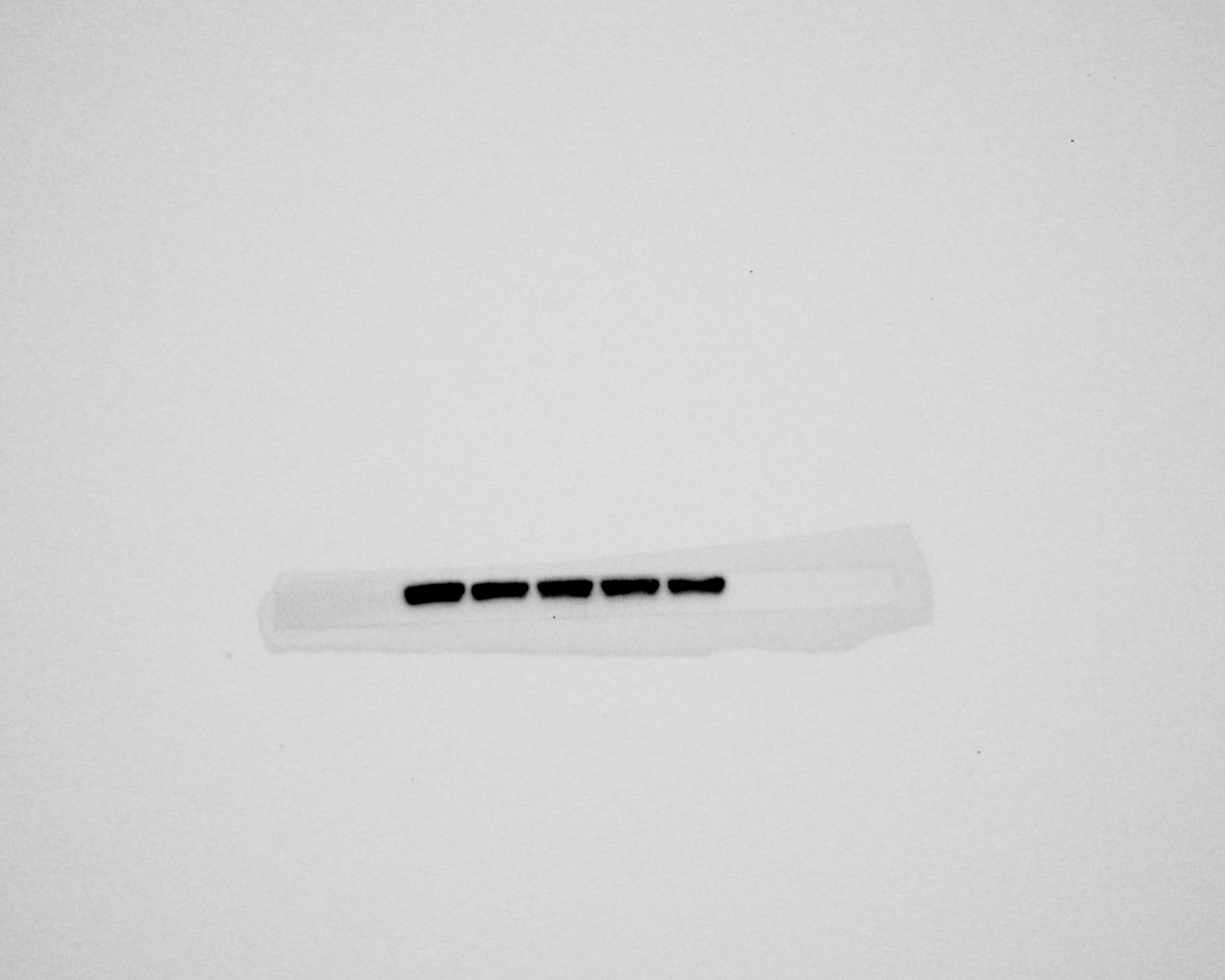

Supplement: S2 File — (ZIP) [file pone.0347758.s002.zip › FIG6D/keap1/1-actin-1227_2(Chemiluminescence).tif]

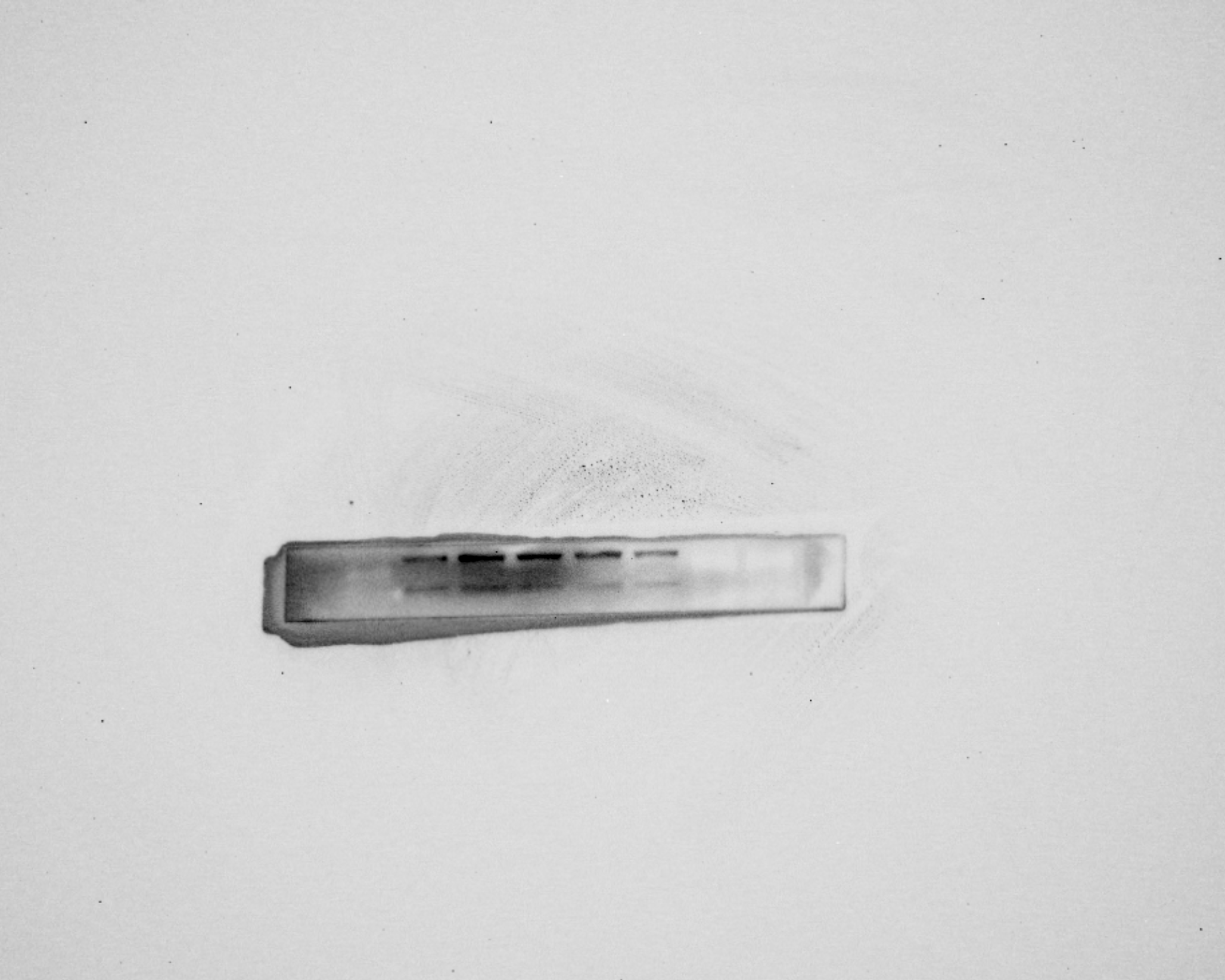

Supplement: S2 File — (ZIP) [file pone.0347758.s002.zip › FIG6D/keap1/1-keap-1227_1(Chemiluminescence).tif]

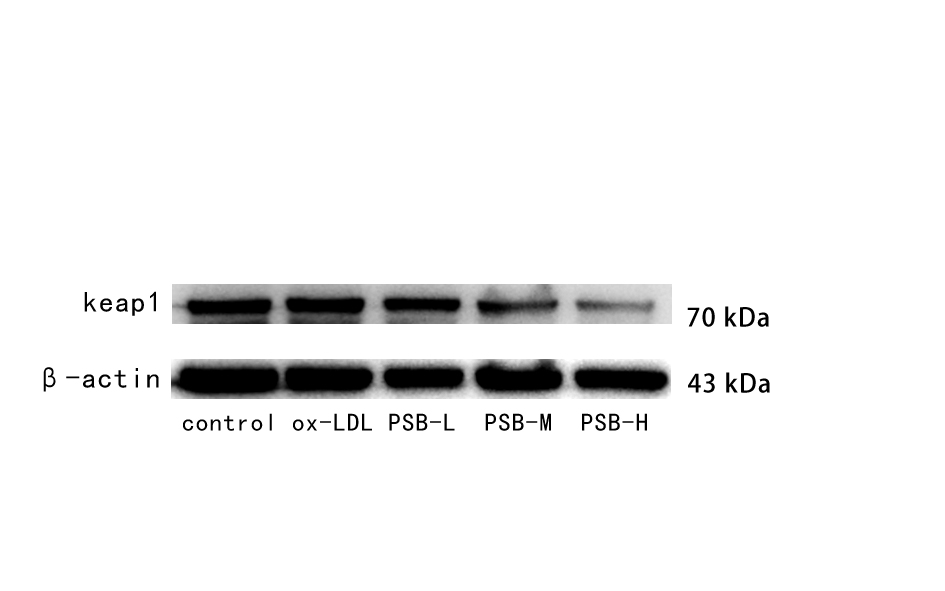

Supplement: S2 File — (ZIP) [file pone.0347758.s002.zip › FIG6D/keap1/1222-keap1-3 拷贝 2.tif]

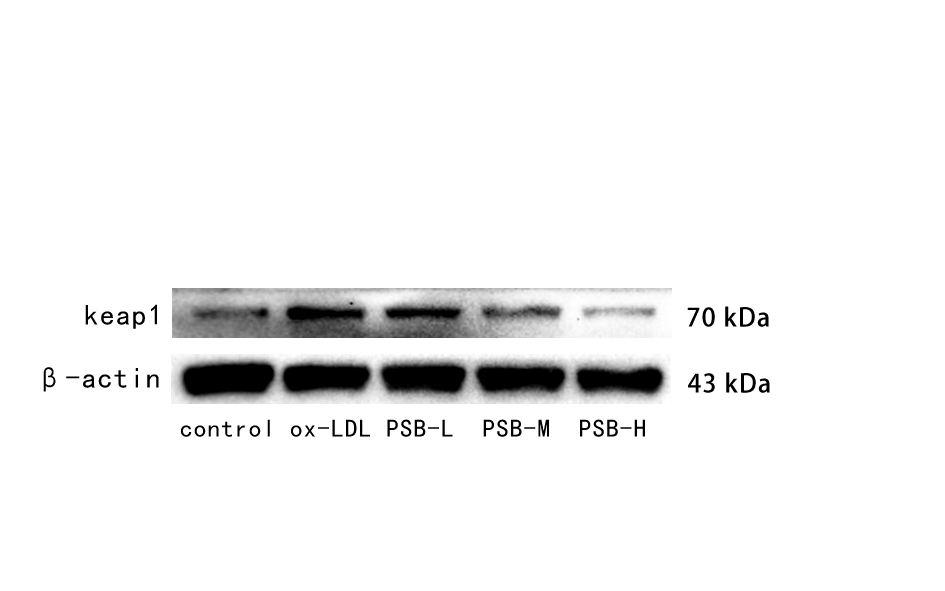

Supplement: S2 File — (ZIP) [file pone.0347758.s002.zip › FIG6D/keap1/1227-keap1-1拷贝 2.tif]

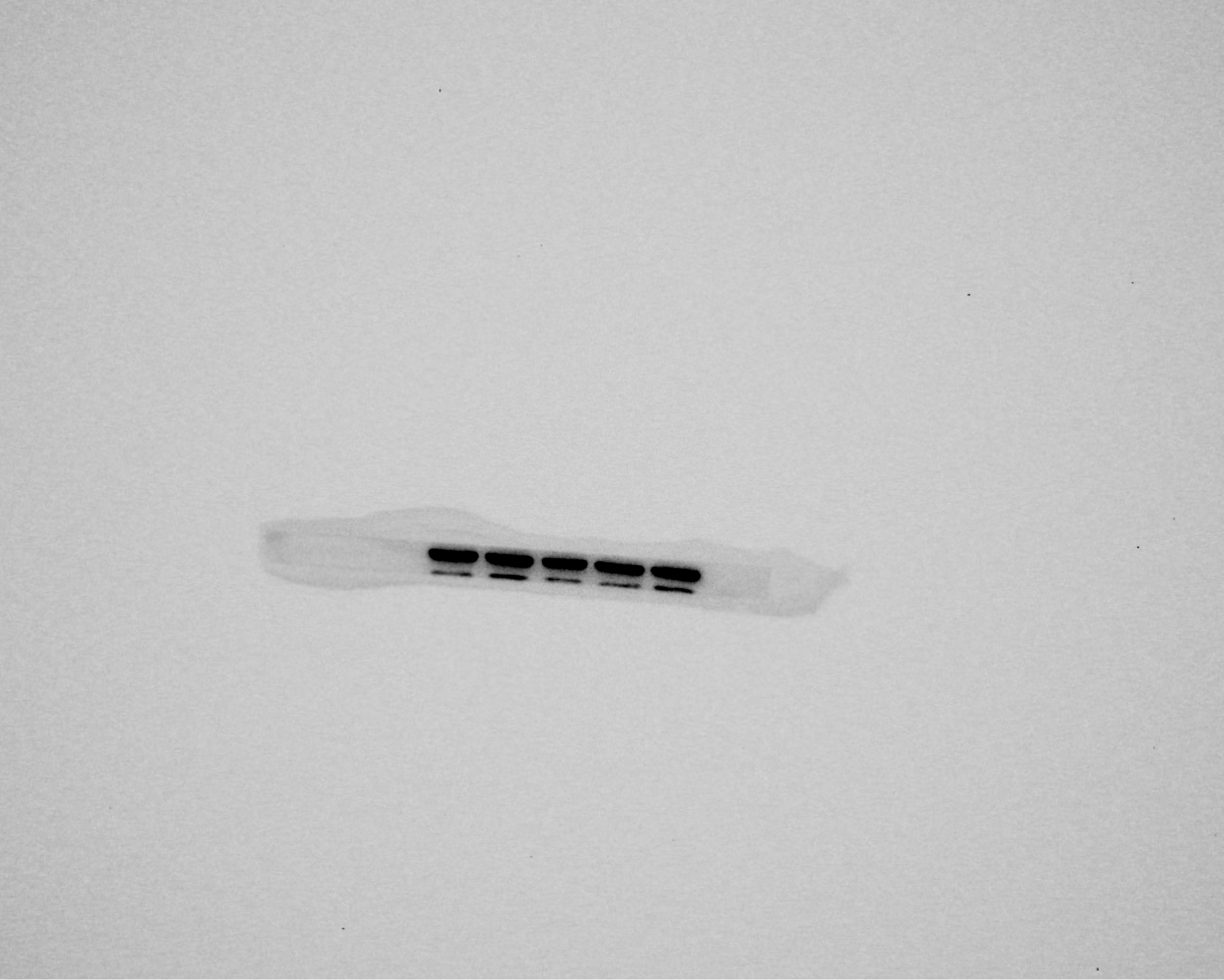

Supplement: S2 File — (ZIP) [file pone.0347758.s002.zip › FIG6D/keap1/2-actin-0106_2(Chemiluminescence).tif]

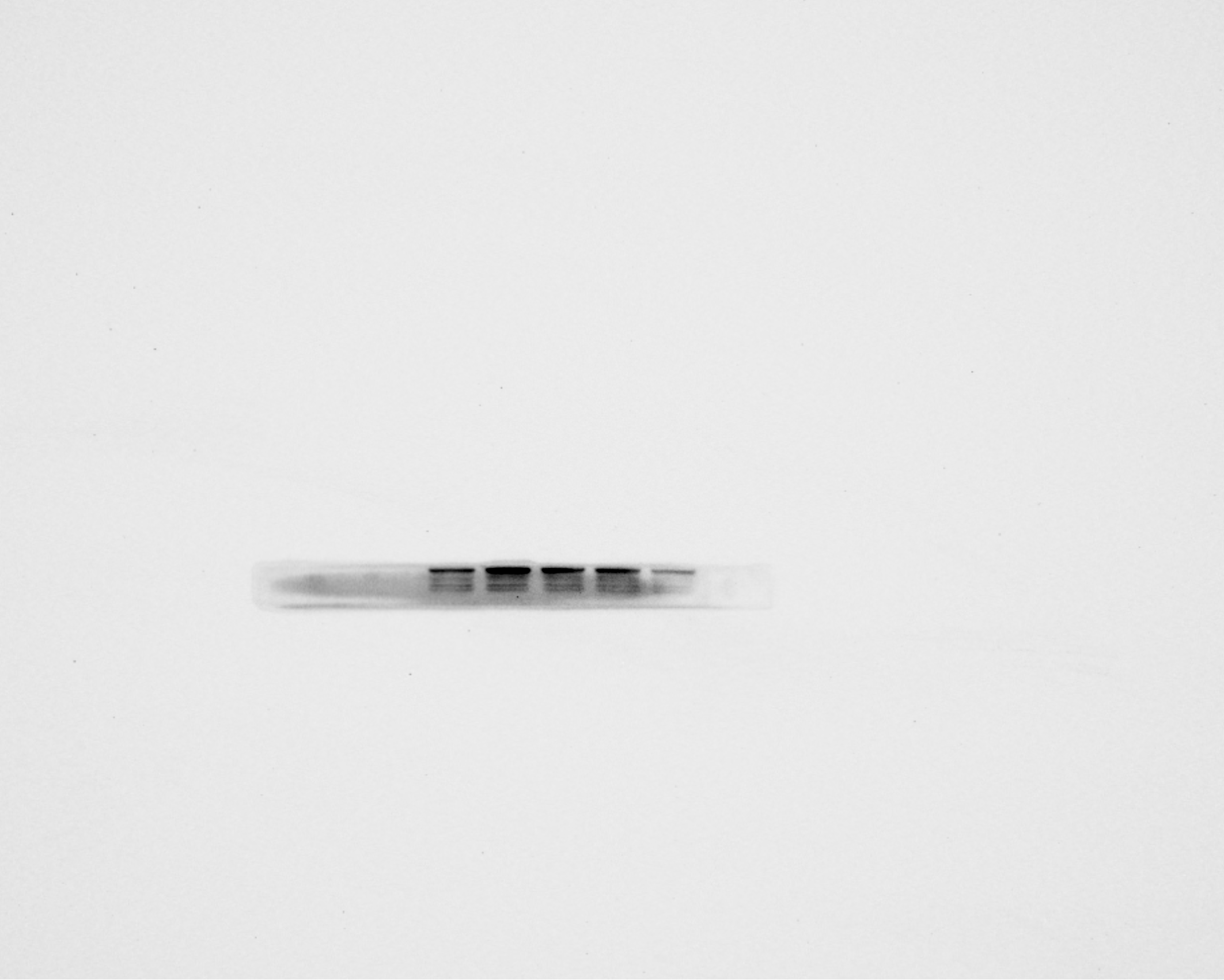

Supplement: S2 File — (ZIP) [file pone.0347758.s002.zip › FIG6D/keap1/2-keap-0106_5(Chemiluminescence).tif]

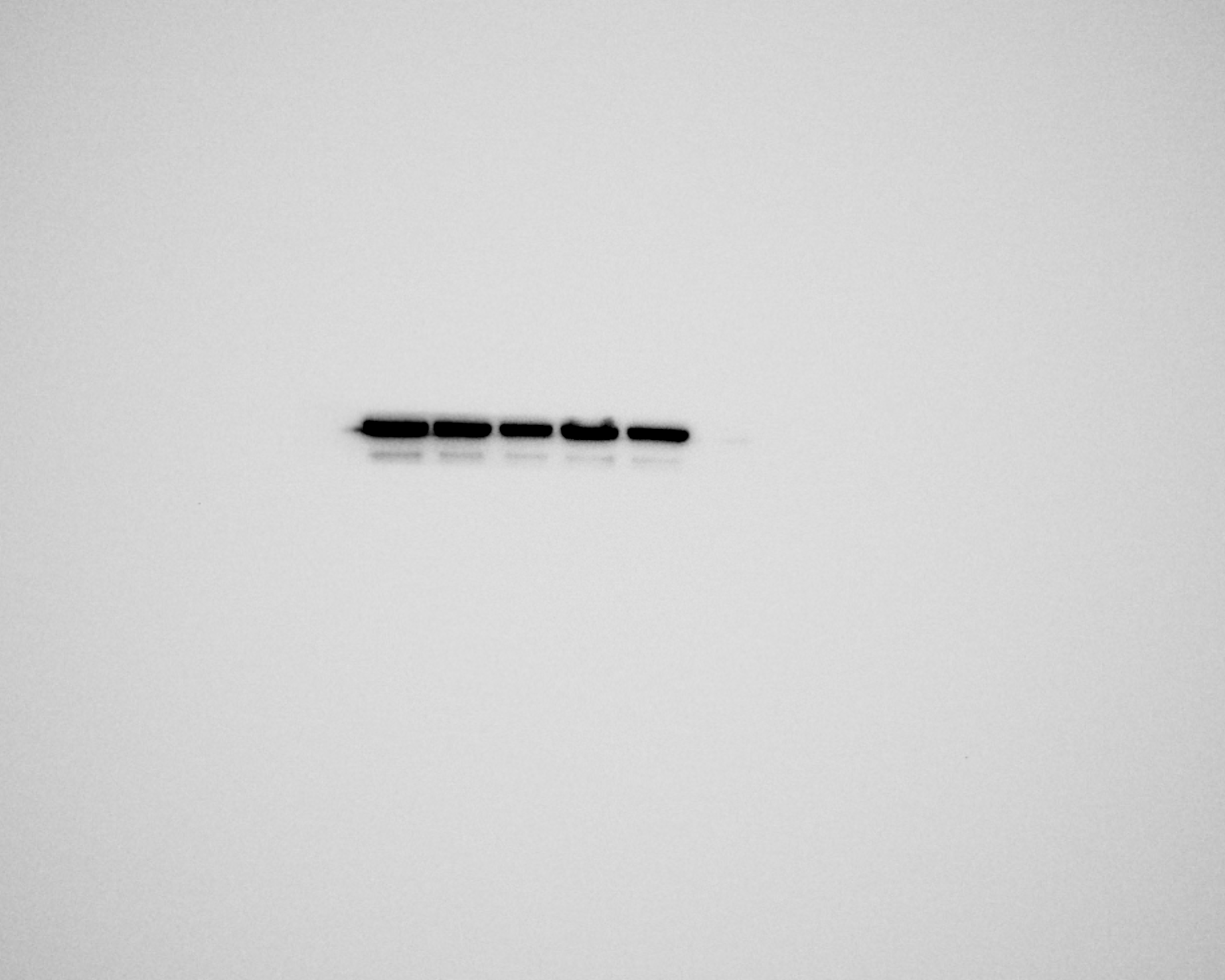

Supplement: S2 File — (ZIP) [file pone.0347758.s002.zip › FIG6D/keap1/3-actin-1222_1(Chemiluminescence).tif]

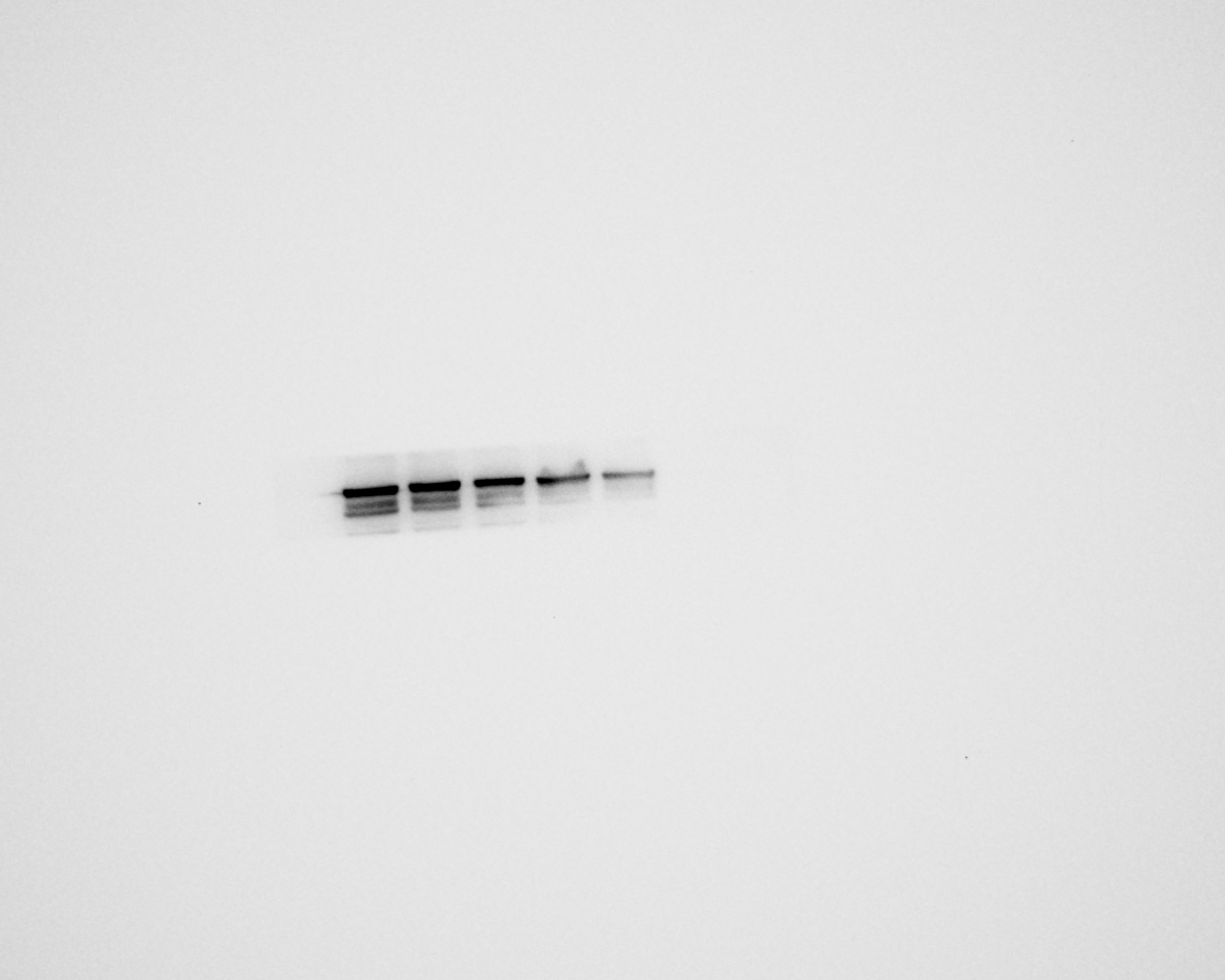

Supplement: S2 File — (ZIP) [file pone.0347758.s002.zip › FIG6D/keap1/3-keap-1222_5(Chemiluminescence).tif]

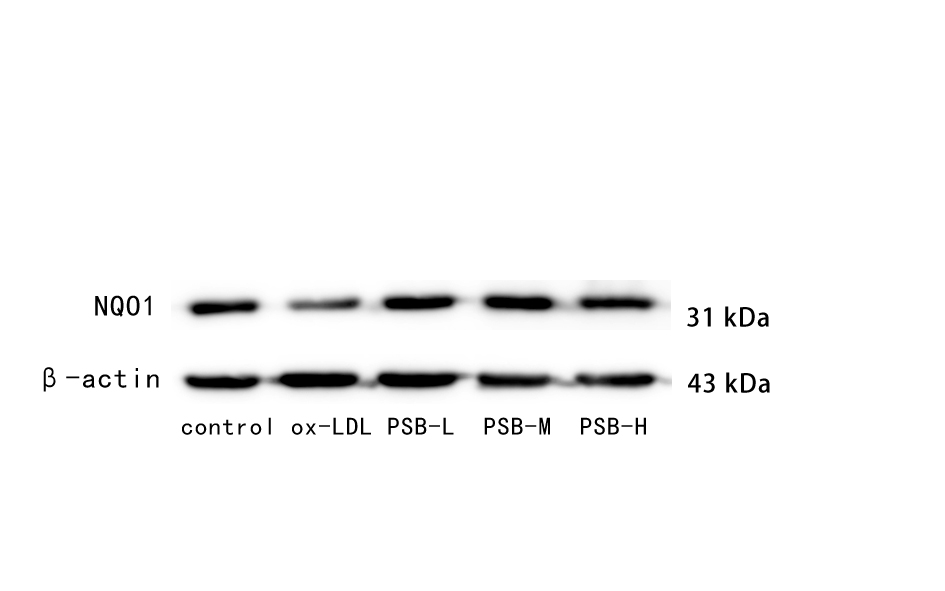

Supplement: S2 File — (ZIP) [file pone.0347758.s002.zip › FIG6D/NQO1/0124 拷贝 2.tif]

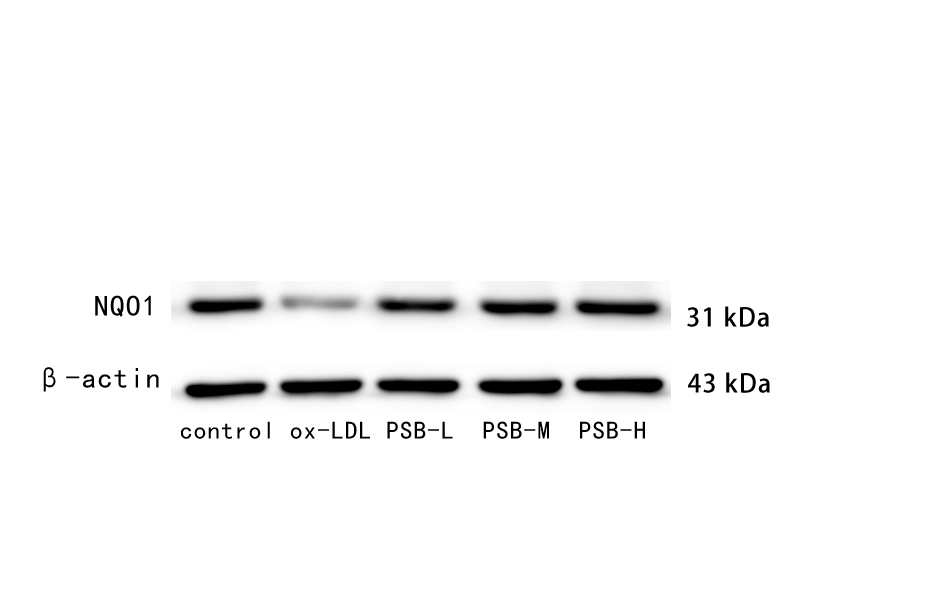

Supplement: S2 File — (ZIP) [file pone.0347758.s002.zip › FIG6D/NQO1/0129 拷贝 2.tif]

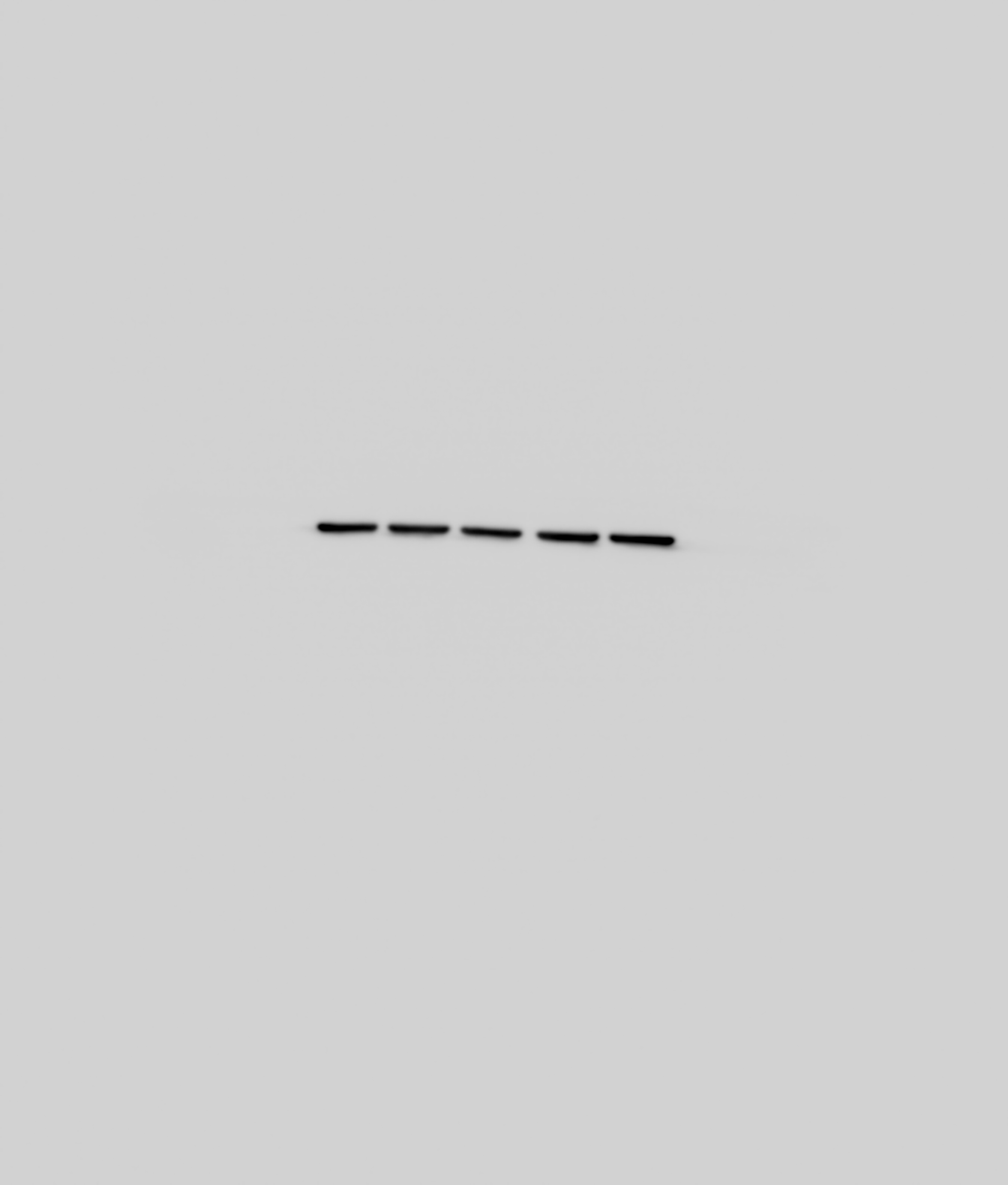

Supplement: S2 File — (ZIP) [file pone.0347758.s002.zip › FIG6D/NQO1/0129-1-actin-sample.tif]

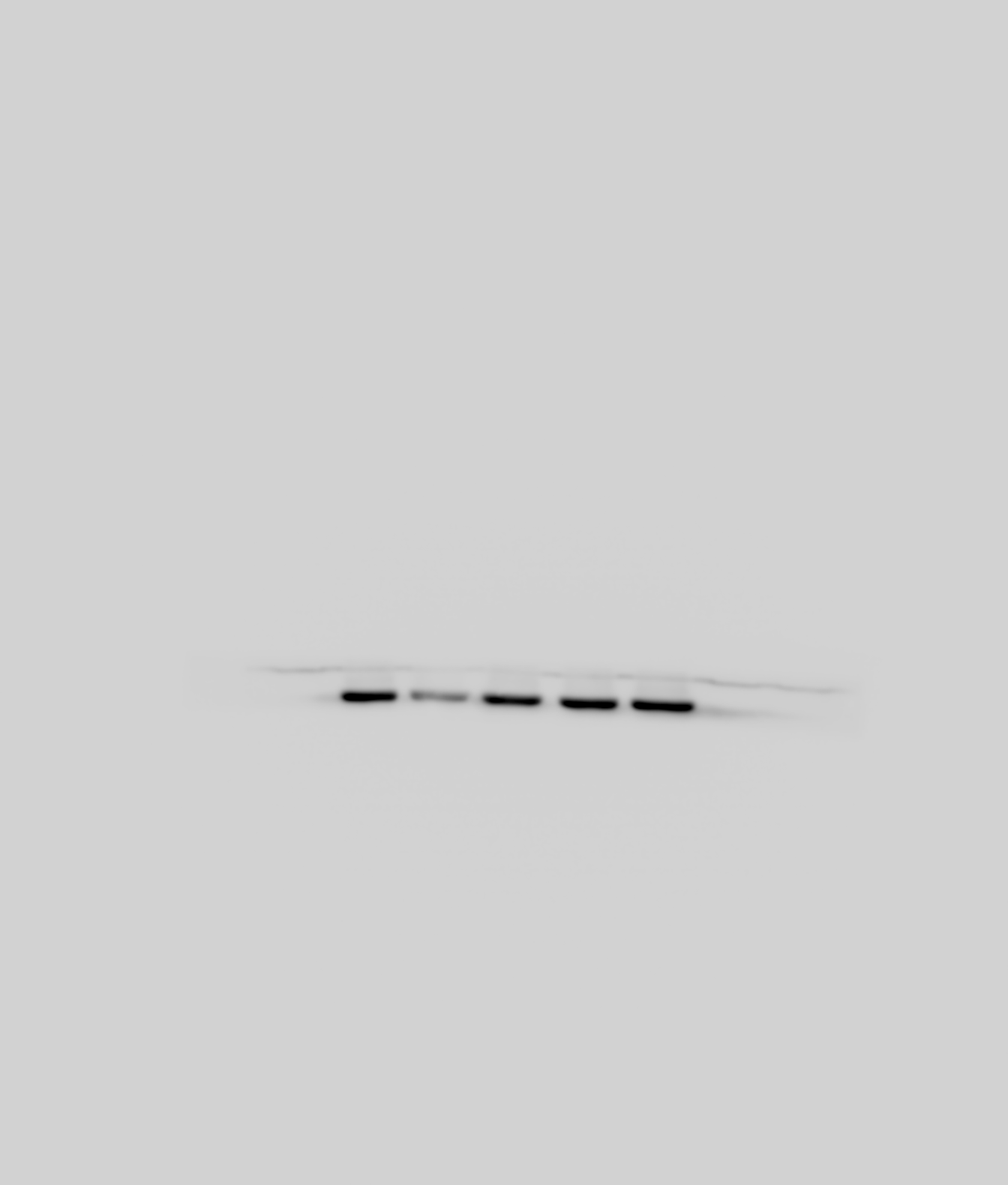

Supplement: S2 File — (ZIP) [file pone.0347758.s002.zip › FIG6D/NQO1/0129-1nqo-sample.tif]

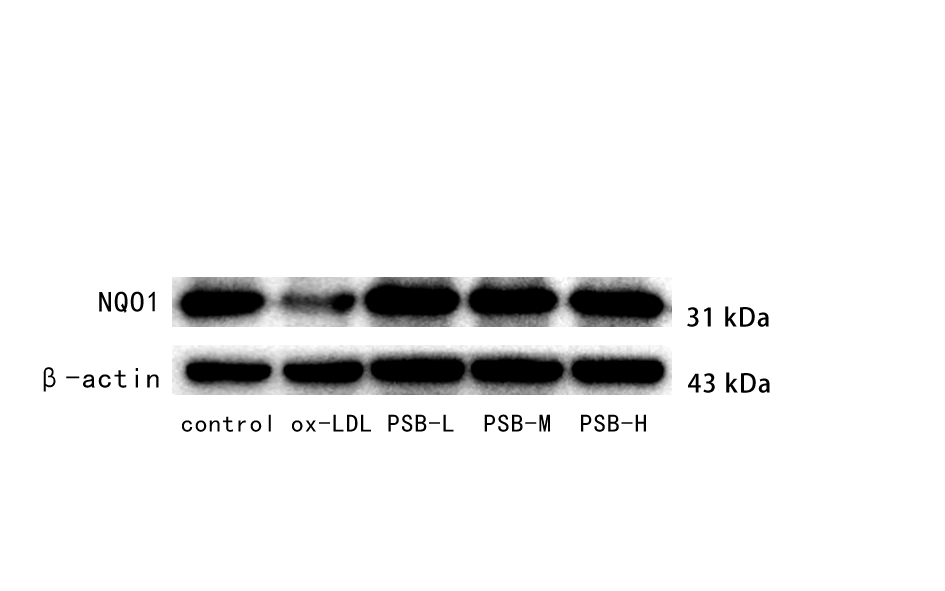

Supplement: S2 File — (ZIP) [file pone.0347758.s002.zip › FIG6D/NQO1/1222-NQO1-2 拷贝 2.tif]

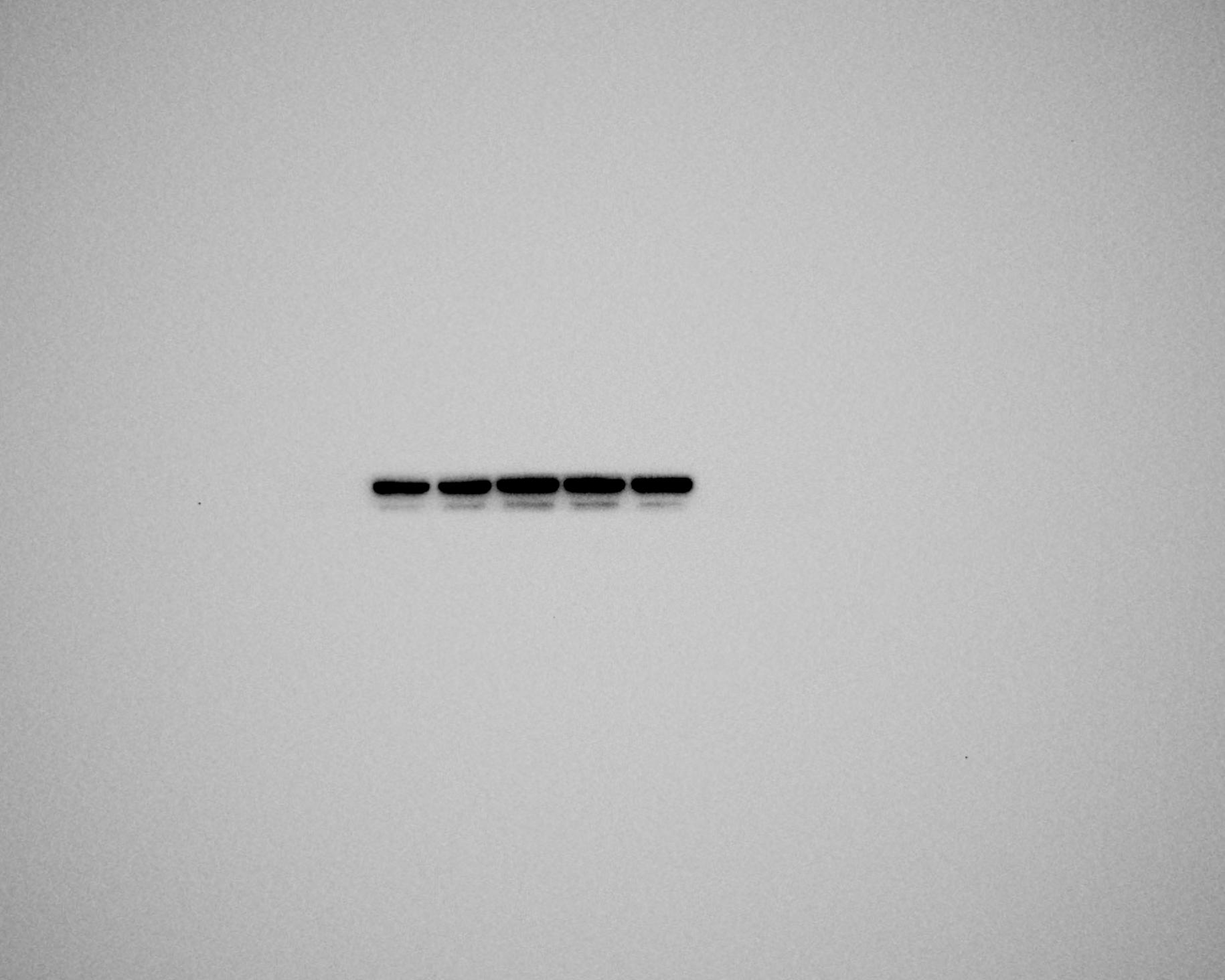

Supplement: S2 File — (ZIP) [file pone.0347758.s002.zip › FIG6D/NQO1/2-actin-1222_1(Chemiluminescence).tif]

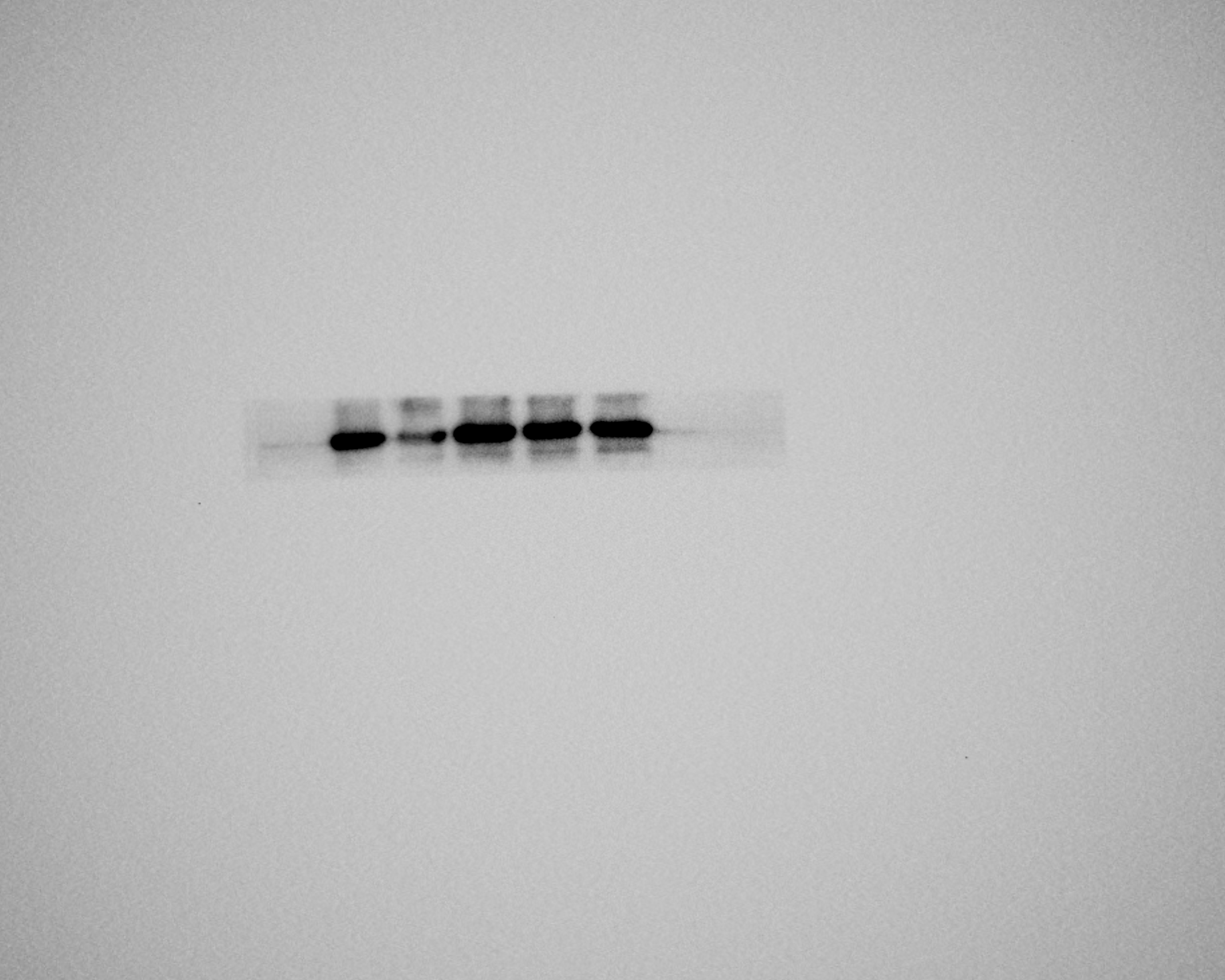

Supplement: S2 File — (ZIP) [file pone.0347758.s002.zip › FIG6D/NQO1/2-nqo-1222_2(Chemiluminescence).tif]

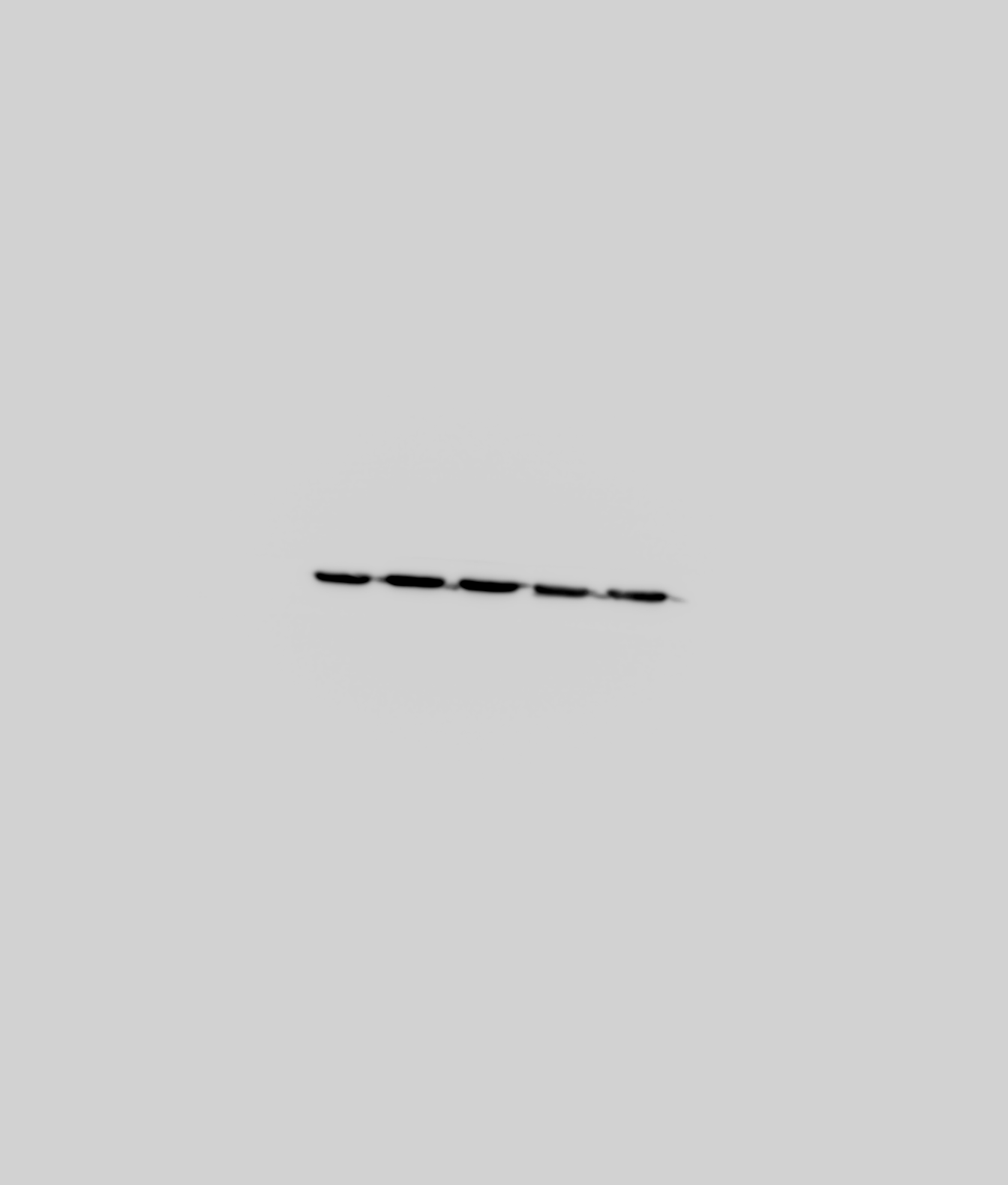

Supplement: S2 File — (ZIP) [file pone.0347758.s002.zip › FIG6D/NQO1/3-ACTIN-0124-sample.tif]

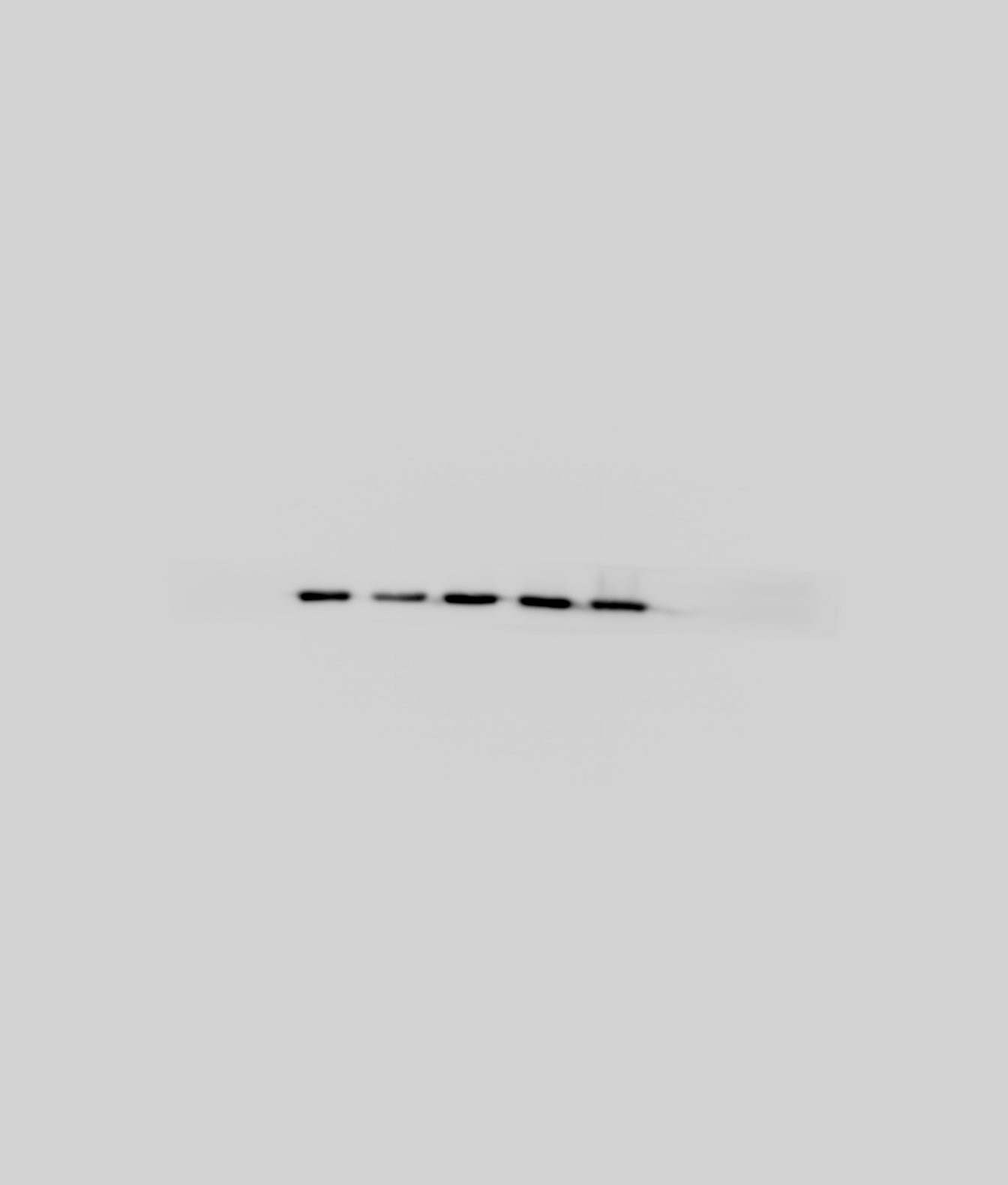

Supplement: S2 File — (ZIP) [file pone.0347758.s002.zip › FIG6D/NQO1/3-NQO-0124-2-sample.tif]

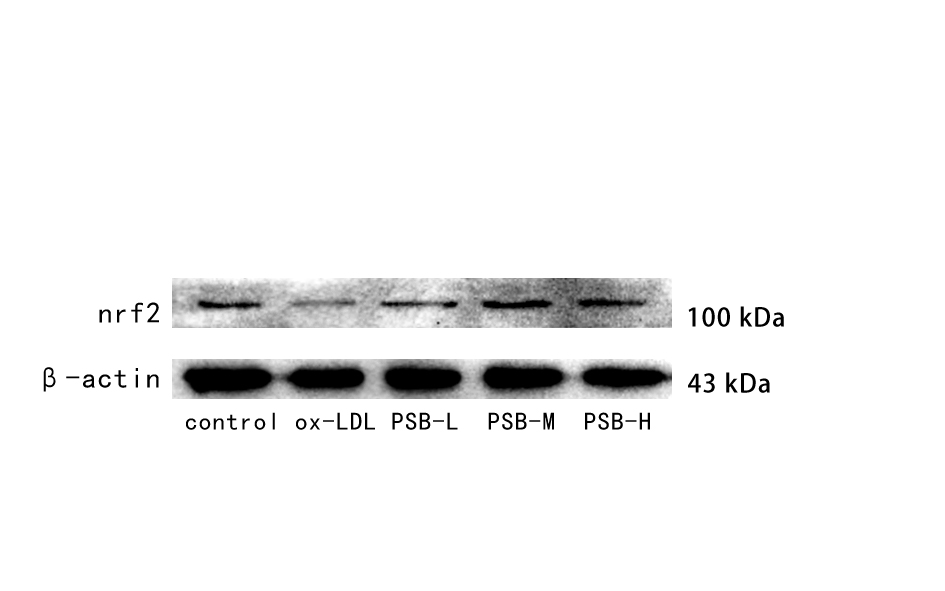

Supplement: S2 File — (ZIP) [file pone.0347758.s002.zip › FIG6D/nrf2/0108-nrf2-3 拷贝 2.tif]

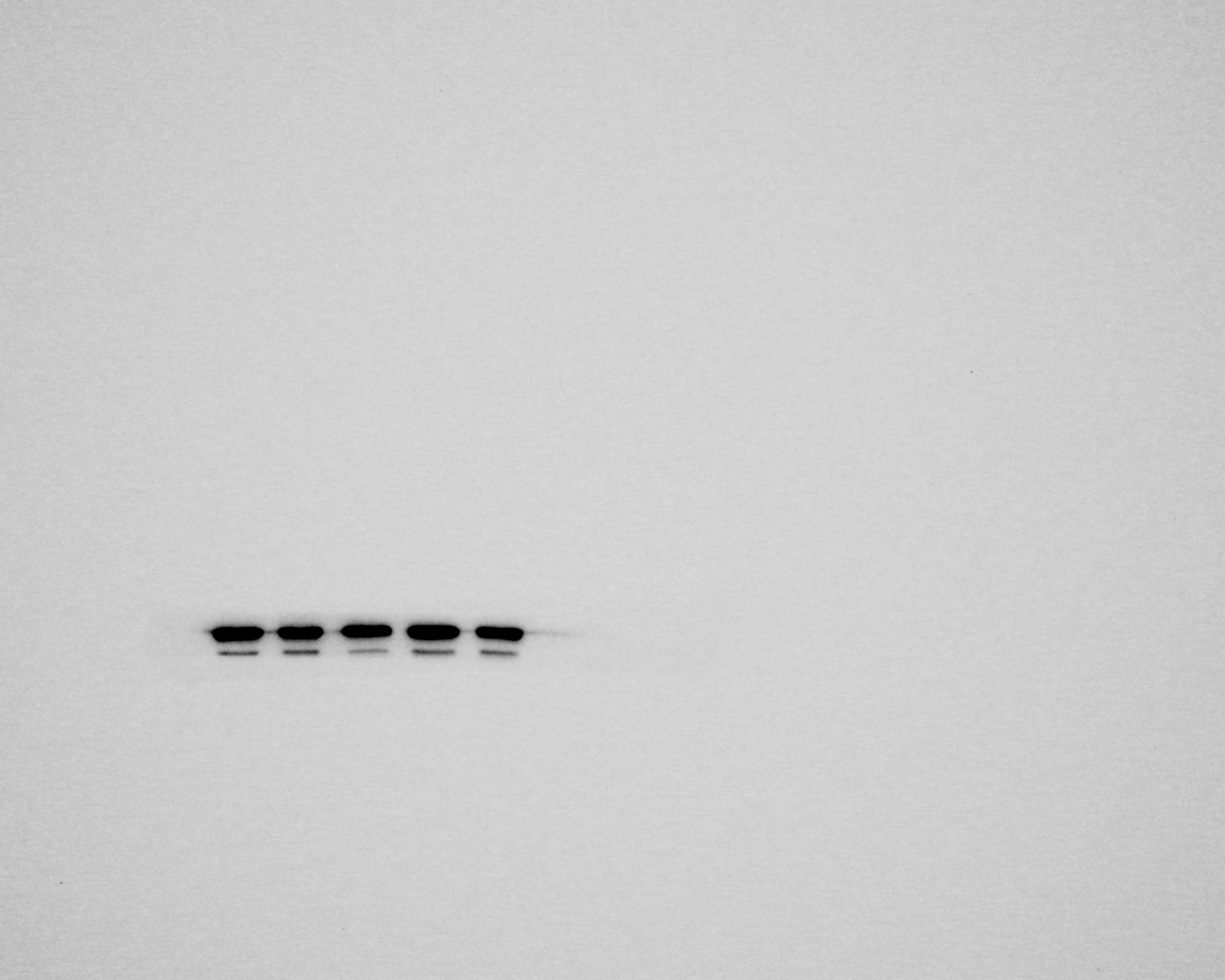

Supplement: S2 File — (ZIP) [file pone.0347758.s002.zip › FIG6D/nrf2/1-actih-1231_2(Chemiluminescence).tif]

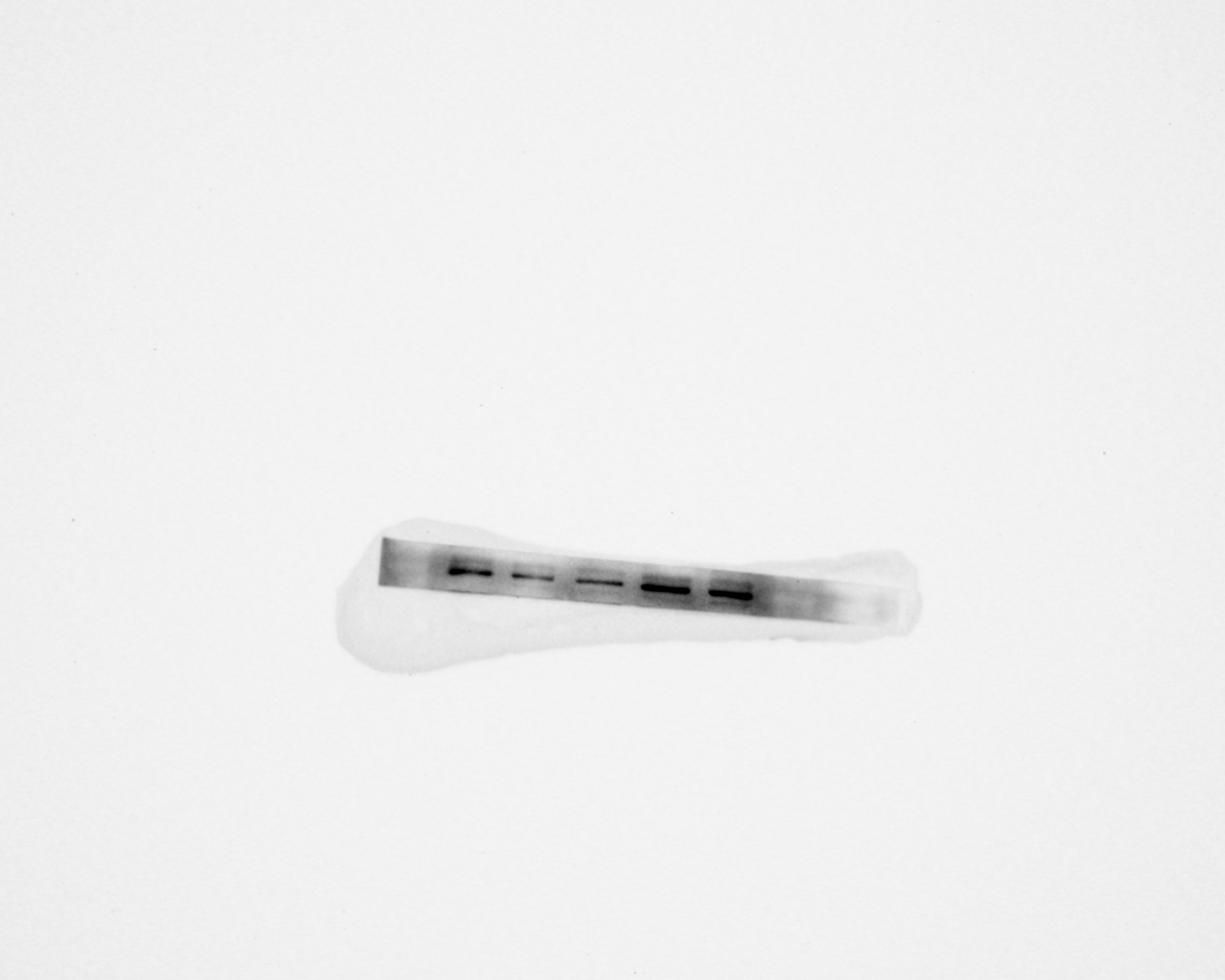

Supplement: S2 File — (ZIP) [file pone.0347758.s002.zip › FIG6D/nrf2/1-nrf2-1231_2(Chemiluminescence).tif]

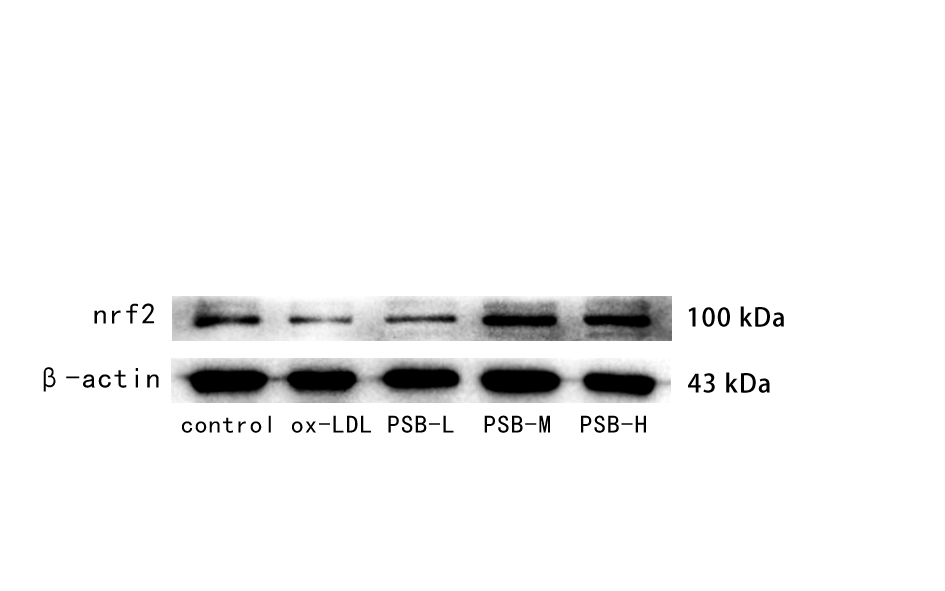

Supplement: S2 File — (ZIP) [file pone.0347758.s002.zip › FIG6D/nrf2/1231-nrf2-1 拷贝 2.tif]

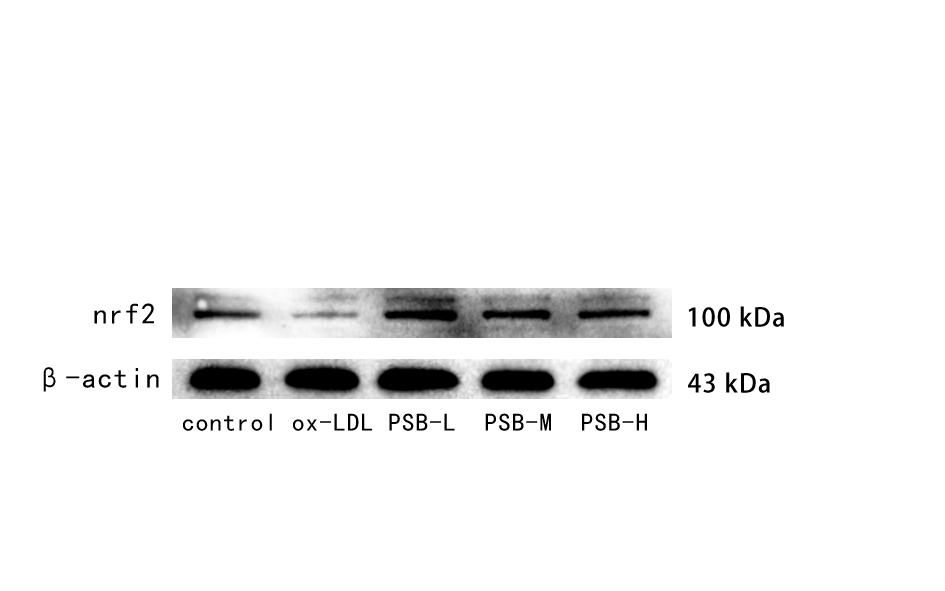

Supplement: S2 File — (ZIP) [file pone.0347758.s002.zip › FIG6D/nrf2/1231-nrf2-2 拷贝 2.tif]

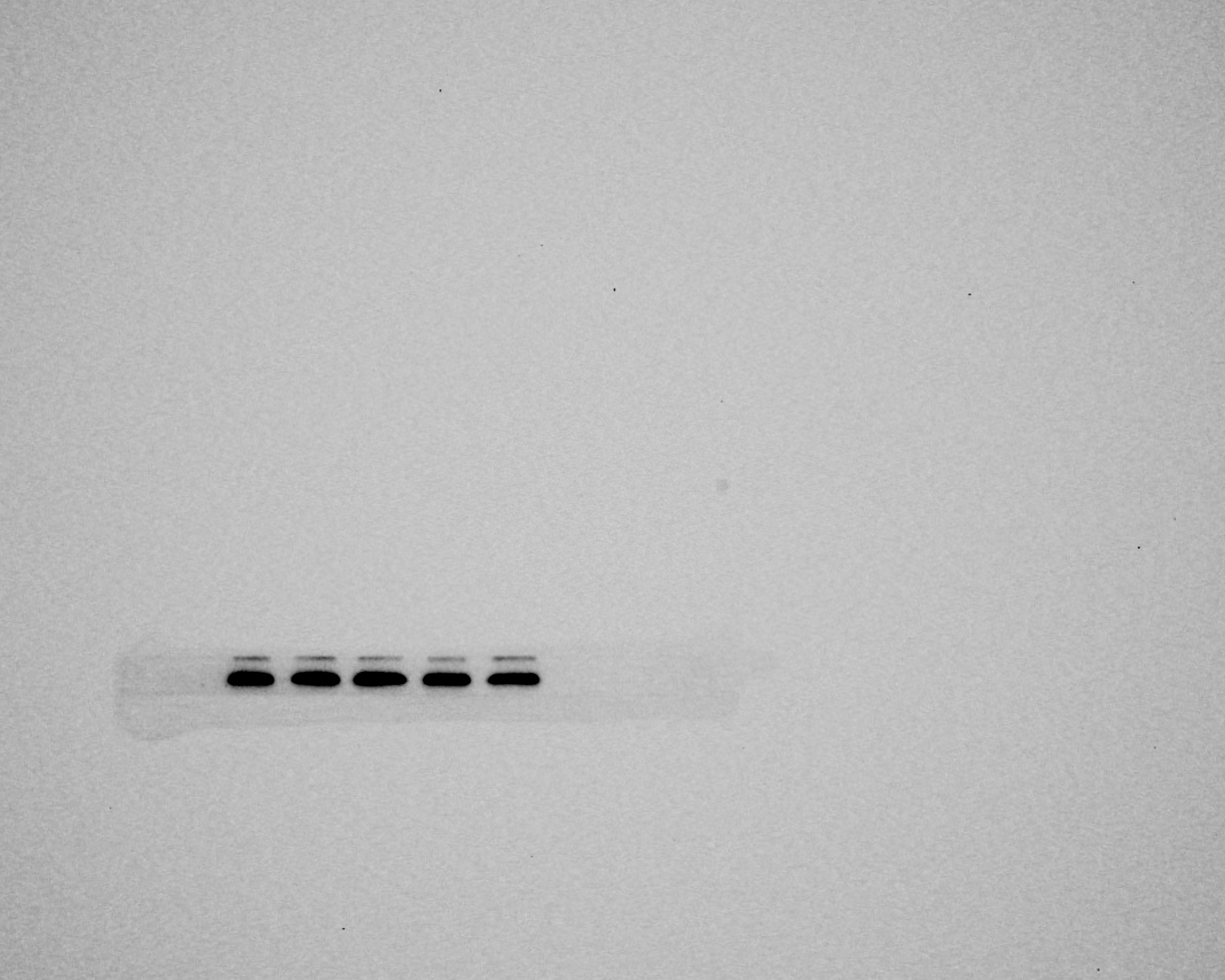

Supplement: S2 File — (ZIP) [file pone.0347758.s002.zip › FIG6D/nrf2/2-actin-1231_2(Chemiluminescence).tif]

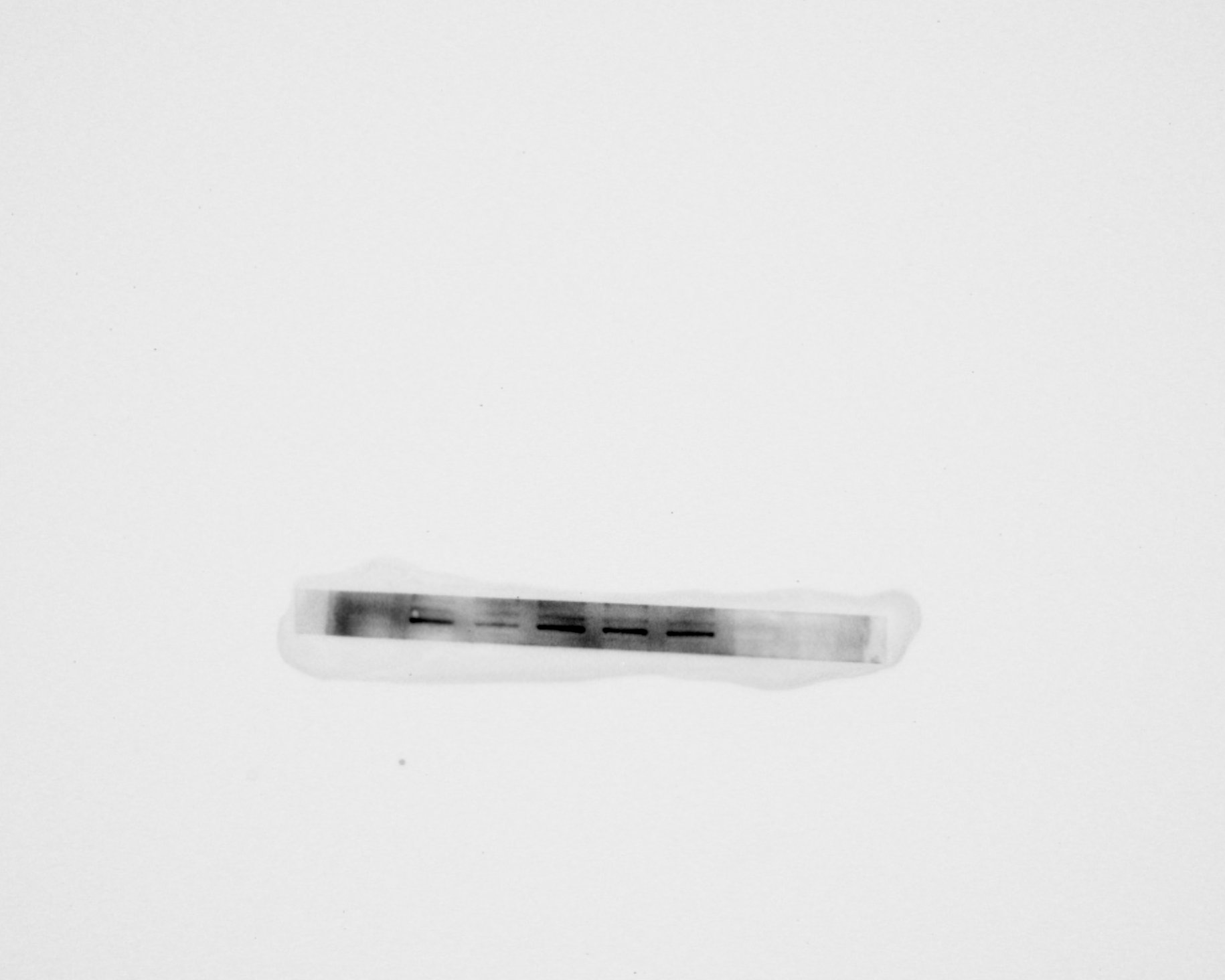

Supplement: S2 File — (ZIP) [file pone.0347758.s002.zip › FIG6D/nrf2/2-nrf-1231_3(Chemiluminescence).tif]

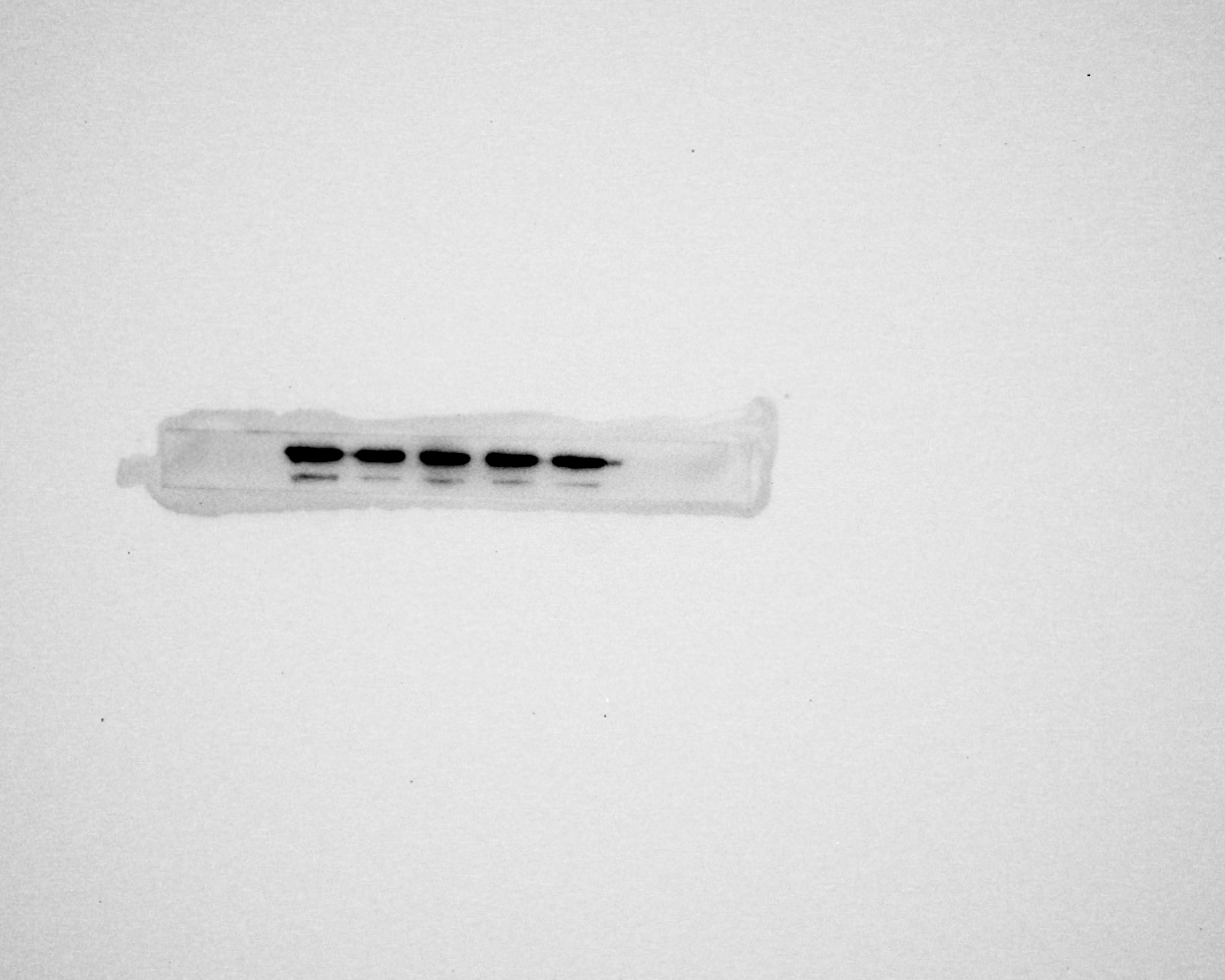

Supplement: S2 File — (ZIP) [file pone.0347758.s002.zip › FIG6D/nrf2/3-actin-0108_2(Chemiluminescence).tif]

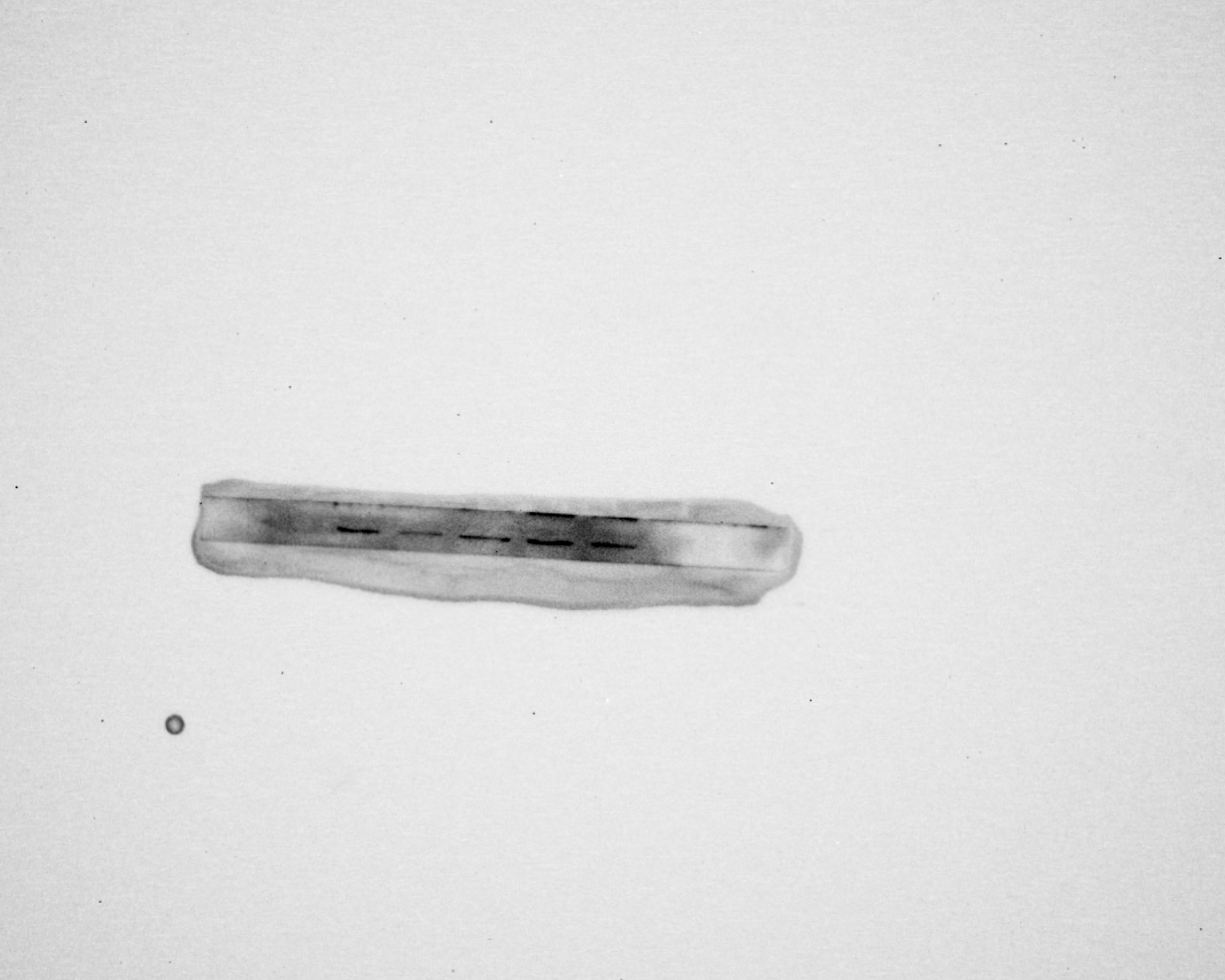

Supplement: S2 File — (ZIP) [file pone.0347758.s002.zip › FIG6D/nrf2/3-nrf-0108_3(Chemiluminescence).tif]

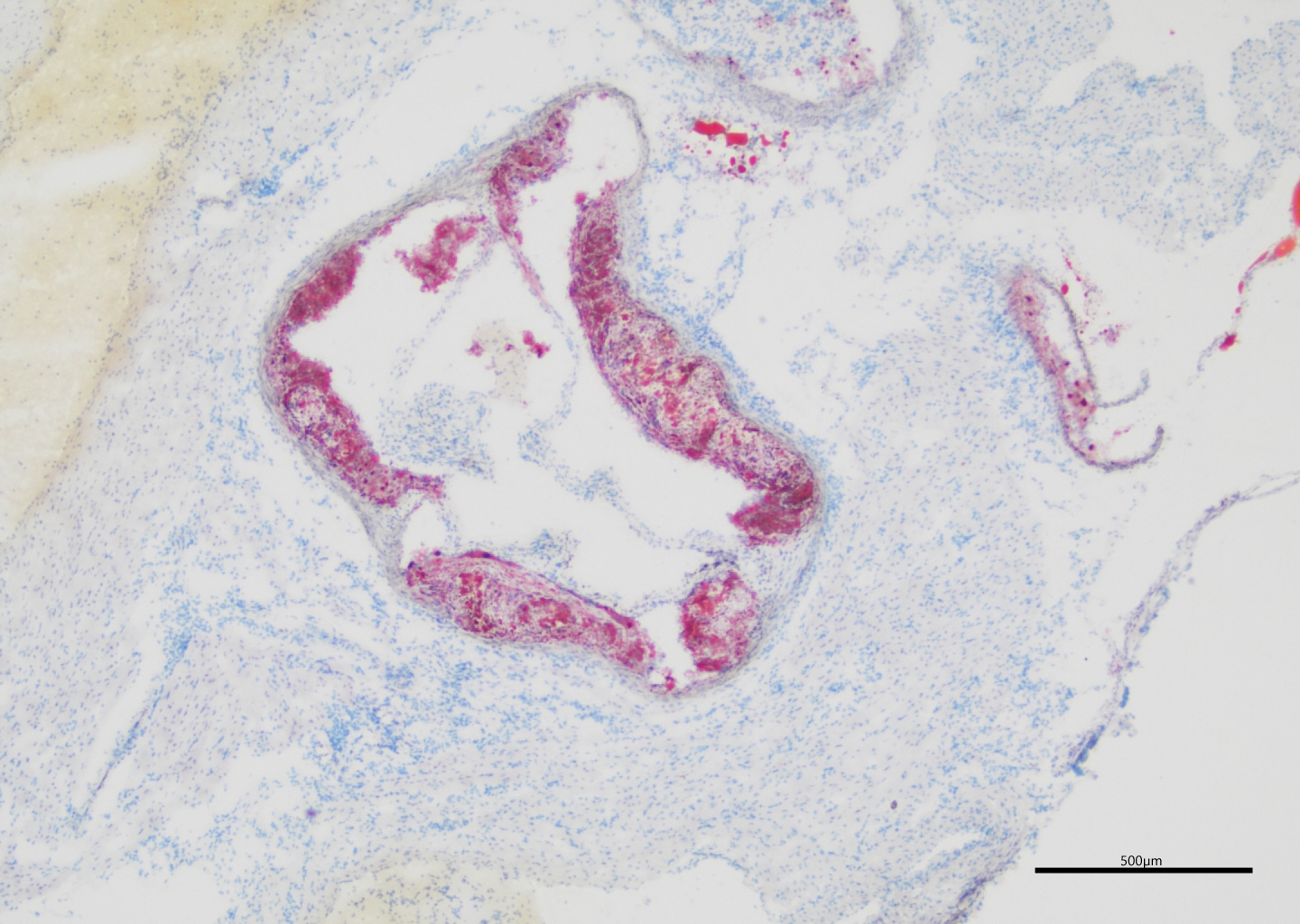

Supplement: S3 File — (ZIP) [file pone.0347758.s003.zip › Oil red O staining of aortic Root/AS/23 40-1.jpg]

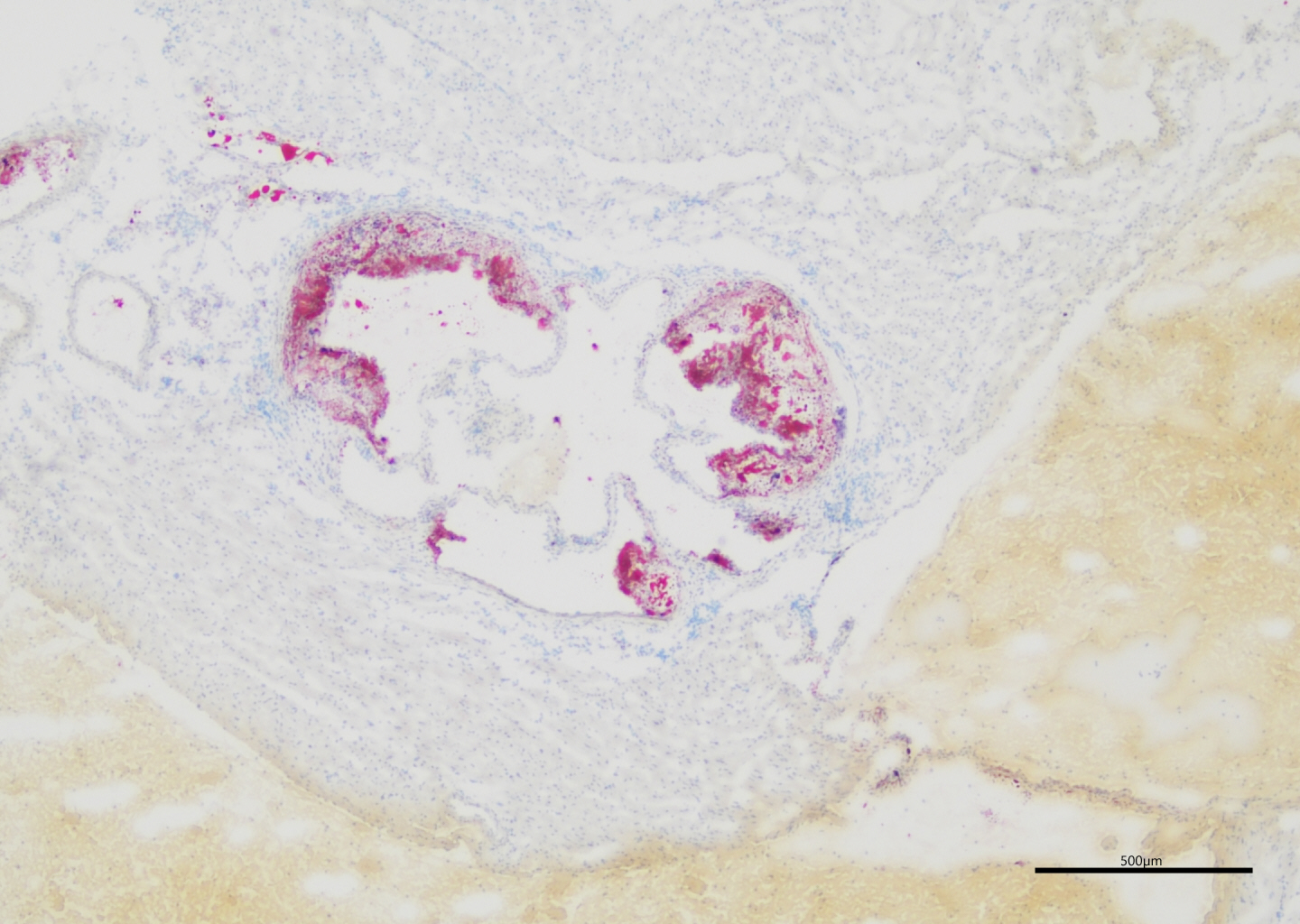

Supplement: S3 File — (ZIP) [file pone.0347758.s003.zip › Oil red O staining of aortic Root/AS/27 40-1.jpg]

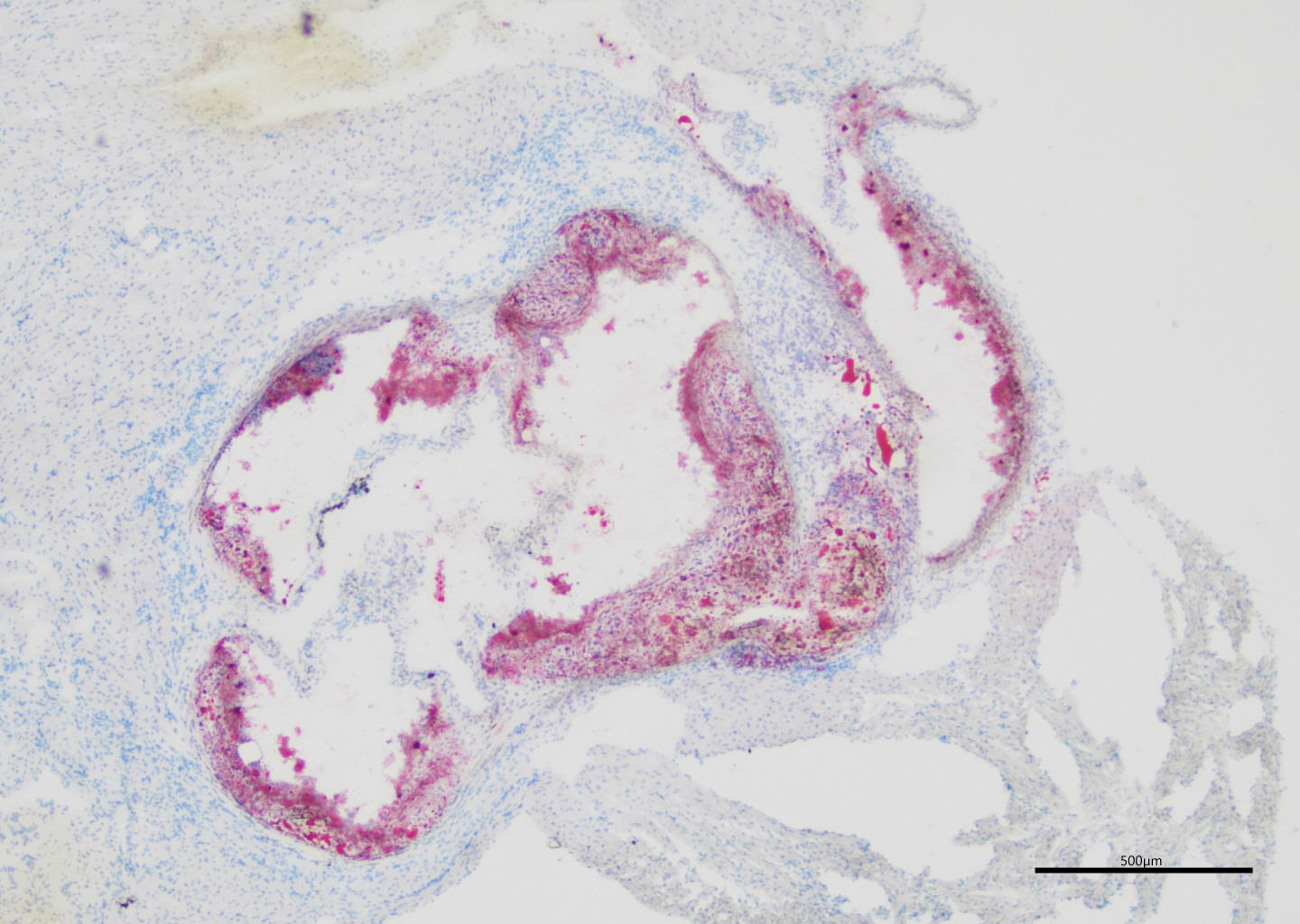

Supplement: S3 File — (ZIP) [file pone.0347758.s003.zip › Oil red O staining of aortic Root/AS/28 40-1.jpg]

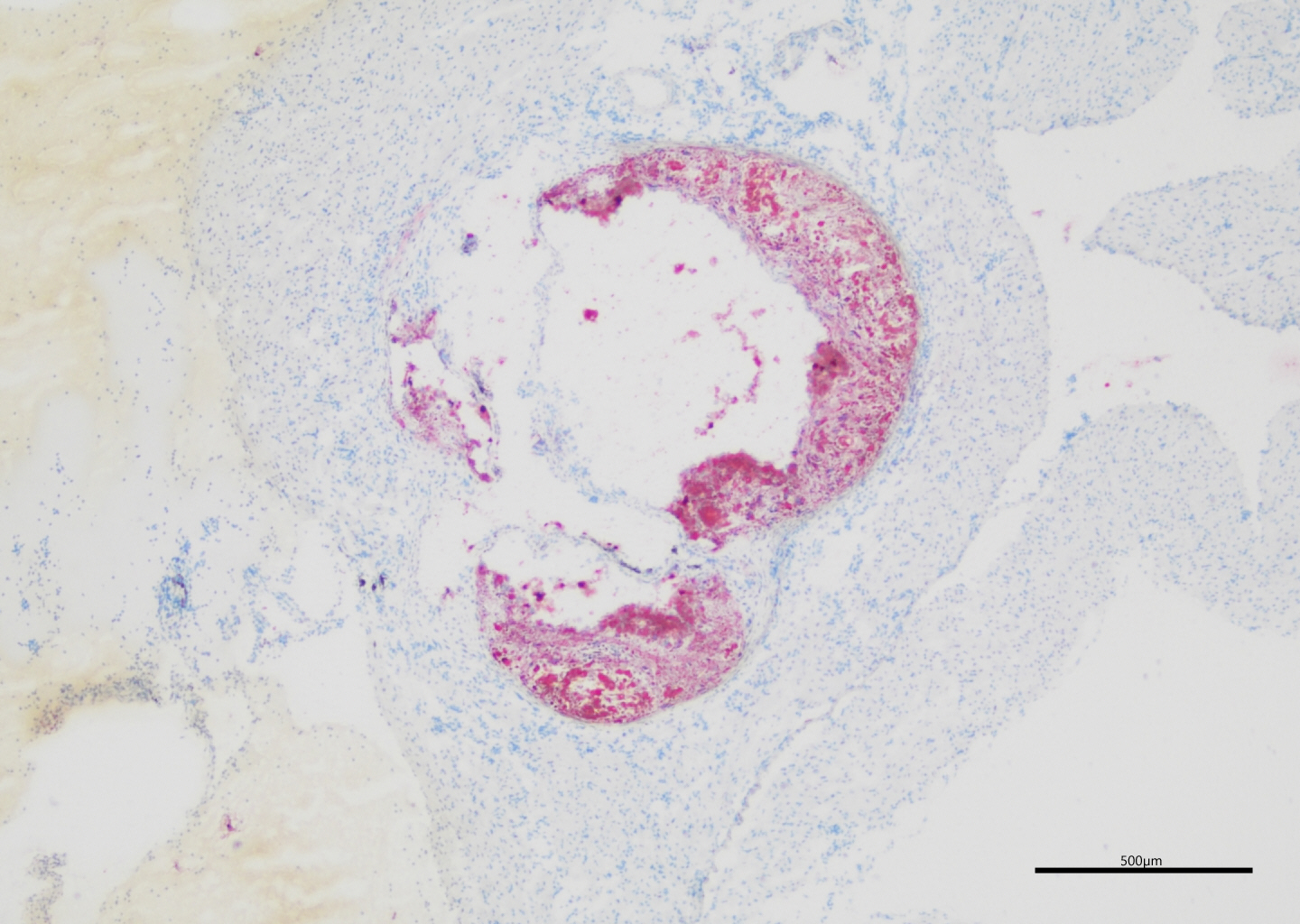

Supplement: S3 File — (ZIP) [file pone.0347758.s003.zip › Oil red O staining of aortic Root/AS/31 40-1.jpg]

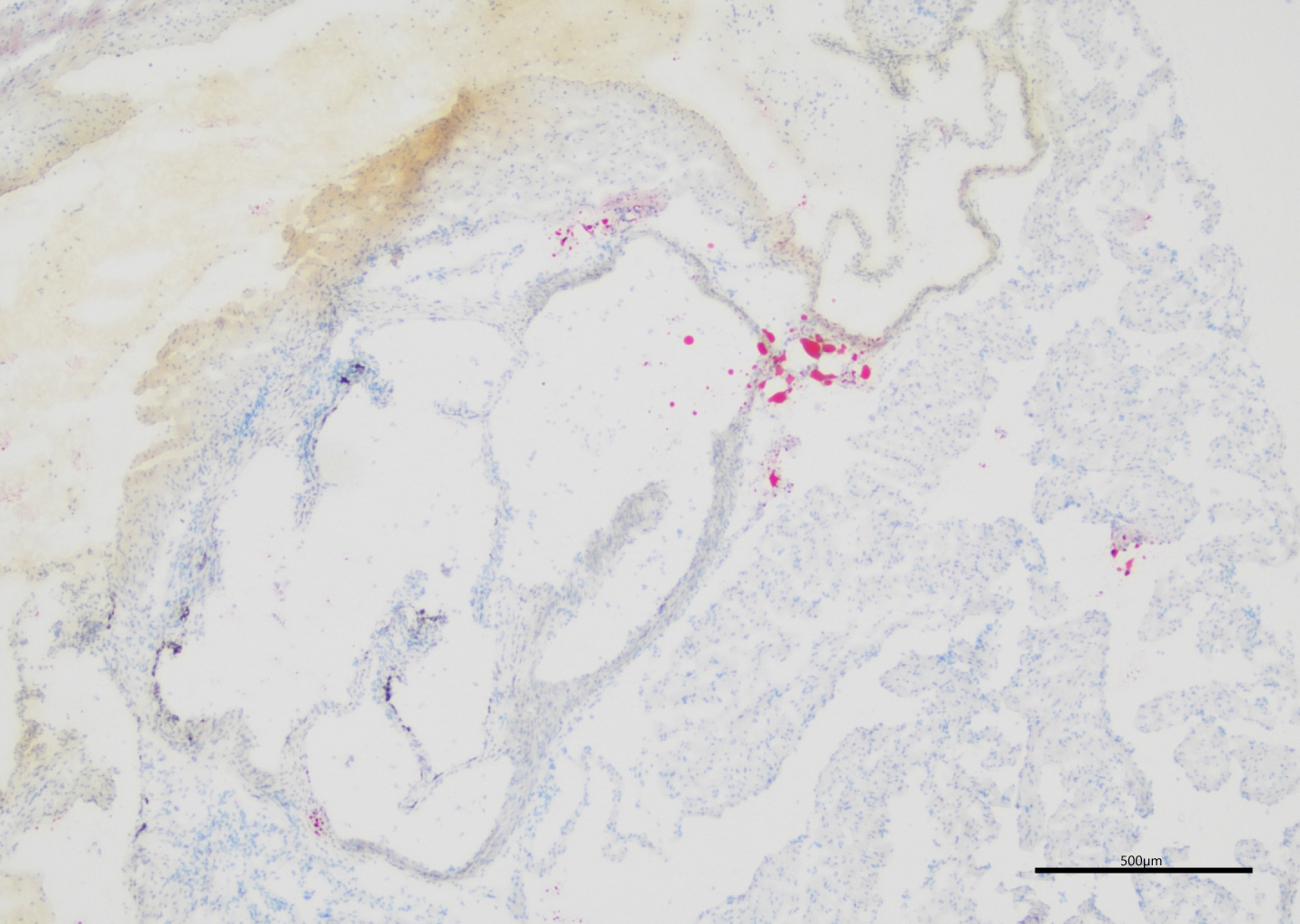

Supplement: S3 File — (ZIP) [file pone.0347758.s003.zip › Oil red O staining of aortic Root/control/1 40-1.jpg]

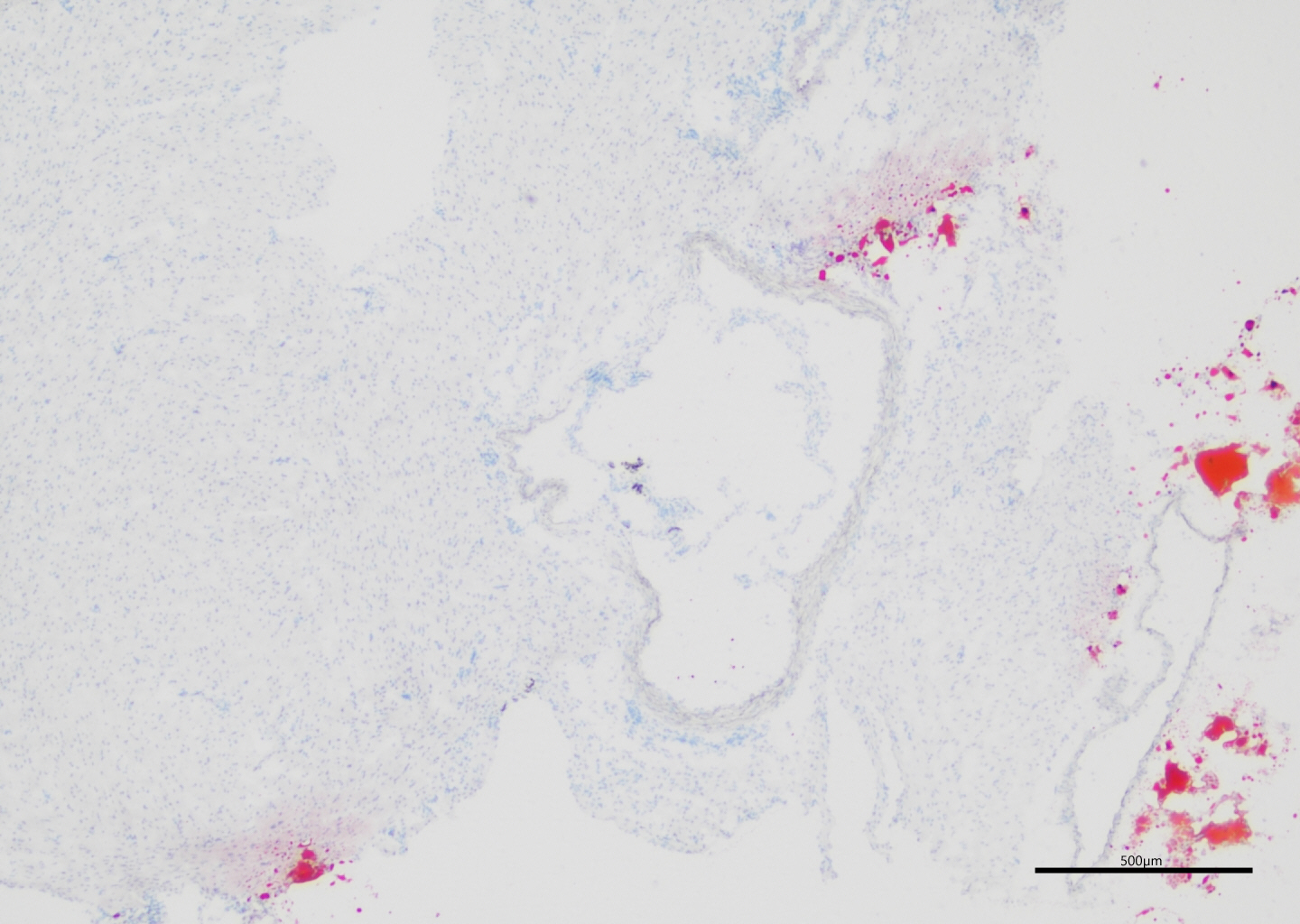

Supplement: S3 File — (ZIP) [file pone.0347758.s003.zip › Oil red O staining of aortic Root/control/2 40-1.jpg]

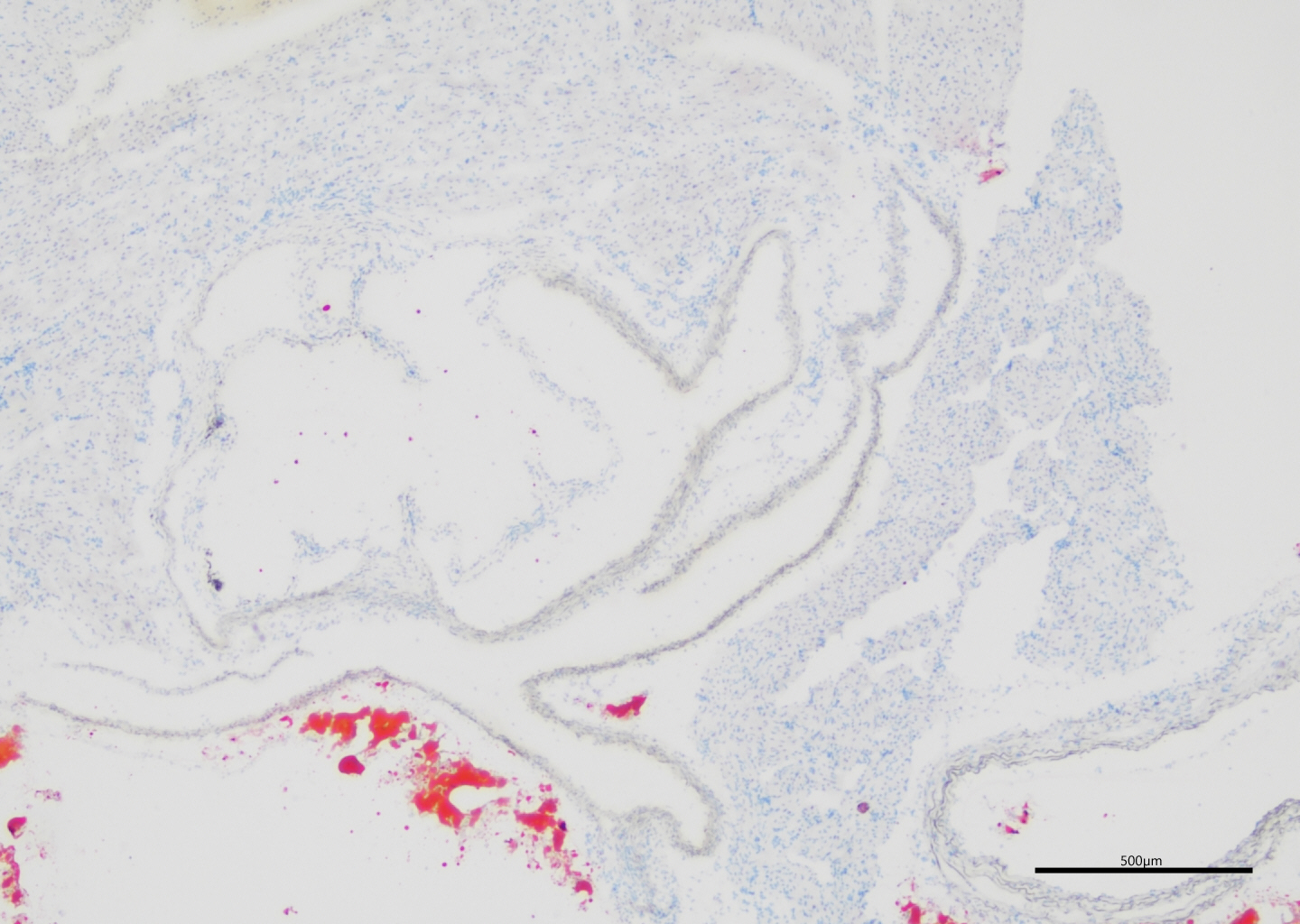

Supplement: S3 File — (ZIP) [file pone.0347758.s003.zip › Oil red O staining of aortic Root/control/6 40-1.jpg]

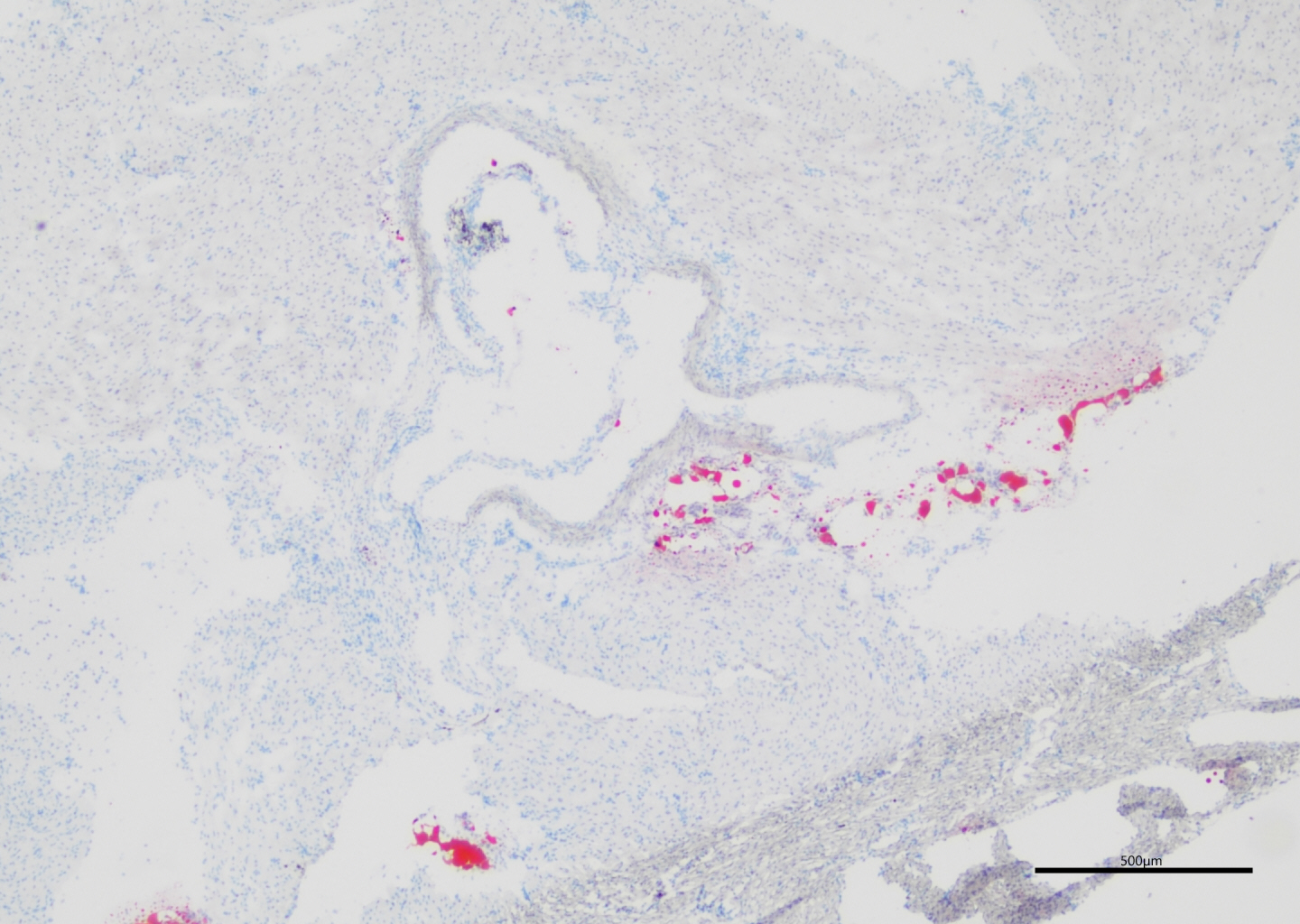

Supplement: S3 File — (ZIP) [file pone.0347758.s003.zip › Oil red O staining of aortic Root/control/7 40-1.jpg]

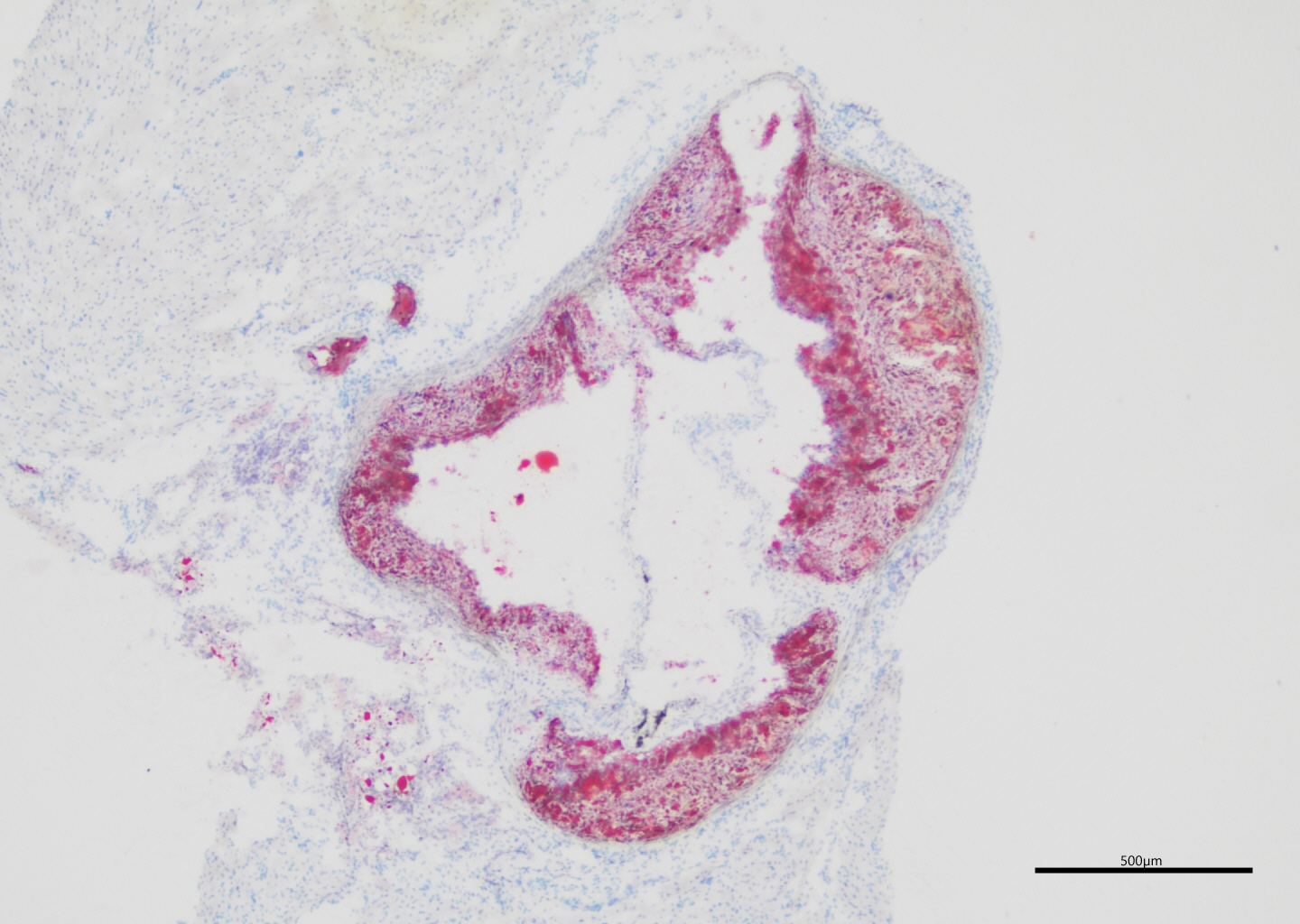

Supplement: S3 File — (ZIP) [file pone.0347758.s003.zip › Oil red O staining of aortic Root/PSB-H/100 40-1.jpg]

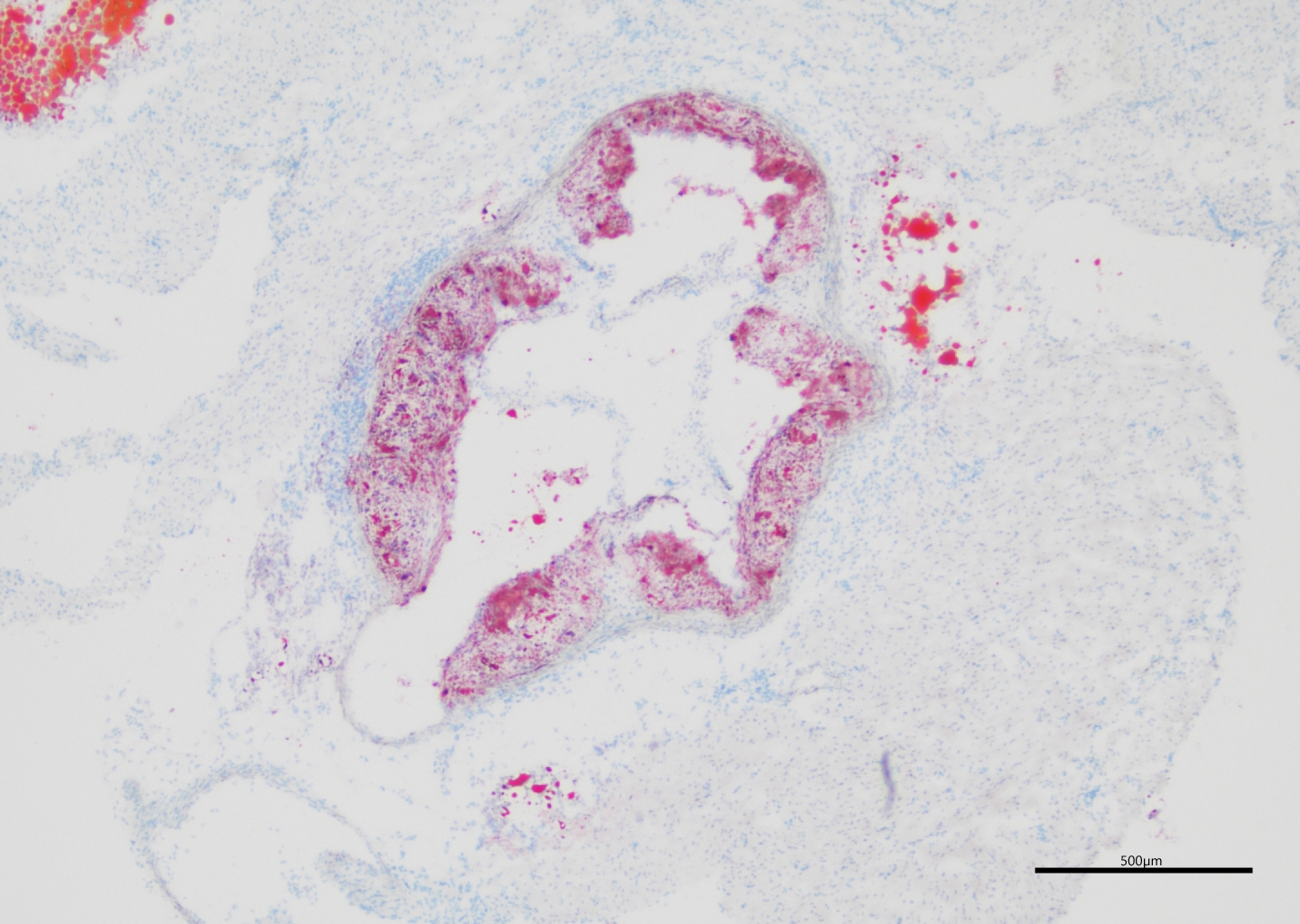

Supplement: S3 File — (ZIP) [file pone.0347758.s003.zip › Oil red O staining of aortic Root/PSB-H/93 40-1.jpg]

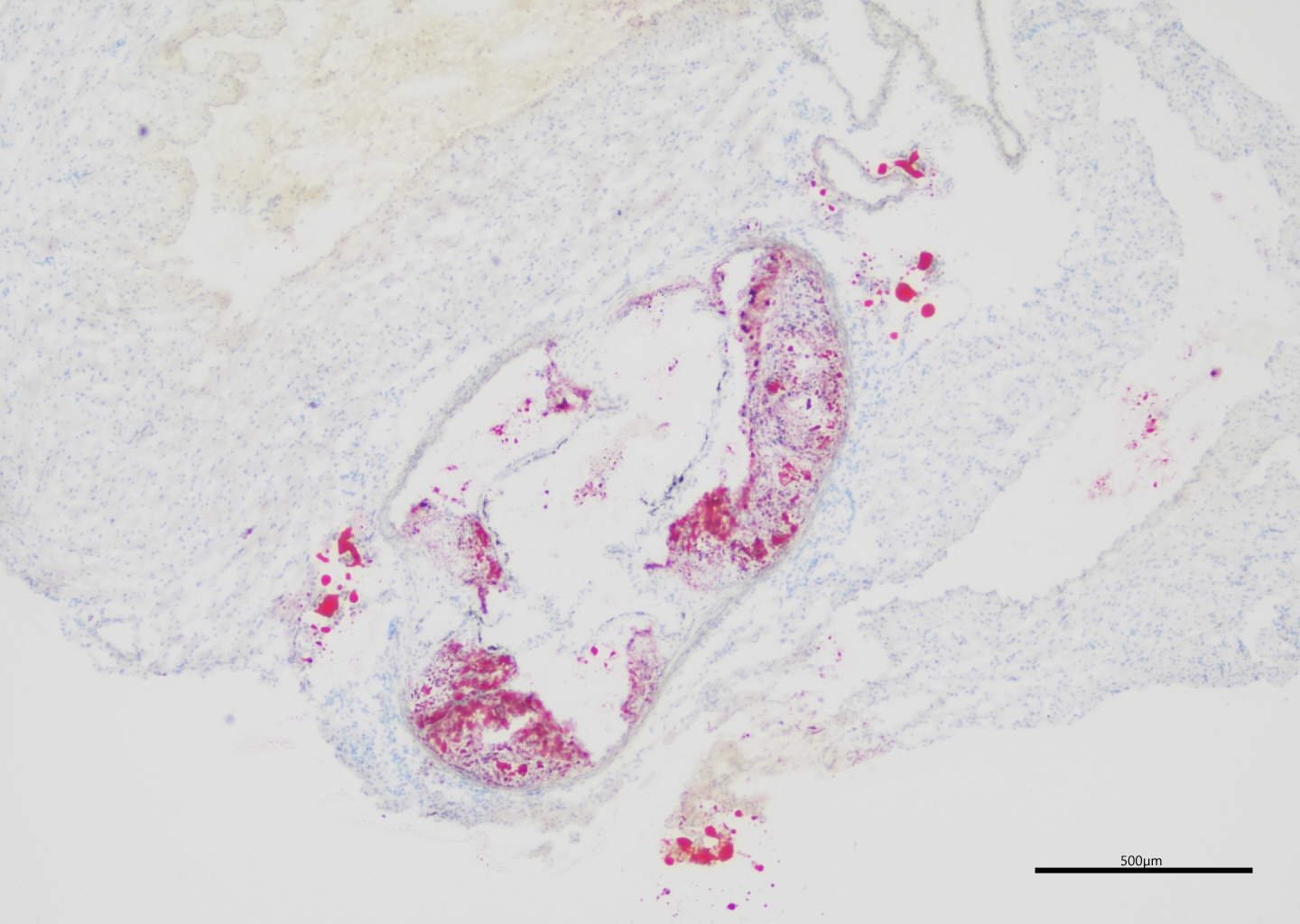

Supplement: S3 File — (ZIP) [file pone.0347758.s003.zip › Oil red O staining of aortic Root/PSB-H/98 40-1.jpg]

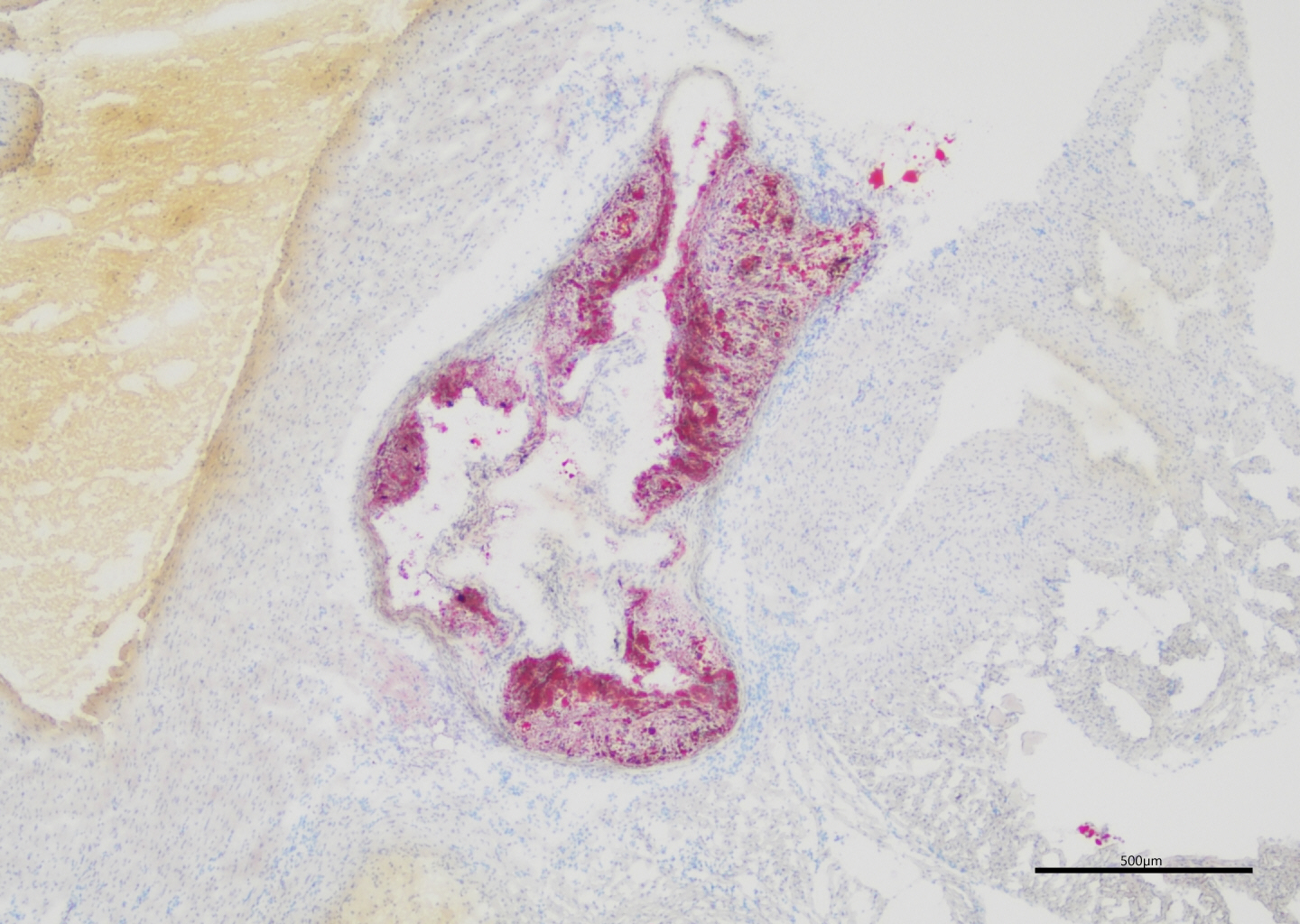

Supplement: S3 File — (ZIP) [file pone.0347758.s003.zip › Oil red O staining of aortic Root/PSB-H/A1 40-1.jpg]

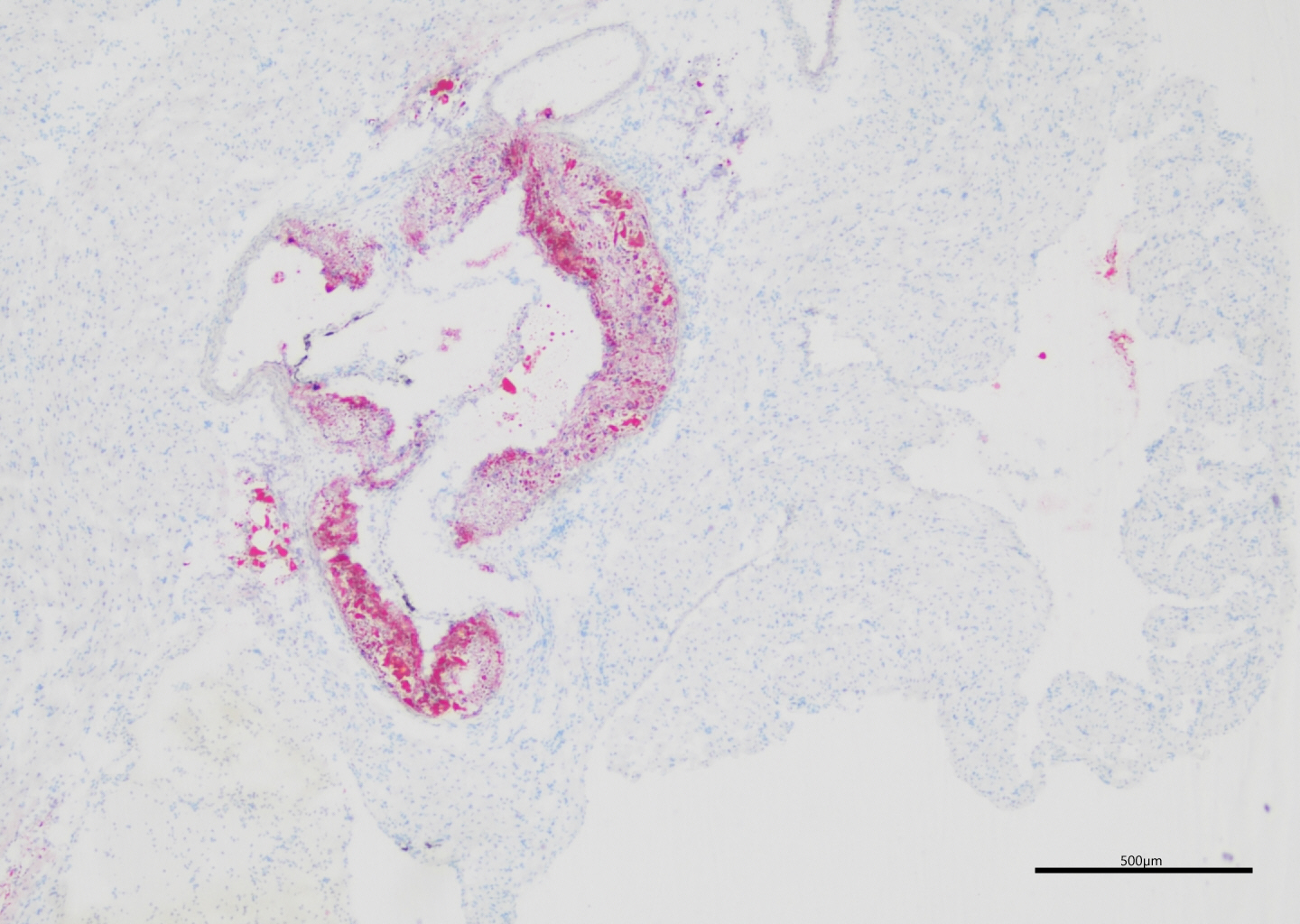

Supplement: S3 File — (ZIP) [file pone.0347758.s003.zip › Oil red O staining of aortic Root/PSB-L/74 40-1.jpg]

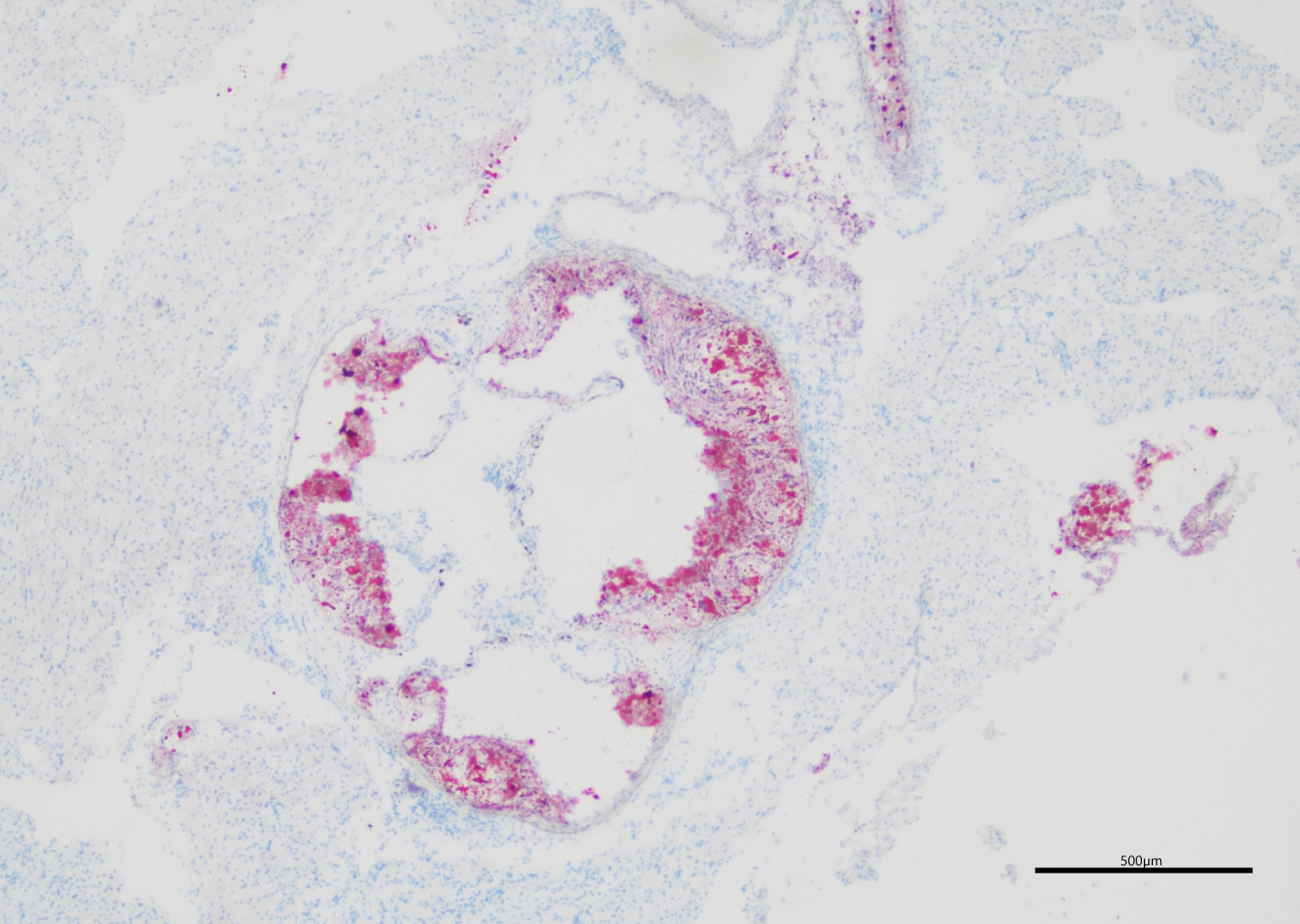

Supplement: S3 File — (ZIP) [file pone.0347758.s003.zip › Oil red O staining of aortic Root/PSB-L/77 40-1.jpg]

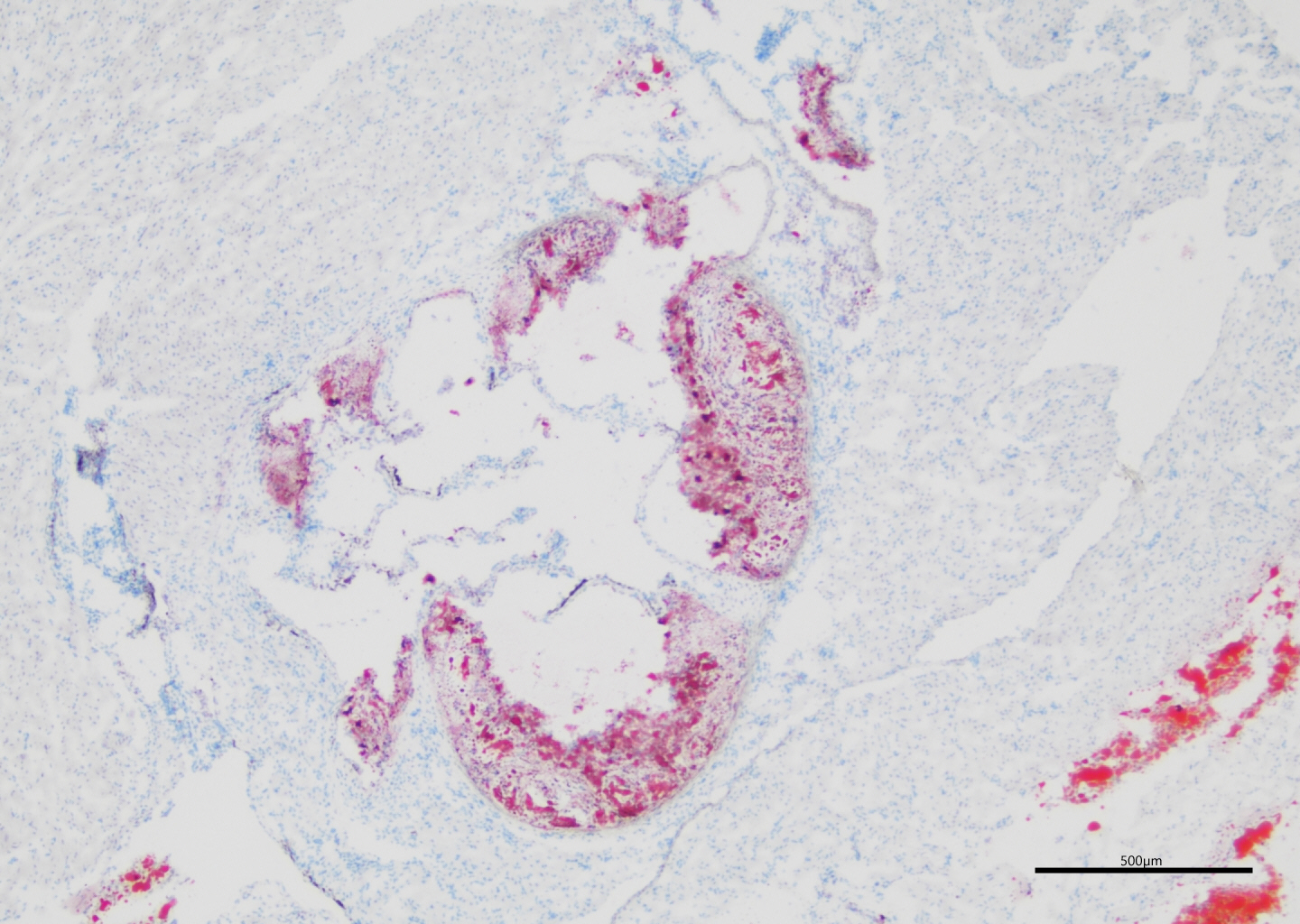

Supplement: S3 File — (ZIP) [file pone.0347758.s003.zip › Oil red O staining of aortic Root/PSB-L/80 40-1.jpg]

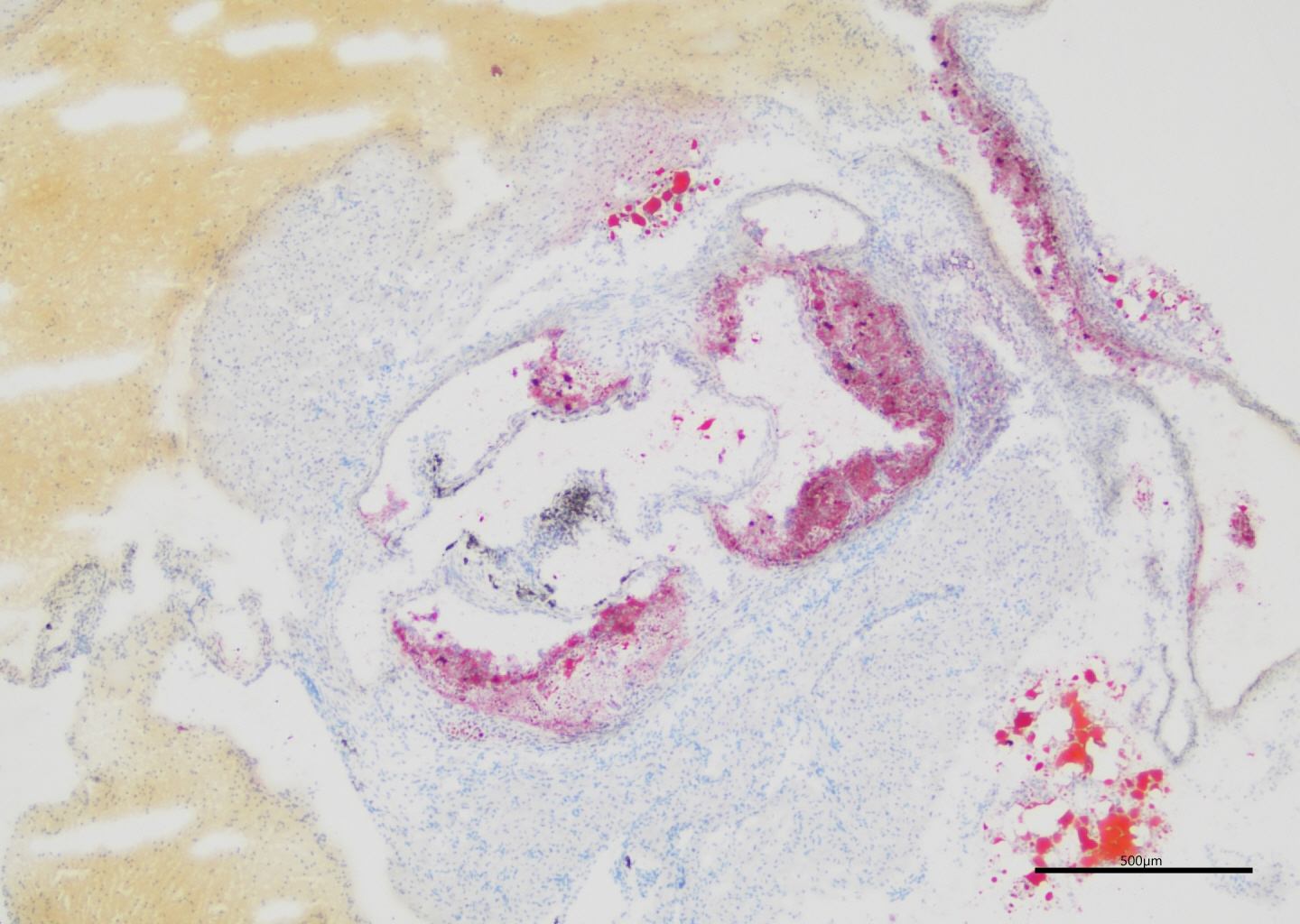

Supplement: S3 File — (ZIP) [file pone.0347758.s003.zip › Oil red O staining of aortic Root/PSB-L/82 40-1.jpg]

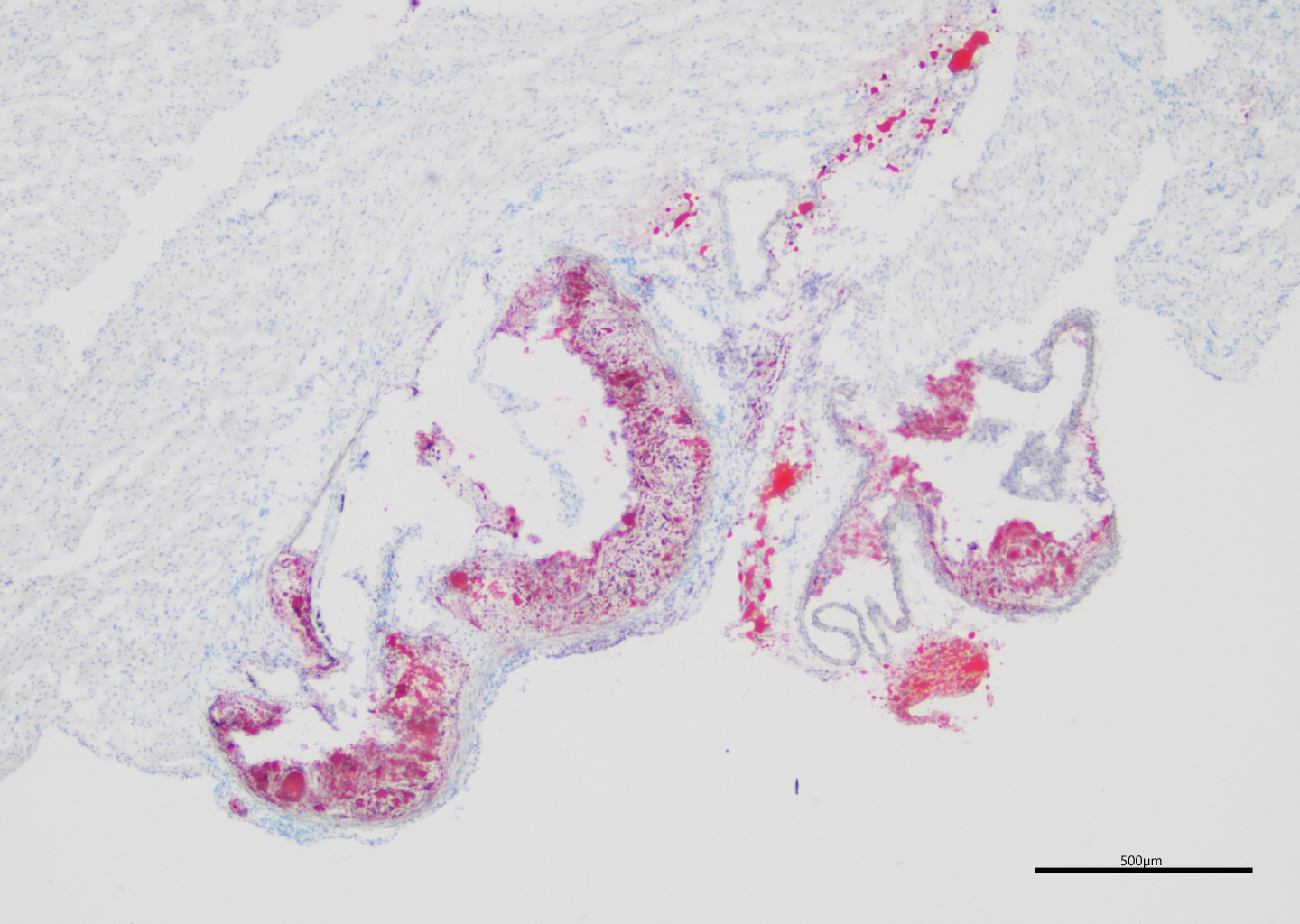

Supplement: S3 File — (ZIP) [file pone.0347758.s003.zip › Oil red O staining of aortic Root/PSB-M/85 40-1.jpg]

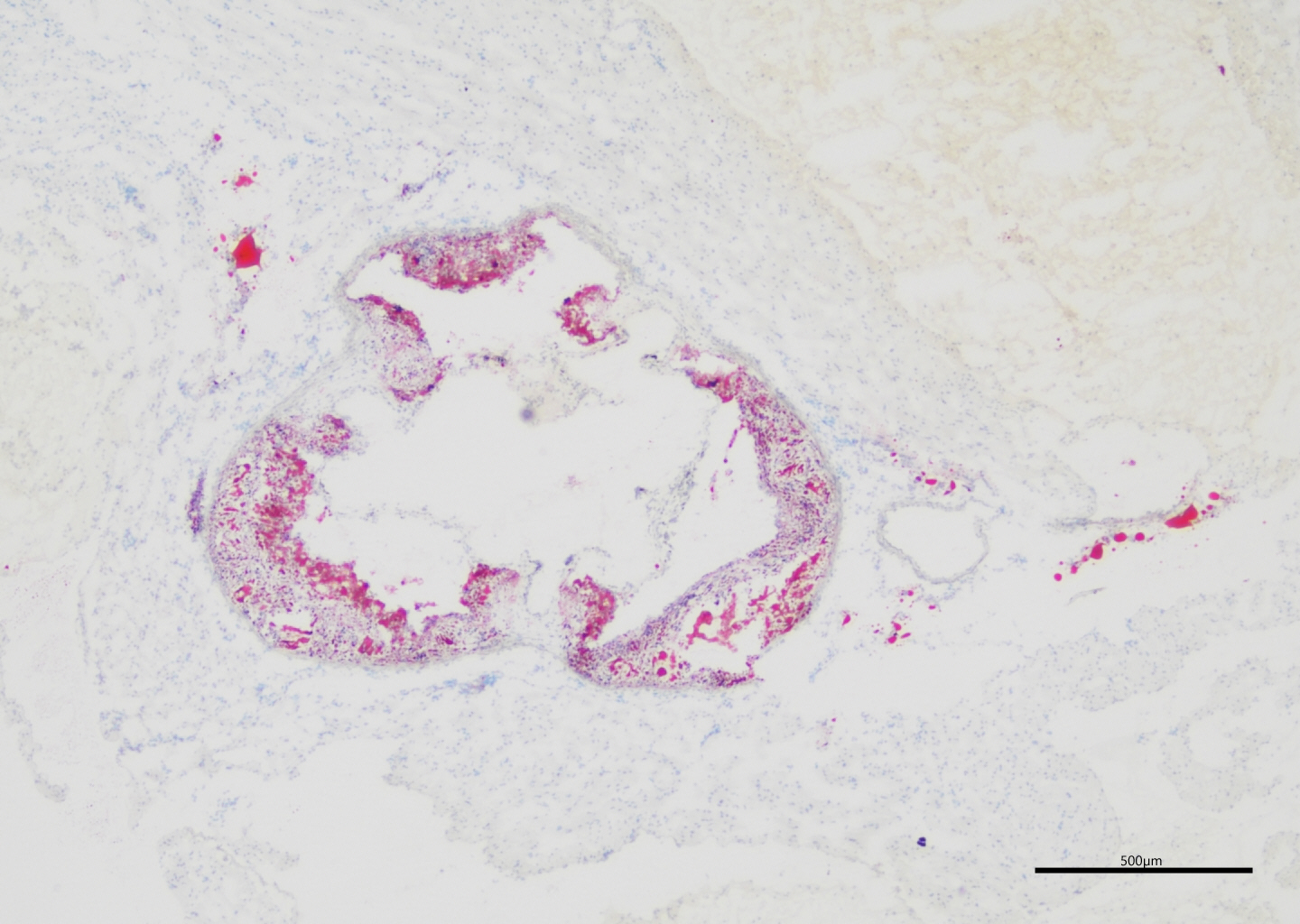

Supplement: S3 File — (ZIP) [file pone.0347758.s003.zip › Oil red O staining of aortic Root/PSB-M/89 40-1.jpg]

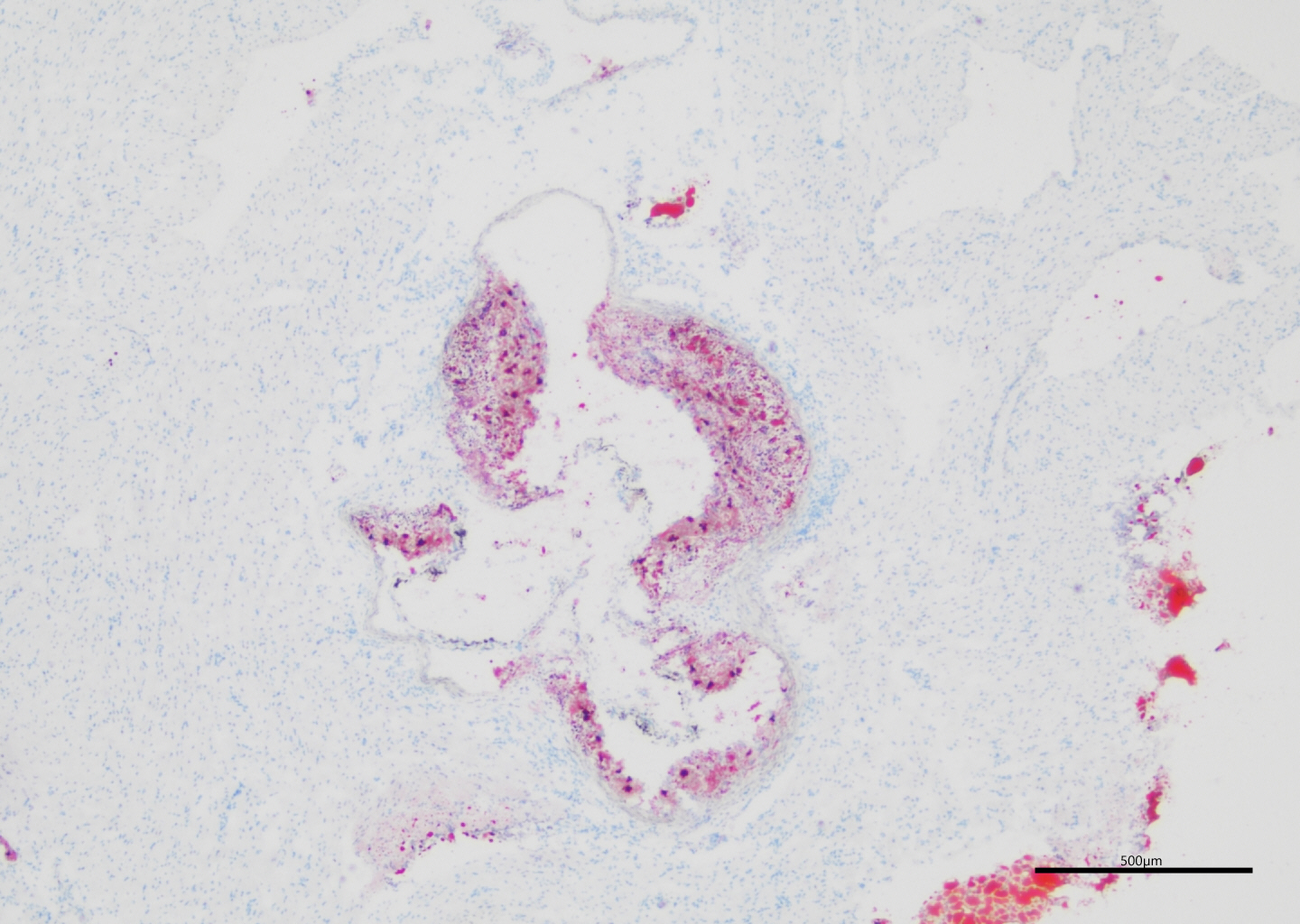

Supplement: S3 File — (ZIP) [file pone.0347758.s003.zip › Oil red O staining of aortic Root/PSB-M/90 40-1.jpg]

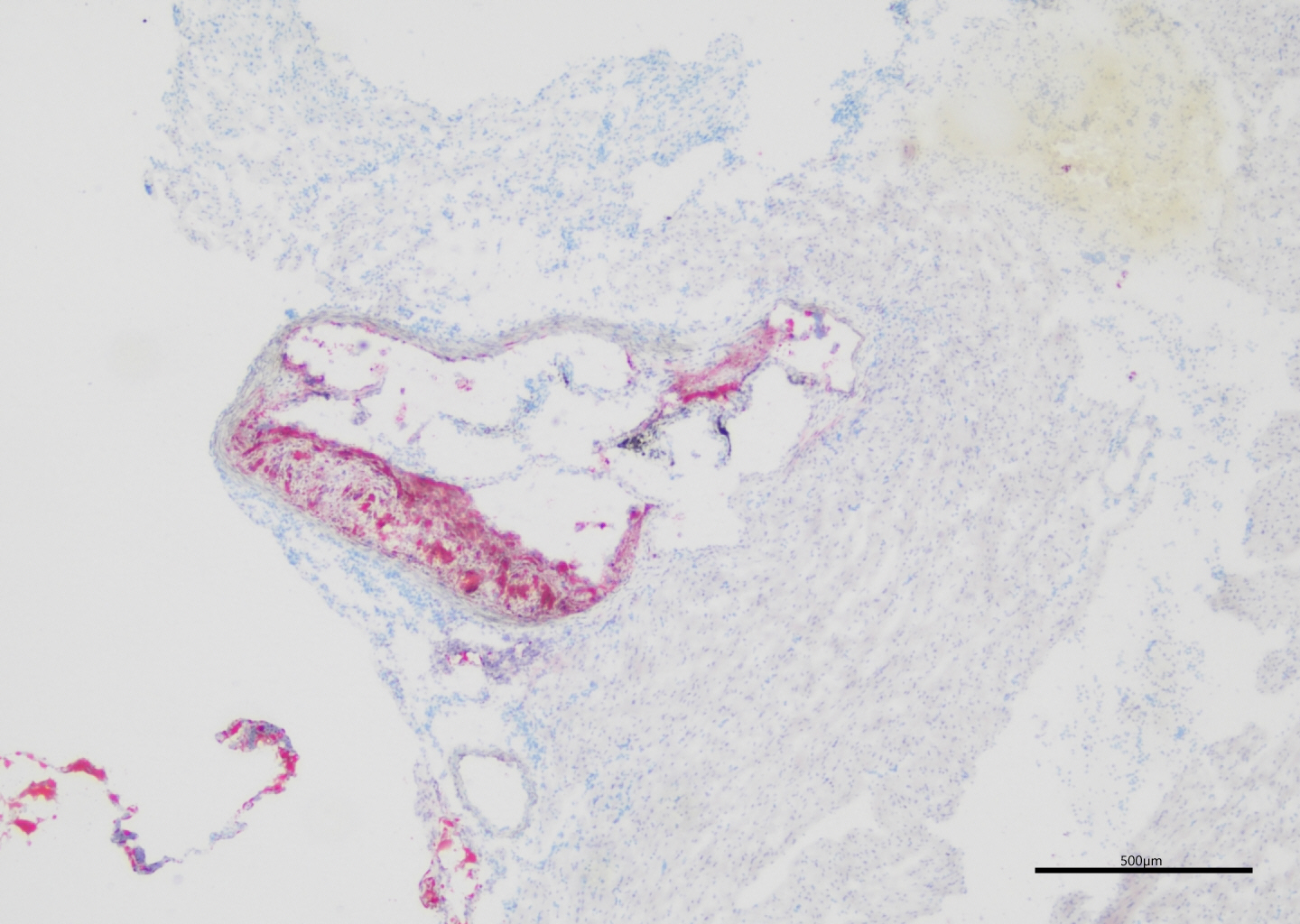

Supplement: S3 File — (ZIP) [file pone.0347758.s003.zip › Oil red O staining of aortic Root/PSB-M/92 40-1.jpg]

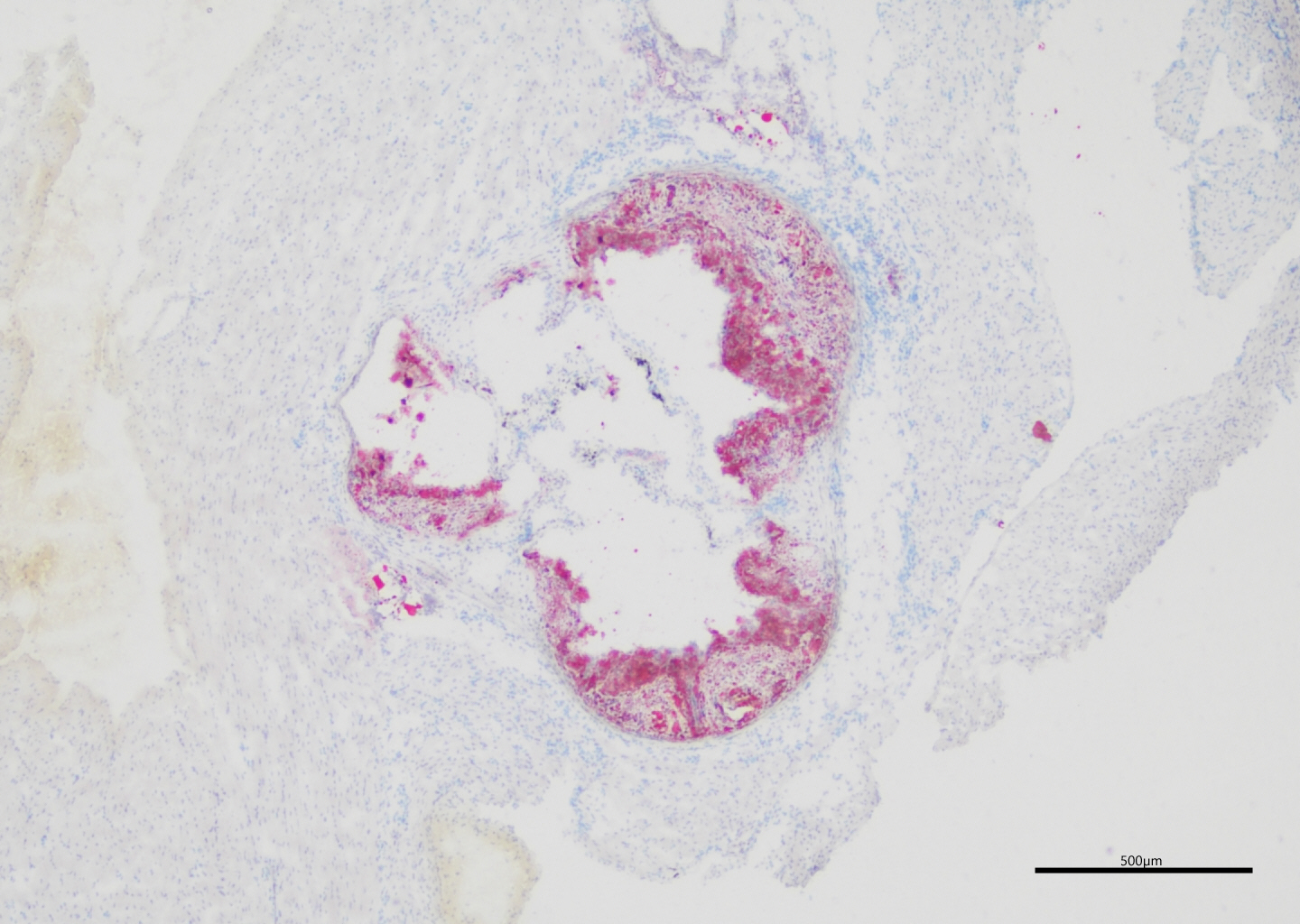

Supplement: S3 File — (ZIP) [file pone.0347758.s003.zip › Oil red O staining of aortic Root/statin/37 40-1.jpg]

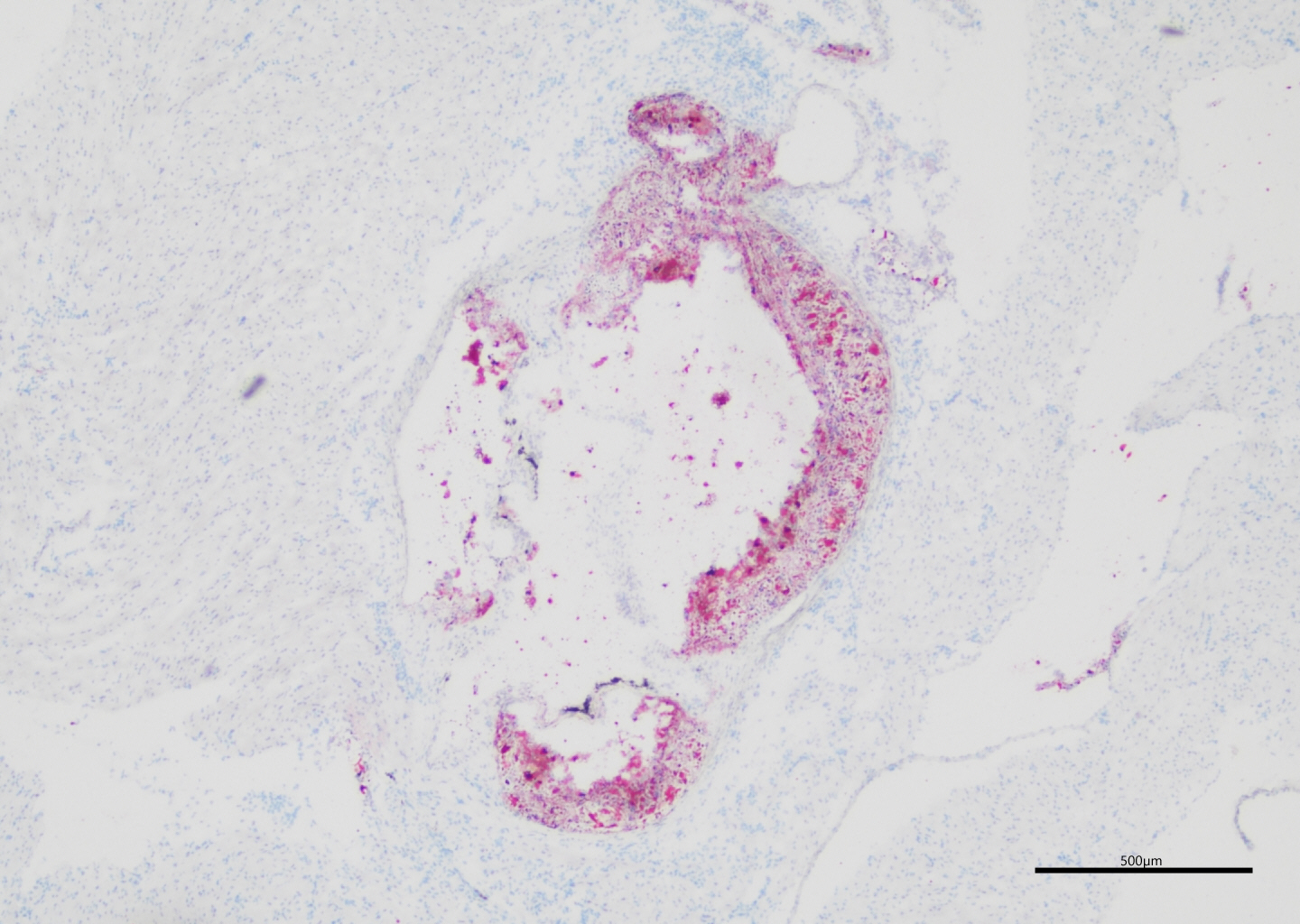

Supplement: S3 File — (ZIP) [file pone.0347758.s003.zip › Oil red O staining of aortic Root/statin/38 40-1.jpg]

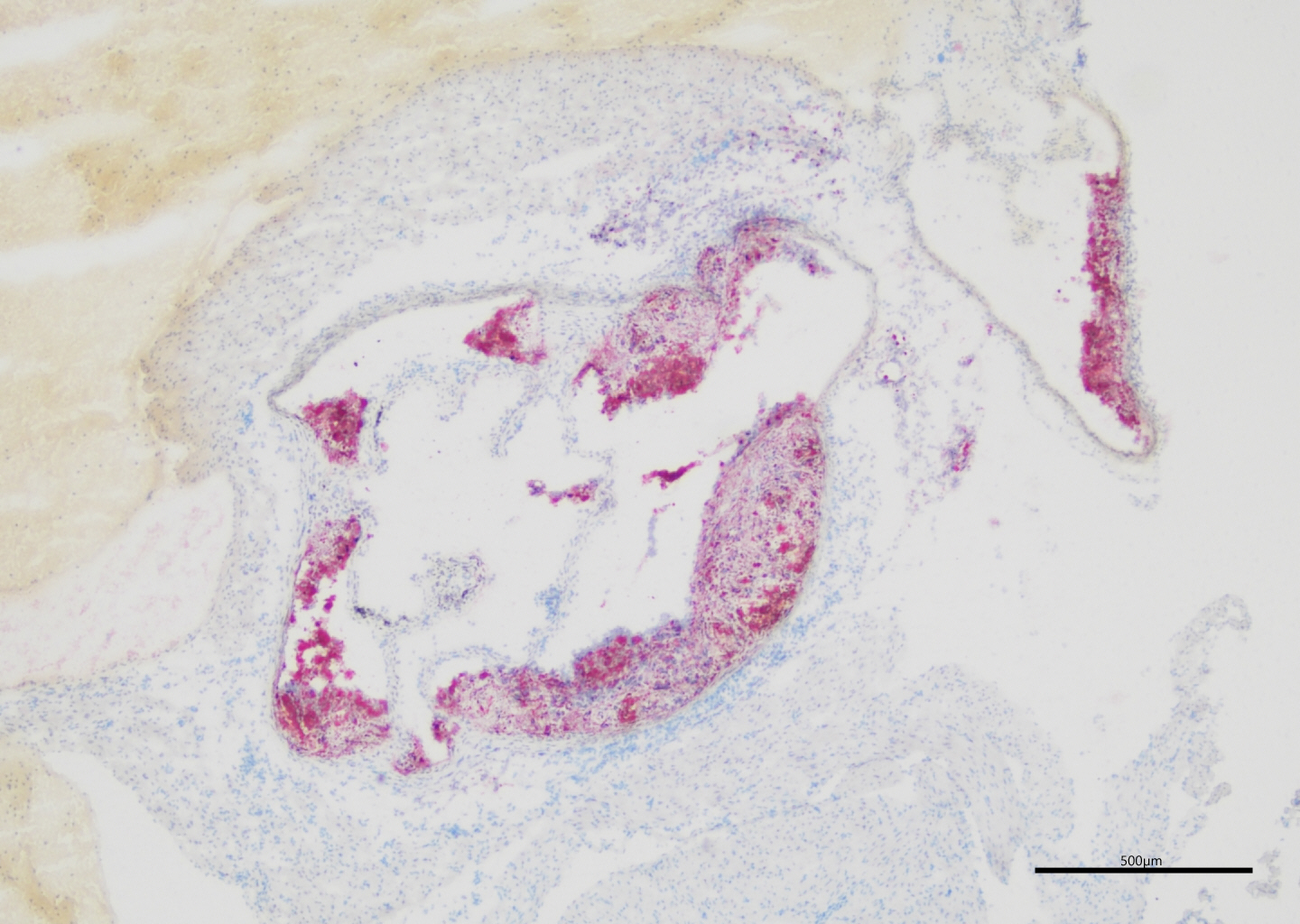

Supplement: S3 File — (ZIP) [file pone.0347758.s003.zip › Oil red O staining of aortic Root/statin/40 40-1.jpg]

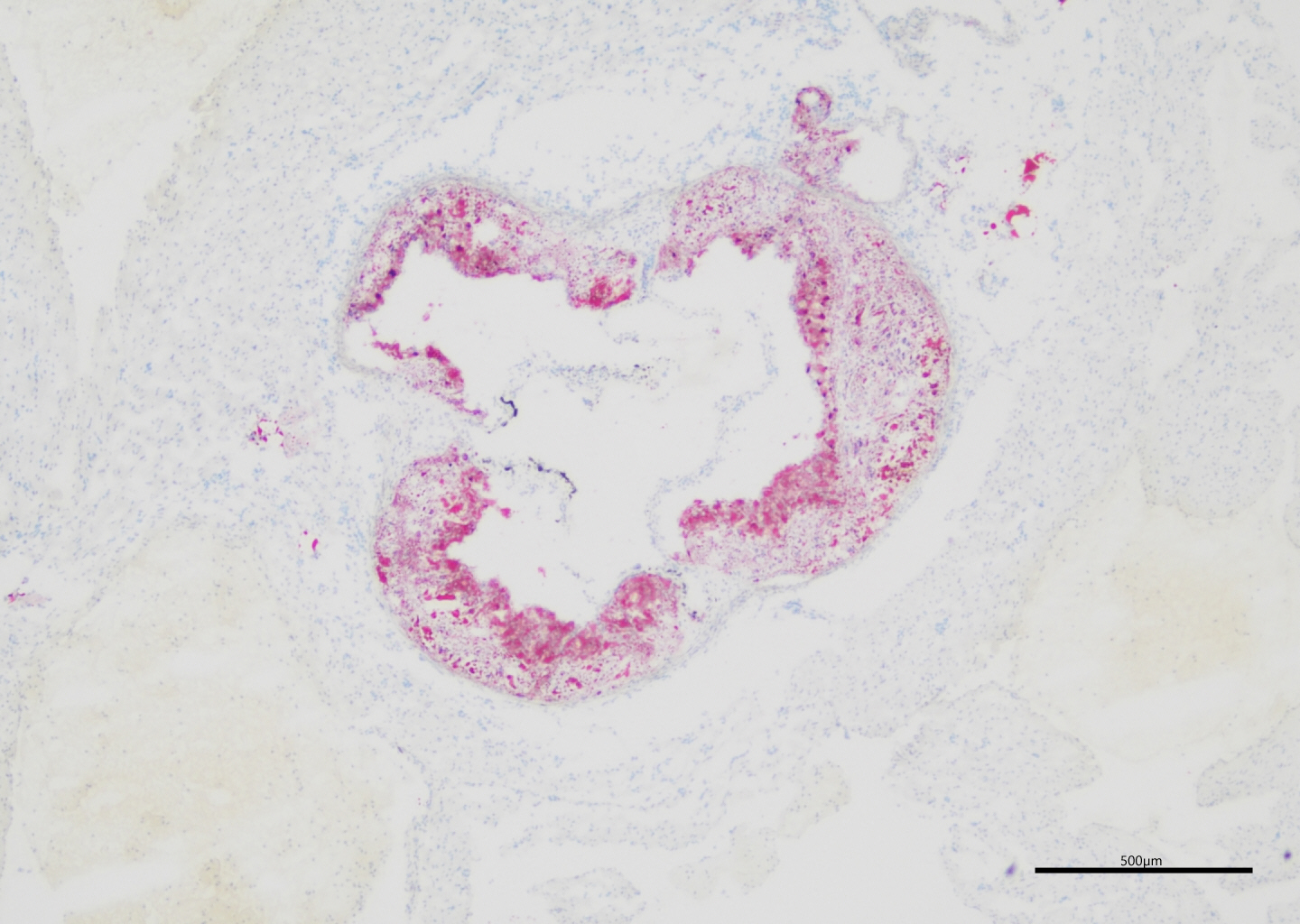

Supplement: S3 File — (ZIP) [file pone.0347758.s003.zip › Oil red O staining of aortic Root/statin/41 40-1.jpg]

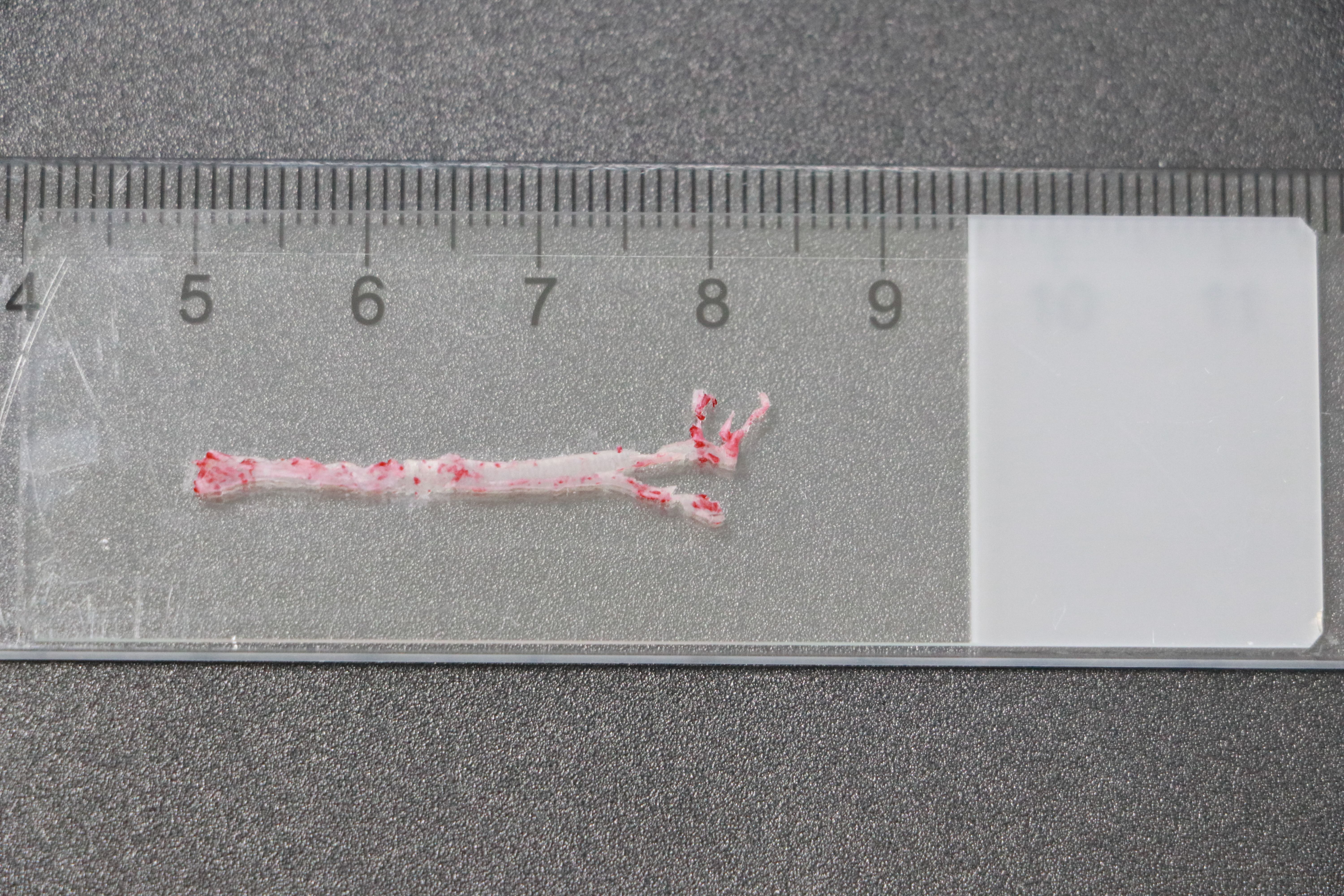

Supplement: S4 File — (ZIP) [file pone.0347758.s004.zip › Oil red O staining of aorta/AS/23.JPG]

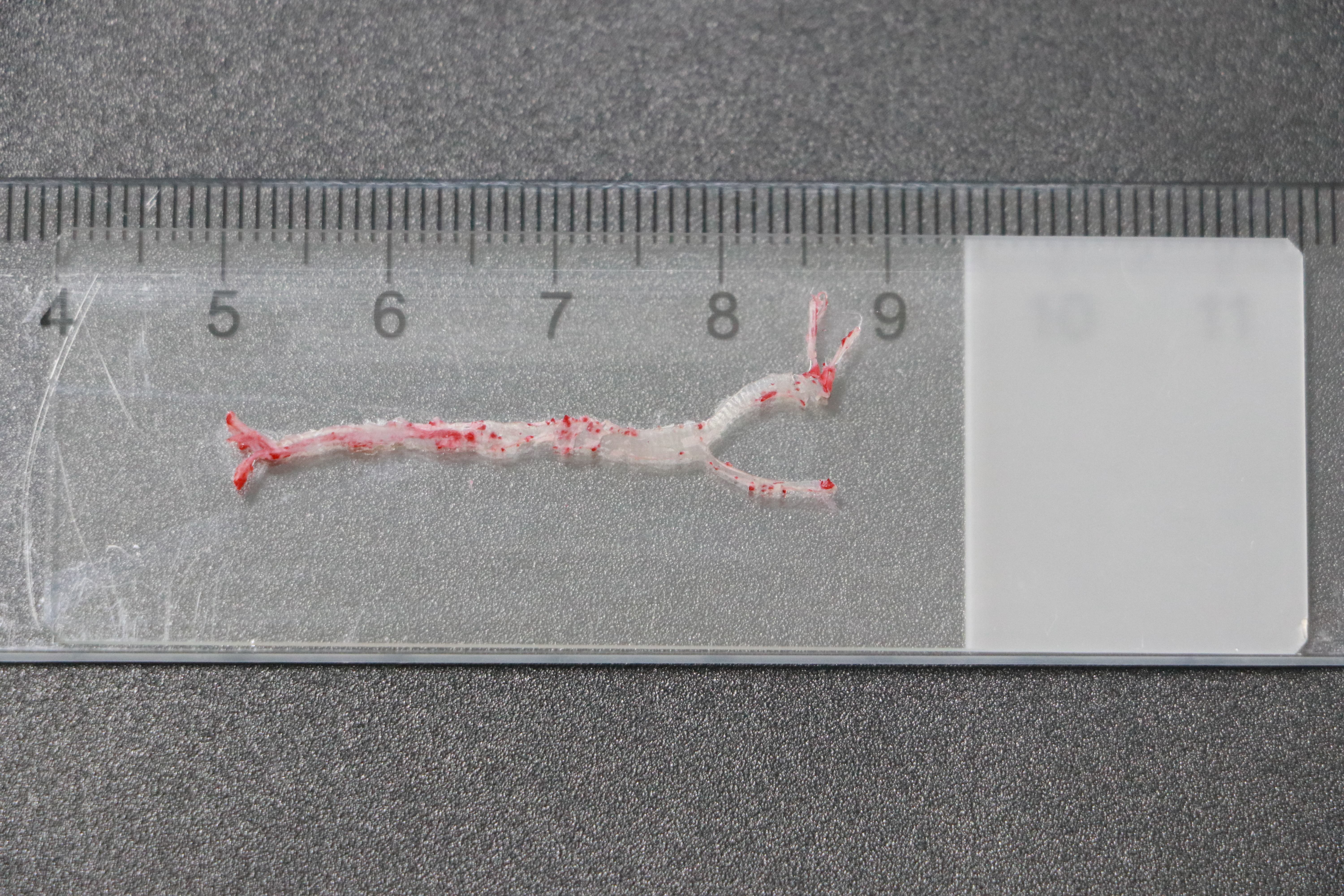

Supplement: S4 File — (ZIP) [file pone.0347758.s004.zip › Oil red O staining of aorta/AS/27.JPG]

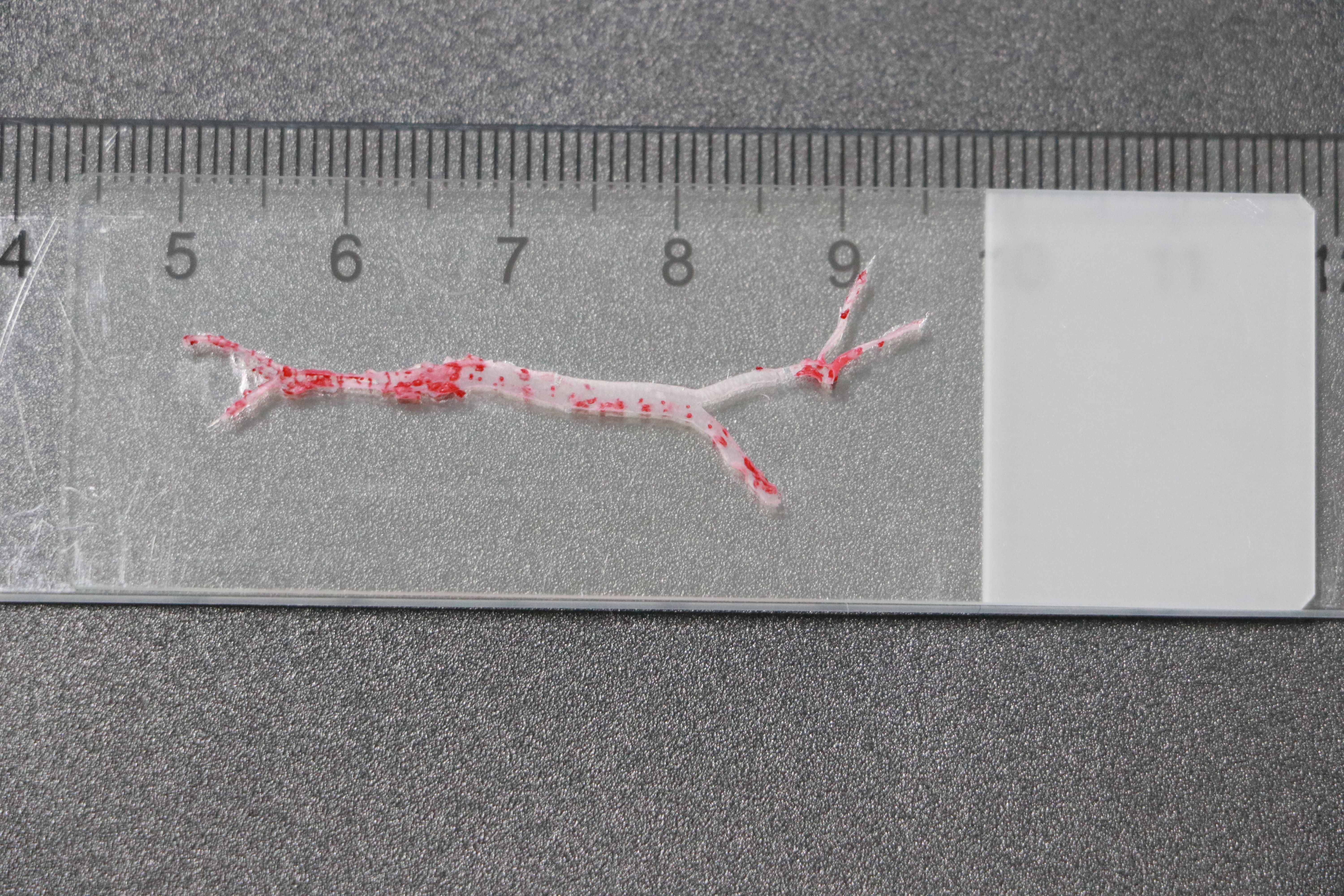

Supplement: S4 File — (ZIP) [file pone.0347758.s004.zip › Oil red O staining of aorta/AS/28.JPG]

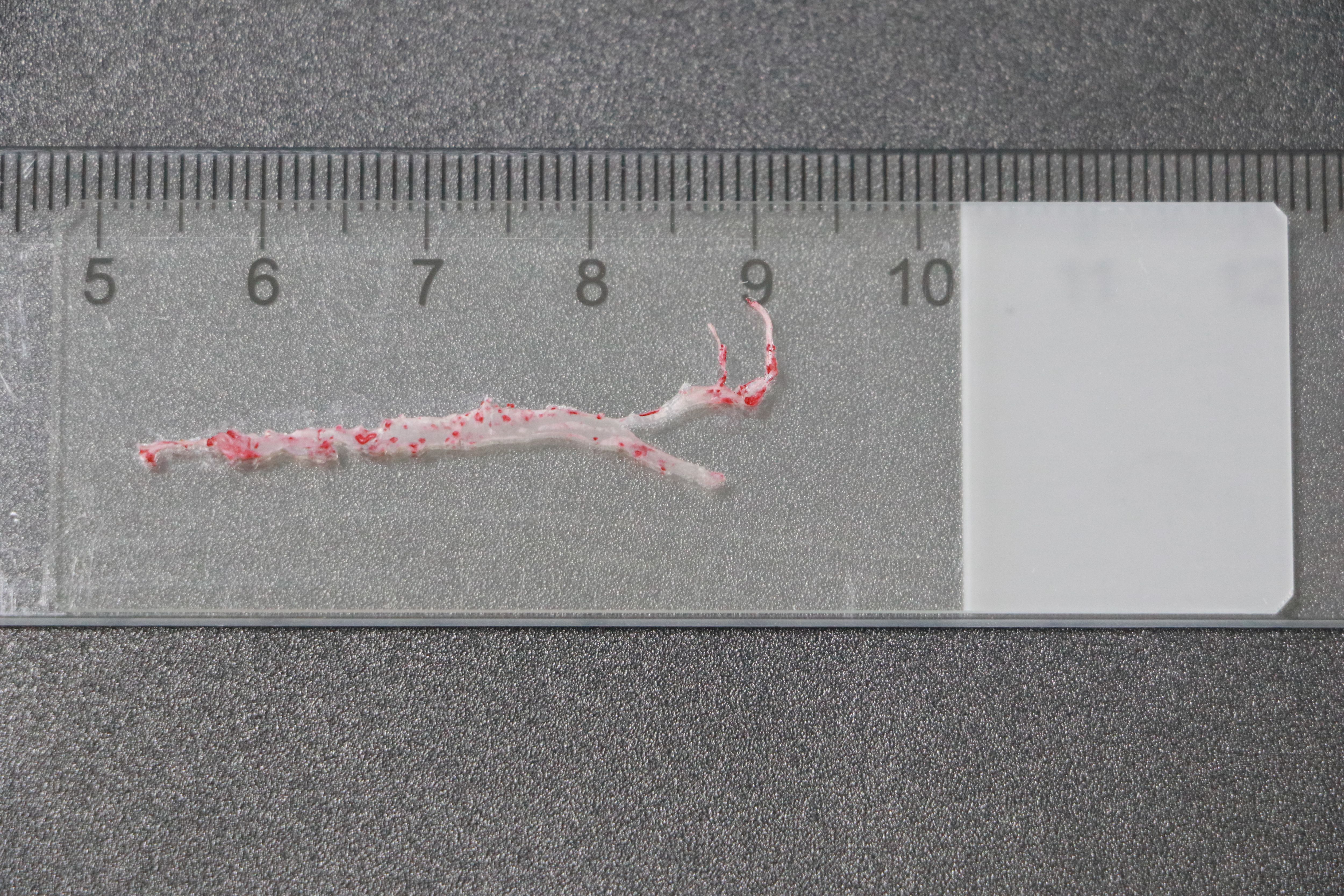

Supplement: S4 File — (ZIP) [file pone.0347758.s004.zip › Oil red O staining of aorta/AS/31.JPG]

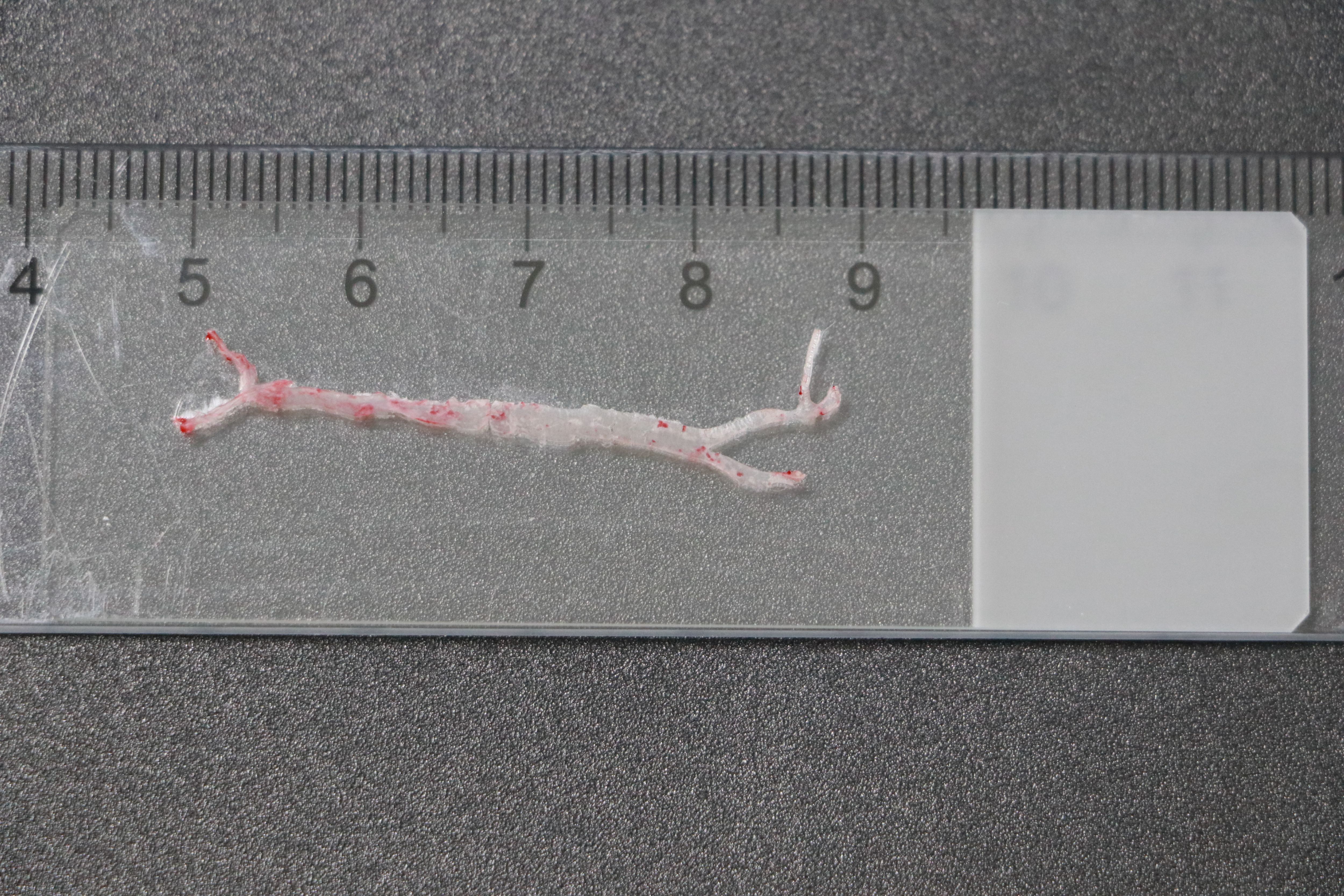

Supplement: S4 File — (ZIP) [file pone.0347758.s004.zip › Oil red O staining of aorta/control/1.JPG]

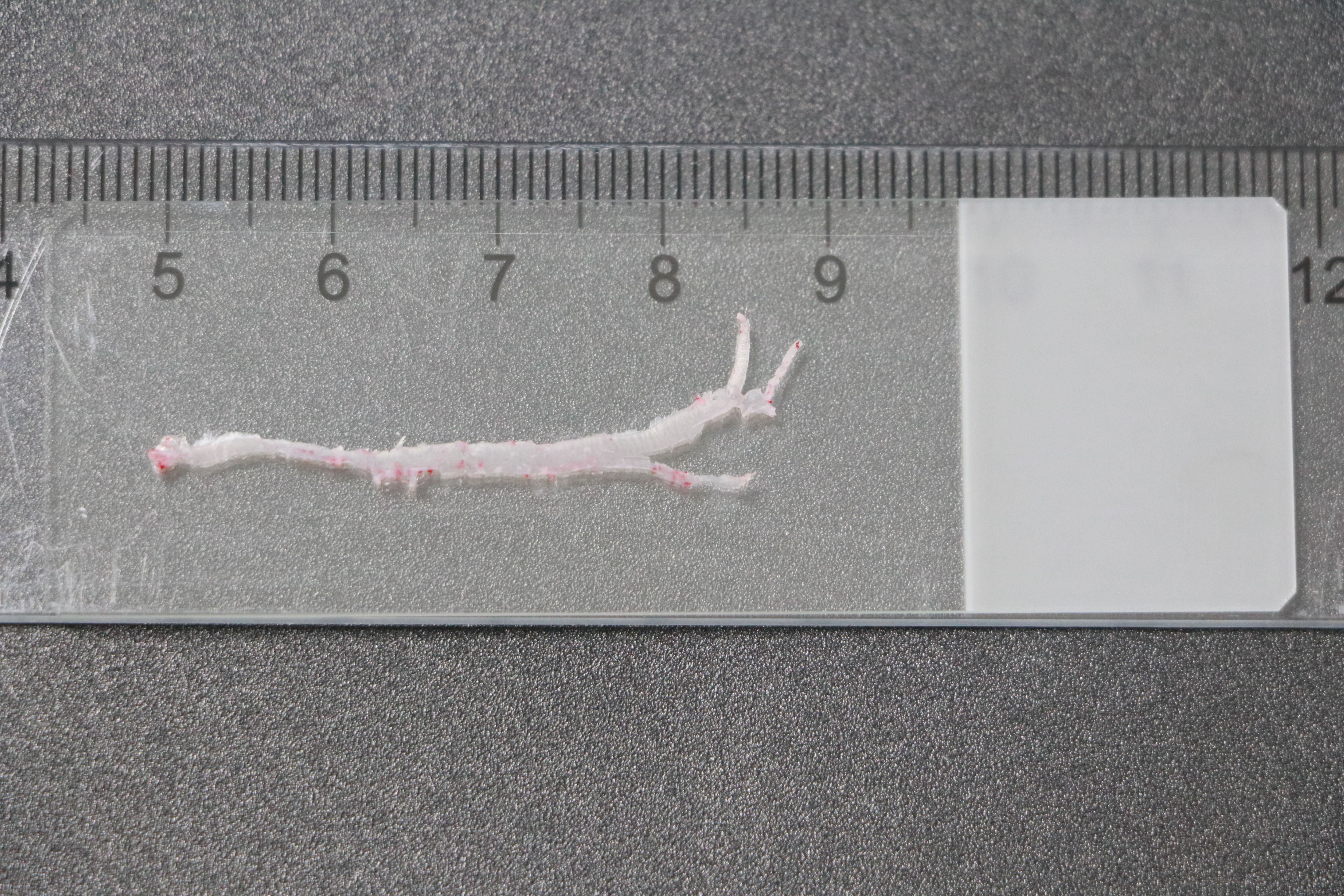

Supplement: S4 File — (ZIP) [file pone.0347758.s004.zip › Oil red O staining of aorta/control/2.JPG]

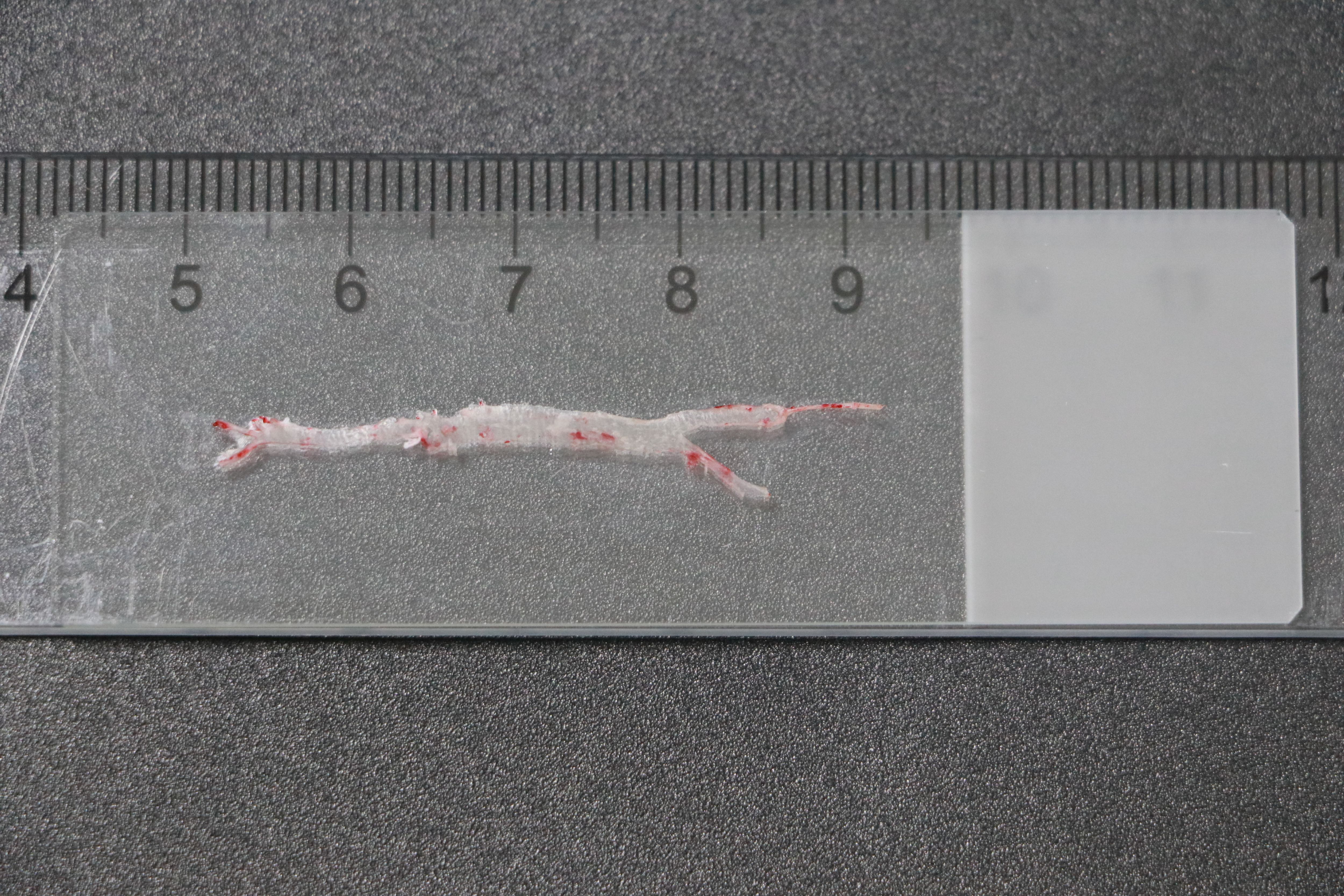

Supplement: S4 File — (ZIP) [file pone.0347758.s004.zip › Oil red O staining of aorta/control/6.JPG]

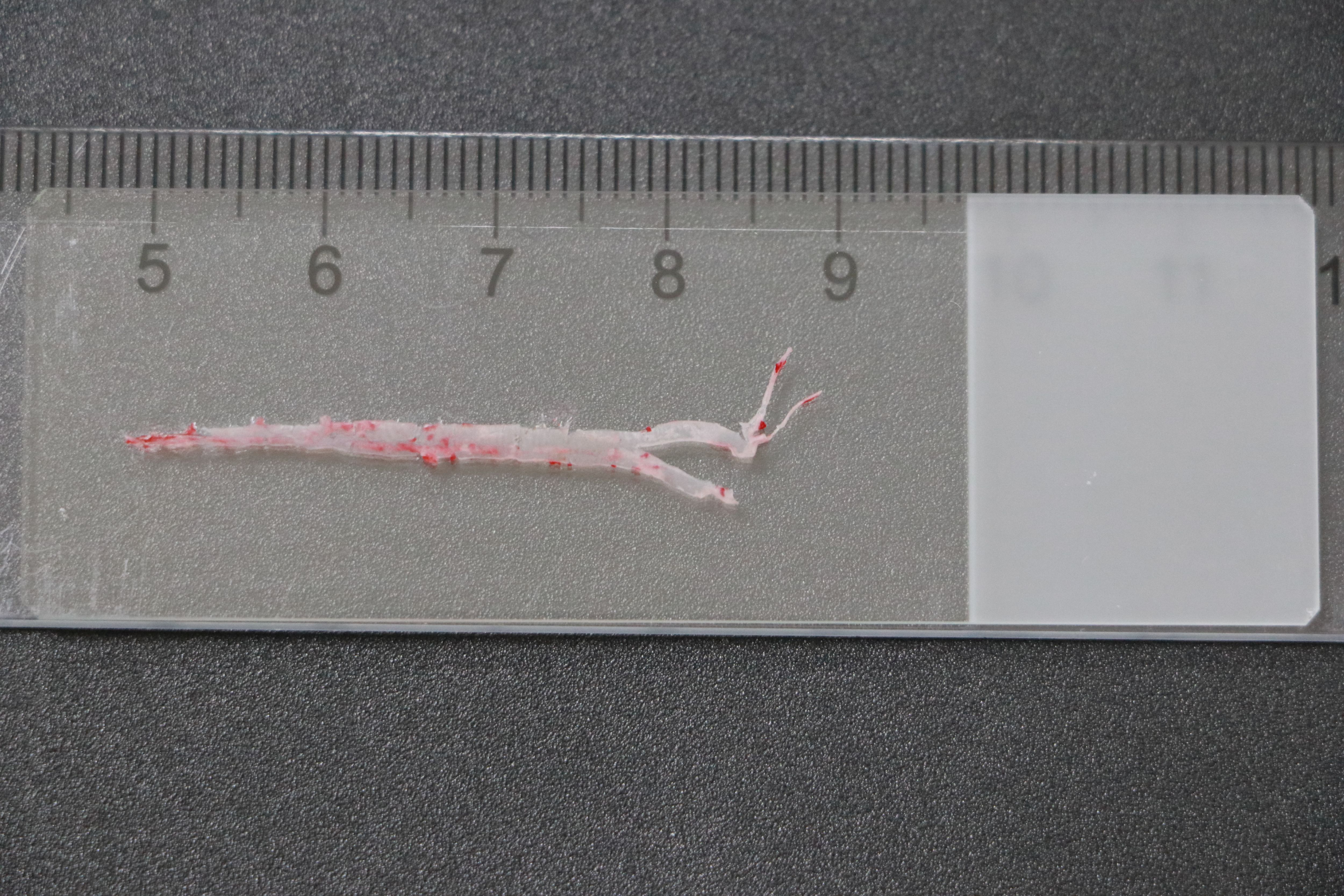

Supplement: S4 File — (ZIP) [file pone.0347758.s004.zip › Oil red O staining of aorta/control/7.JPG]

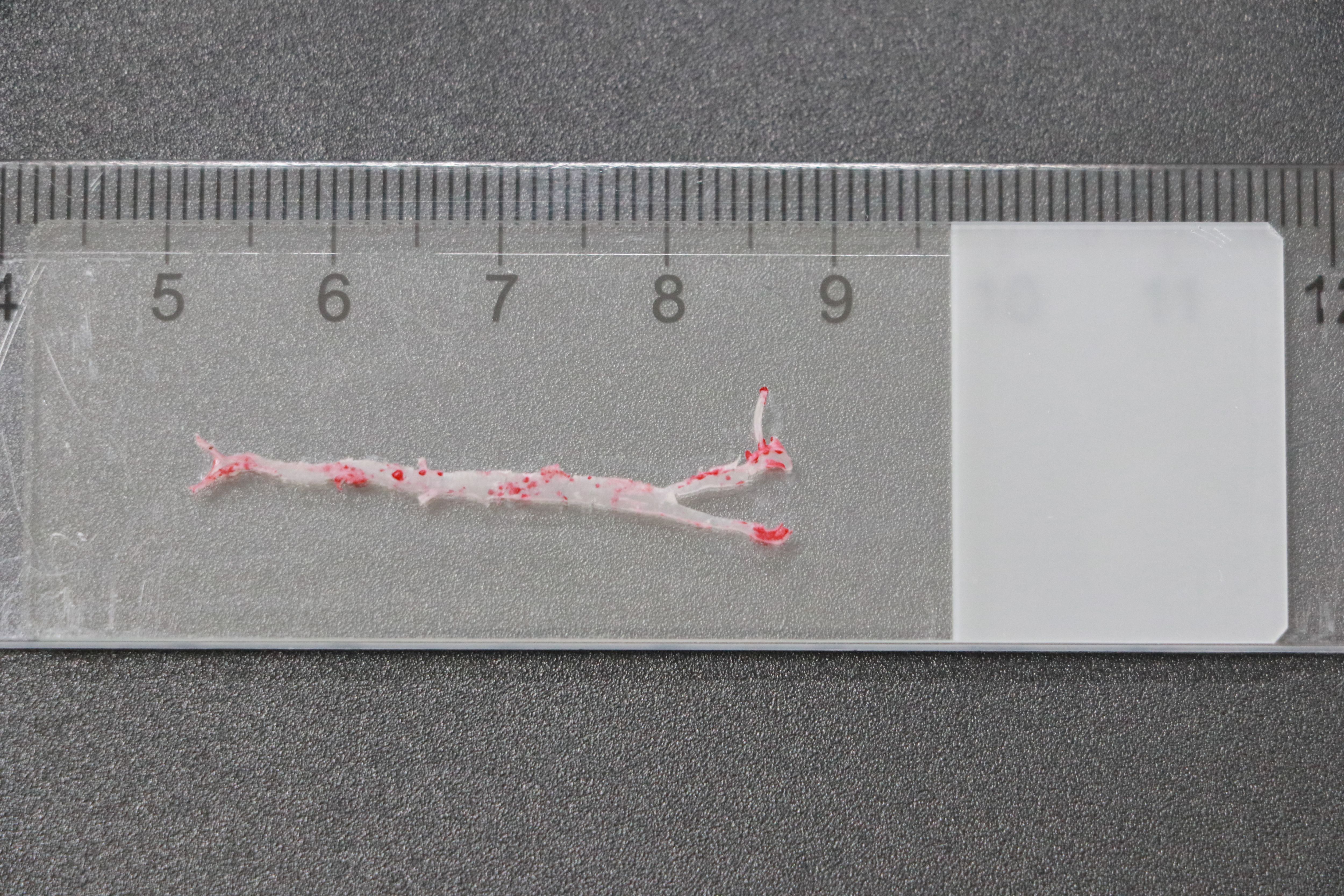

Supplement: S4 File — (ZIP) [file pone.0347758.s004.zip › Oil red O staining of aorta/PSB-H/100.JPG]

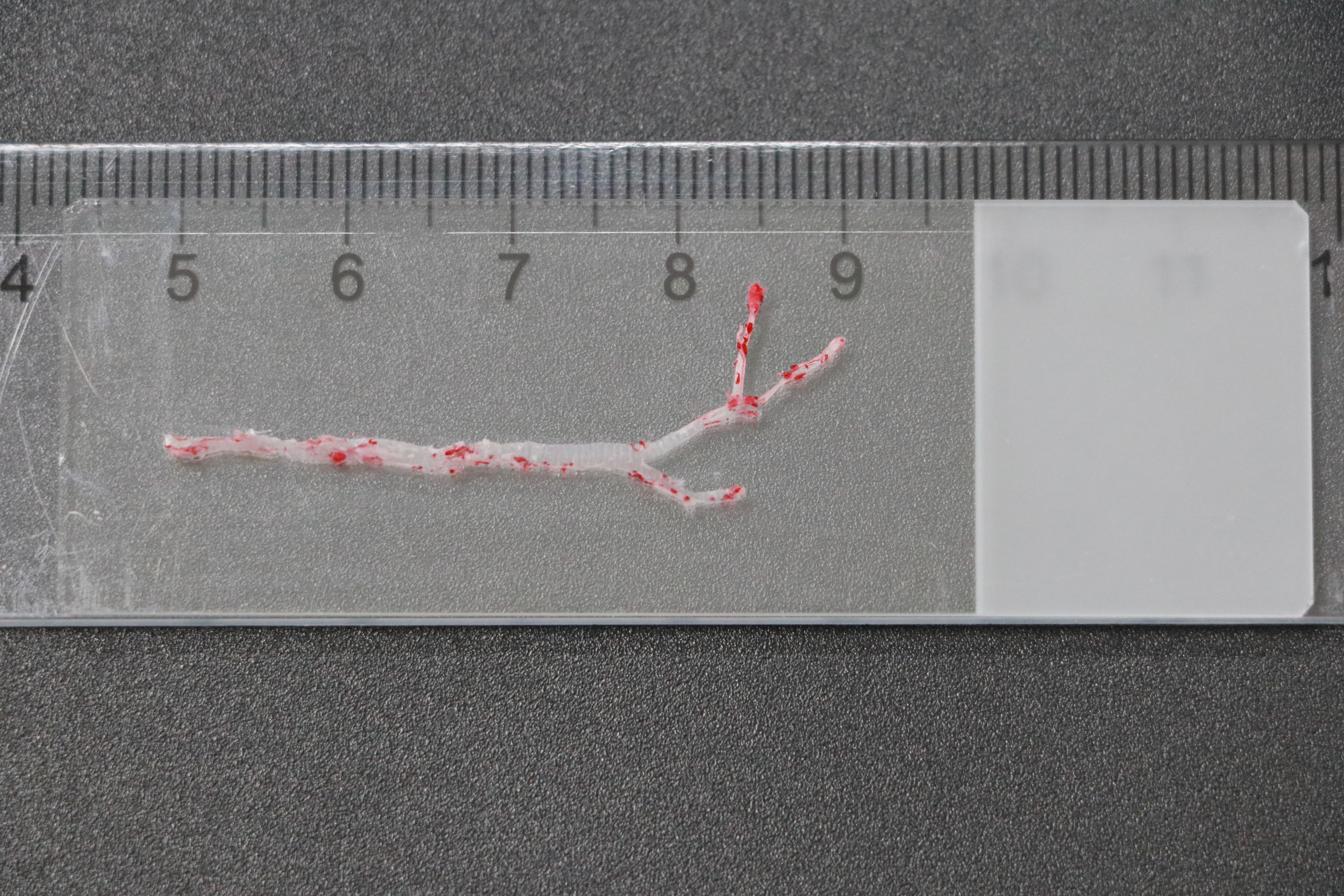

Supplement: S4 File — (ZIP) [file pone.0347758.s004.zip › Oil red O staining of aorta/PSB-H/93.JPG]

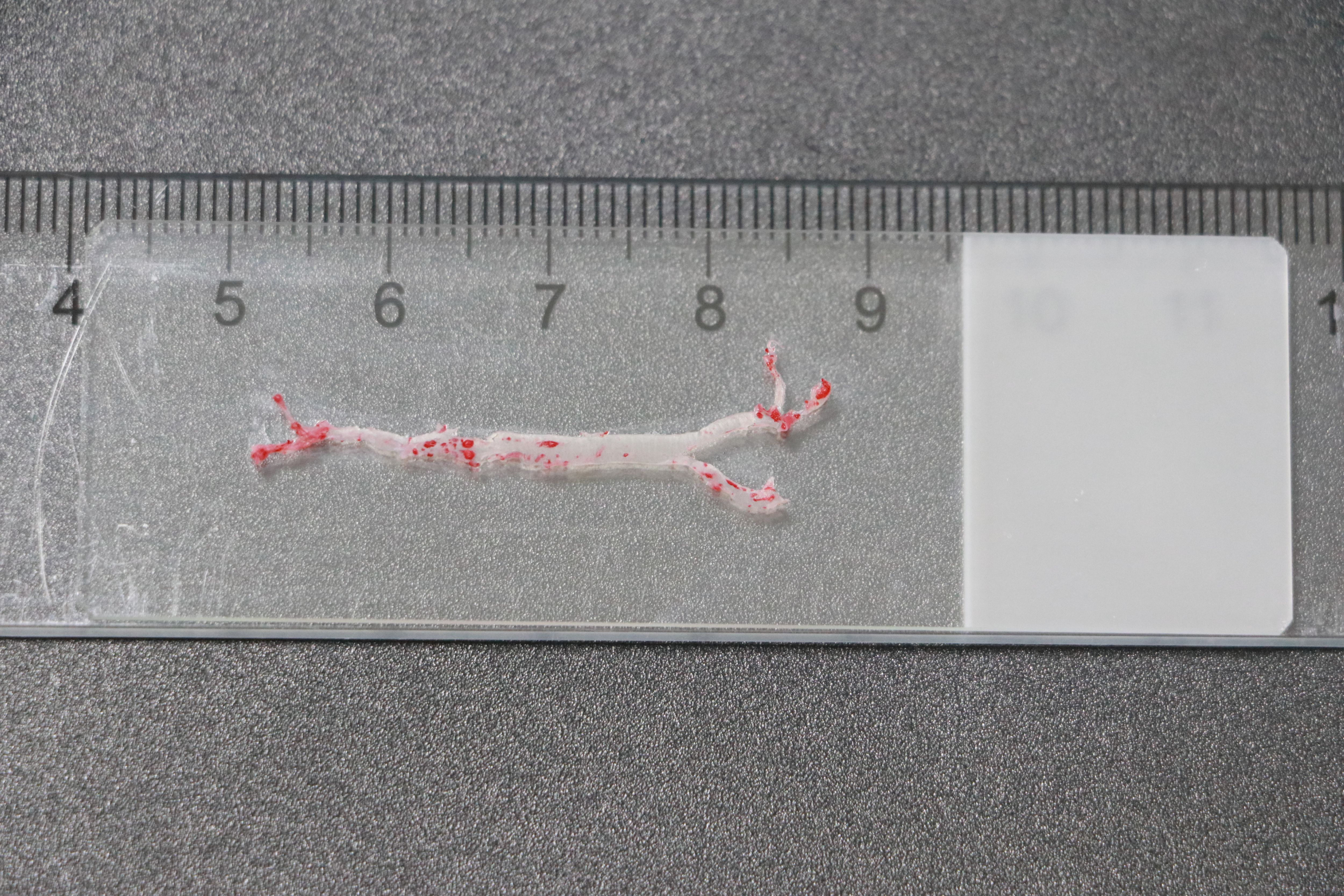

Supplement: S4 File — (ZIP) [file pone.0347758.s004.zip › Oil red O staining of aorta/PSB-H/94.JPG]

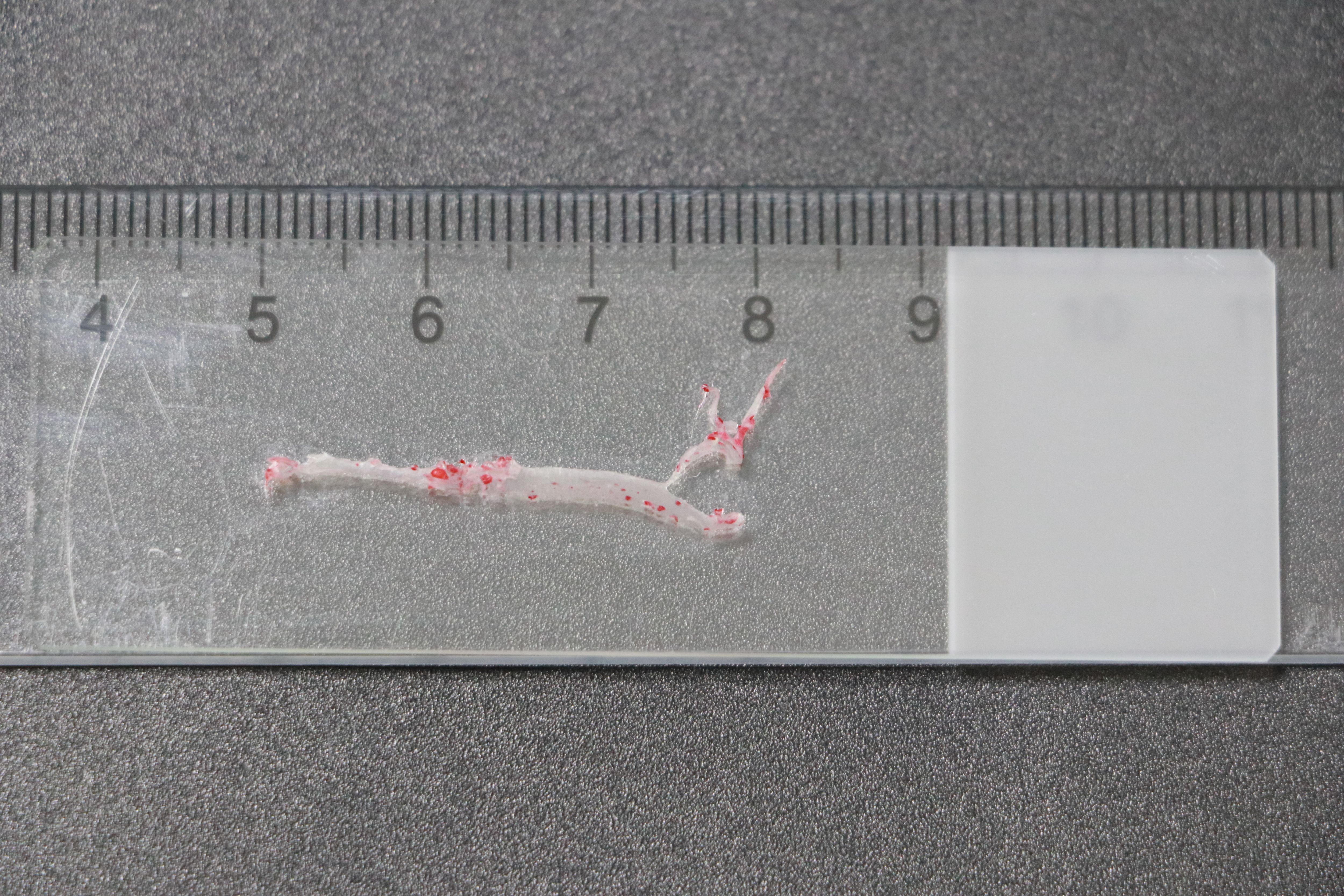

Supplement: S4 File — (ZIP) [file pone.0347758.s004.zip › Oil red O staining of aorta/PSB-H/98.JPG]

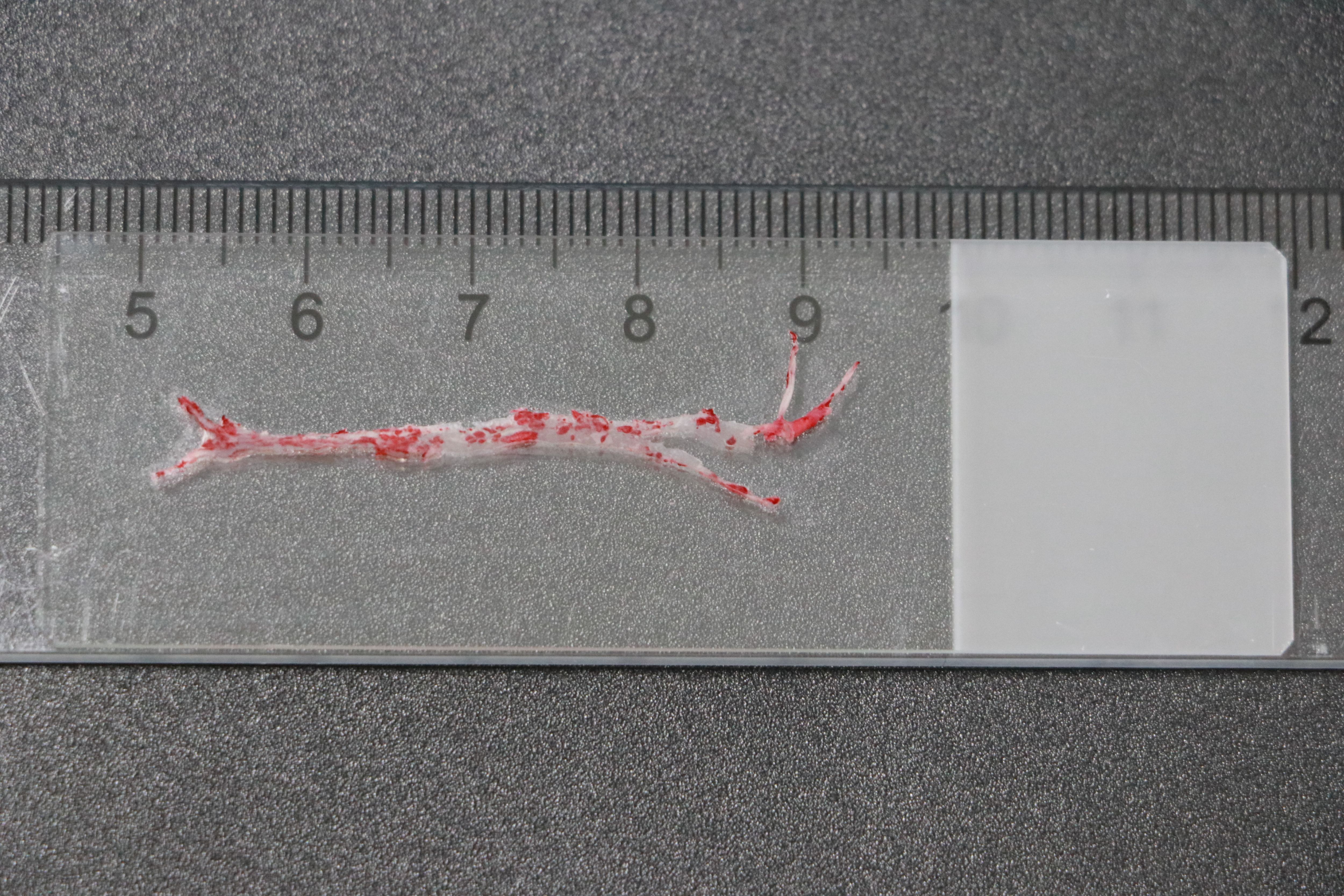

Supplement: S4 File — (ZIP) [file pone.0347758.s004.zip › Oil red O staining of aorta/PSB-L/74.JPG]

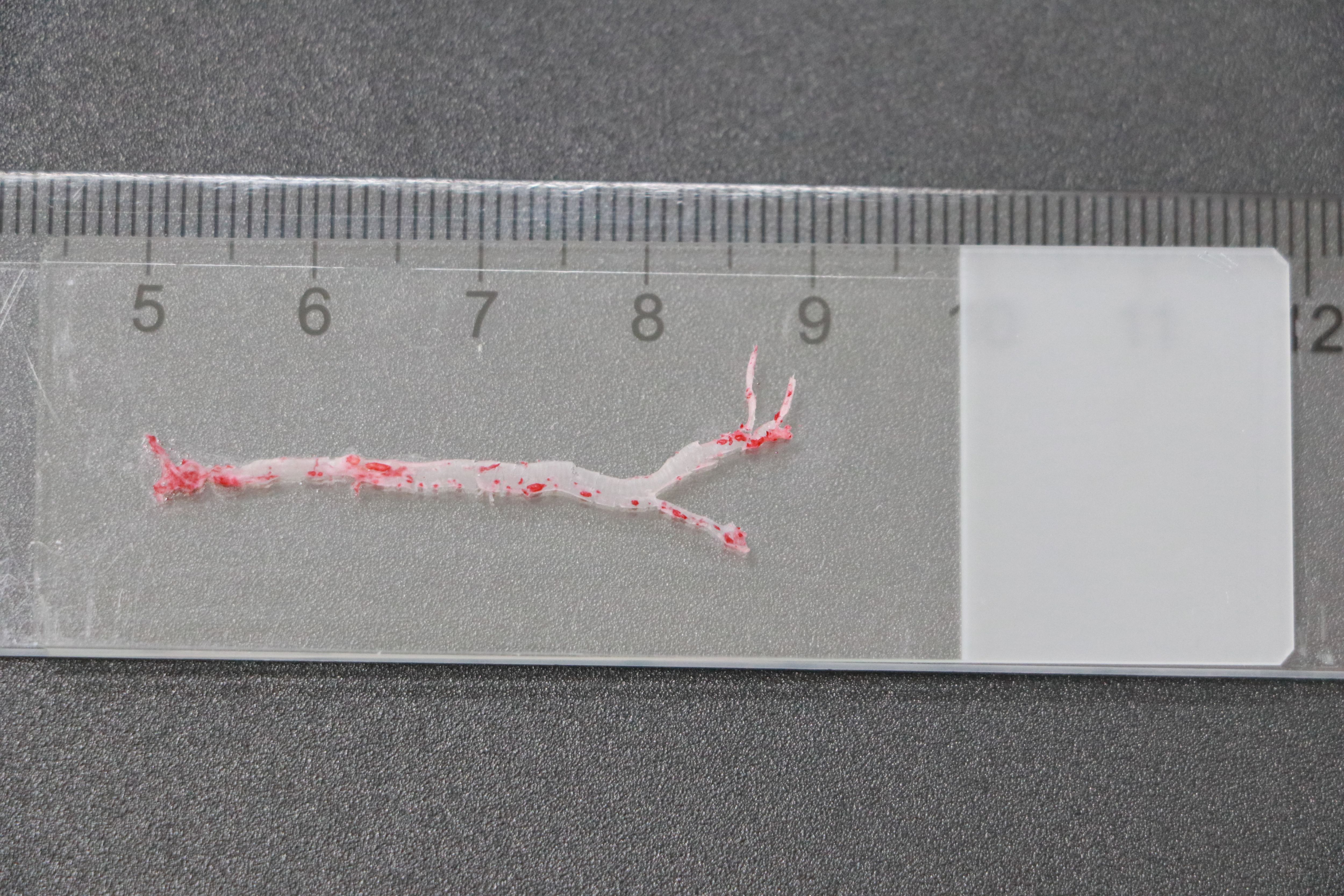

Supplement: S4 File — (ZIP) [file pone.0347758.s004.zip › Oil red O staining of aorta/PSB-L/77.JPG]

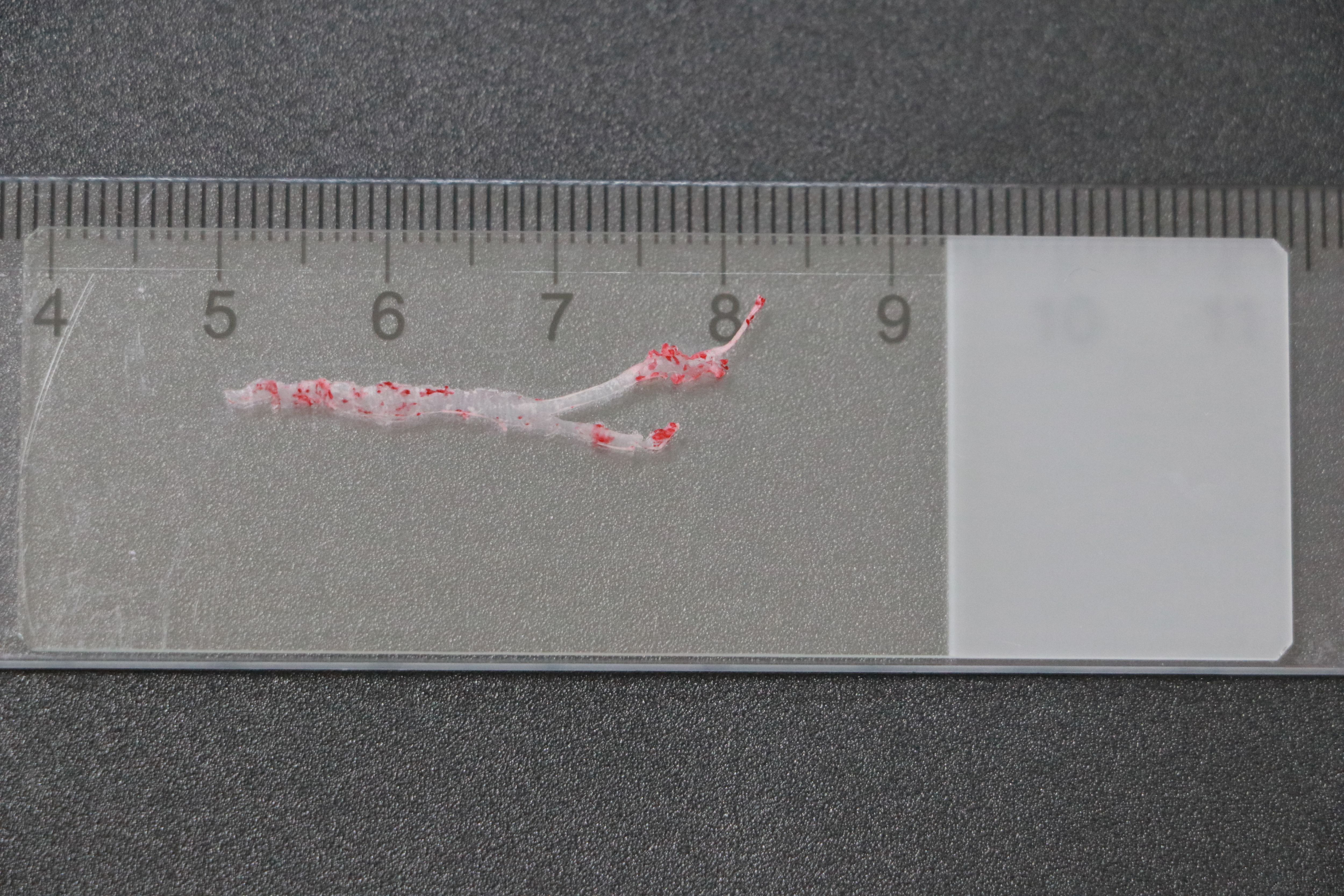

Supplement: S4 File — (ZIP) [file pone.0347758.s004.zip › Oil red O staining of aorta/PSB-L/80.JPG]

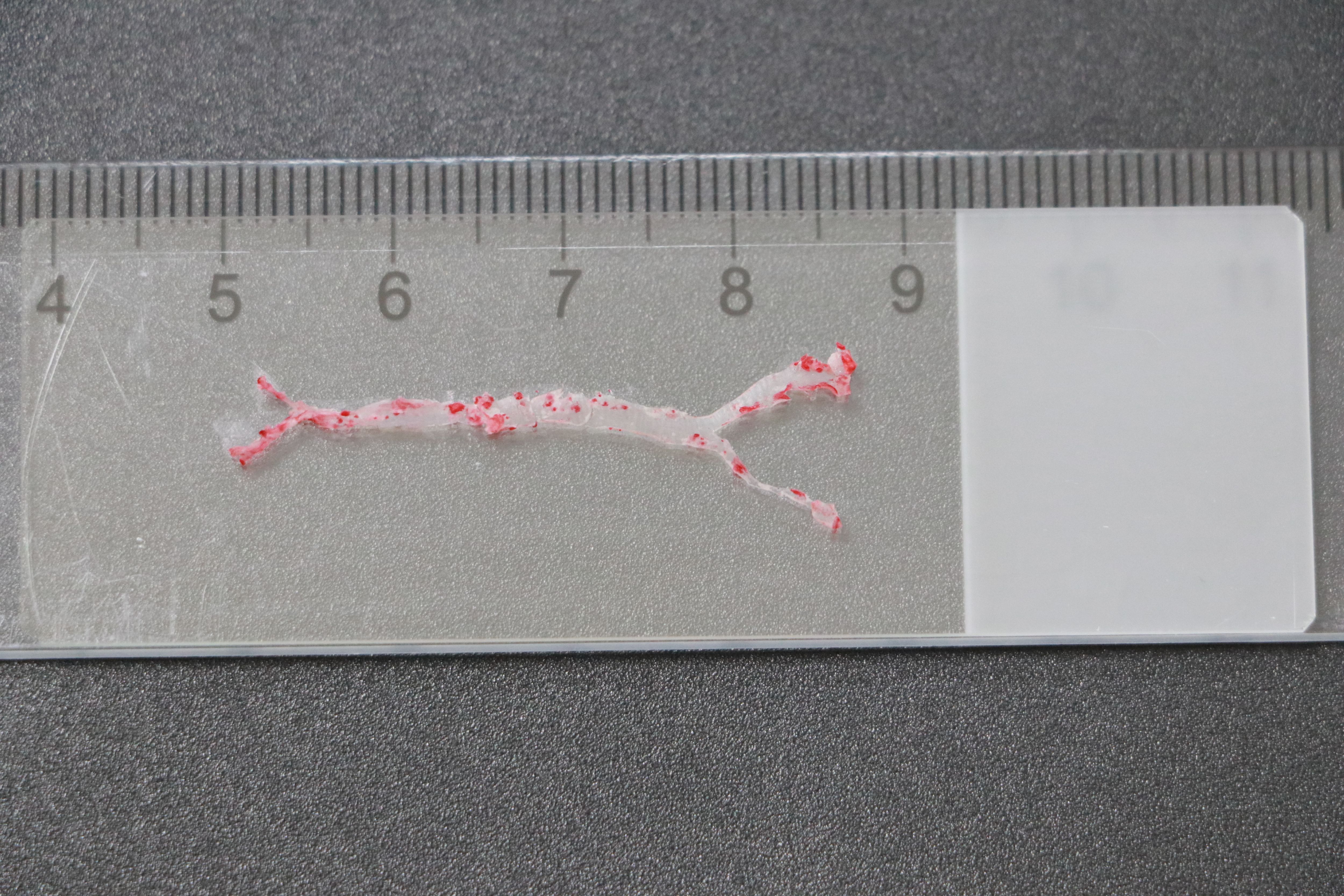

Supplement: S4 File — (ZIP) [file pone.0347758.s004.zip › Oil red O staining of aorta/PSB-L/82.JPG]

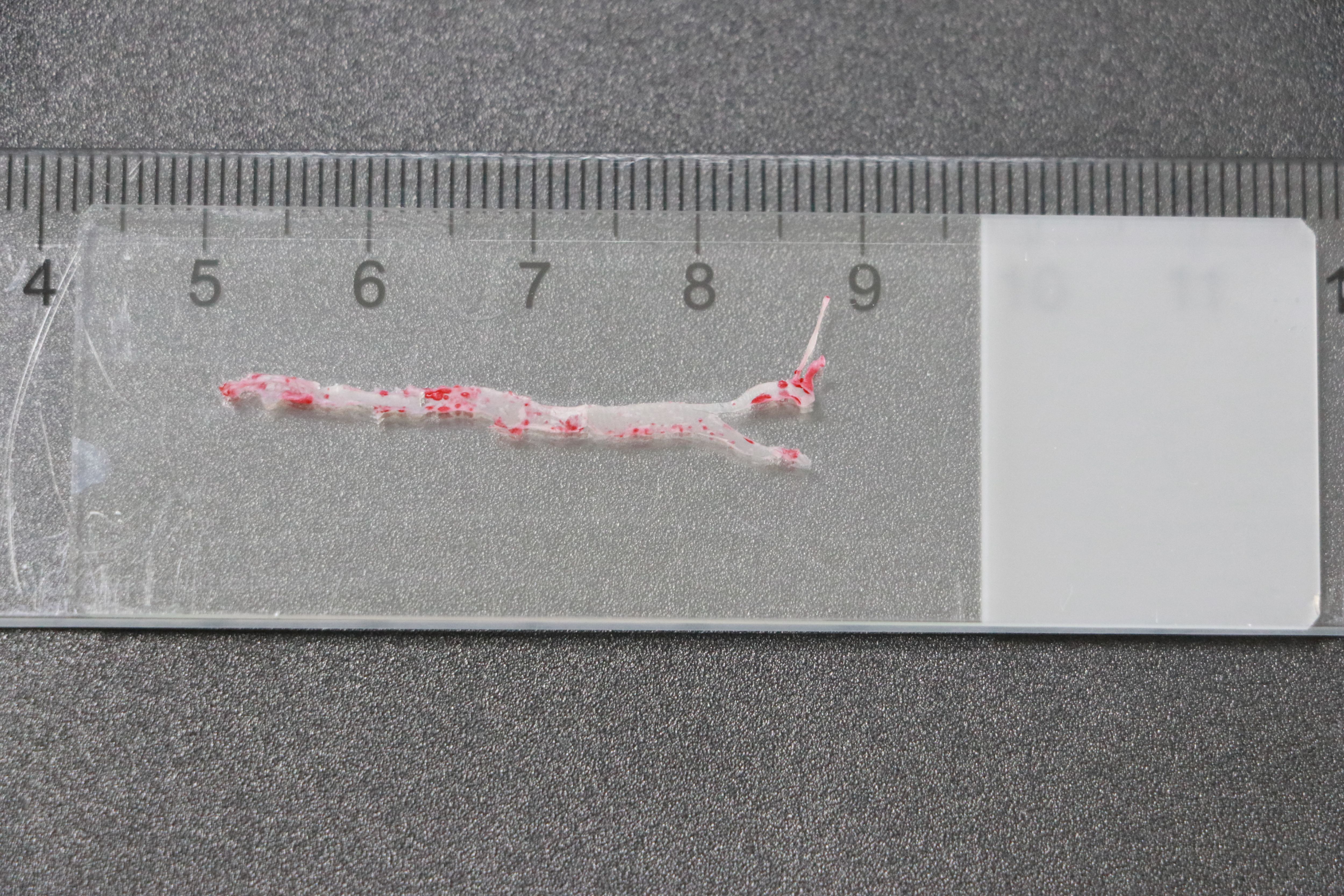

Supplement: S4 File — (ZIP) [file pone.0347758.s004.zip › Oil red O staining of aorta/PSB-M/85.JPG]

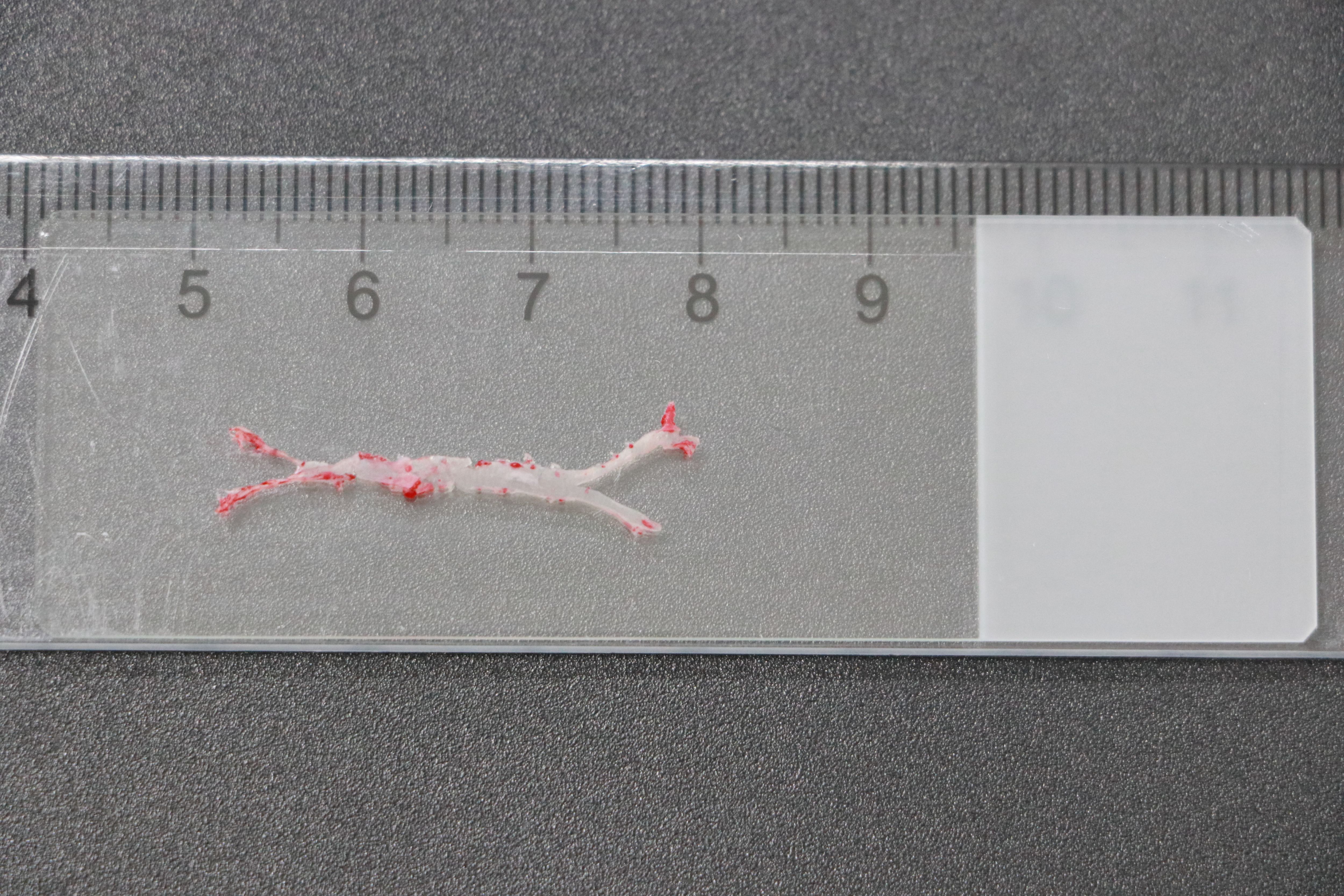

Supplement: S4 File — (ZIP) [file pone.0347758.s004.zip › Oil red O staining of aorta/PSB-M/89.JPG]

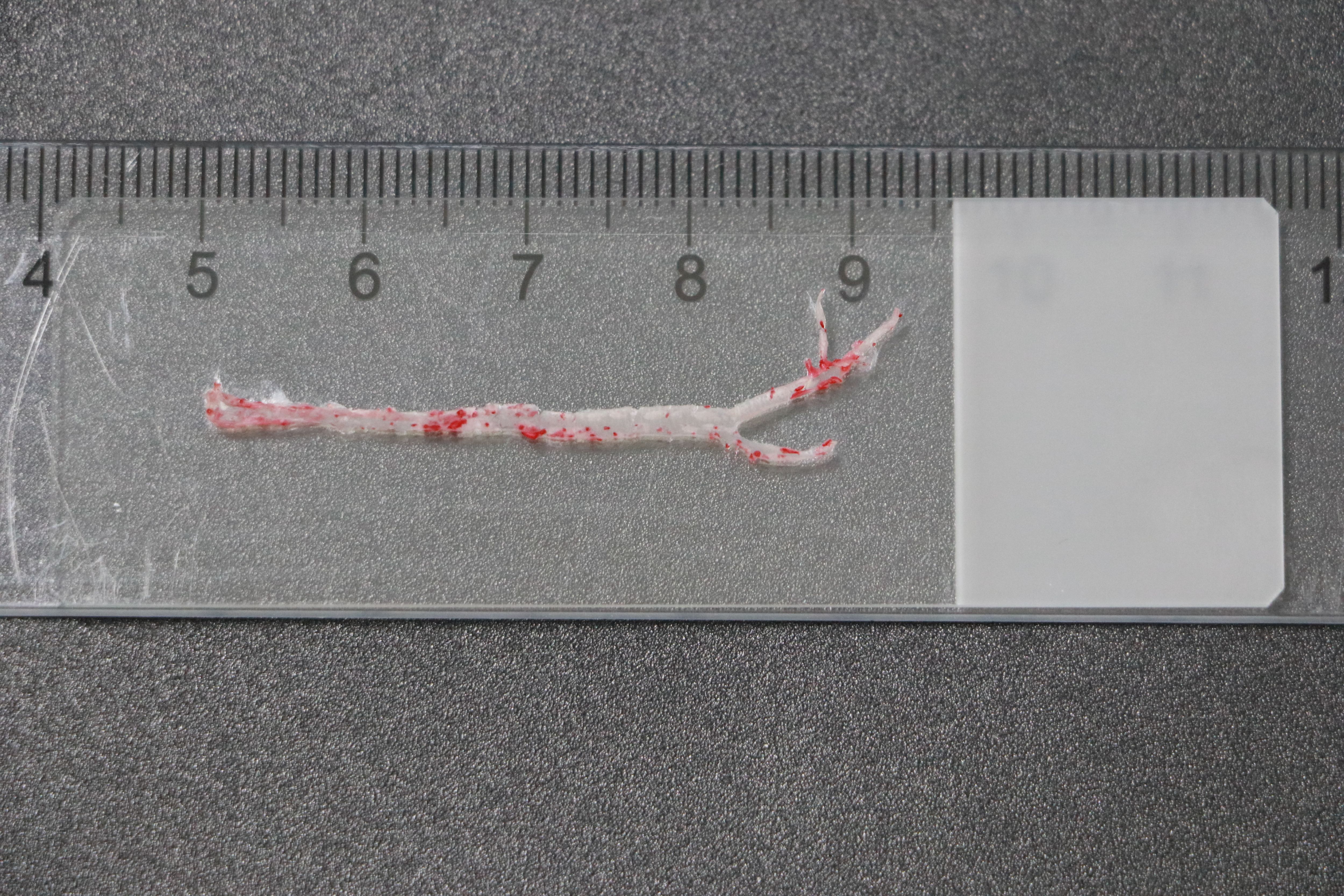

Supplement: S4 File — (ZIP) [file pone.0347758.s004.zip › Oil red O staining of aorta/PSB-M/90.JPG]

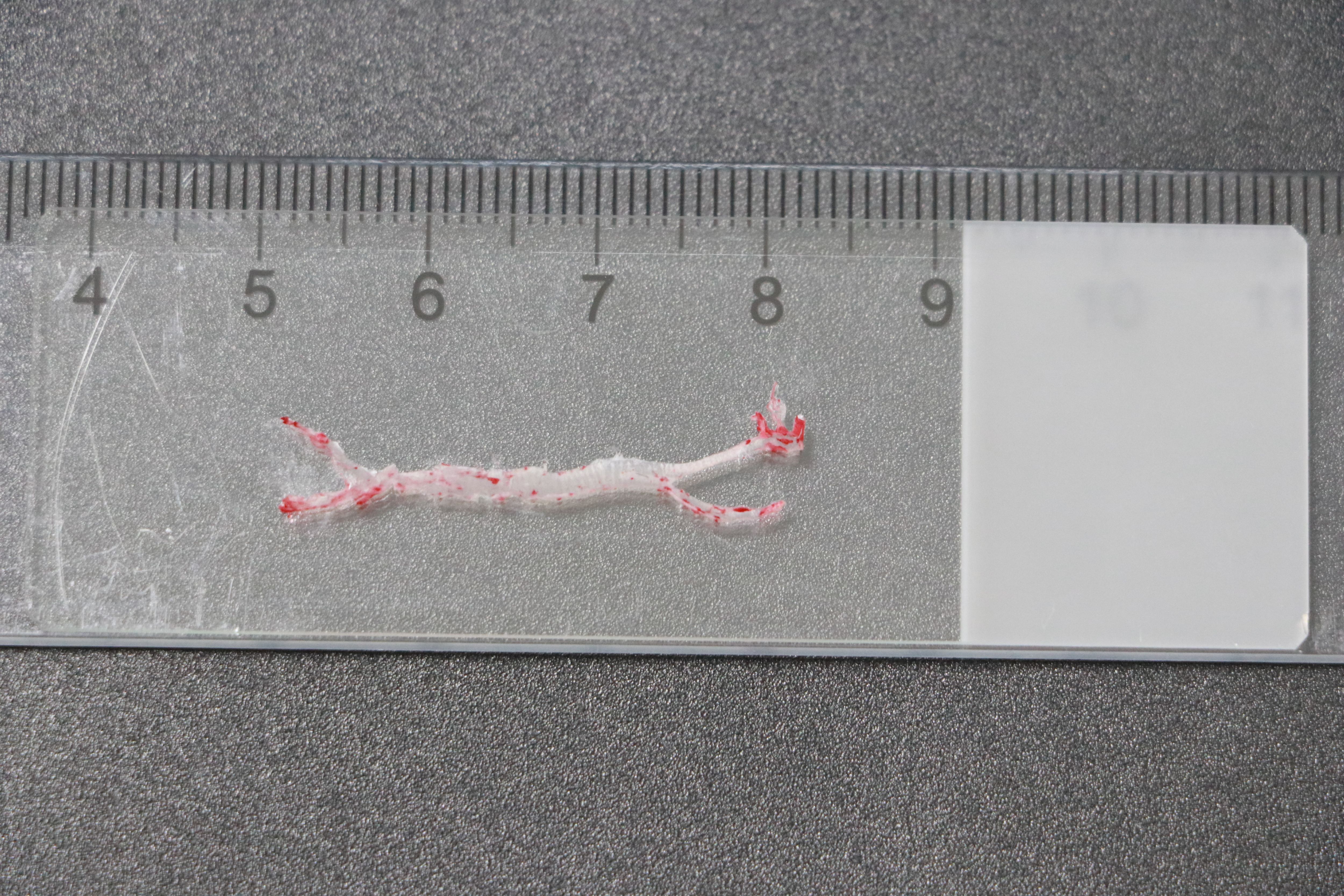

Supplement: S4 File — (ZIP) [file pone.0347758.s004.zip › Oil red O staining of aorta/statin/37.JPG]

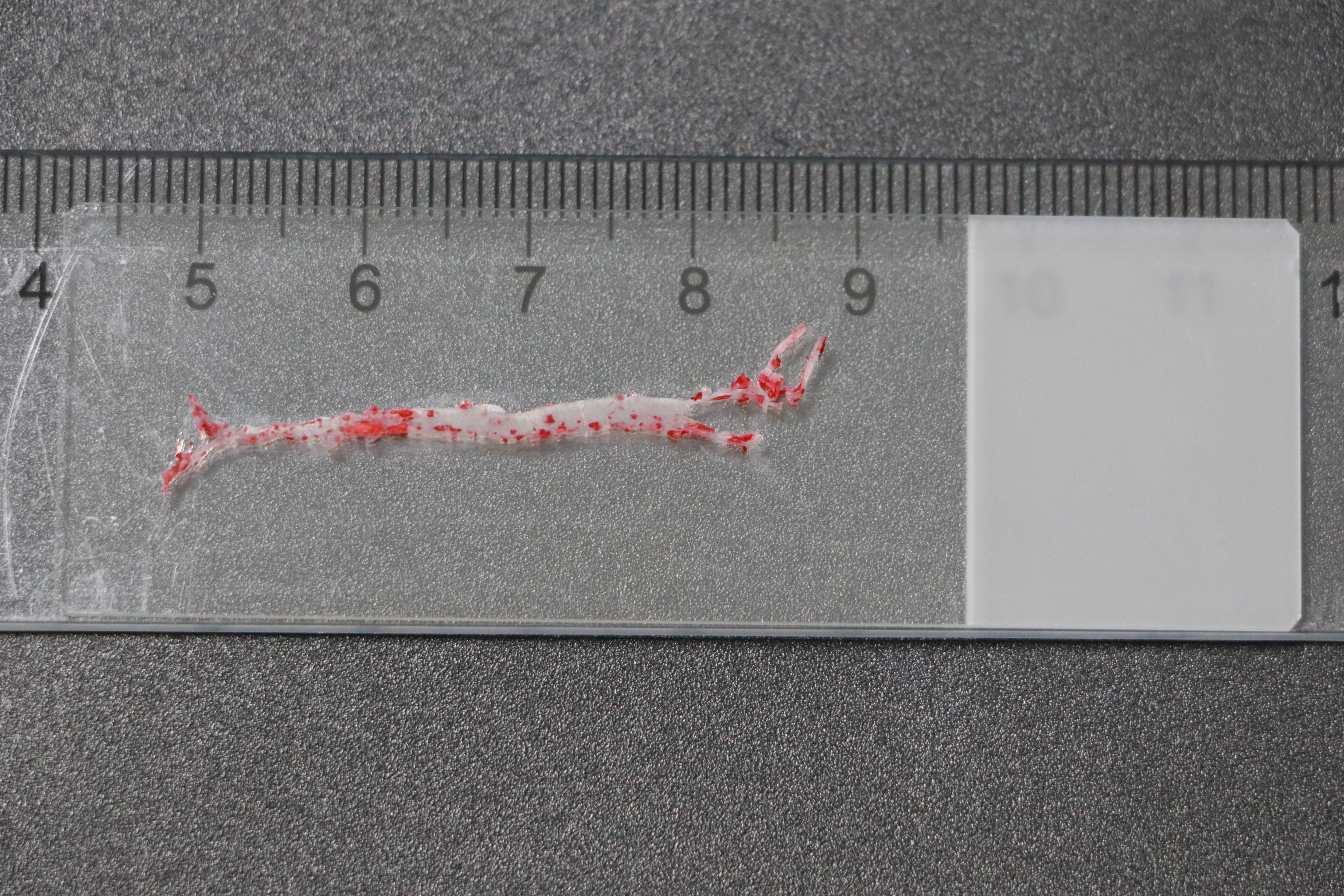

Supplement: S4 File — (ZIP) [file pone.0347758.s004.zip › Oil red O staining of aorta/statin/38.JPG]

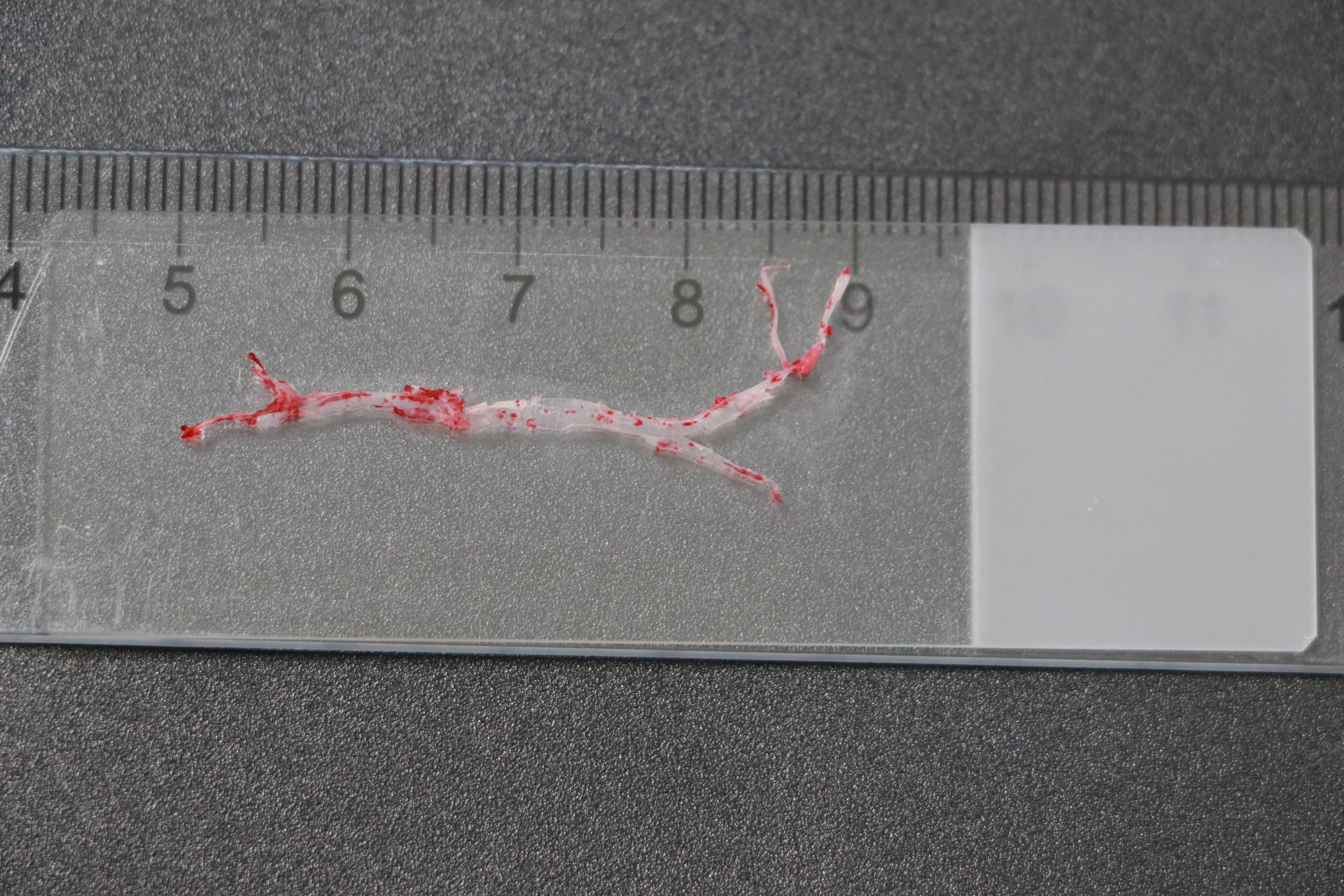

Supplement: S4 File — (ZIP) [file pone.0347758.s004.zip › Oil red O staining of aorta/statin/40.JPG]

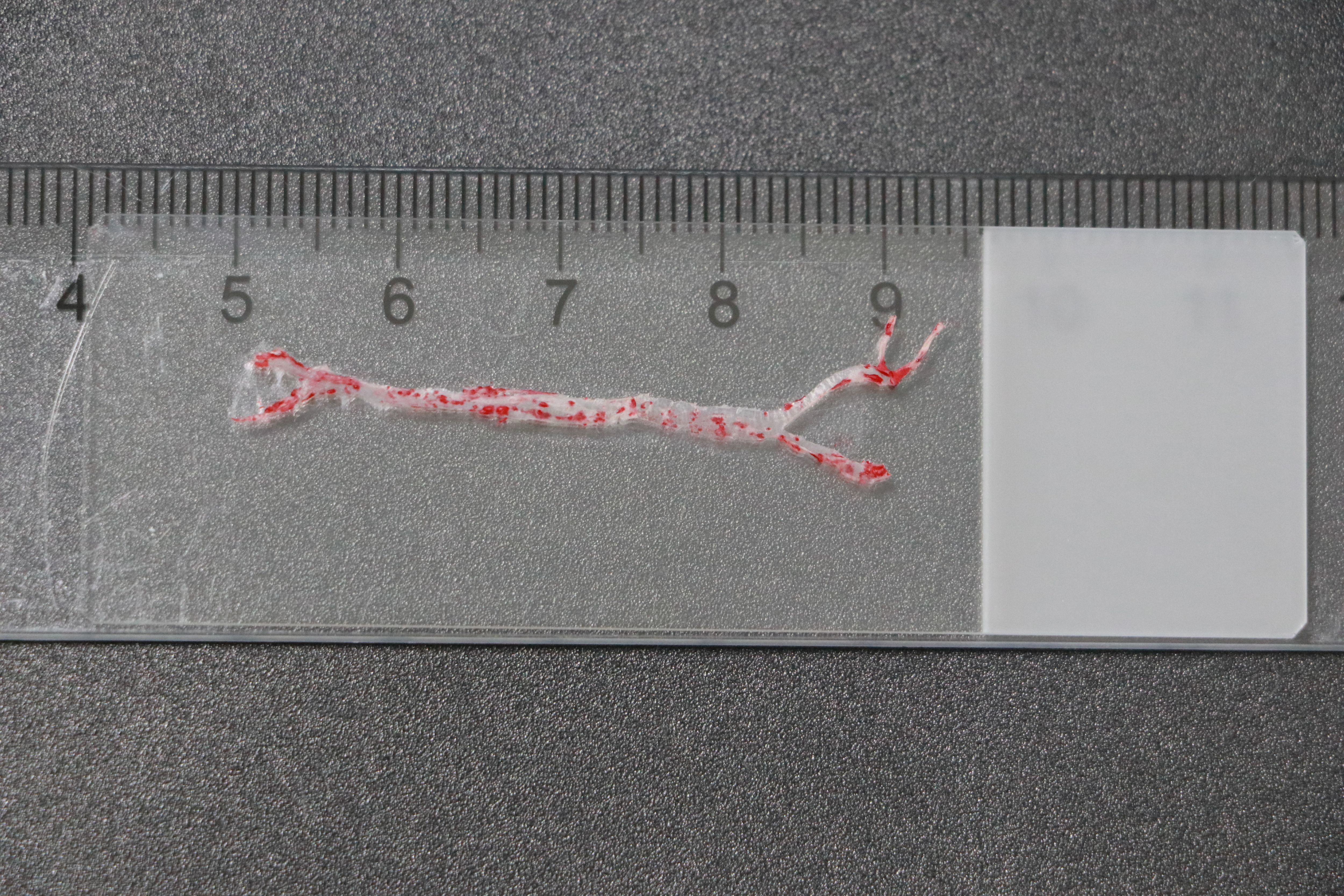

Supplement: S4 File — (ZIP) [file pone.0347758.s004.zip › Oil red O staining of aorta/statin/41.JPG]

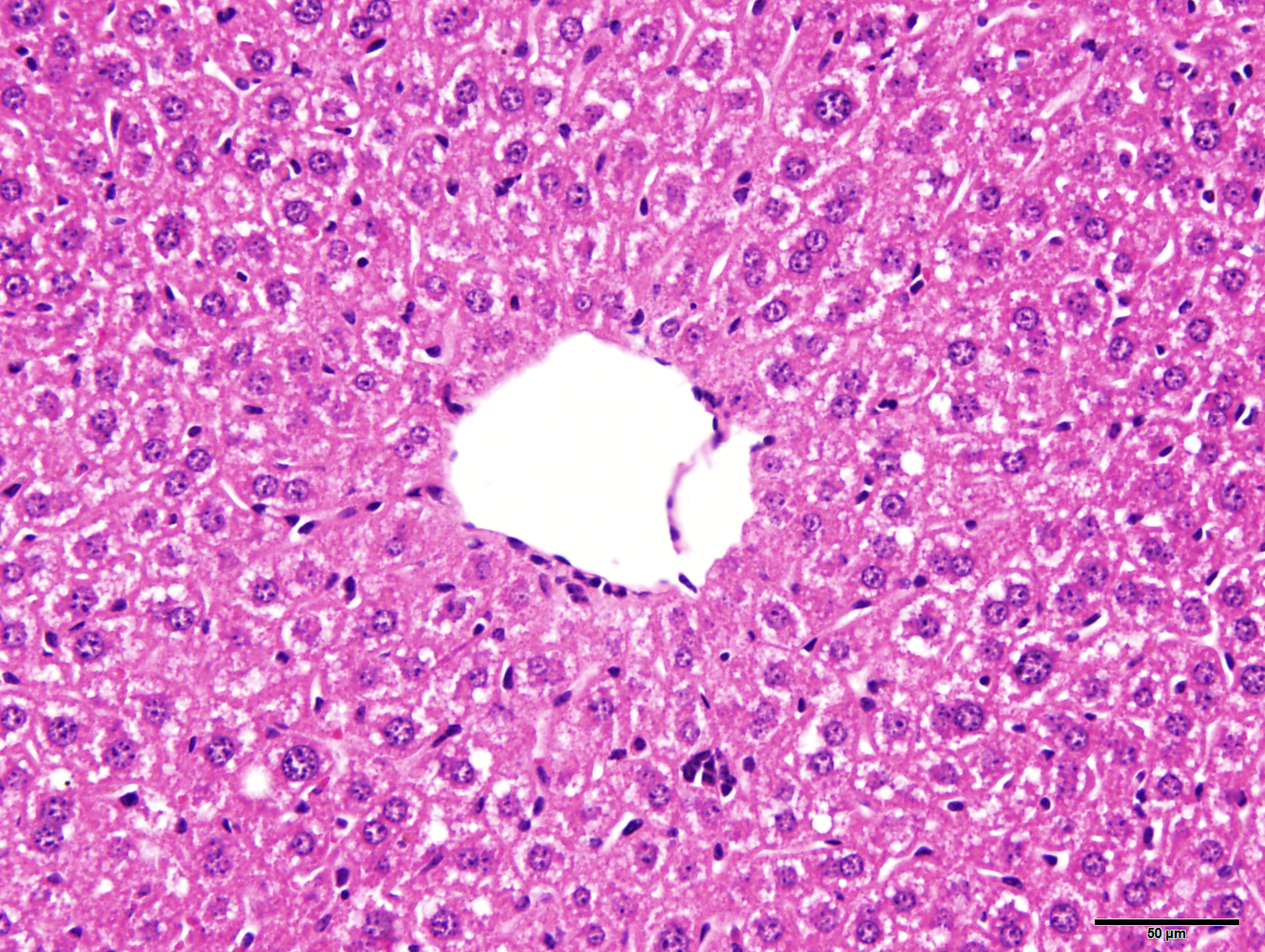

Supplement: S5 File — (ZIP) [file pone.0347758.s005.zip › Liver H&E staining/AS/23.tif]

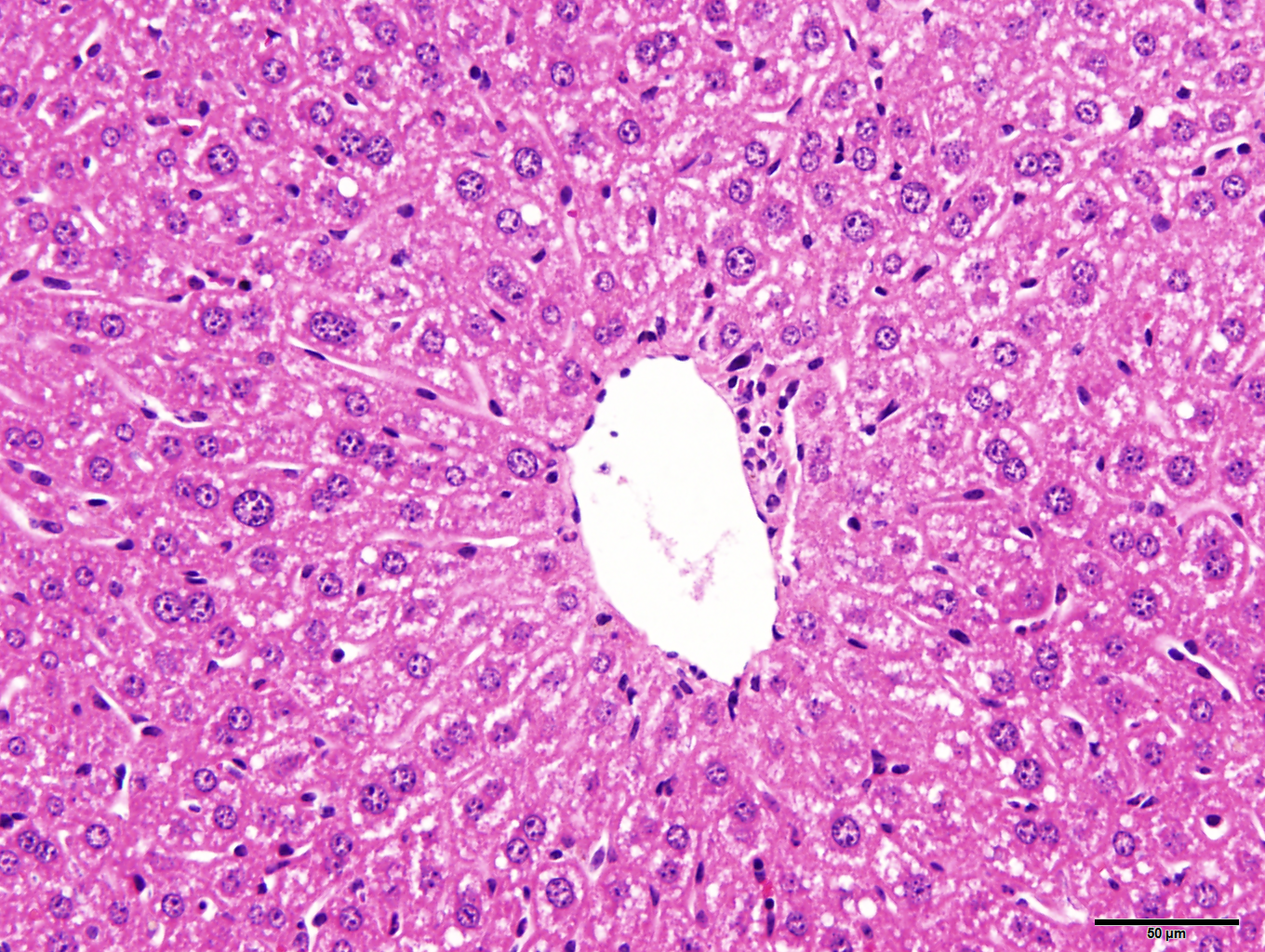

Supplement: S5 File — (ZIP) [file pone.0347758.s005.zip › Liver H&E staining/AS/25.tif]

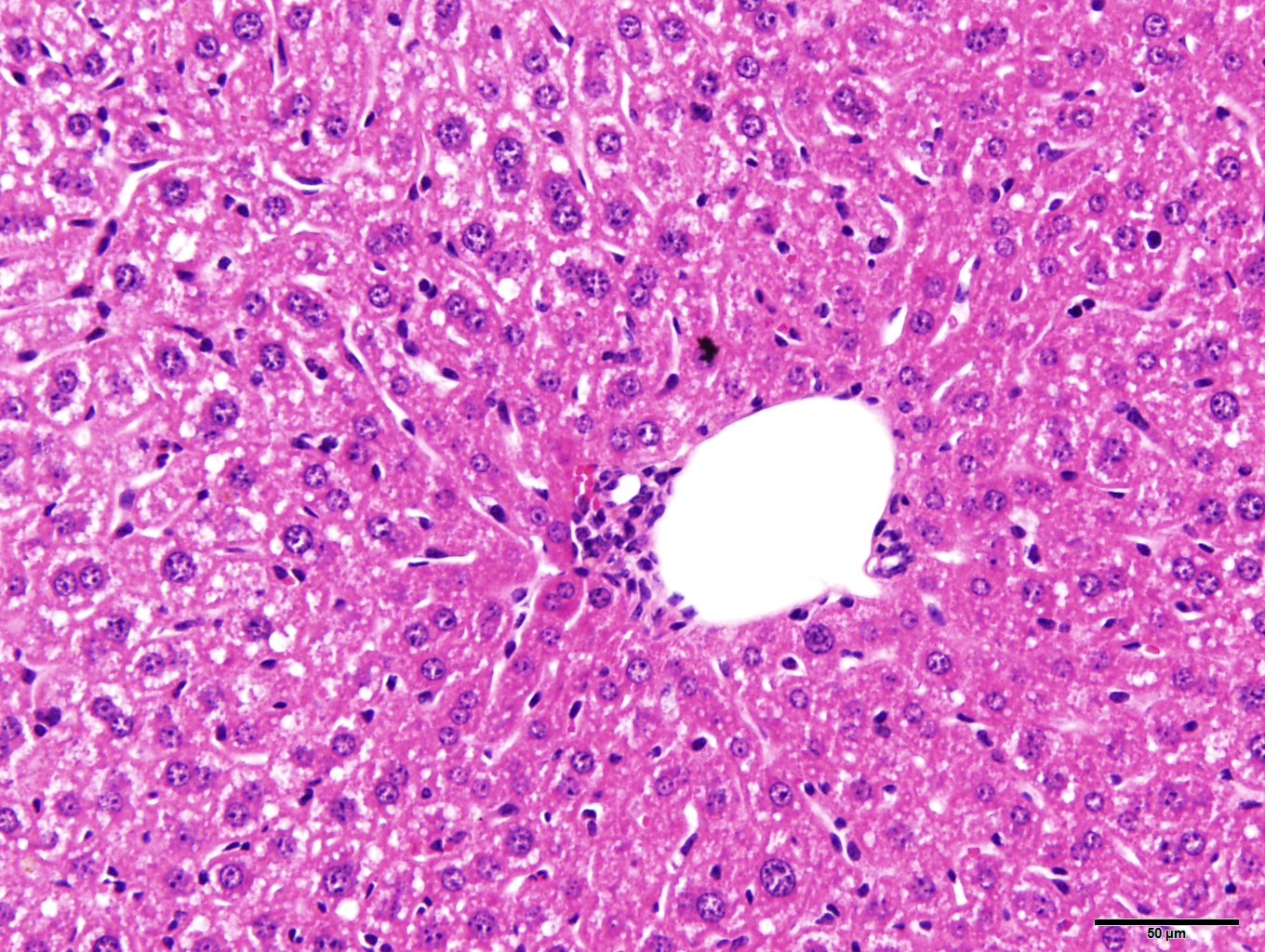

Supplement: S5 File — (ZIP) [file pone.0347758.s005.zip › Liver H&E staining/AS/26.tif]

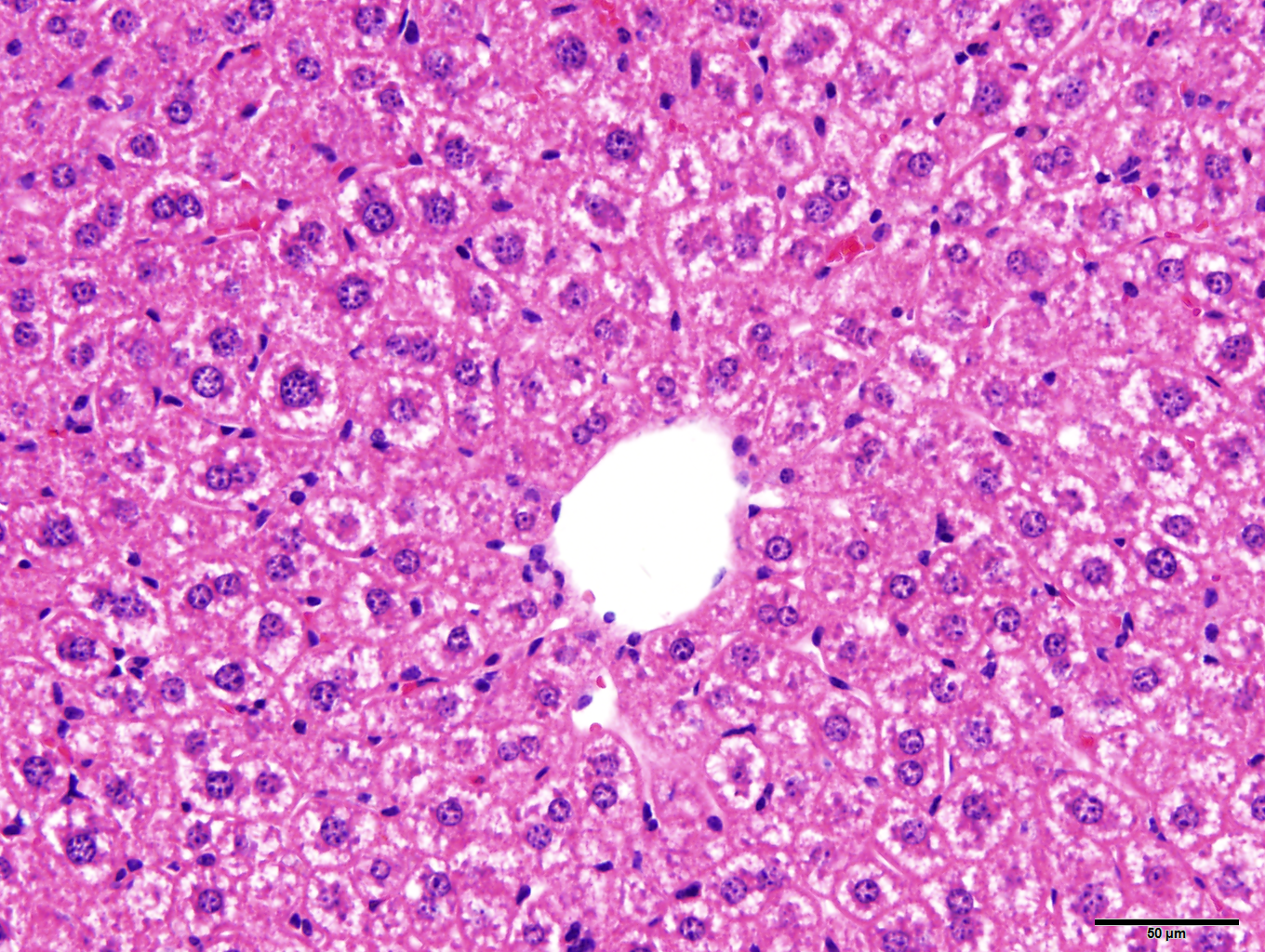

Supplement: S5 File — (ZIP) [file pone.0347758.s005.zip › Liver H&E staining/AS/31.tif]

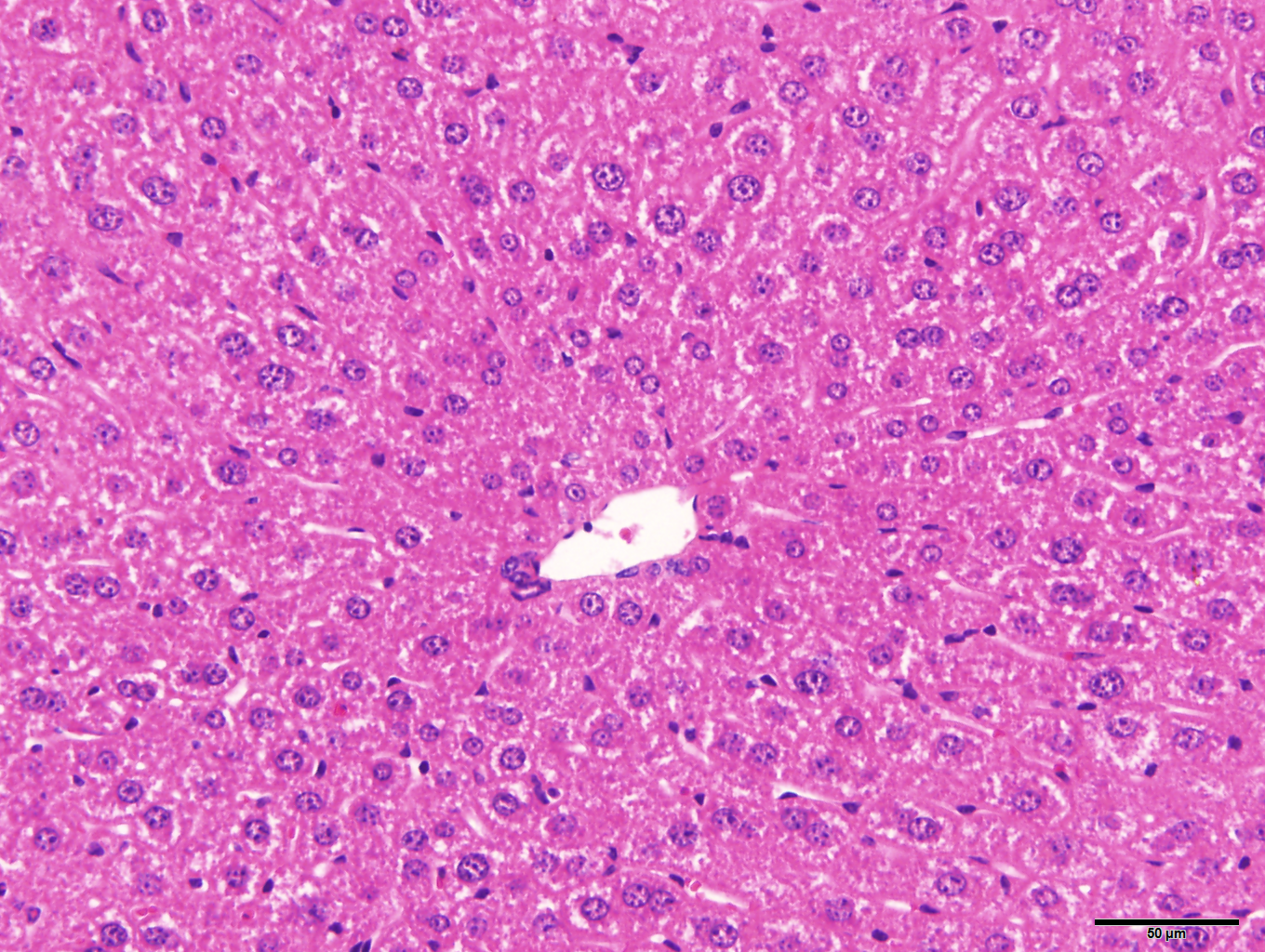

Supplement: S5 File — (ZIP) [file pone.0347758.s005.zip › Liver H&E staining/control/1.tif]

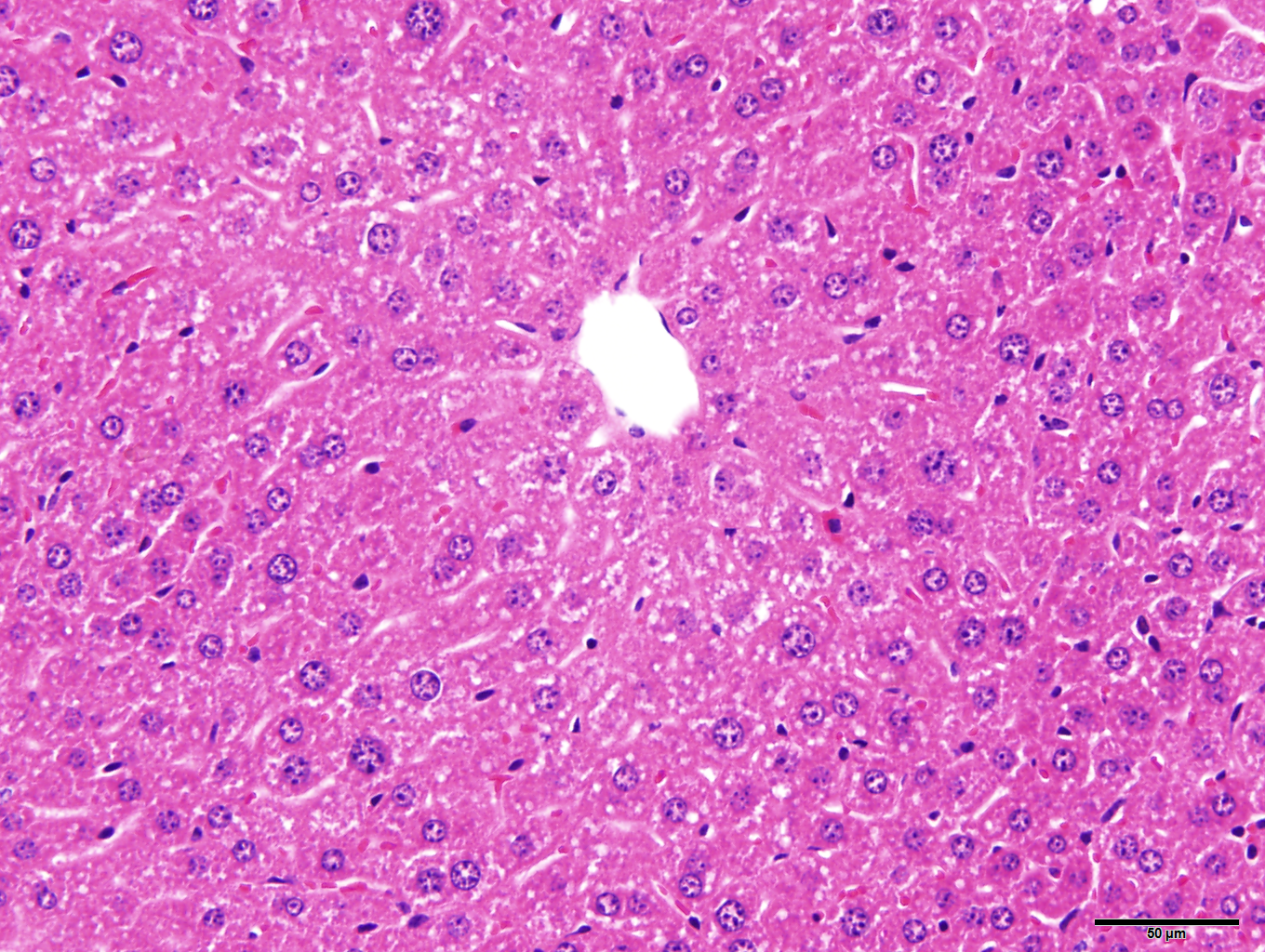

Supplement: S5 File — (ZIP) [file pone.0347758.s005.zip › Liver H&E staining/control/10.tif]

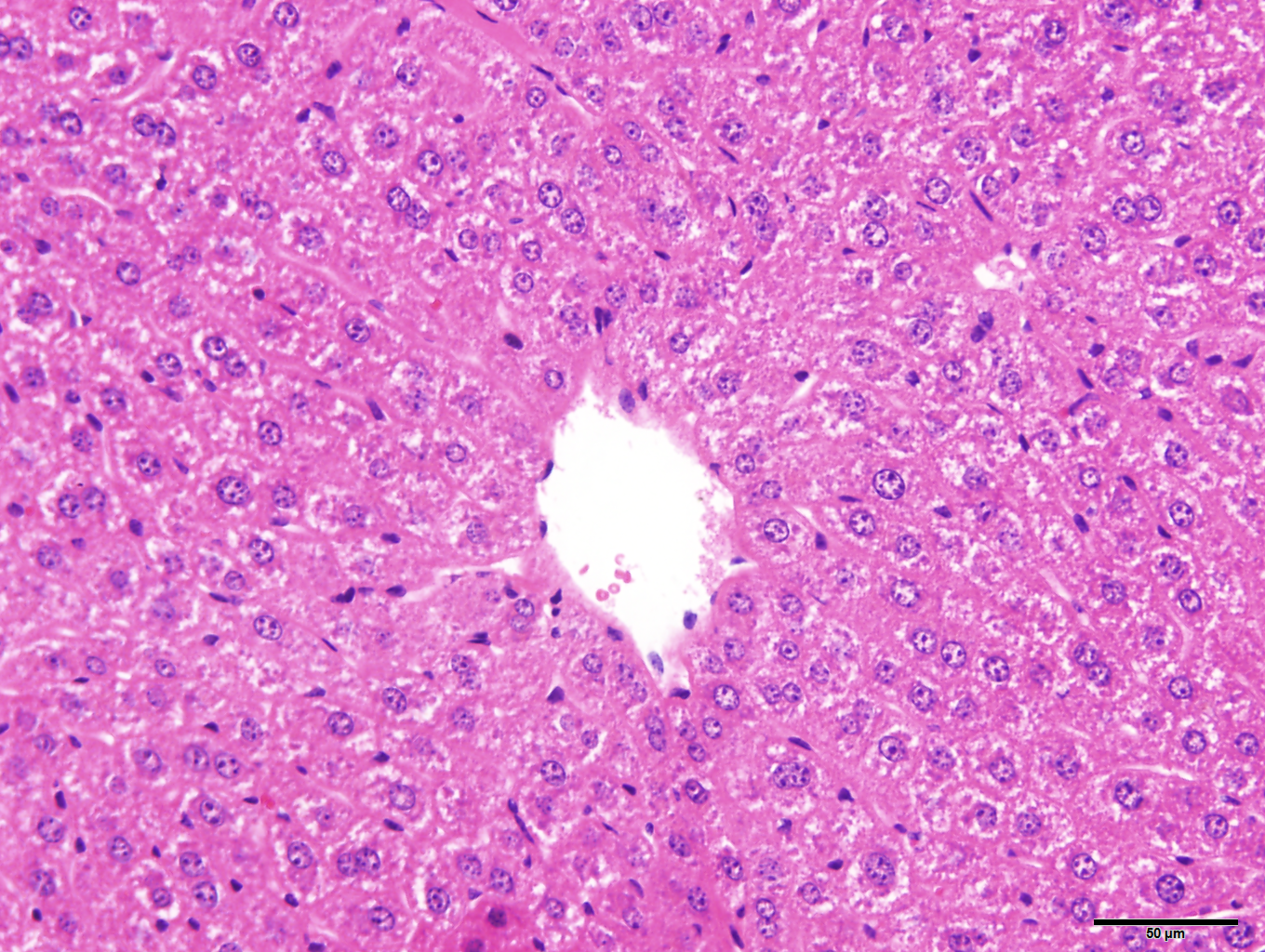

Supplement: S5 File — (ZIP) [file pone.0347758.s005.zip › Liver H&E staining/control/2.tif]

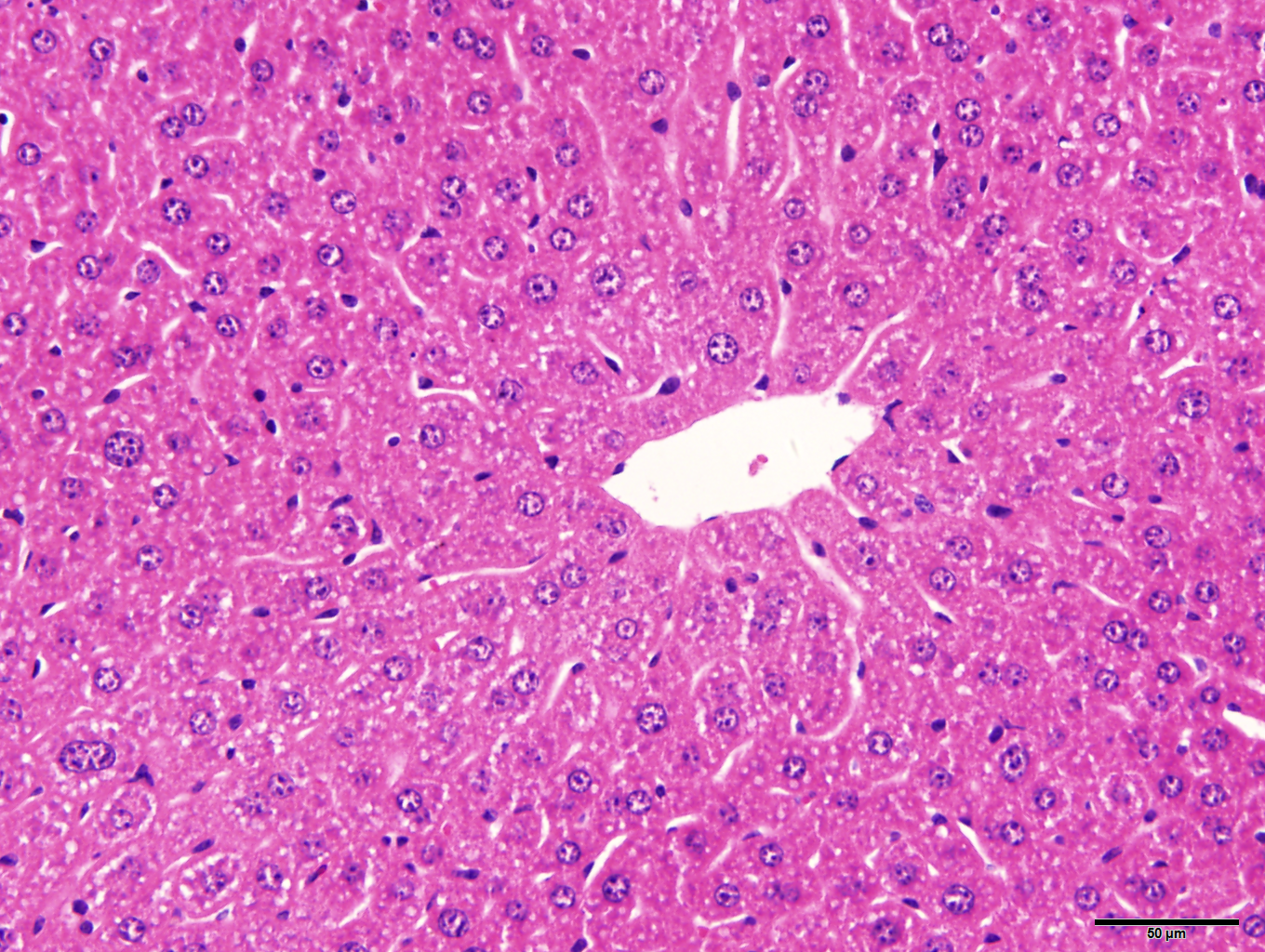

Supplement: S5 File — (ZIP) [file pone.0347758.s005.zip › Liver H&E staining/control/3.tif]
