# Supplementary figures and images for: Polyphenol extract of Syzygium brachythyrsum mitigates atherosclerosis in high-fat diet induced ApoE-/- mice by regulating ROS/Keap1/Nrf2 pathway (part 2 of 4)
Source: PLoS One. 2026 May 5;21(5):e0347758. doi: 10.1371/journal.pone.0347758 (PMC13143111; doi:10.1371/journal.pone.0347758)

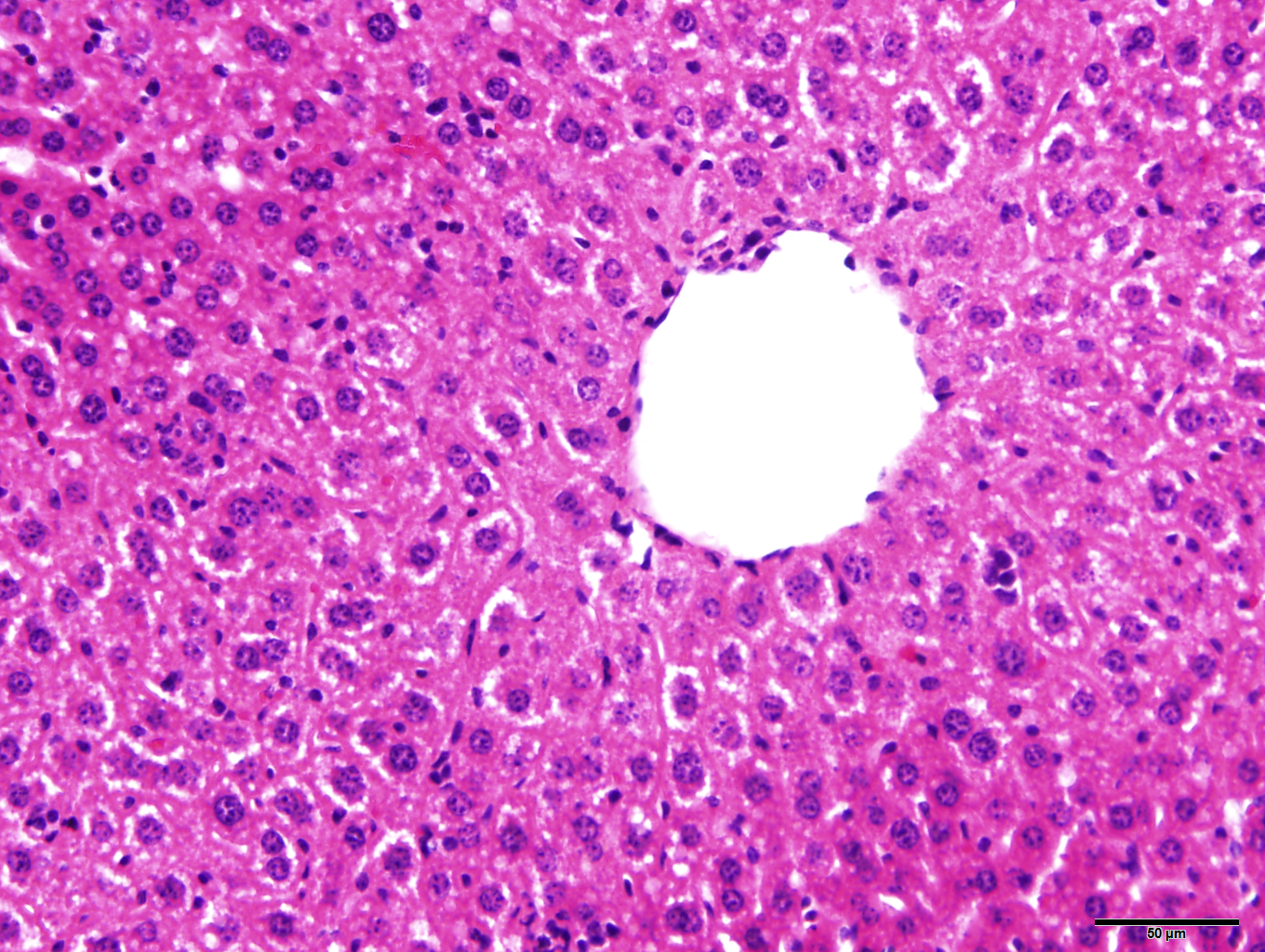

Supplement: S5 File — (ZIP) [file pone.0347758.s005.zip › Liver H&E staining/PSB-H/100.tif]

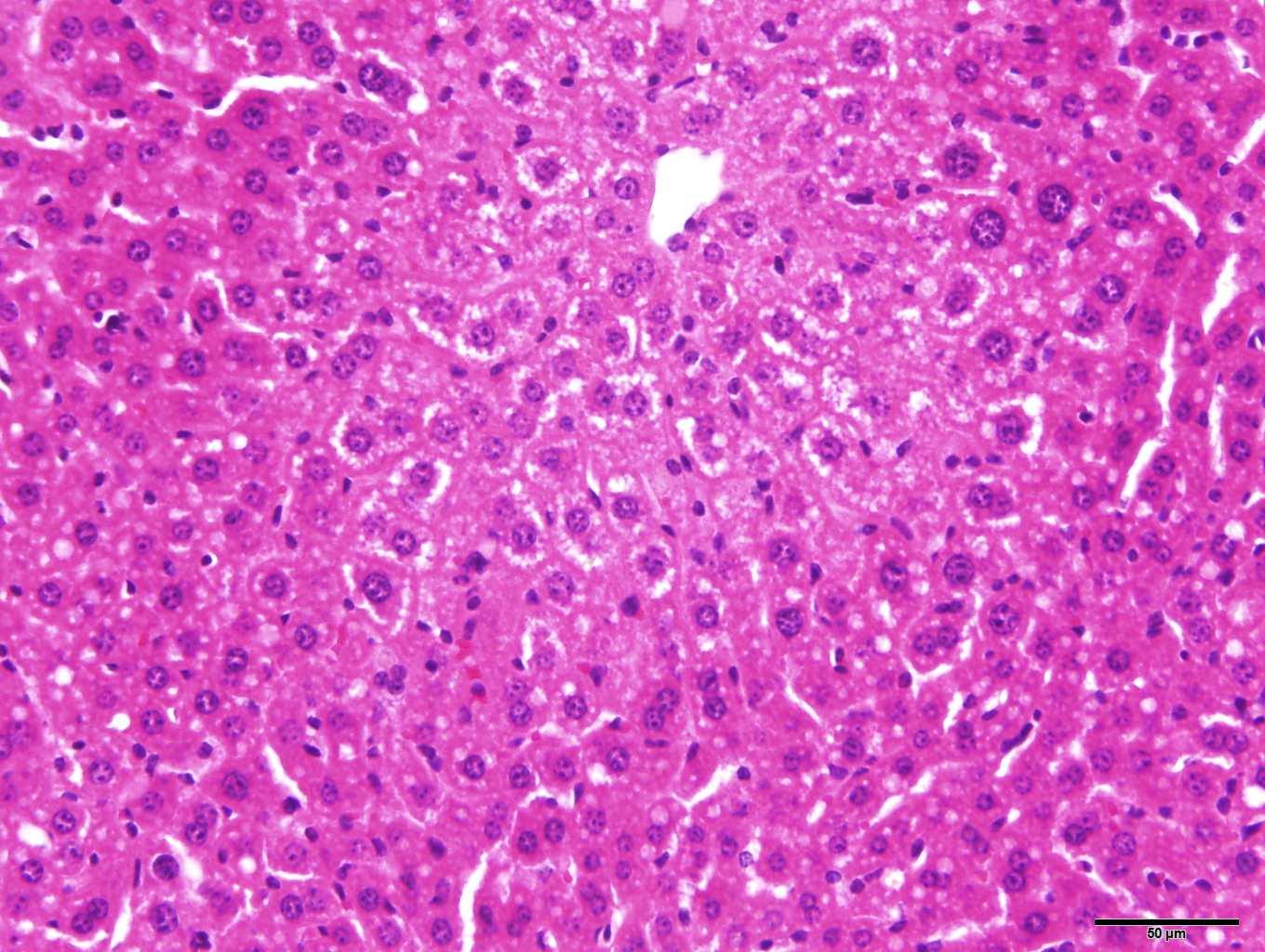

Supplement: S5 File — (ZIP) [file pone.0347758.s005.zip › Liver H&E staining/PSB-H/96.tif]

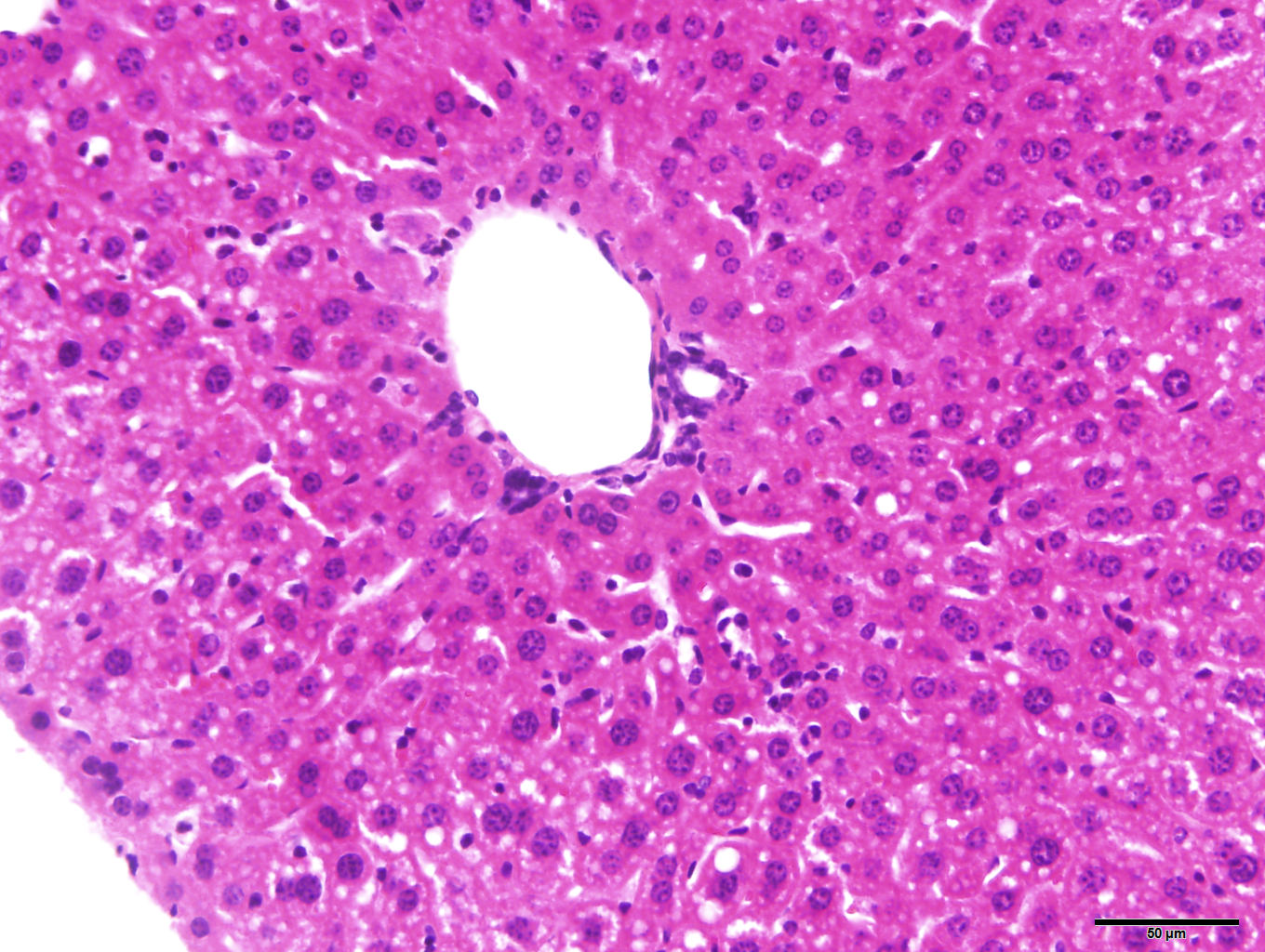

Supplement: S5 File — (ZIP) [file pone.0347758.s005.zip › Liver H&E staining/PSB-H/97.tif]

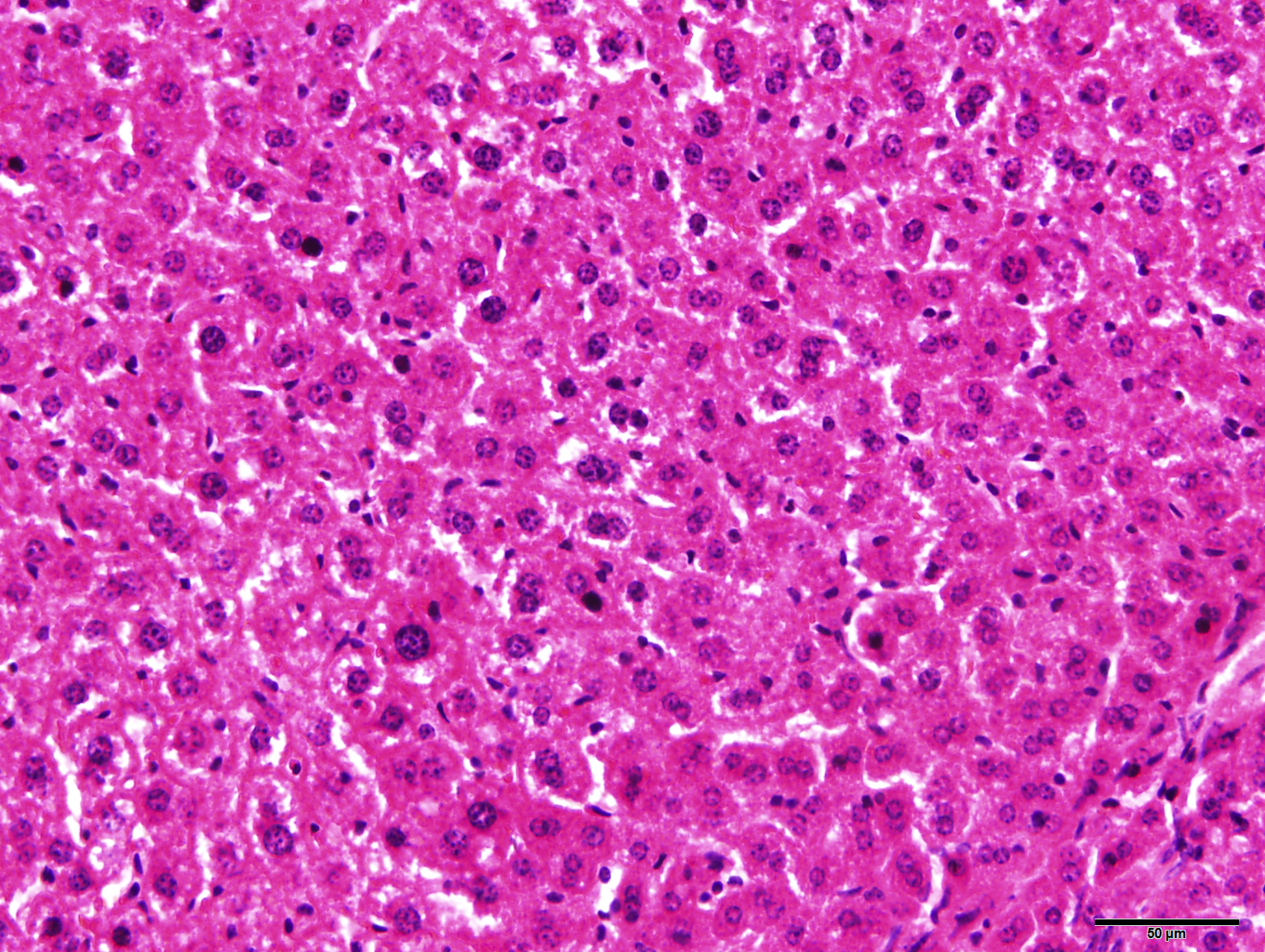

Supplement: S5 File — (ZIP) [file pone.0347758.s005.zip › Liver H&E staining/PSB-H/98.tif]

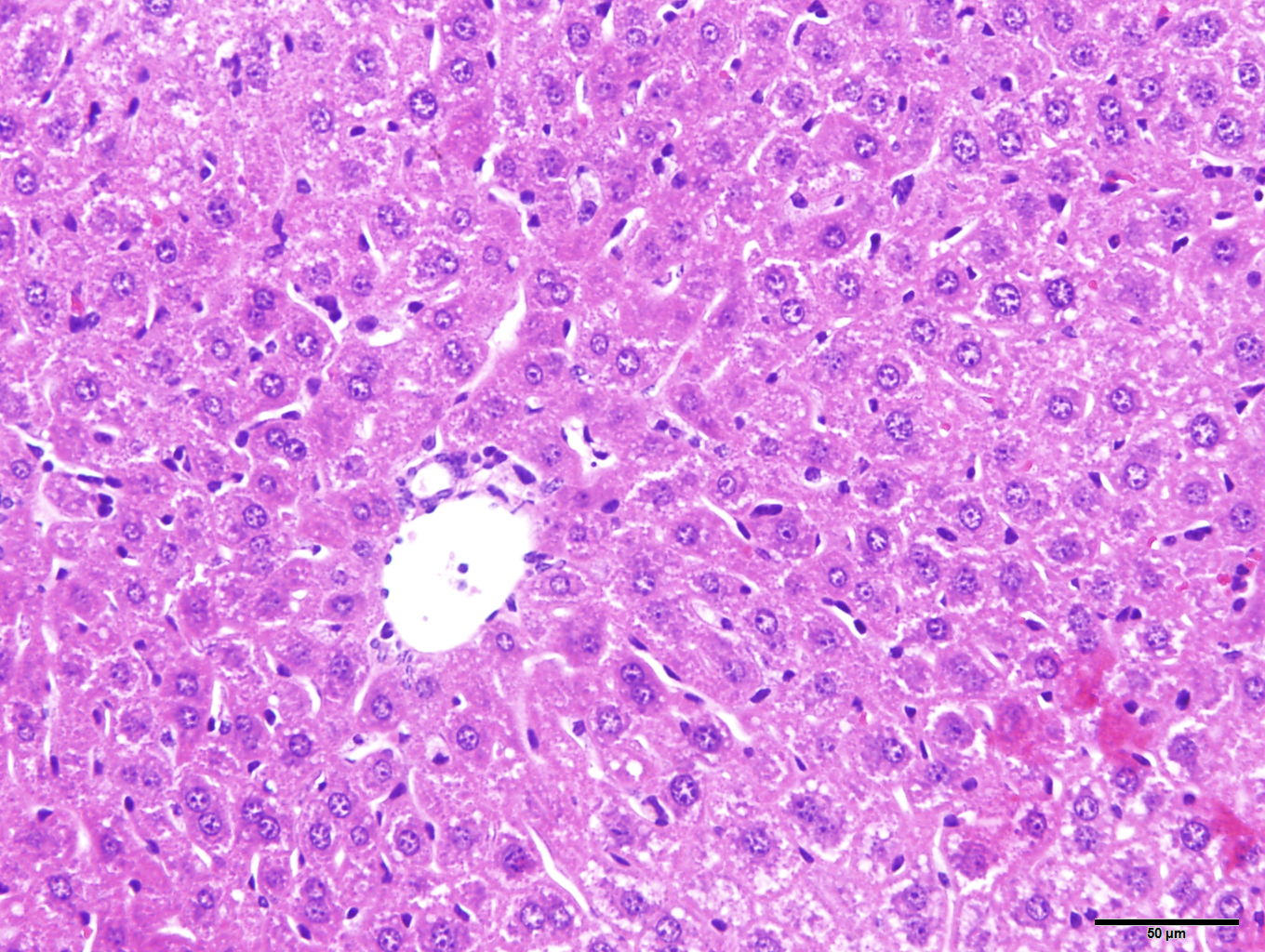

Supplement: S5 File — (ZIP) [file pone.0347758.s005.zip › Liver H&E staining/PSB-L/74.tif]

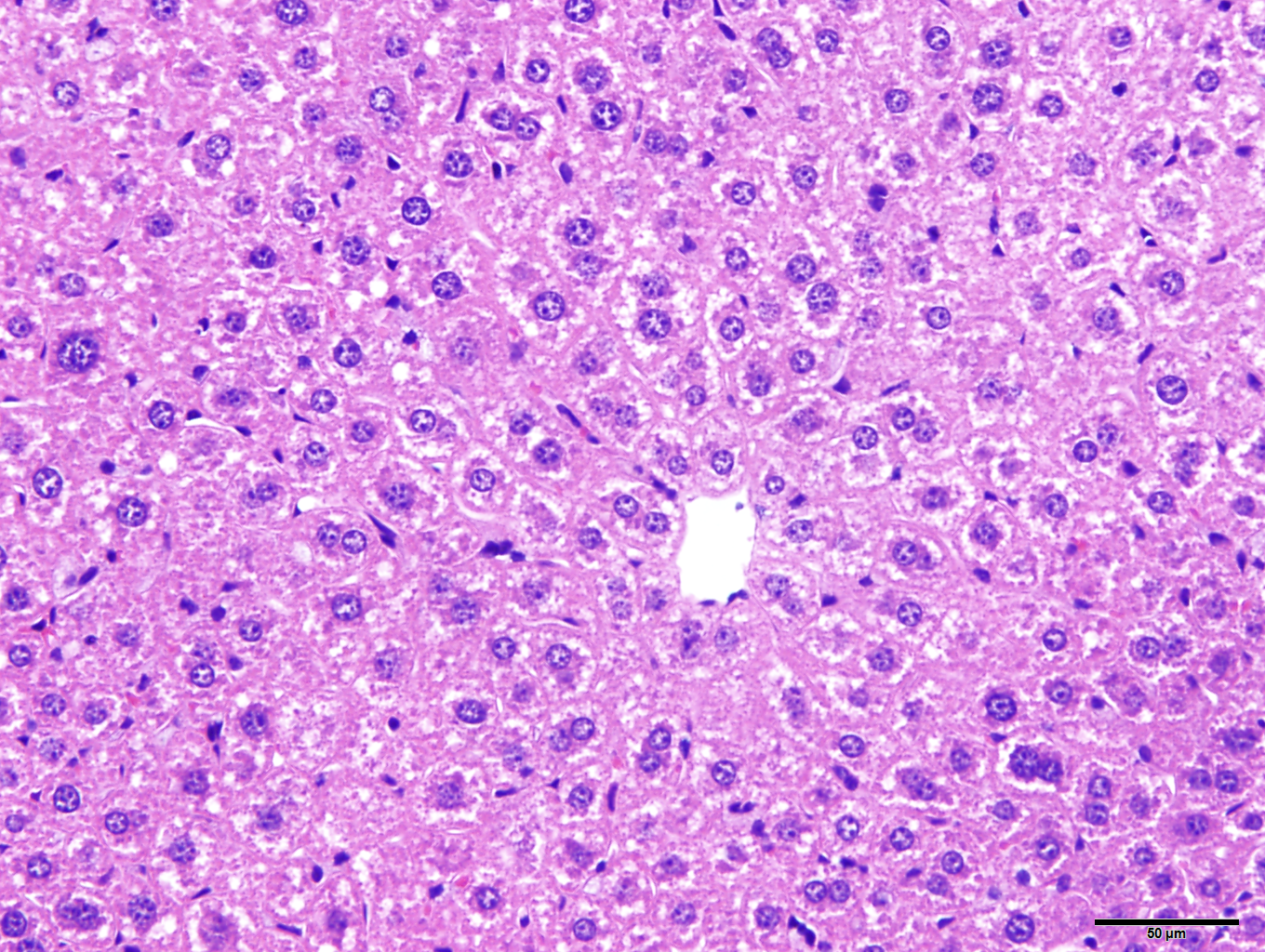

Supplement: S5 File — (ZIP) [file pone.0347758.s005.zip › Liver H&E staining/PSB-L/77.tif]

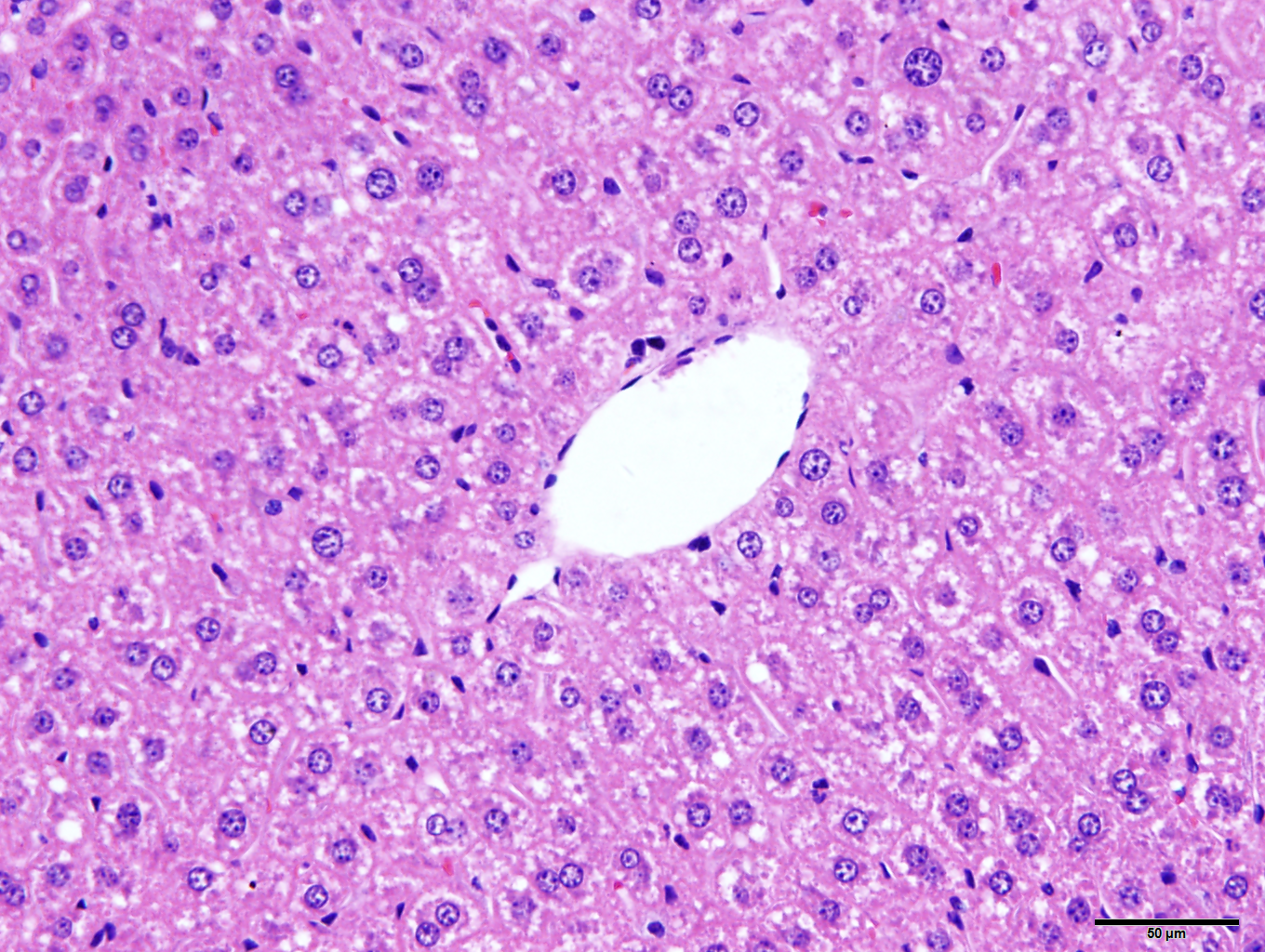

Supplement: S5 File — (ZIP) [file pone.0347758.s005.zip › Liver H&E staining/PSB-L/80.tif]

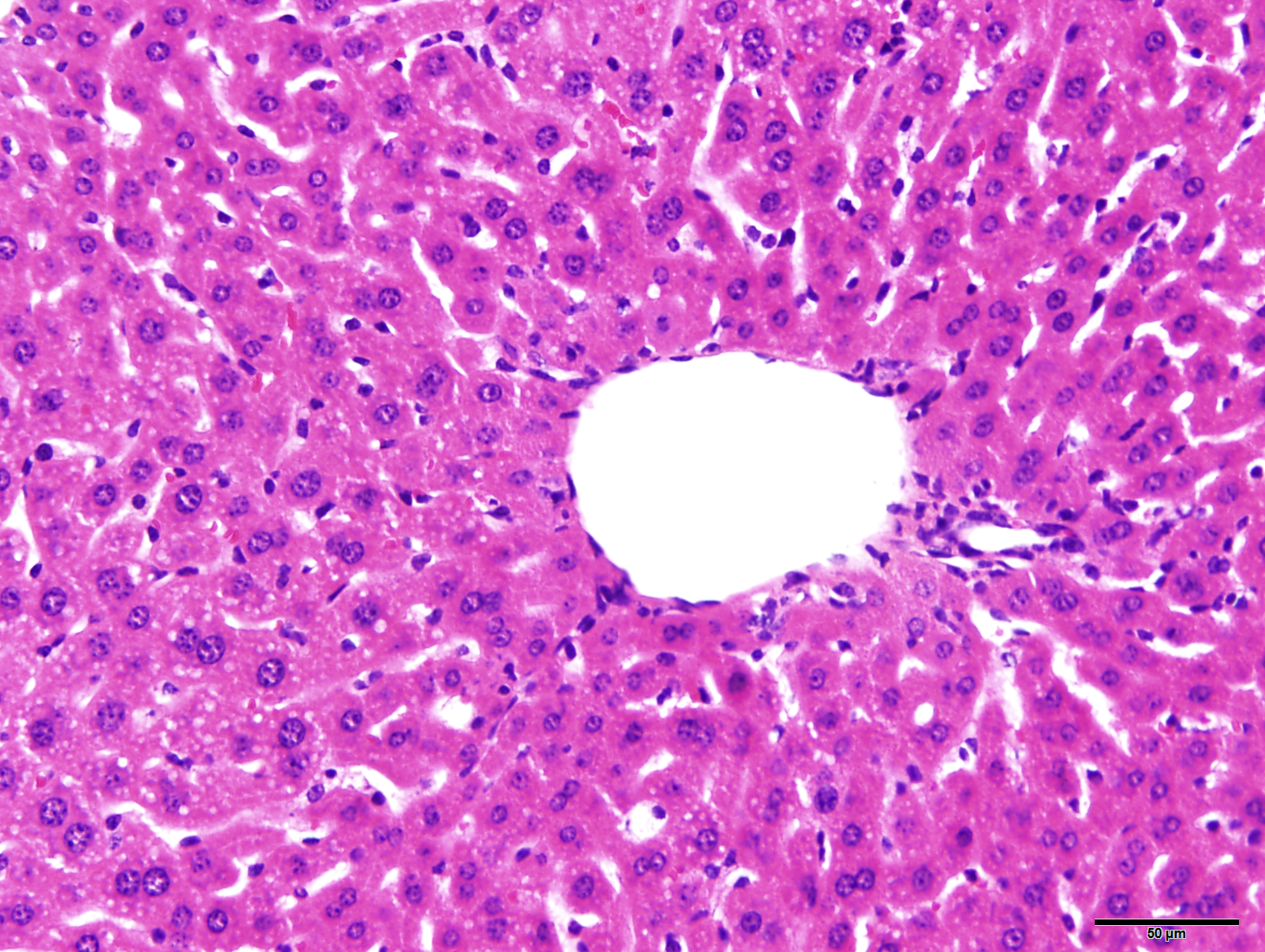

Supplement: S5 File — (ZIP) [file pone.0347758.s005.zip › Liver H&E staining/PSB-L/81.tif]

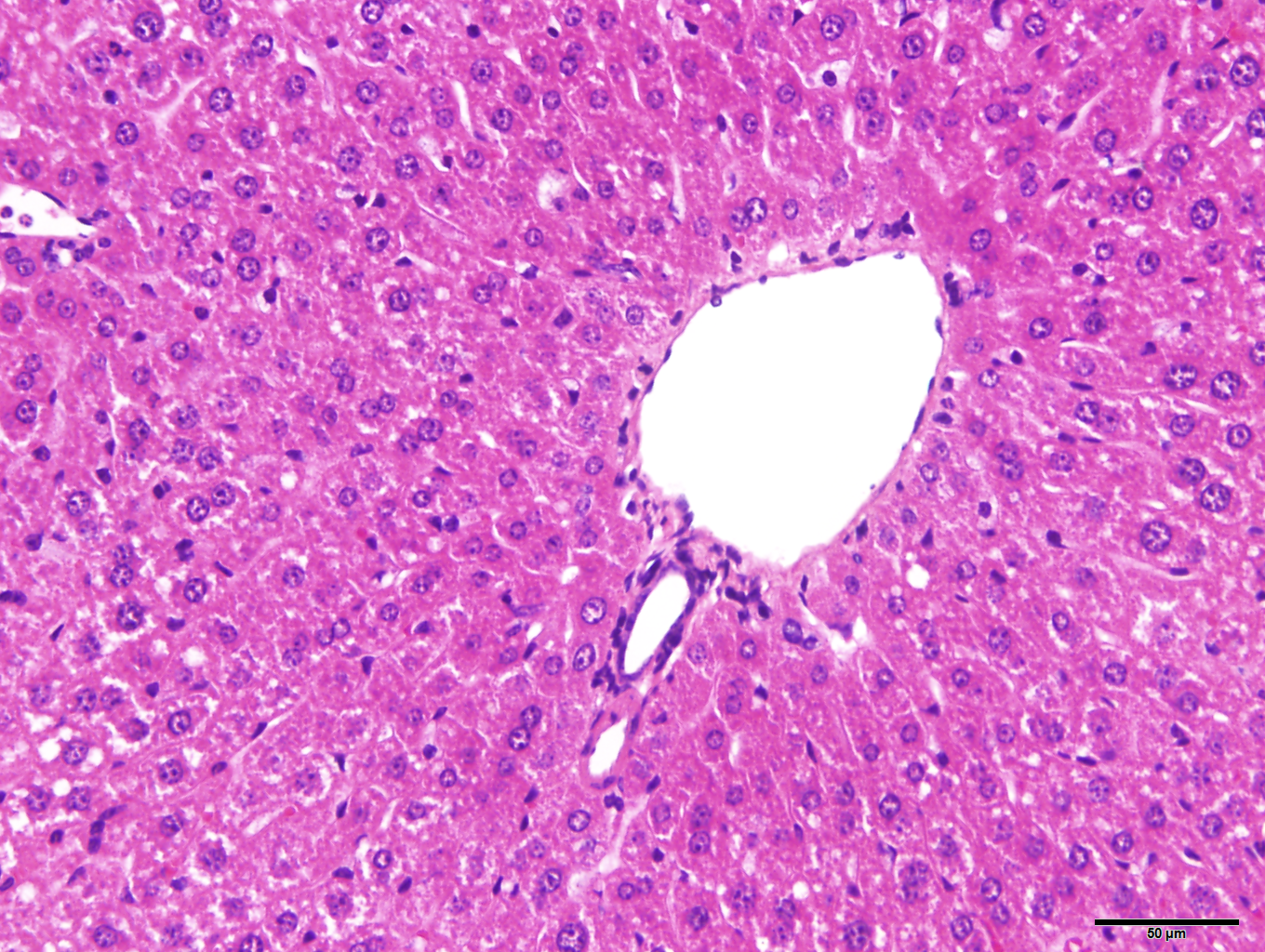

Supplement: S5 File — (ZIP) [file pone.0347758.s005.zip › Liver H&E staining/PSB-M/85.tif]

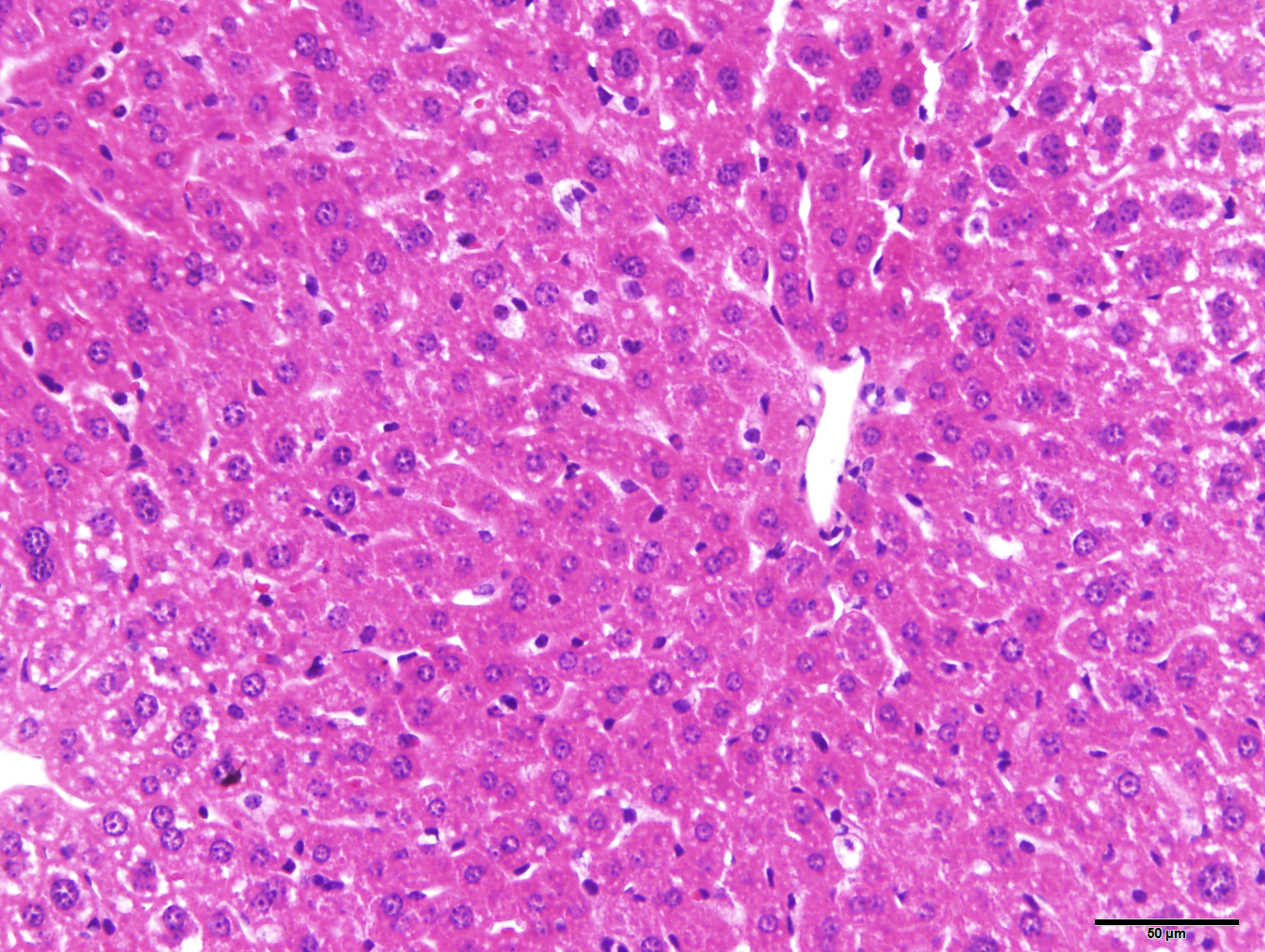

Supplement: S5 File — (ZIP) [file pone.0347758.s005.zip › Liver H&E staining/PSB-M/86.tif]

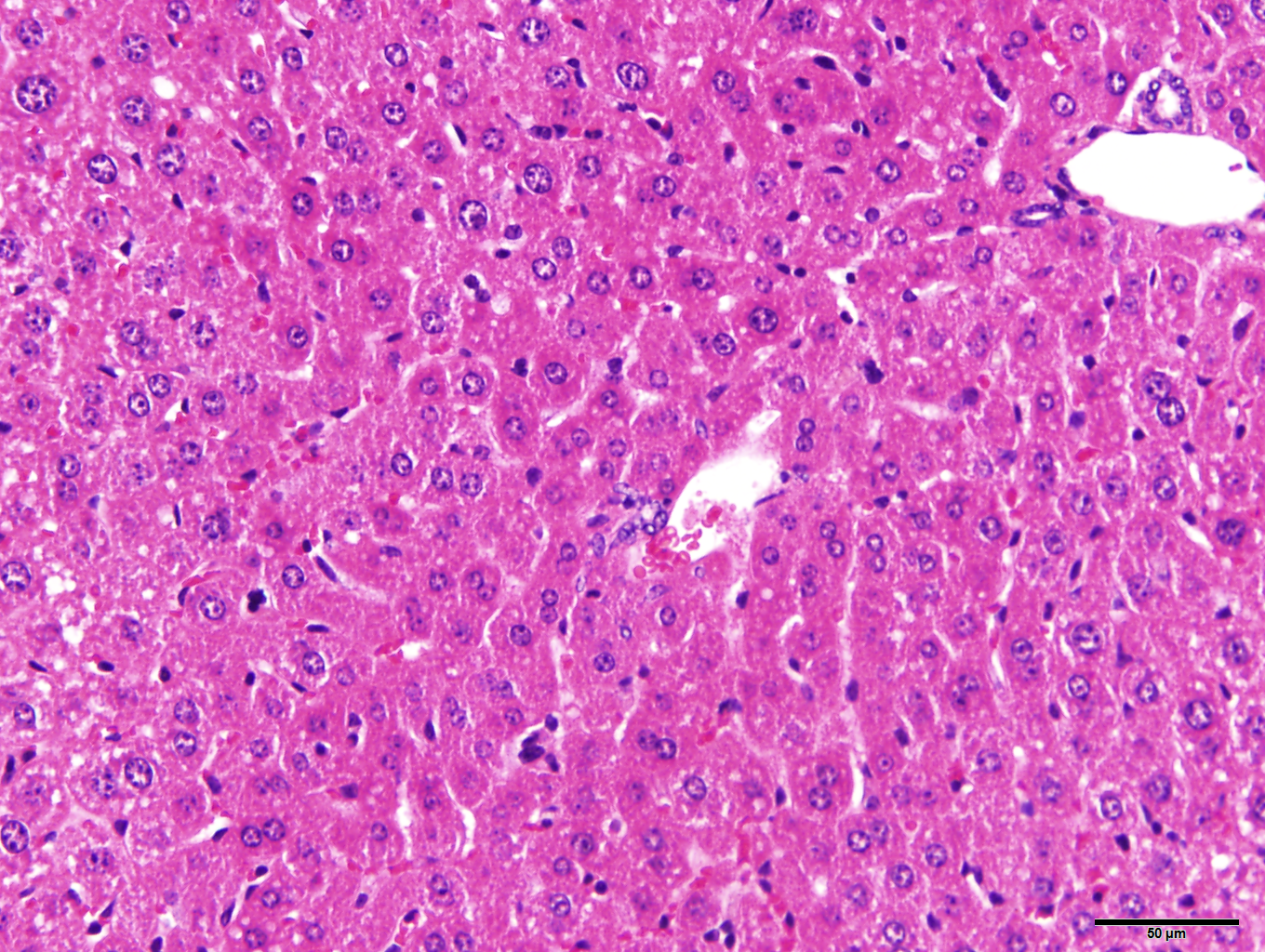

Supplement: S5 File — (ZIP) [file pone.0347758.s005.zip › Liver H&E staining/PSB-M/91.tif]

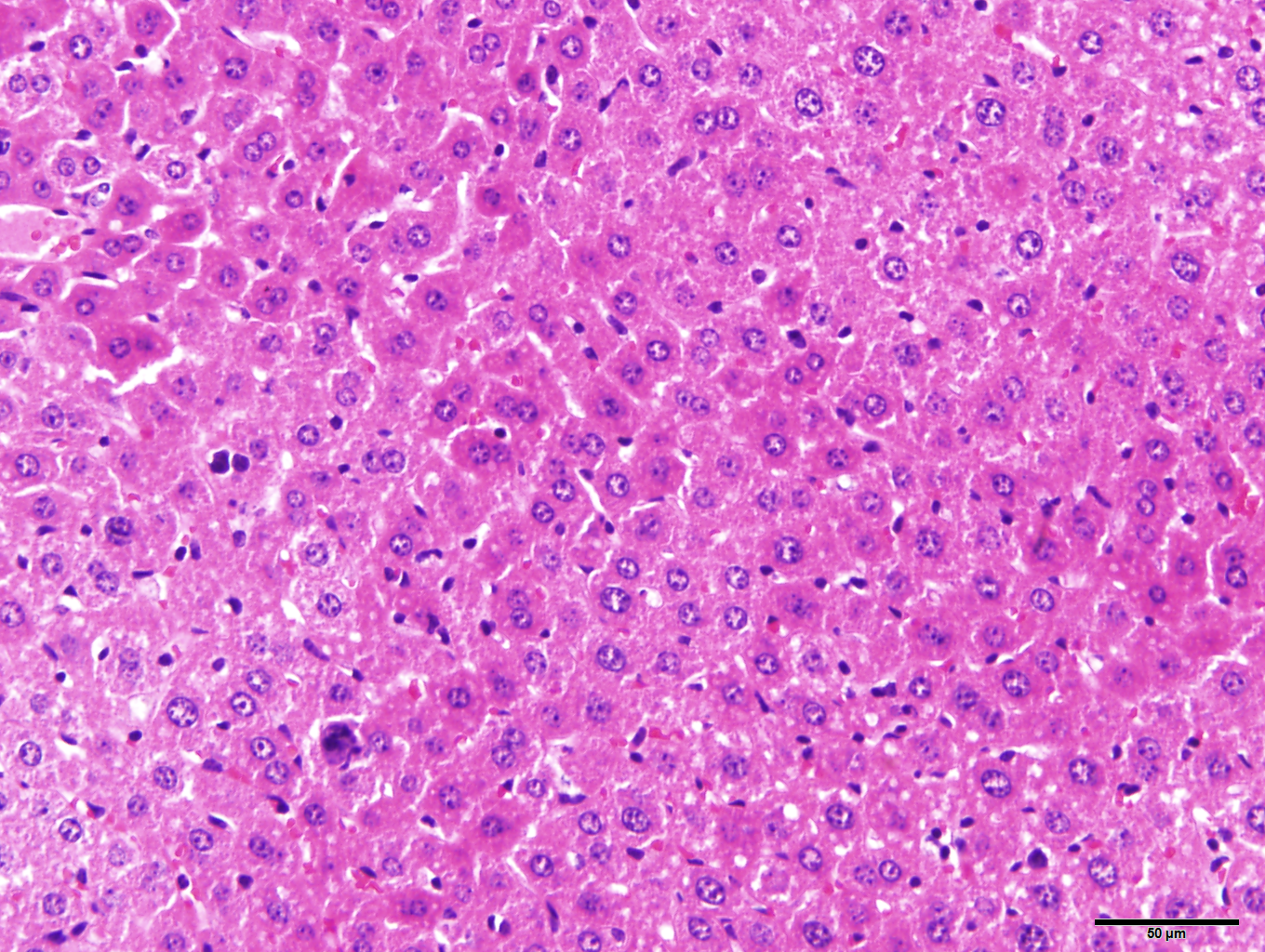

Supplement: S5 File — (ZIP) [file pone.0347758.s005.zip › Liver H&E staining/PSB-M/92.tif]

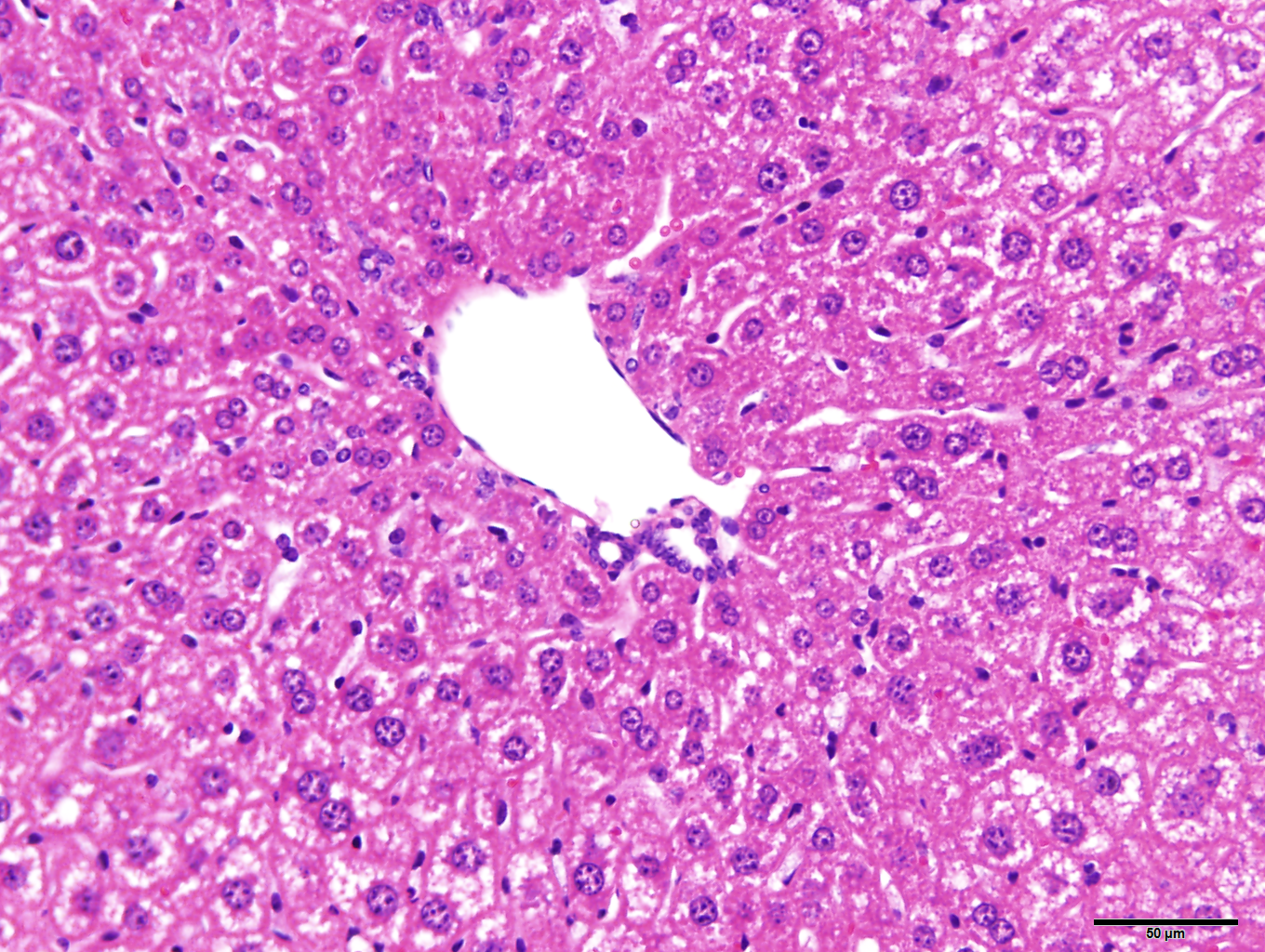

Supplement: S5 File — (ZIP) [file pone.0347758.s005.zip › Liver H&E staining/statin/35.tif]

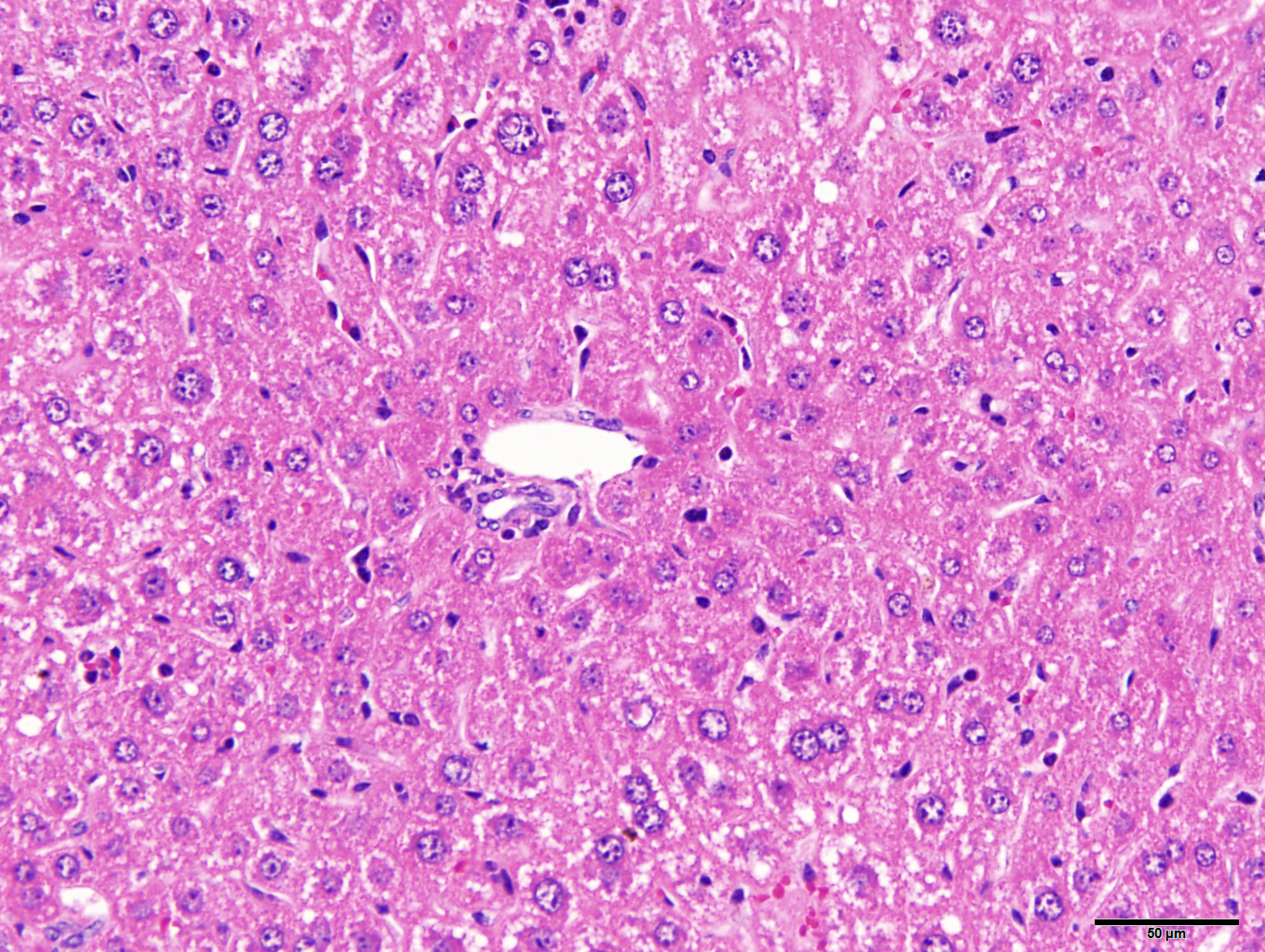

Supplement: S5 File — (ZIP) [file pone.0347758.s005.zip › Liver H&E staining/statin/36.tif]

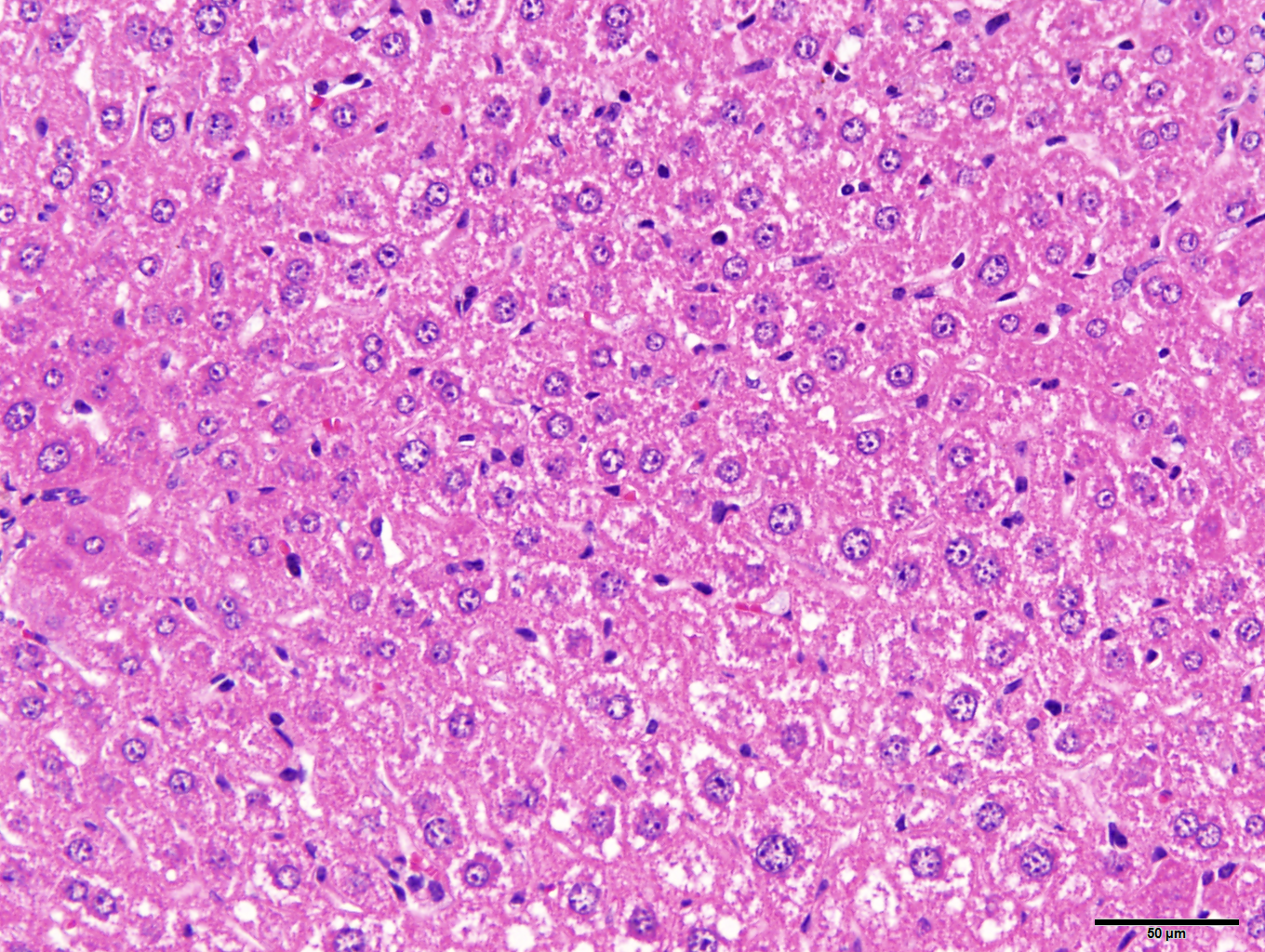

Supplement: S5 File — (ZIP) [file pone.0347758.s005.zip › Liver H&E staining/statin/38.tif]

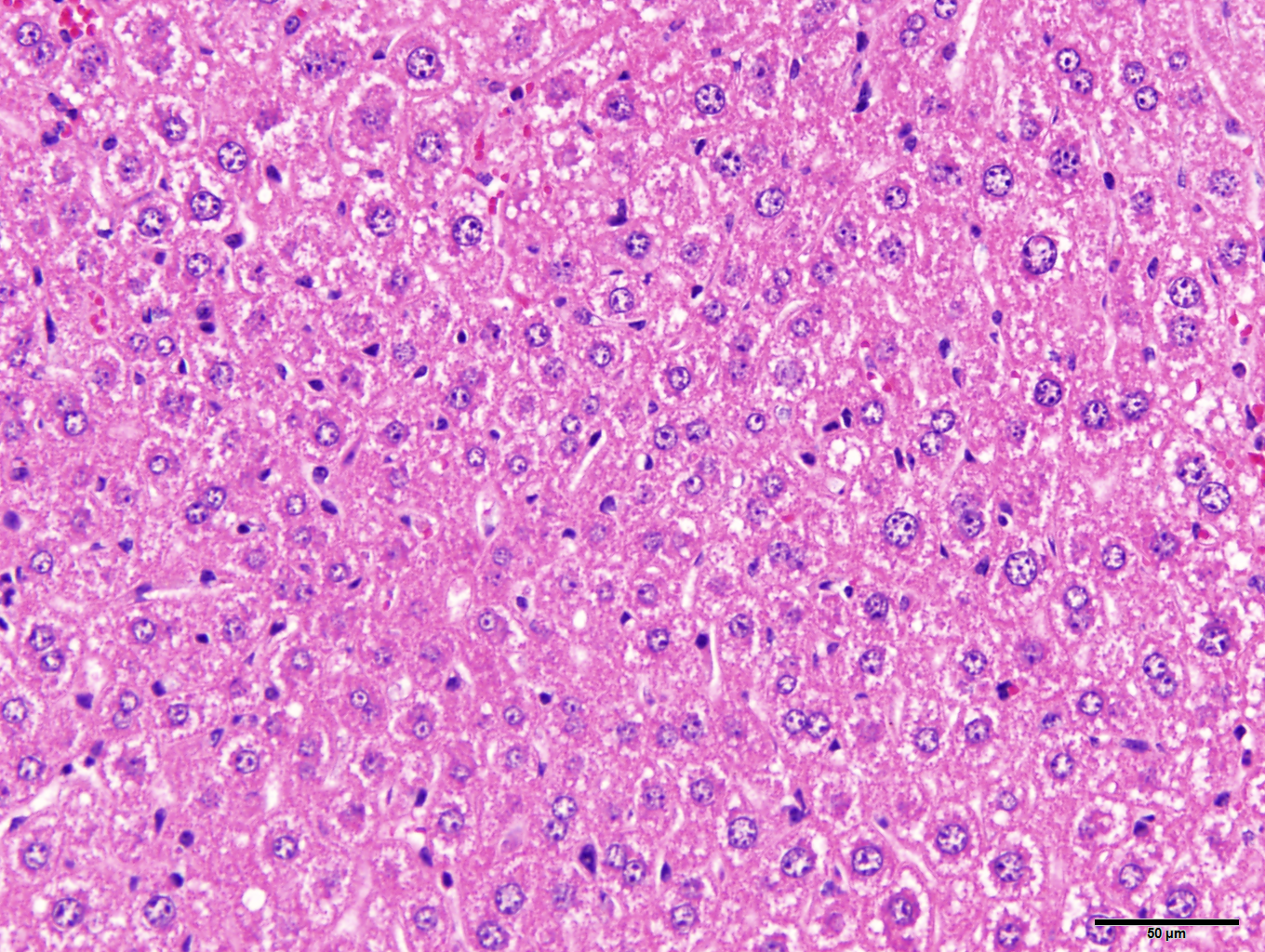

Supplement: S5 File — (ZIP) [file pone.0347758.s005.zip › Liver H&E staining/statin/40.tif]

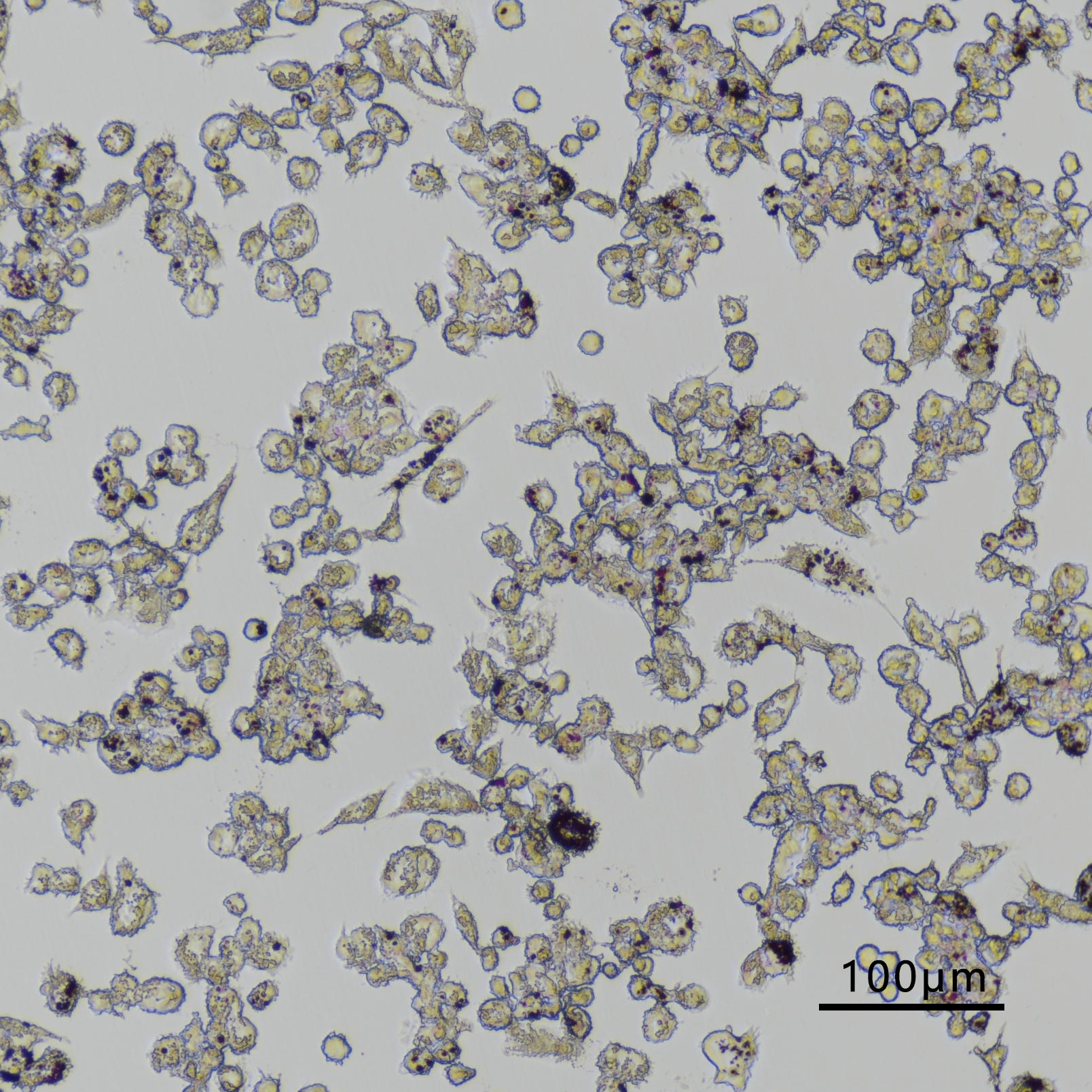

Supplement: S6 File — (ZIP) [file pone.0347758.s006.zip › cell Oil red O staining/control/1.tif]

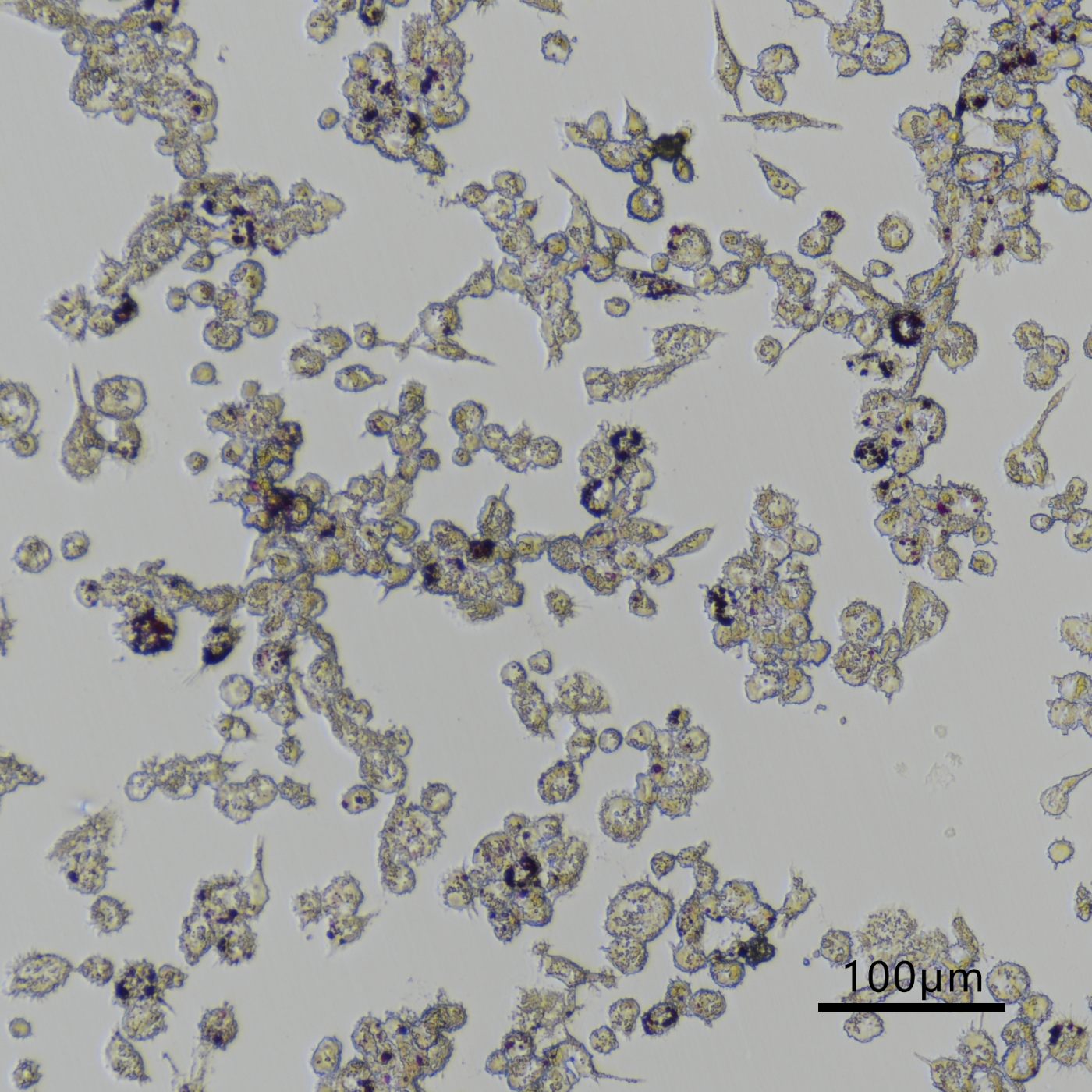

Supplement: S6 File — (ZIP) [file pone.0347758.s006.zip › cell Oil red O staining/control/2.tif]

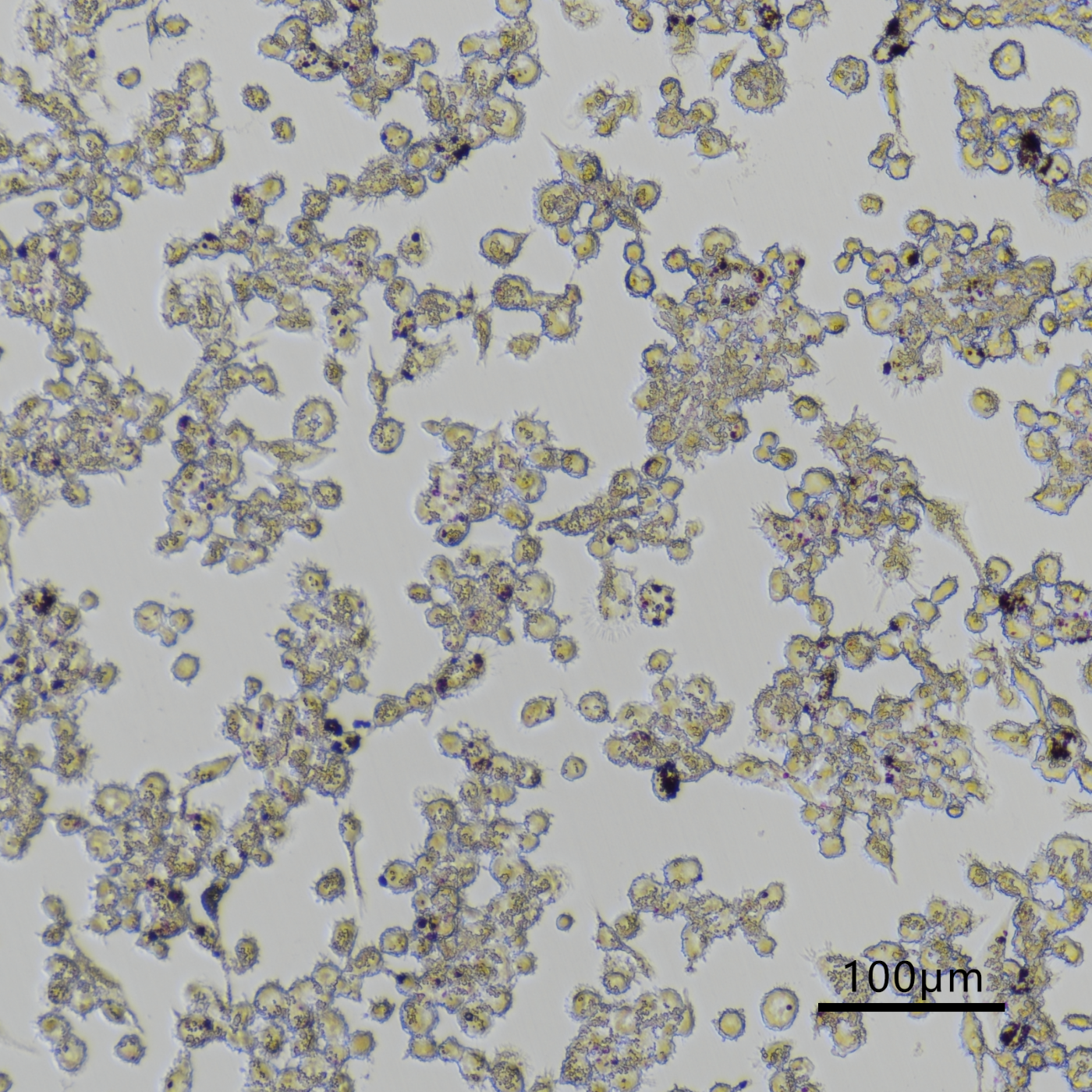

Supplement: S6 File — (ZIP) [file pone.0347758.s006.zip › cell Oil red O staining/control/3.tif]

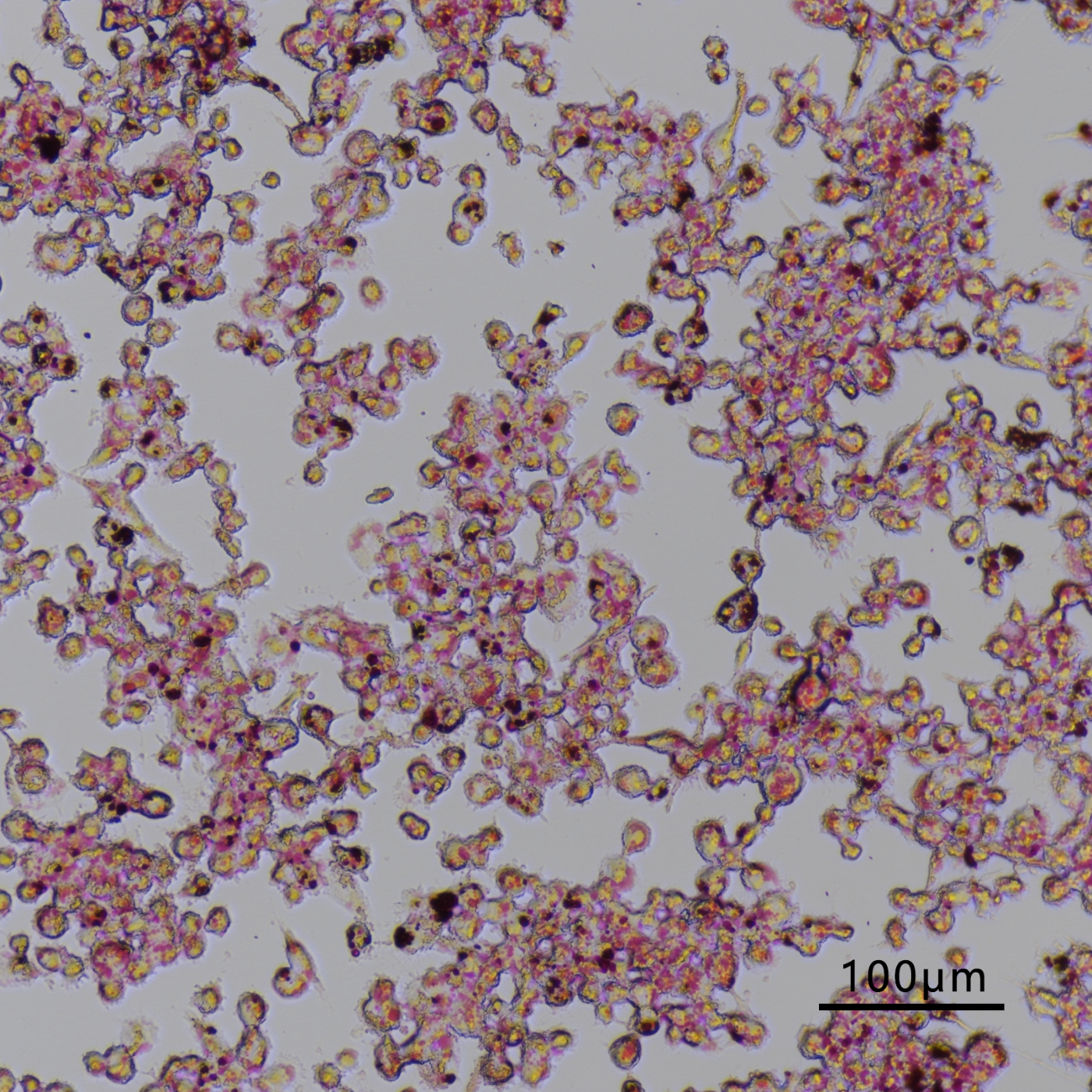

Supplement: S6 File — (ZIP) [file pone.0347758.s006.zip › cell Oil red O staining/ox-LDL/1.tif]

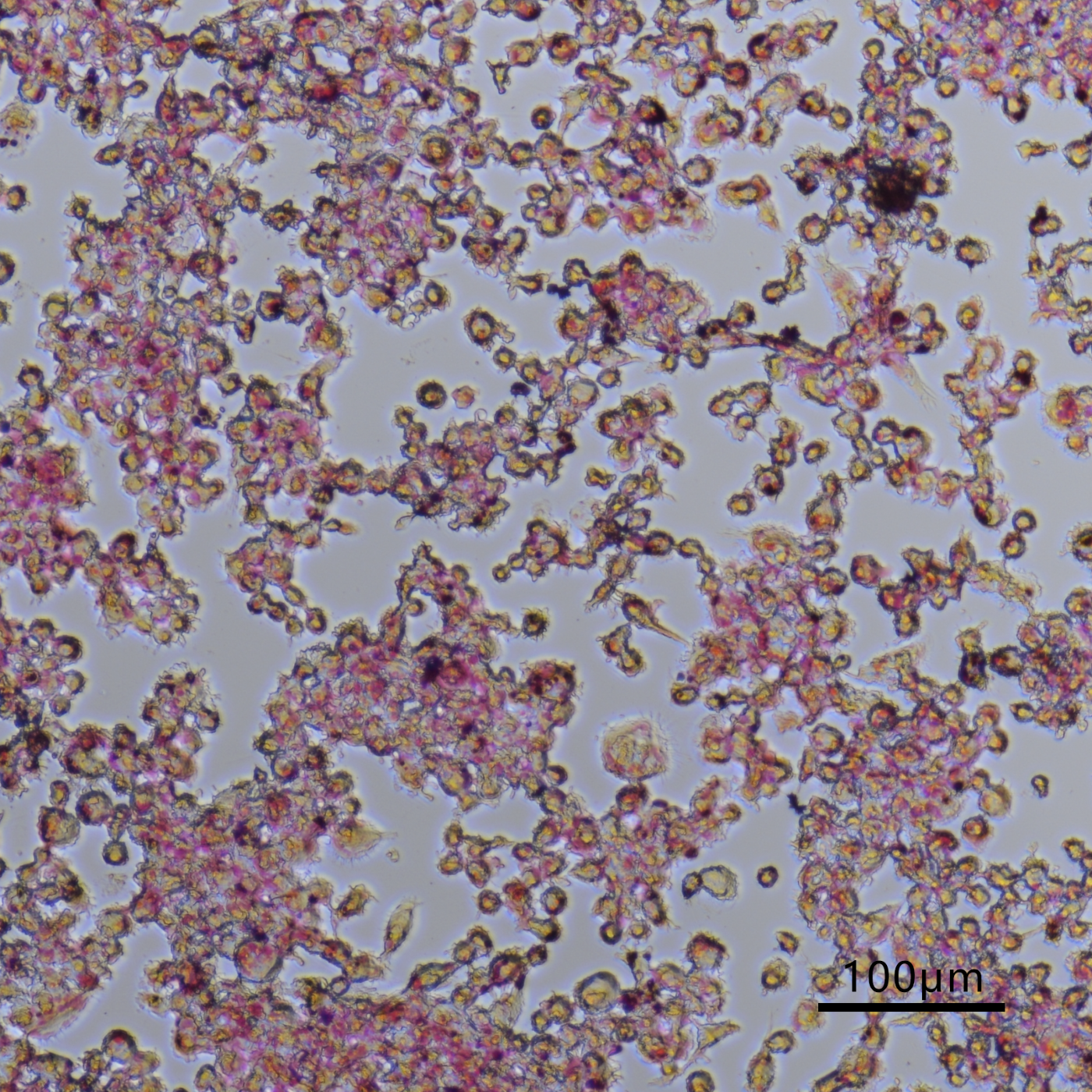

Supplement: S6 File — (ZIP) [file pone.0347758.s006.zip › cell Oil red O staining/ox-LDL/2.tif]

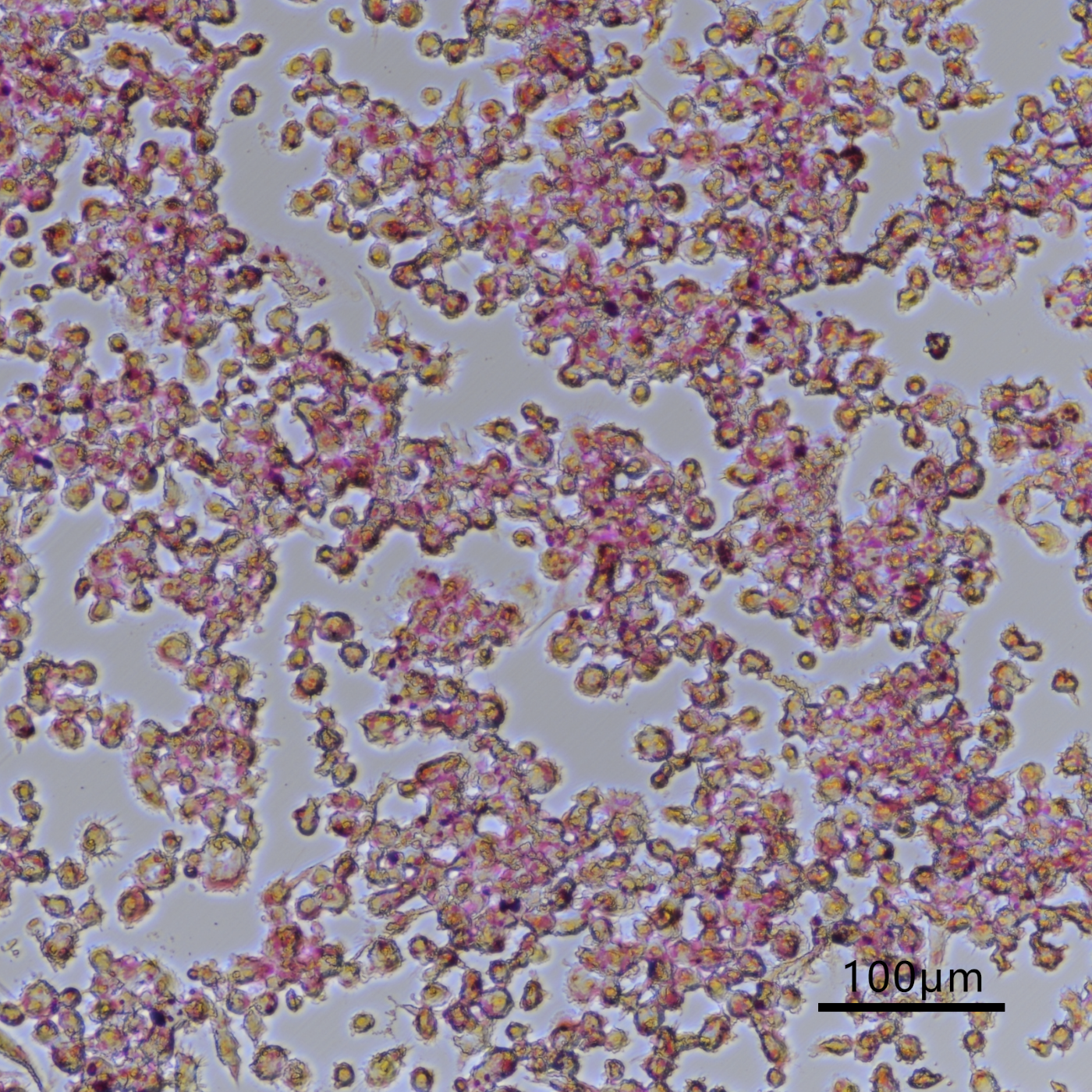

Supplement: S6 File — (ZIP) [file pone.0347758.s006.zip › cell Oil red O staining/ox-LDL/3.tif]

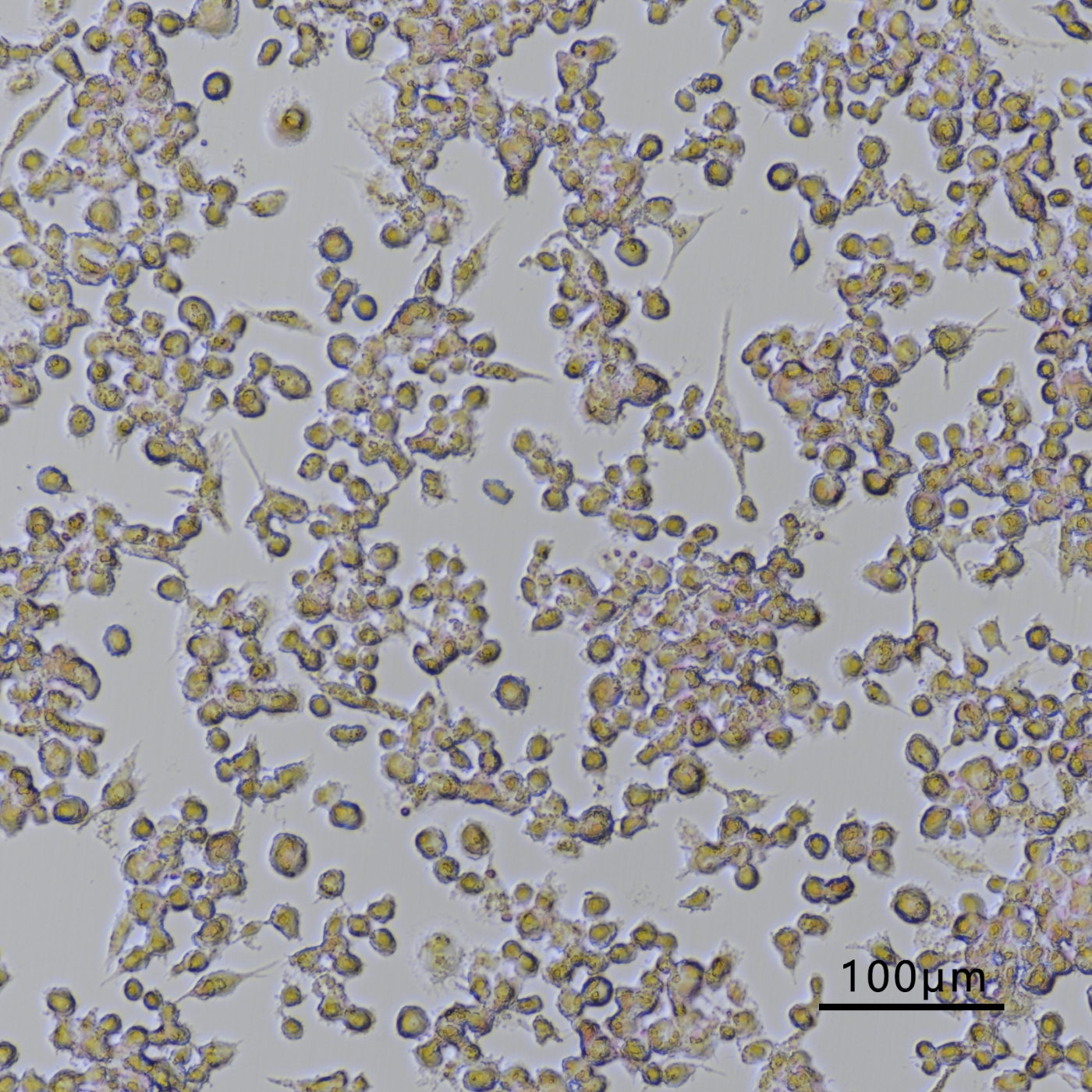

Supplement: S6 File — (ZIP) [file pone.0347758.s006.zip › cell Oil red O staining/PSB-H/1.tif]

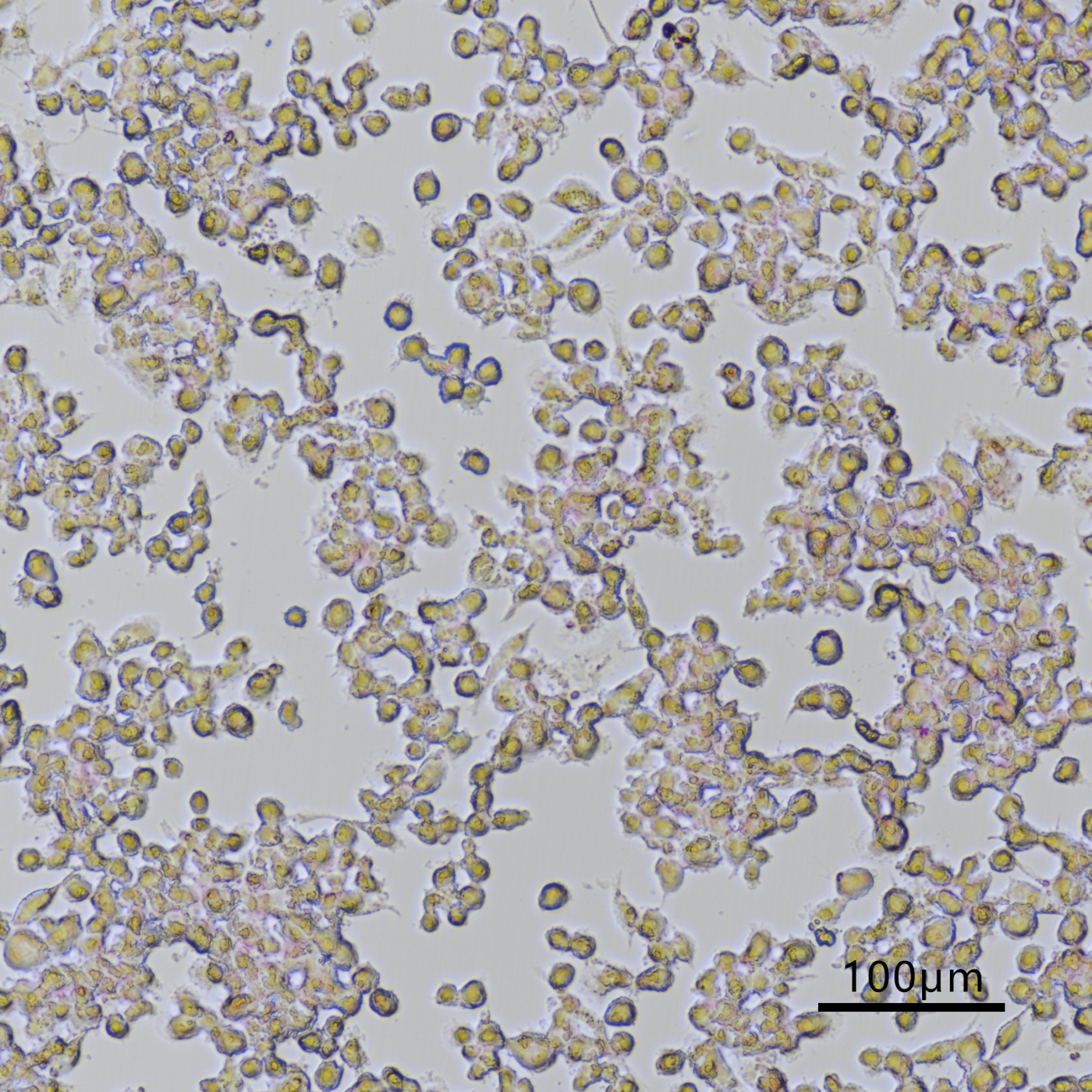

Supplement: S6 File — (ZIP) [file pone.0347758.s006.zip › cell Oil red O staining/PSB-H/2.tif]

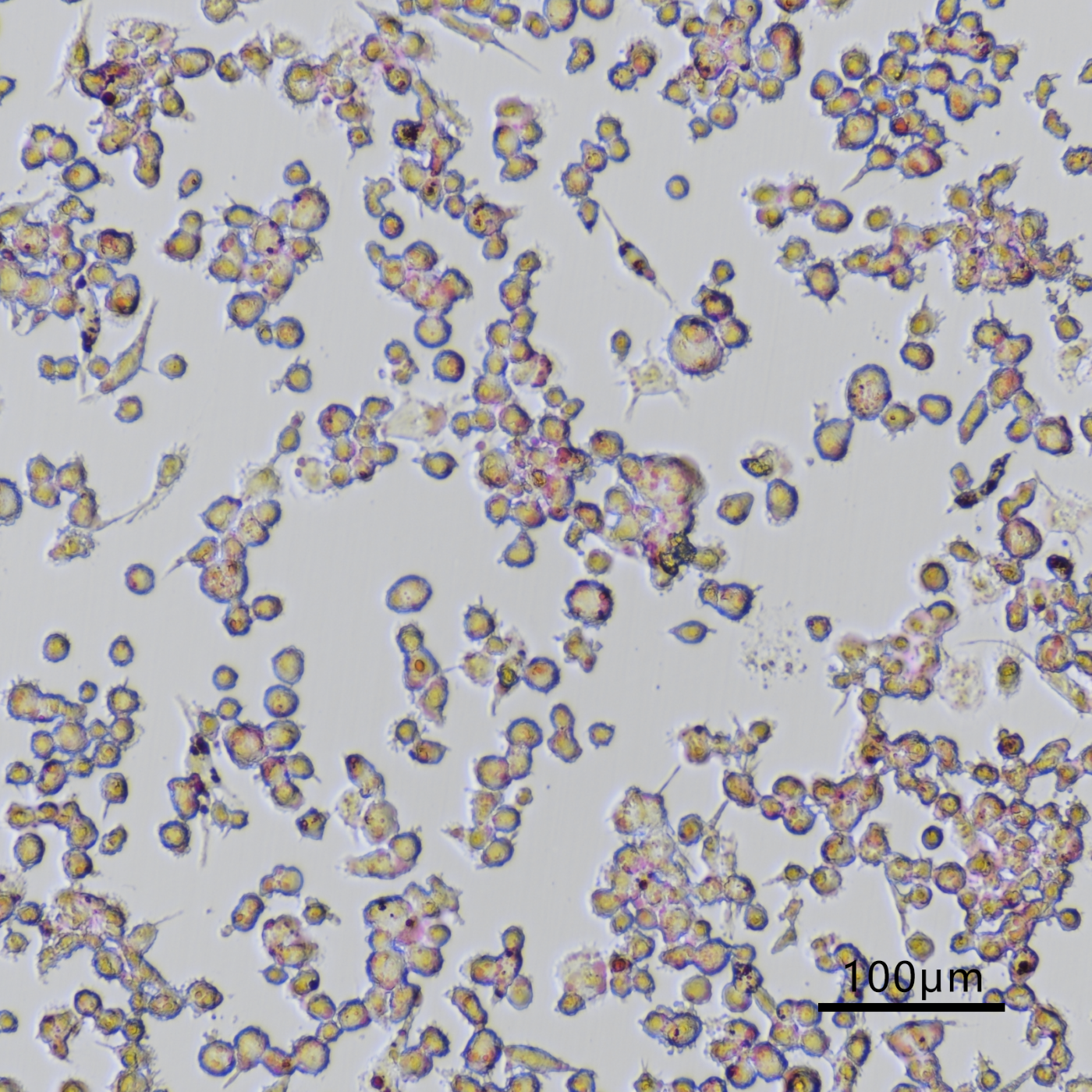

Supplement: S6 File — (ZIP) [file pone.0347758.s006.zip › cell Oil red O staining/PSB-H/3.tif]

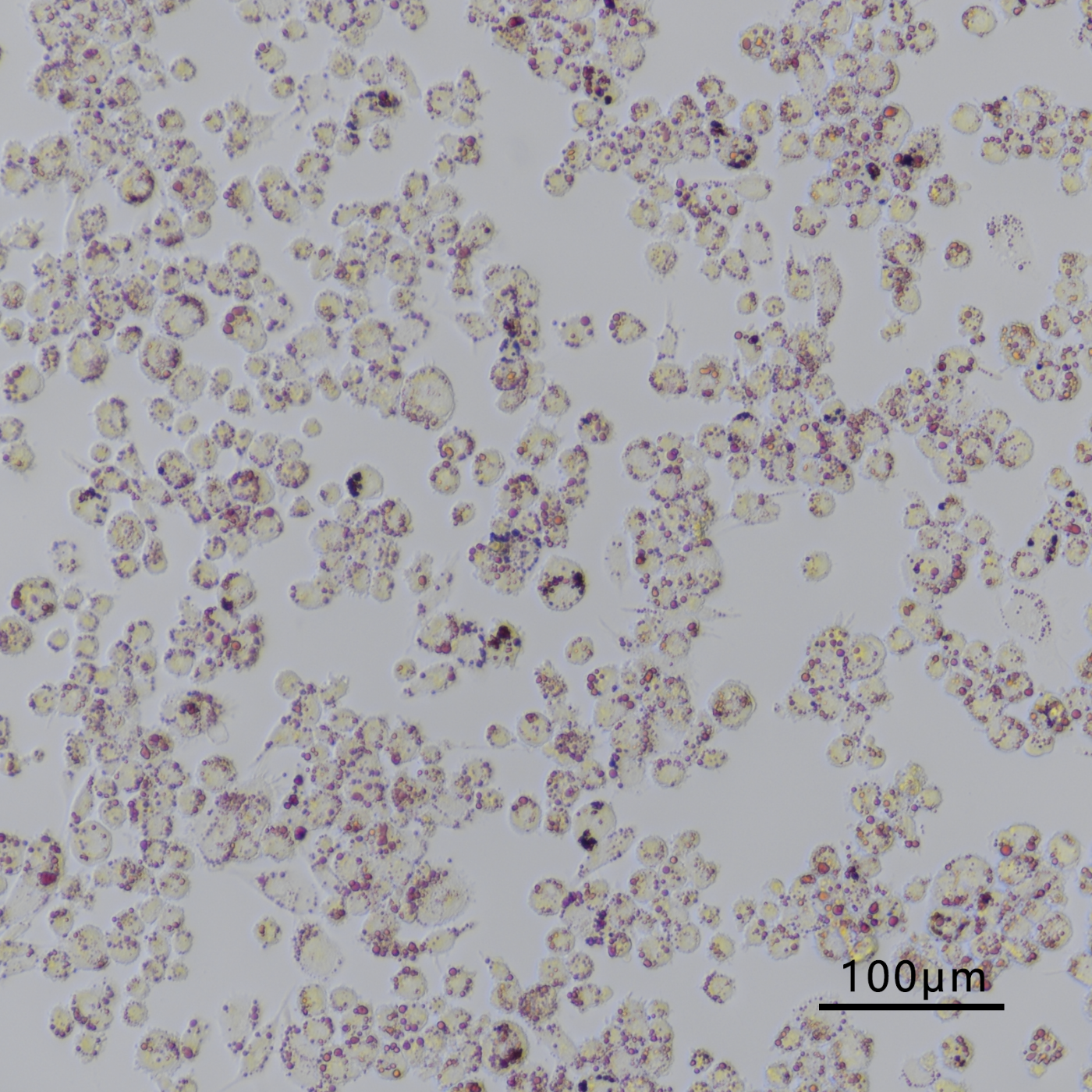

Supplement: S6 File — (ZIP) [file pone.0347758.s006.zip › cell Oil red O staining/PSB-L/L-1.tif]

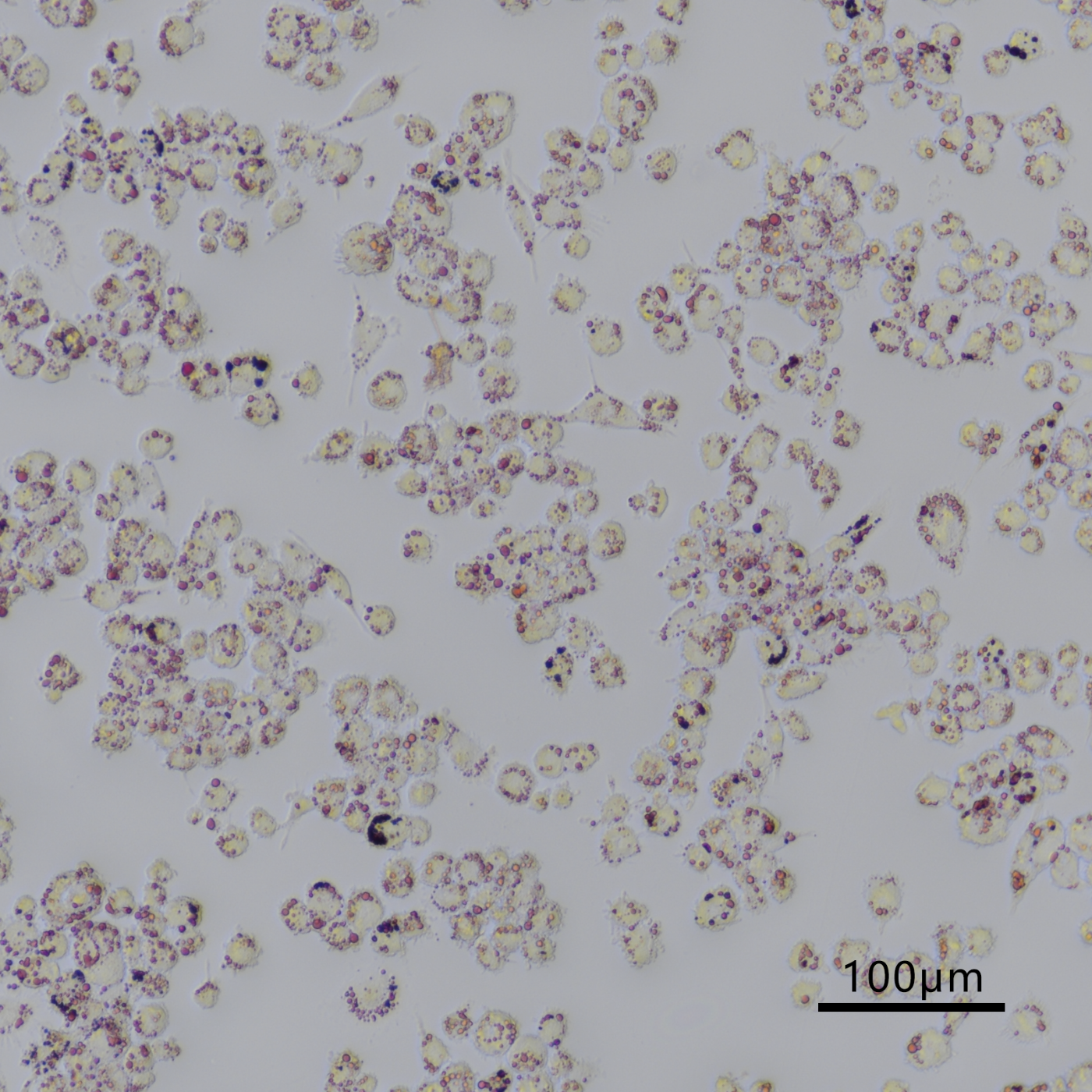

Supplement: S6 File — (ZIP) [file pone.0347758.s006.zip › cell Oil red O staining/PSB-L/L-2.tif]

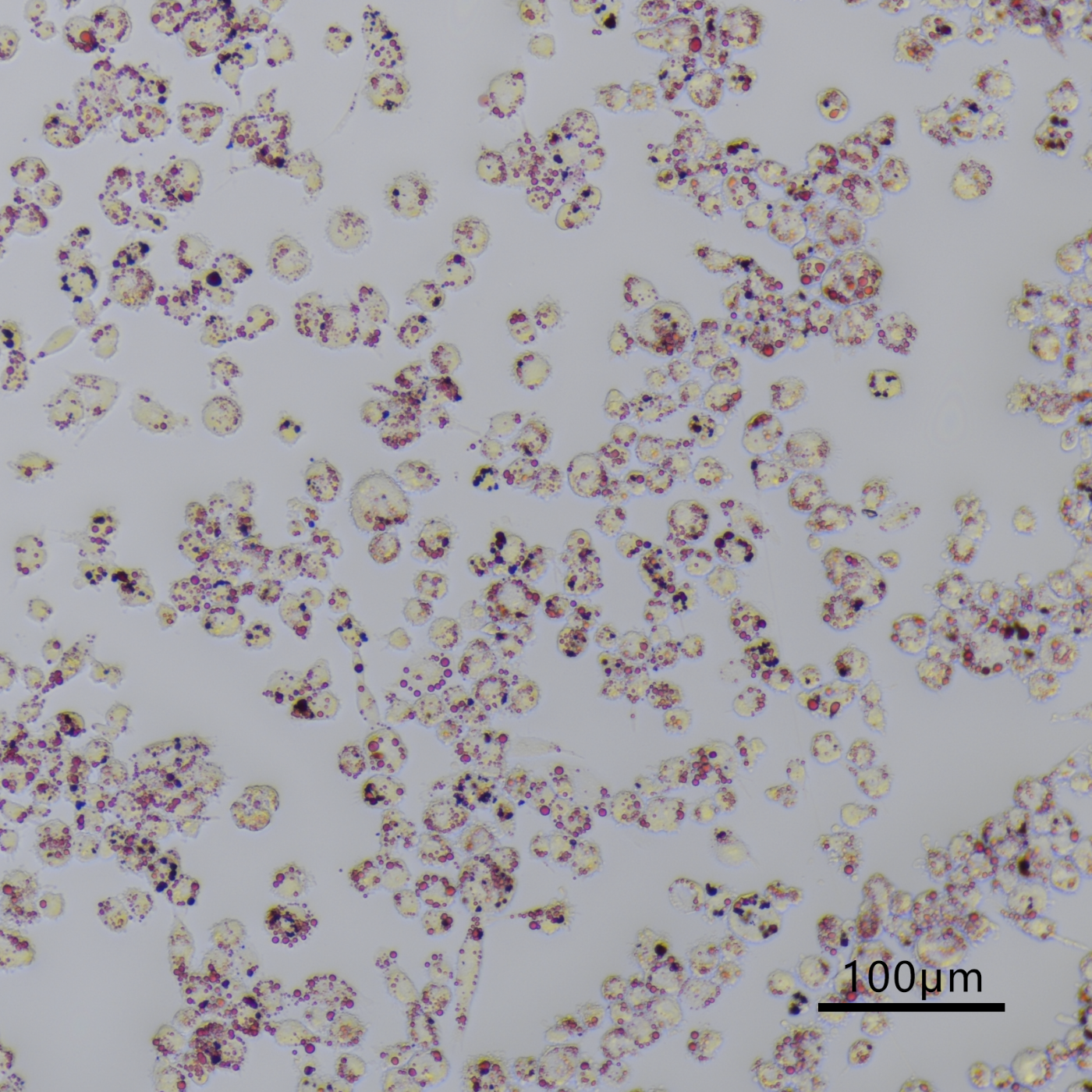

Supplement: S6 File — (ZIP) [file pone.0347758.s006.zip › cell Oil red O staining/PSB-L/L-3.tif]

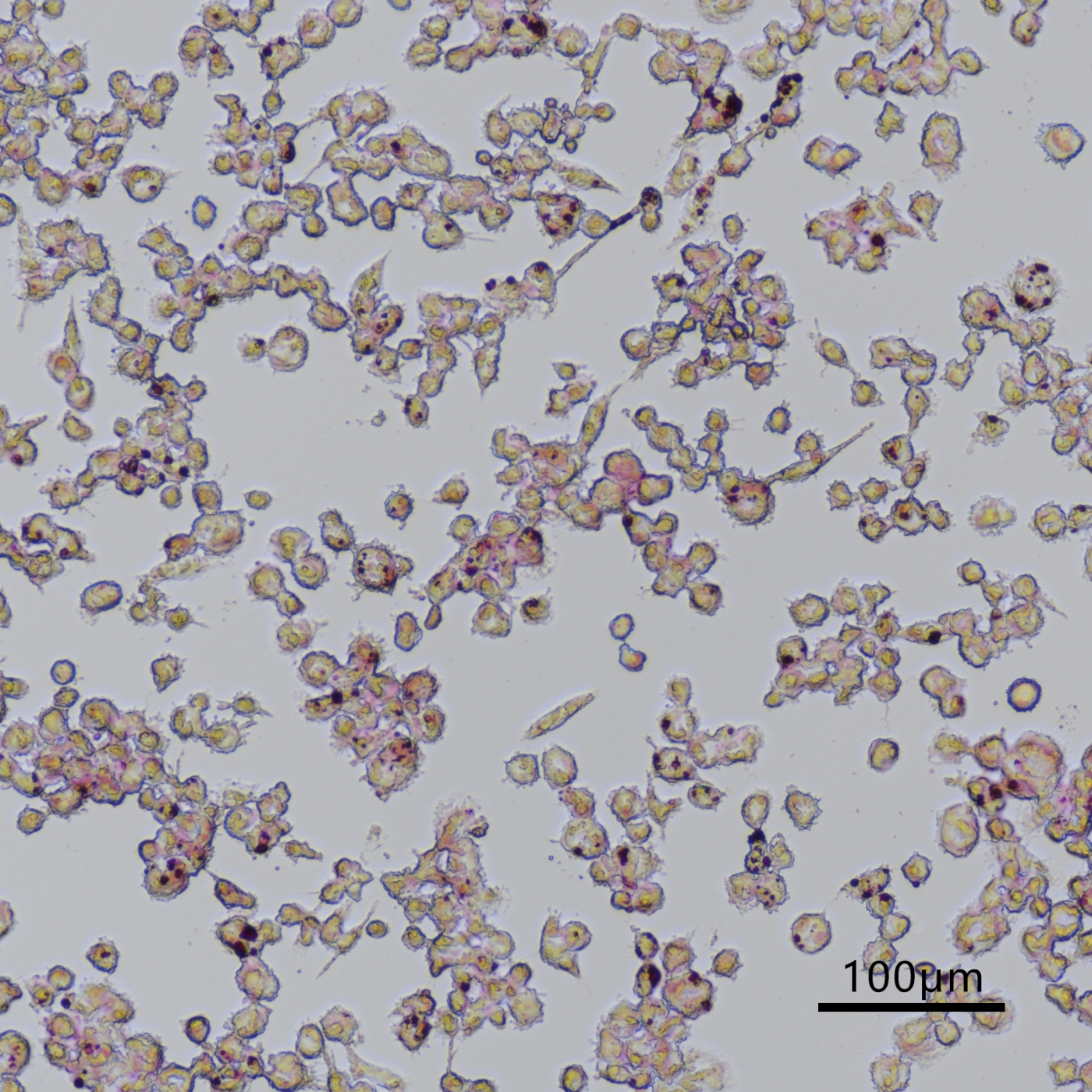

Supplement: S6 File — (ZIP) [file pone.0347758.s006.zip › cell Oil red O staining/PSB-M/M-1.tif]

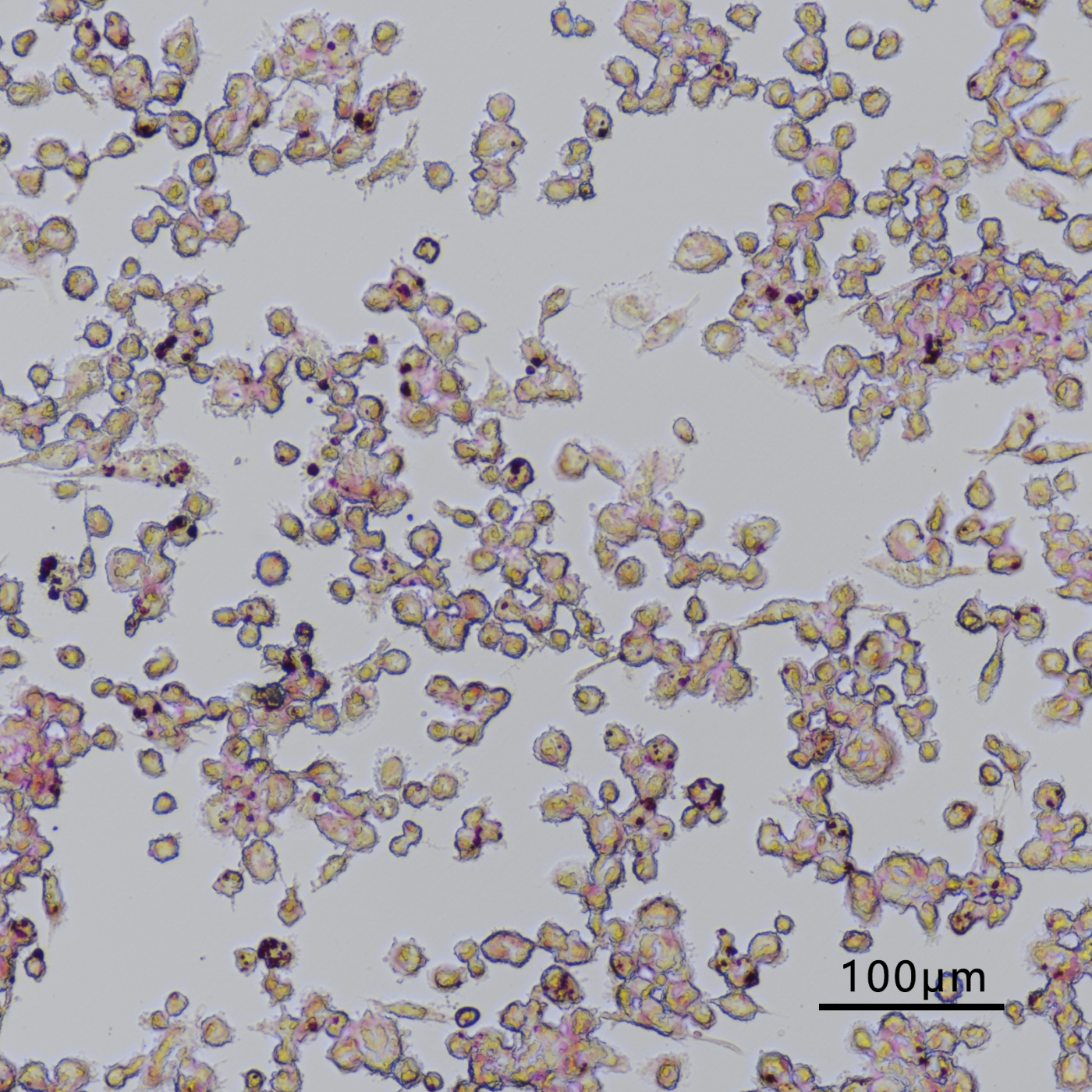

Supplement: S6 File — (ZIP) [file pone.0347758.s006.zip › cell Oil red O staining/PSB-M/M-2.tif]

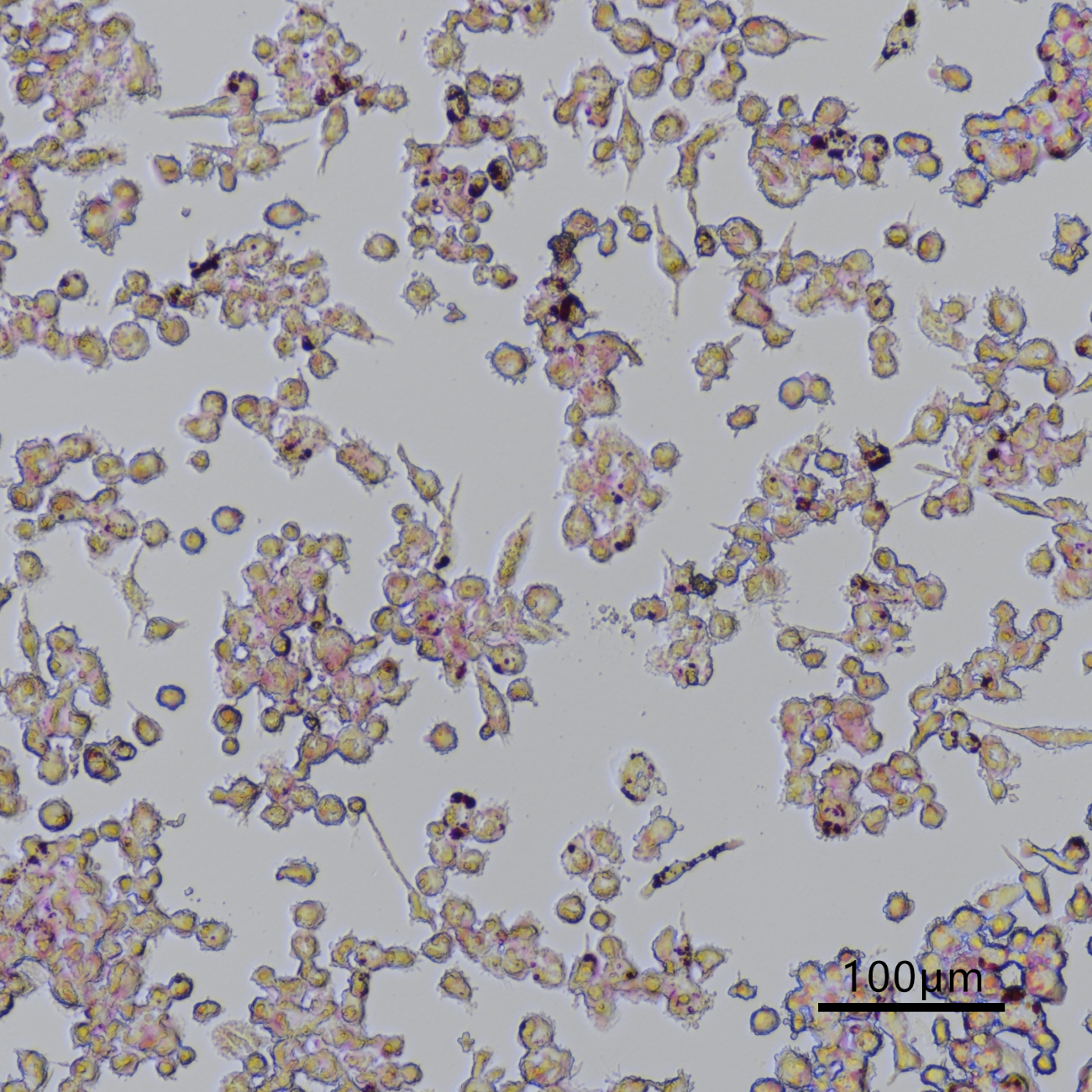

Supplement: S6 File — (ZIP) [file pone.0347758.s006.zip › cell Oil red O staining/PSB-M/M-3.tif]

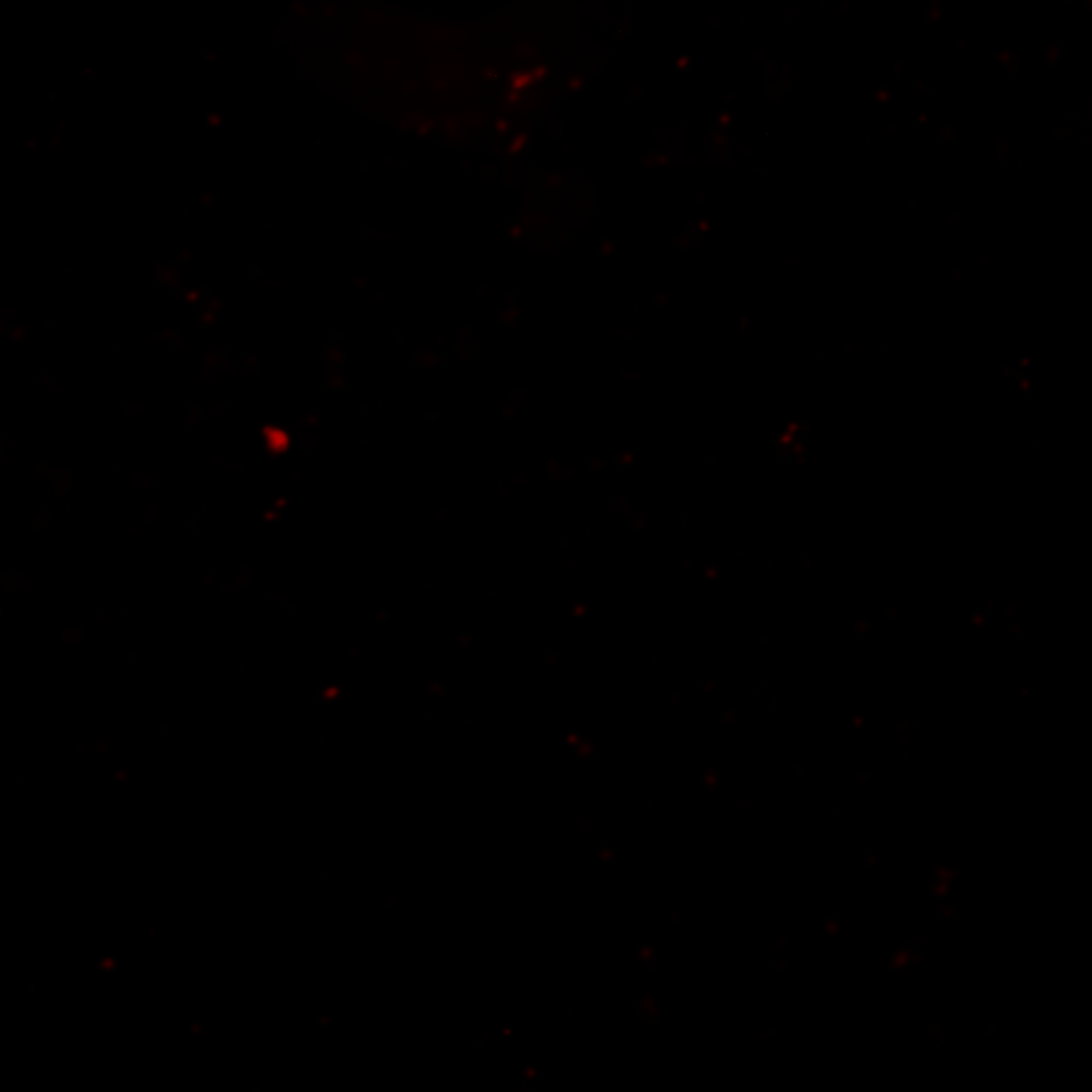

Supplement: S7 File — (ZIP) [file pone.0347758.s007.zip › cell ROS/control/blank1.tif]

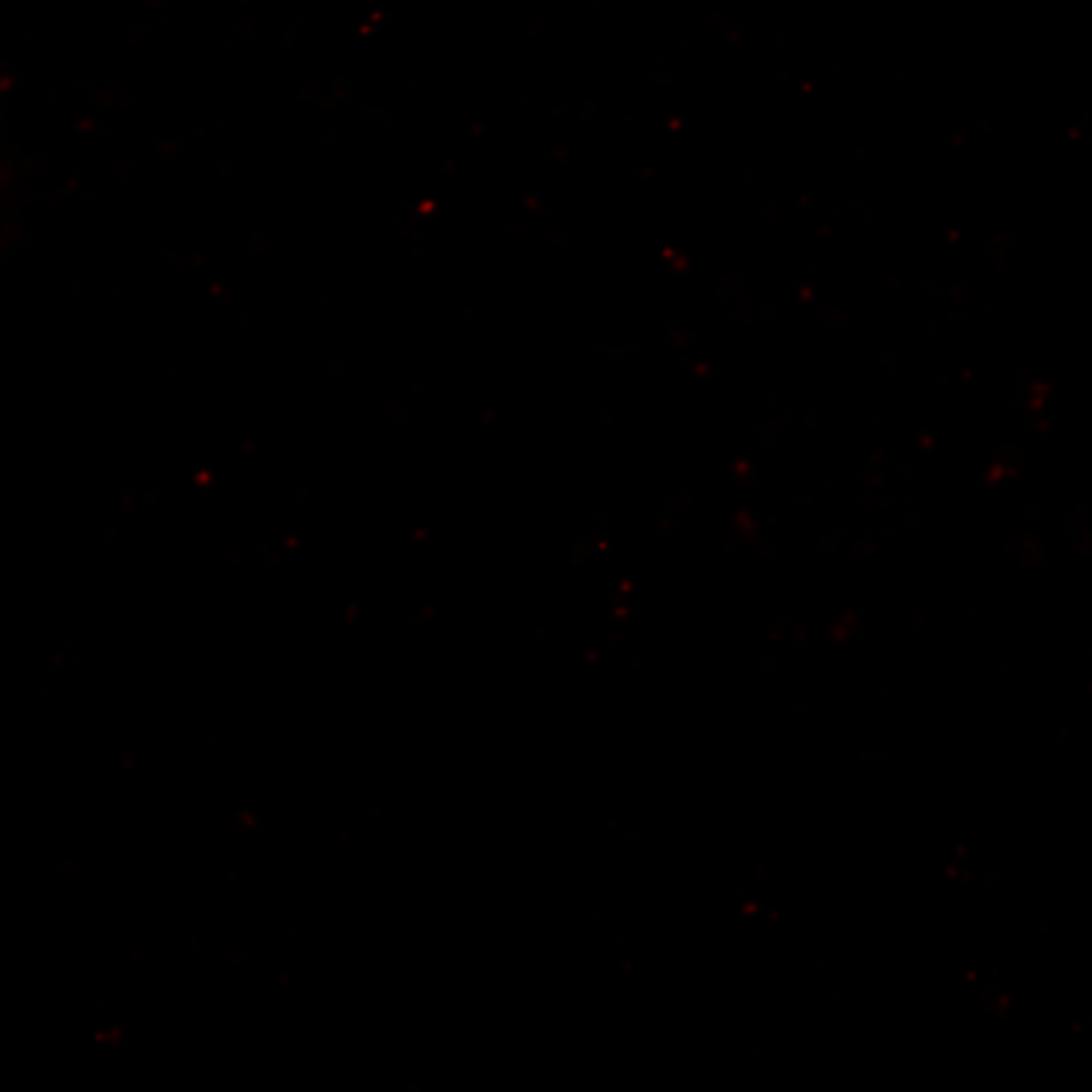

Supplement: S7 File — (ZIP) [file pone.0347758.s007.zip › cell ROS/control/blank2.tif]

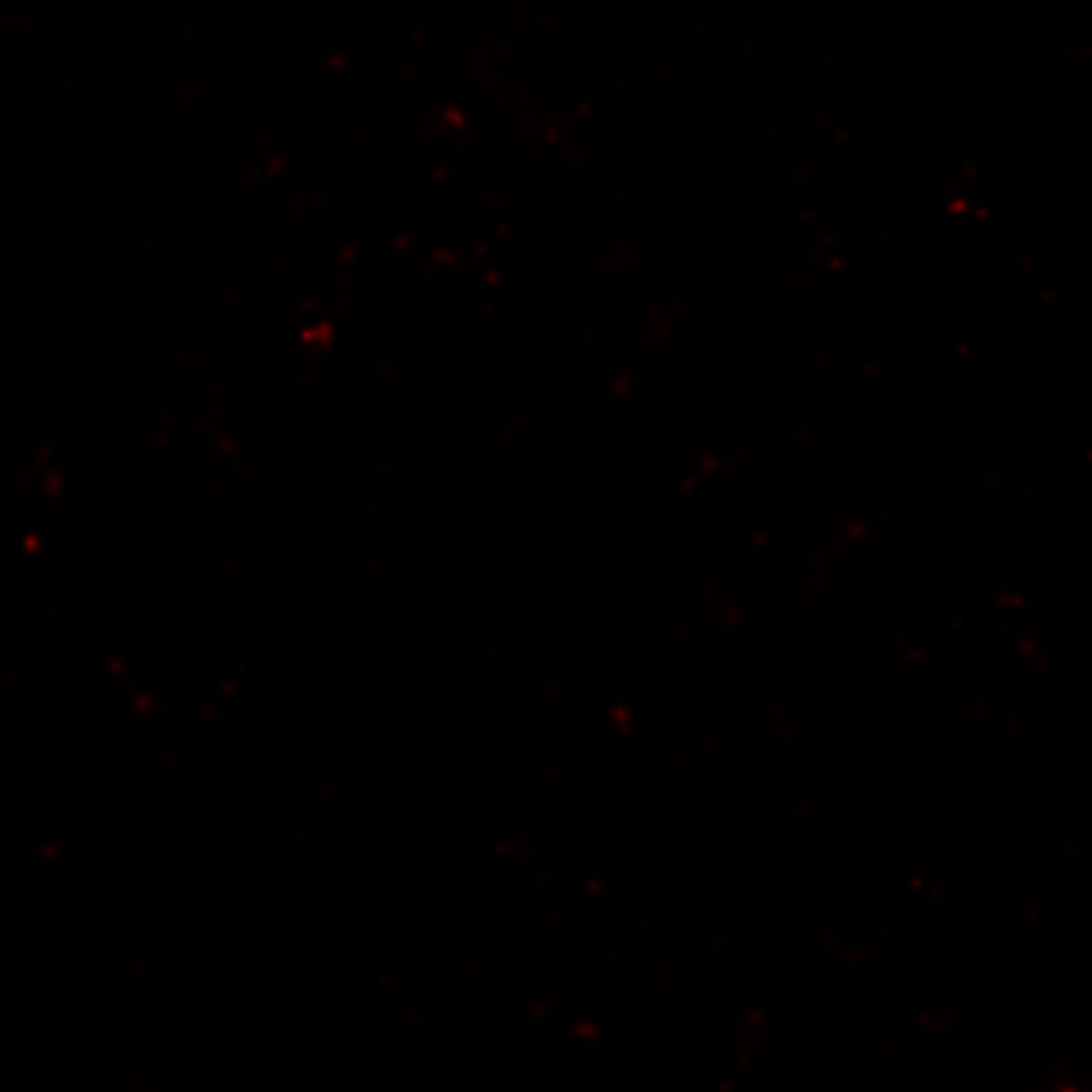

Supplement: S7 File — (ZIP) [file pone.0347758.s007.zip › cell ROS/control/blank3.tif]

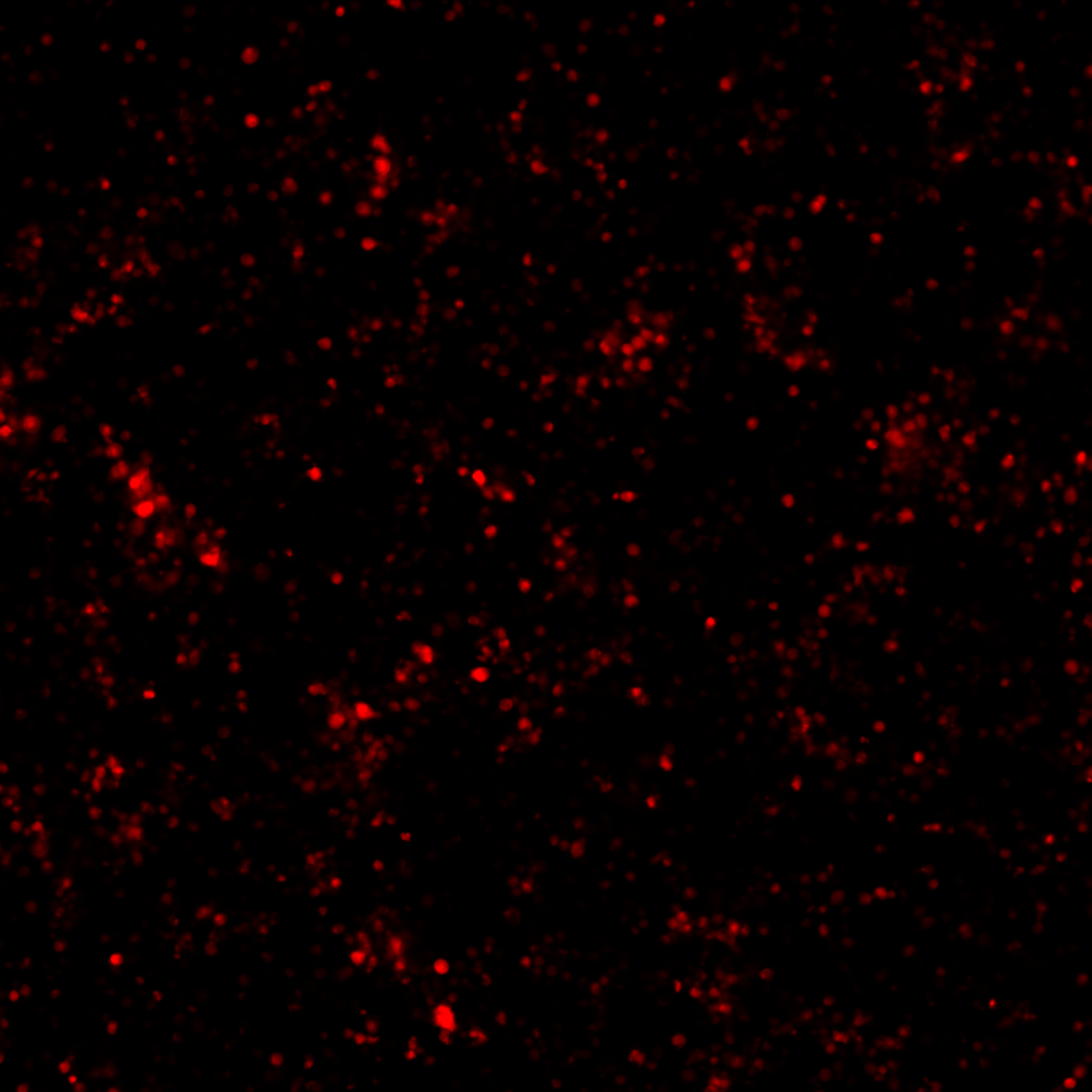

Supplement: S7 File — (ZIP) [file pone.0347758.s007.zip › cell ROS/ox-LDL/m3_RGB.tif]

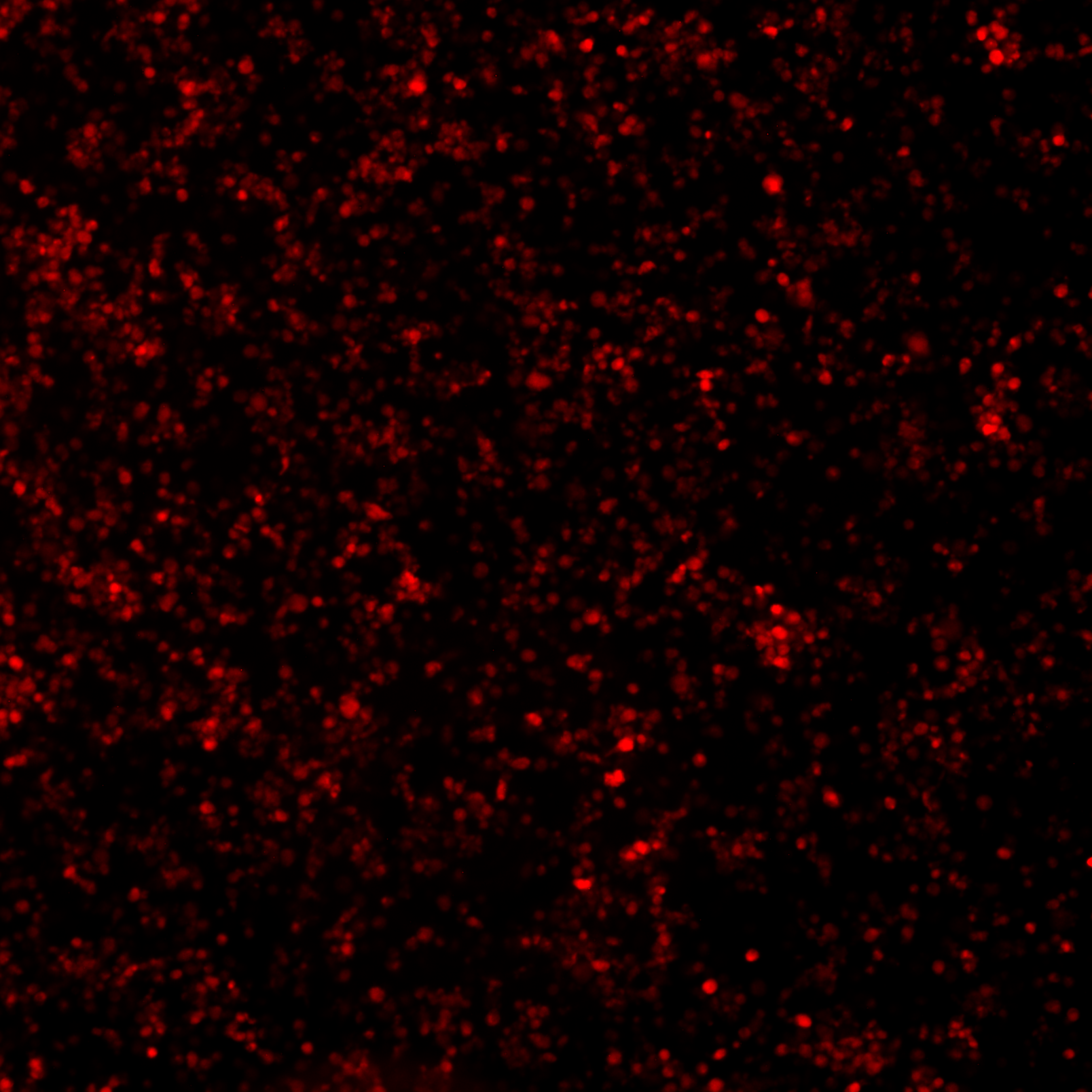

Supplement: S7 File — (ZIP) [file pone.0347758.s007.zip › cell ROS/ox-LDL/m5_RGB.tif]

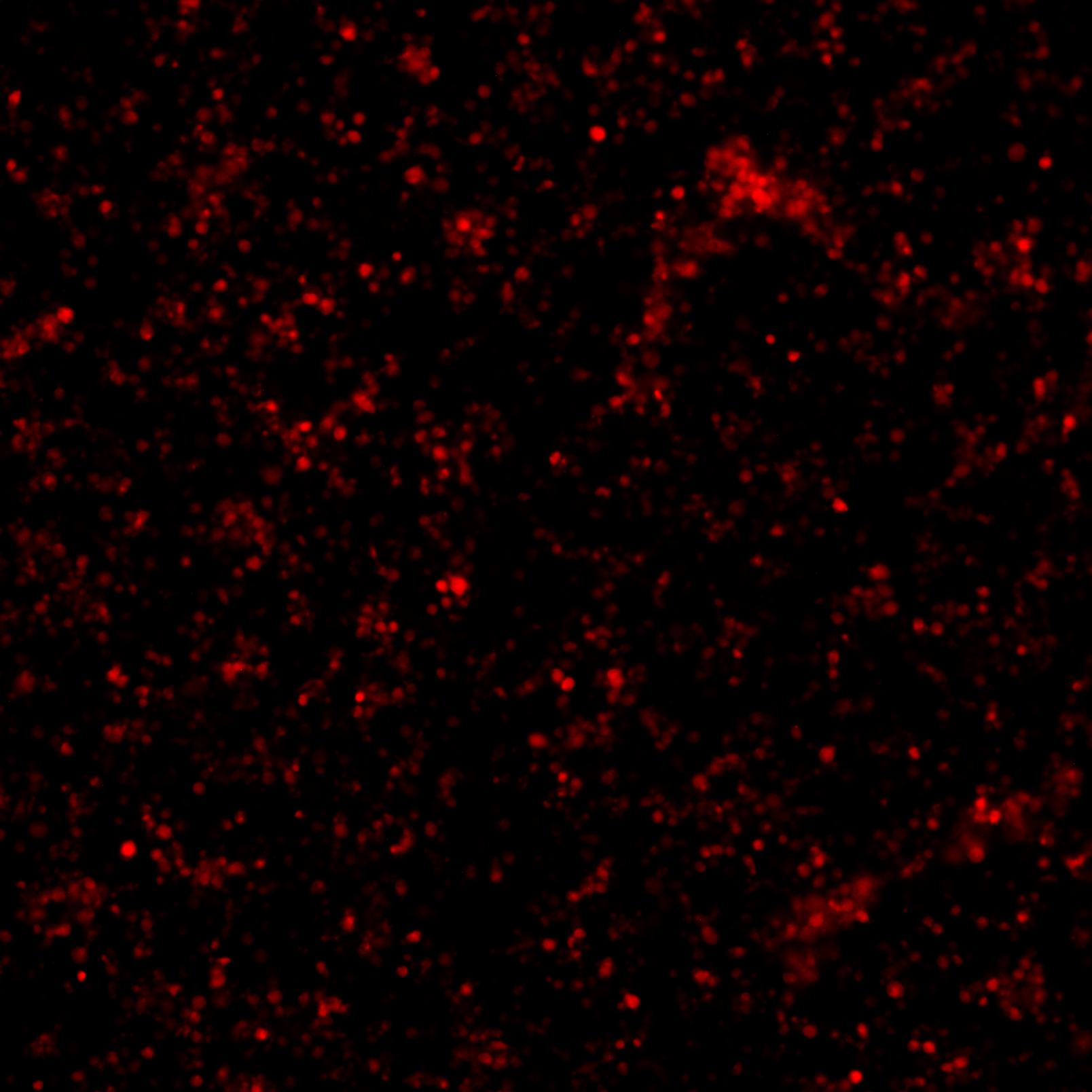

Supplement: S7 File — (ZIP) [file pone.0347758.s007.zip › cell ROS/ox-LDL/m6_RGB.tif]

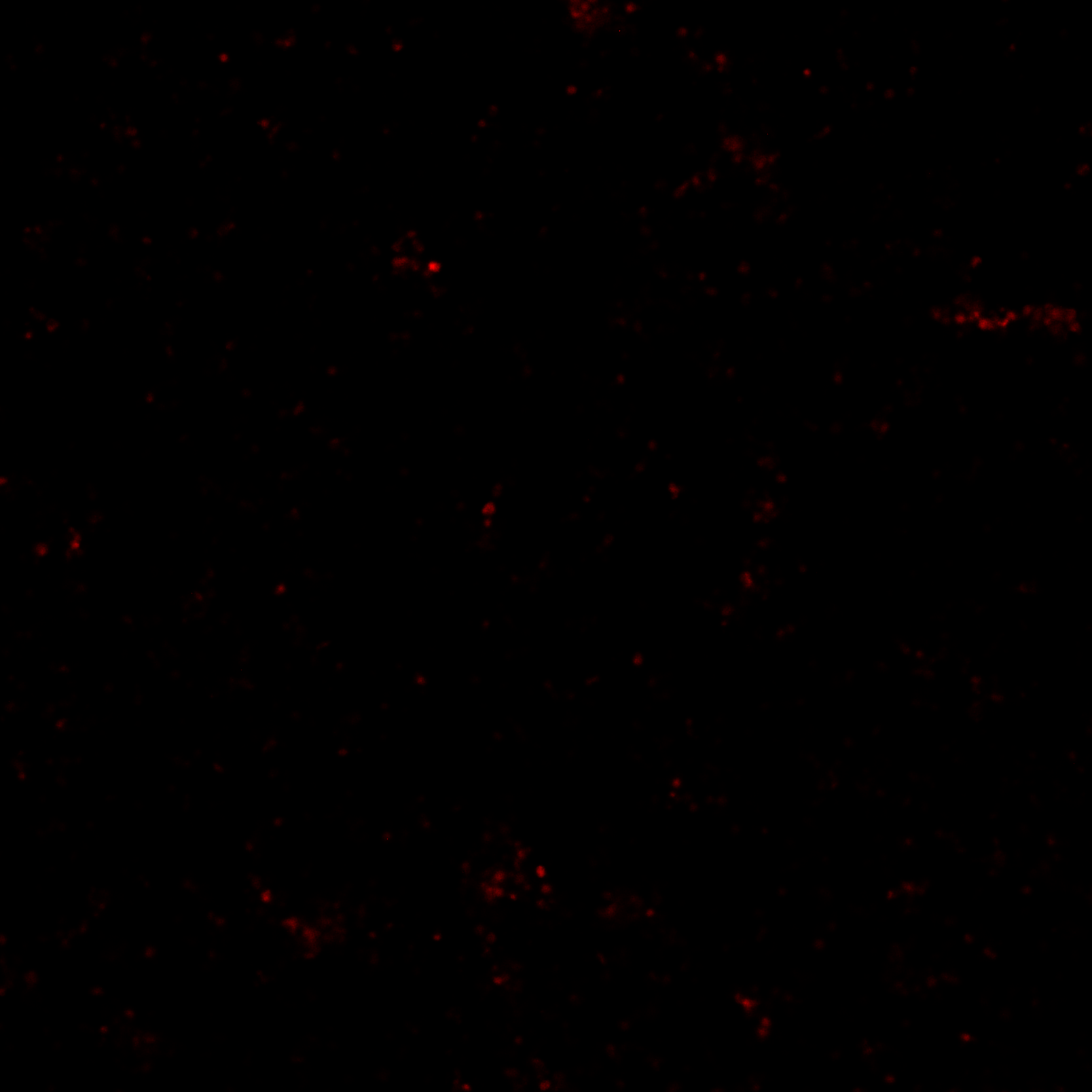

Supplement: S7 File — (ZIP) [file pone.0347758.s007.zip › cell ROS/PSB-H/H2_RGB.tif]

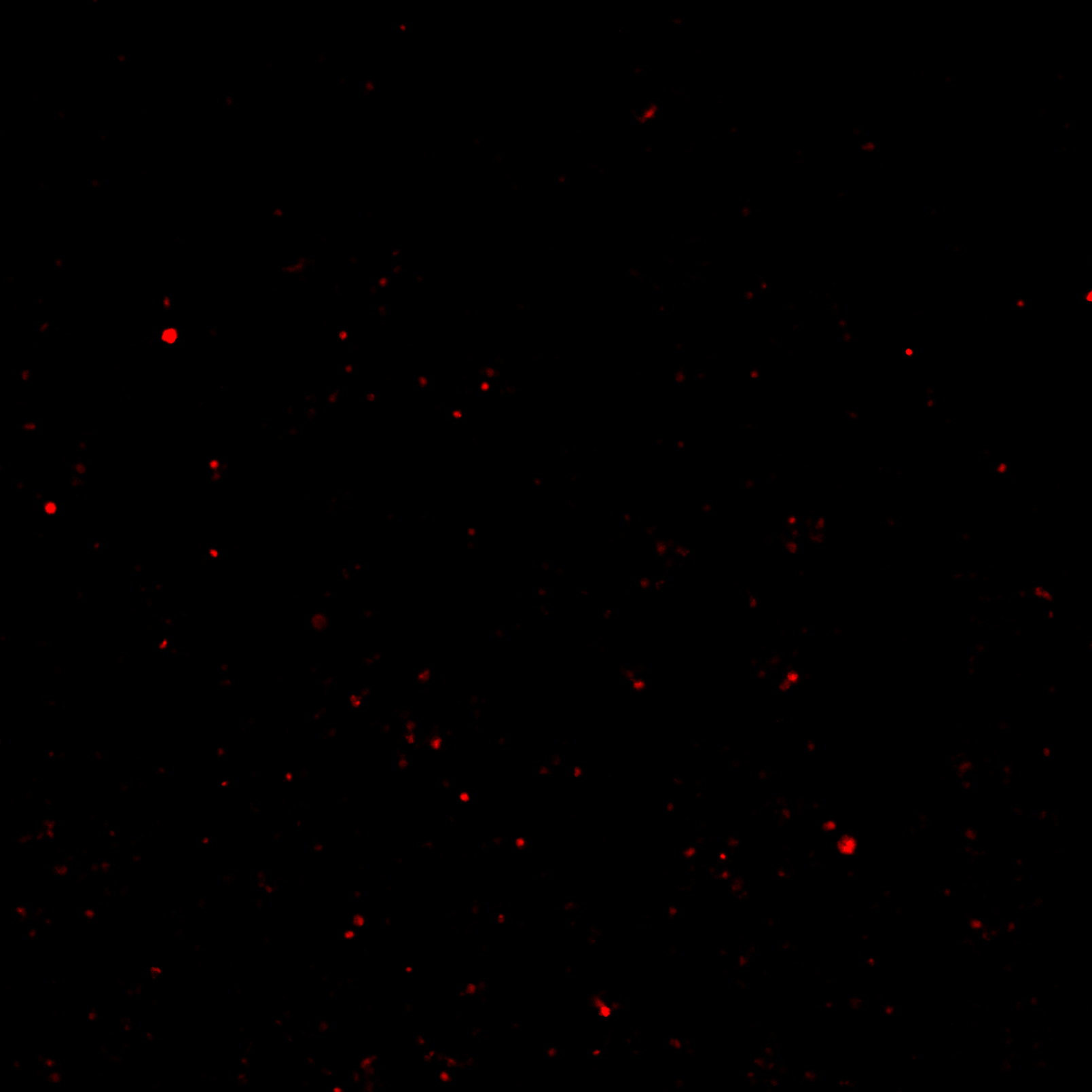

Supplement: S7 File — (ZIP) [file pone.0347758.s007.zip › cell ROS/PSB-H/H3_RGB.tif]

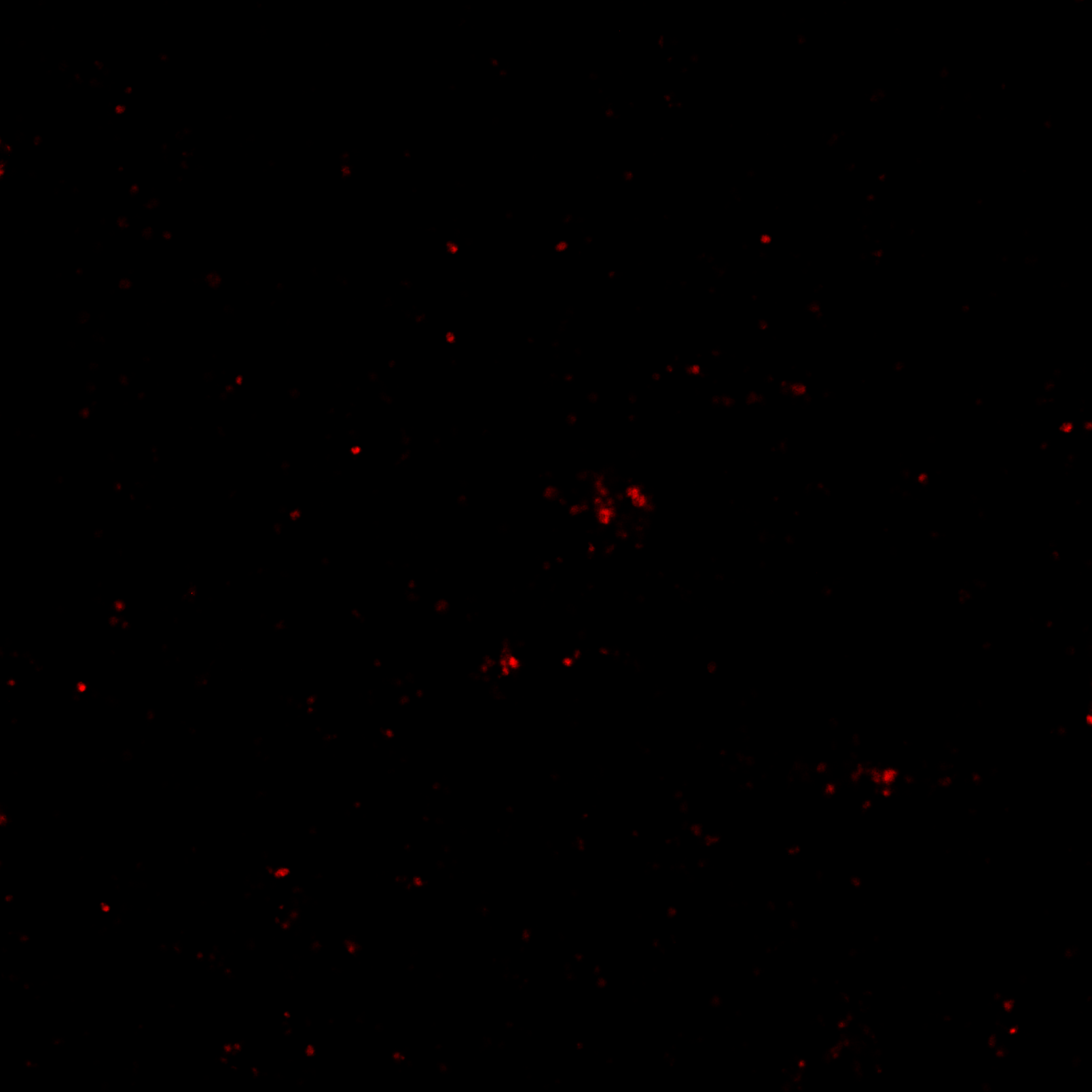

Supplement: S7 File — (ZIP) [file pone.0347758.s007.zip › cell ROS/PSB-H/H4_RGB.tif]

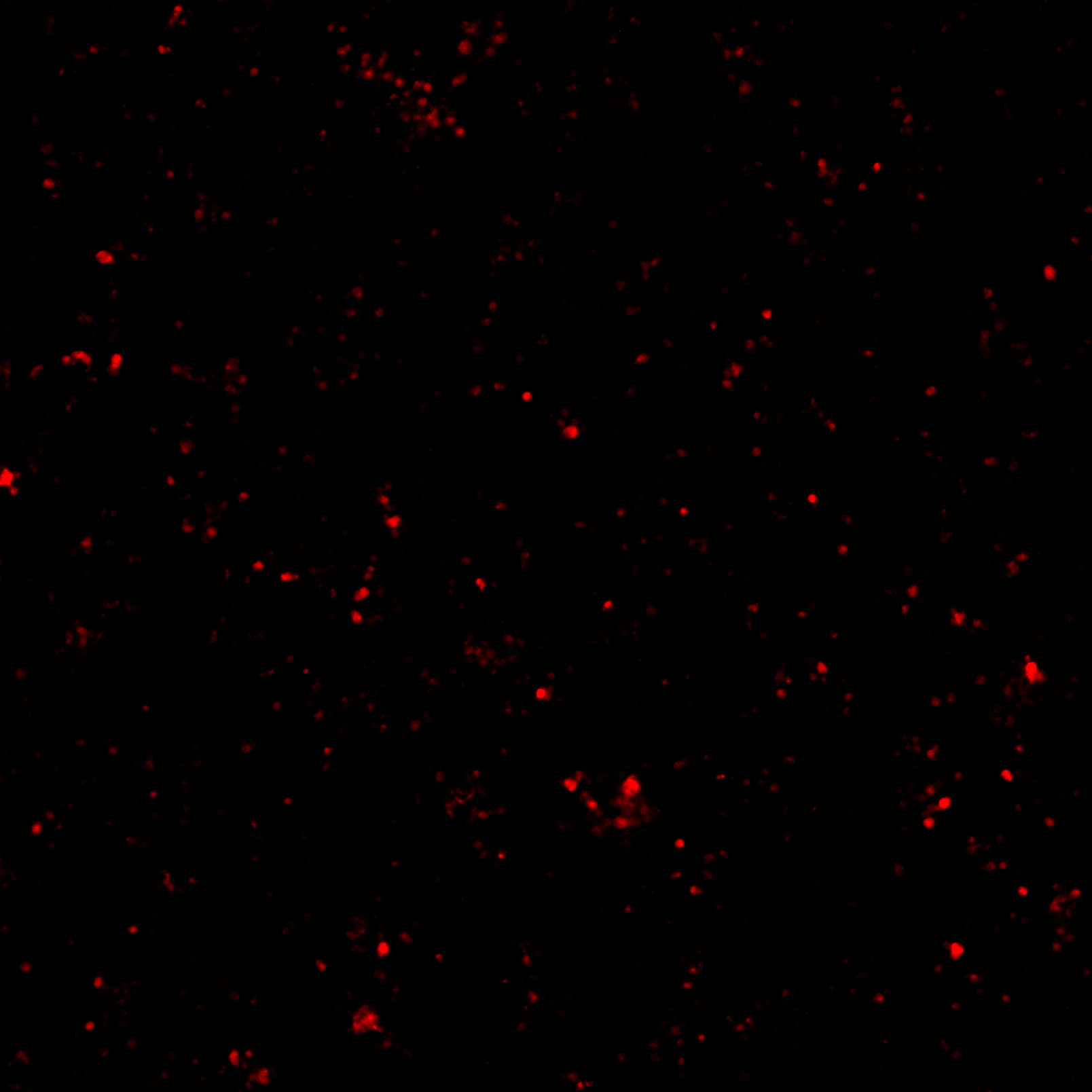

Supplement: S7 File — (ZIP) [file pone.0347758.s007.zip › cell ROS/PSB-L/L4_RGB.tif]

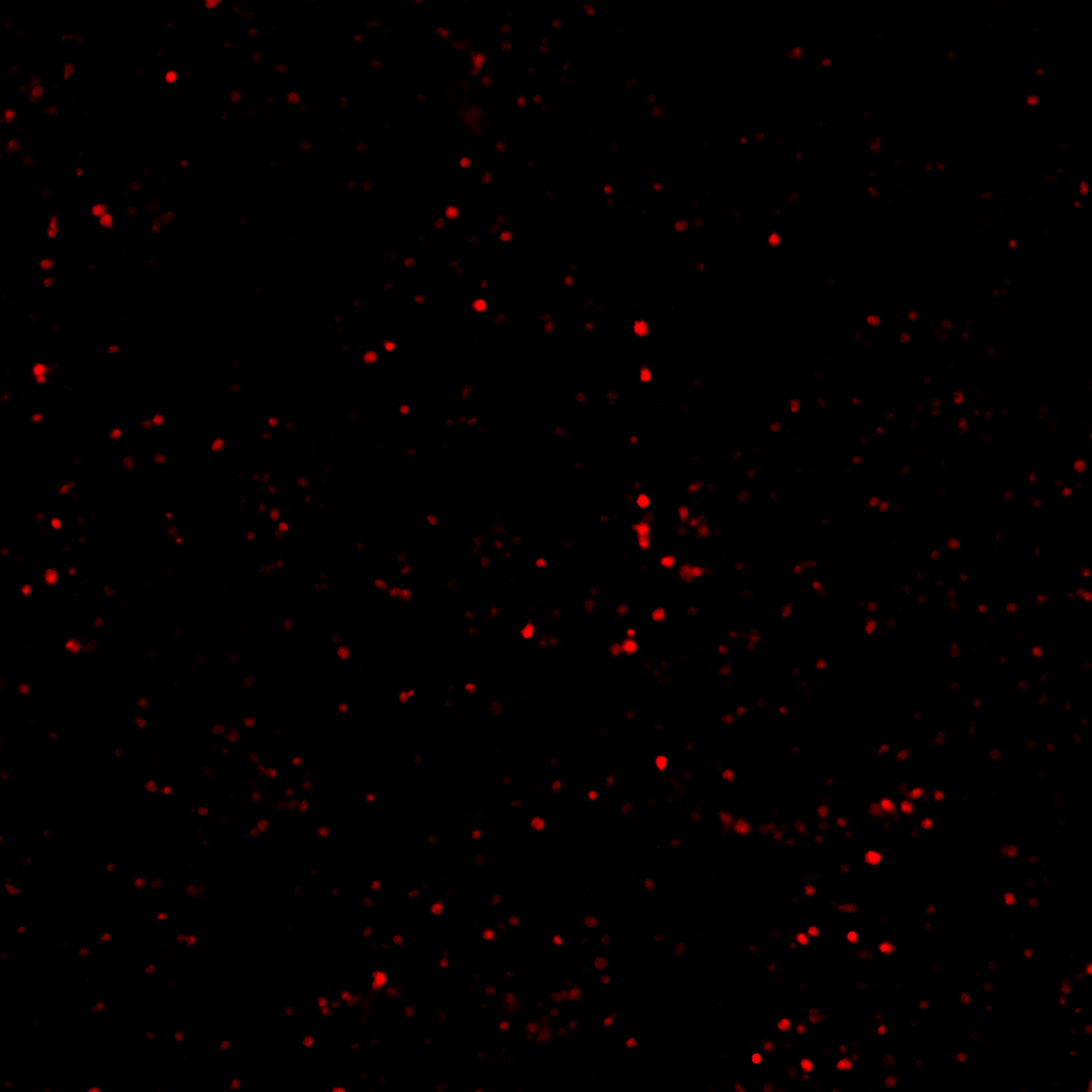

Supplement: S7 File — (ZIP) [file pone.0347758.s007.zip › cell ROS/PSB-L/L5_RGB.tif]

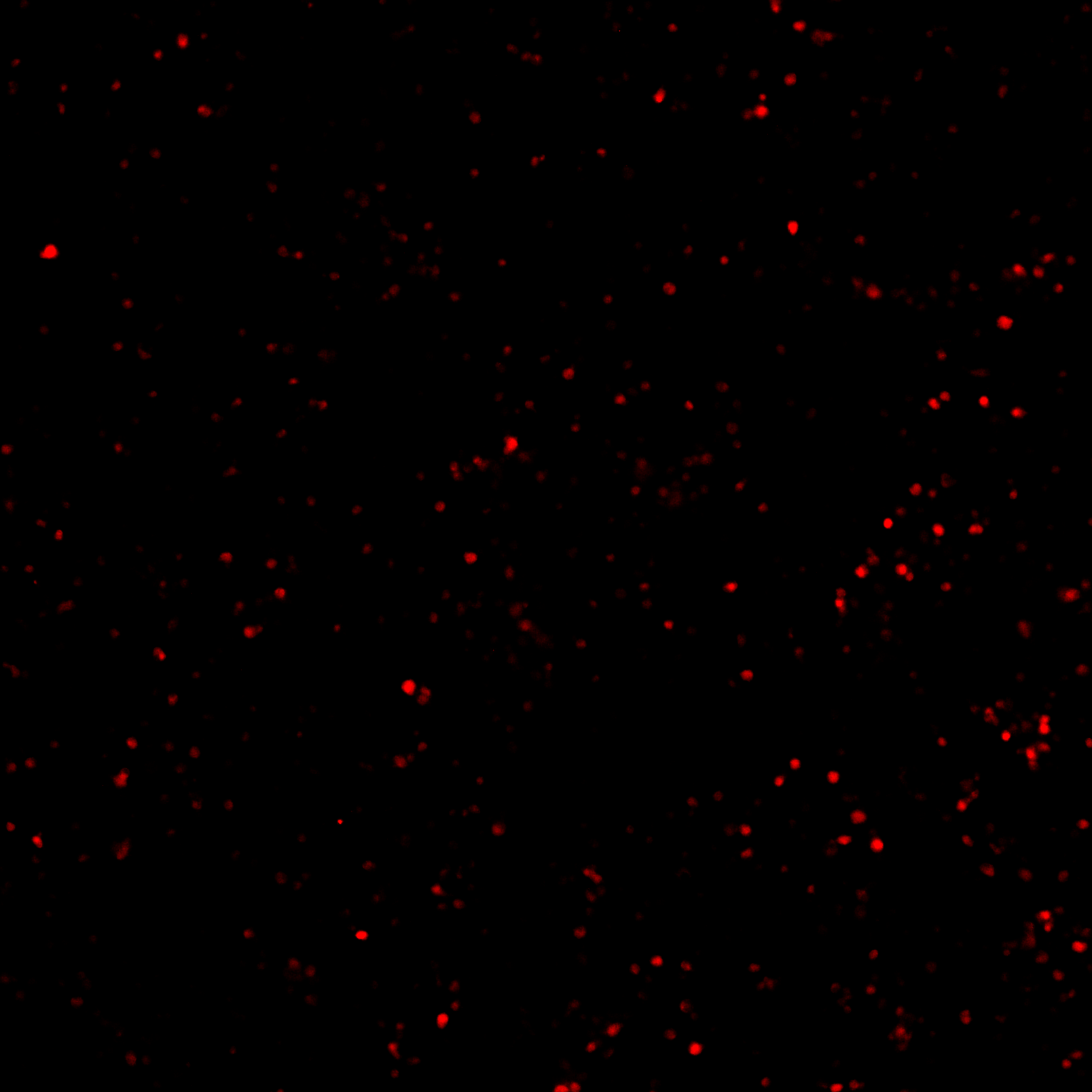

Supplement: S7 File — (ZIP) [file pone.0347758.s007.zip › cell ROS/PSB-L/L6_RGB.tif]

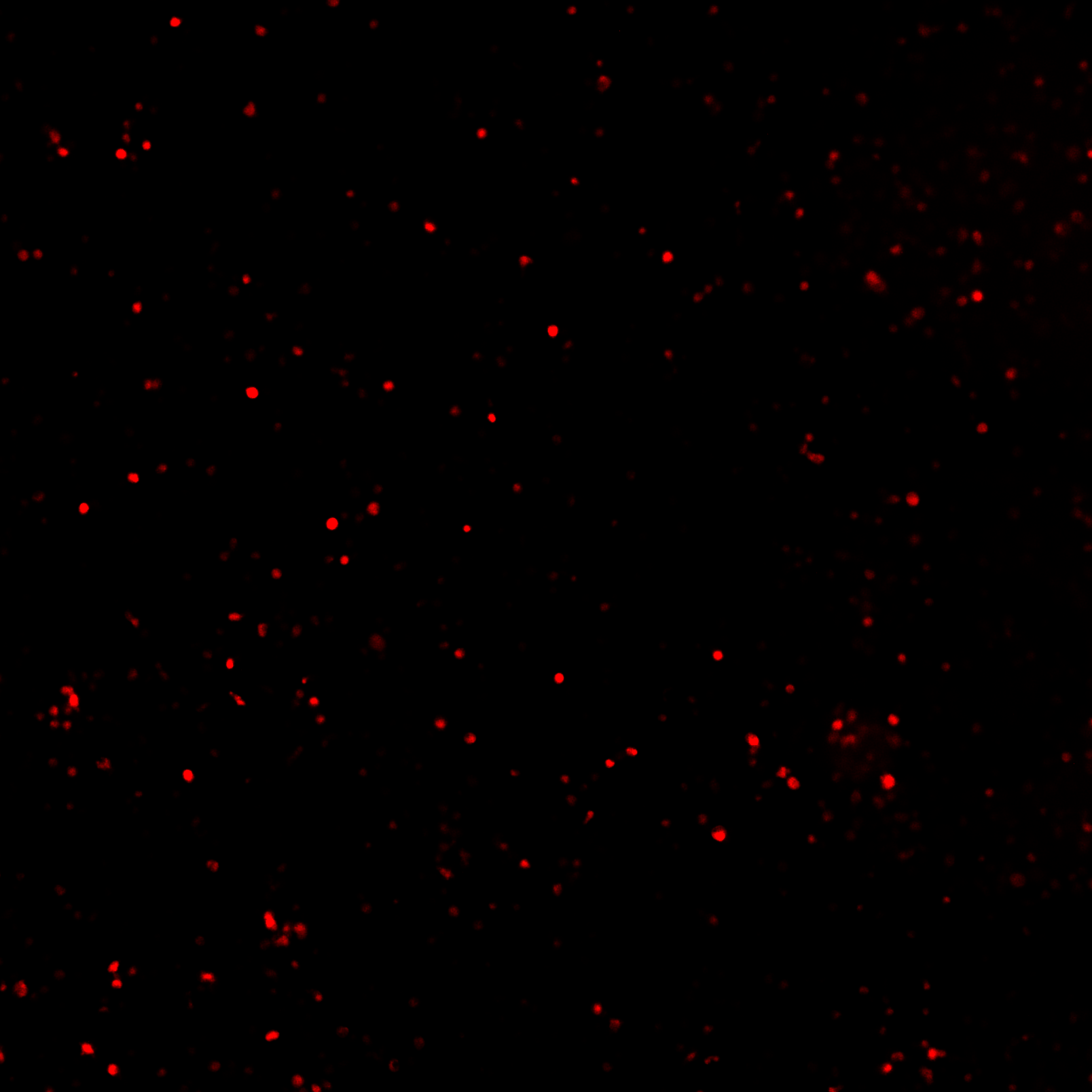

Supplement: S7 File — (ZIP) [file pone.0347758.s007.zip › cell ROS/PSB-M/MID4_RGB.tif]

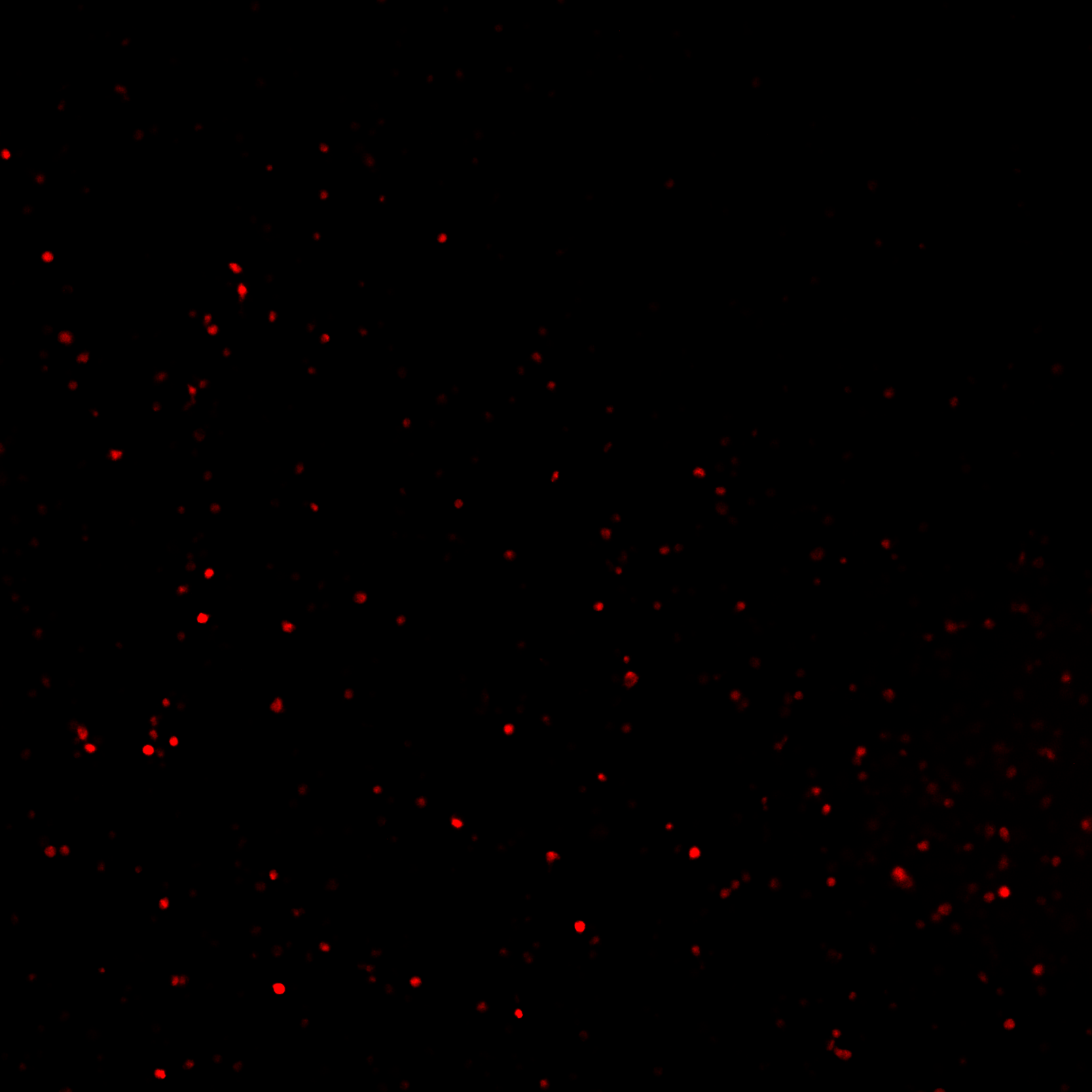

Supplement: S7 File — (ZIP) [file pone.0347758.s007.zip › cell ROS/PSB-M/MID5_RGB.tif]

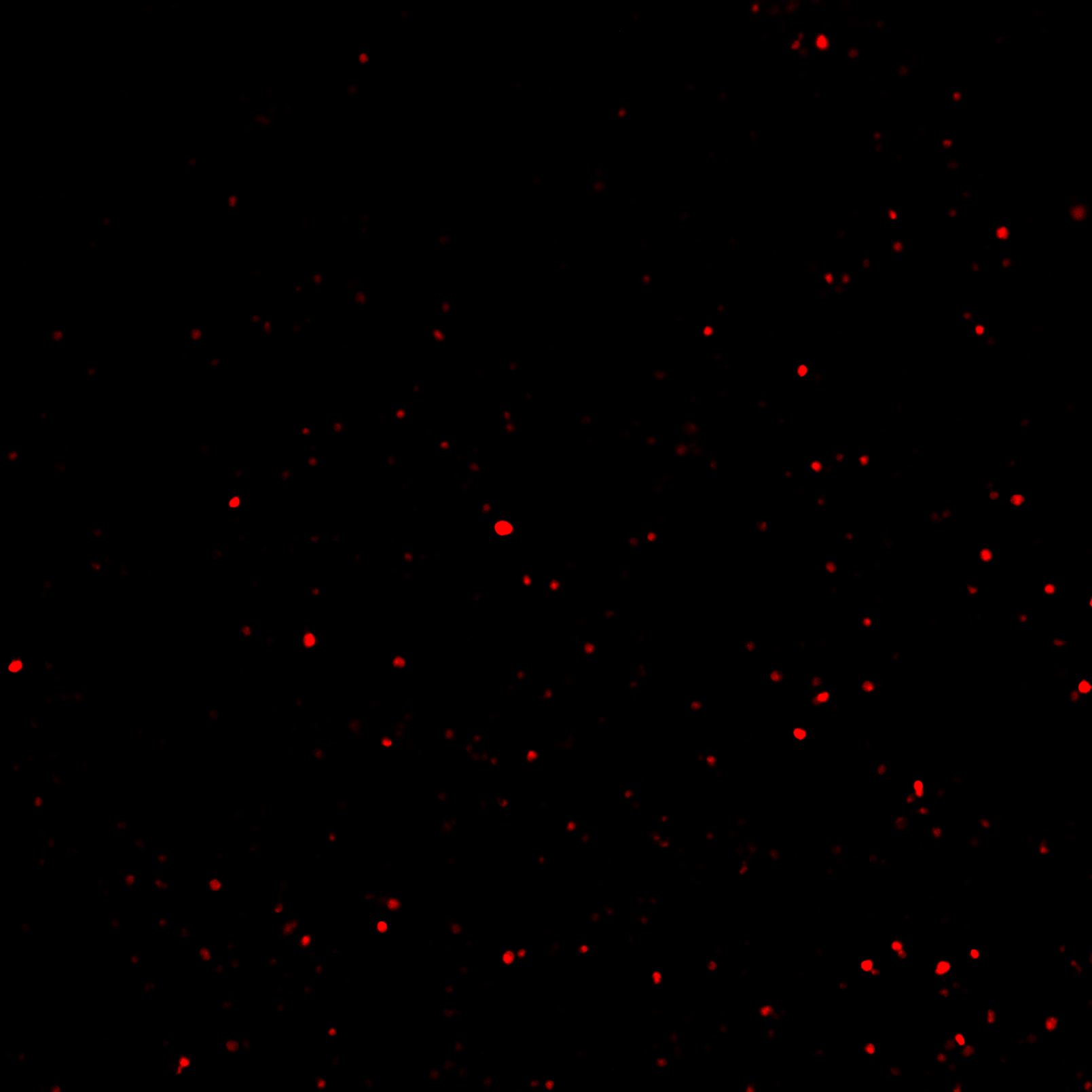

Supplement: S7 File — (ZIP) [file pone.0347758.s007.zip › cell ROS/PSB-M/MID6_RGB.tif]

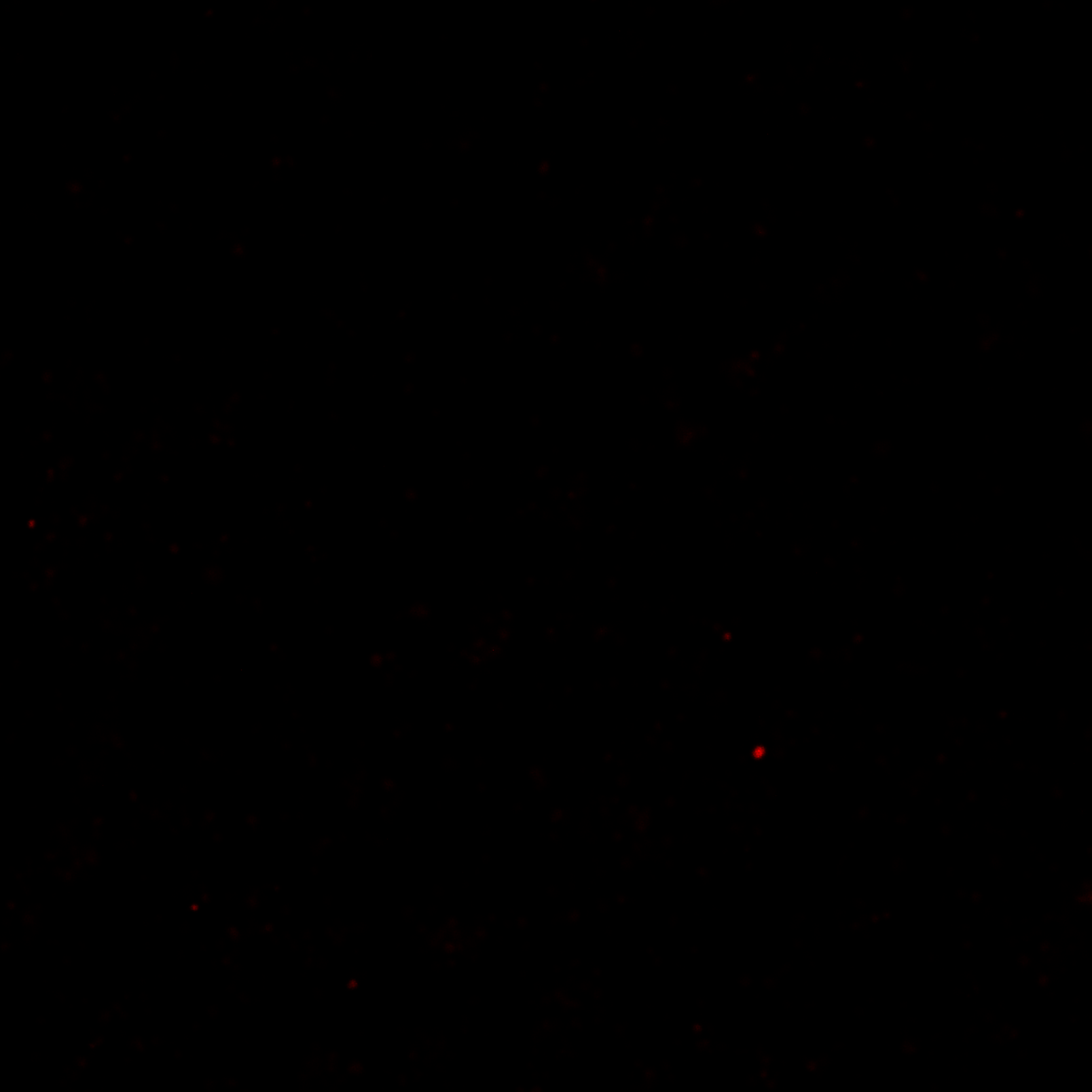

Supplement: S8 File — (ZIP) [file pone.0347758.s008.zip › cell ROS wiith ML inhibitor/control/blank-1.tif]

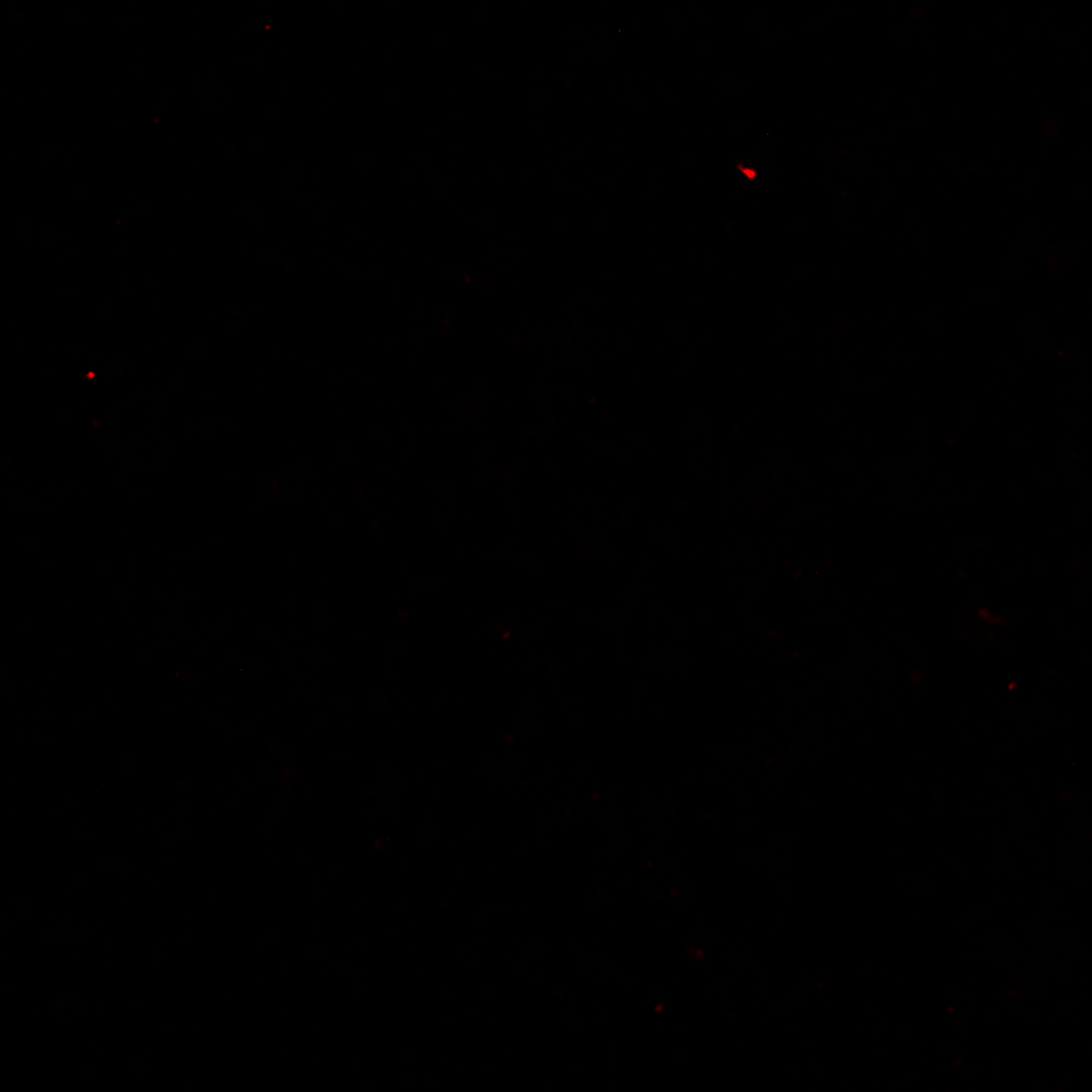

Supplement: S8 File — (ZIP) [file pone.0347758.s008.zip › cell ROS wiith ML inhibitor/control/blank-2.tif]

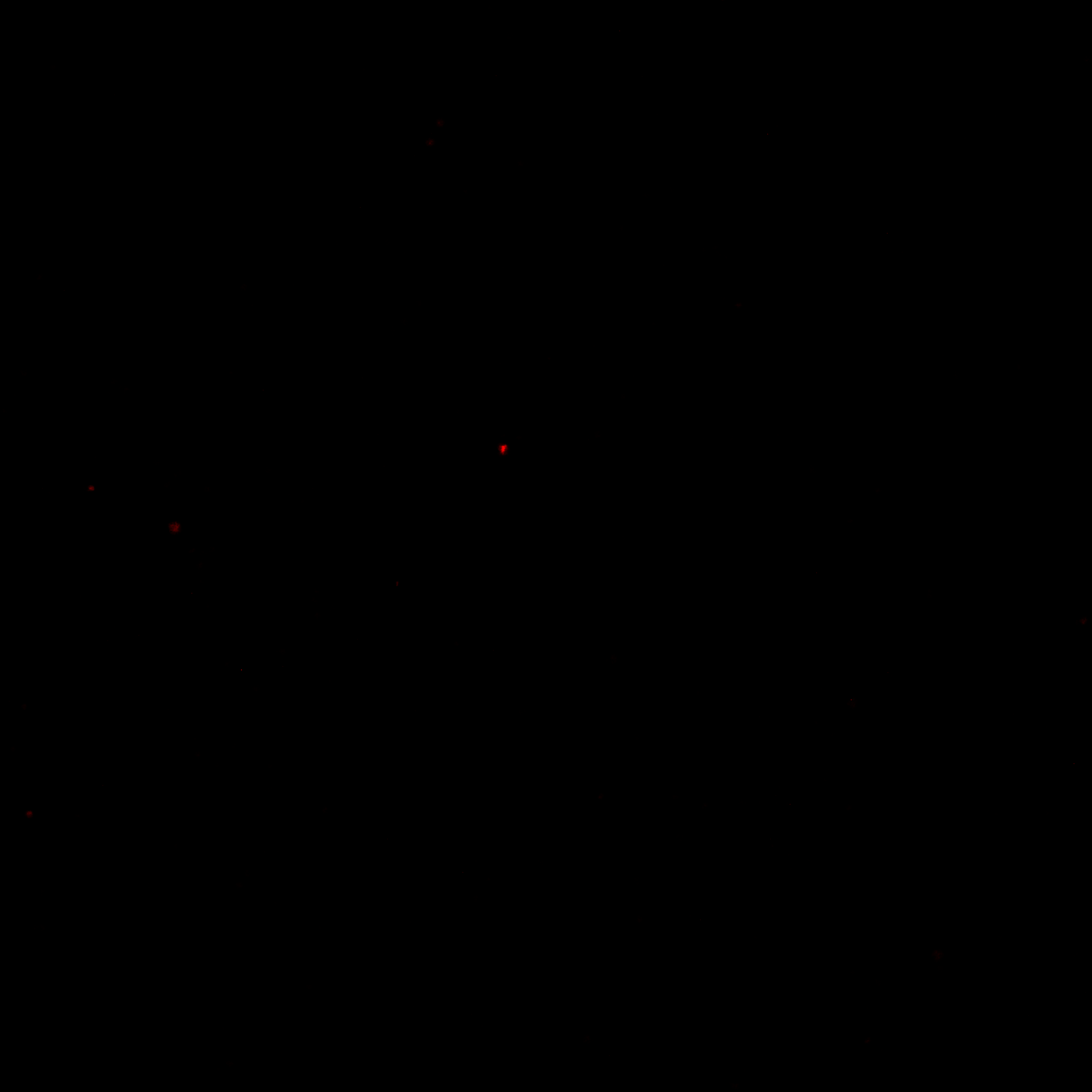

Supplement: S8 File — (ZIP) [file pone.0347758.s008.zip › cell ROS wiith ML inhibitor/control/blank-3.tif]

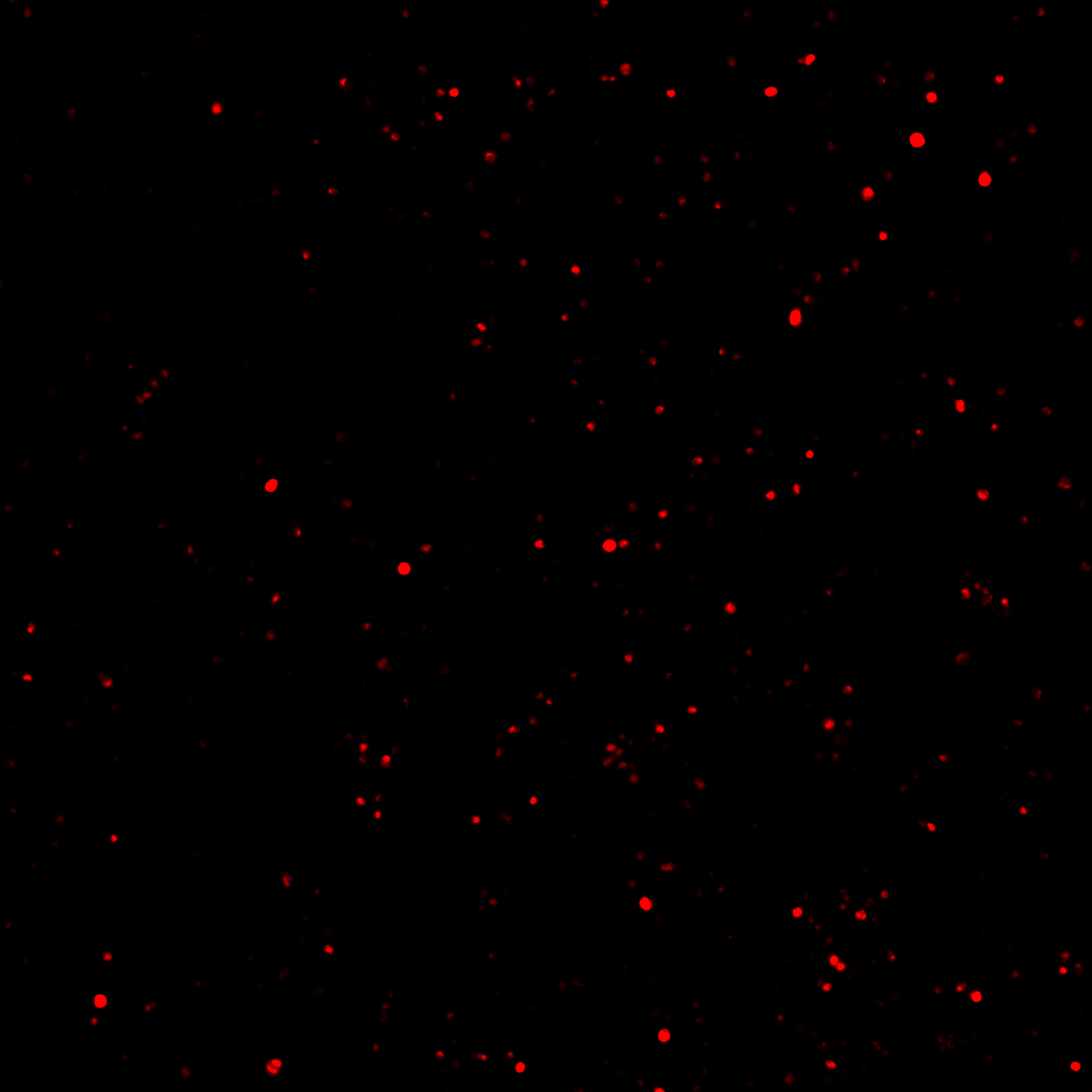

Supplement: S8 File — (ZIP) [file pone.0347758.s008.zip › cell ROS wiith ML inhibitor/ML/ML4_RGB.tif]

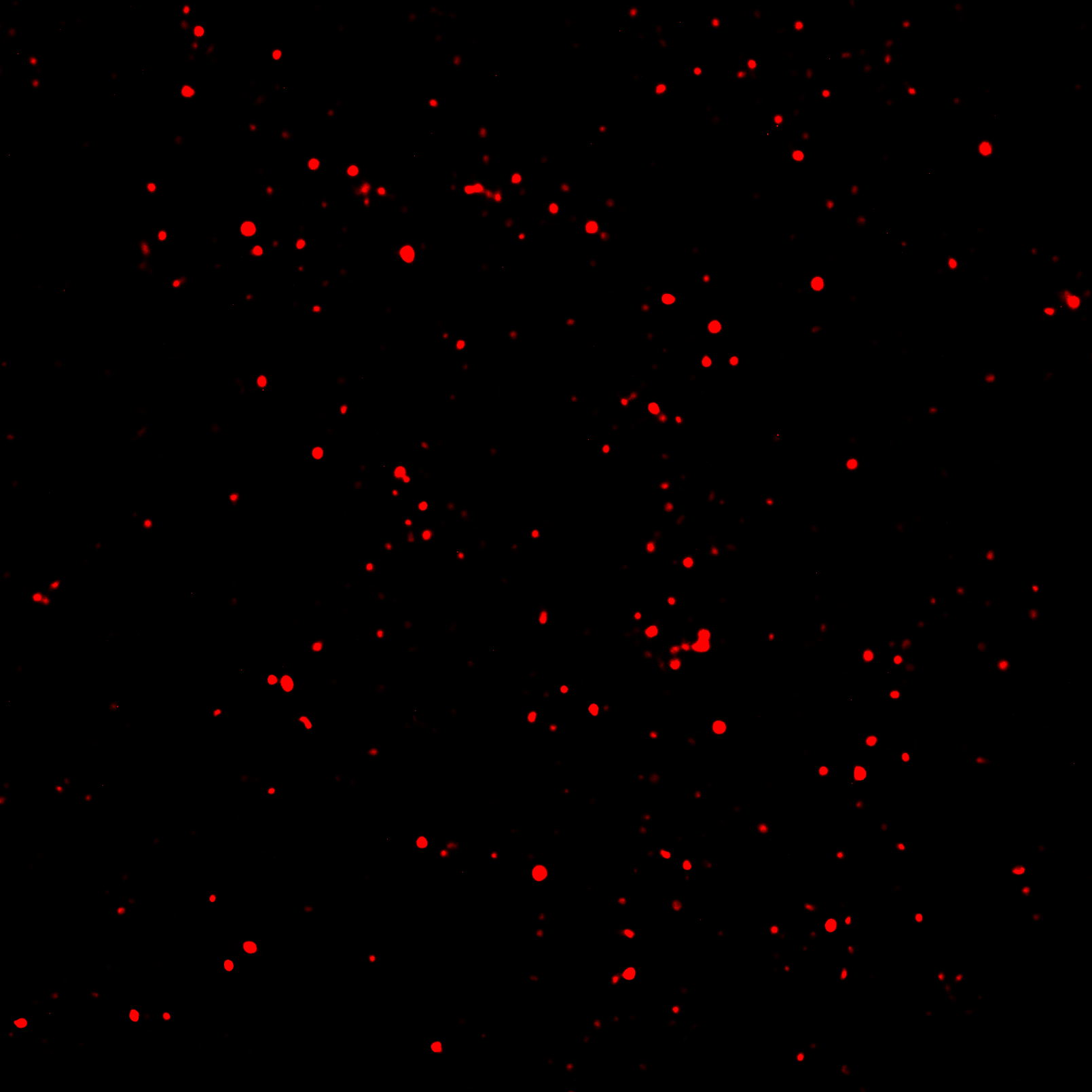

Supplement: S8 File — (ZIP) [file pone.0347758.s008.zip › cell ROS wiith ML inhibitor/ML/ML5_RGB.tif]

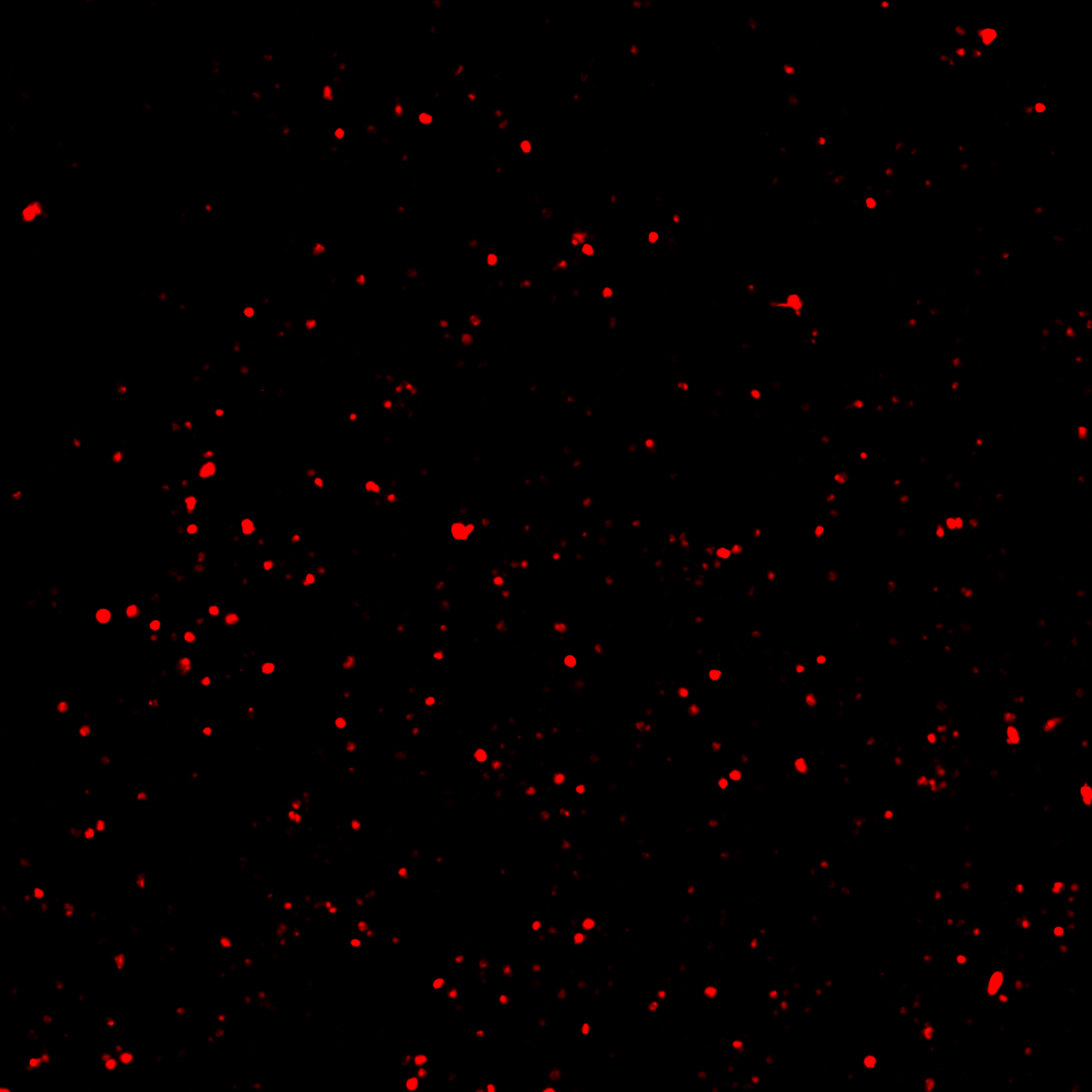

Supplement: S8 File — (ZIP) [file pone.0347758.s008.zip › cell ROS wiith ML inhibitor/ML/ML6_RGB.tif]

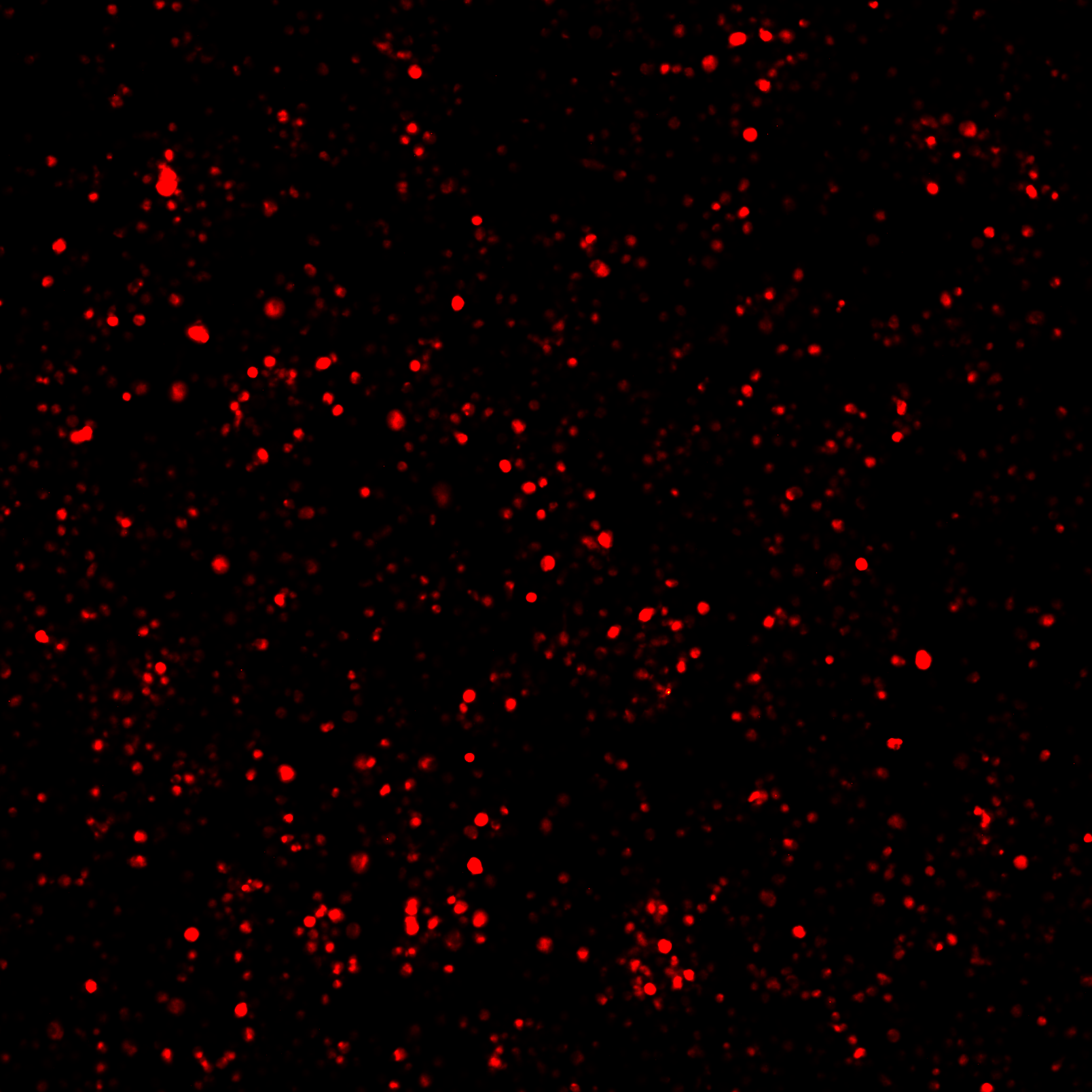

Supplement: S8 File — (ZIP) [file pone.0347758.s008.zip › cell ROS wiith ML inhibitor/ox-LDL/M1_RGB.tif]

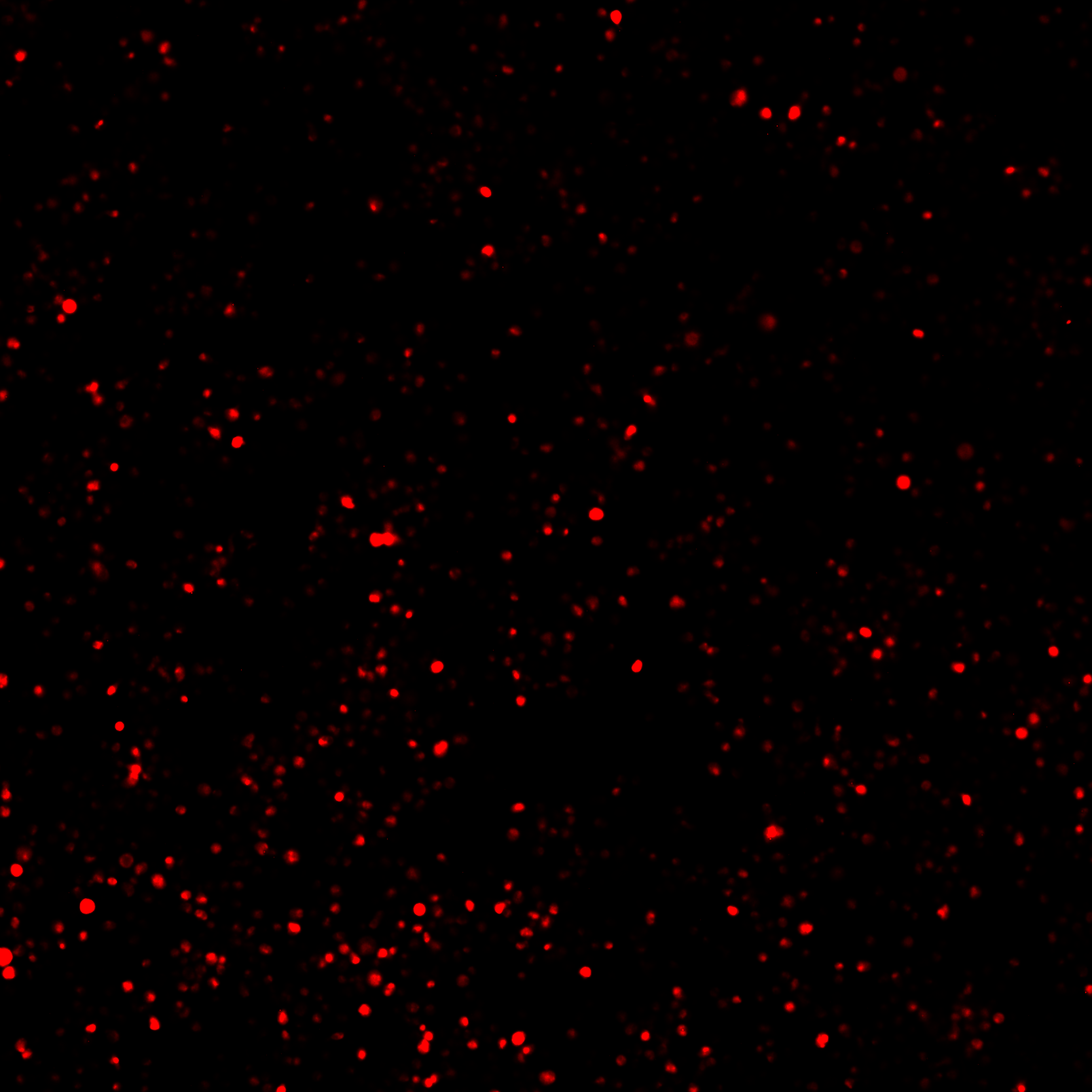

Supplement: S8 File — (ZIP) [file pone.0347758.s008.zip › cell ROS wiith ML inhibitor/ox-LDL/M2_RGB.tif]

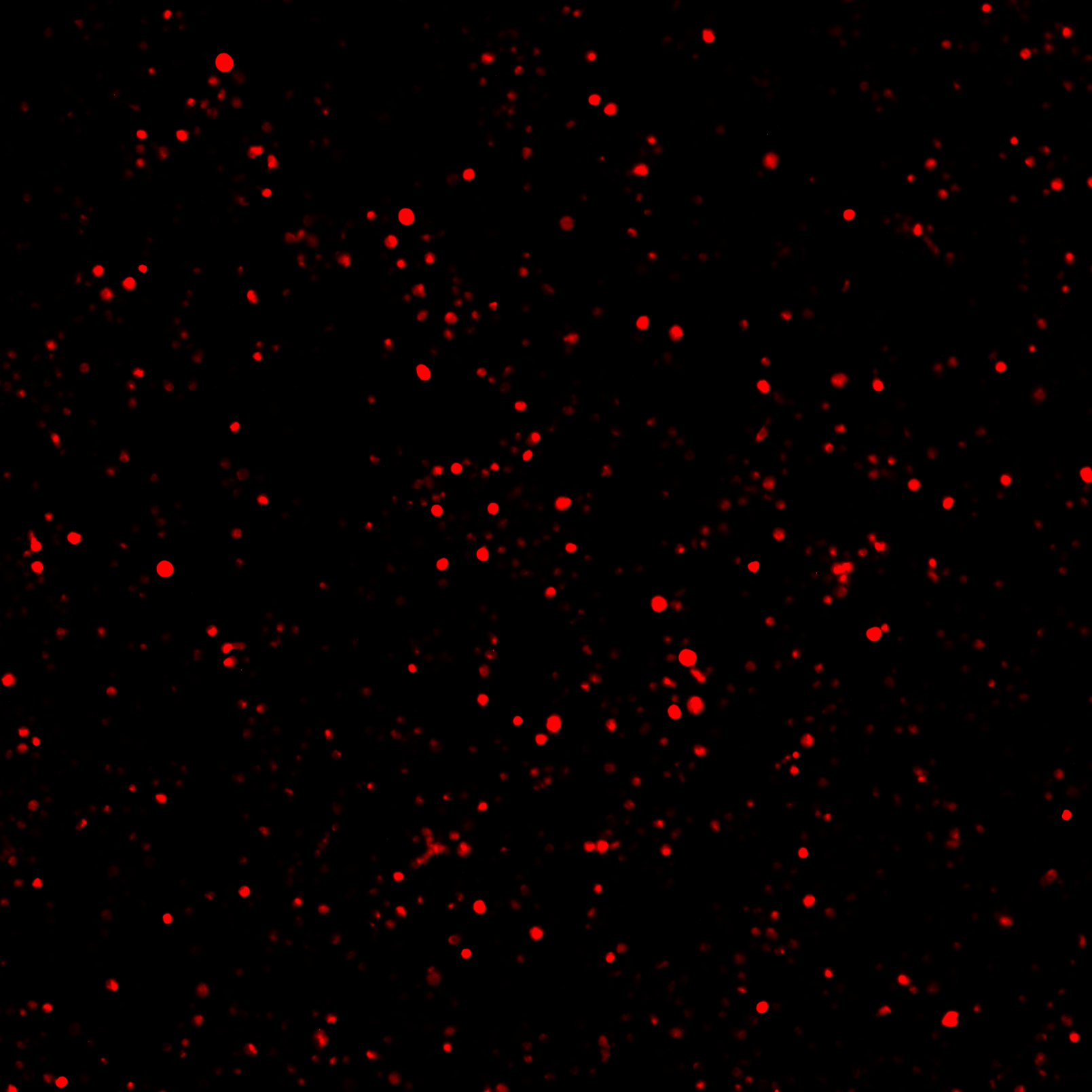

Supplement: S8 File — (ZIP) [file pone.0347758.s008.zip › cell ROS wiith ML inhibitor/ox-LDL/M3_RGB.tif]

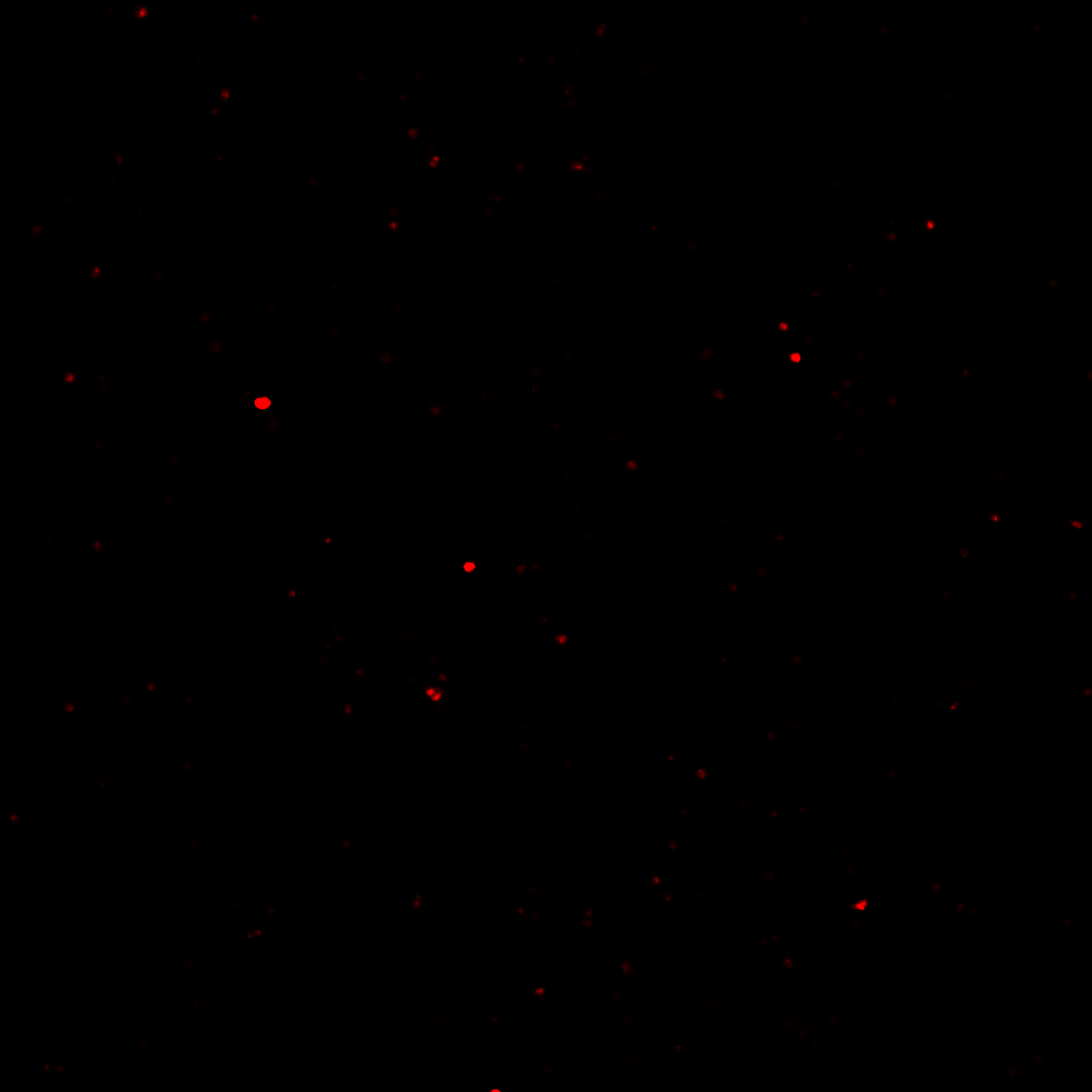

Supplement: S8 File — (ZIP) [file pone.0347758.s008.zip › cell ROS wiith ML inhibitor/PSB-H/PT-2_RGB_RGB.tif]

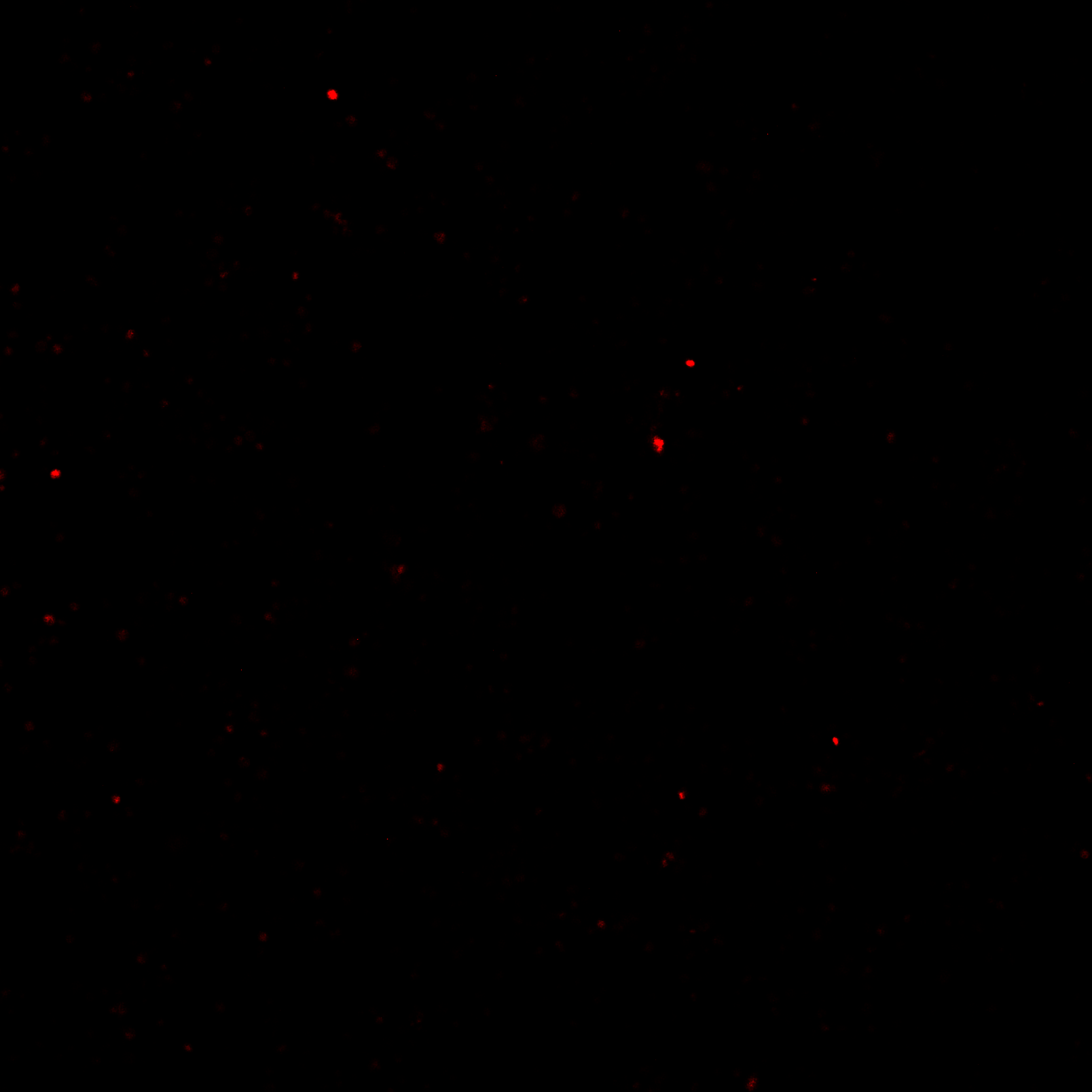

Supplement: S8 File — (ZIP) [file pone.0347758.s008.zip › cell ROS wiith ML inhibitor/PSB-H/PT-3_RGB_RGB_RGB.tif]

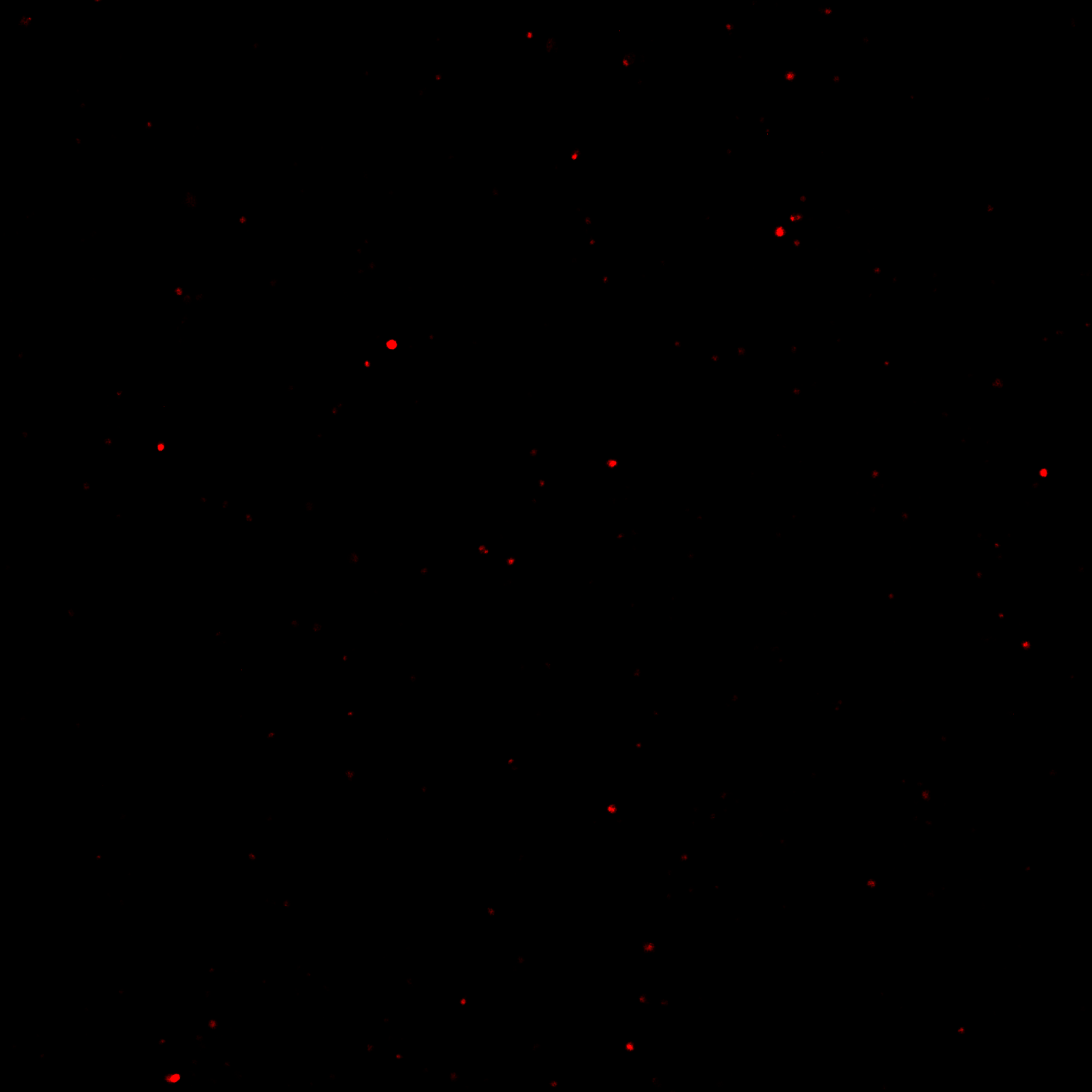

Supplement: S8 File — (ZIP) [file pone.0347758.s008.zip › cell ROS wiith ML inhibitor/PSB-H/PT1_RGB.tif]

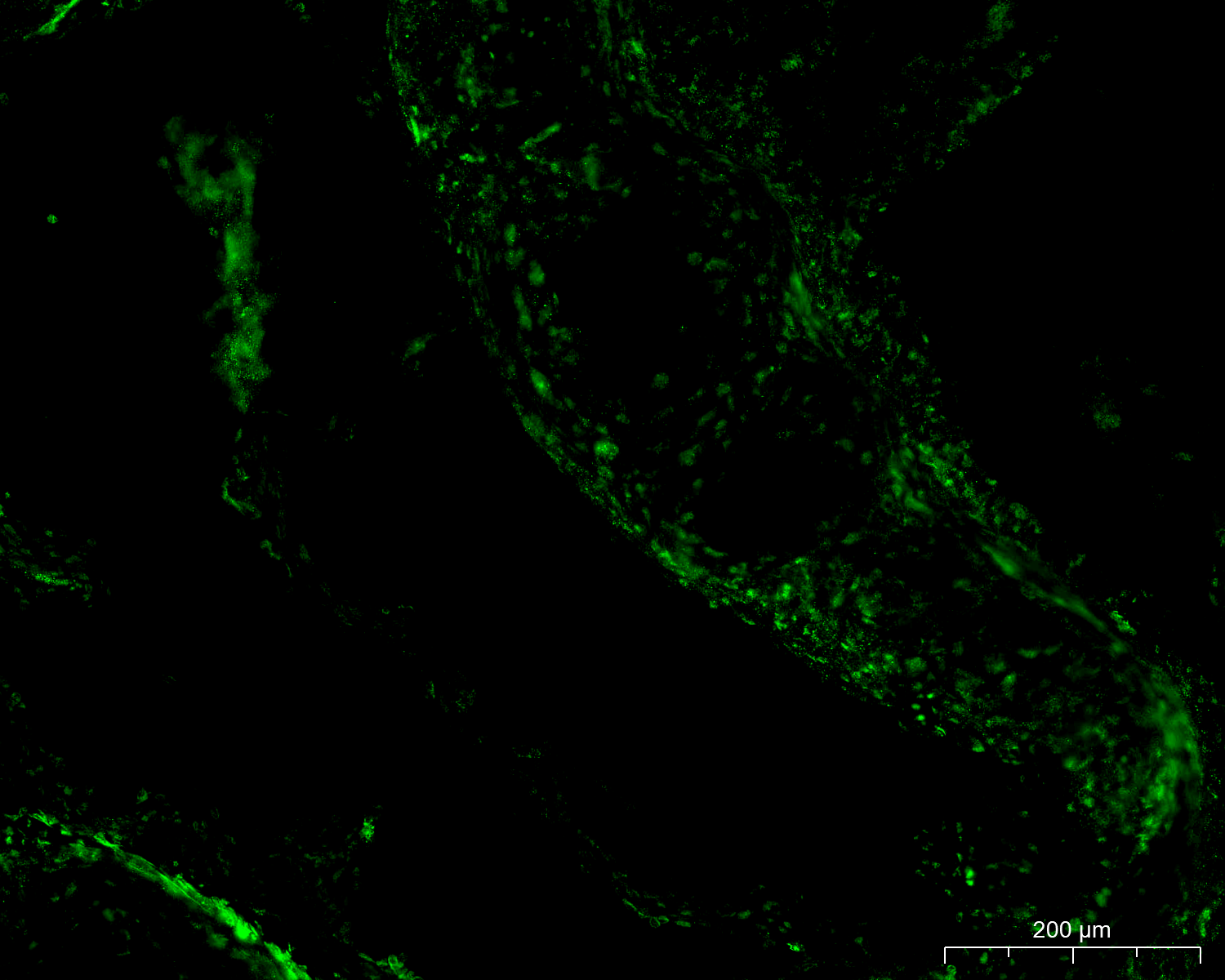

Supplement: S9 File — (ZIP) [file pone.0347758.s009.zip › 主动脉CD36/CD36/AS/23 CD36绿_20.0x.tif]

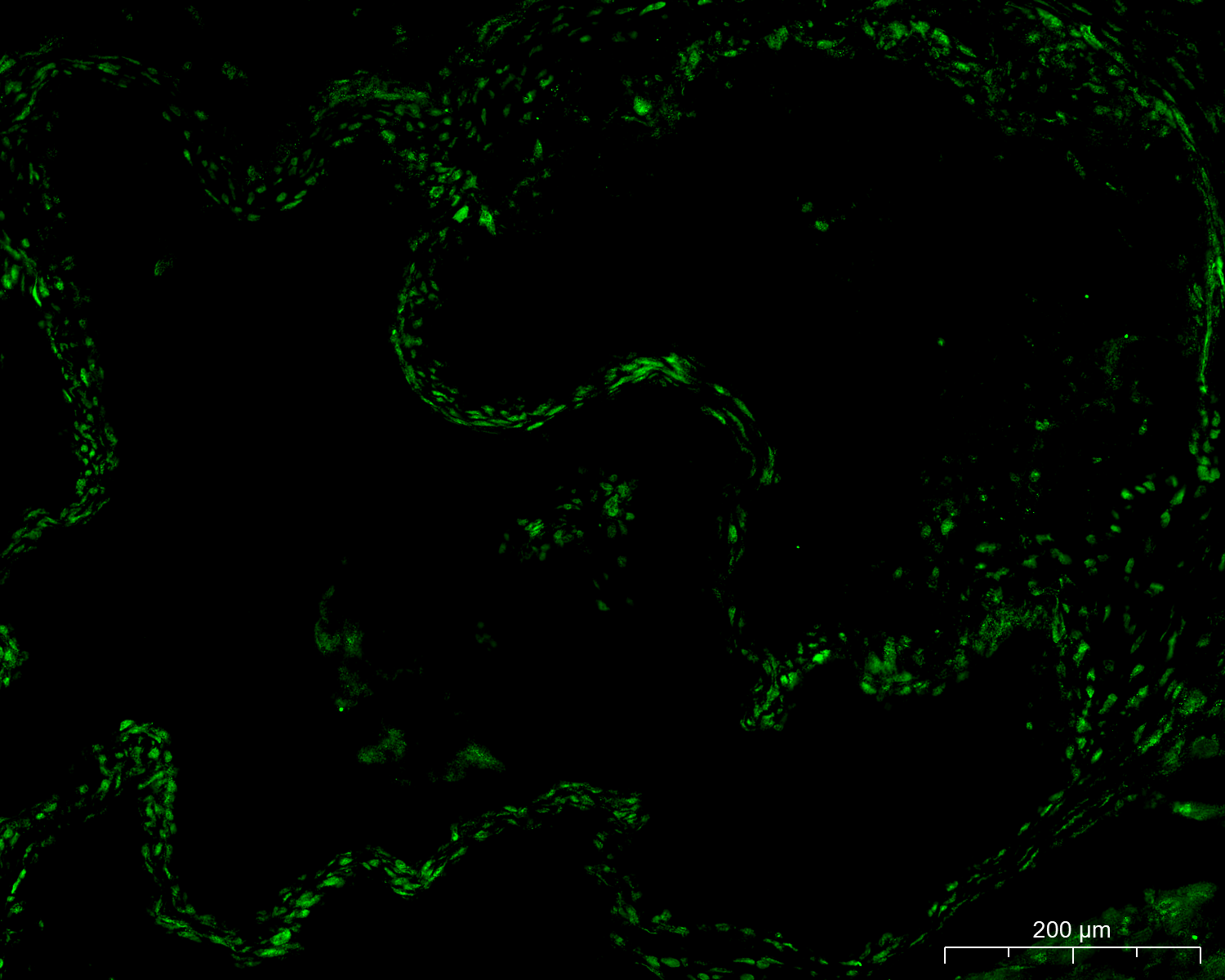

Supplement: S9 File — (ZIP) [file pone.0347758.s009.zip › 主动脉CD36/CD36/AS/27 CD36绿_20.0x.tif]

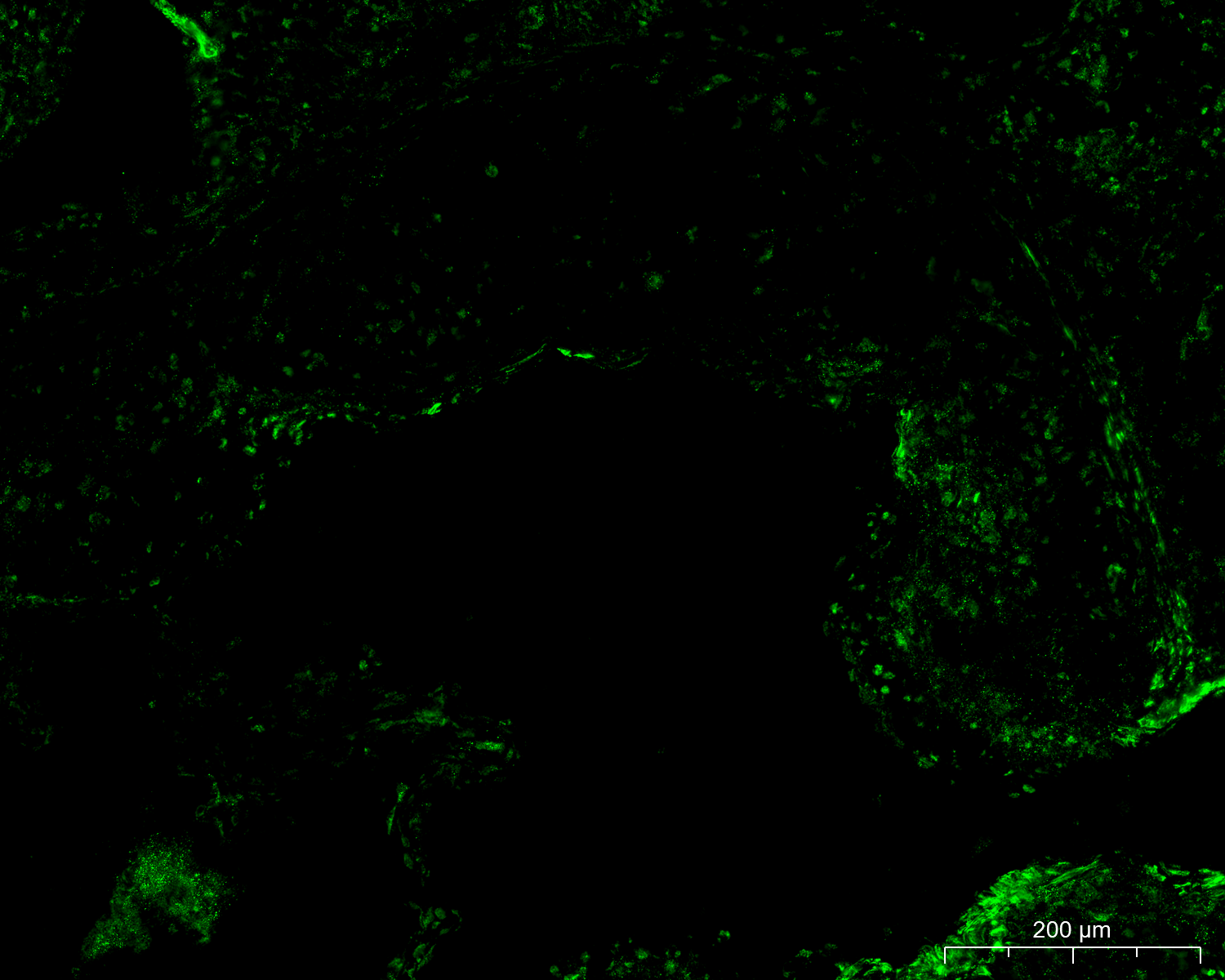

Supplement: S9 File — (ZIP) [file pone.0347758.s009.zip › 主动脉CD36/CD36/AS/28 CD36绿_20.0x.tif]

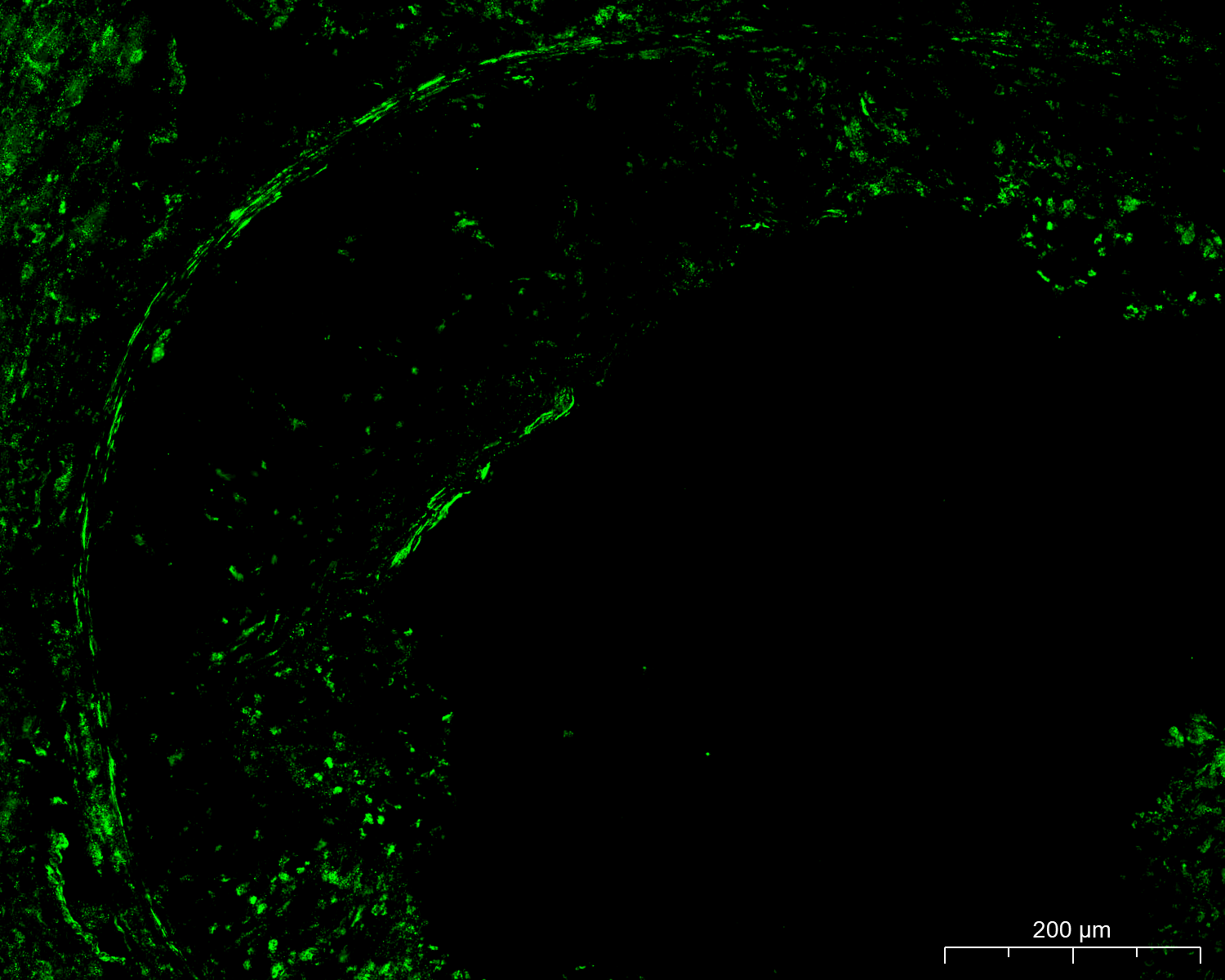

Supplement: S9 File — (ZIP) [file pone.0347758.s009.zip › 主动脉CD36/CD36/AS/31 CD36绿_20.0x.tif]

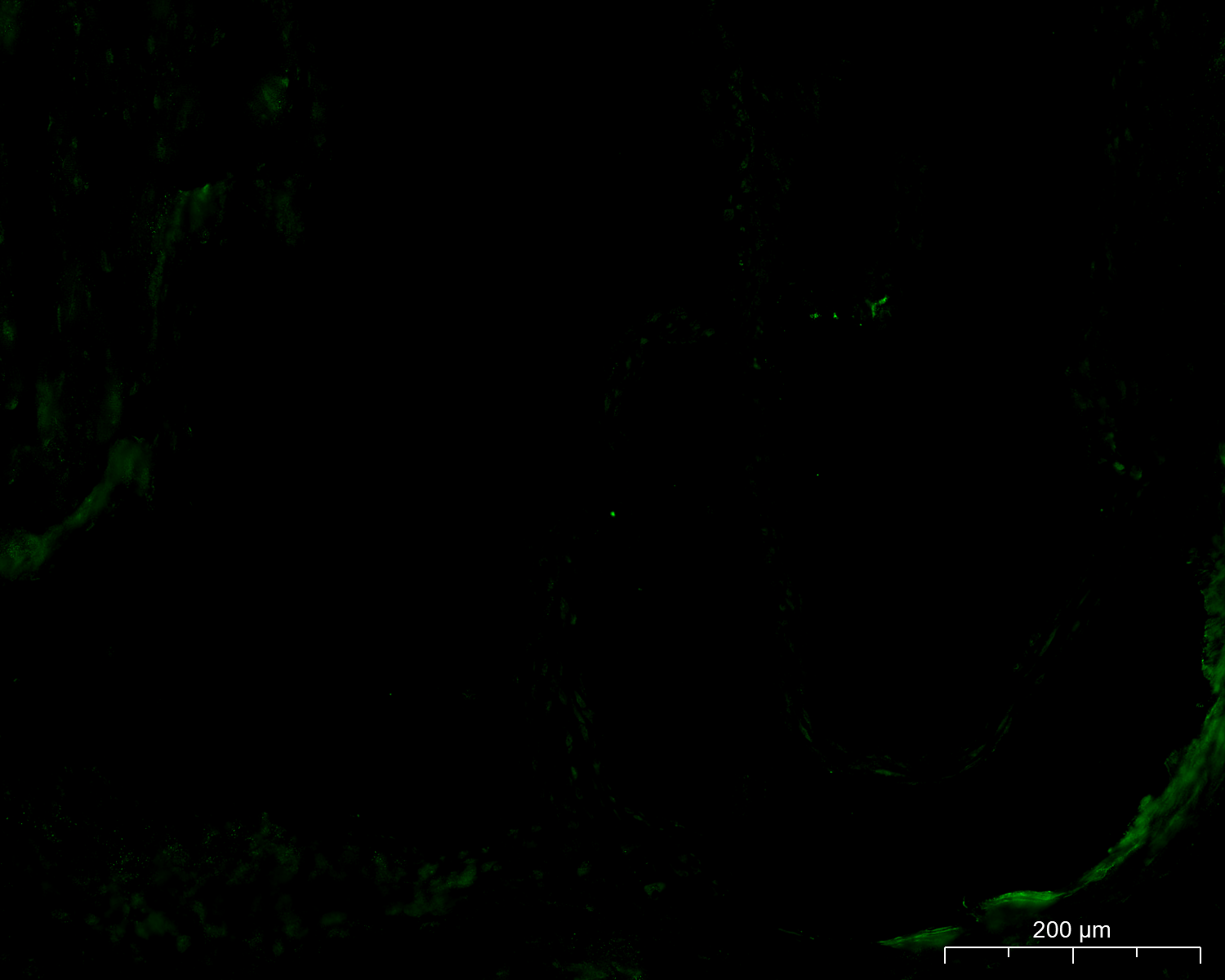

Supplement: S9 File — (ZIP) [file pone.0347758.s009.zip › 主动脉CD36/CD36/control/1 CD36绿_20.0x.tif]

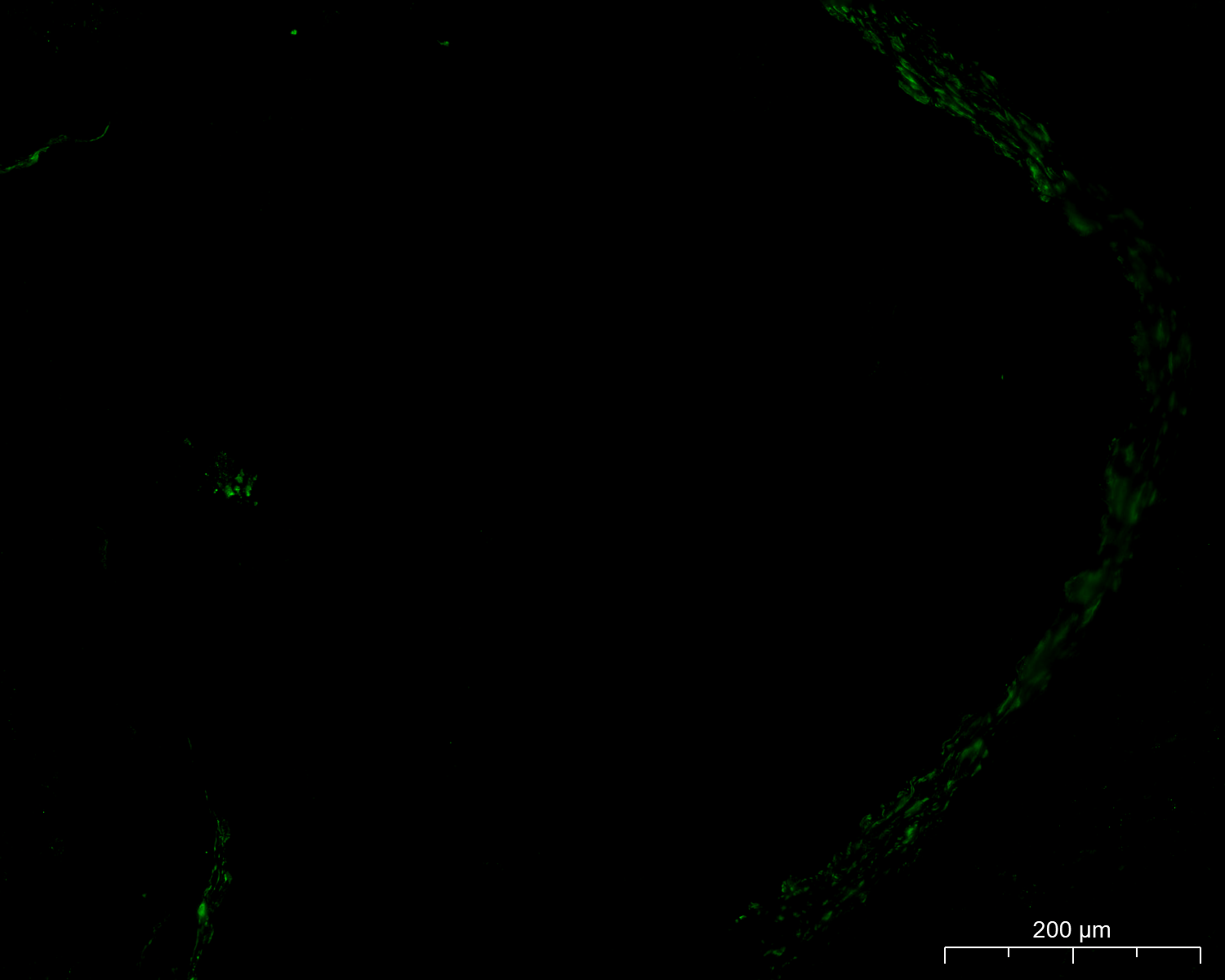

Supplement: S9 File — (ZIP) [file pone.0347758.s009.zip › 主动脉CD36/CD36/control/2 CD36绿_20.0x.tif]

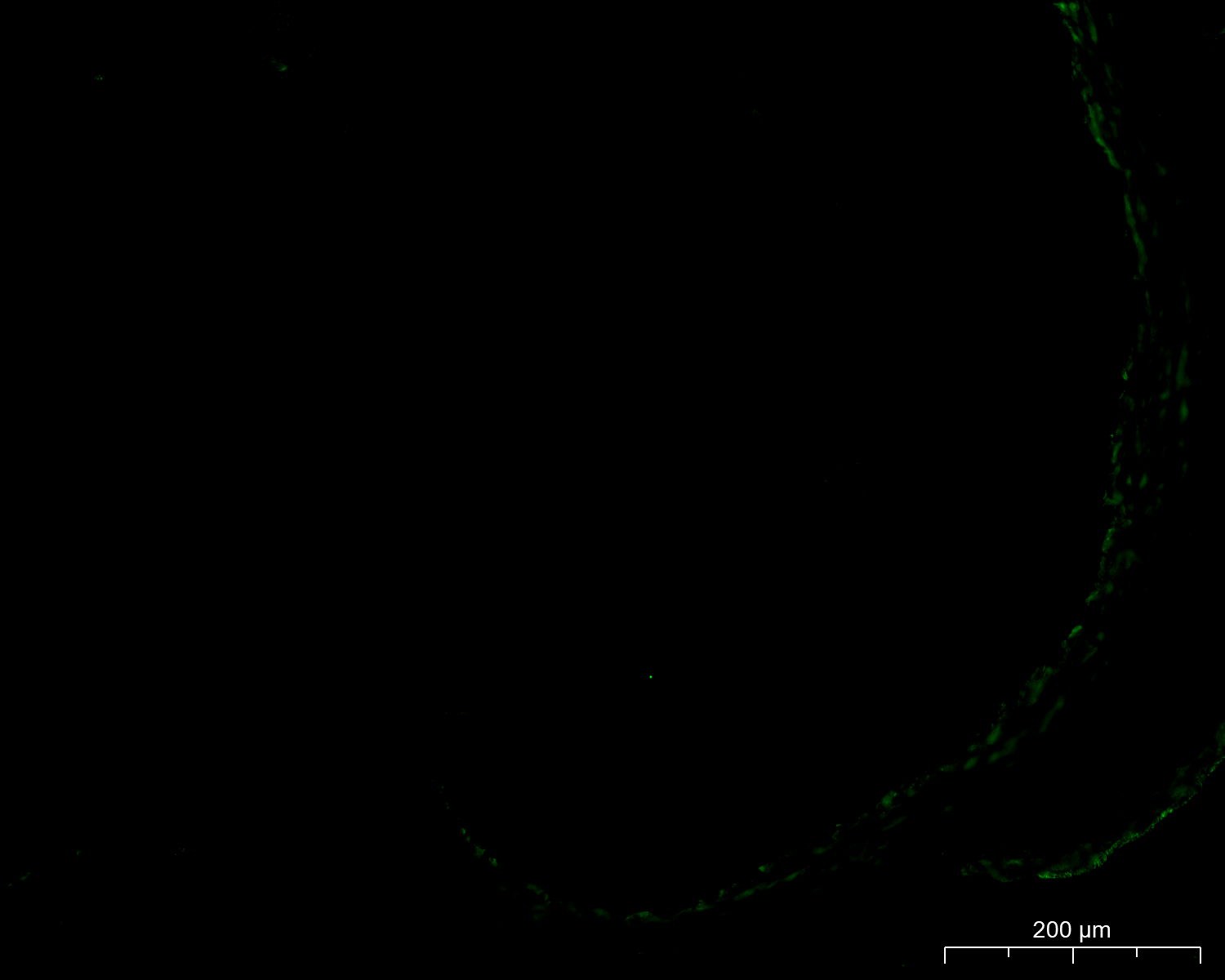

Supplement: S9 File — (ZIP) [file pone.0347758.s009.zip › 主动脉CD36/CD36/control/6 CD36绿_20.0x.tif]

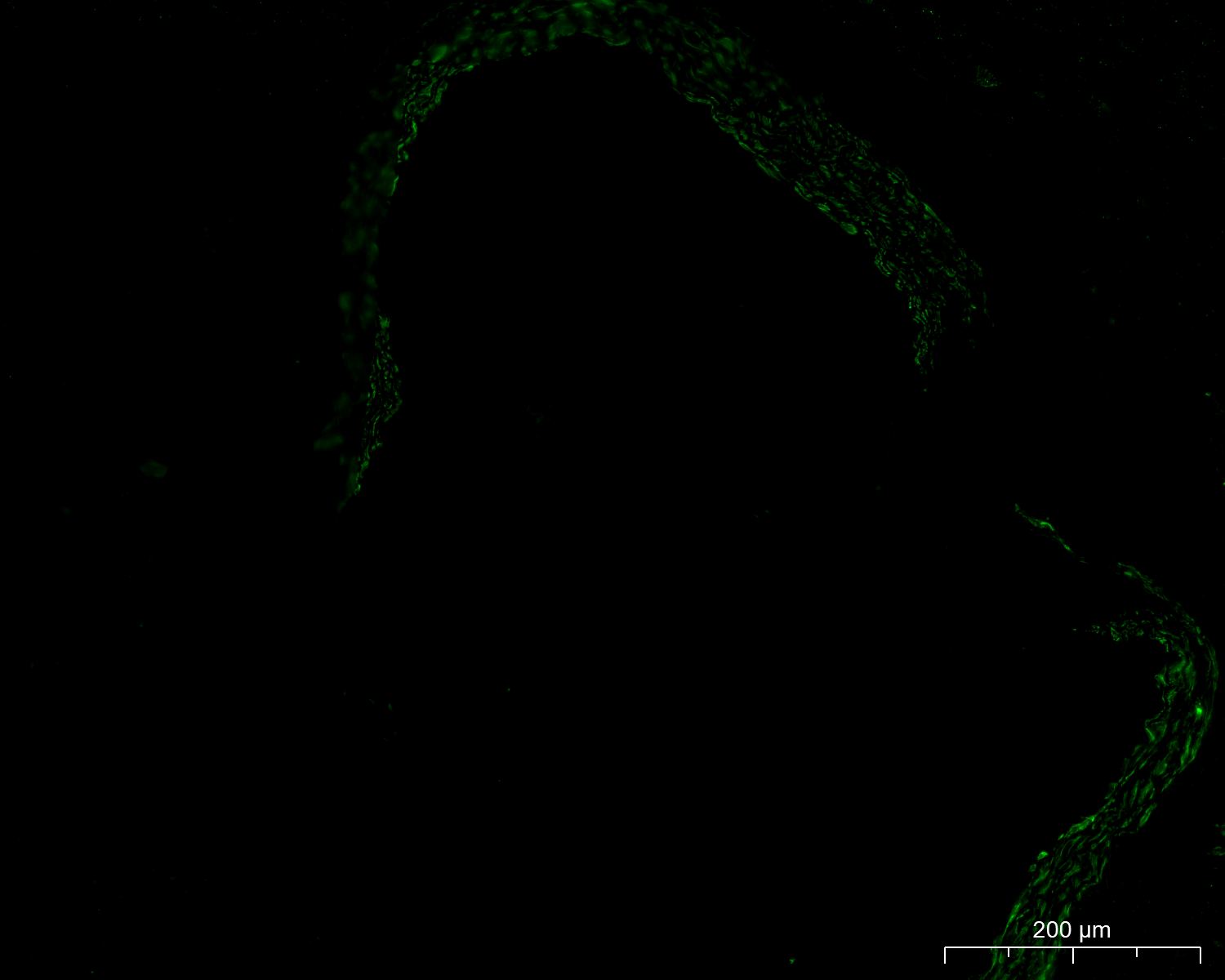

Supplement: S9 File — (ZIP) [file pone.0347758.s009.zip › 主动脉CD36/CD36/control/7 CD36绿_20.0x.tif]

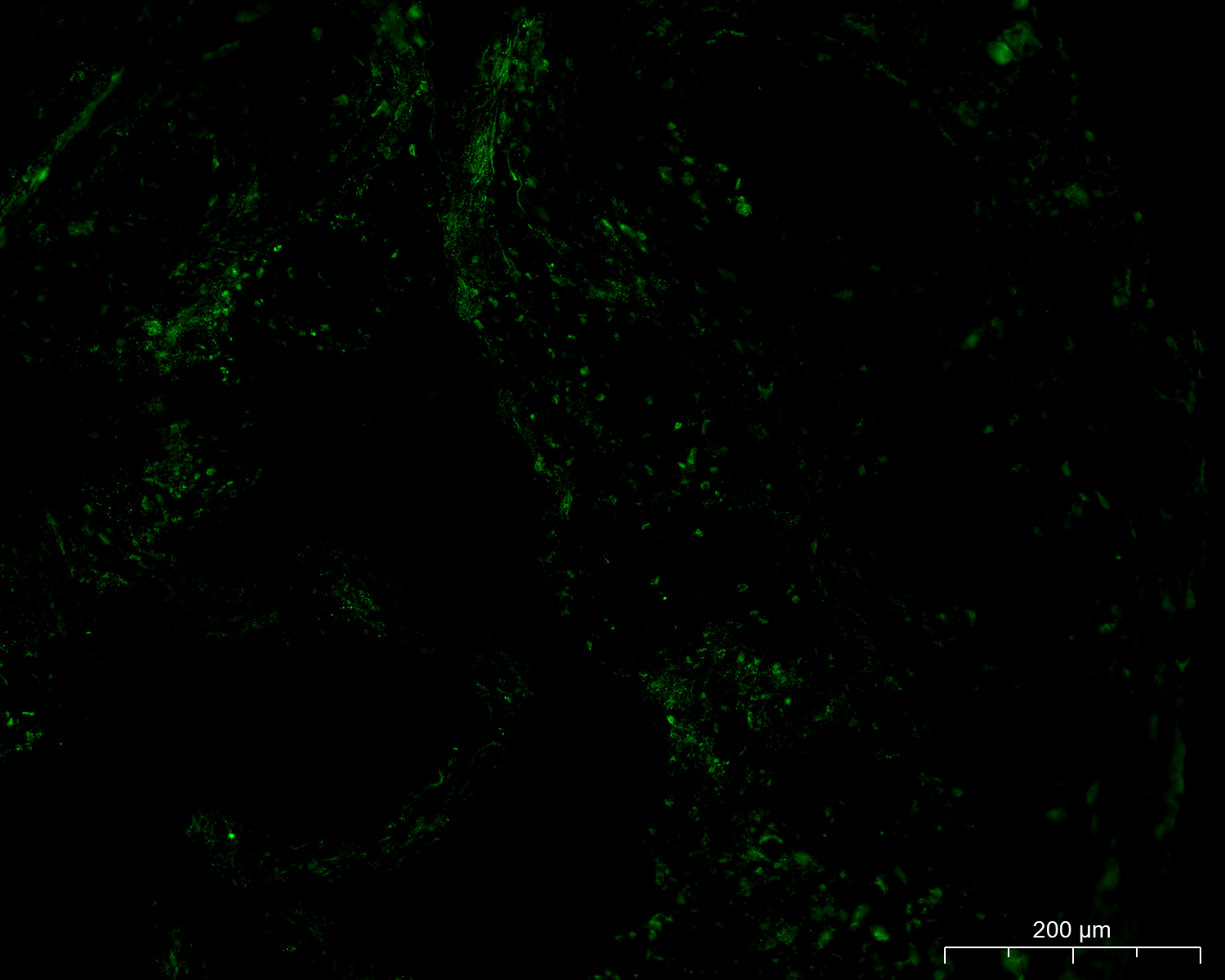

Supplement: S9 File — (ZIP) [file pone.0347758.s009.zip › 主动脉CD36/CD36/PSB-H/100 CD36绿_20.0x.tif]

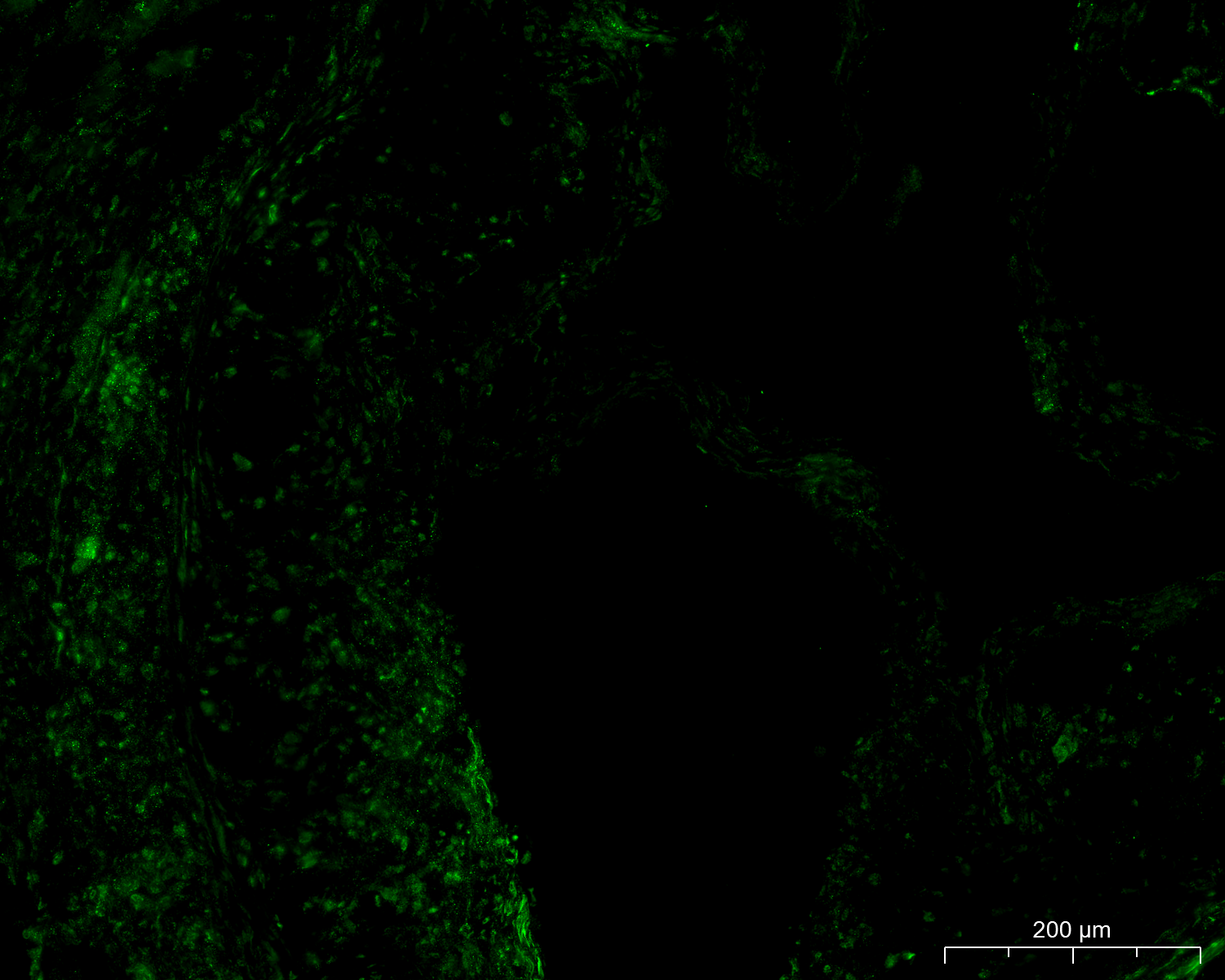

Supplement: S9 File — (ZIP) [file pone.0347758.s009.zip › 主动脉CD36/CD36/PSB-H/93 CD36绿_20.0x.tif]

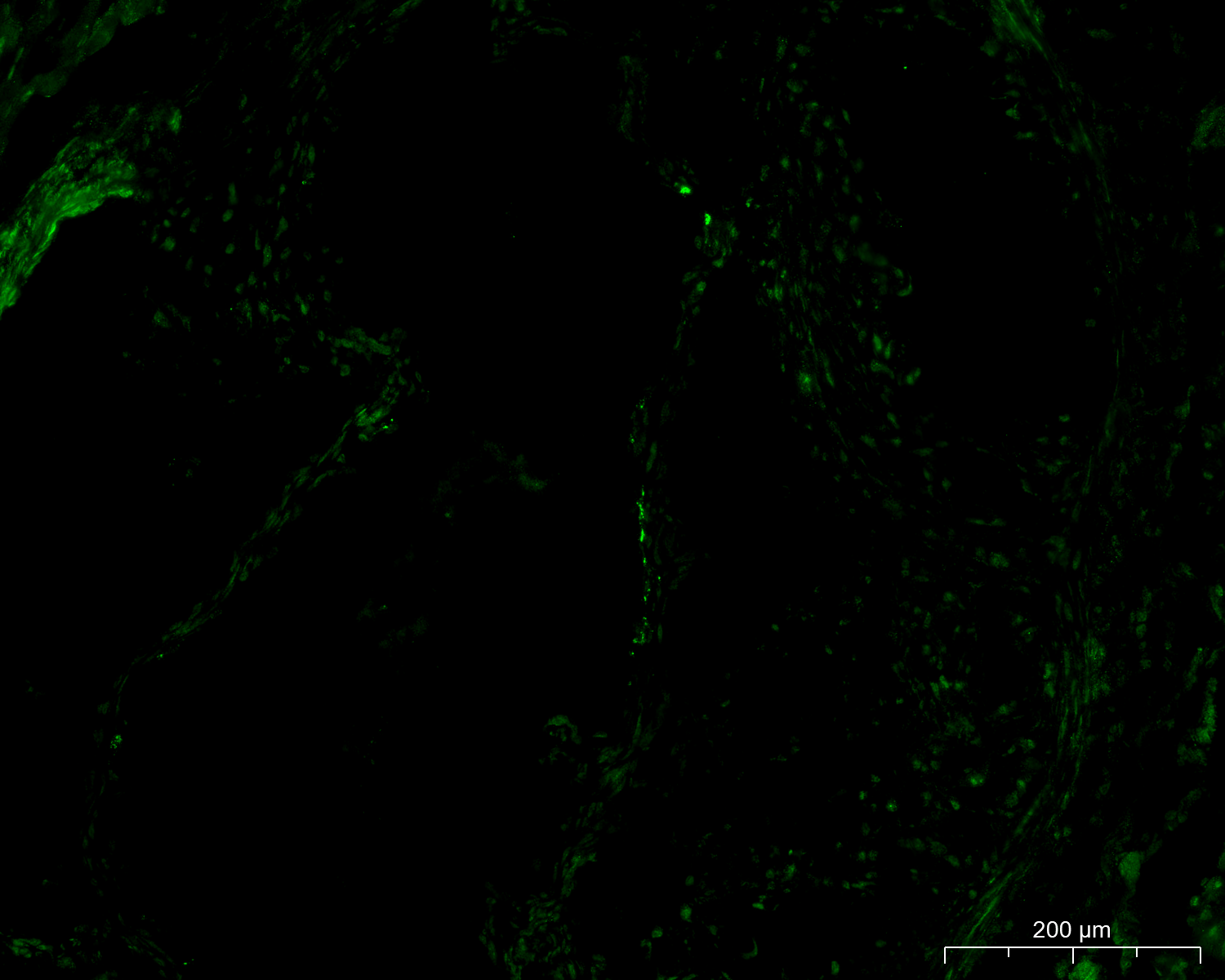

Supplement: S9 File — (ZIP) [file pone.0347758.s009.zip › 主动脉CD36/CD36/PSB-H/98 CD36绿_20.0x.tif]

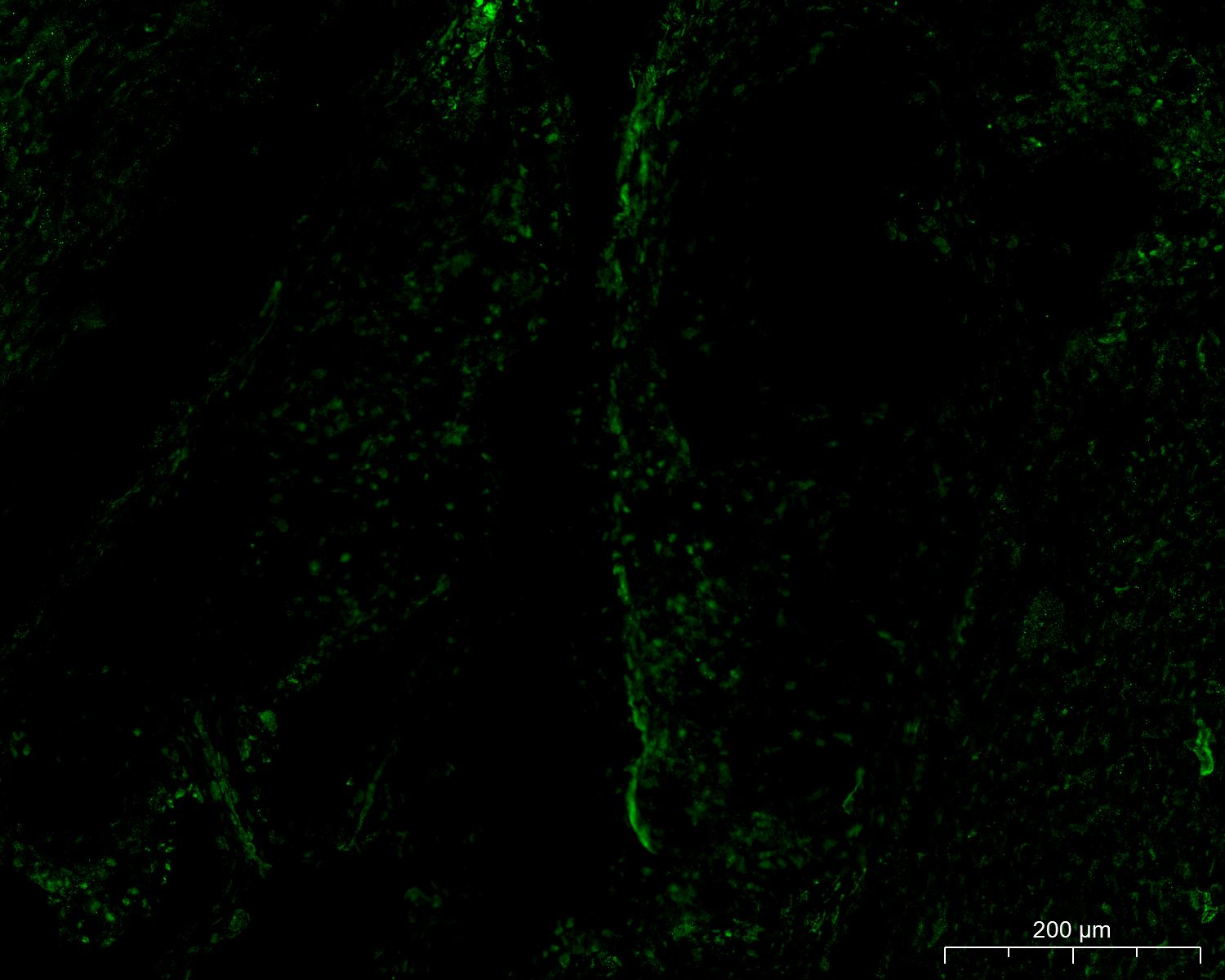

Supplement: S9 File — (ZIP) [file pone.0347758.s009.zip › 主动脉CD36/CD36/PSB-H/A1 CD36绿_20.0x.tif]

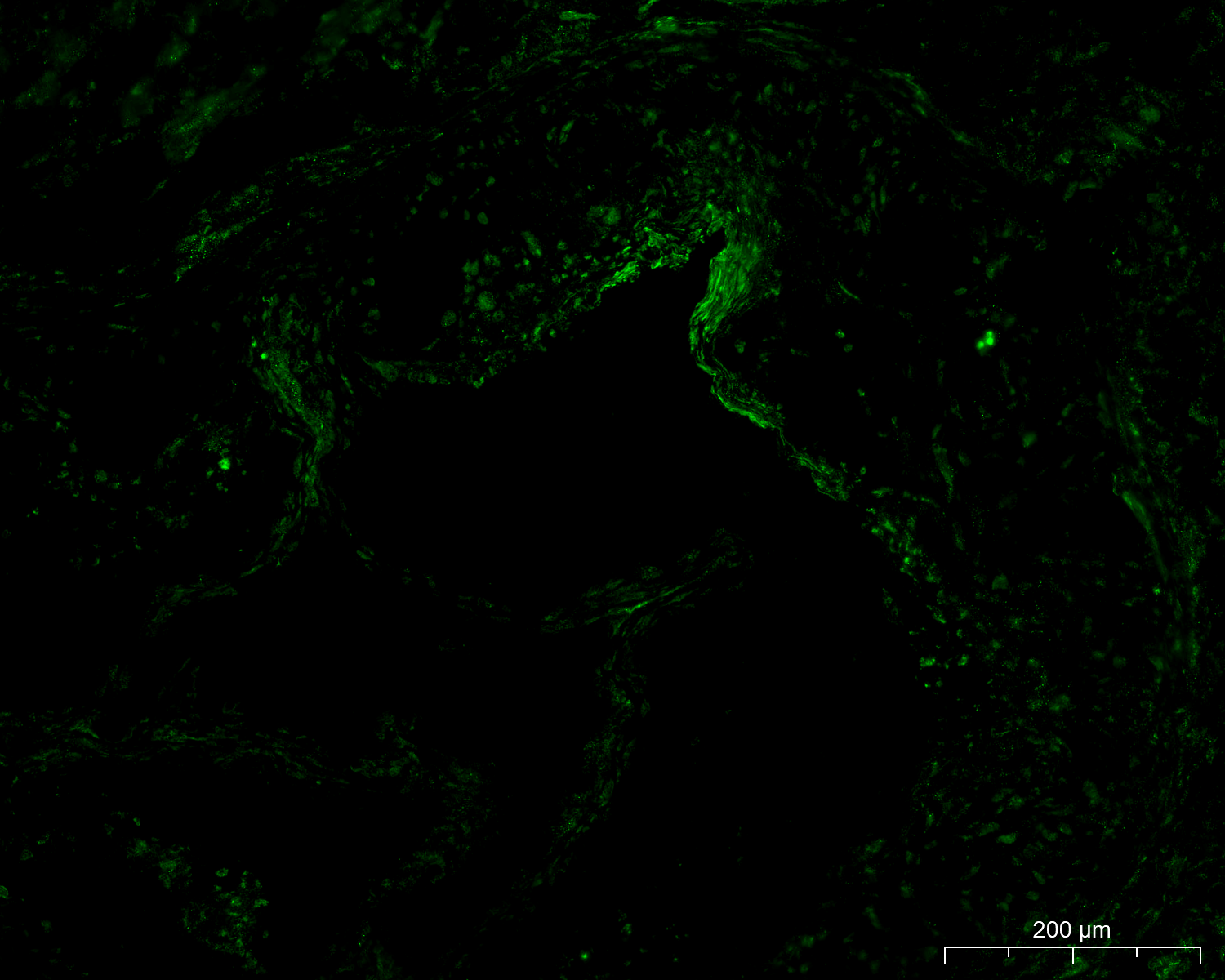

Supplement: S9 File — (ZIP) [file pone.0347758.s009.zip › 主动脉CD36/CD36/PSB-L/74 CD36绿_20.0x.tif]

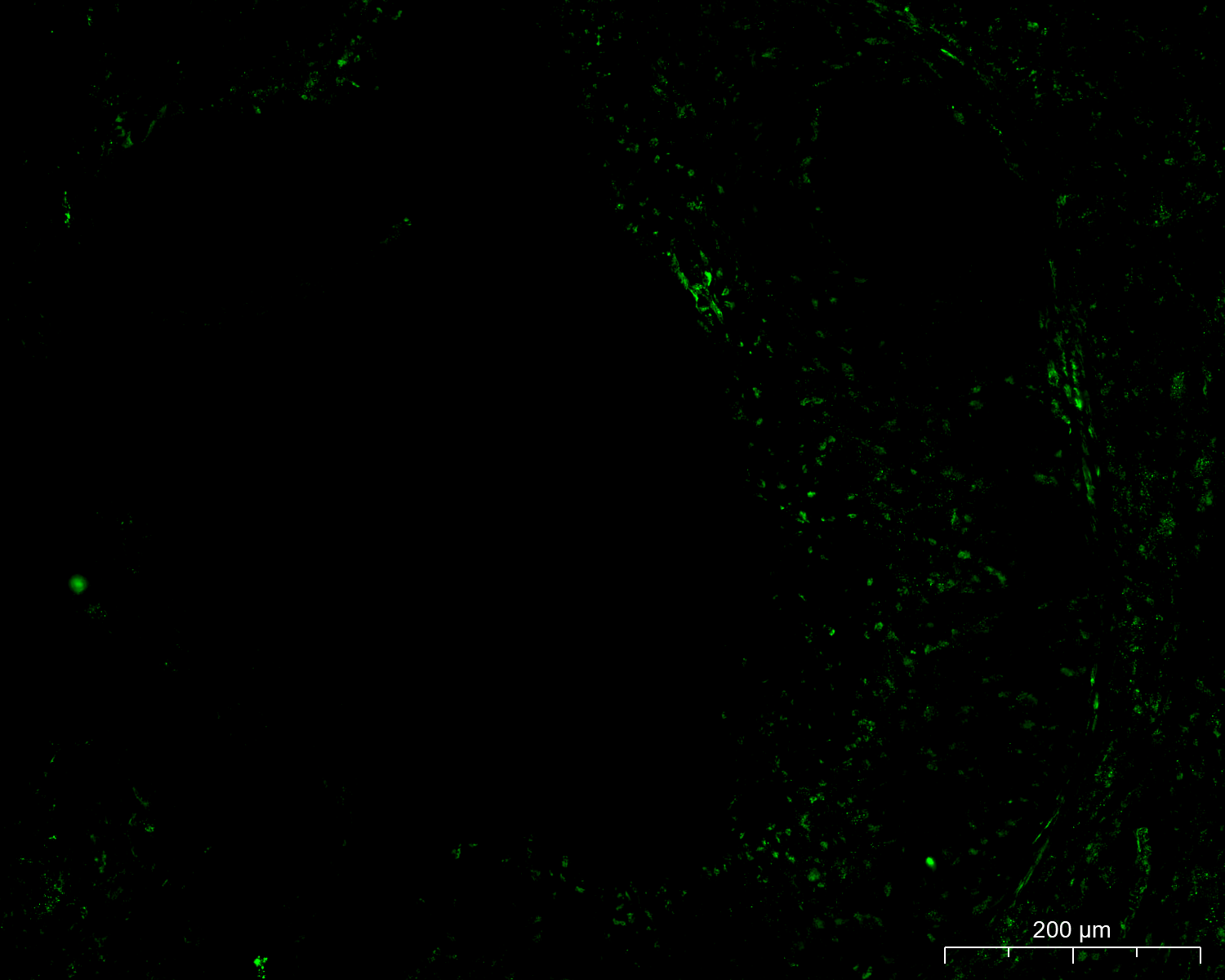

Supplement: S9 File — (ZIP) [file pone.0347758.s009.zip › 主动脉CD36/CD36/PSB-L/77 CD36绿_20.0x.tif]

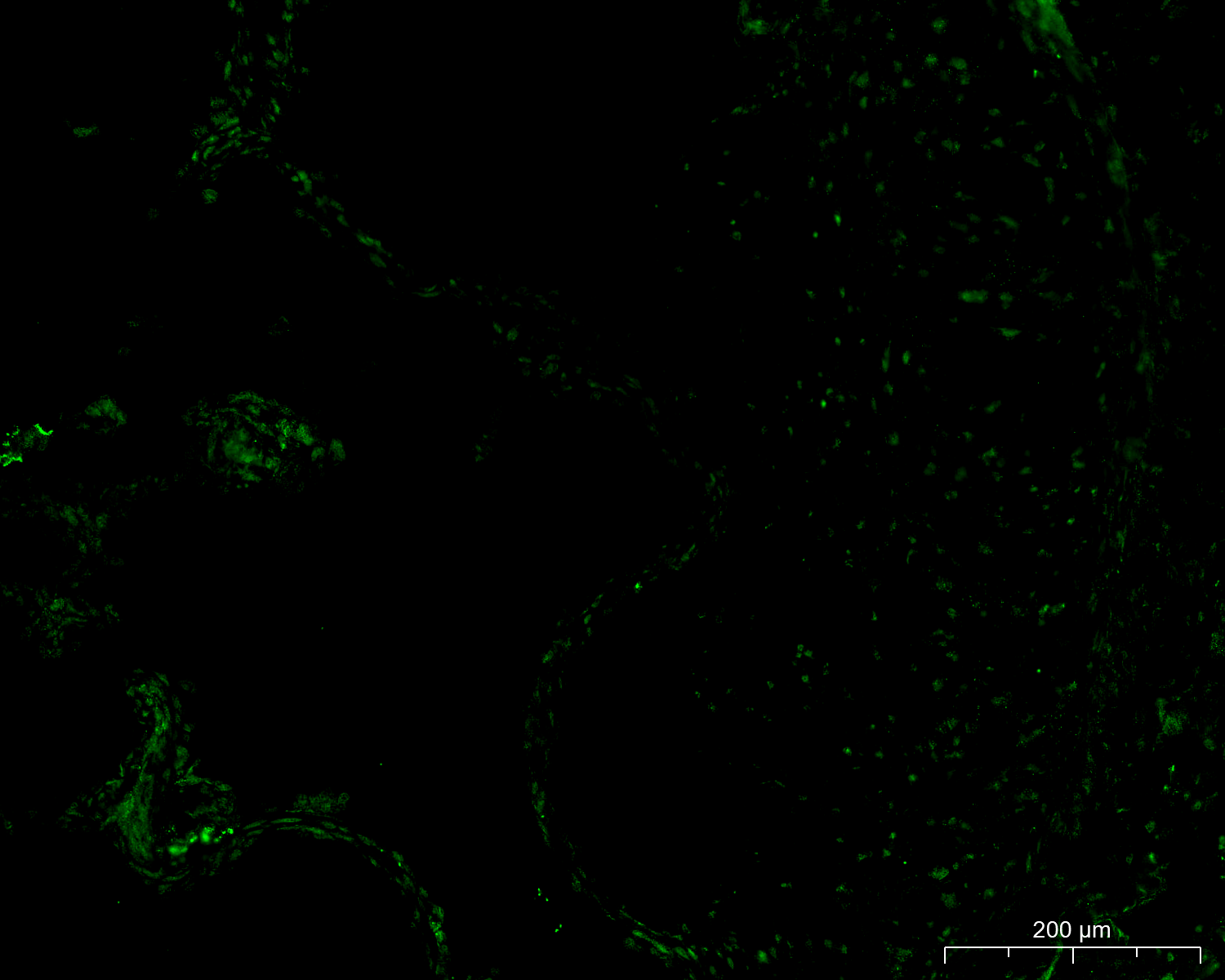

Supplement: S9 File — (ZIP) [file pone.0347758.s009.zip › 主动脉CD36/CD36/PSB-L/80 CD36绿_20.0x.tif]

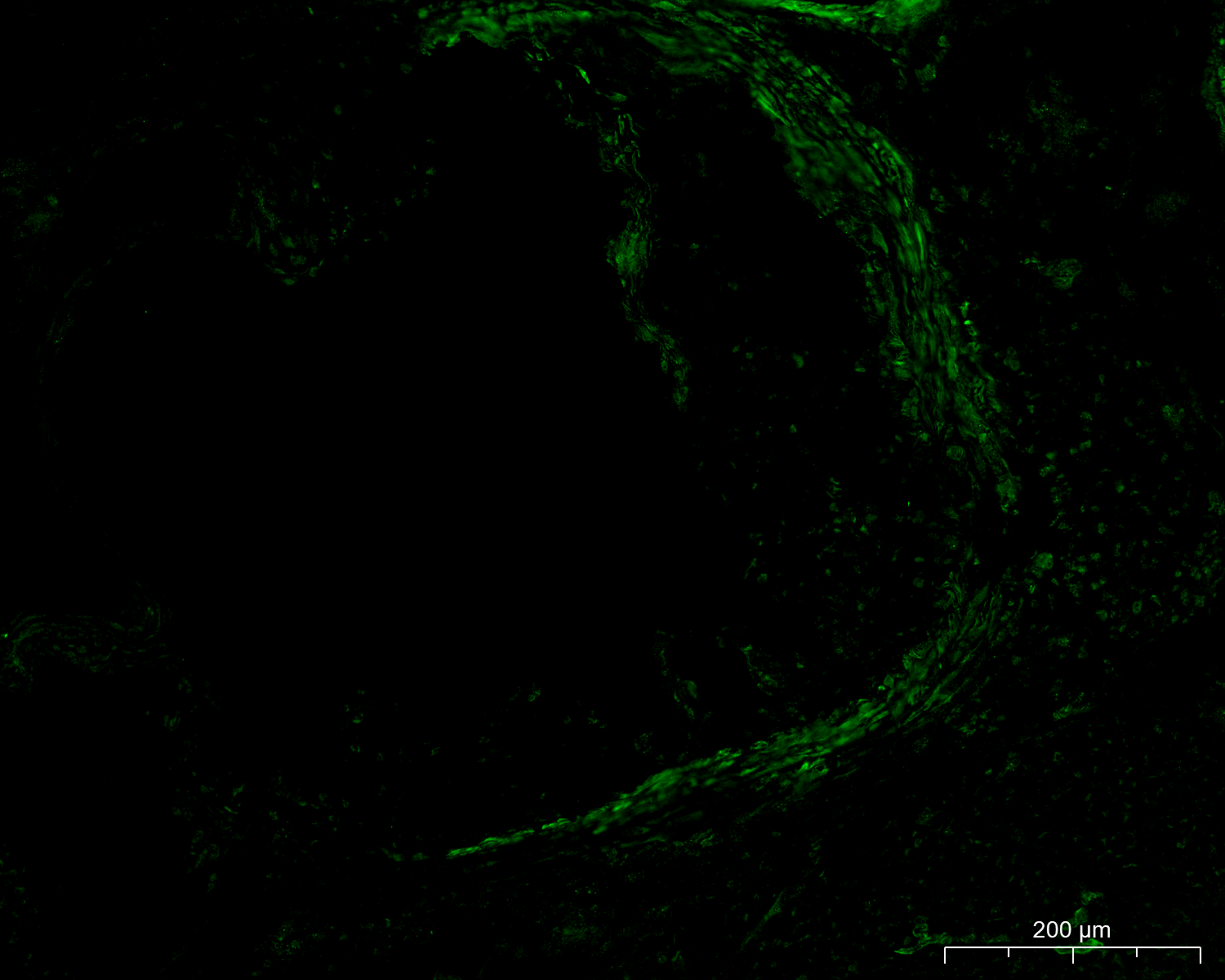

Supplement: S9 File — (ZIP) [file pone.0347758.s009.zip › 主动脉CD36/CD36/PSB-L/82 CD36绿_20.0x.tif]

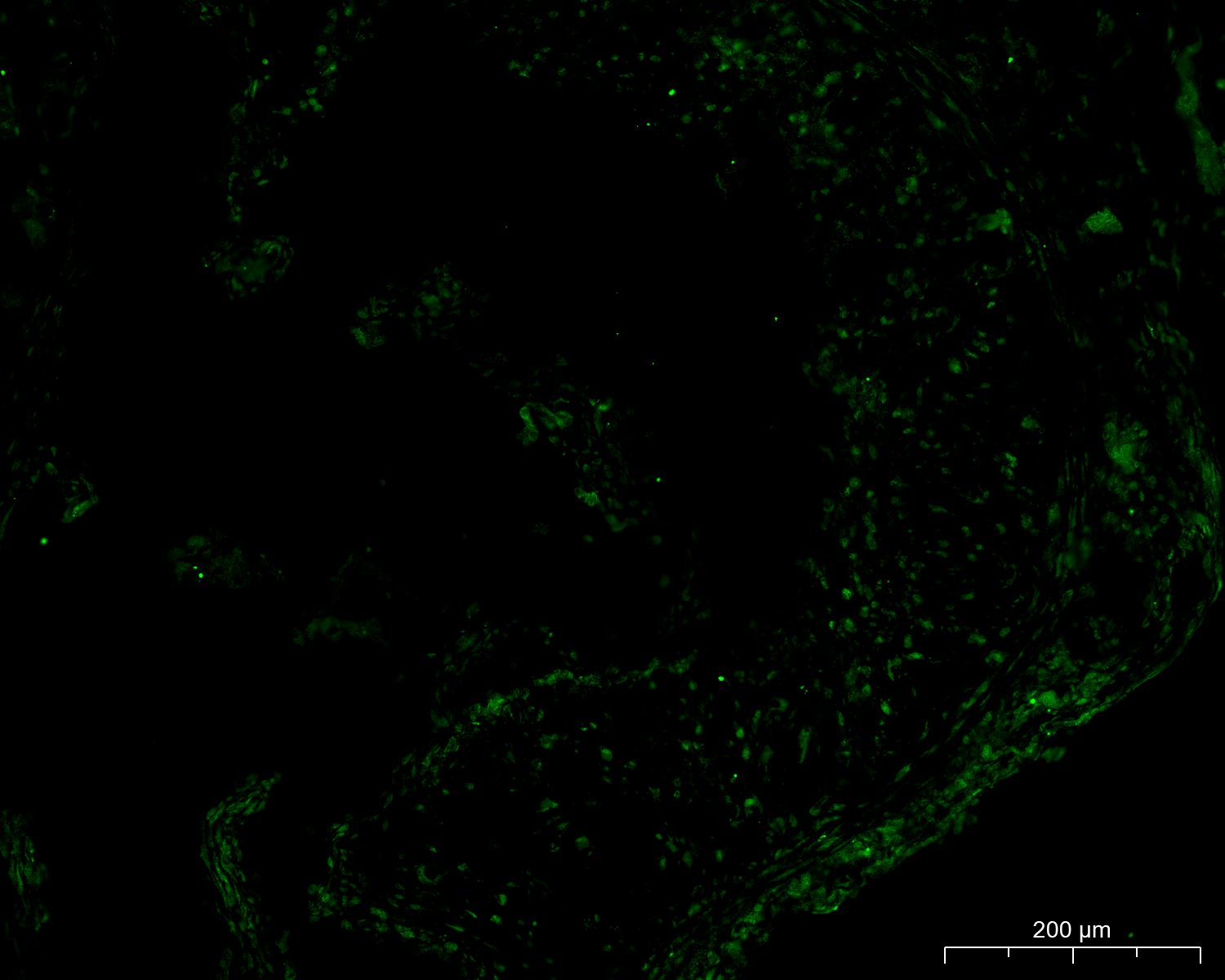

Supplement: S9 File — (ZIP) [file pone.0347758.s009.zip › 主动脉CD36/CD36/PSB-M/85 CD36绿_20.0x.tif]

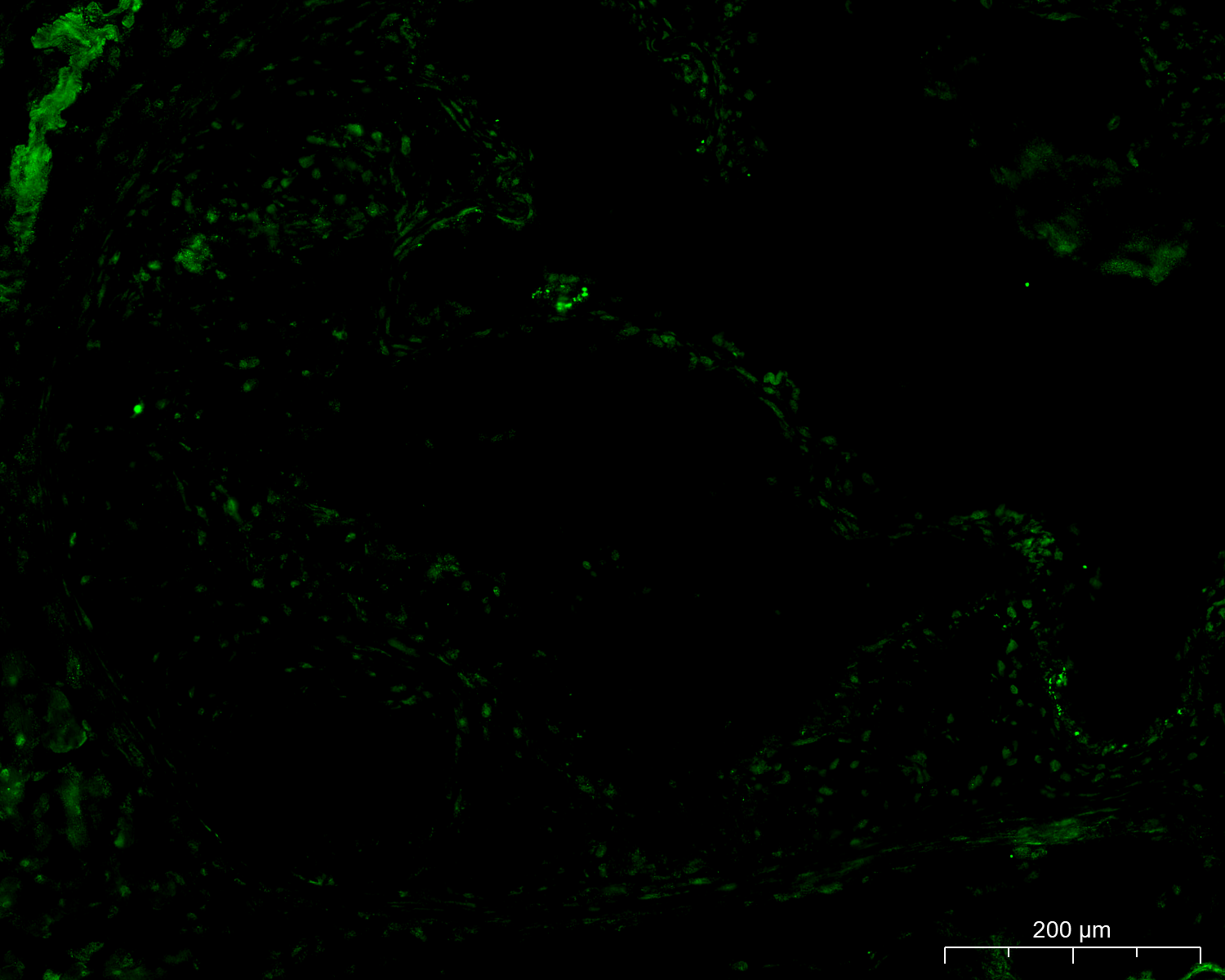

Supplement: S9 File — (ZIP) [file pone.0347758.s009.zip › 主动脉CD36/CD36/PSB-M/89 CD36绿_20.0x.tif]

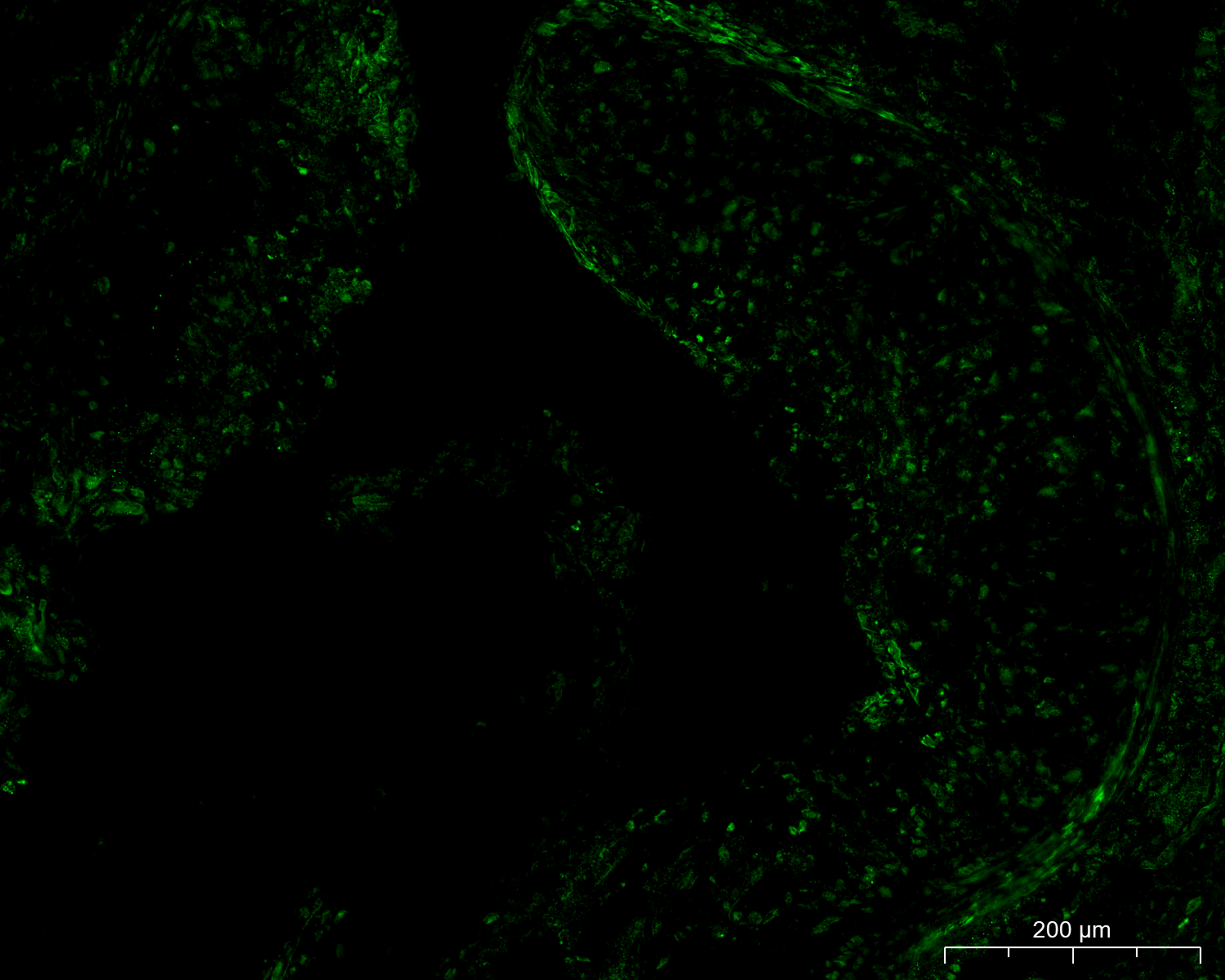

Supplement: S9 File — (ZIP) [file pone.0347758.s009.zip › 主动脉CD36/CD36/PSB-M/90 CD36绿_20.0x.tif]

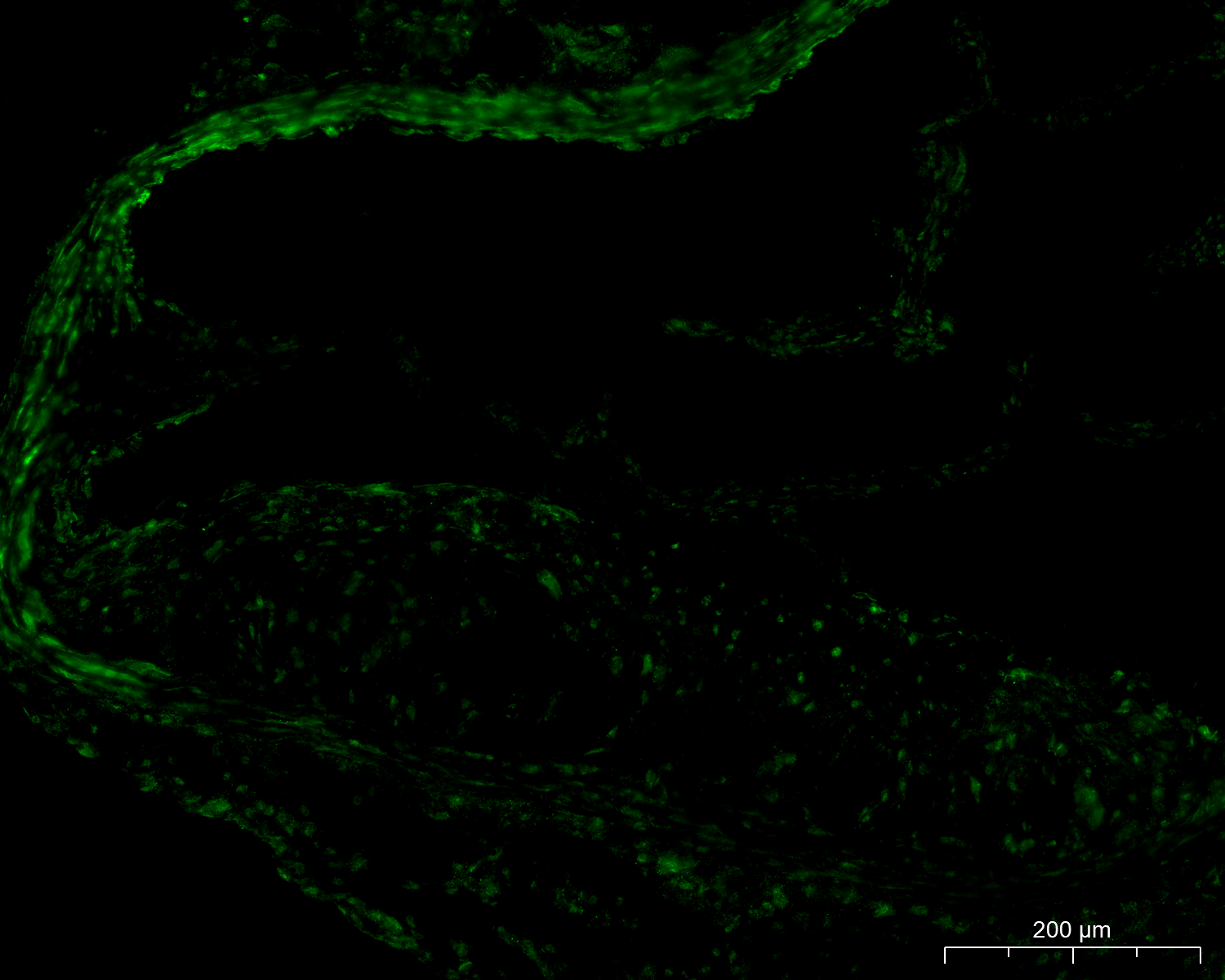

Supplement: S9 File — (ZIP) [file pone.0347758.s009.zip › 主动脉CD36/CD36/PSB-M/92 CD36绿_20.0x.tif]

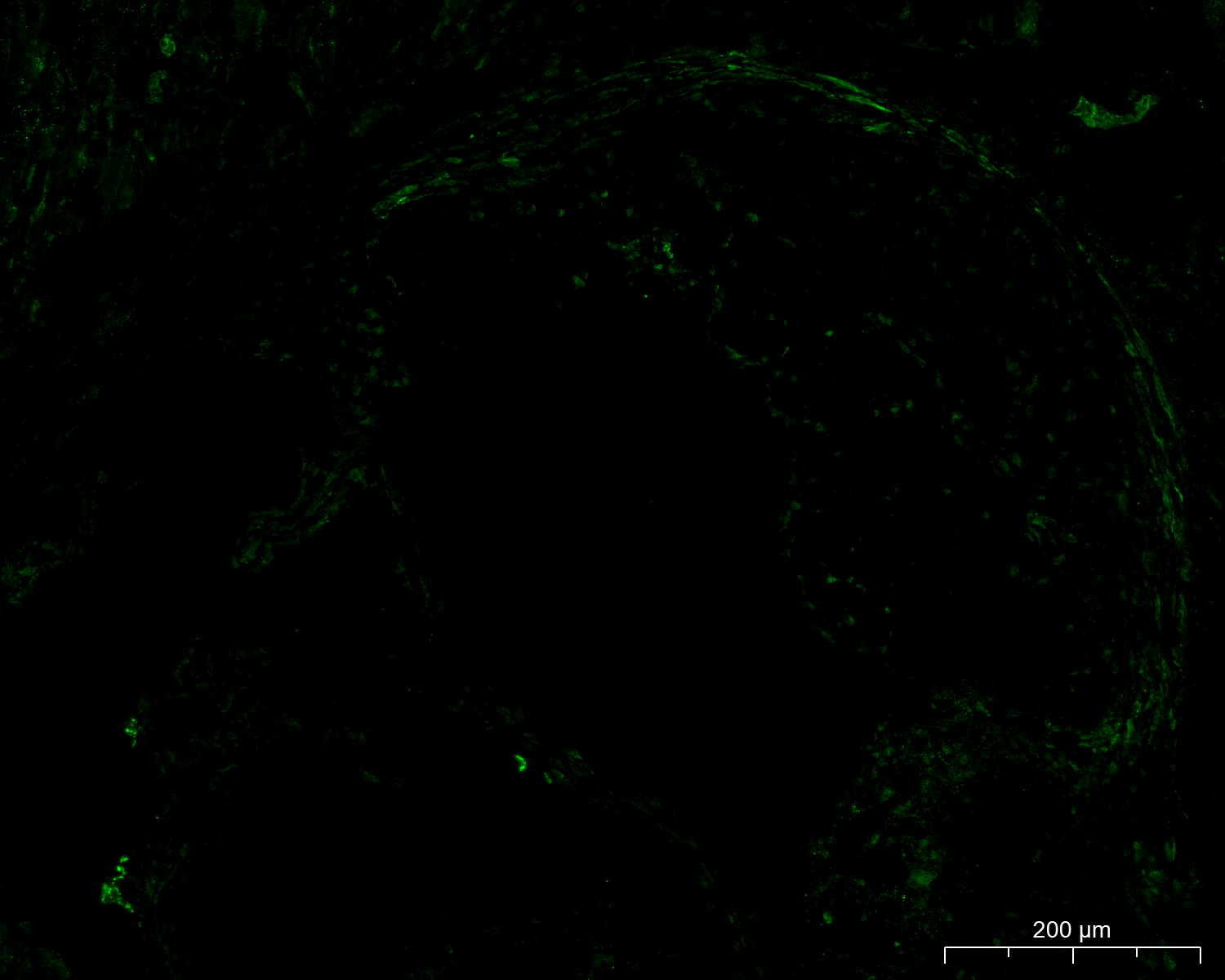

Supplement: S9 File — (ZIP) [file pone.0347758.s009.zip › 主动脉CD36/CD36/statin/37 CD36绿_20.0x.tif]

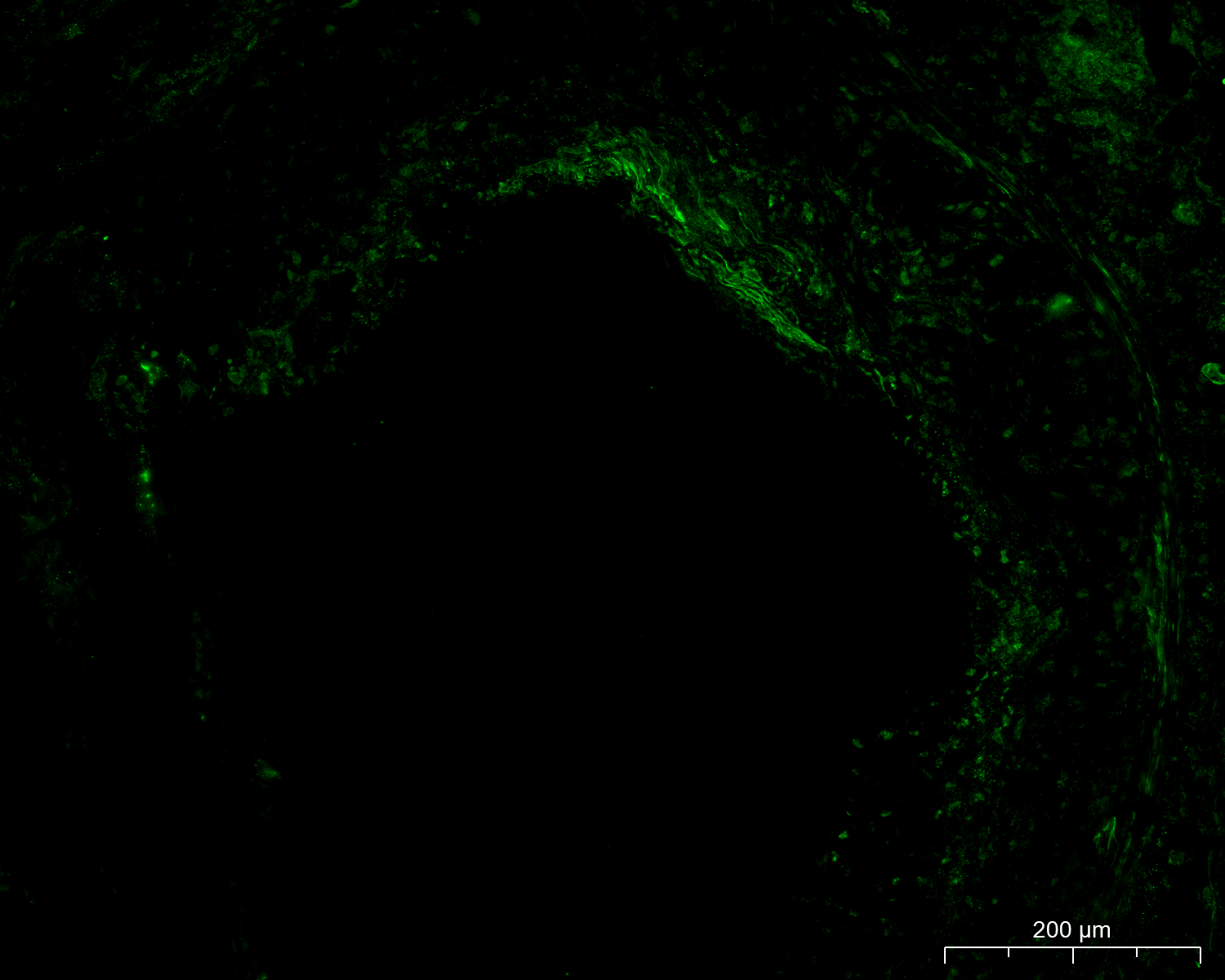

Supplement: S9 File — (ZIP) [file pone.0347758.s009.zip › 主动脉CD36/CD36/statin/38 CD36绿_20.0x.tif]

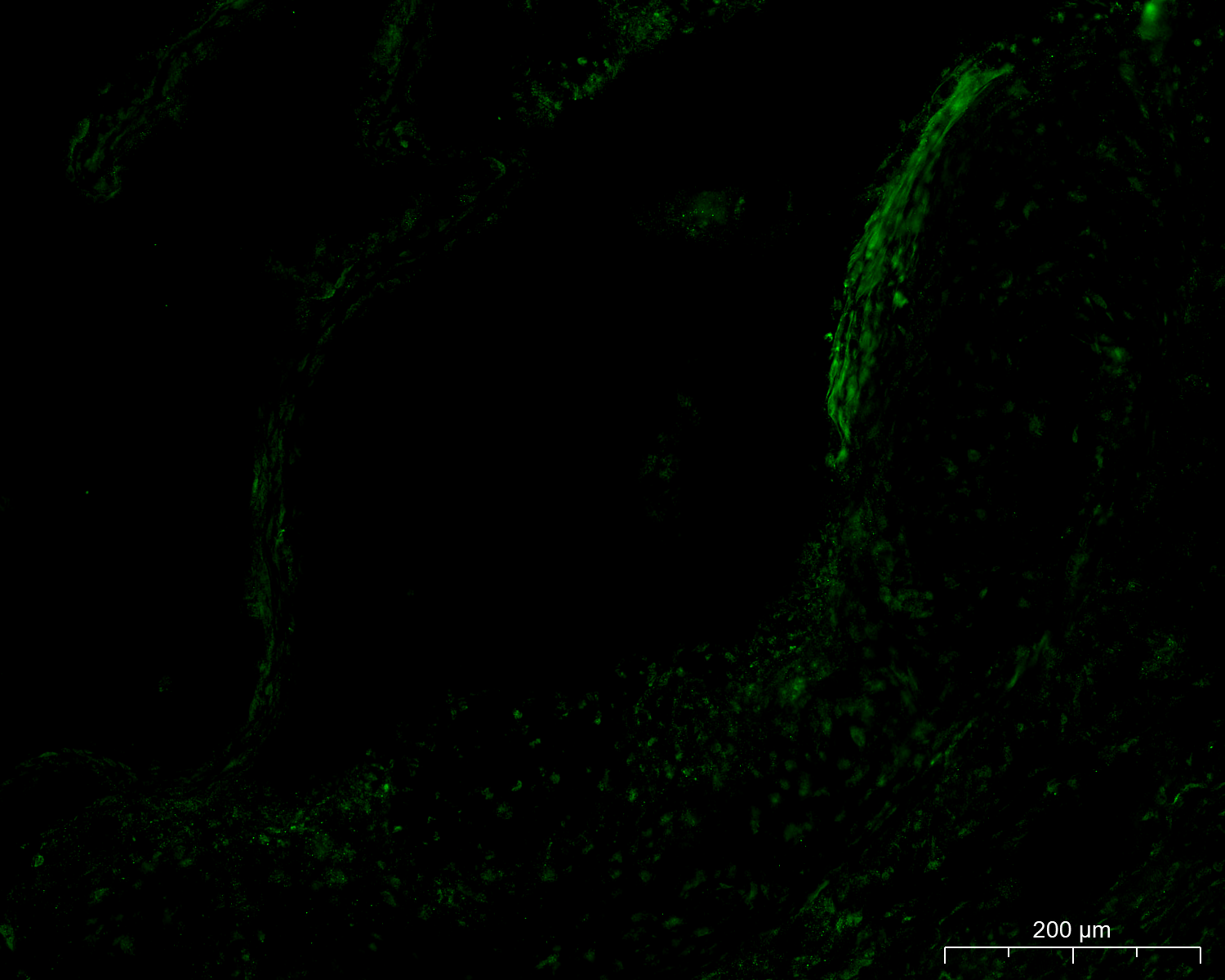

Supplement: S9 File — (ZIP) [file pone.0347758.s009.zip › 主动脉CD36/CD36/statin/40 CD36绿_20.0x.tif]

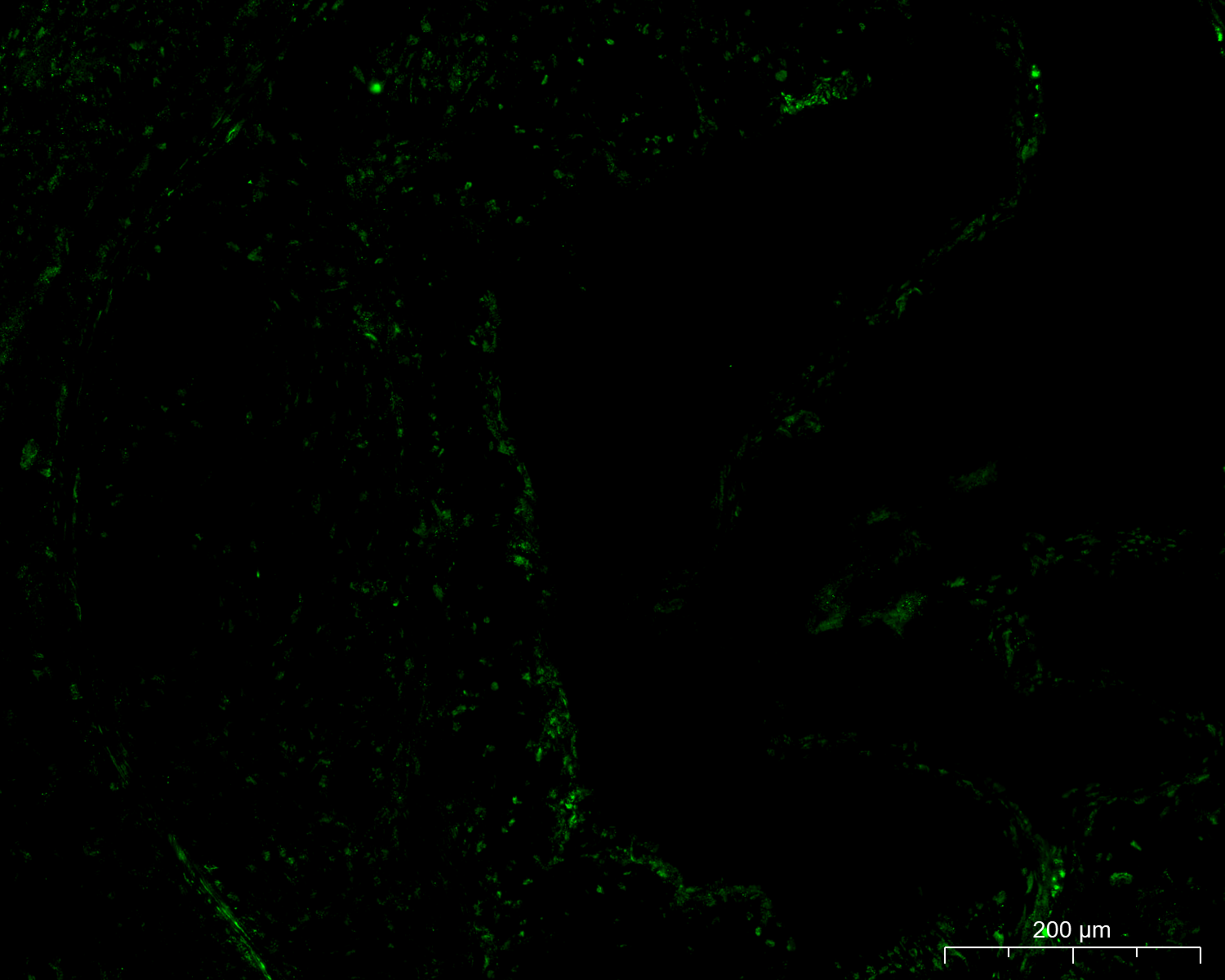

Supplement: S9 File — (ZIP) [file pone.0347758.s009.zip › 主动脉CD36/CD36/statin/41 CD36绿_20.0x.tif]

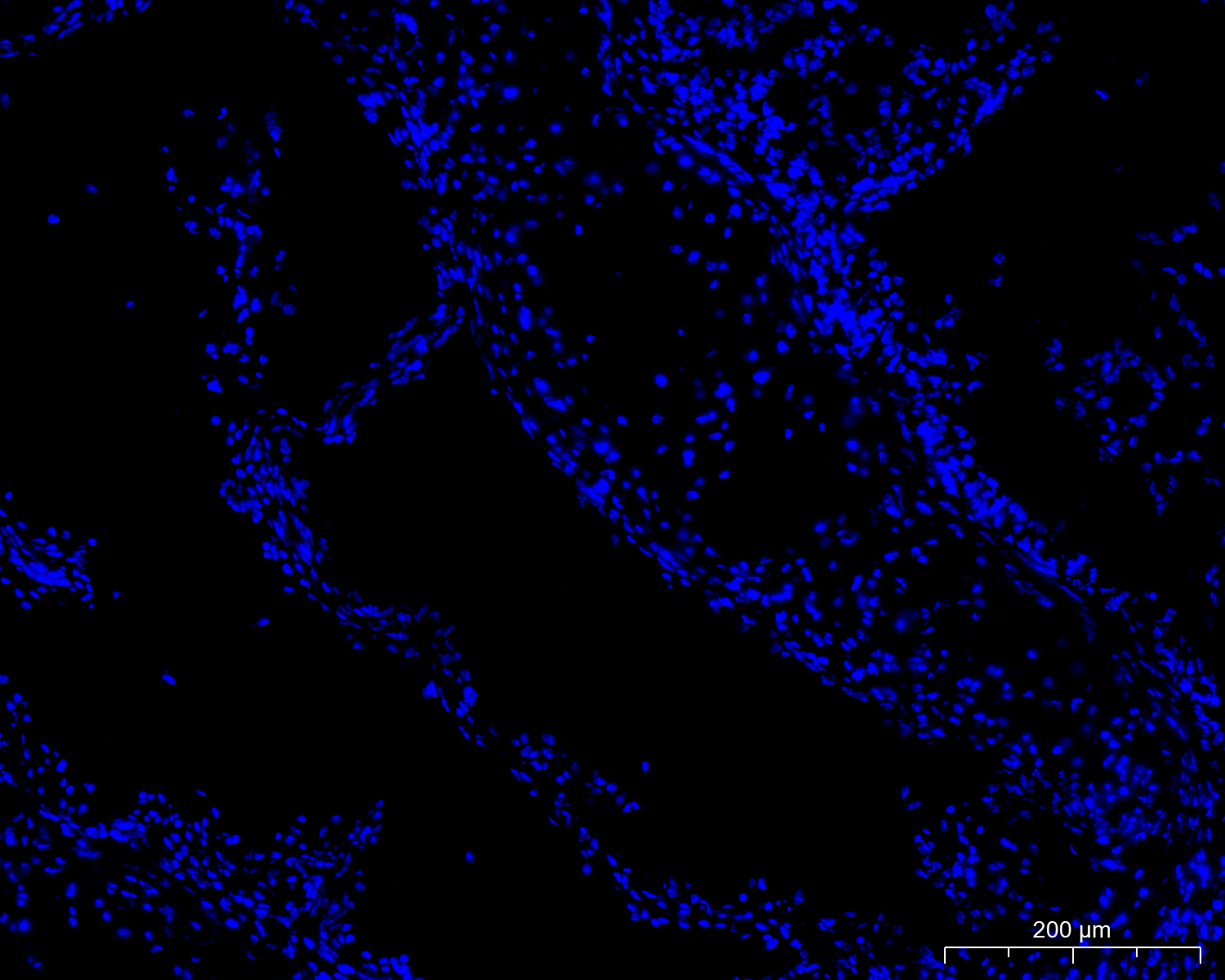

Supplement: S9 File — (ZIP) [file pone.0347758.s009.zip › 主动脉CD36/DAPI/AS/23 CD36绿_20.0x.tif]

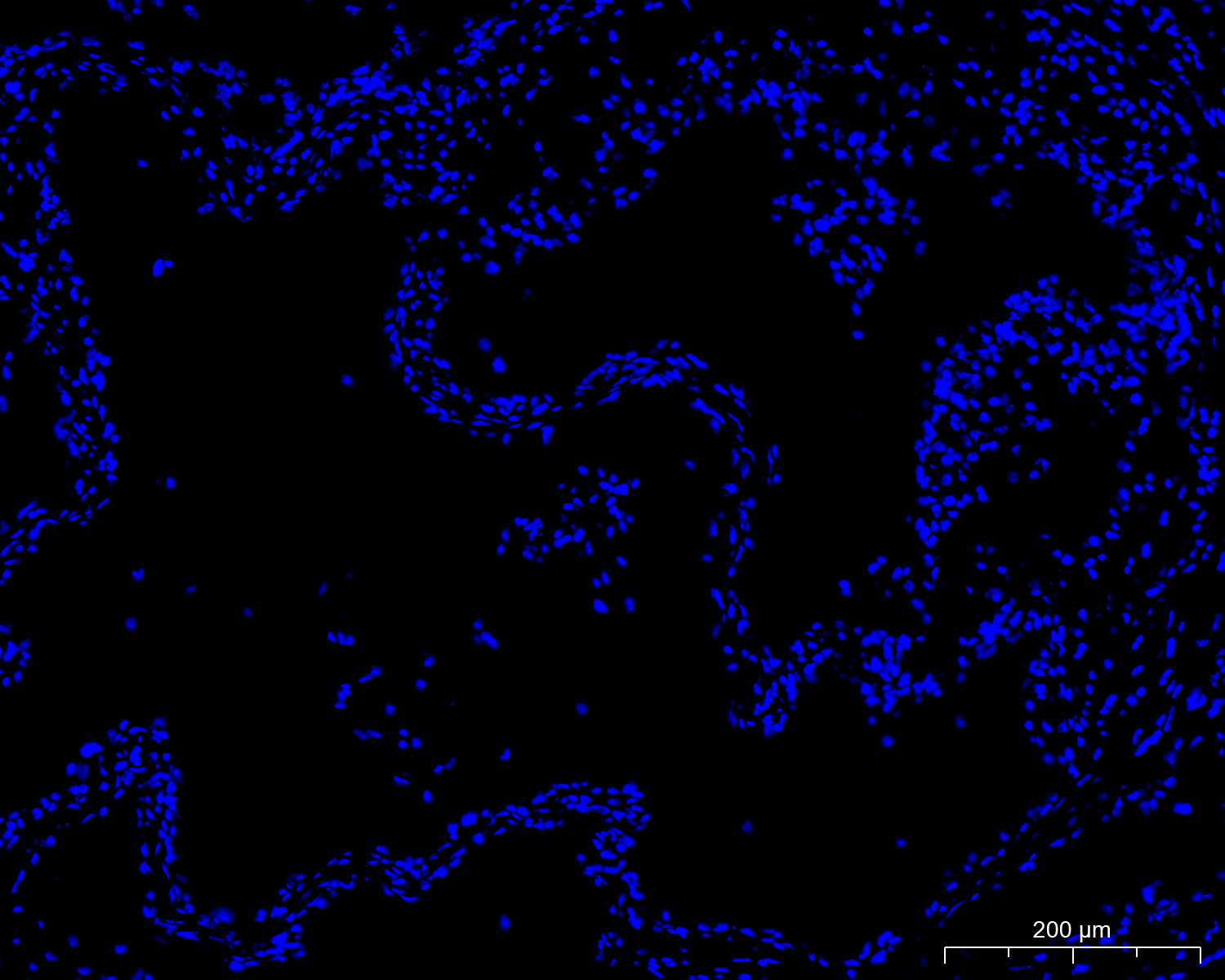

Supplement: S9 File — (ZIP) [file pone.0347758.s009.zip › 主动脉CD36/DAPI/AS/27 CD36绿_20.0x.tif]

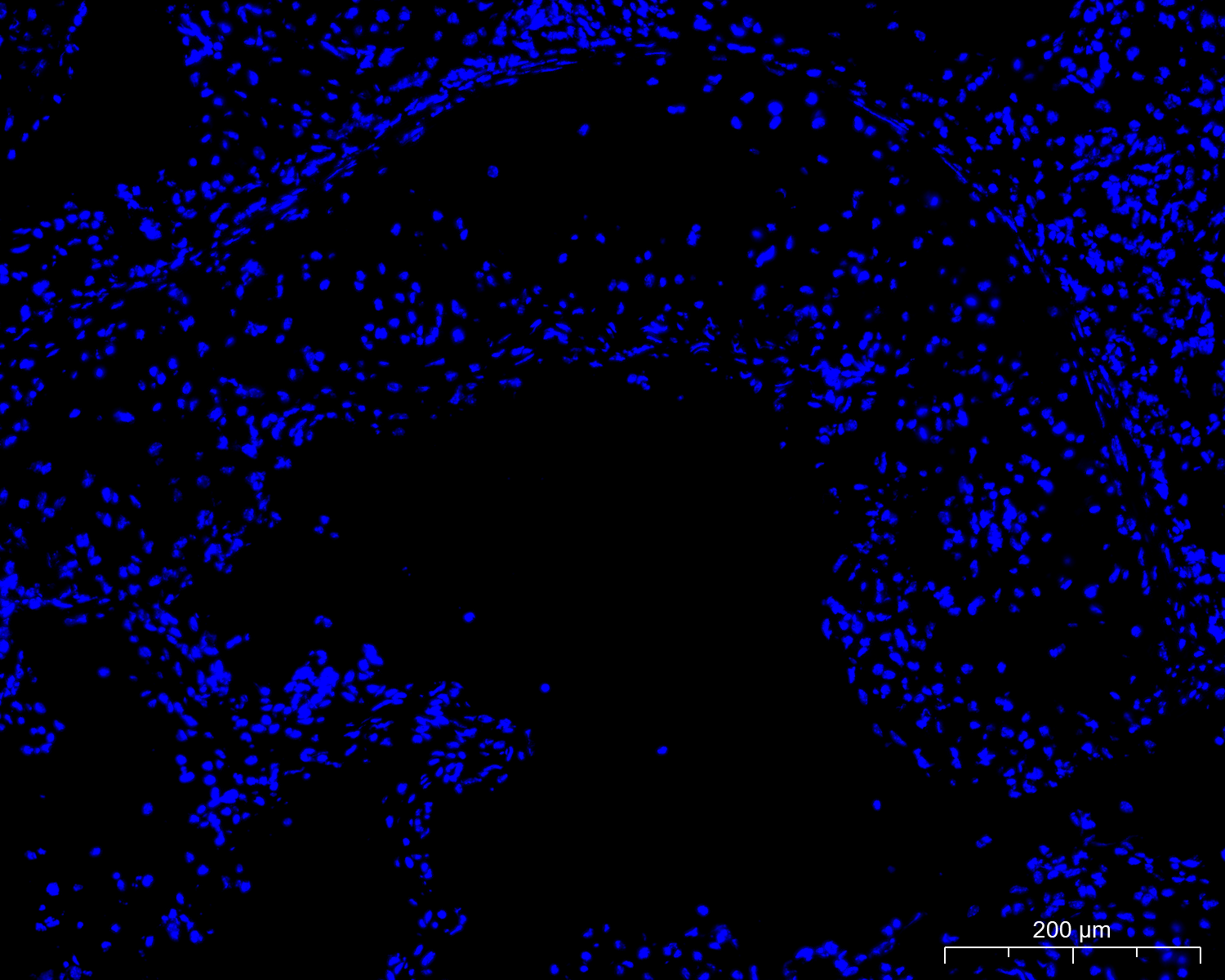

Supplement: S9 File — (ZIP) [file pone.0347758.s009.zip › 主动脉CD36/DAPI/AS/28 CD36绿_20.0x.tif]

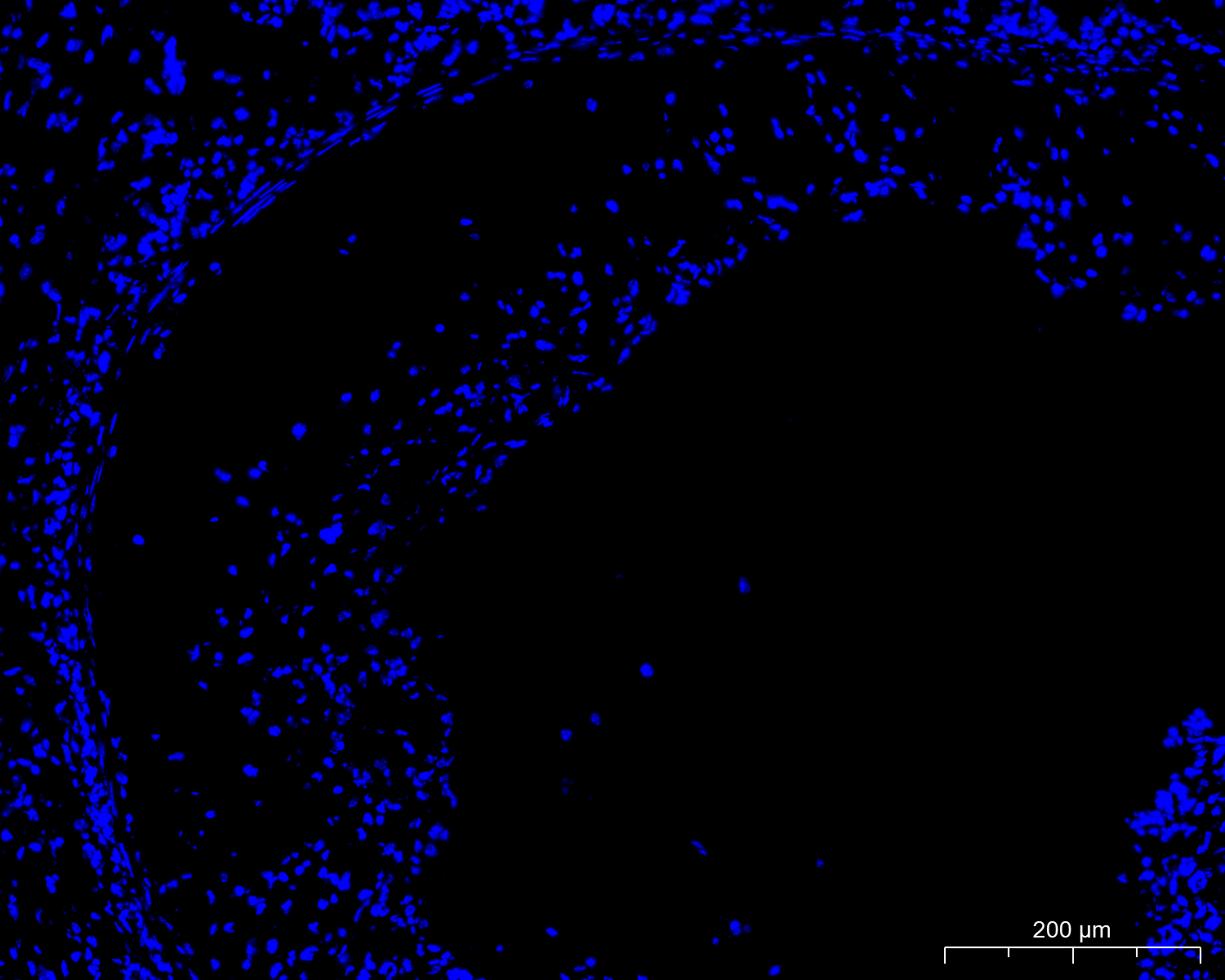

Supplement: S9 File — (ZIP) [file pone.0347758.s009.zip › 主动脉CD36/DAPI/AS/31 CD36绿_20.0x.tif]

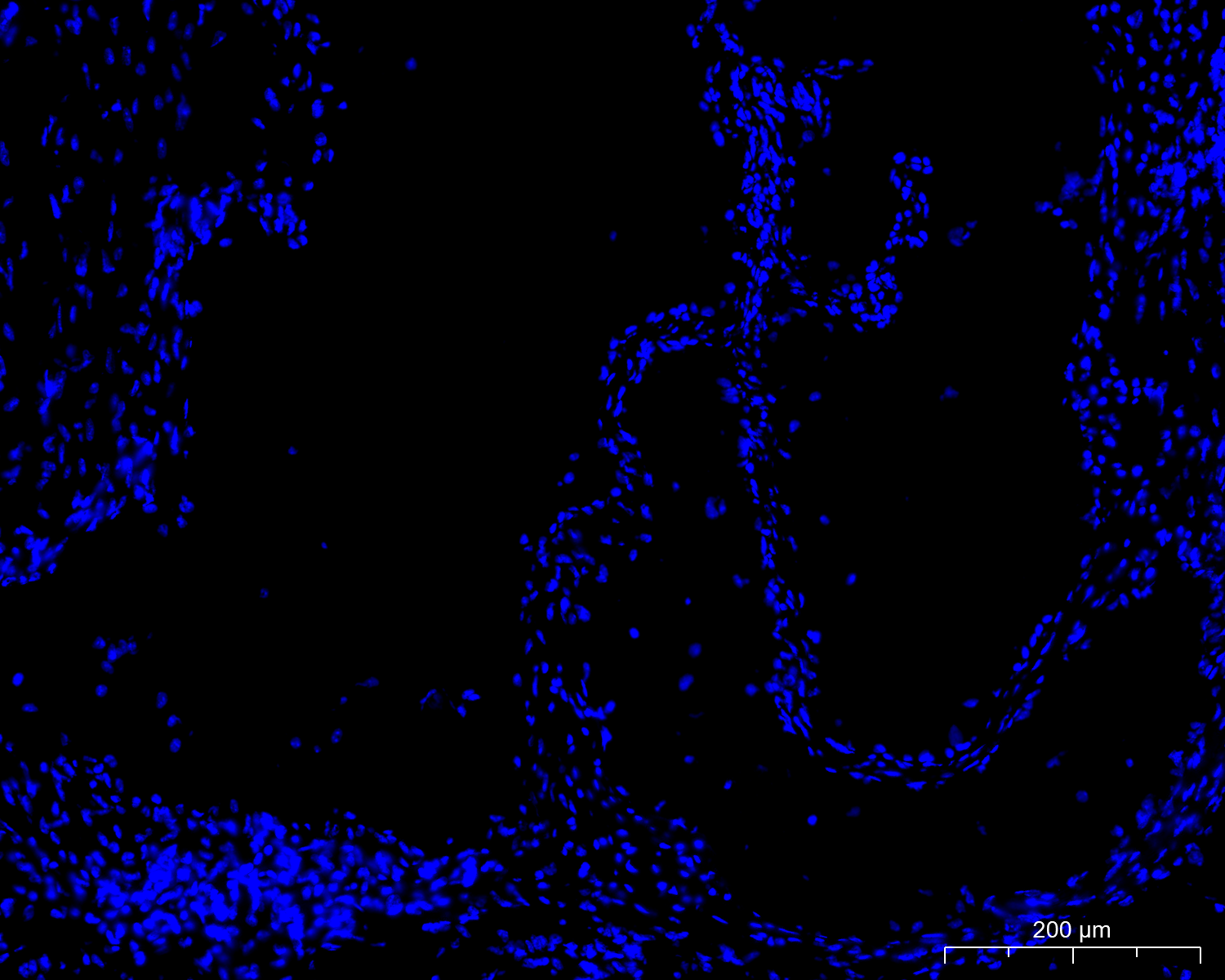

Supplement: S9 File — (ZIP) [file pone.0347758.s009.zip › 主动脉CD36/DAPI/control/1 CD36绿_20.0x.tif]

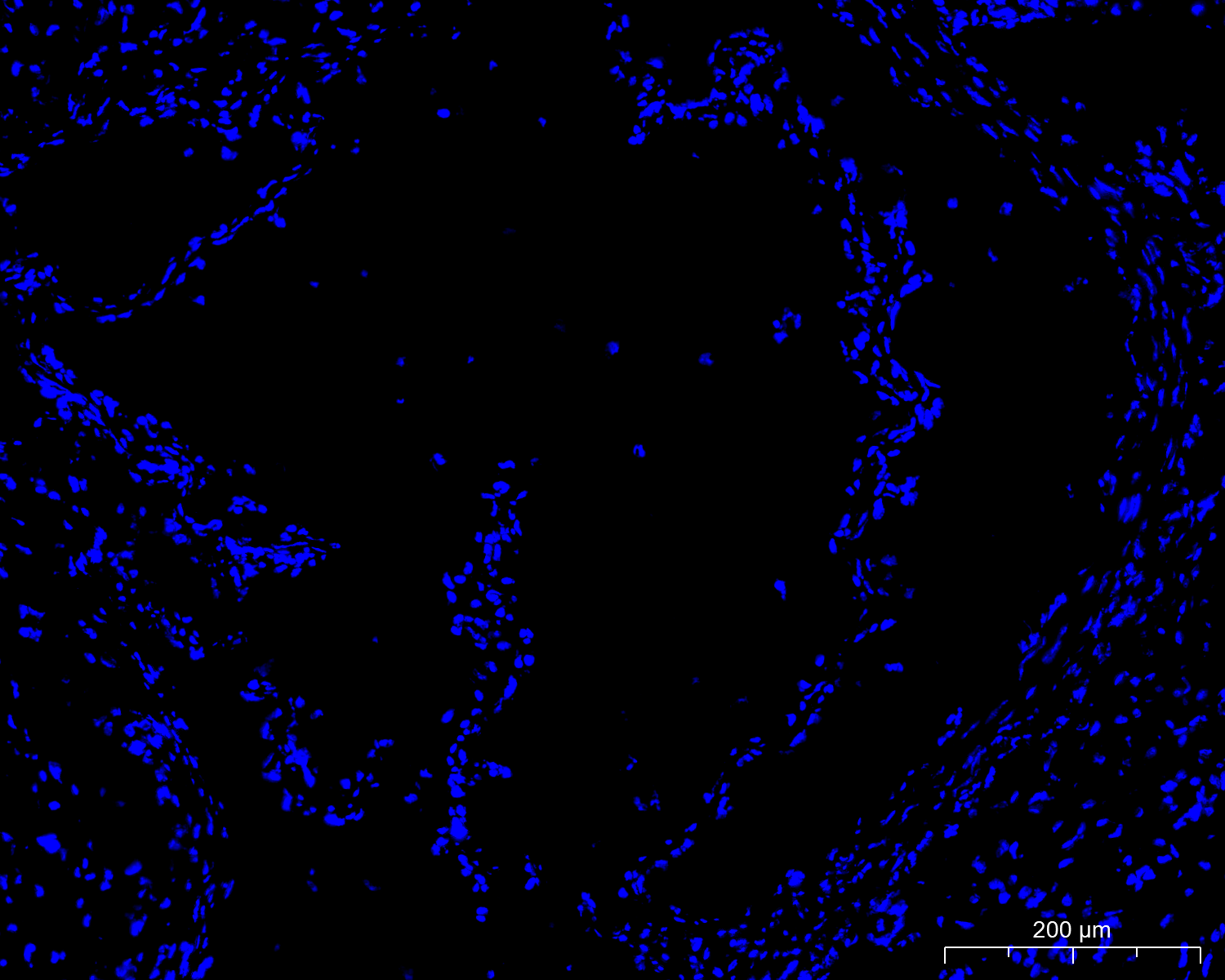

Supplement: S9 File — (ZIP) [file pone.0347758.s009.zip › 主动脉CD36/DAPI/control/2 CD36绿_20.0x.tif]

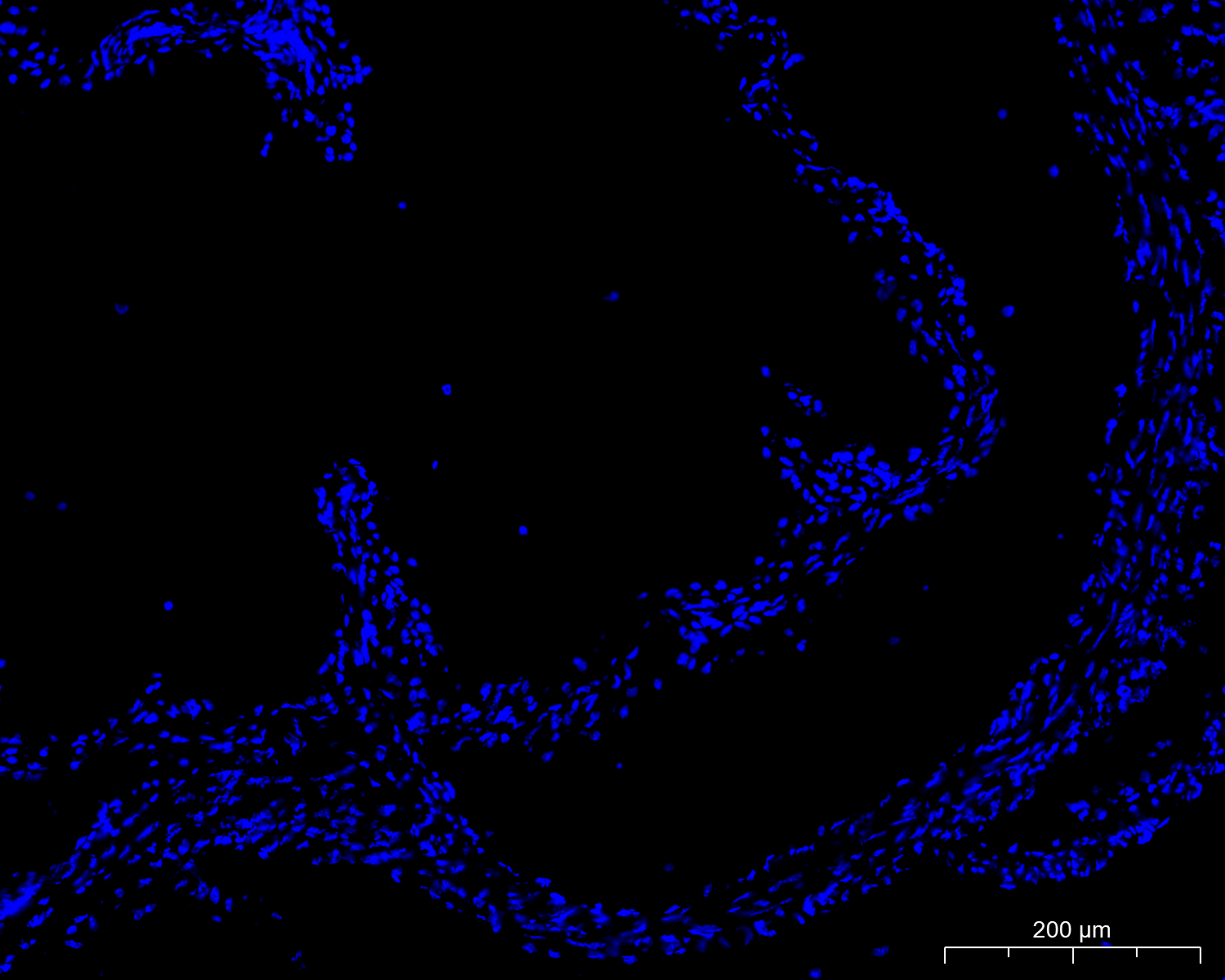

Supplement: S9 File — (ZIP) [file pone.0347758.s009.zip › 主动脉CD36/DAPI/control/6 CD36绿_20.0x.tif]

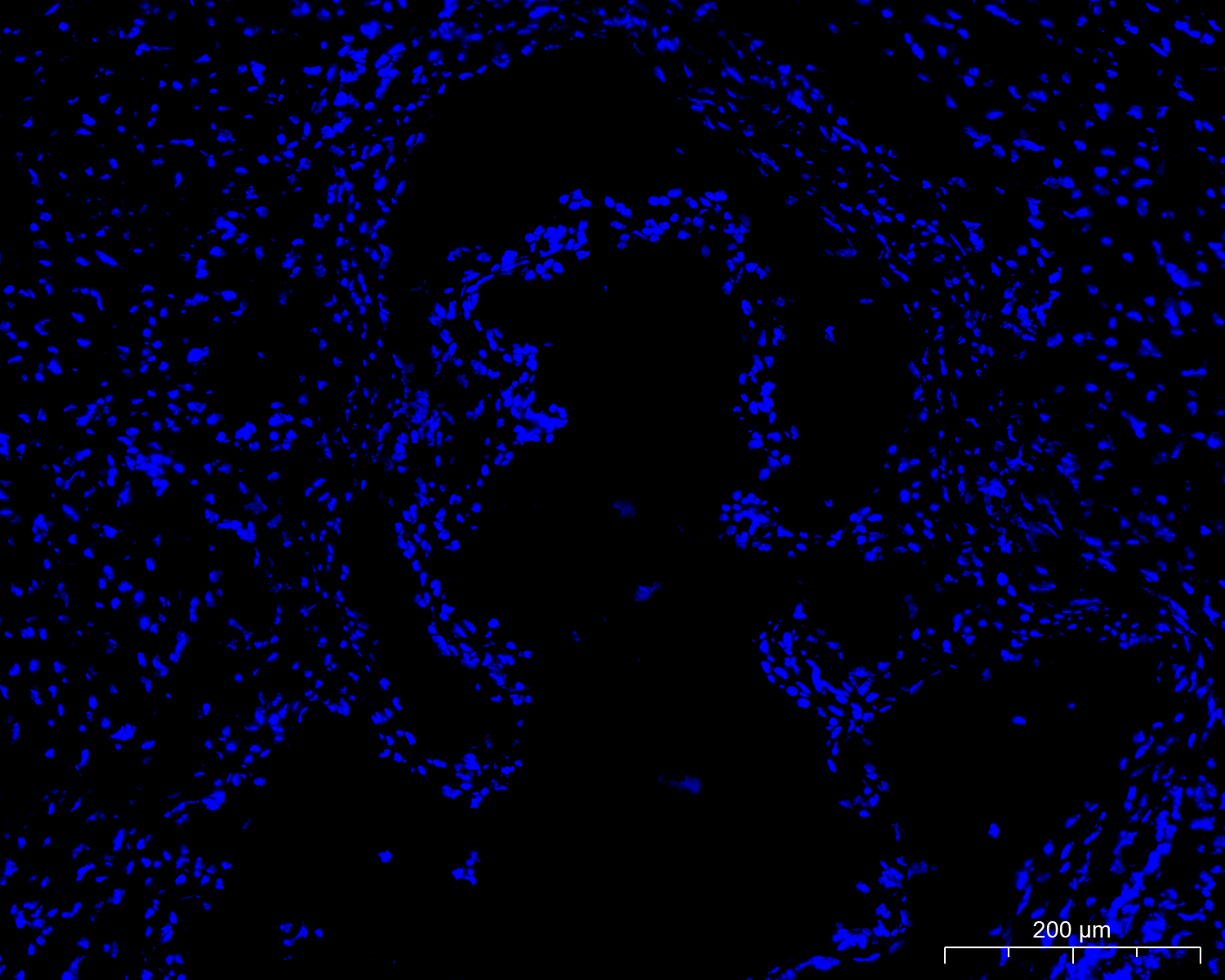

Supplement: S9 File — (ZIP) [file pone.0347758.s009.zip › 主动脉CD36/DAPI/control/7 CD36绿_20.0x.tif]

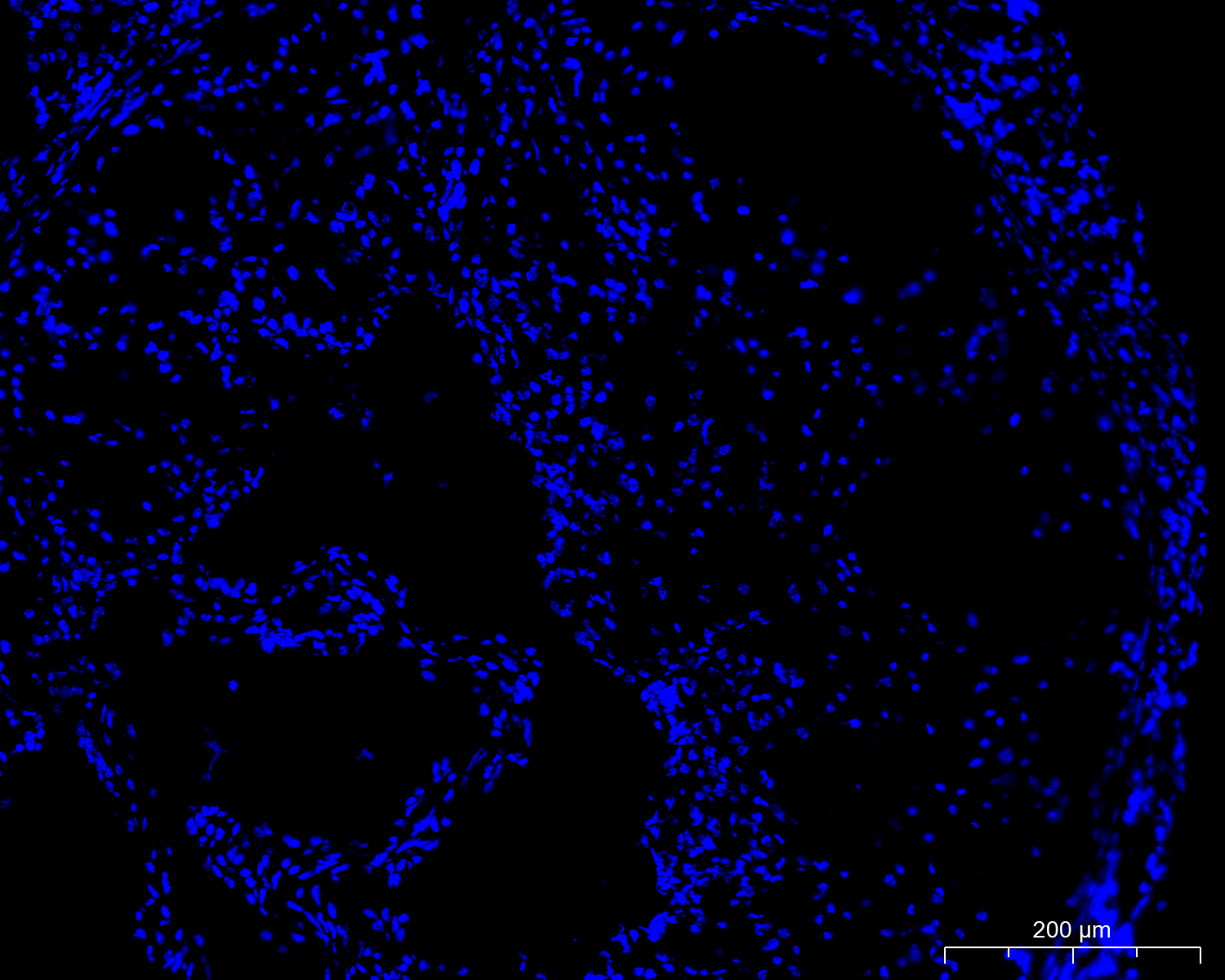

Supplement: S9 File — (ZIP) [file pone.0347758.s009.zip › 主动脉CD36/DAPI/PSB-H/100 CD36绿_20.0x.tif]

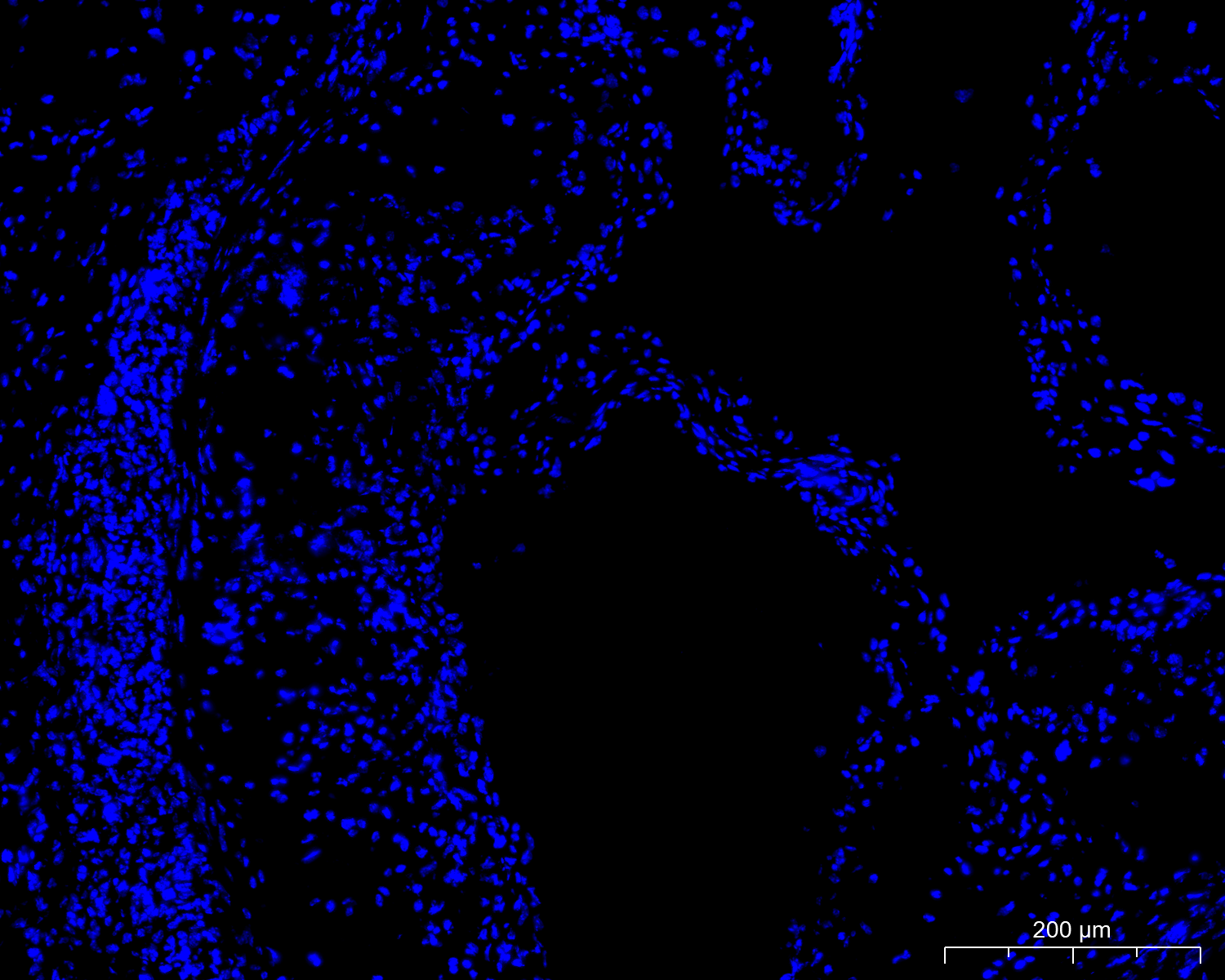

Supplement: S9 File — (ZIP) [file pone.0347758.s009.zip › 主动脉CD36/DAPI/PSB-H/93 CD36绿_20.0x.tif]

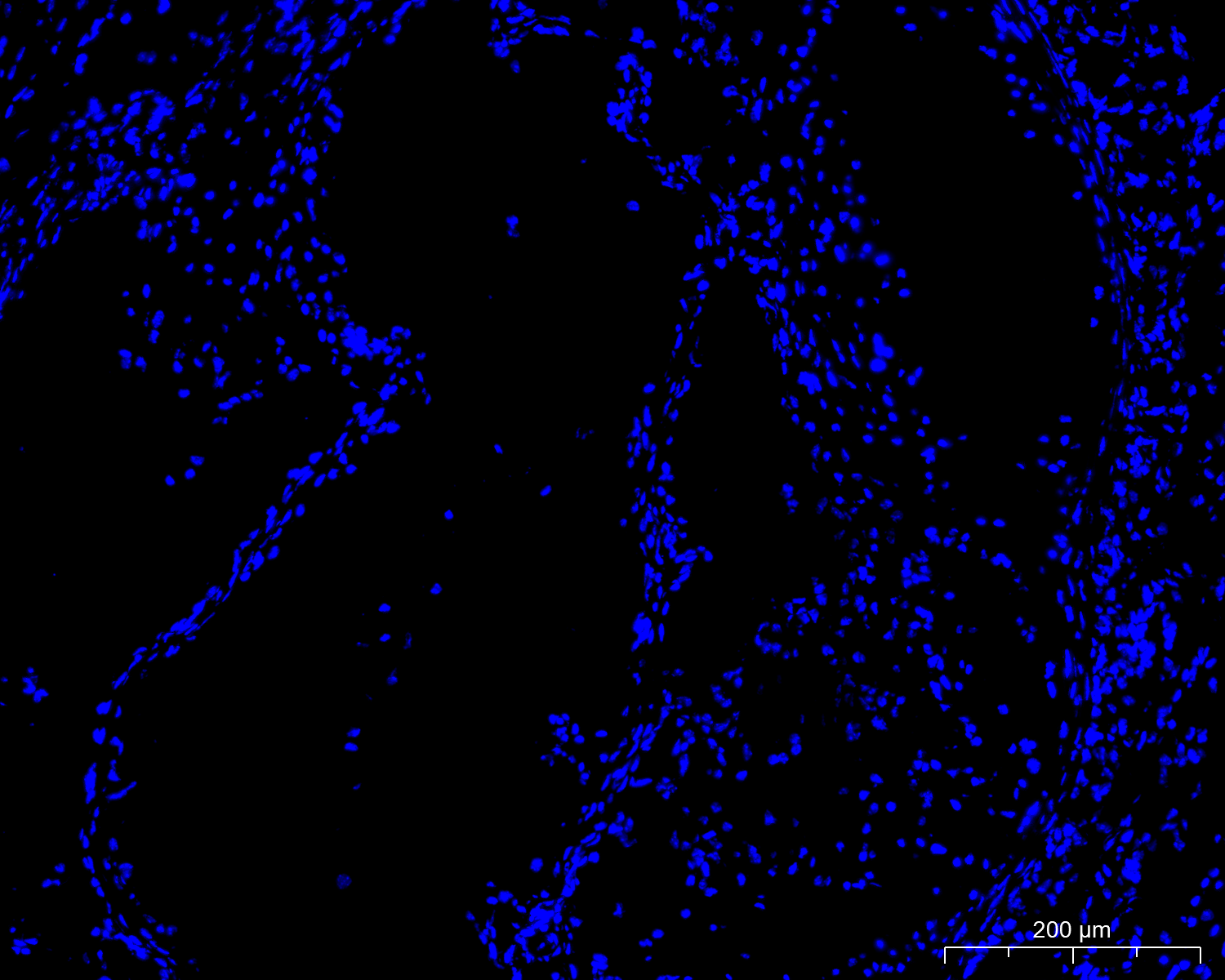

Supplement: S9 File — (ZIP) [file pone.0347758.s009.zip › 主动脉CD36/DAPI/PSB-H/98 CD36绿_20.0x.tif]

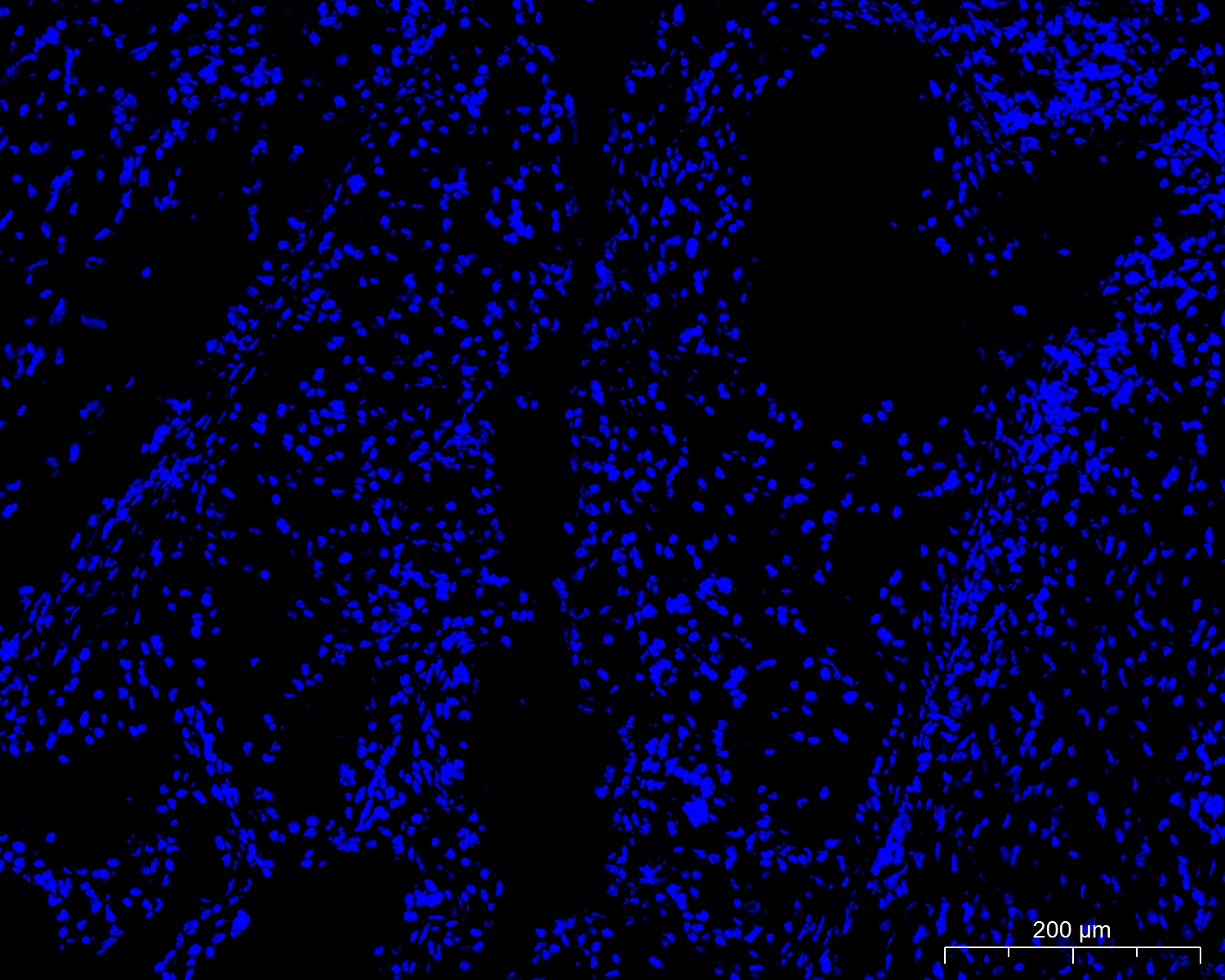

Supplement: S9 File — (ZIP) [file pone.0347758.s009.zip › 主动脉CD36/DAPI/PSB-H/A1 CD36绿_20.0x.tif]

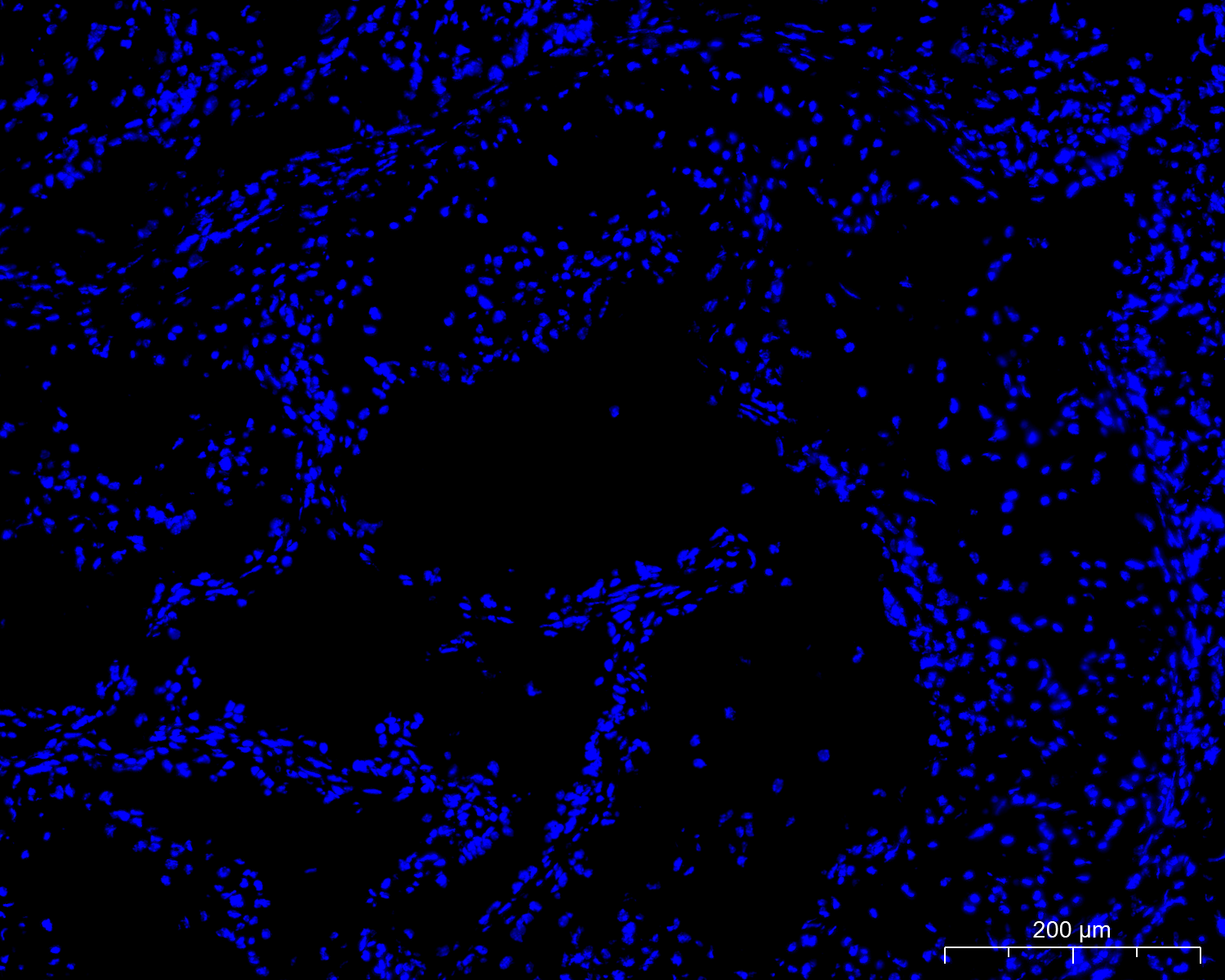

Supplement: S9 File — (ZIP) [file pone.0347758.s009.zip › 主动脉CD36/DAPI/PSB-L/74 CD36绿_20.0x.tif]

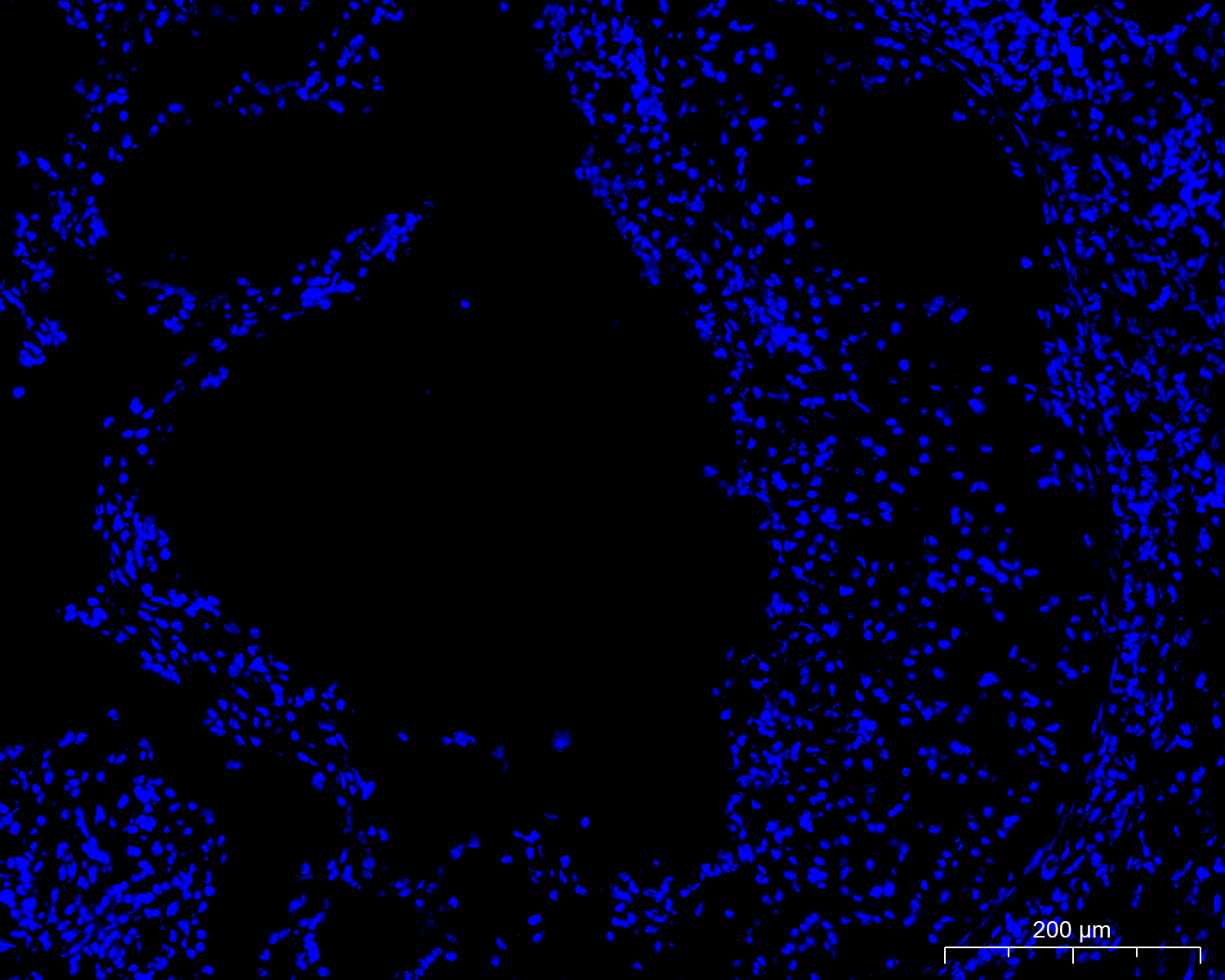

Supplement: S9 File — (ZIP) [file pone.0347758.s009.zip › 主动脉CD36/DAPI/PSB-L/77 CD36绿_20.0x.tif]

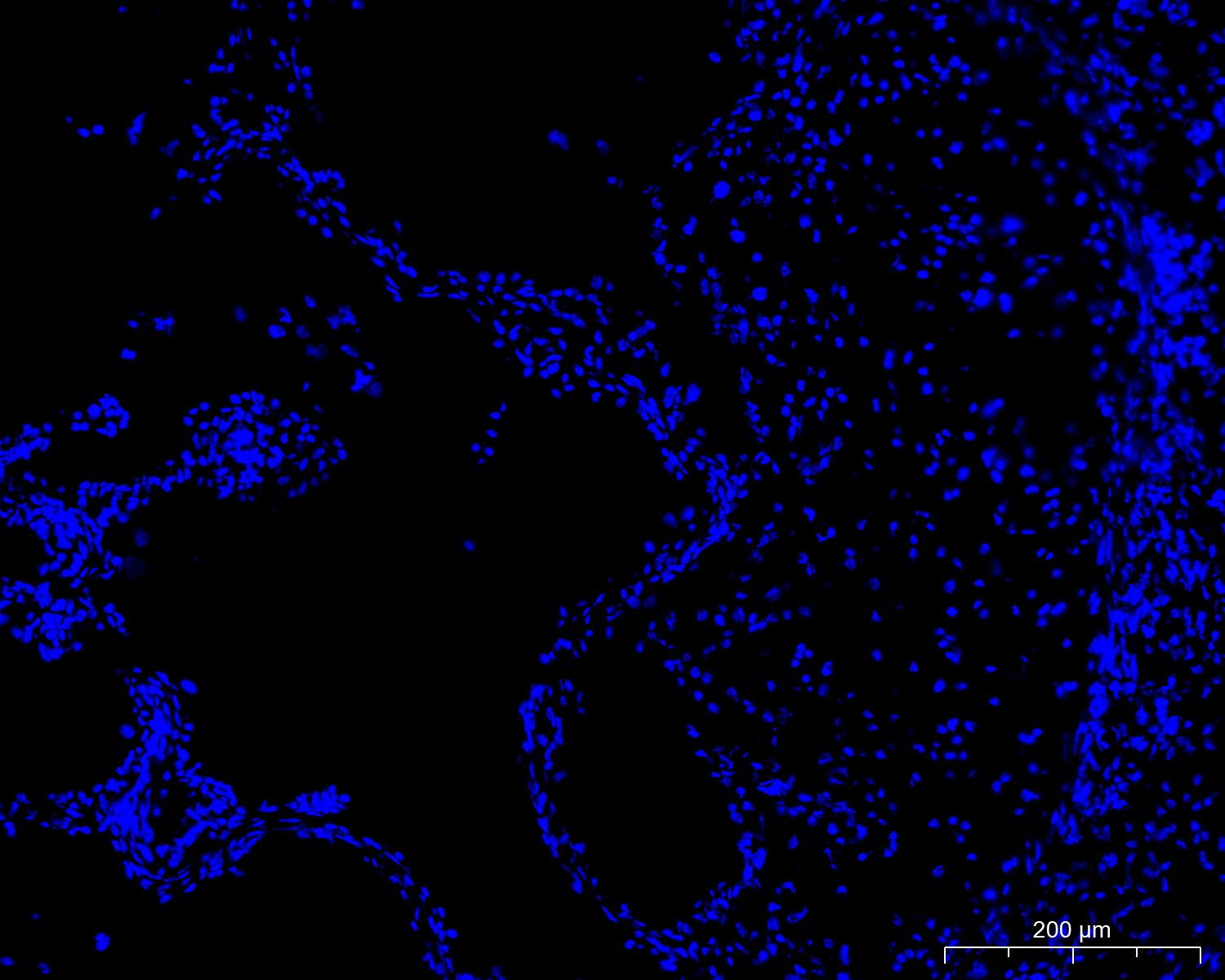

Supplement: S9 File — (ZIP) [file pone.0347758.s009.zip › 主动脉CD36/DAPI/PSB-L/80 CD36绿_20.0x.tif]

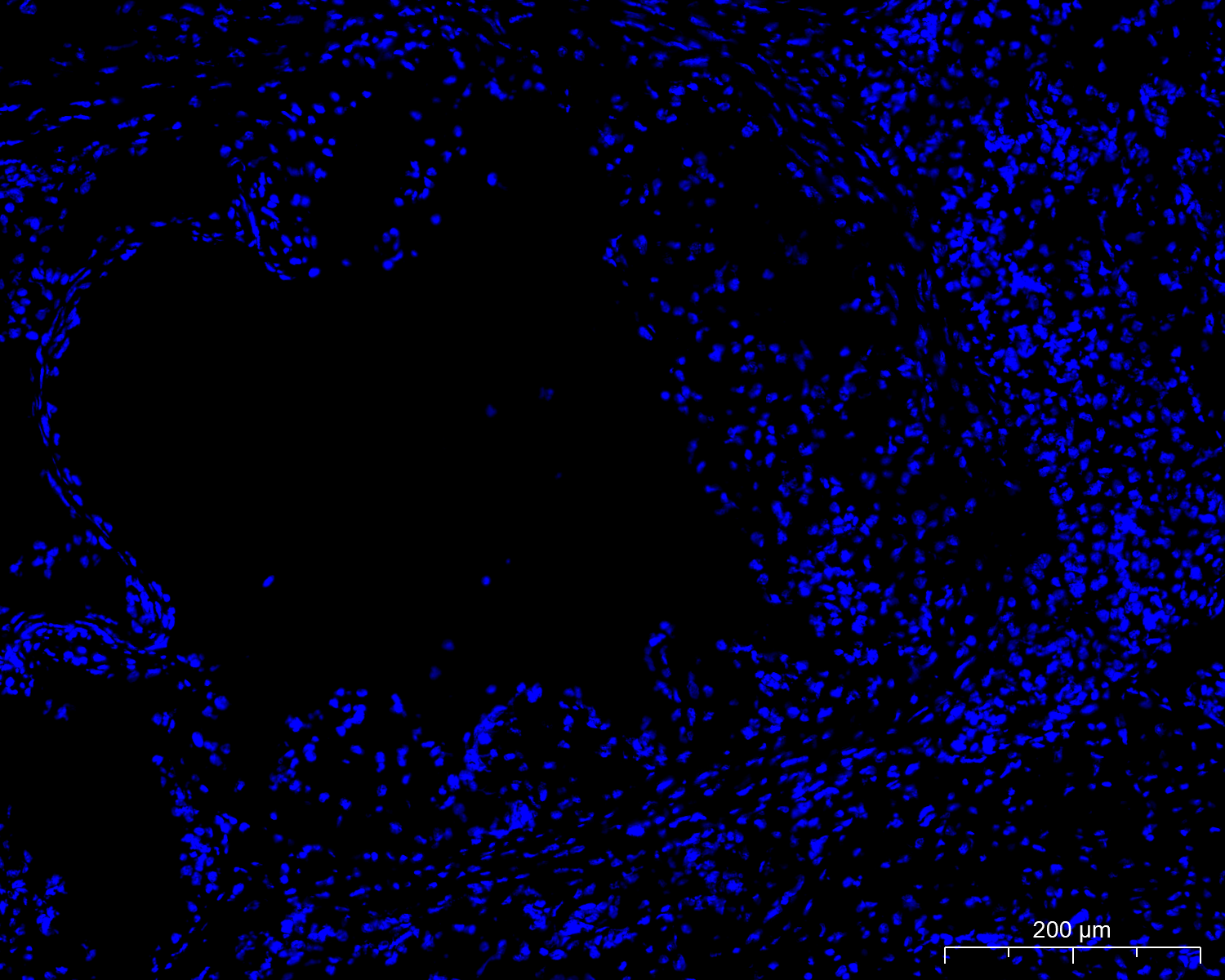

Supplement: S9 File — (ZIP) [file pone.0347758.s009.zip › 主动脉CD36/DAPI/PSB-L/82 CD36绿_20.0x.tif]

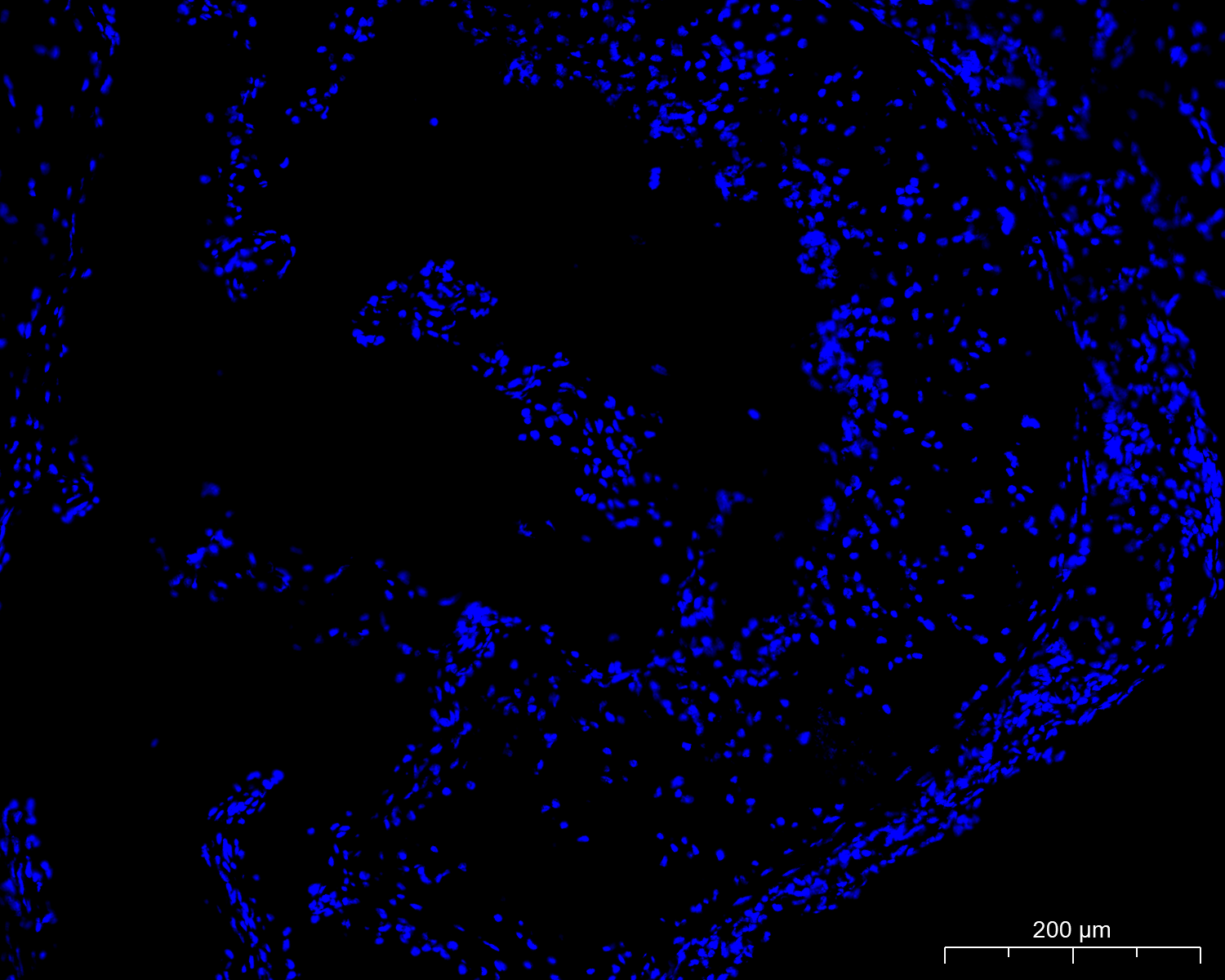

Supplement: S9 File — (ZIP) [file pone.0347758.s009.zip › 主动脉CD36/DAPI/PSB-M/85 CD36绿_20.0x.tif]

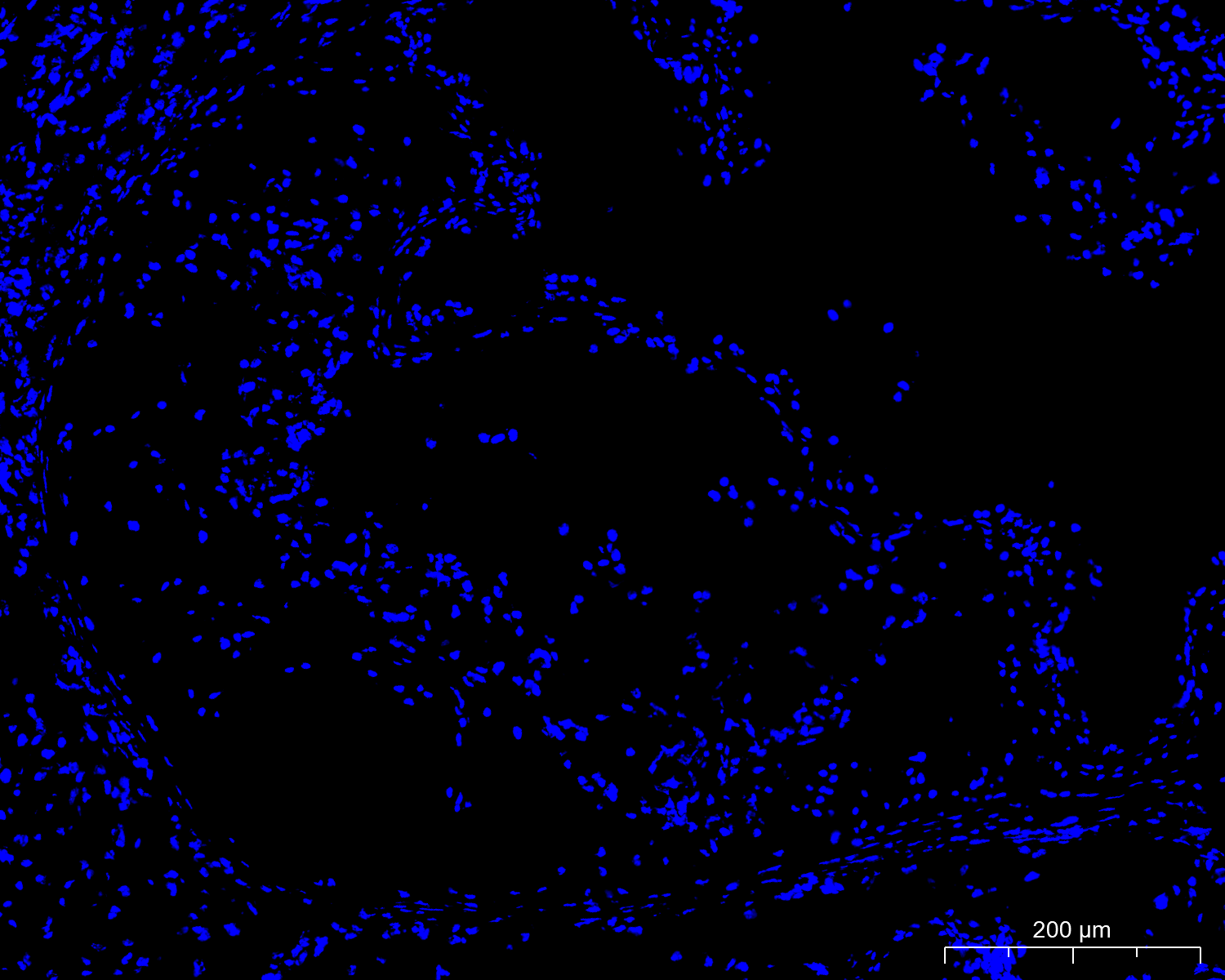

Supplement: S9 File — (ZIP) [file pone.0347758.s009.zip › 主动脉CD36/DAPI/PSB-M/89 CD36绿_20.0x.tif]
