# Supplementary figures and images for: Polyphenol extract of Syzygium brachythyrsum mitigates atherosclerosis in high-fat diet induced ApoE-/- mice by regulating ROS/Keap1/Nrf2 pathway (part 3 of 4)
Source: PLoS One. 2026 May 5;21(5):e0347758. doi: 10.1371/journal.pone.0347758 (PMC13143111; doi:10.1371/journal.pone.0347758)

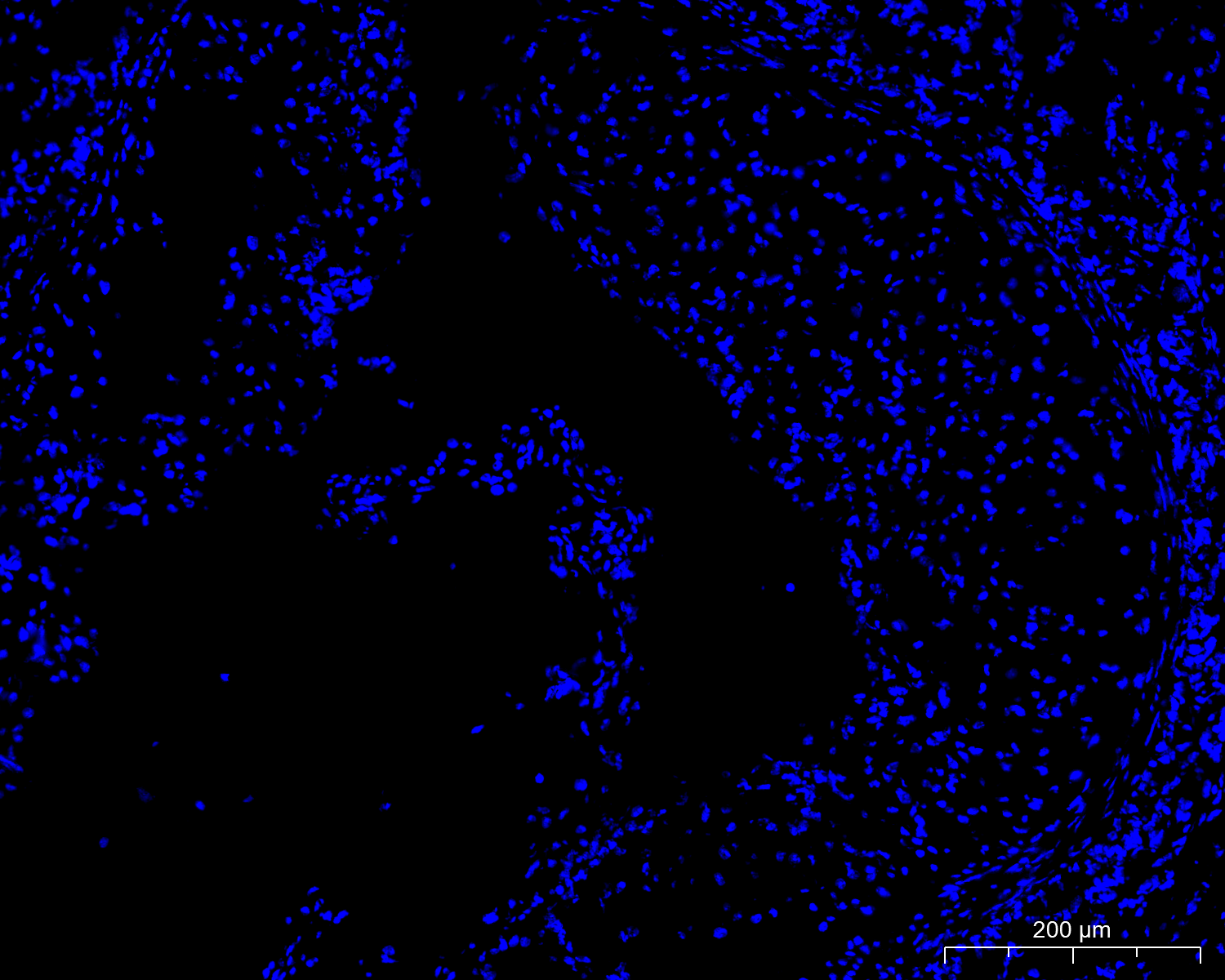

Supplement: S9 File — (ZIP) [file pone.0347758.s009.zip › 主动脉CD36/DAPI/PSB-M/90 CD36绿_20.0x.tif]

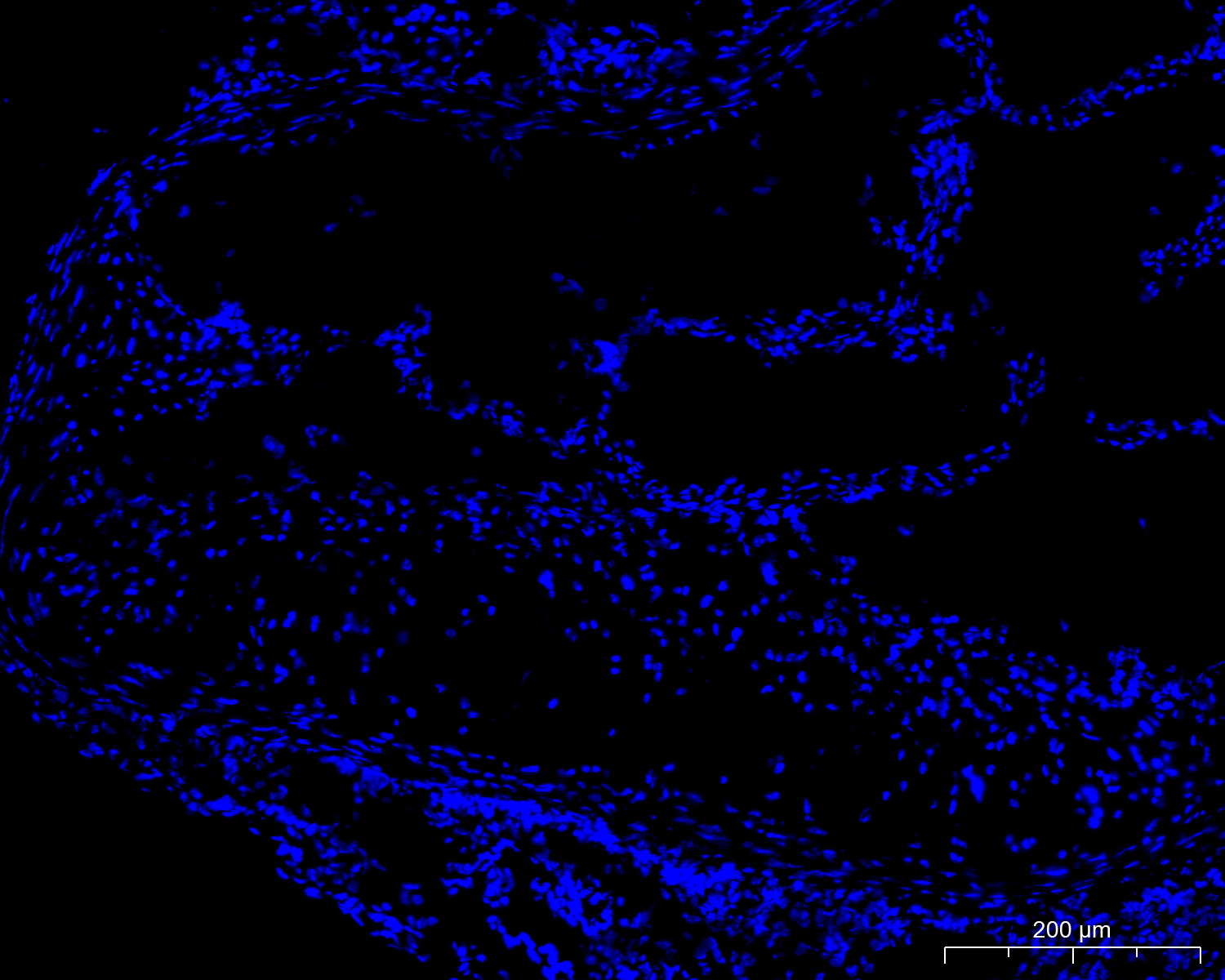

Supplement: S9 File — (ZIP) [file pone.0347758.s009.zip › 主动脉CD36/DAPI/PSB-M/92 CD36绿_20.0x.tif]

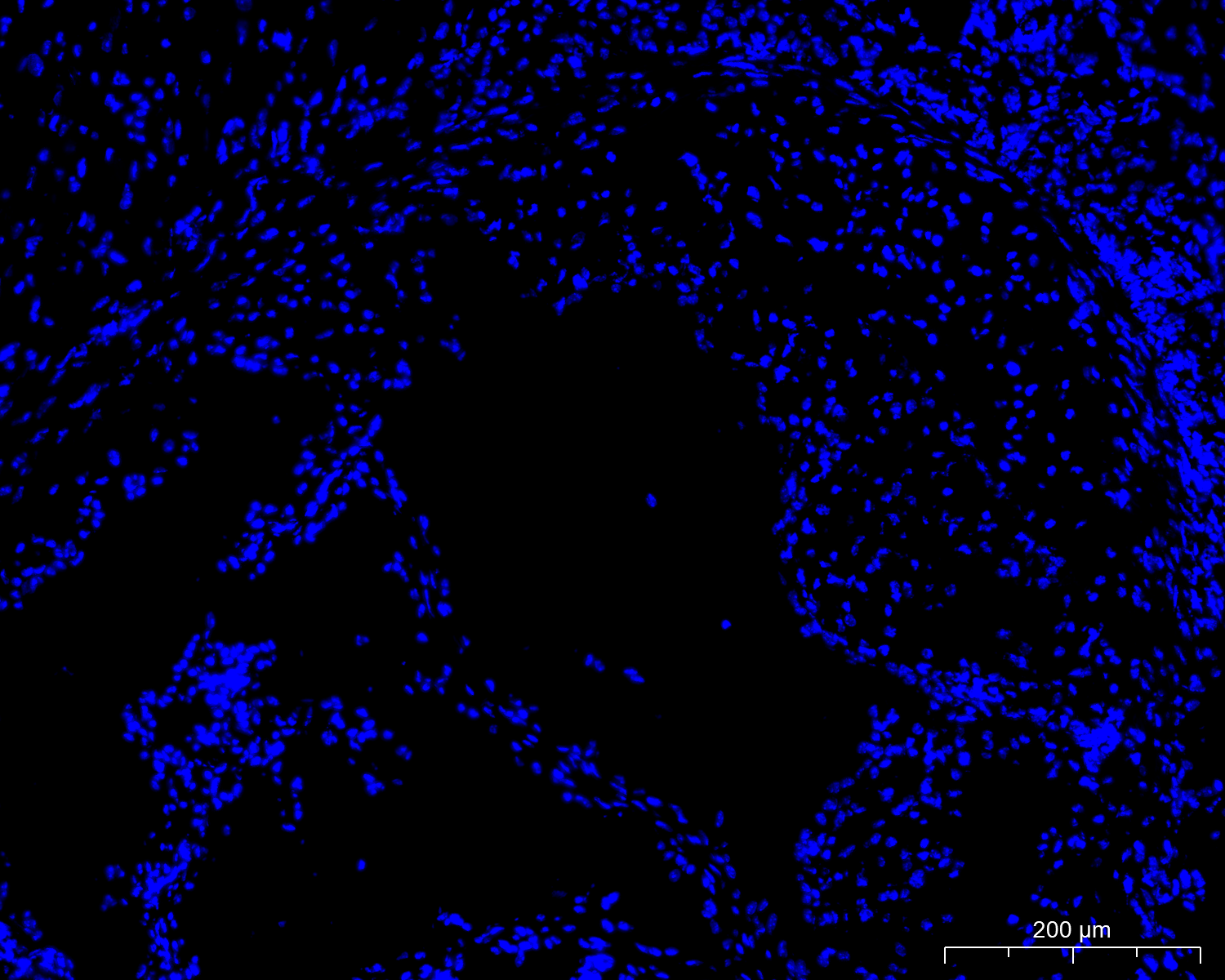

Supplement: S9 File — (ZIP) [file pone.0347758.s009.zip › 主动脉CD36/DAPI/statin/37 CD36绿_20.0x.tif]

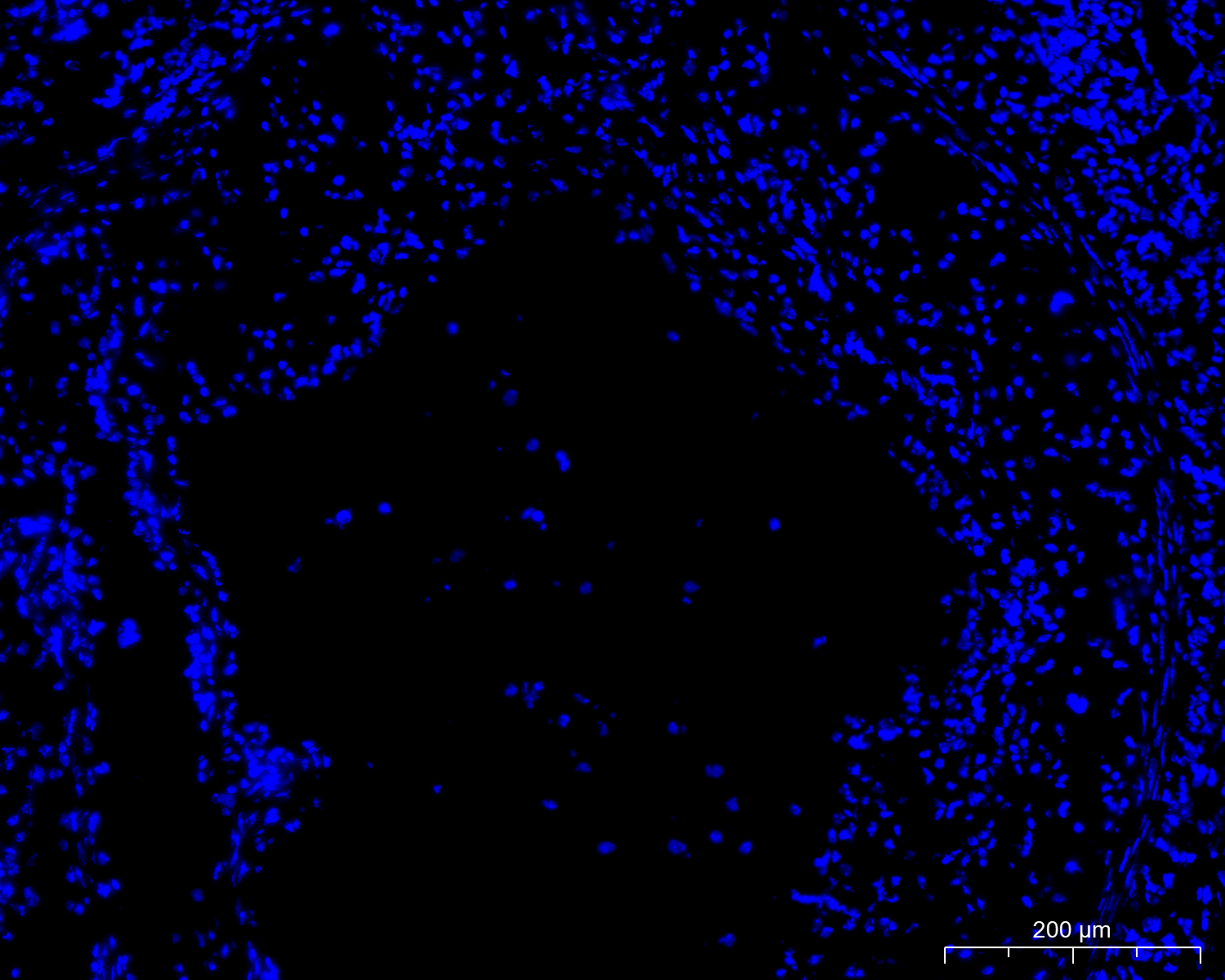

Supplement: S9 File — (ZIP) [file pone.0347758.s009.zip › 主动脉CD36/DAPI/statin/38 CD36绿_20.0x.tif]

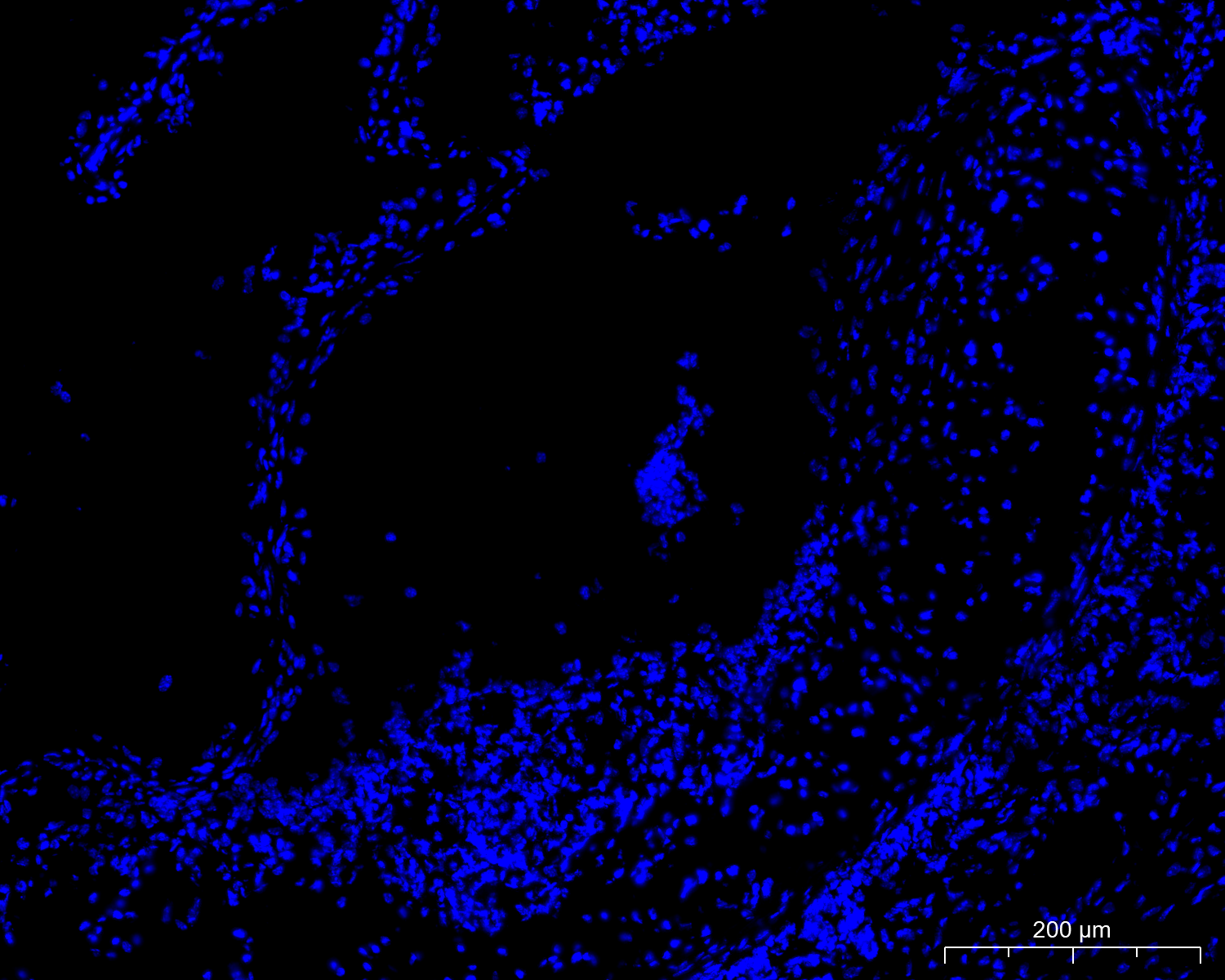

Supplement: S9 File — (ZIP) [file pone.0347758.s009.zip › 主动脉CD36/DAPI/statin/40 CD36绿_20.0x.tif]

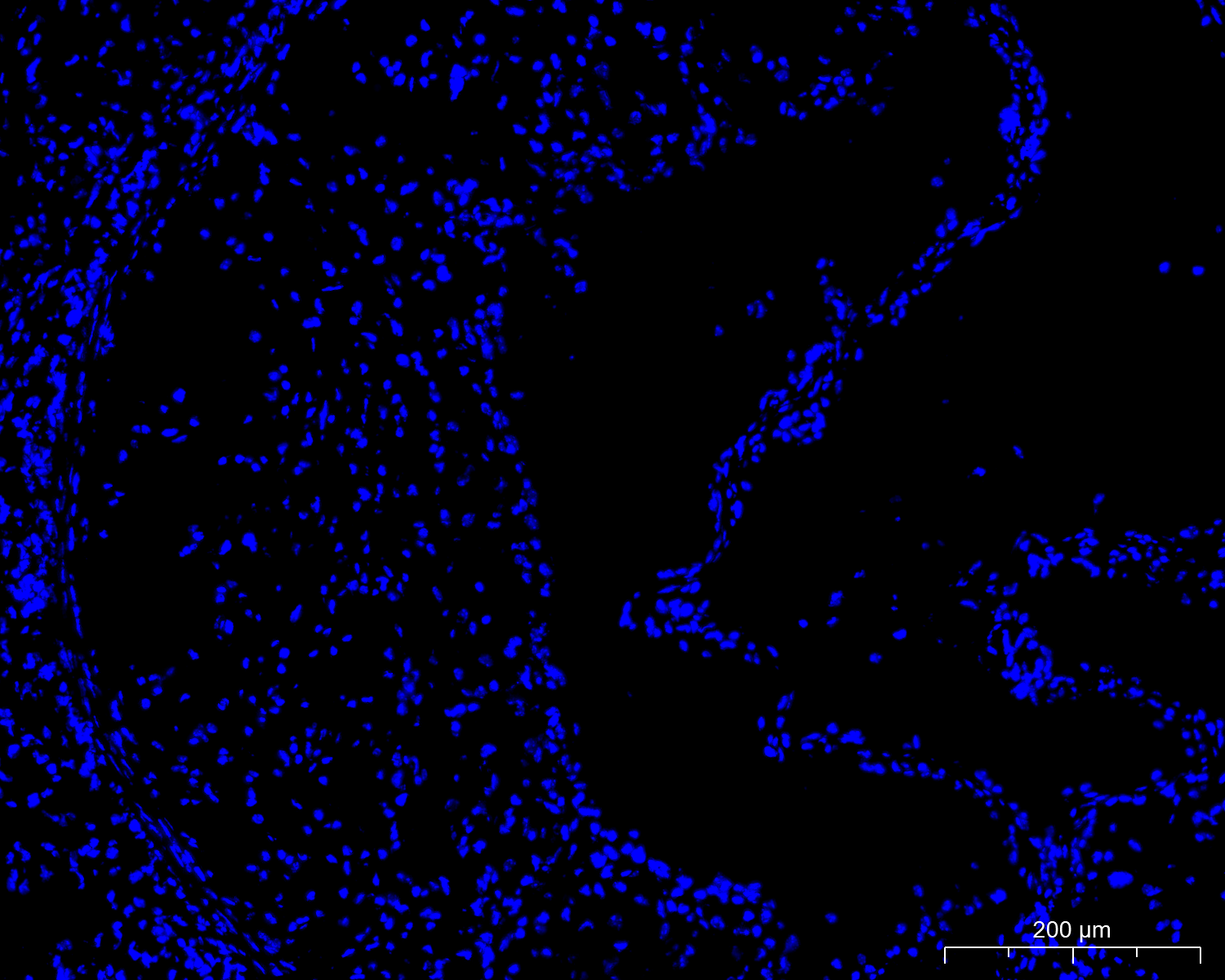

Supplement: S9 File — (ZIP) [file pone.0347758.s009.zip › 主动脉CD36/DAPI/statin/41 CD36绿_20.0x.tif]

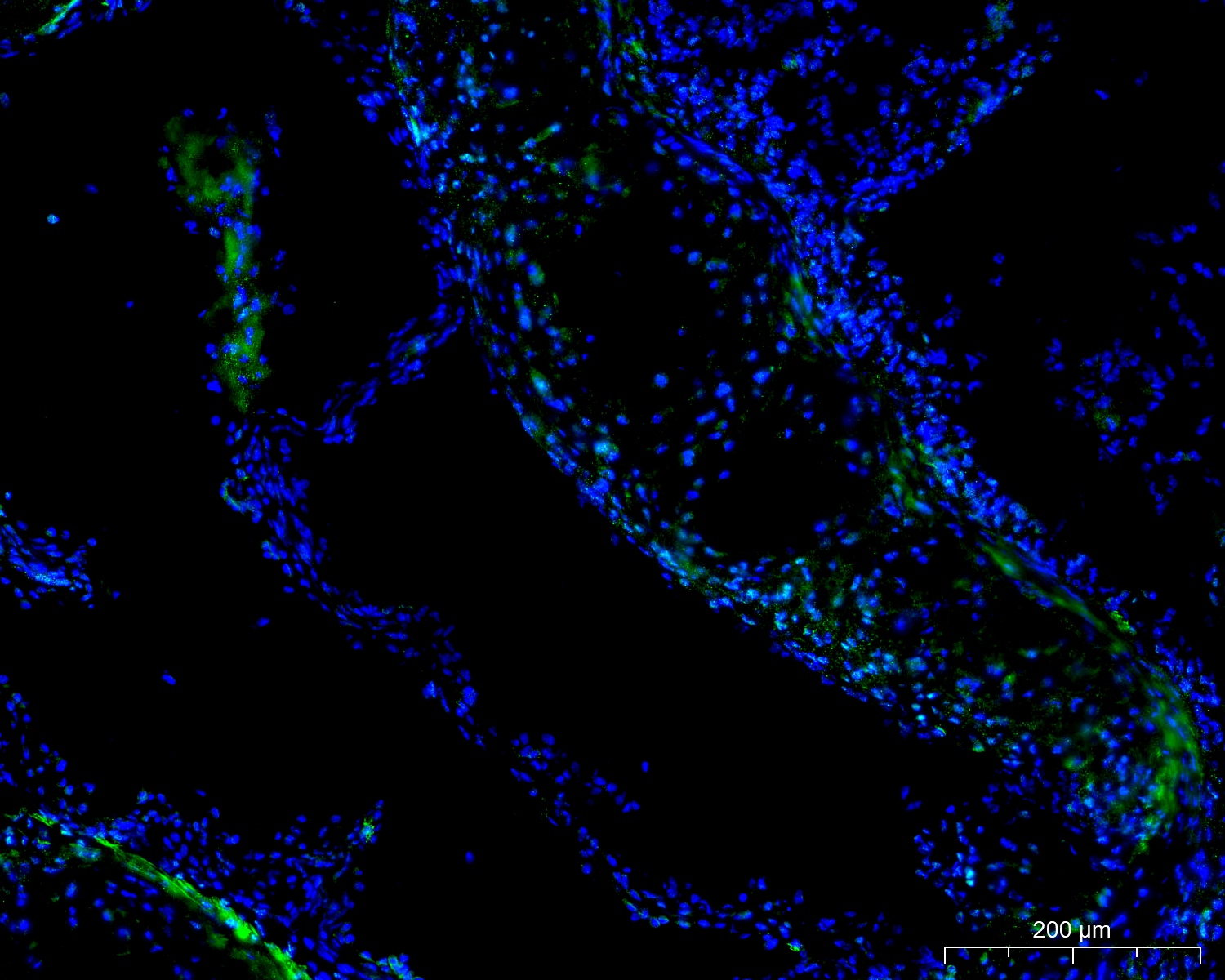

Supplement: S9 File — (ZIP) [file pone.0347758.s009.zip › 主动脉CD36/merge/AS/23 CD36绿_20.0x.tif]

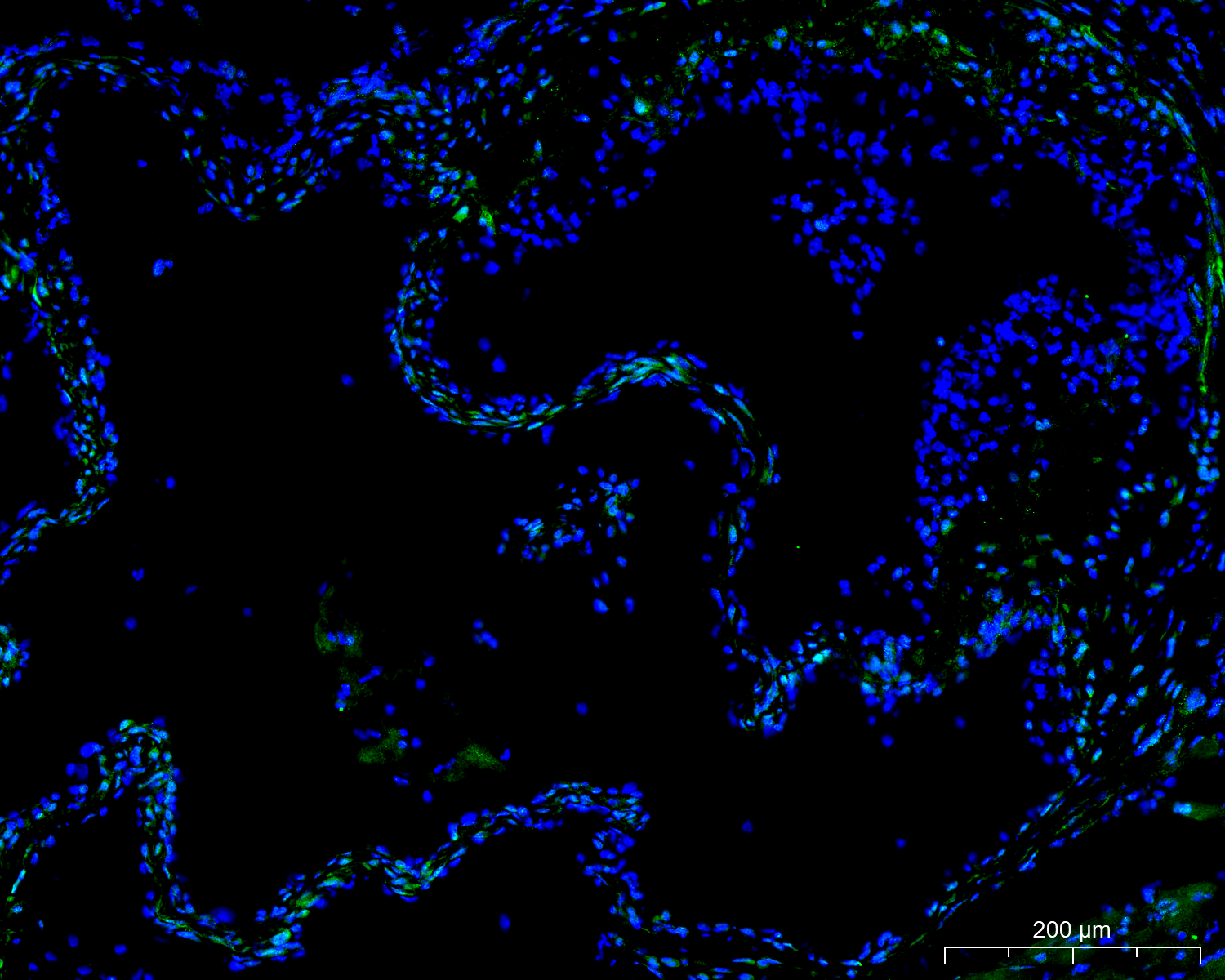

Supplement: S9 File — (ZIP) [file pone.0347758.s009.zip › 主动脉CD36/merge/AS/27 CD36绿_20.0x.tif]

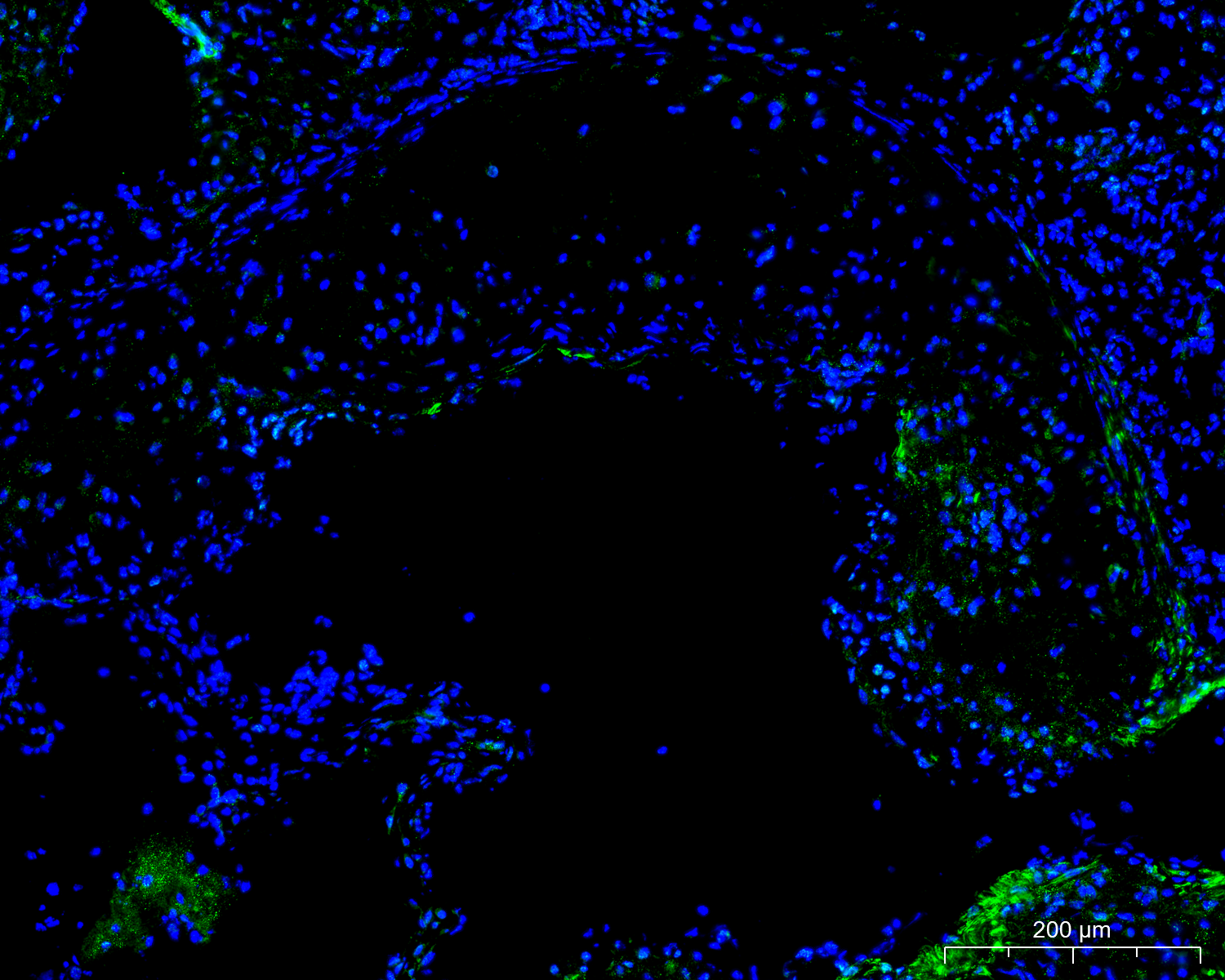

Supplement: S9 File — (ZIP) [file pone.0347758.s009.zip › 主动脉CD36/merge/AS/28 CD36绿_20.0x.tif]

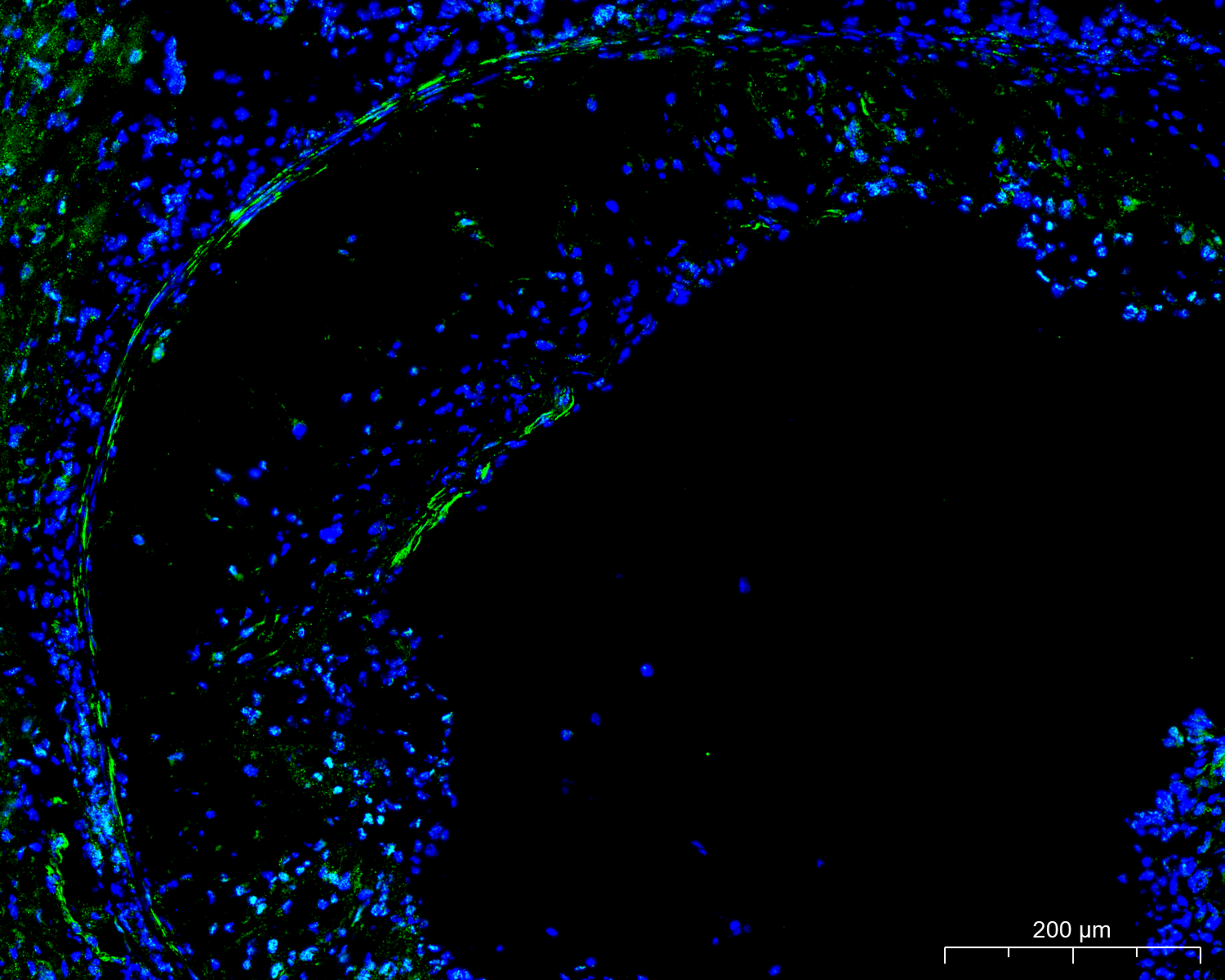

Supplement: S9 File — (ZIP) [file pone.0347758.s009.zip › 主动脉CD36/merge/AS/31 CD36绿_20.0x.tif]

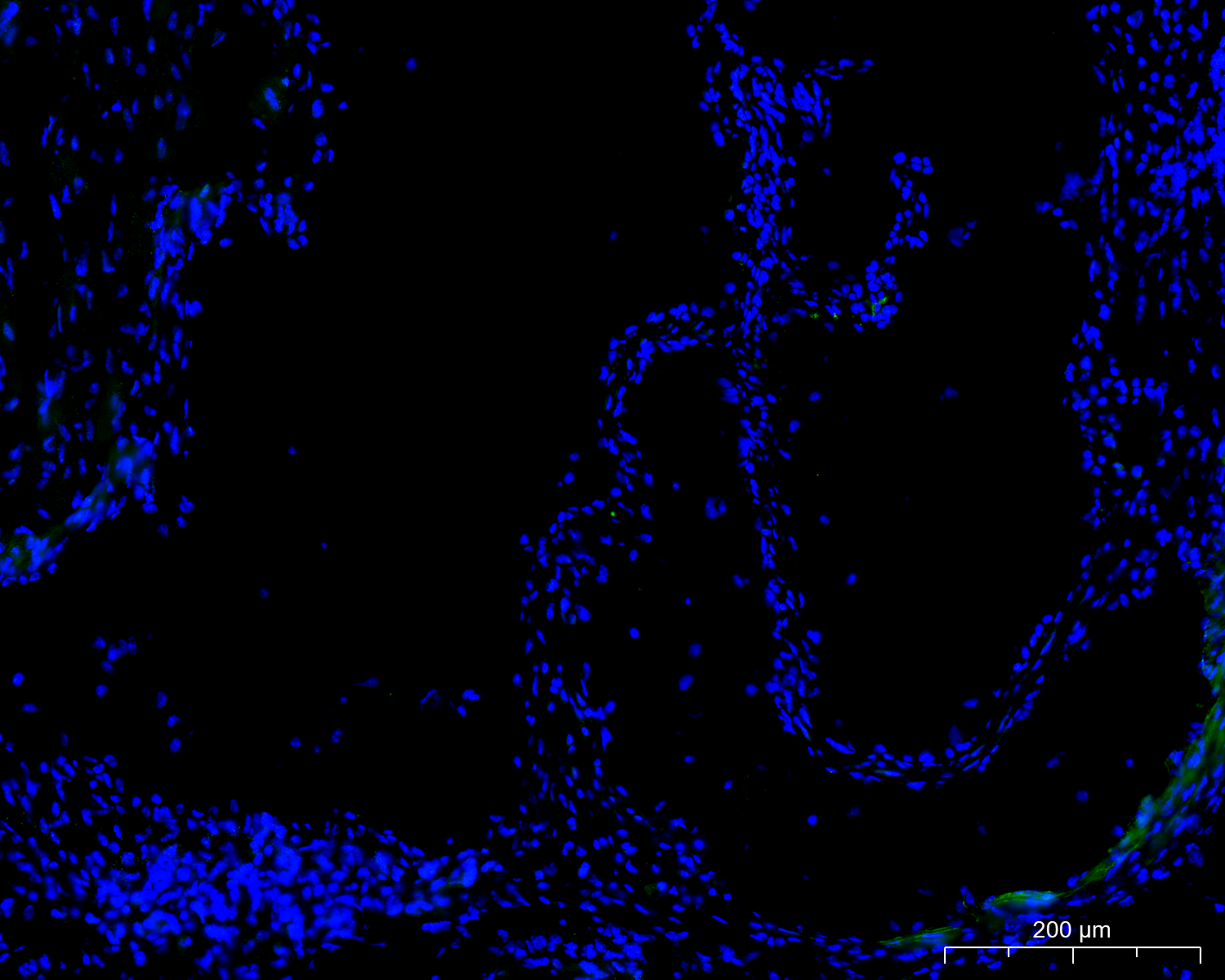

Supplement: S9 File — (ZIP) [file pone.0347758.s009.zip › 主动脉CD36/merge/control/1 CD36绿_20.0x.tif]

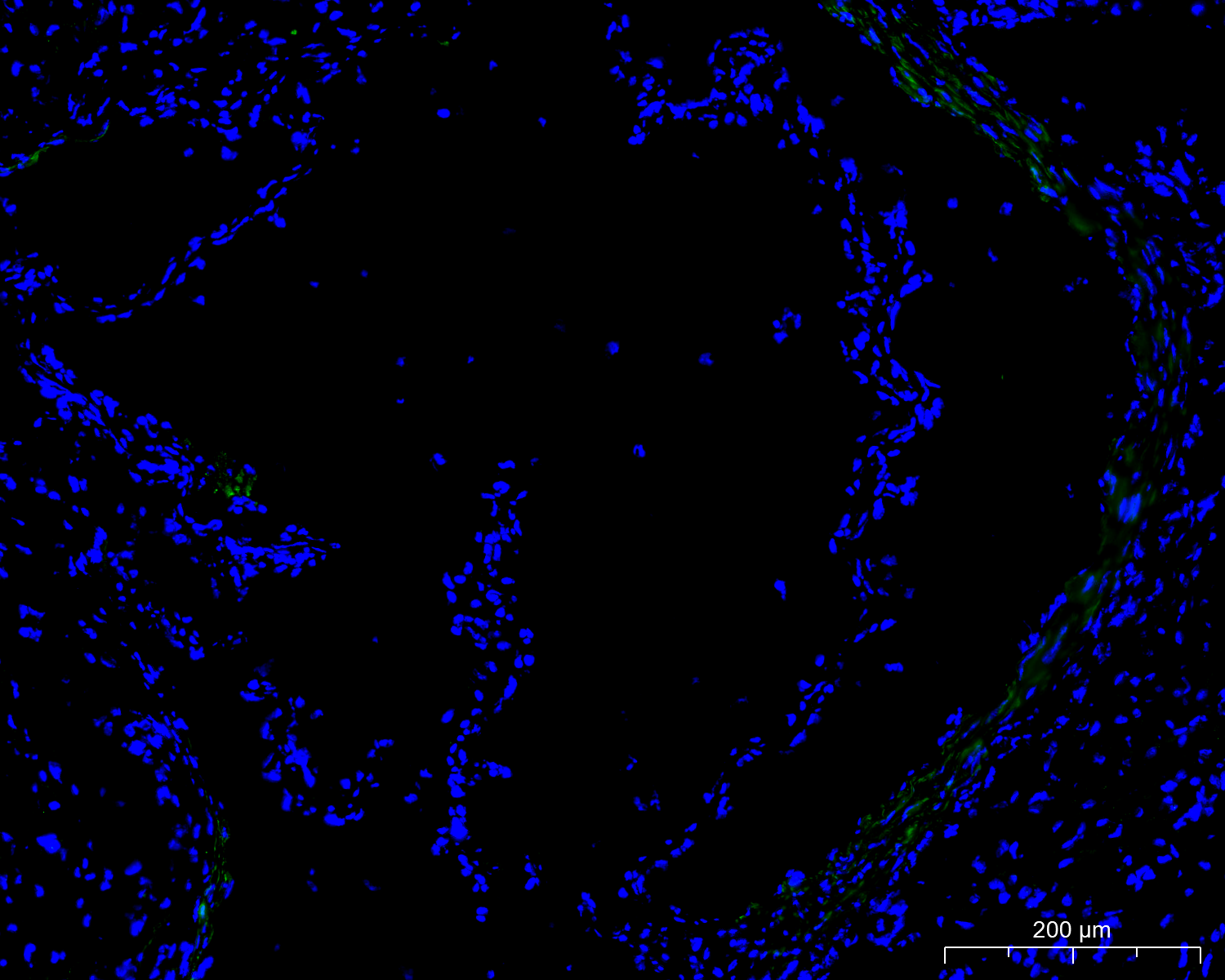

Supplement: S9 File — (ZIP) [file pone.0347758.s009.zip › 主动脉CD36/merge/control/2 CD36绿_20.0x.tif]

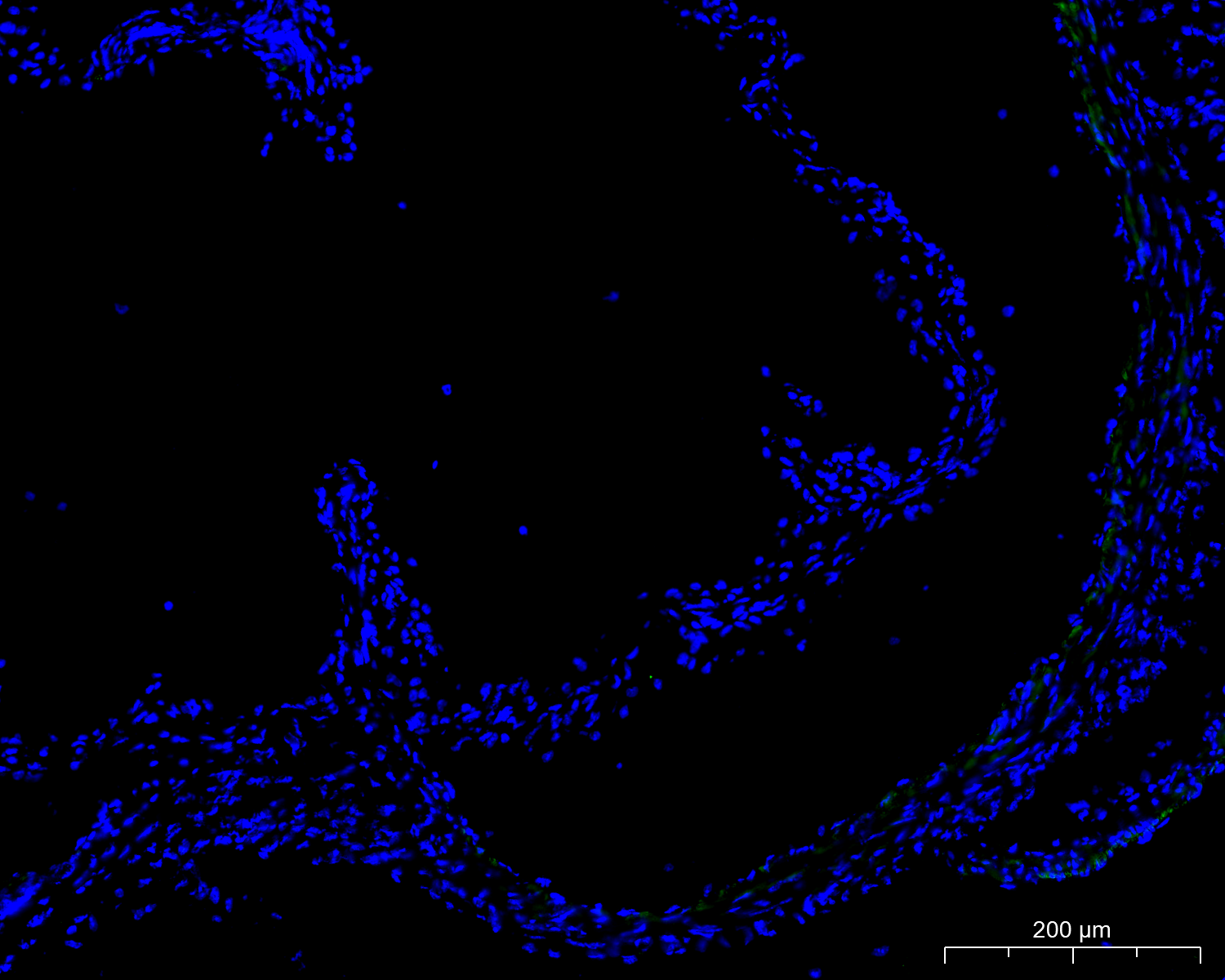

Supplement: S9 File — (ZIP) [file pone.0347758.s009.zip › 主动脉CD36/merge/control/6 CD36绿_20.0x.tif]

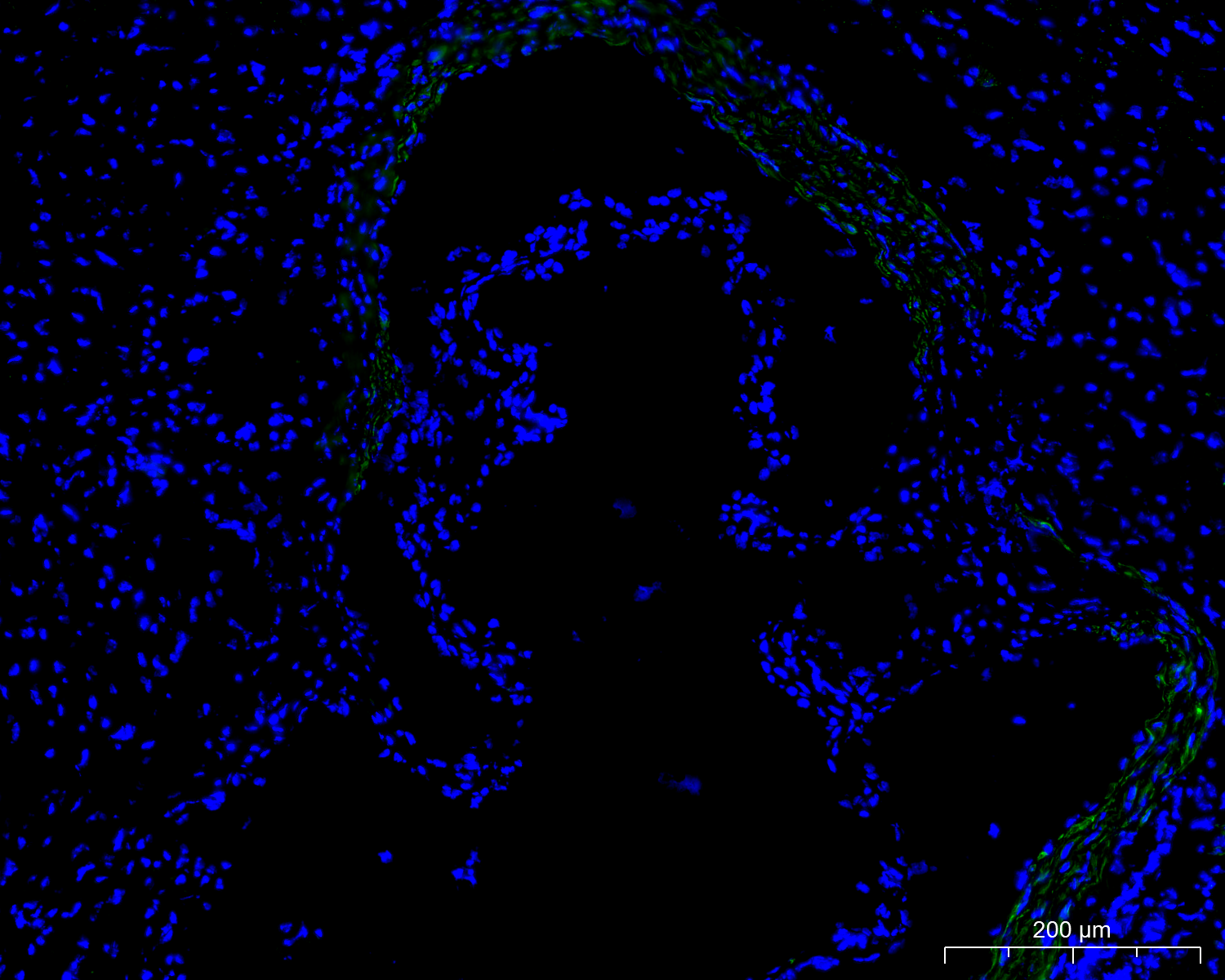

Supplement: S9 File — (ZIP) [file pone.0347758.s009.zip › 主动脉CD36/merge/control/7 CD36绿_20.0x.tif]

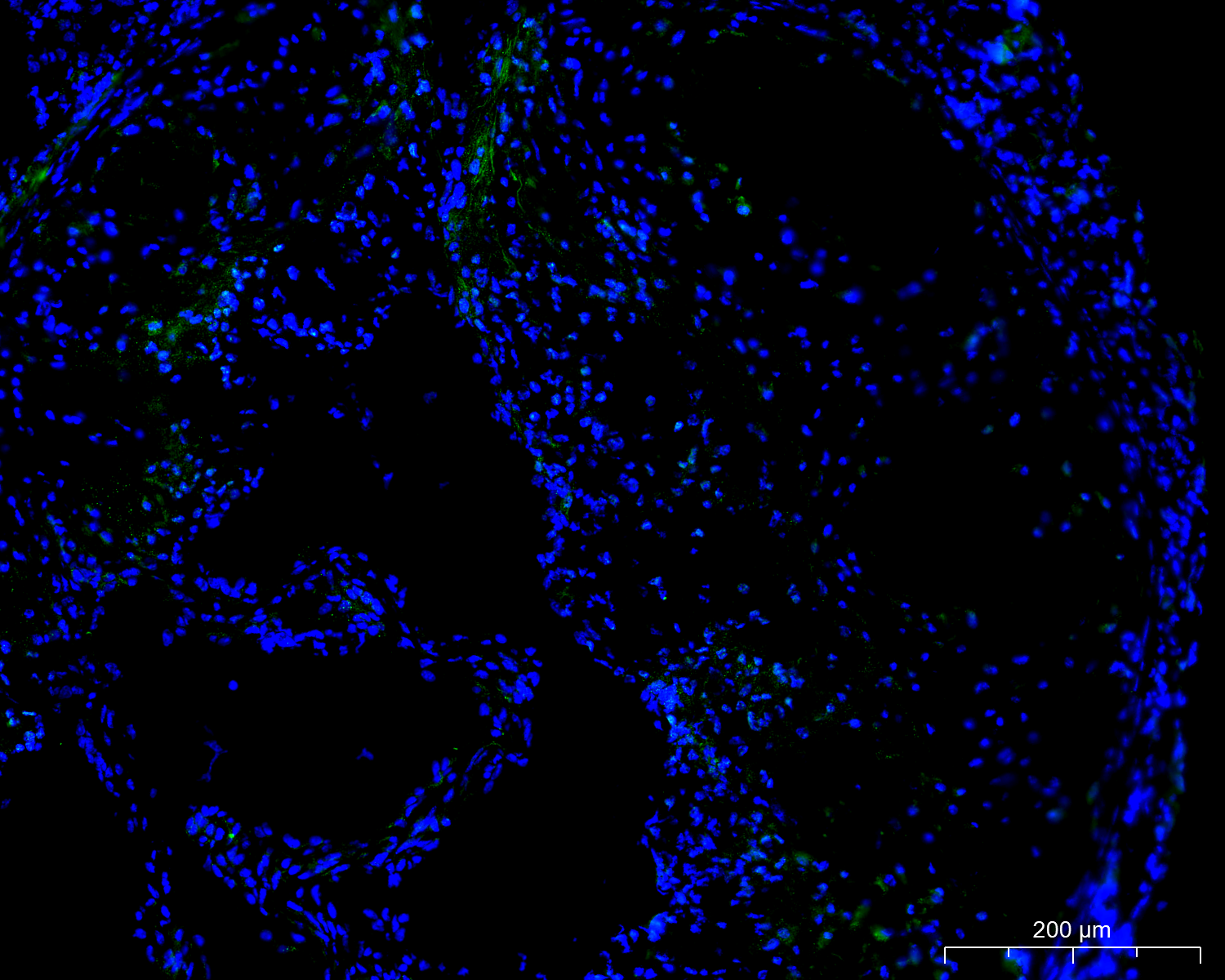

Supplement: S9 File — (ZIP) [file pone.0347758.s009.zip › 主动脉CD36/merge/PSB-H/100 CD36绿_20.0x.tif]

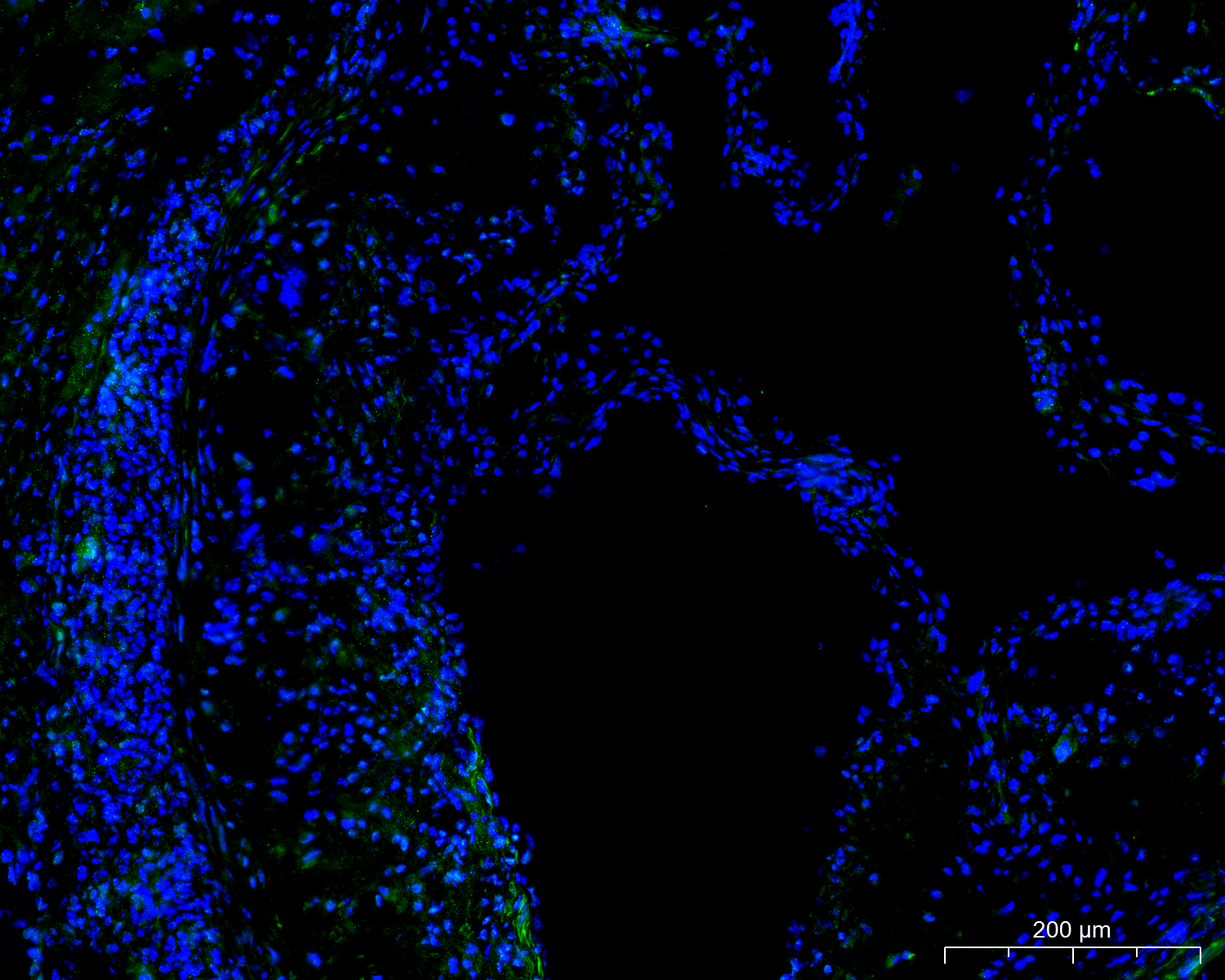

Supplement: S9 File — (ZIP) [file pone.0347758.s009.zip › 主动脉CD36/merge/PSB-H/93 CD36绿_20.0x.tif]

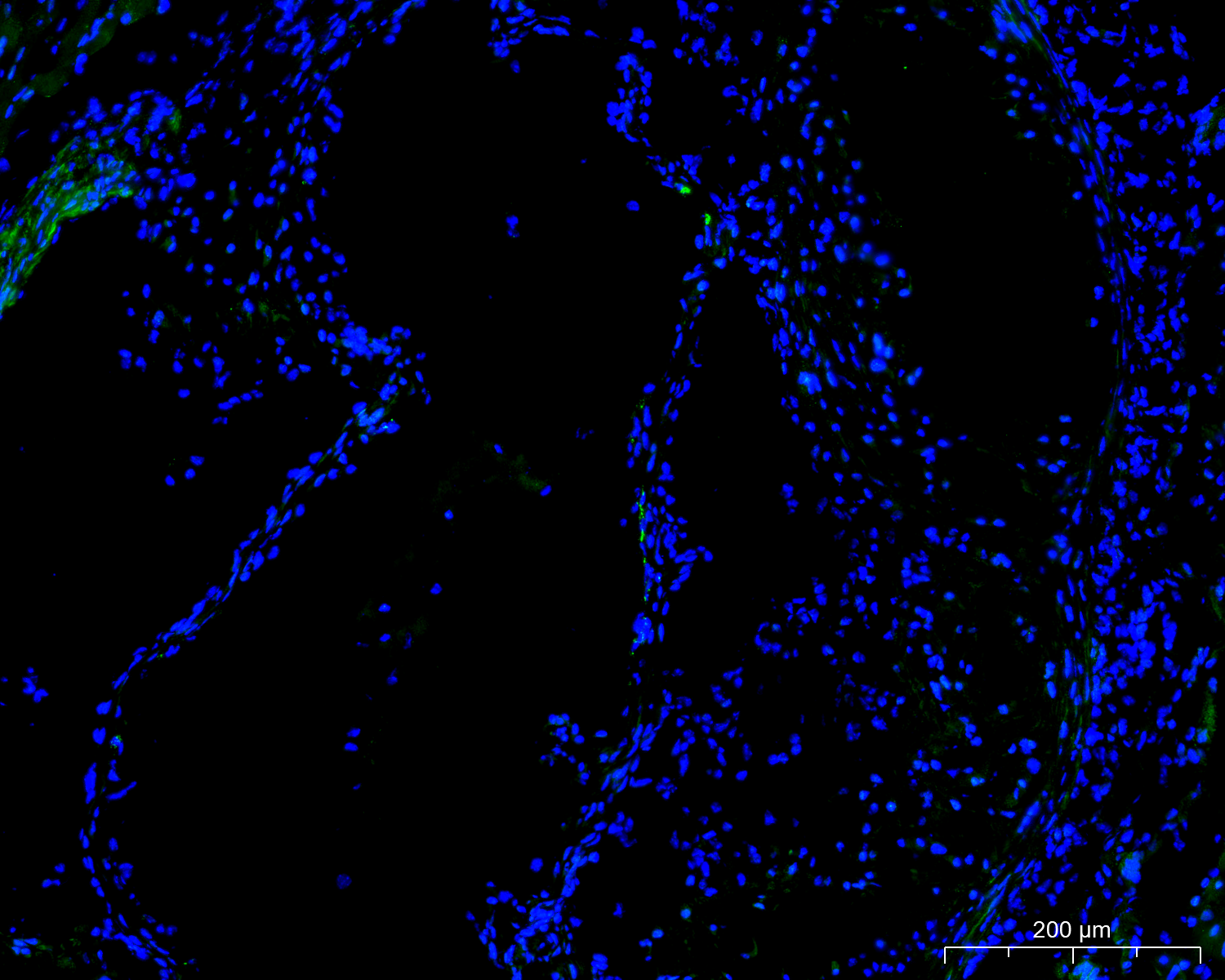

Supplement: S9 File — (ZIP) [file pone.0347758.s009.zip › 主动脉CD36/merge/PSB-H/98 CD36绿_20.0x.tif]

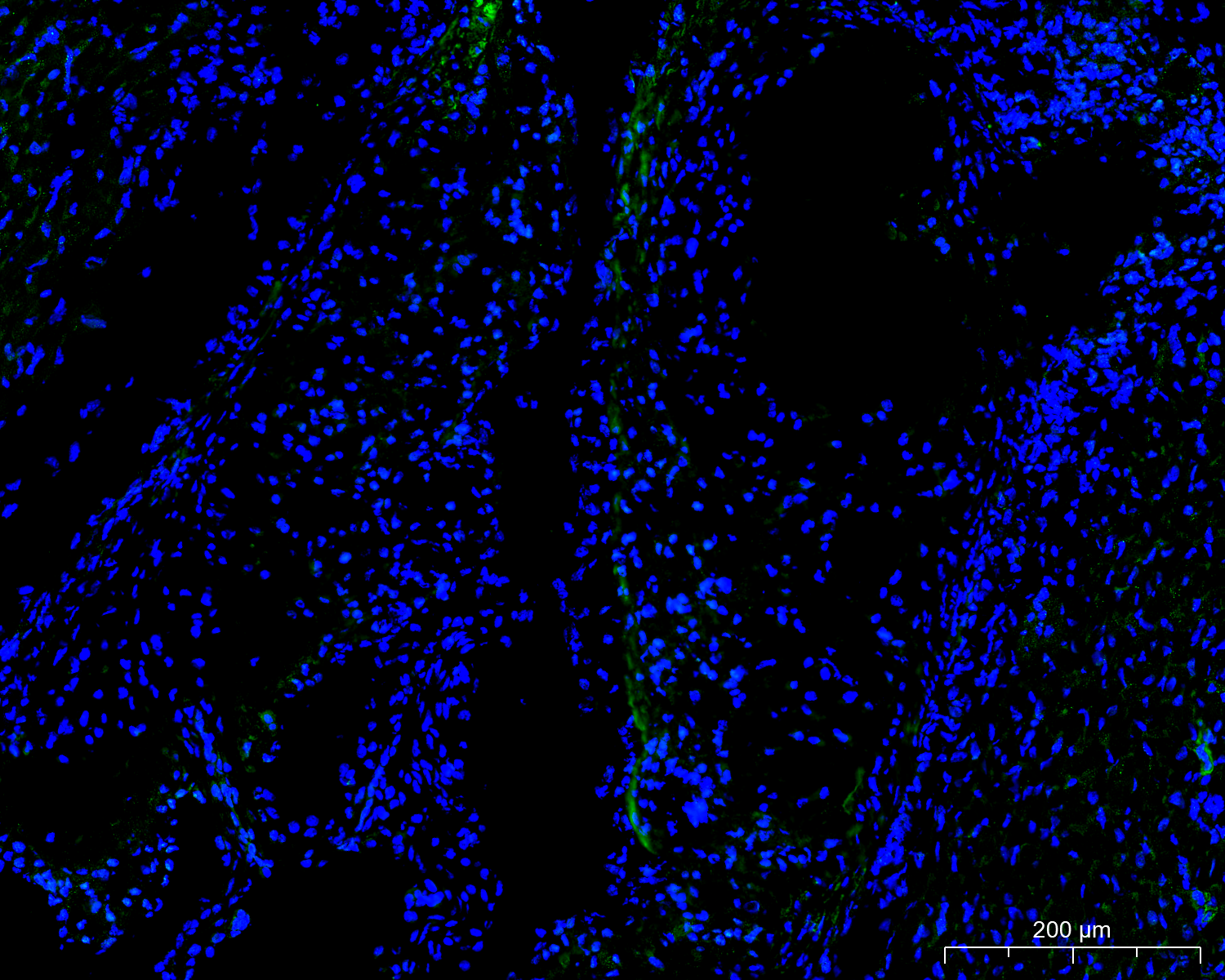

Supplement: S9 File — (ZIP) [file pone.0347758.s009.zip › 主动脉CD36/merge/PSB-H/A1 CD36绿_20.0x.tif]

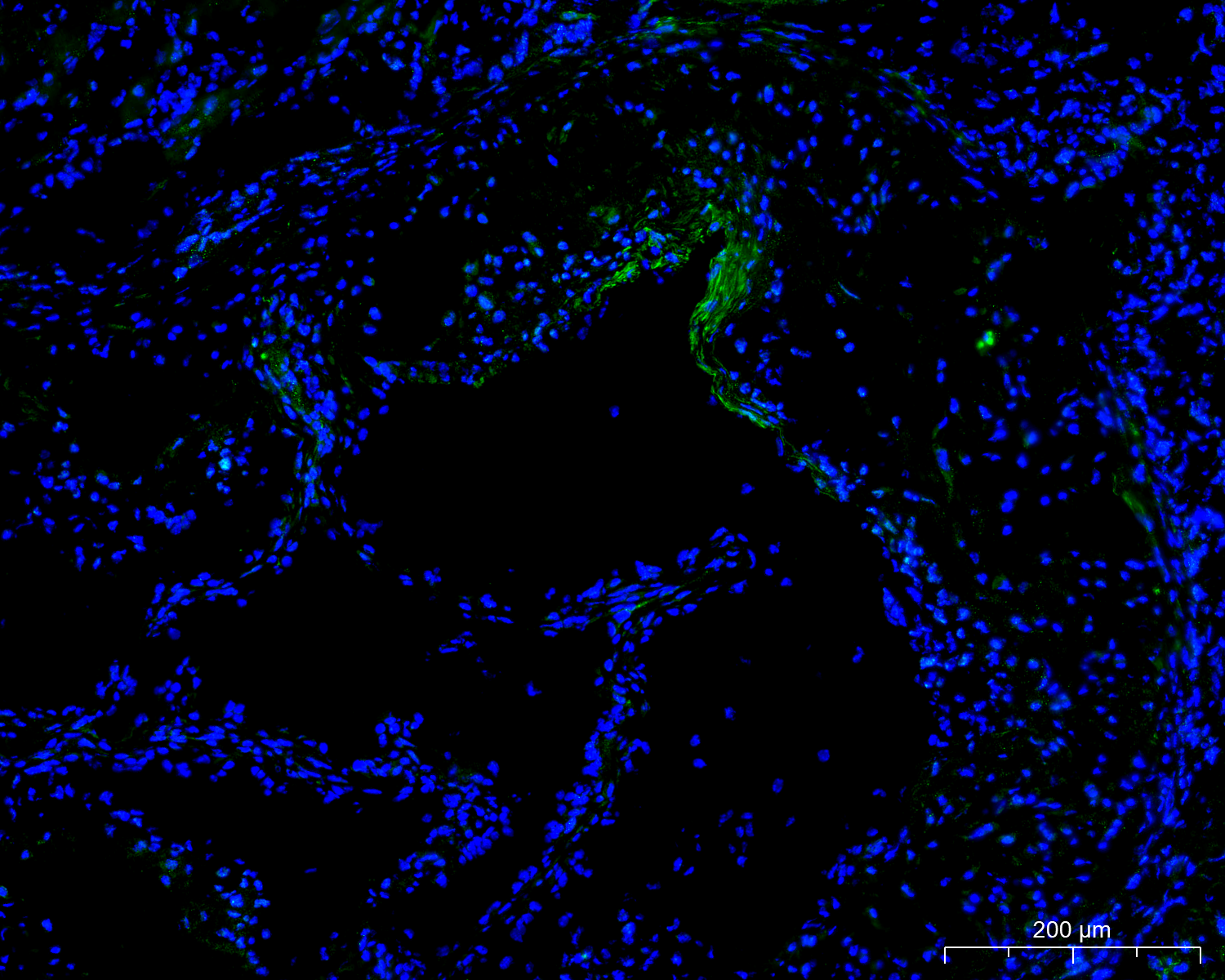

Supplement: S9 File — (ZIP) [file pone.0347758.s009.zip › 主动脉CD36/merge/PSB-L/74 CD36绿_20.0x.tif]

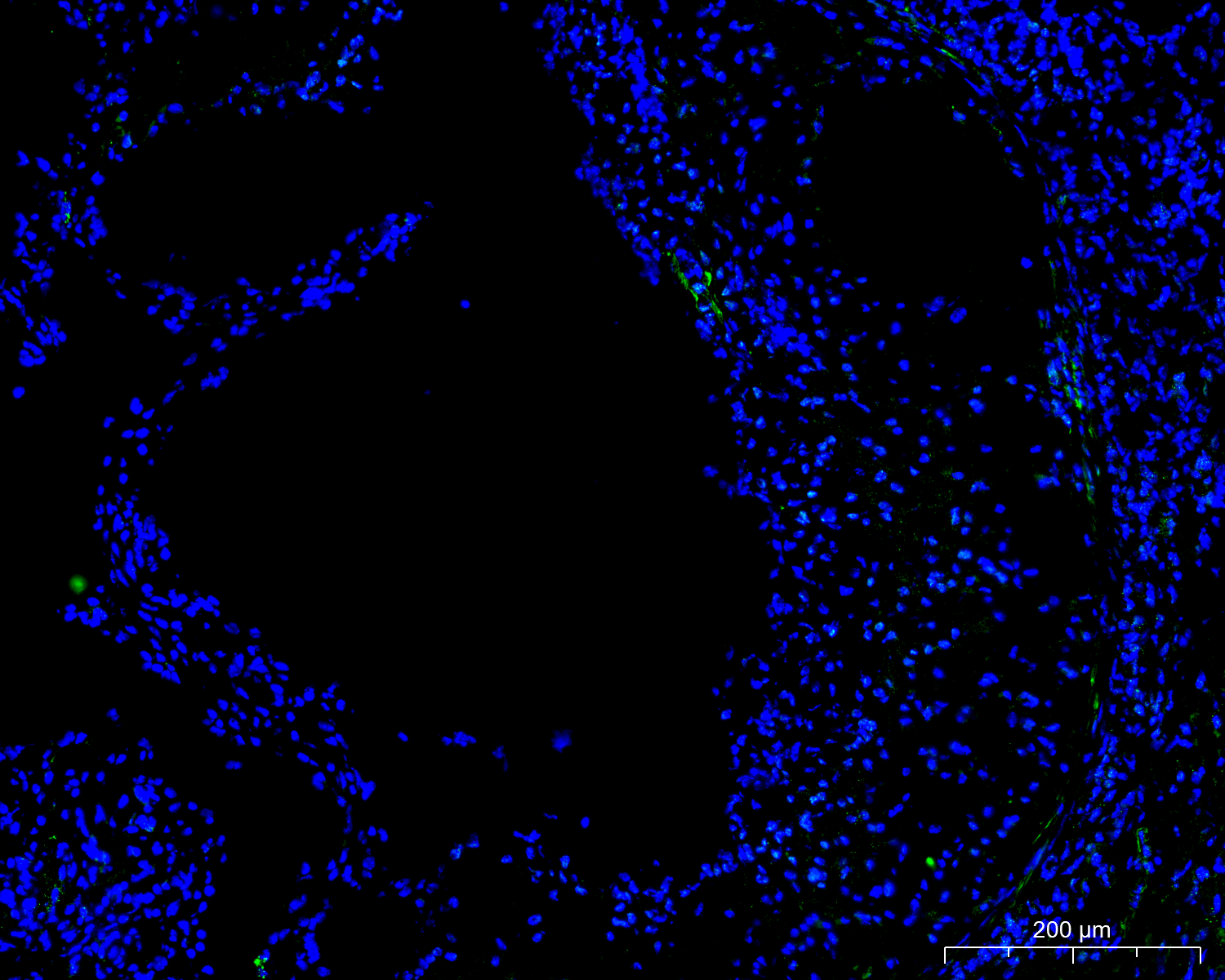

Supplement: S9 File — (ZIP) [file pone.0347758.s009.zip › 主动脉CD36/merge/PSB-L/77 CD36绿_20.0x.tif]

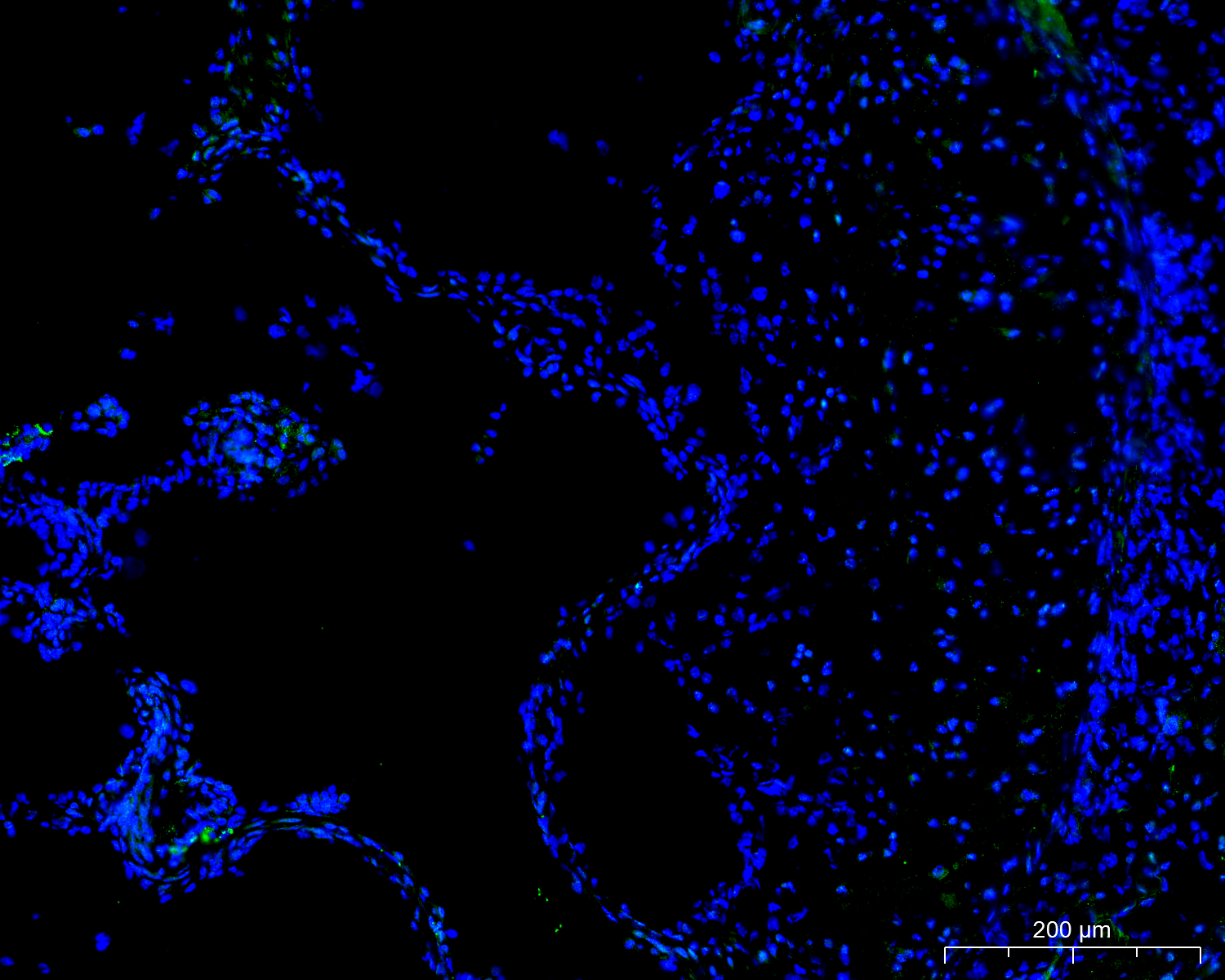

Supplement: S9 File — (ZIP) [file pone.0347758.s009.zip › 主动脉CD36/merge/PSB-L/80 CD36绿_20.0x.tif]

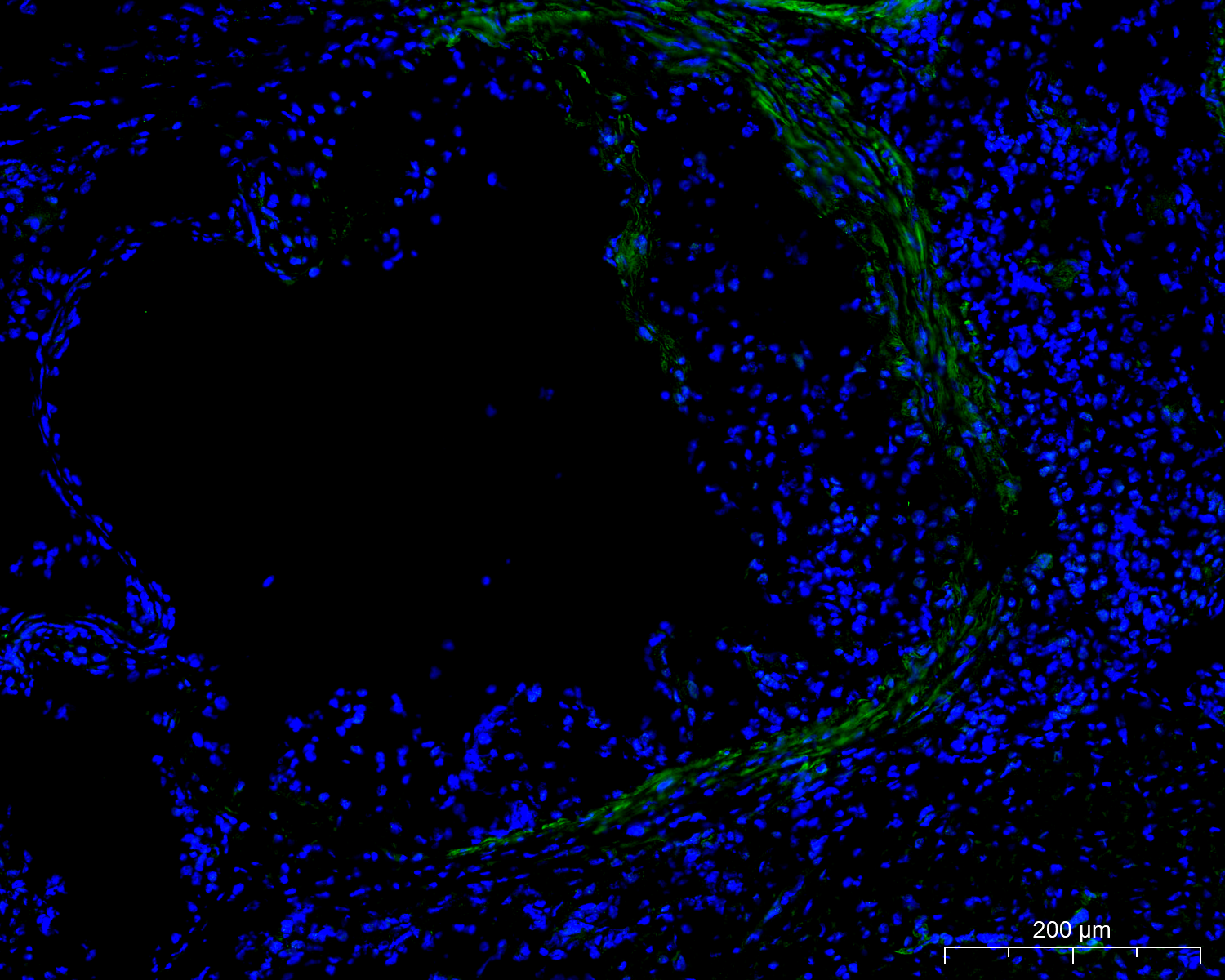

Supplement: S9 File — (ZIP) [file pone.0347758.s009.zip › 主动脉CD36/merge/PSB-L/82 CD36绿_20.0x.tif]

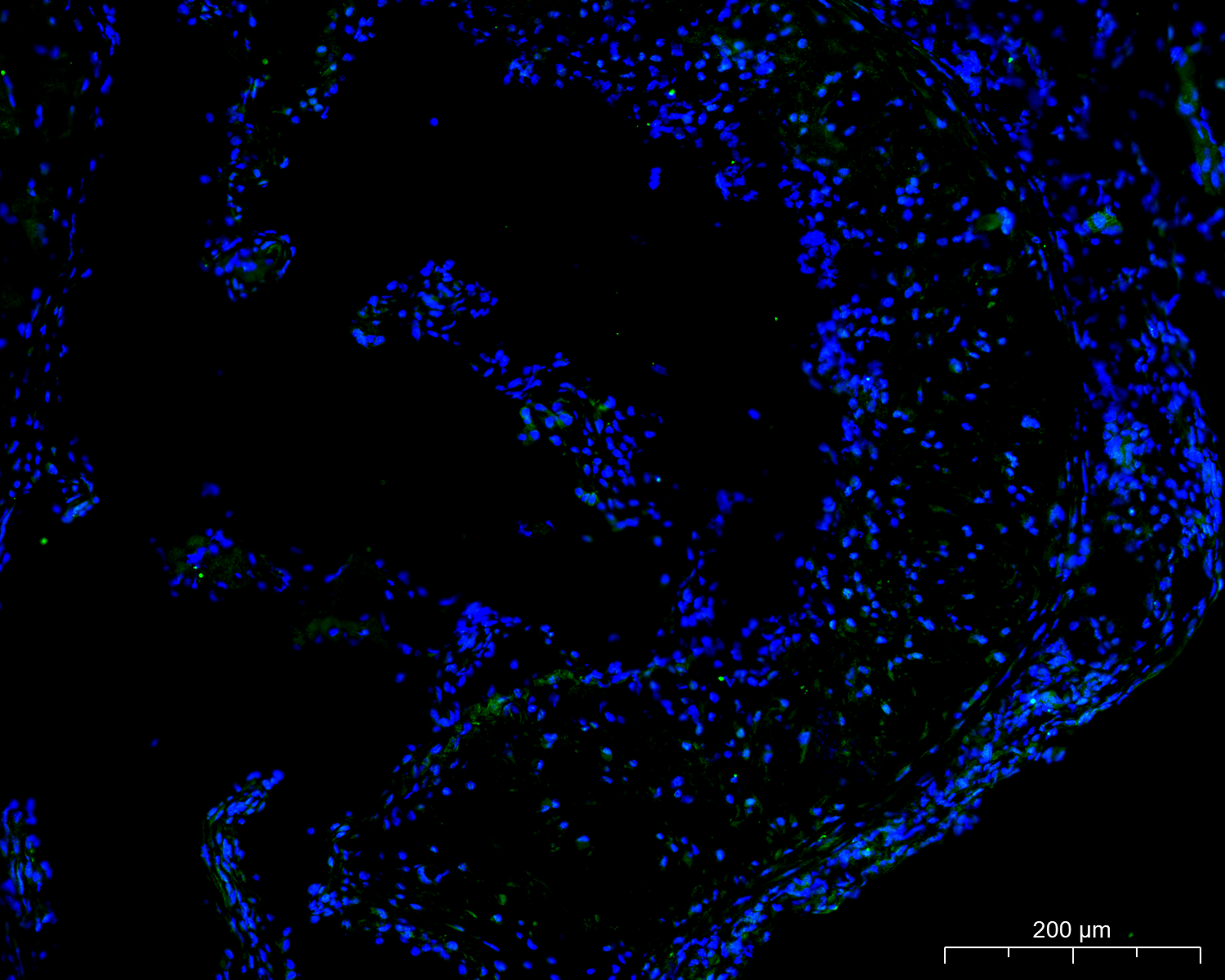

Supplement: S9 File — (ZIP) [file pone.0347758.s009.zip › 主动脉CD36/merge/PSB-M/85 CD36绿_20.0x.tif]

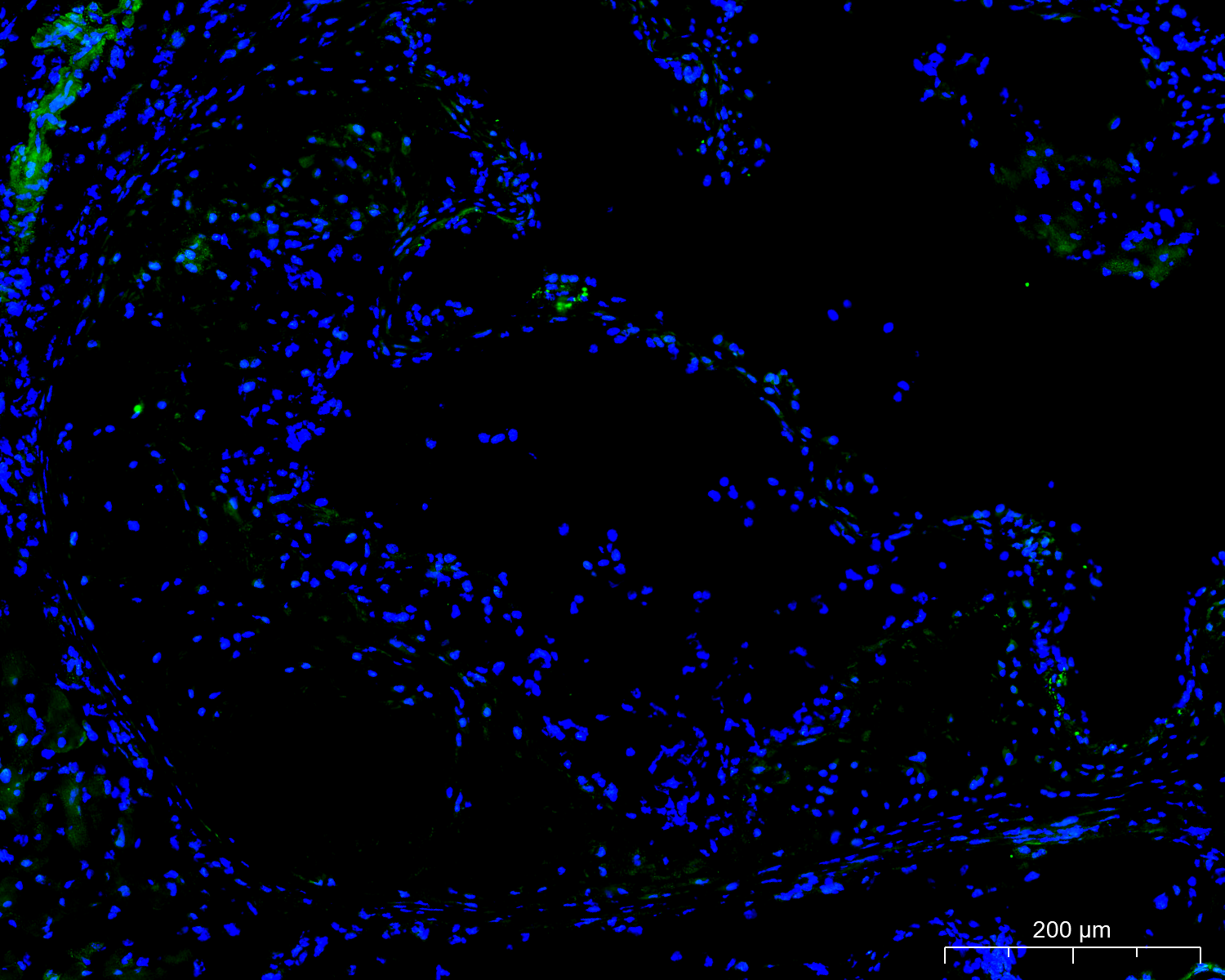

Supplement: S9 File — (ZIP) [file pone.0347758.s009.zip › 主动脉CD36/merge/PSB-M/89 CD36绿_20.0x.tif]

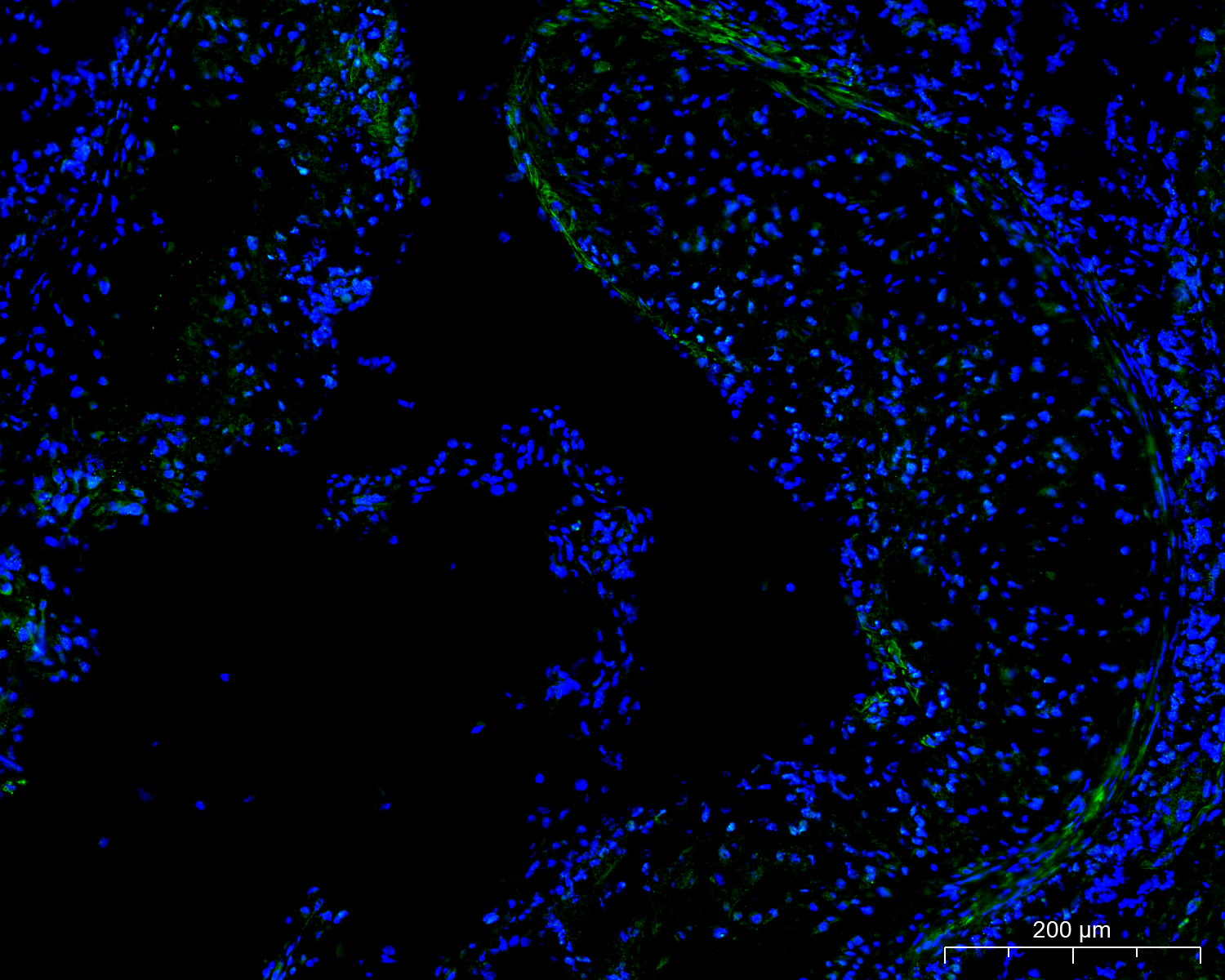

Supplement: S9 File — (ZIP) [file pone.0347758.s009.zip › 主动脉CD36/merge/PSB-M/90 CD36绿_20.0x.tif]

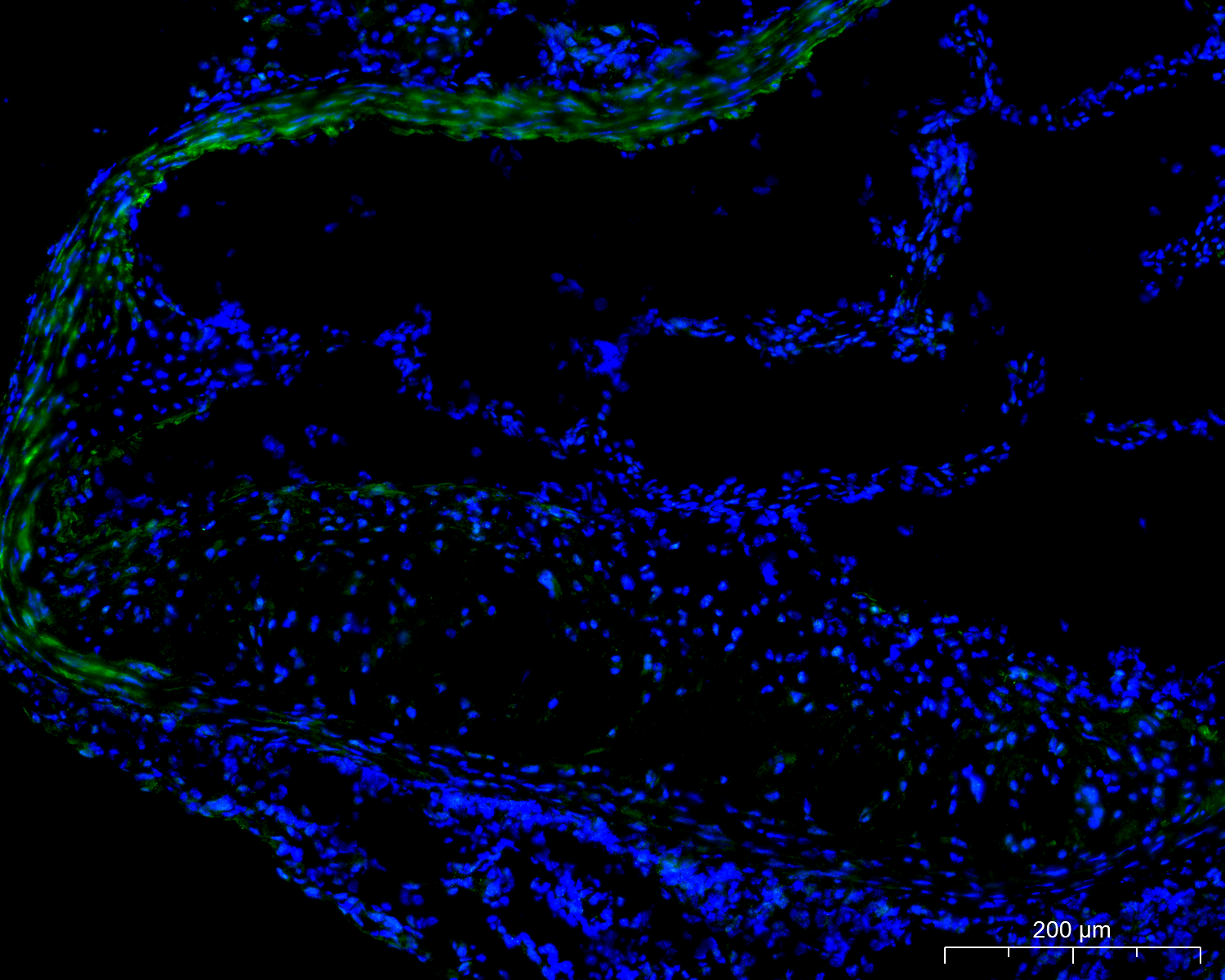

Supplement: S9 File — (ZIP) [file pone.0347758.s009.zip › 主动脉CD36/merge/PSB-M/92 CD36绿_20.0x.tif]

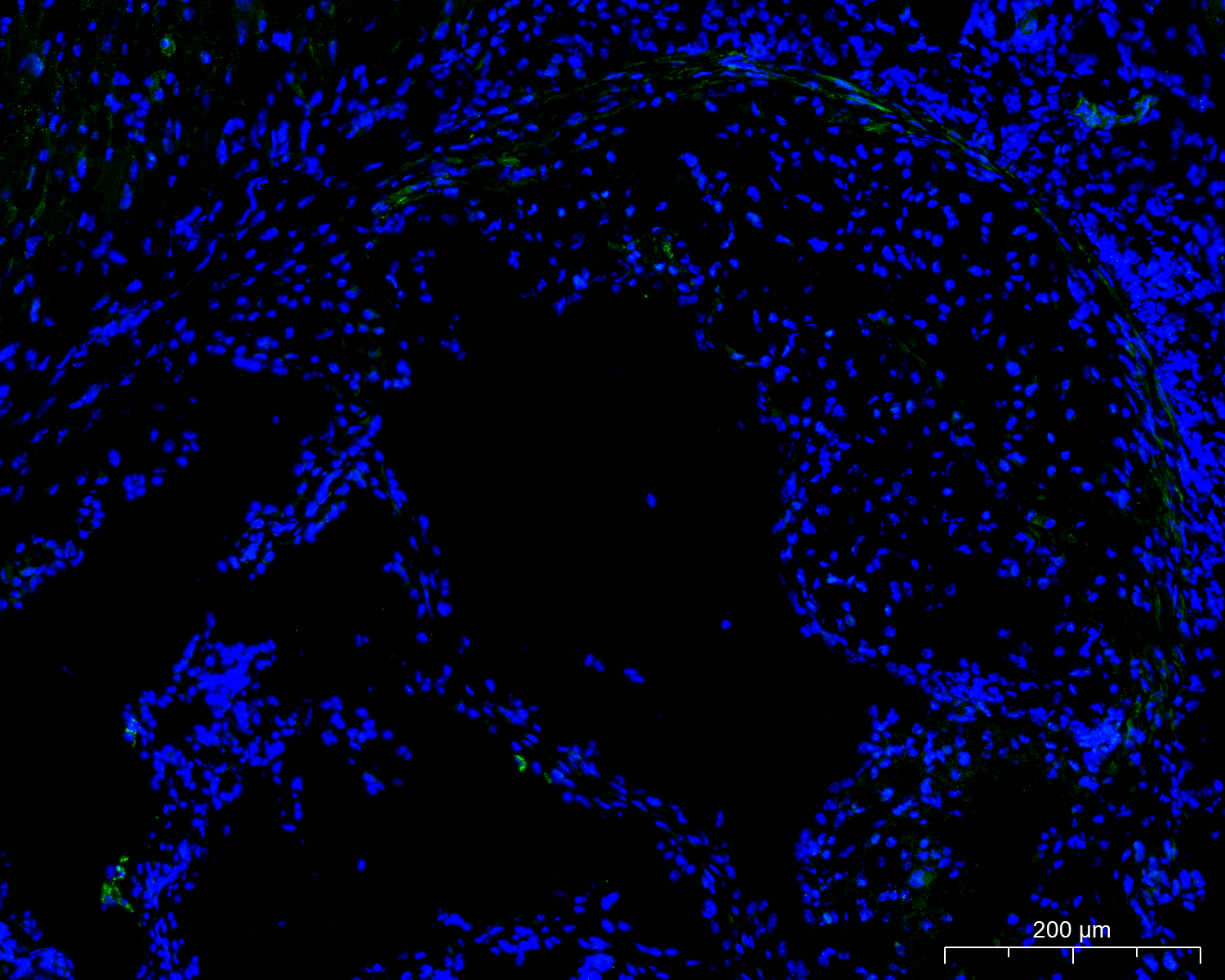

Supplement: S9 File — (ZIP) [file pone.0347758.s009.zip › 主动脉CD36/merge/statin/37 CD36绿_20.0x.tif]

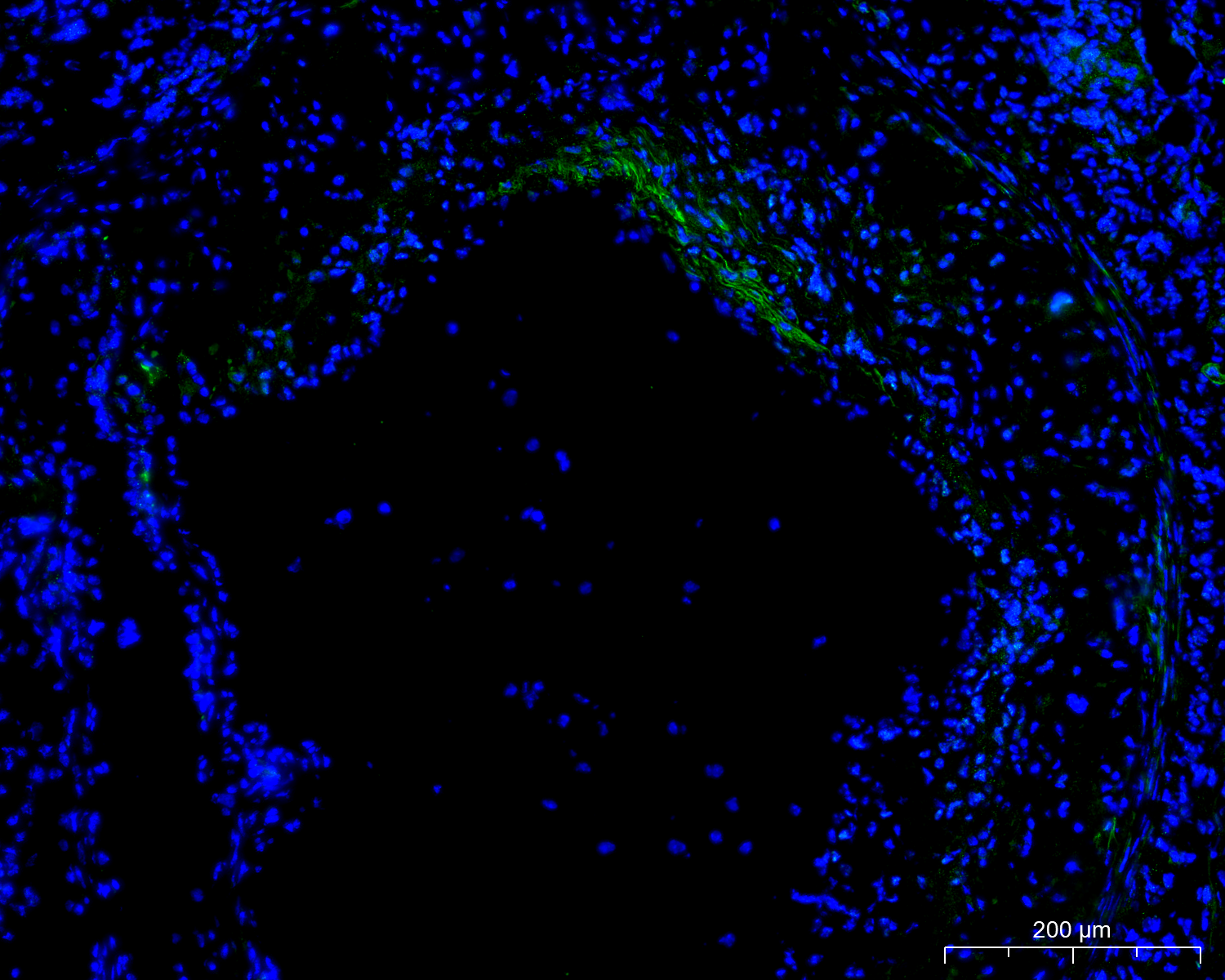

Supplement: S9 File — (ZIP) [file pone.0347758.s009.zip › 主动脉CD36/merge/statin/38 CD36绿_20.0x.tif]

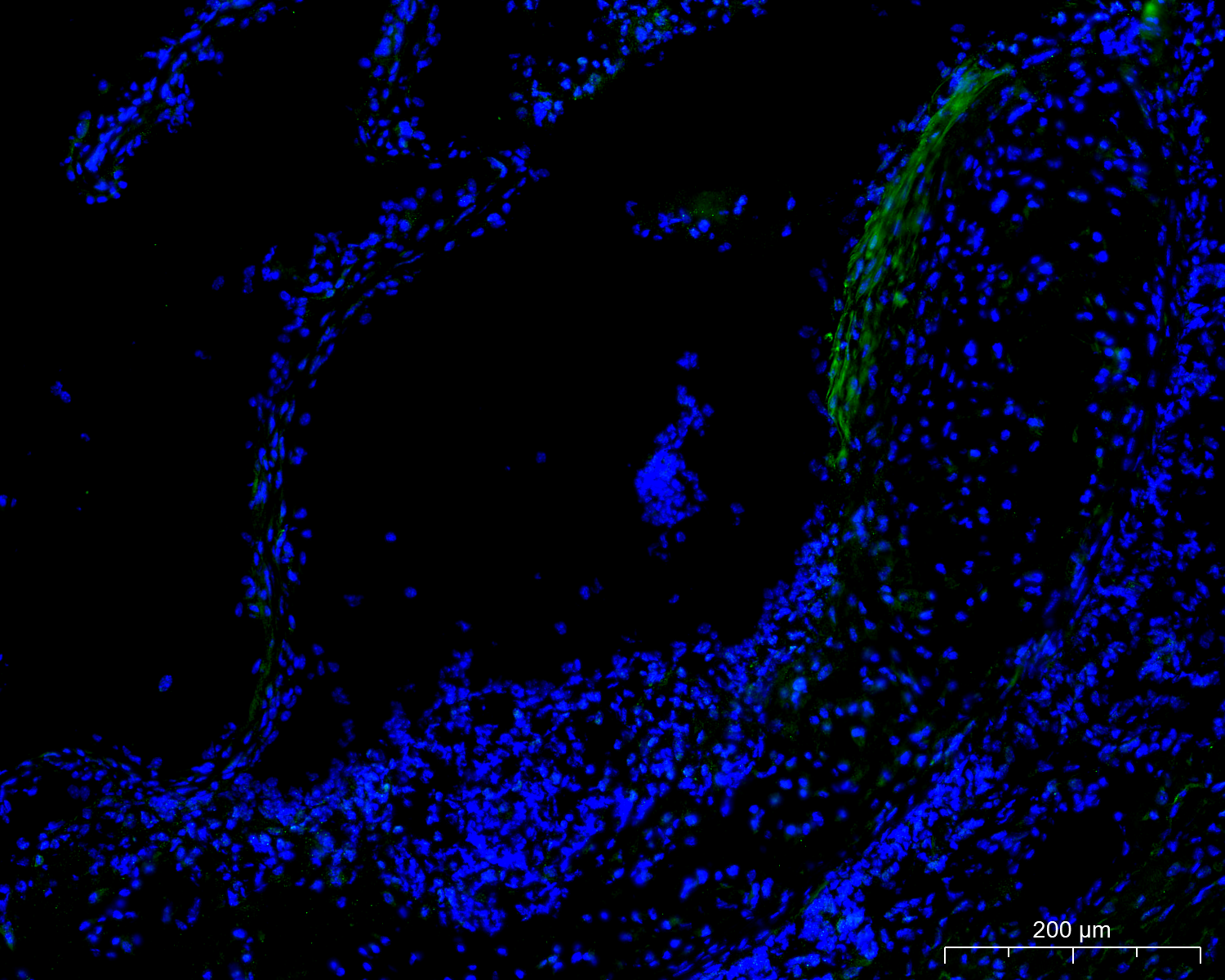

Supplement: S9 File — (ZIP) [file pone.0347758.s009.zip › 主动脉CD36/merge/statin/40 CD36绿_20.0x.tif]

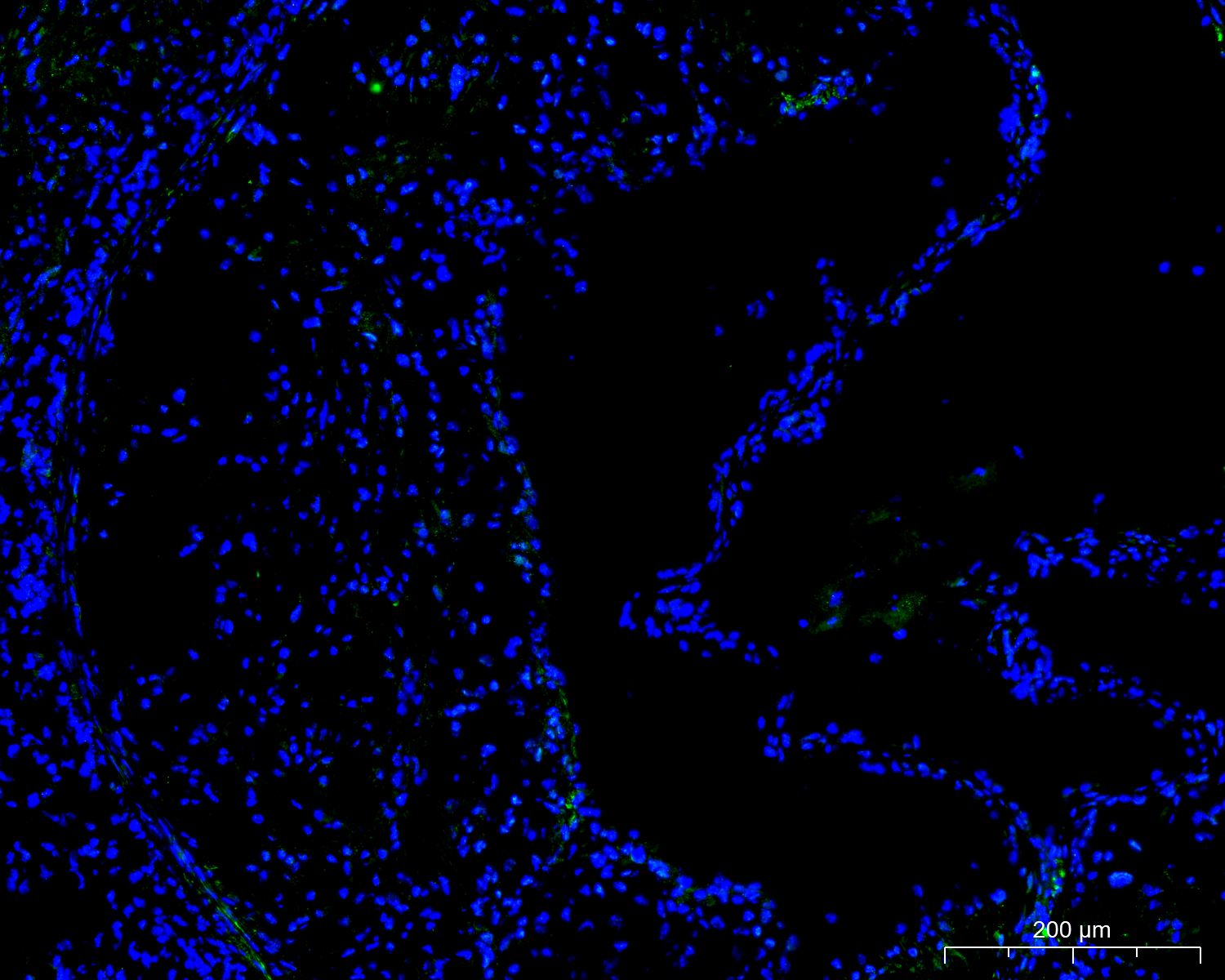

Supplement: S9 File — (ZIP) [file pone.0347758.s009.zip › 主动脉CD36/merge/statin/41 CD36绿_20.0x.tif]

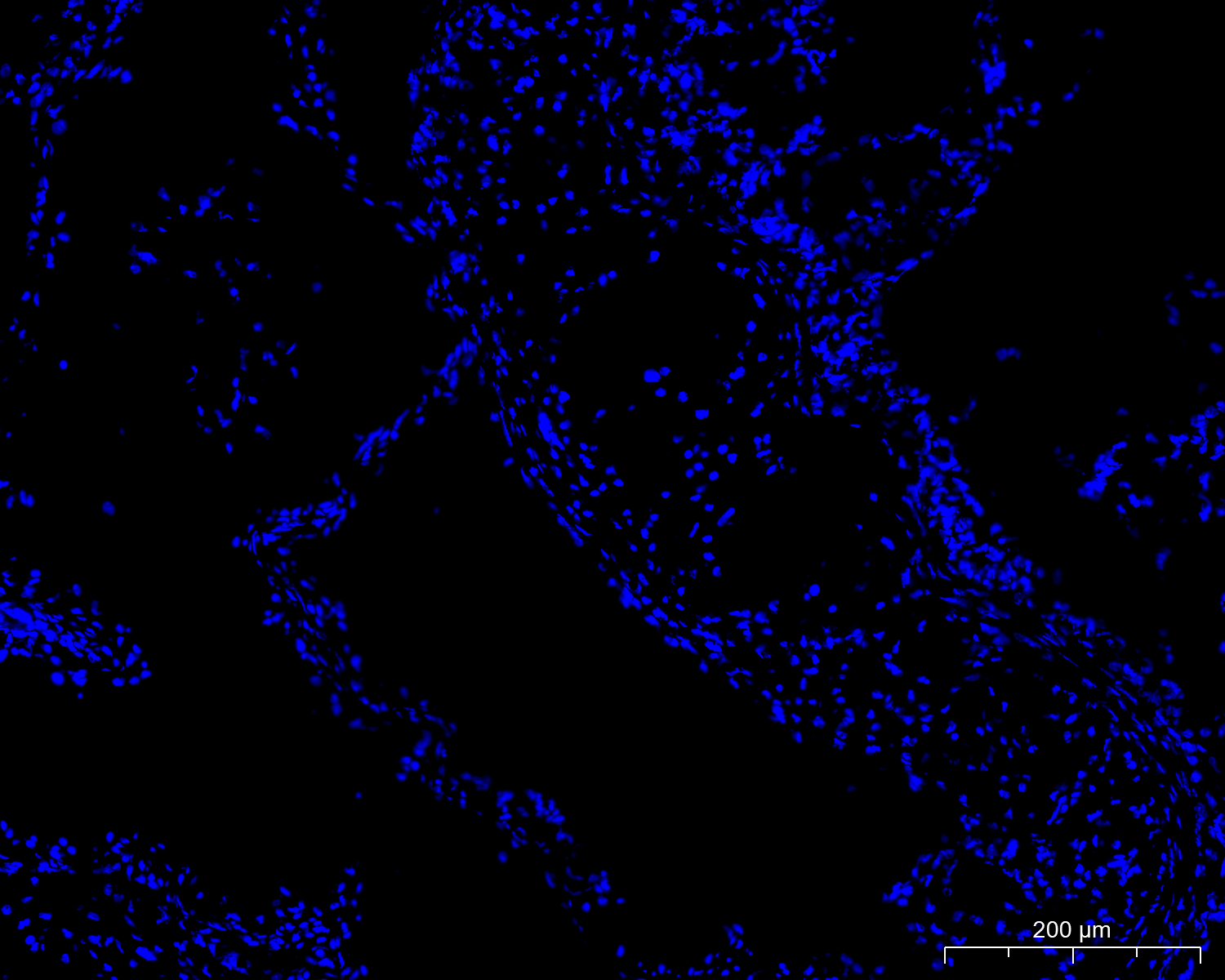

Supplement: S10 File — (ZIP) [file pone.0347758.s010.zip › 主动脉ROS/DAPI/AS/23 ROS红_20.0x.tif]

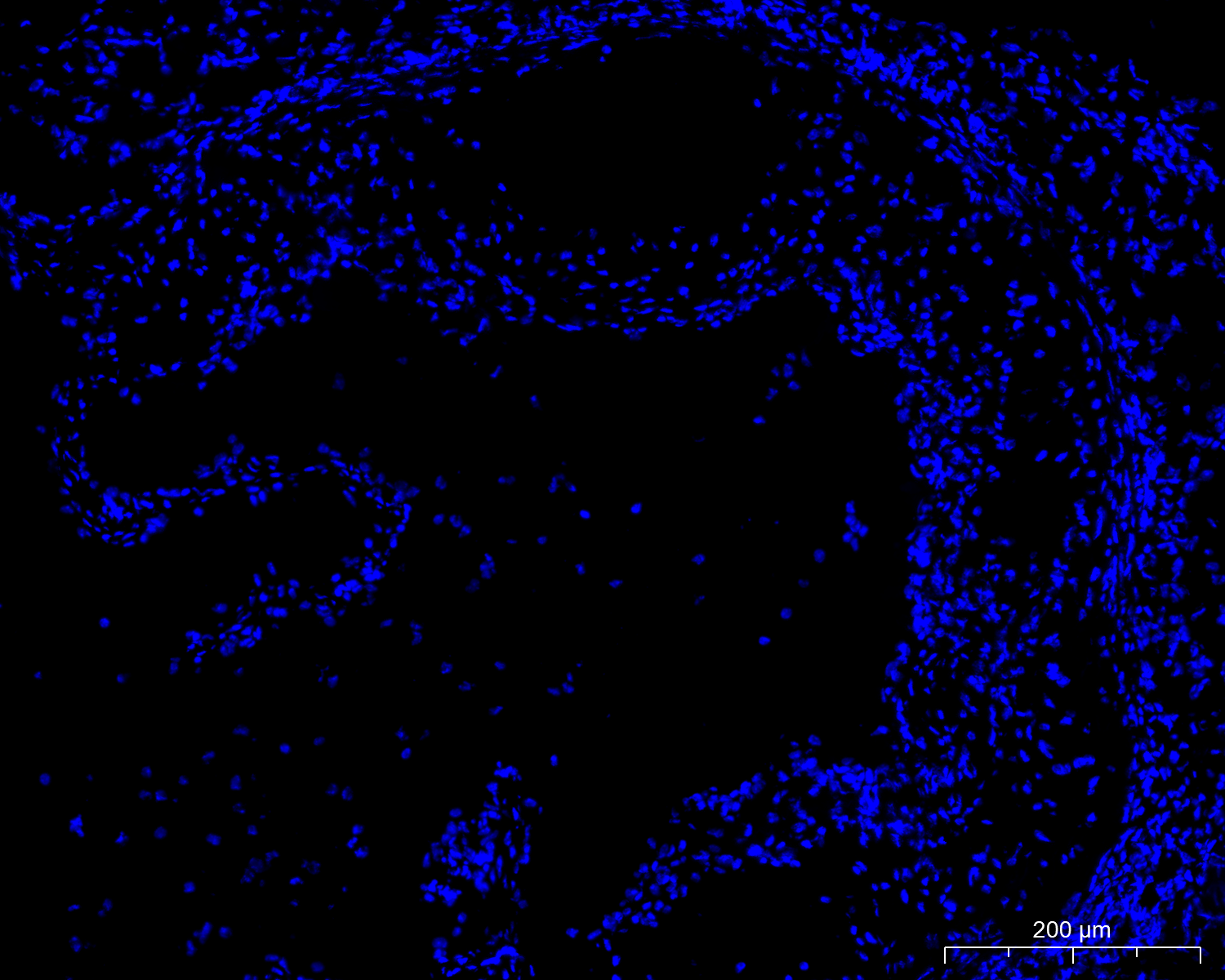

Supplement: S10 File — (ZIP) [file pone.0347758.s010.zip › 主动脉ROS/DAPI/AS/27 ROS红_20.0x.tif]

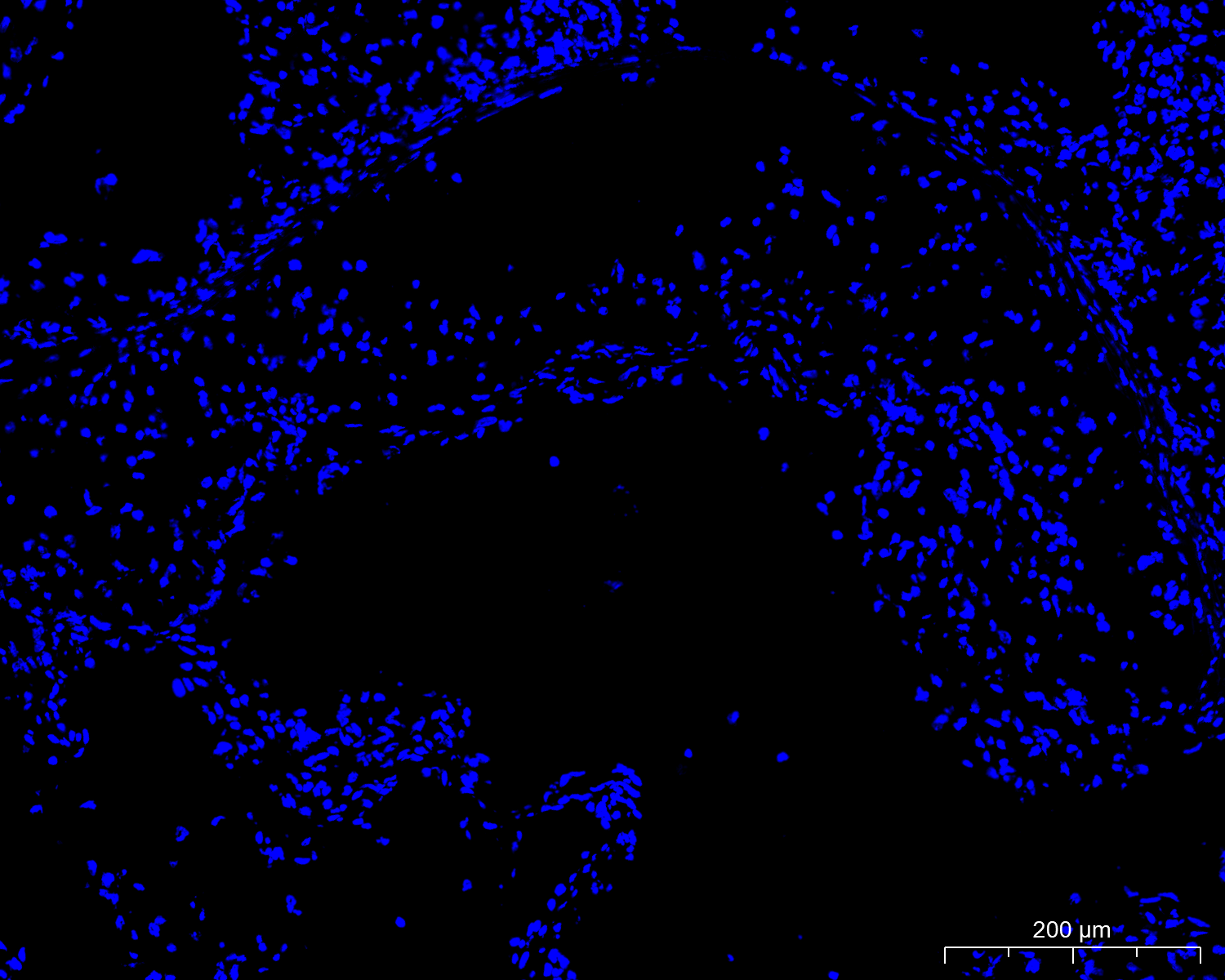

Supplement: S10 File — (ZIP) [file pone.0347758.s010.zip › 主动脉ROS/DAPI/AS/28 ROS红_20.0x.tif]

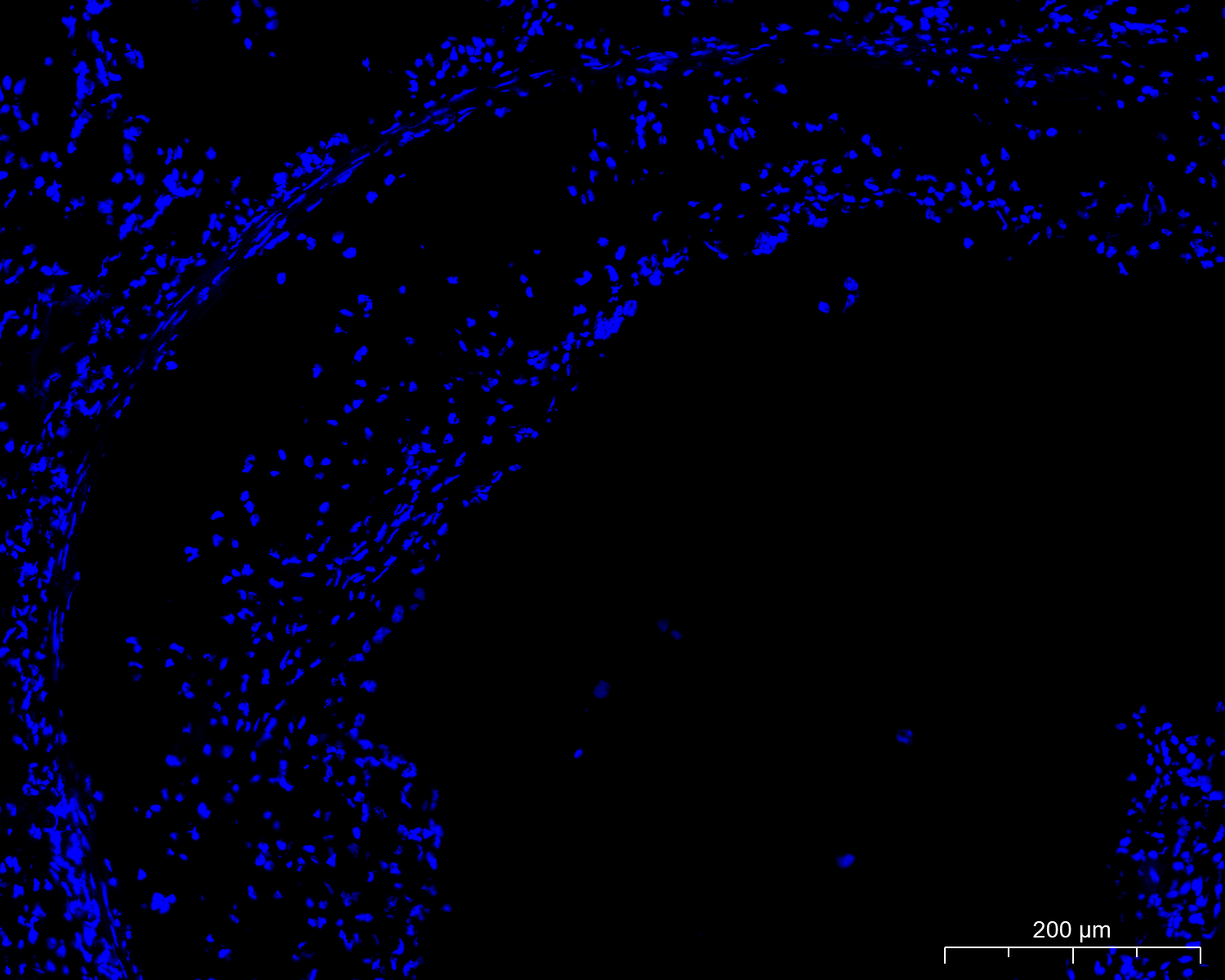

Supplement: S10 File — (ZIP) [file pone.0347758.s010.zip › 主动脉ROS/DAPI/AS/31 ROS红_20.0x.tif]

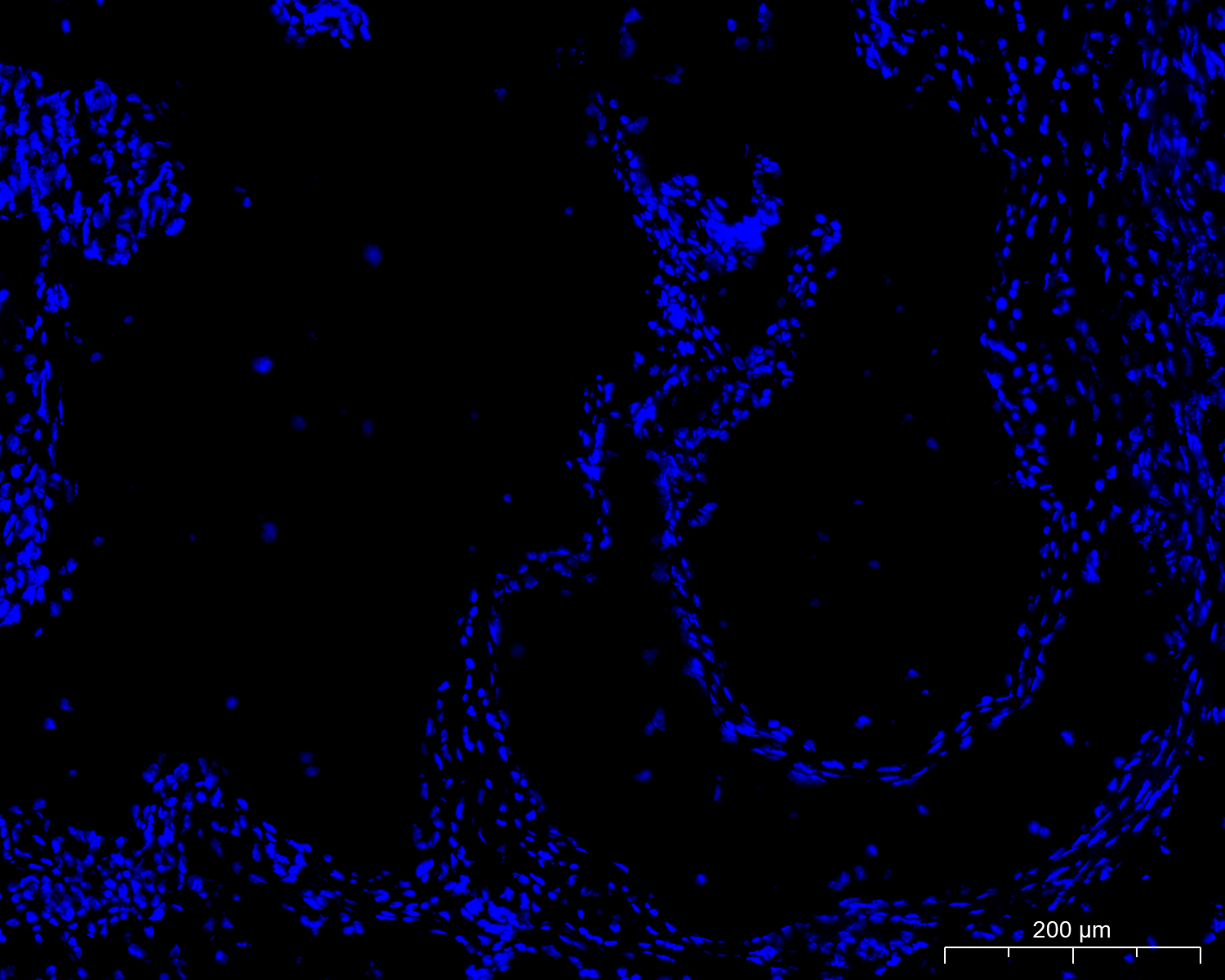

Supplement: S10 File — (ZIP) [file pone.0347758.s010.zip › 主动脉ROS/DAPI/control/1 ROS红_20.0x.tif]

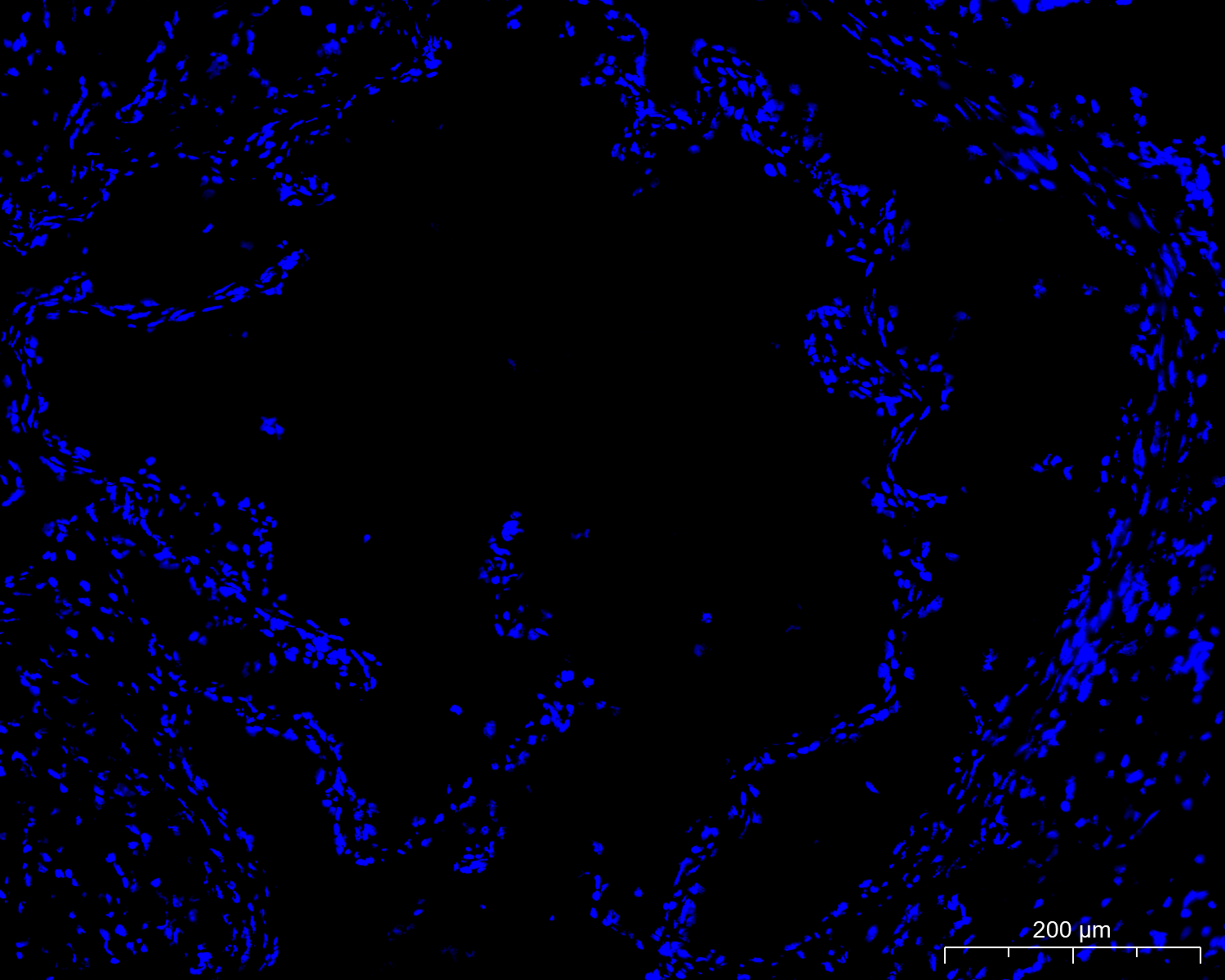

Supplement: S10 File — (ZIP) [file pone.0347758.s010.zip › 主动脉ROS/DAPI/control/2 ROS红_20.0x.tif]

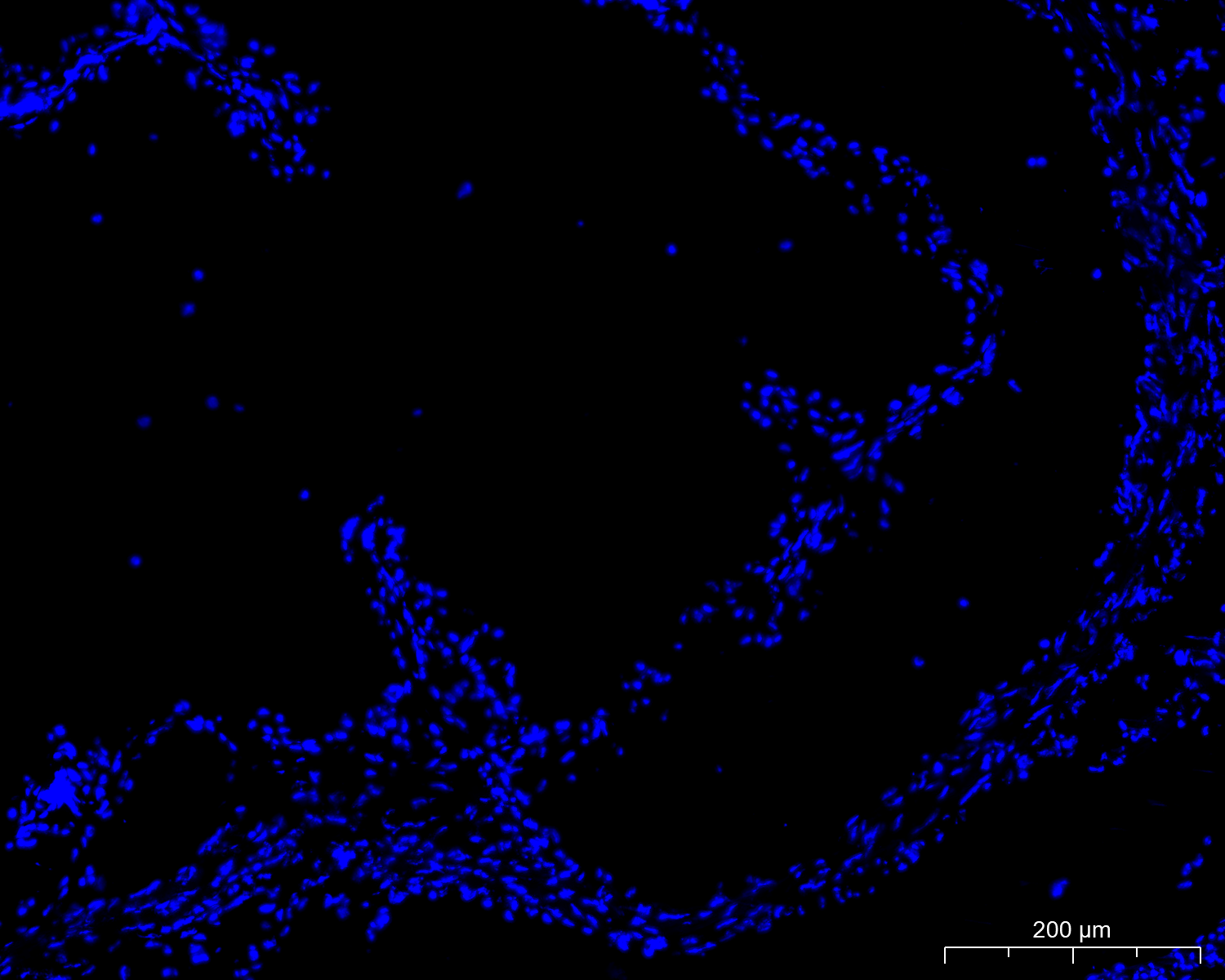

Supplement: S10 File — (ZIP) [file pone.0347758.s010.zip › 主动脉ROS/DAPI/control/6 ROS红_20.0x.tif]

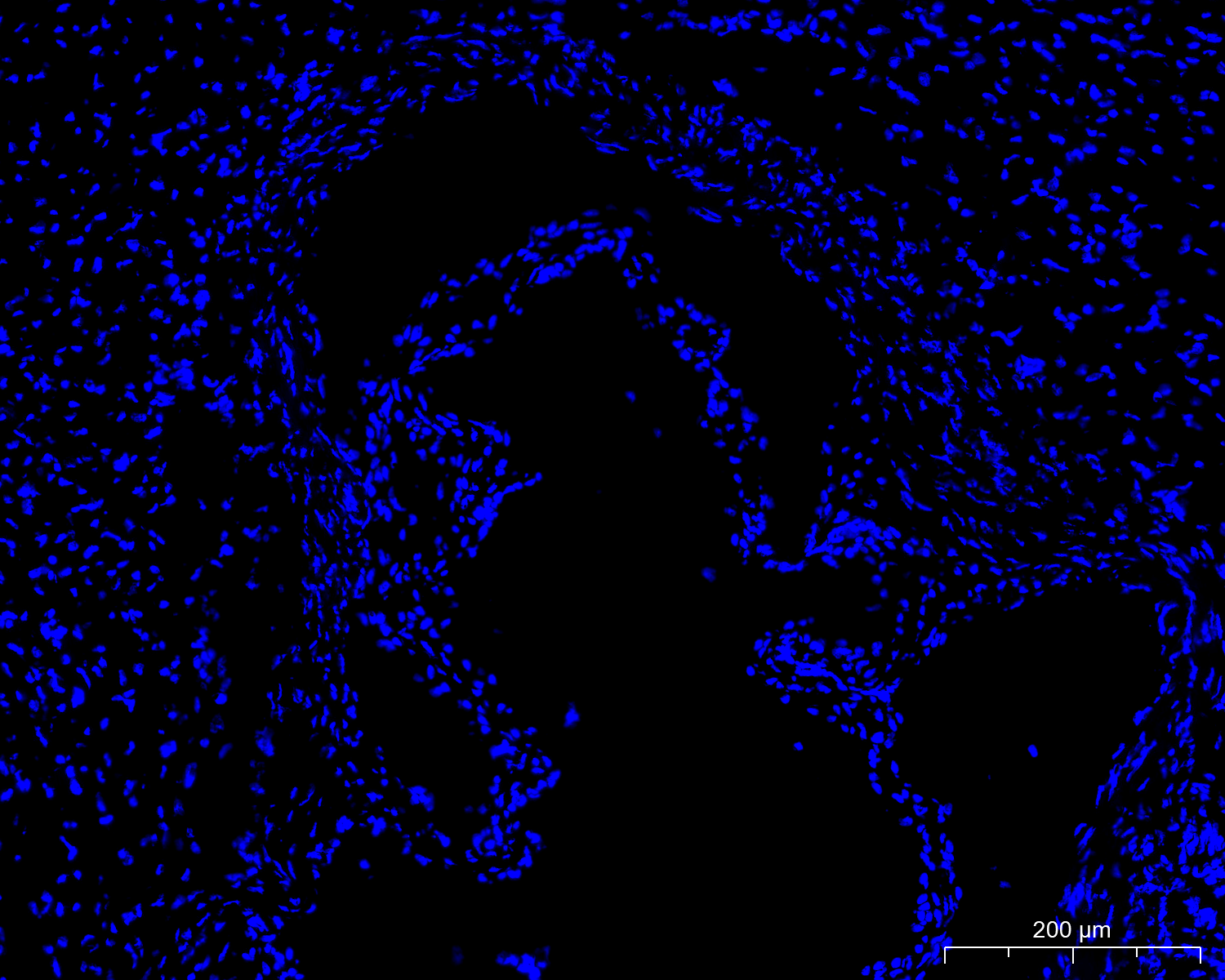

Supplement: S10 File — (ZIP) [file pone.0347758.s010.zip › 主动脉ROS/DAPI/control/7 ROS红_20.0x.tif]

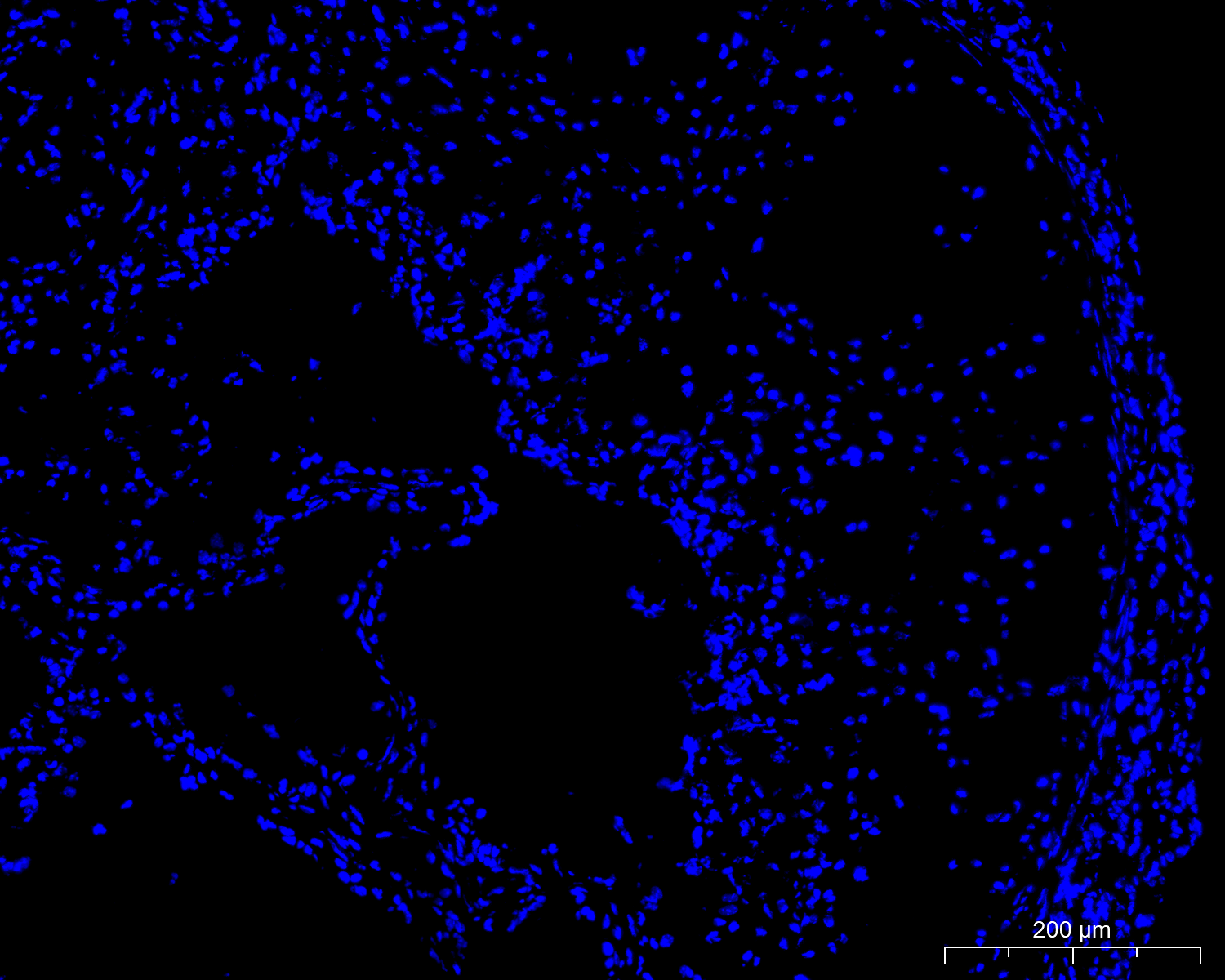

Supplement: S10 File — (ZIP) [file pone.0347758.s010.zip › 主动脉ROS/DAPI/PSB-H/100 ROS红_20.0x.tif]

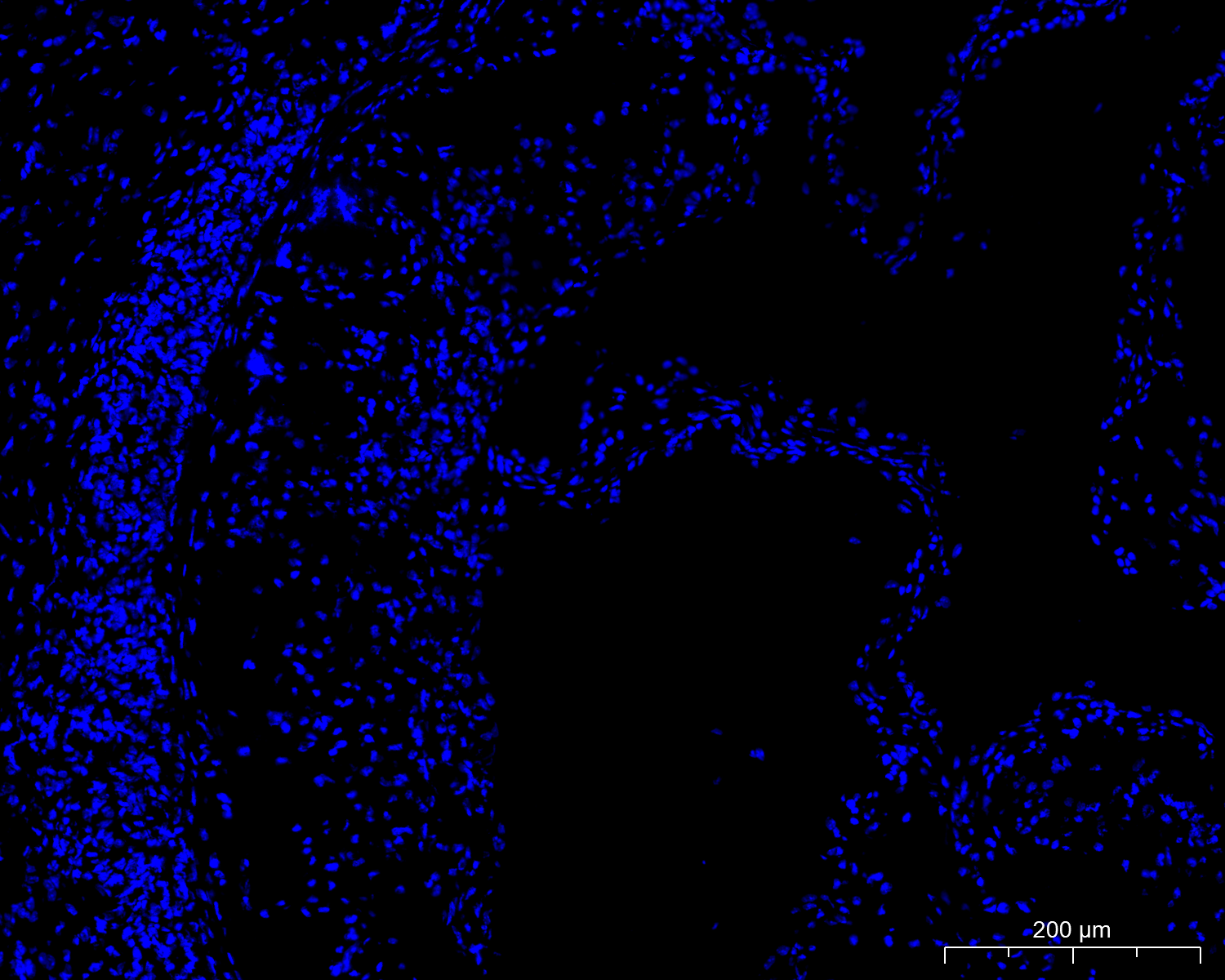

Supplement: S10 File — (ZIP) [file pone.0347758.s010.zip › 主动脉ROS/DAPI/PSB-H/93 ROS红_20.0x.tif]

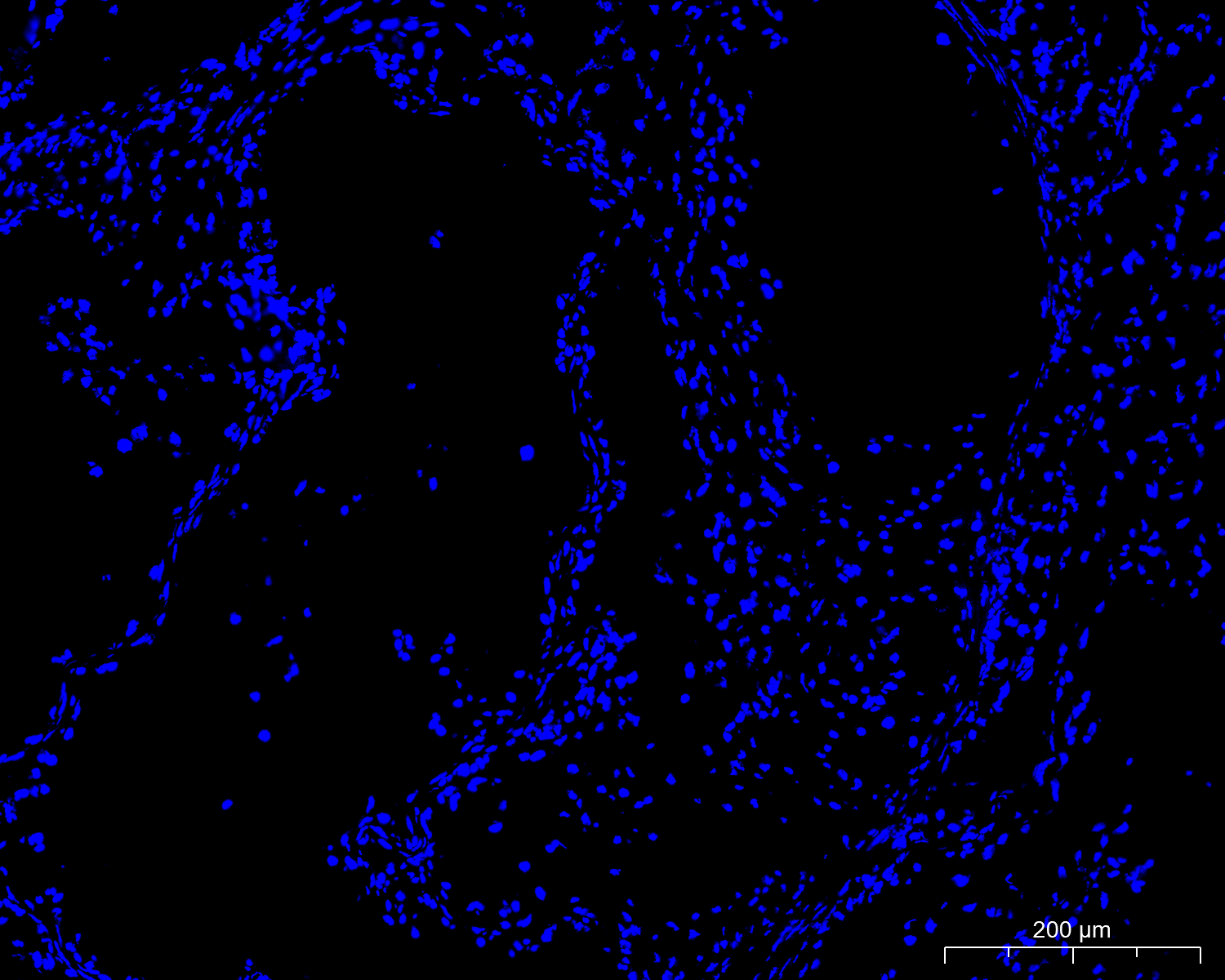

Supplement: S10 File — (ZIP) [file pone.0347758.s010.zip › 主动脉ROS/DAPI/PSB-H/98 ROS红_20.0x.tif]

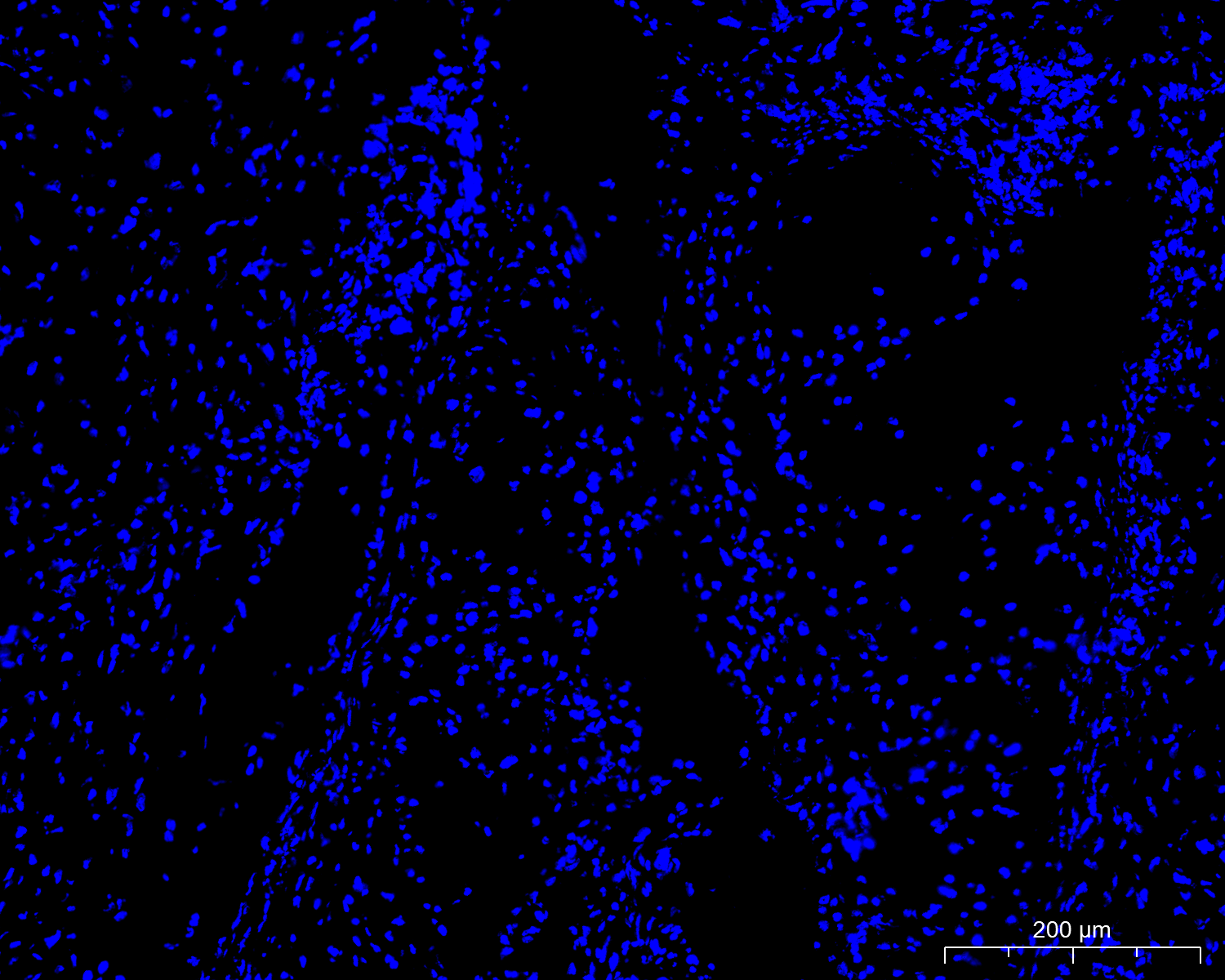

Supplement: S10 File — (ZIP) [file pone.0347758.s010.zip › 主动脉ROS/DAPI/PSB-H/A1 ROS红_20.0x.tif]

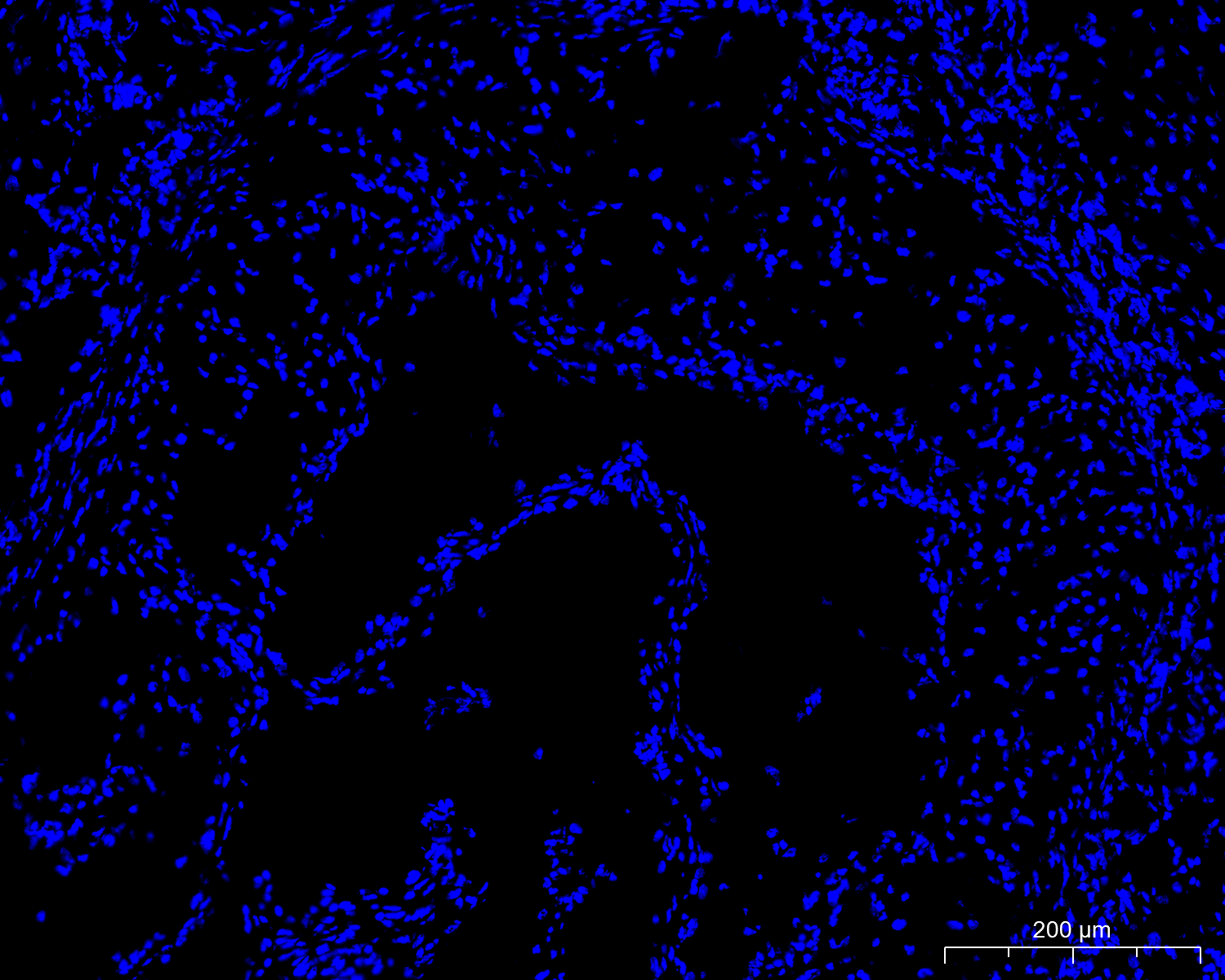

Supplement: S10 File — (ZIP) [file pone.0347758.s010.zip › 主动脉ROS/DAPI/PSB-L/74 ROS红_20.0x.tif]

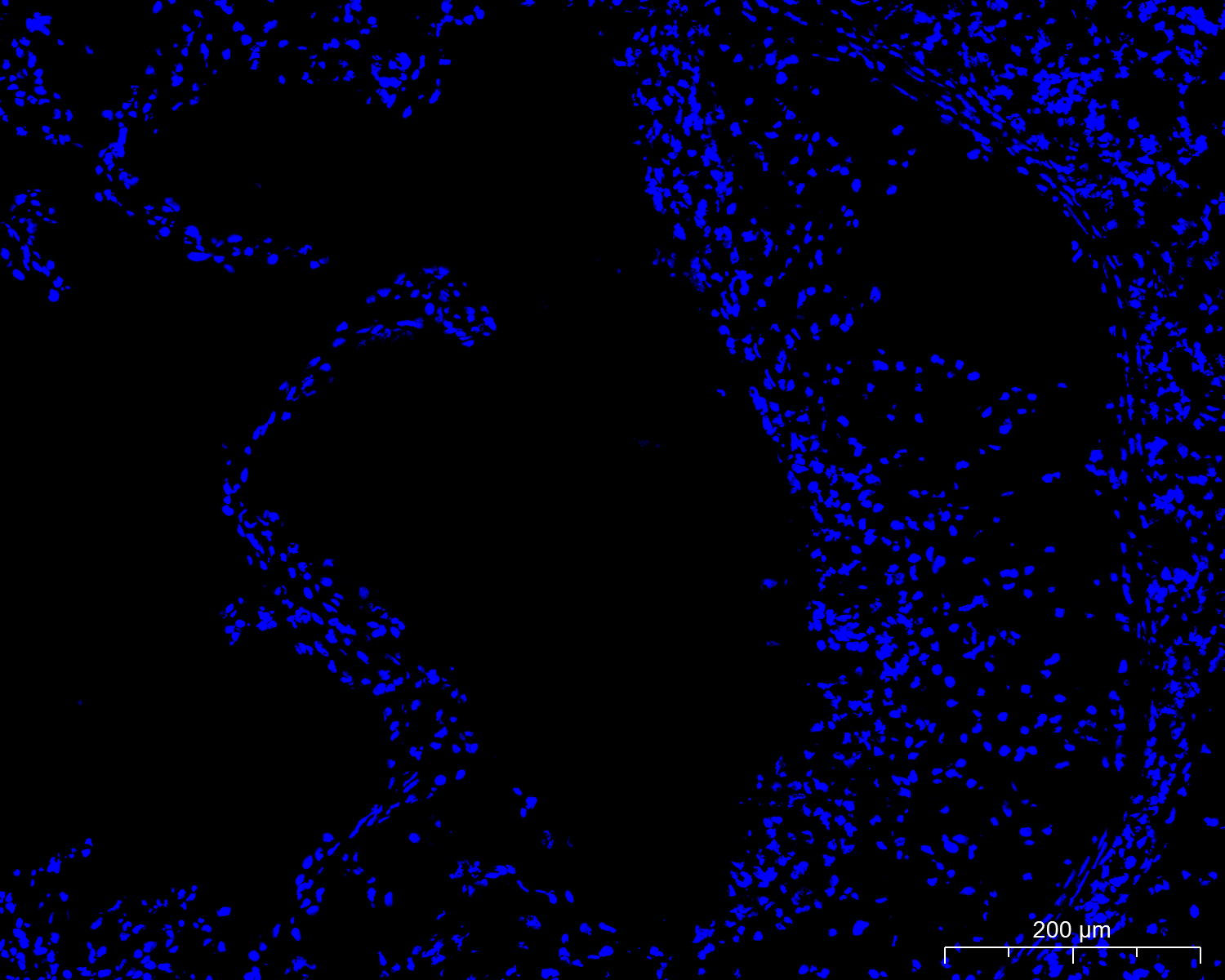

Supplement: S10 File — (ZIP) [file pone.0347758.s010.zip › 主动脉ROS/DAPI/PSB-L/77 ROS红_20.0x.tif]

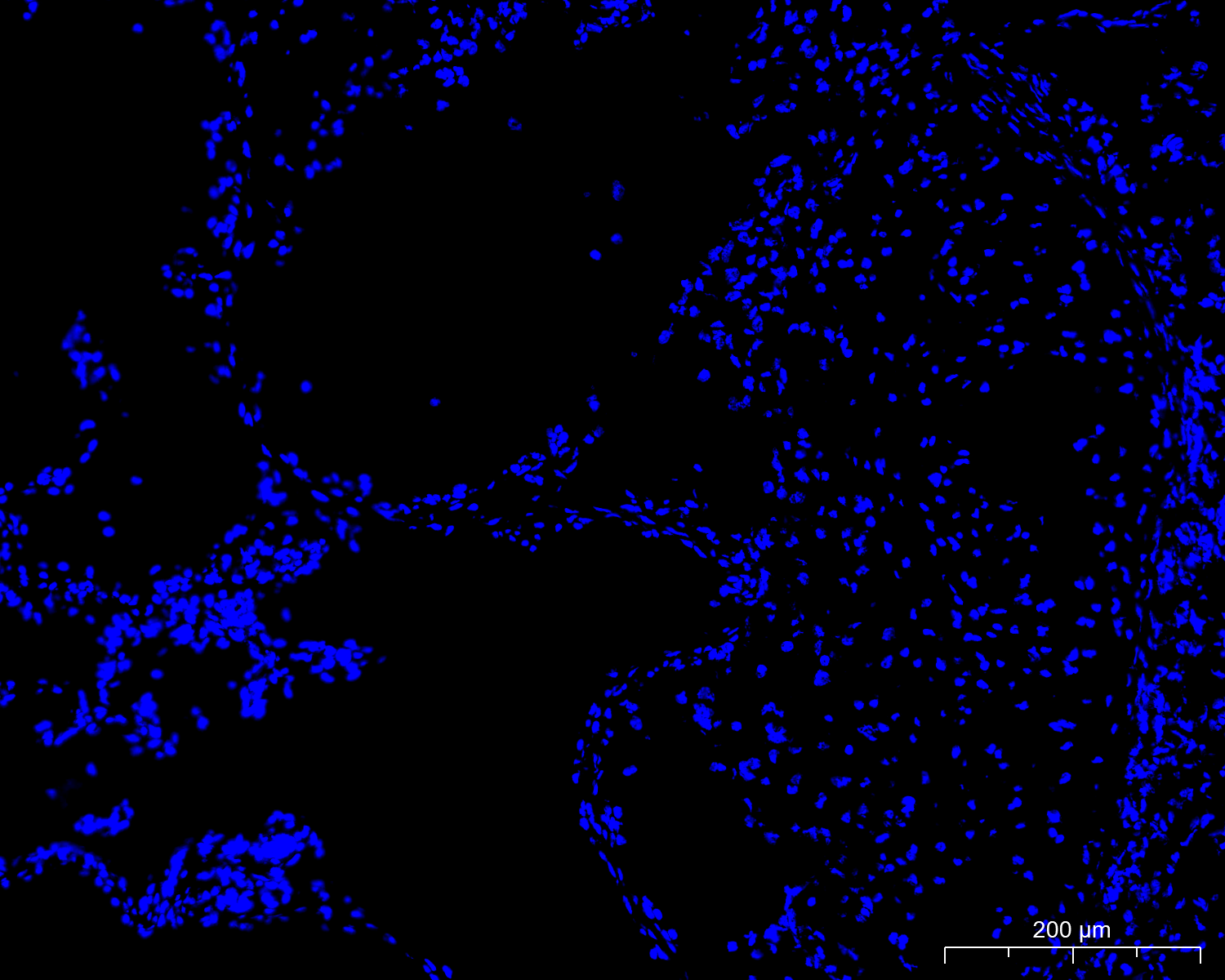

Supplement: S10 File — (ZIP) [file pone.0347758.s010.zip › 主动脉ROS/DAPI/PSB-L/80 ROS红_20.0x.tif]

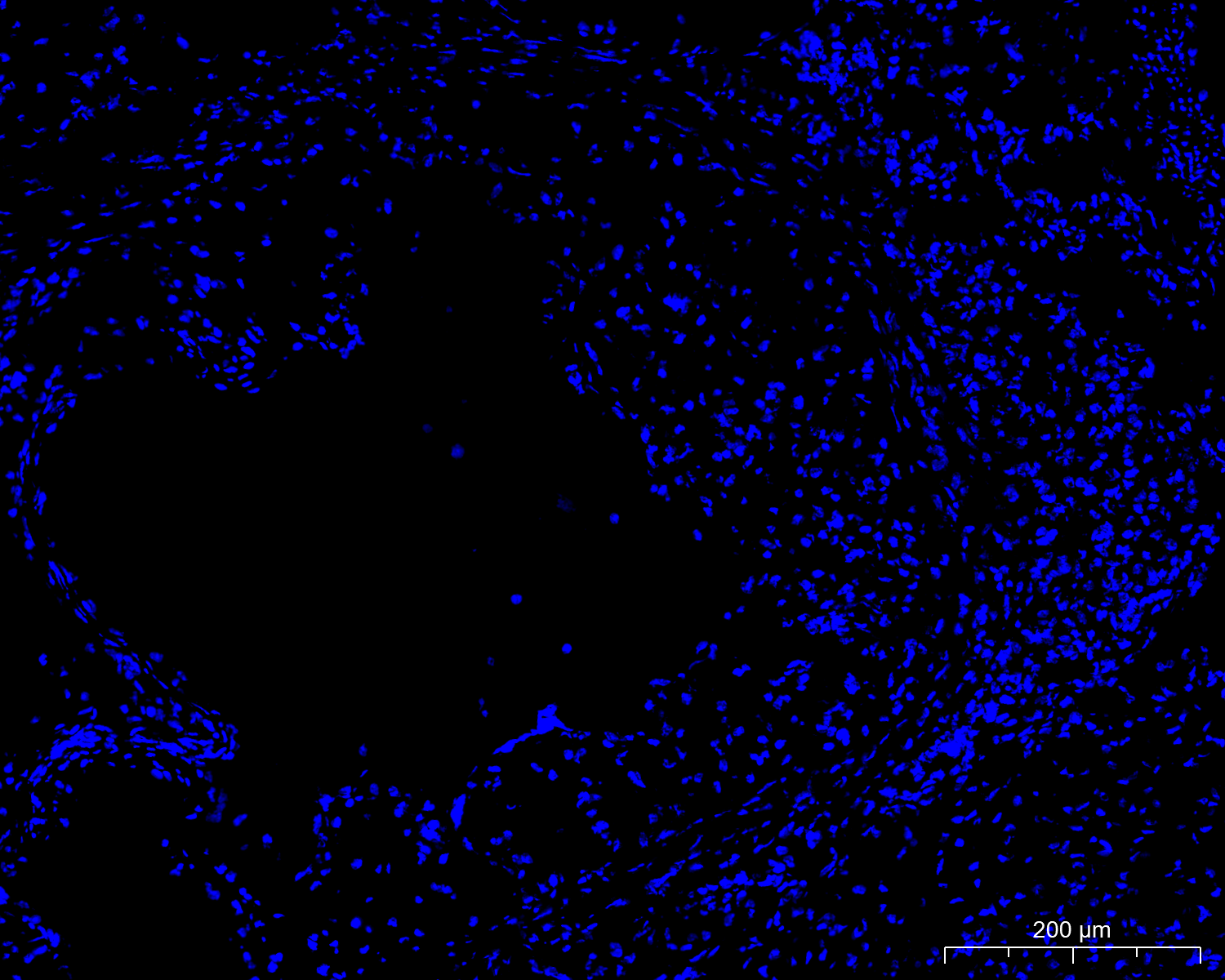

Supplement: S10 File — (ZIP) [file pone.0347758.s010.zip › 主动脉ROS/DAPI/PSB-L/82 ROS红_20.0x.tif]

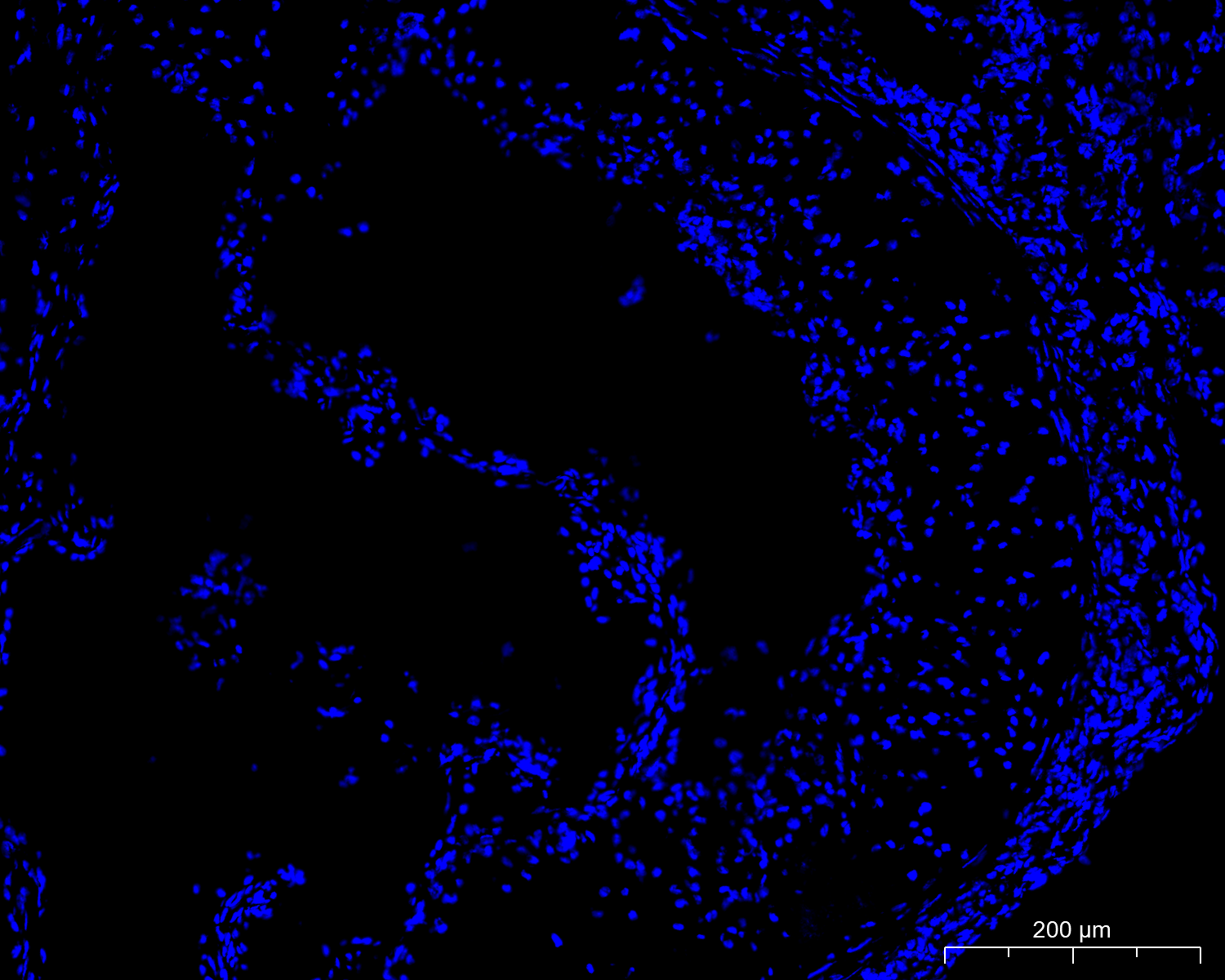

Supplement: S10 File — (ZIP) [file pone.0347758.s010.zip › 主动脉ROS/DAPI/PSB-M/85 ROS红_20.0x.tif]

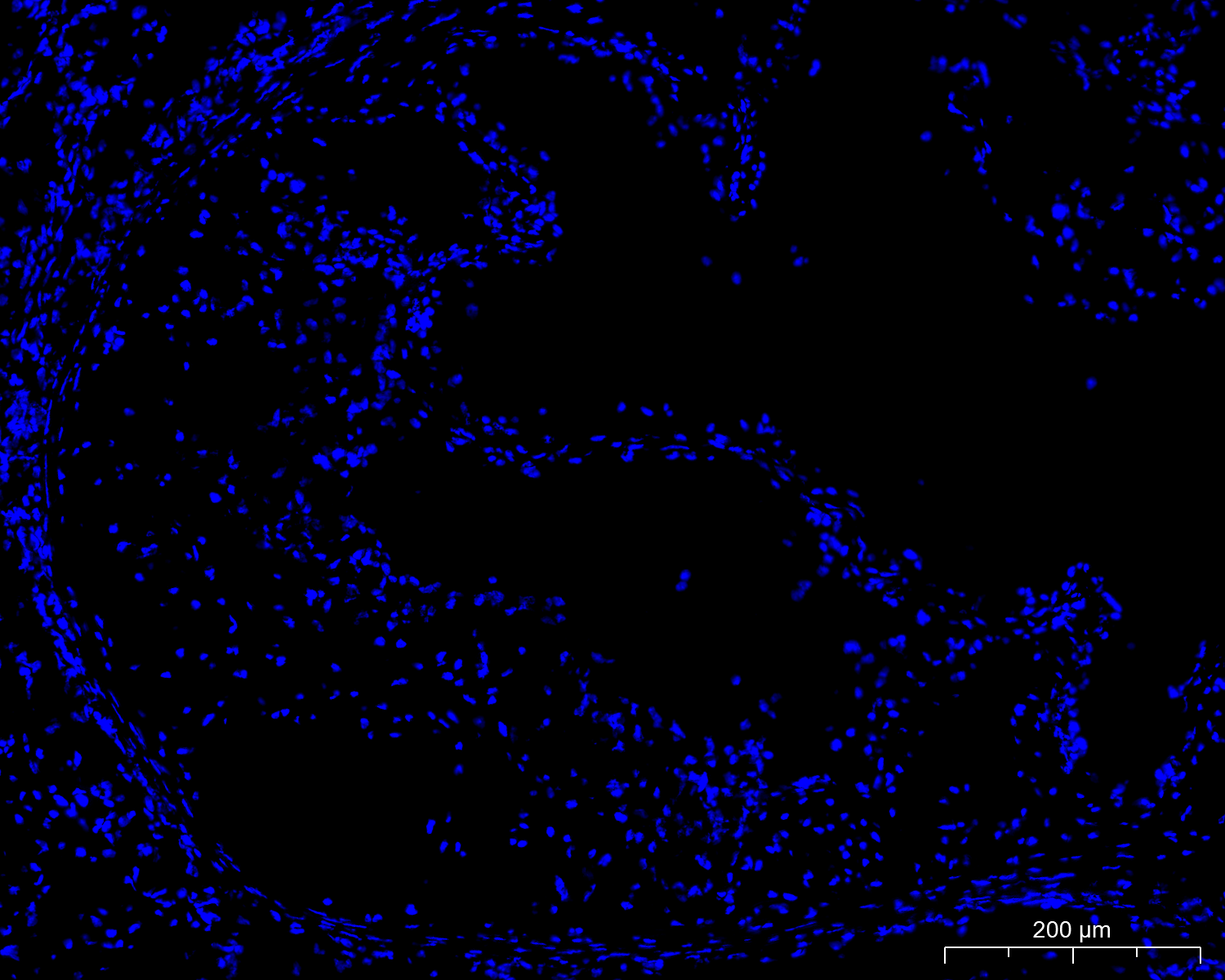

Supplement: S10 File — (ZIP) [file pone.0347758.s010.zip › 主动脉ROS/DAPI/PSB-M/89 ROS红_20.0x.tif]

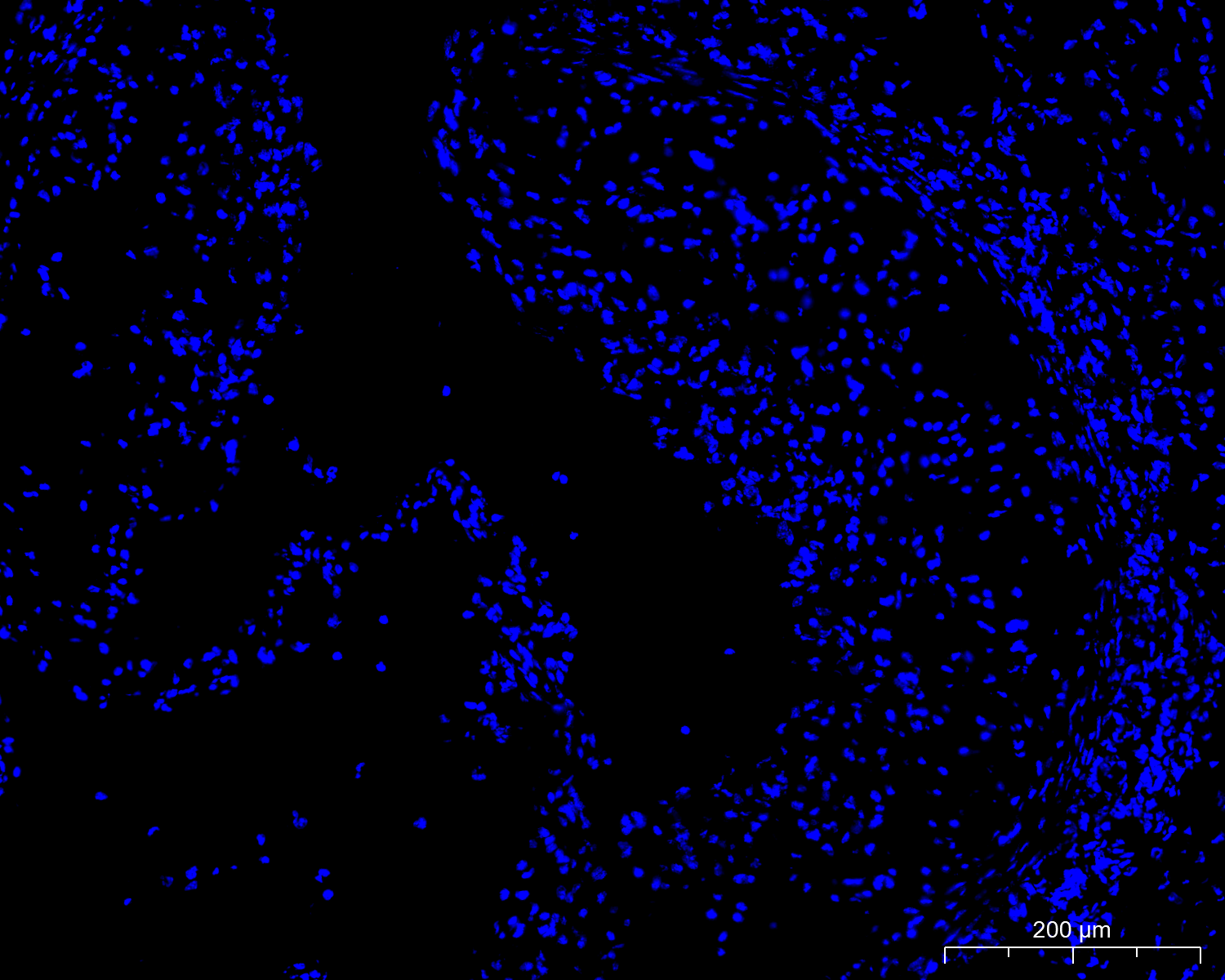

Supplement: S10 File — (ZIP) [file pone.0347758.s010.zip › 主动脉ROS/DAPI/PSB-M/90 ROS红_20.0x.tif]

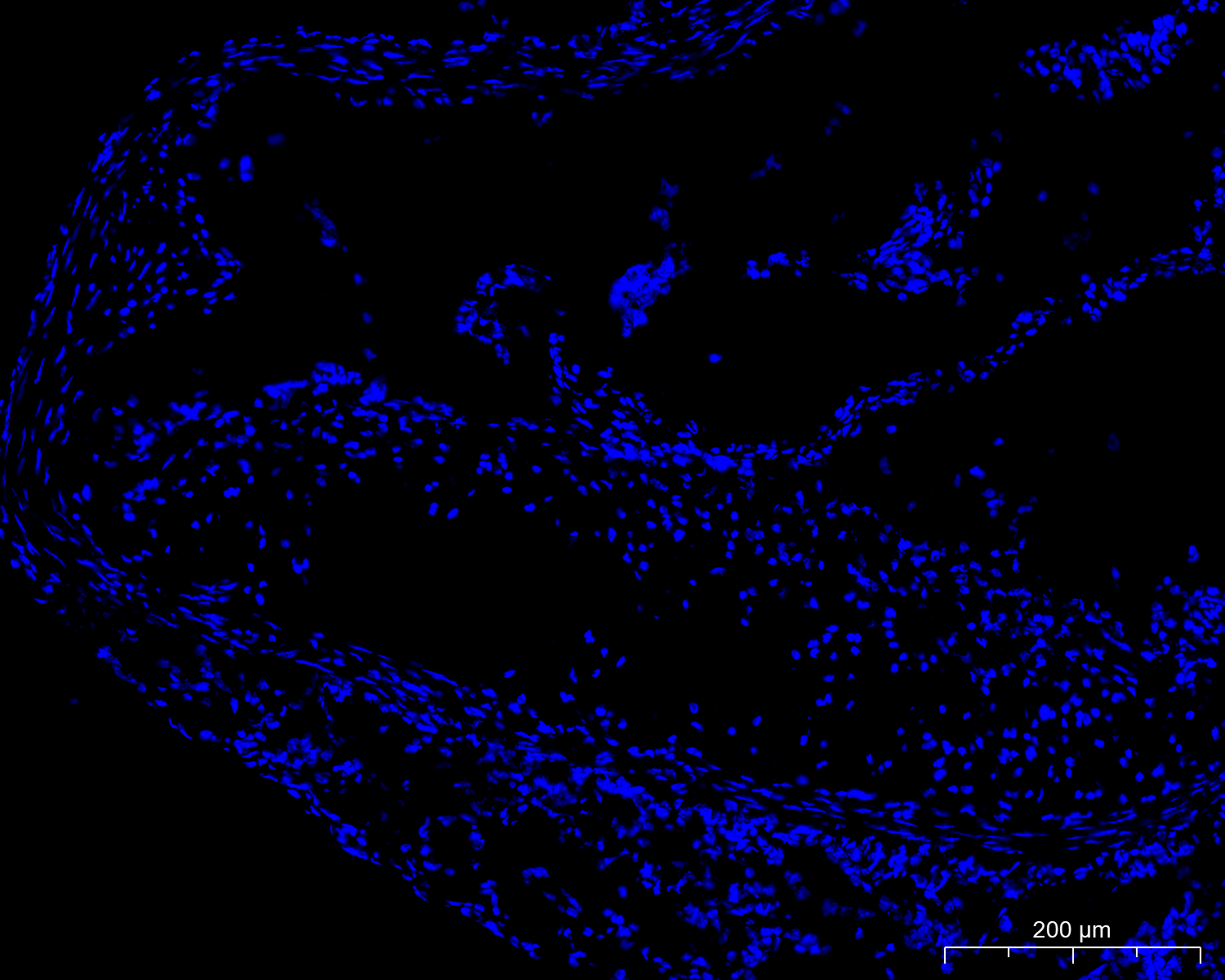

Supplement: S10 File — (ZIP) [file pone.0347758.s010.zip › 主动脉ROS/DAPI/PSB-M/92 ROS红_20.0x.tif]

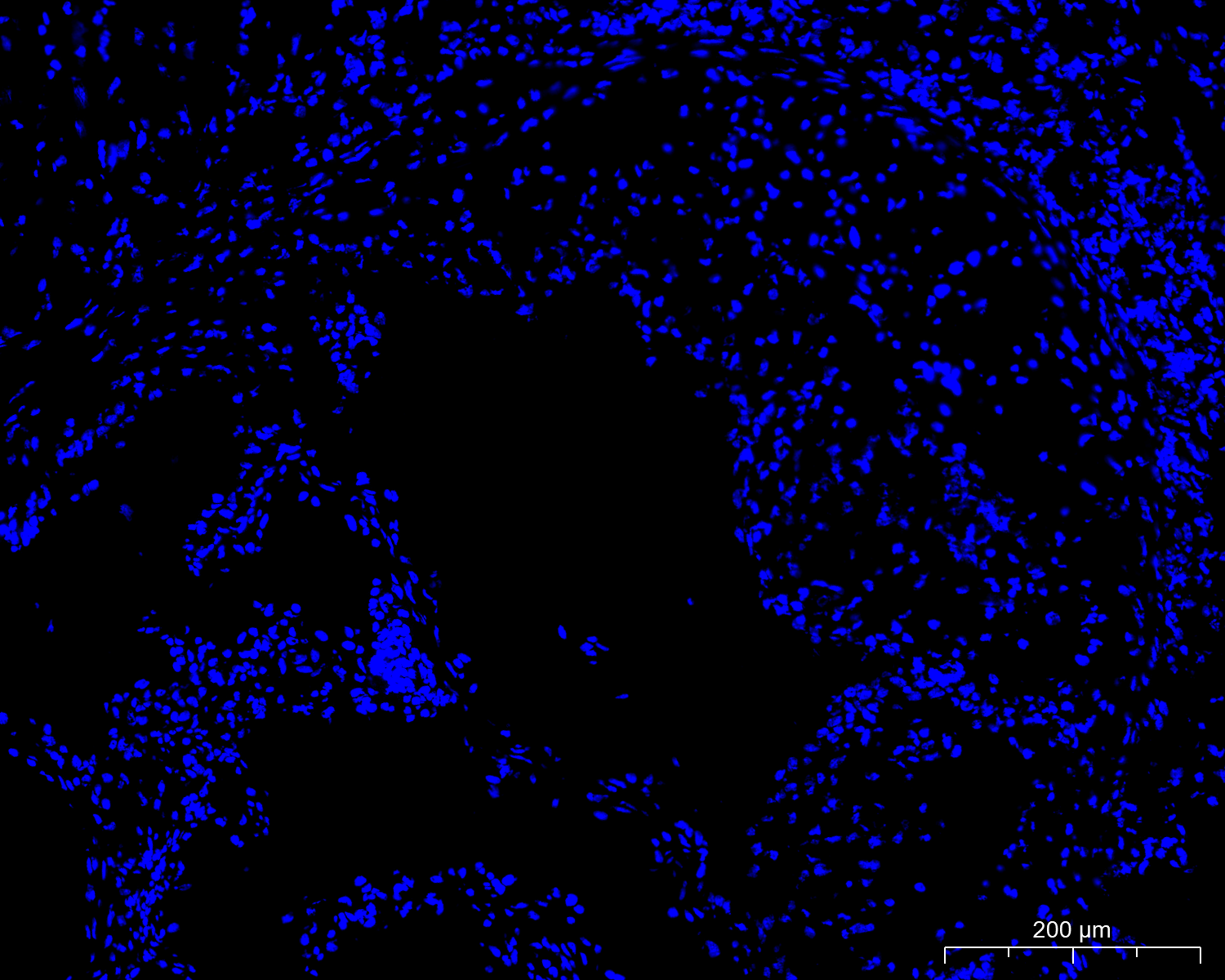

Supplement: S10 File — (ZIP) [file pone.0347758.s010.zip › 主动脉ROS/DAPI/statin/37 ROS红_20.0x.tif]

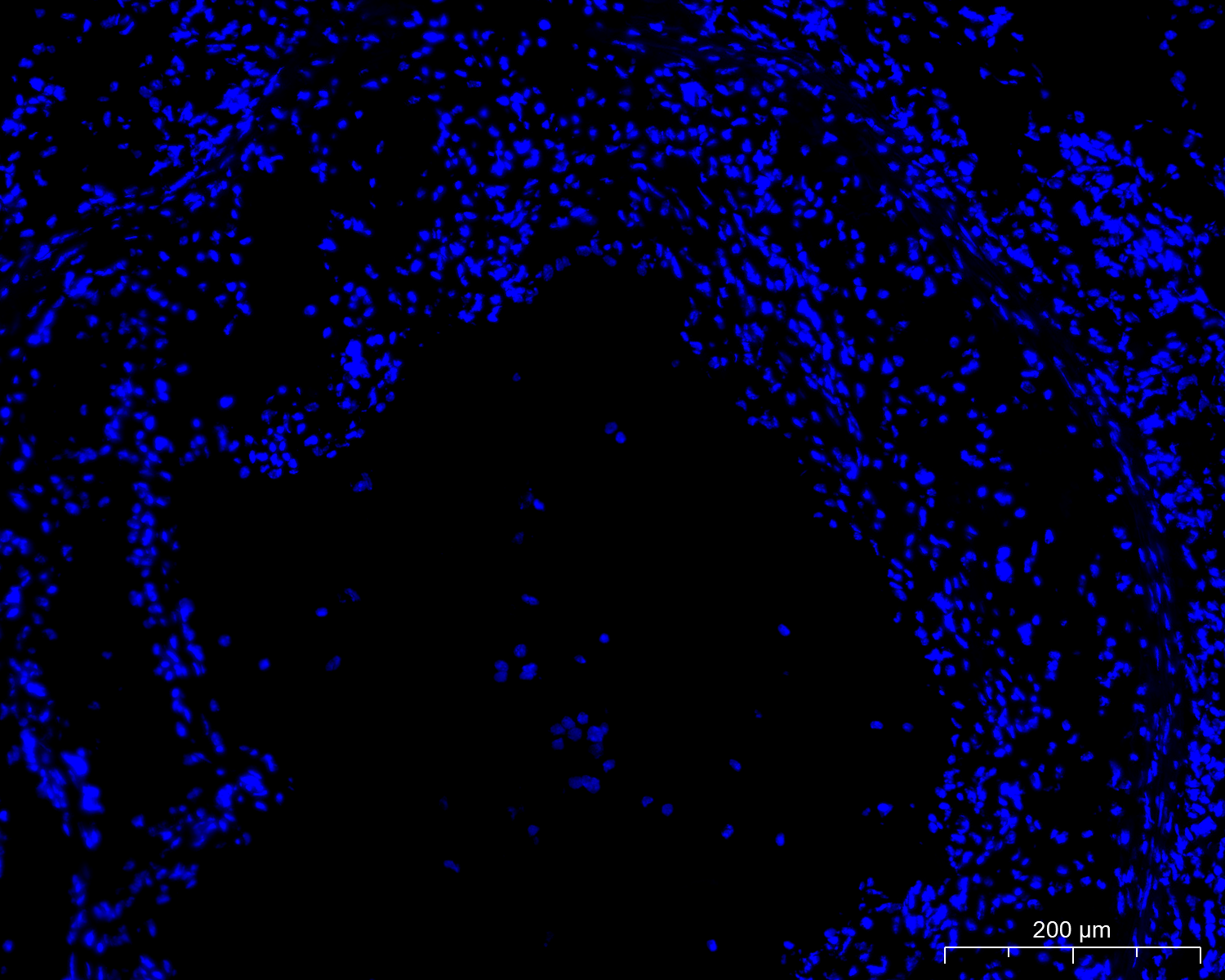

Supplement: S10 File — (ZIP) [file pone.0347758.s010.zip › 主动脉ROS/DAPI/statin/38 ROS红_20.0x.tif]

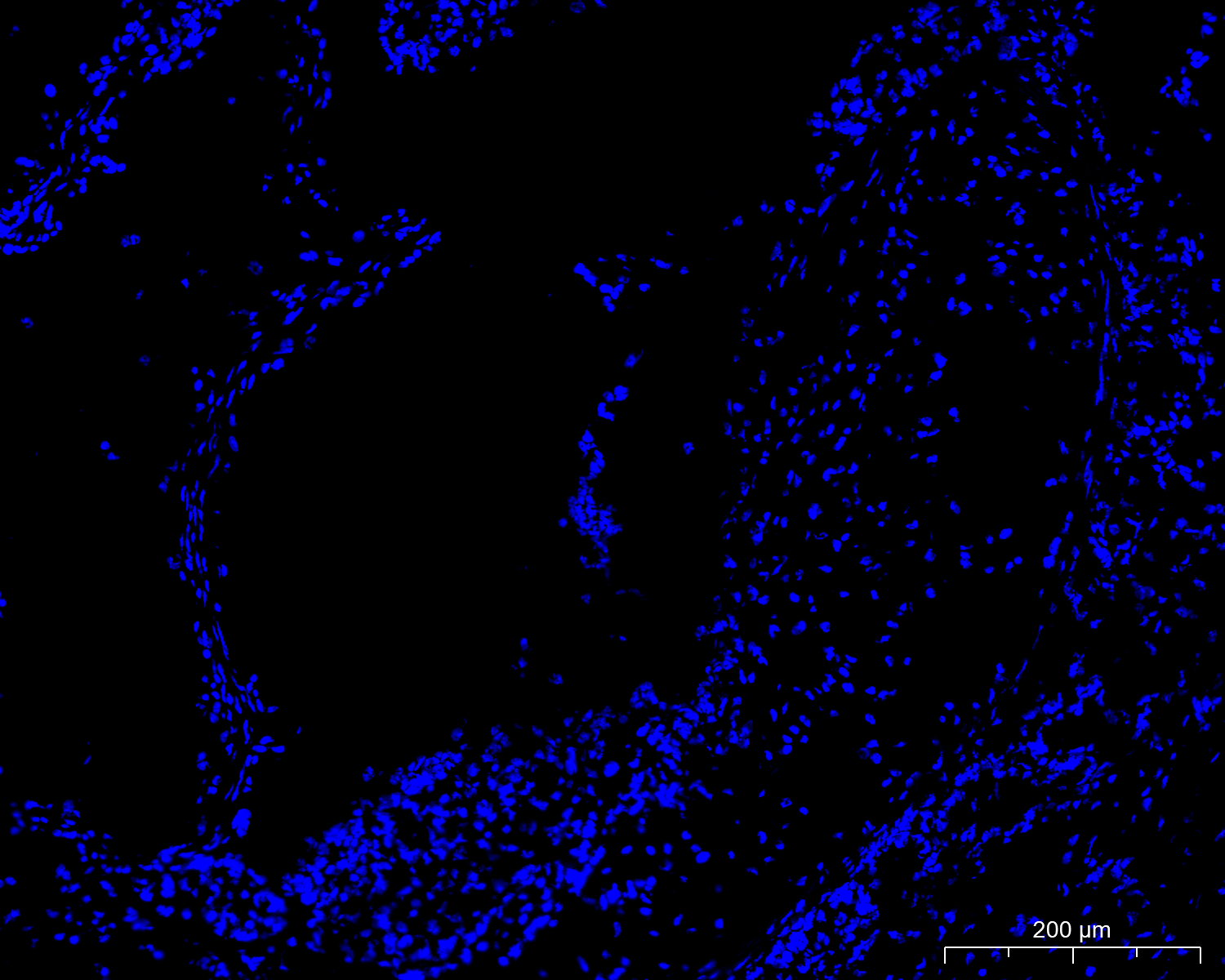

Supplement: S10 File — (ZIP) [file pone.0347758.s010.zip › 主动脉ROS/DAPI/statin/40 ROS红_20.0x.tif]

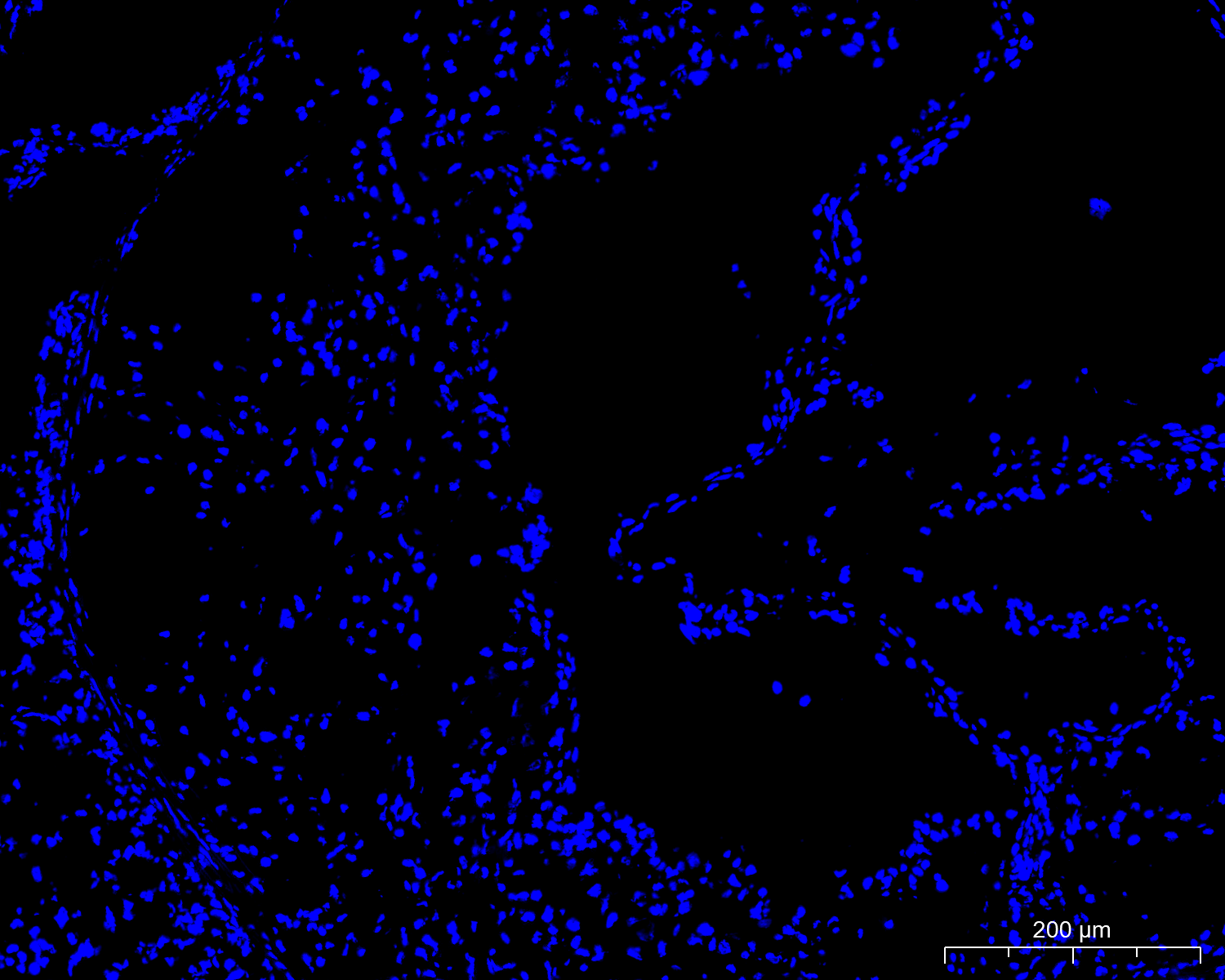

Supplement: S10 File — (ZIP) [file pone.0347758.s010.zip › 主动脉ROS/DAPI/statin/41 ROS红_20.0x.tif]

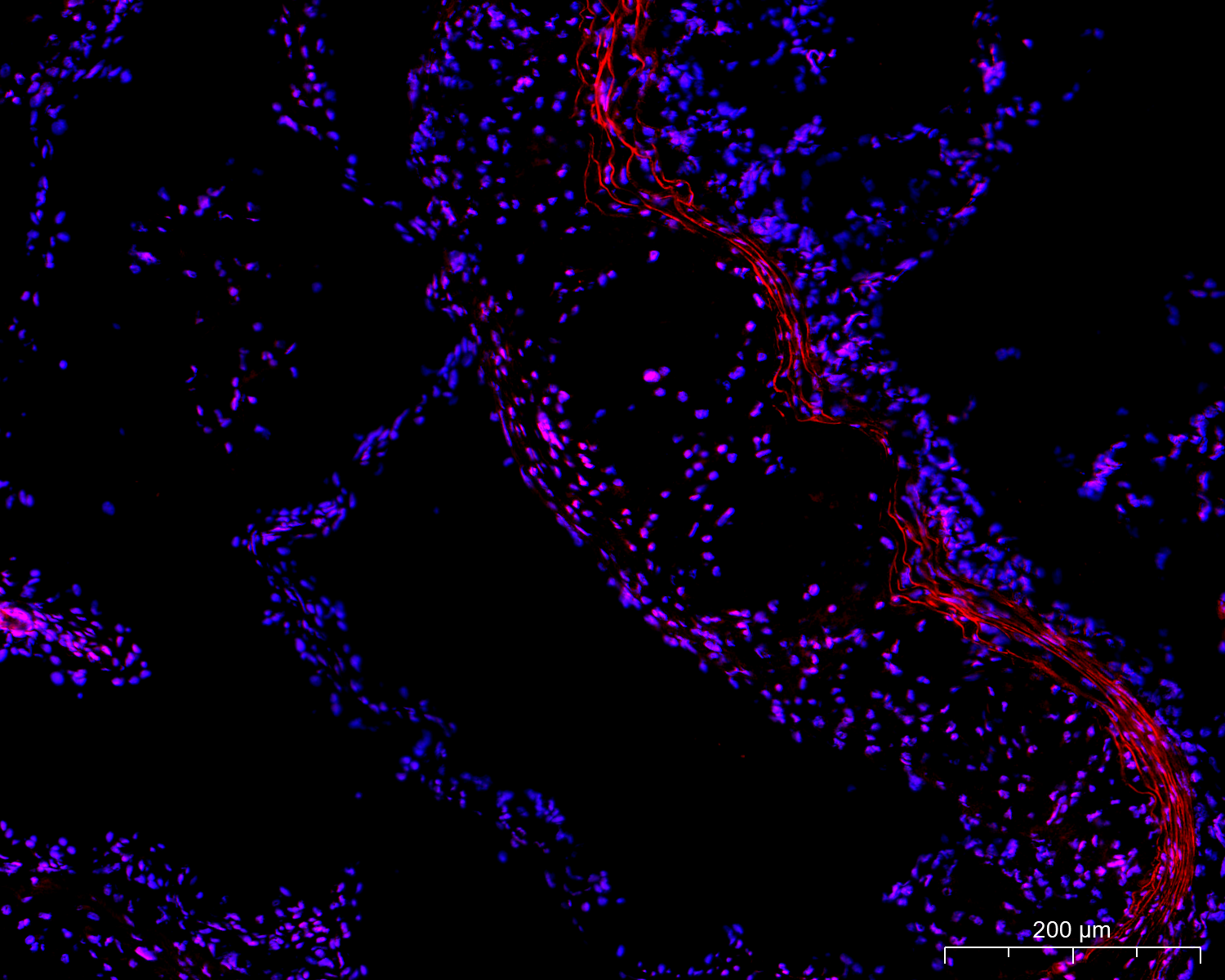

Supplement: S10 File — (ZIP) [file pone.0347758.s010.zip › 主动脉ROS/merge/AS/23 ROS红_20.0x.tif]

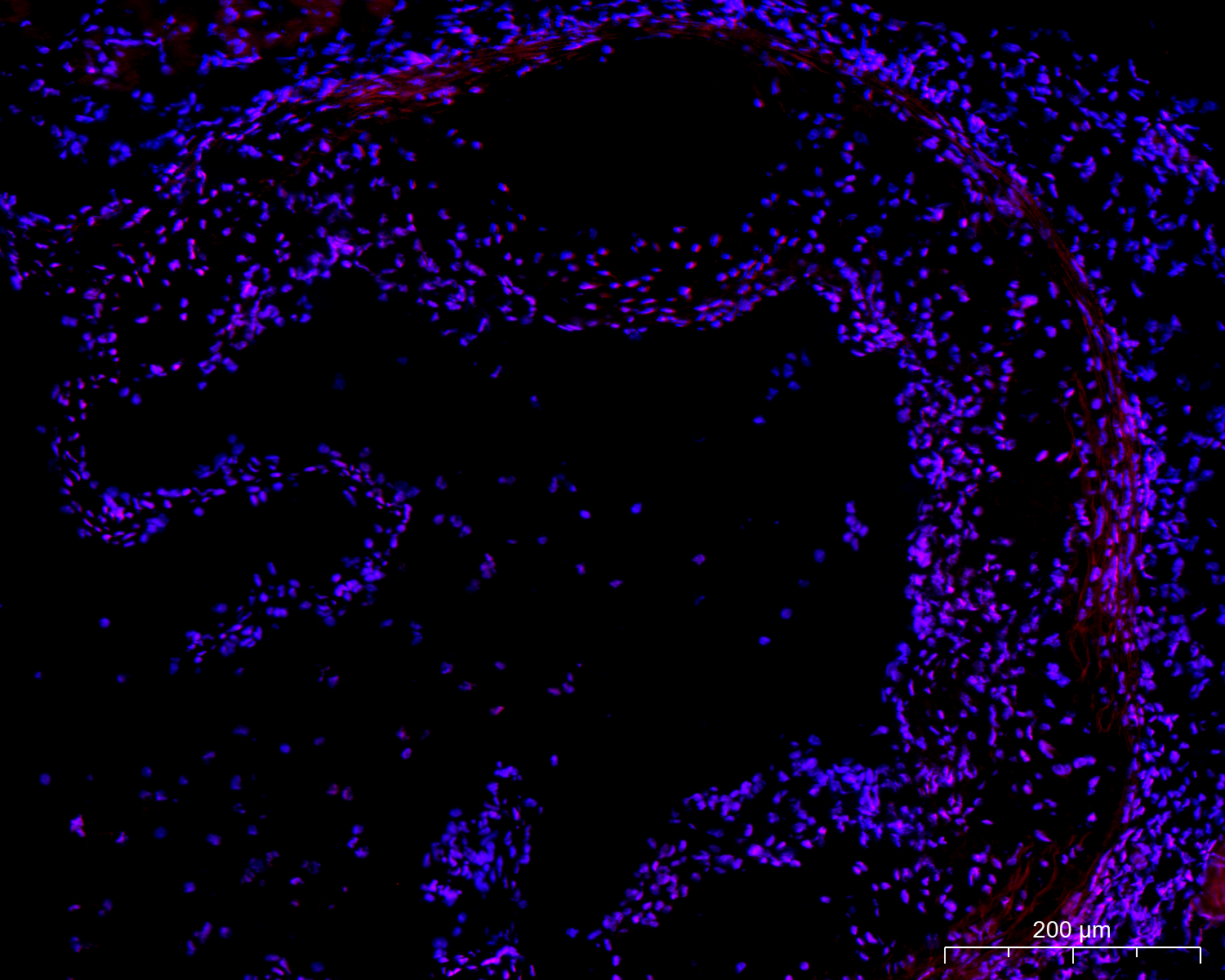

Supplement: S10 File — (ZIP) [file pone.0347758.s010.zip › 主动脉ROS/merge/AS/27 ROS红_20.0x.tif]

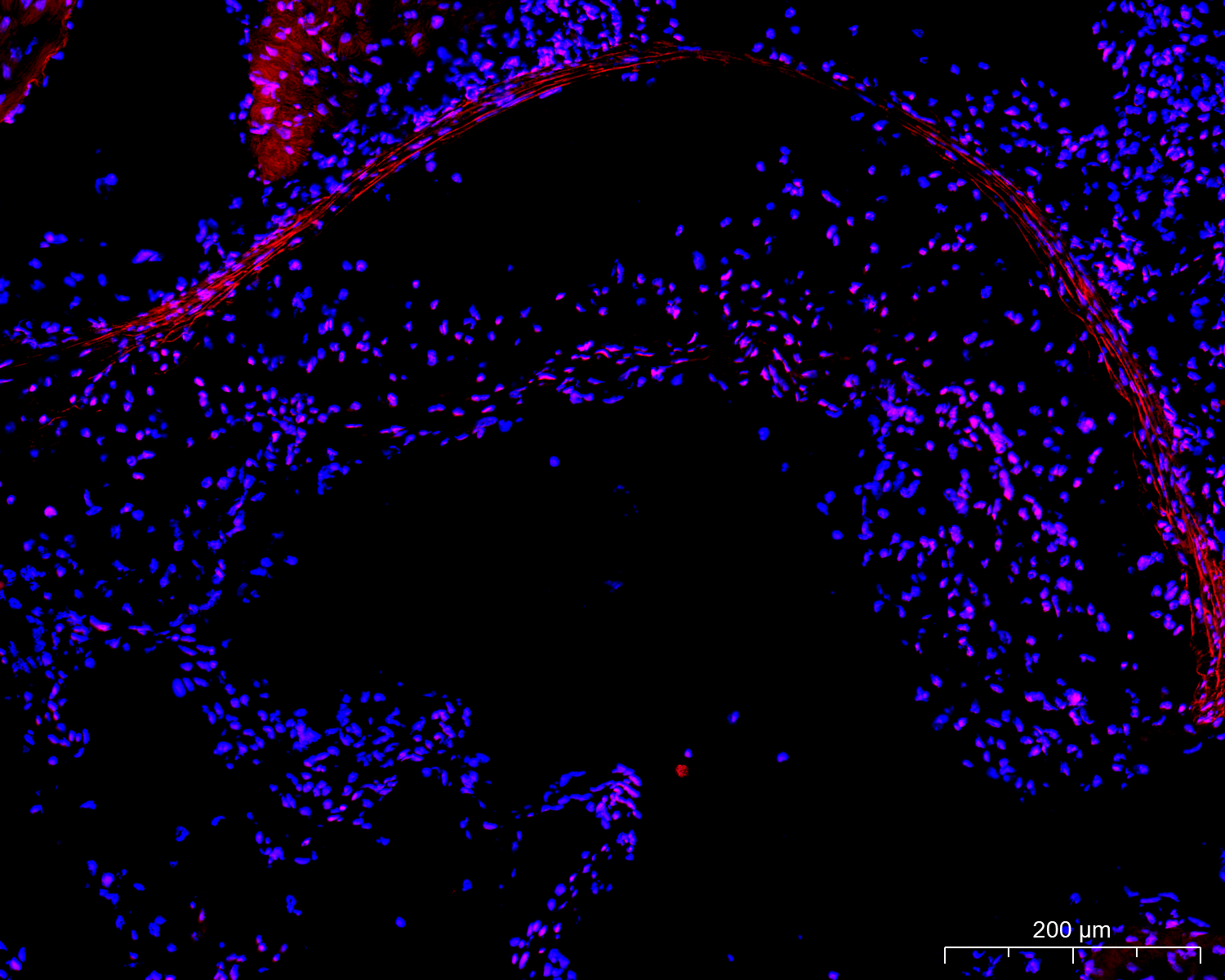

Supplement: S10 File — (ZIP) [file pone.0347758.s010.zip › 主动脉ROS/merge/AS/28 ROS红_20.0x.tif]

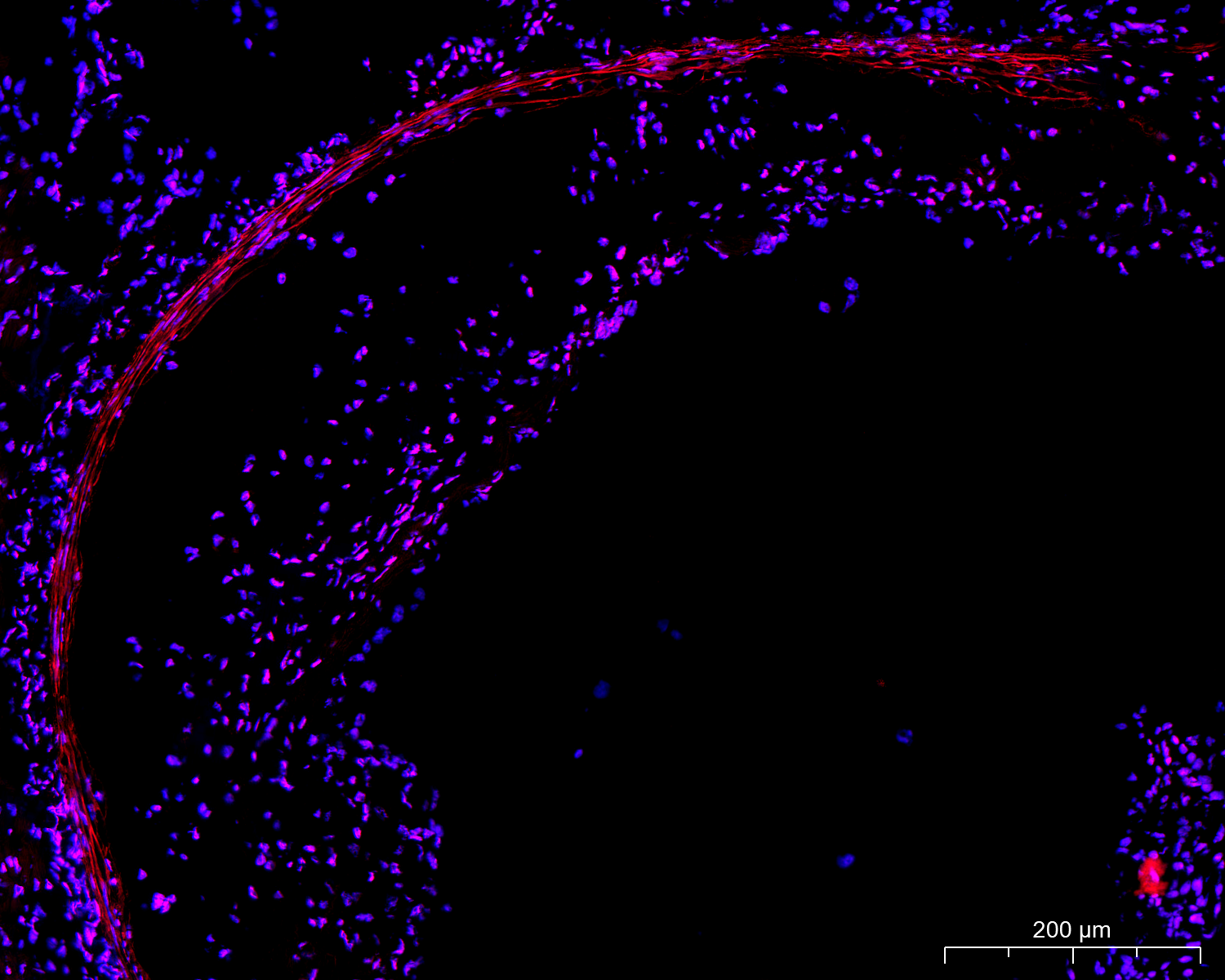

Supplement: S10 File — (ZIP) [file pone.0347758.s010.zip › 主动脉ROS/merge/AS/31 ROS红_20.0x.tif]

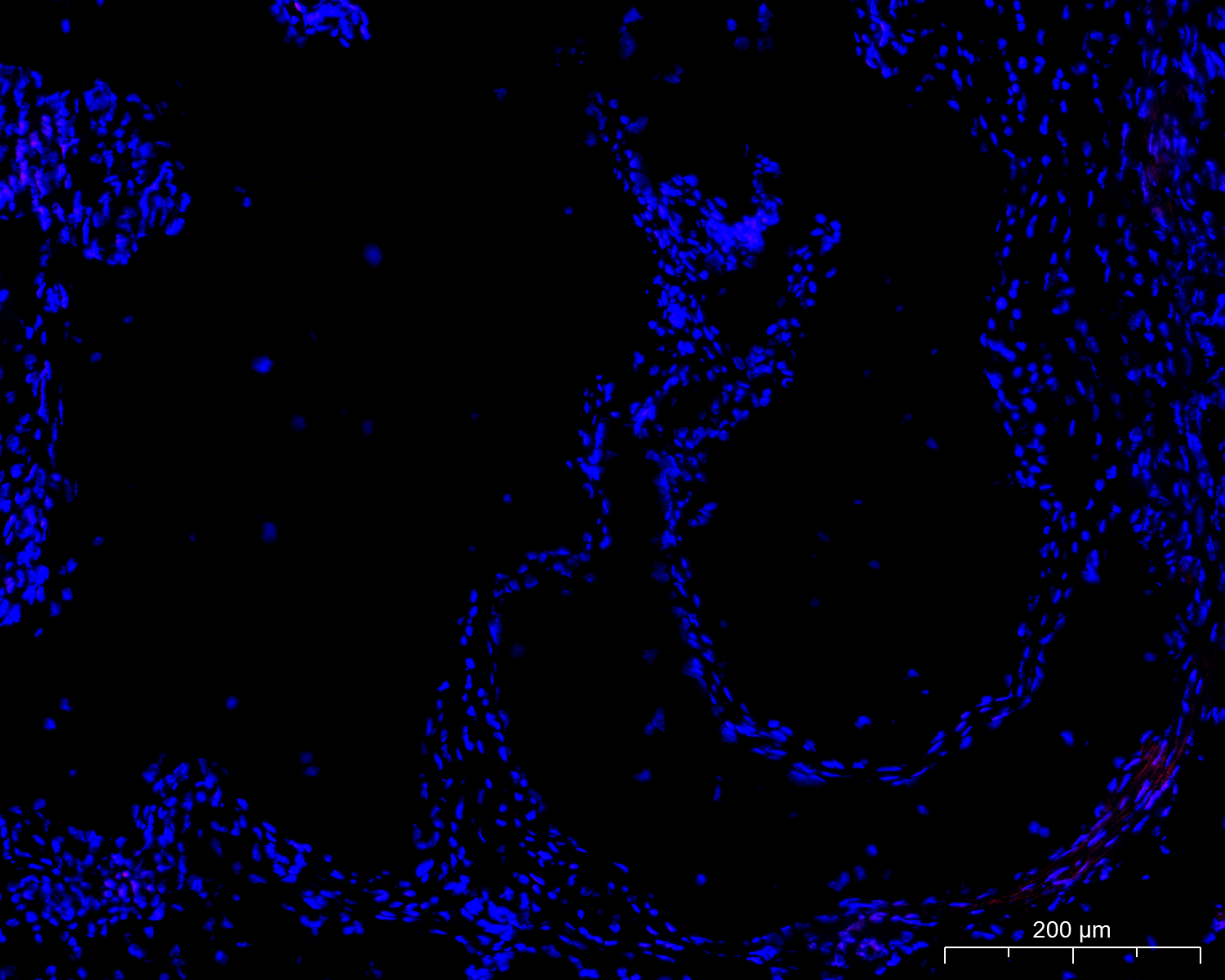

Supplement: S10 File — (ZIP) [file pone.0347758.s010.zip › 主动脉ROS/merge/control/1 ROS红_20.0x.tif]

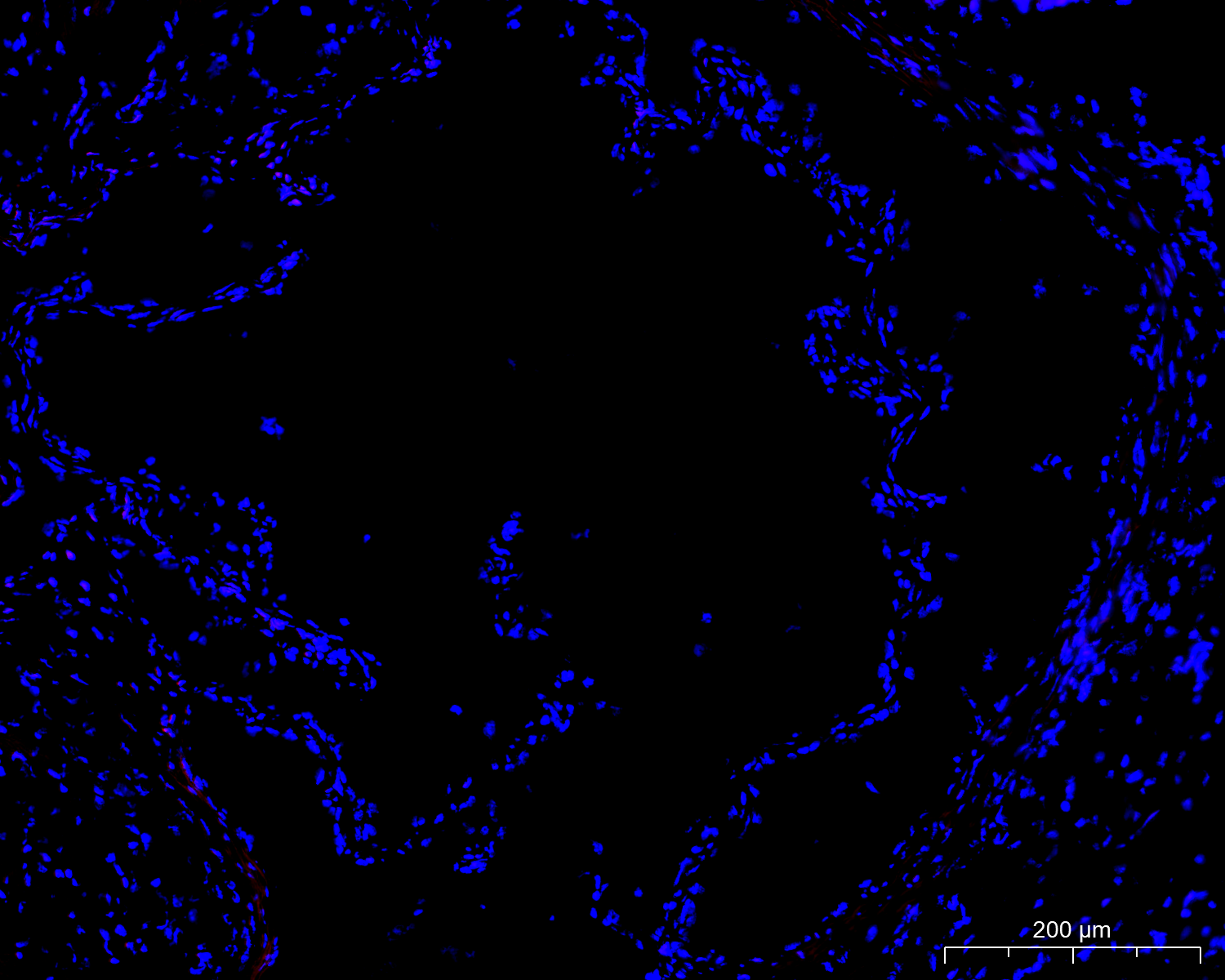

Supplement: S10 File — (ZIP) [file pone.0347758.s010.zip › 主动脉ROS/merge/control/2 ROS红_20.0x.tif]

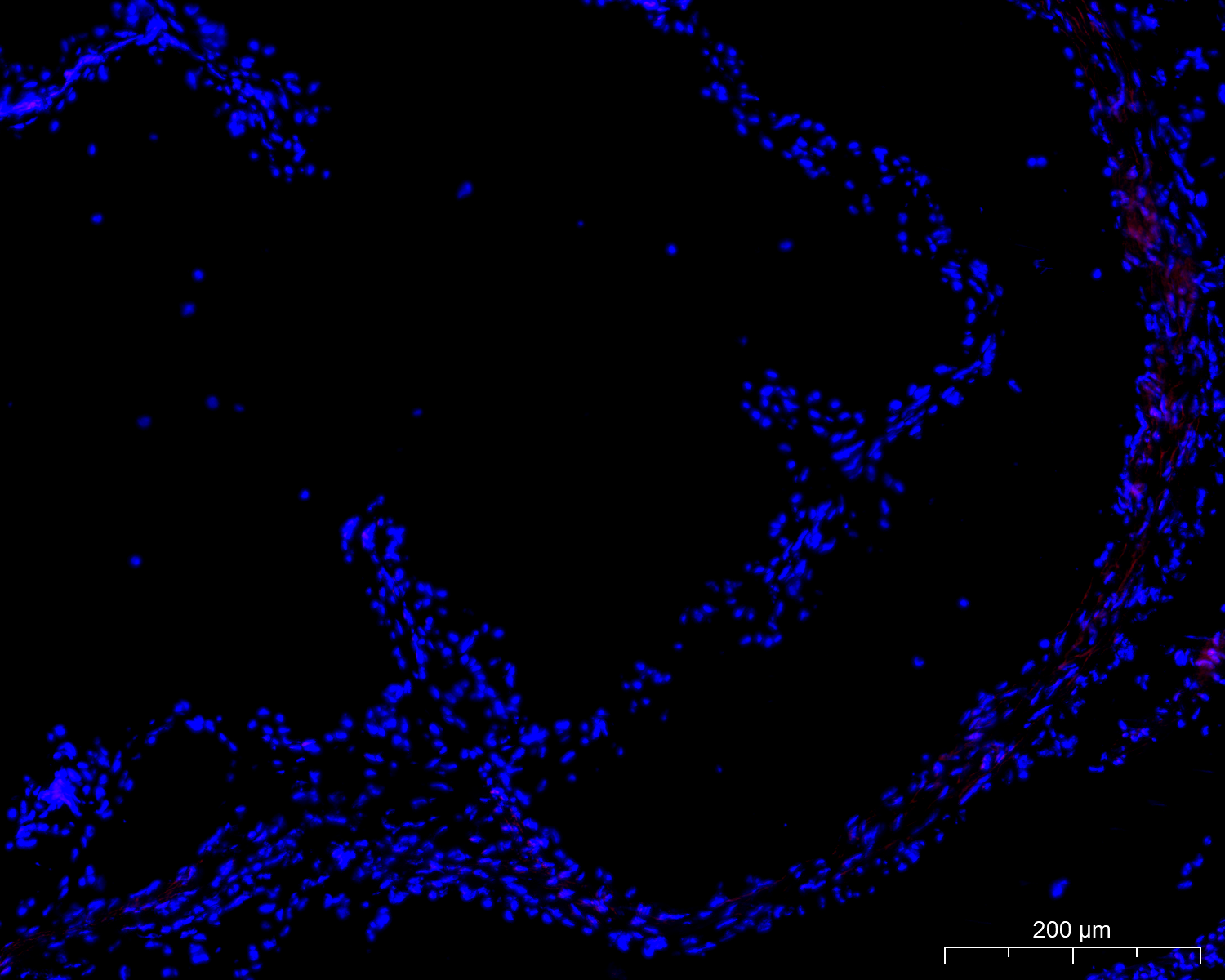

Supplement: S10 File — (ZIP) [file pone.0347758.s010.zip › 主动脉ROS/merge/control/6 ROS红_20.0x.tif]

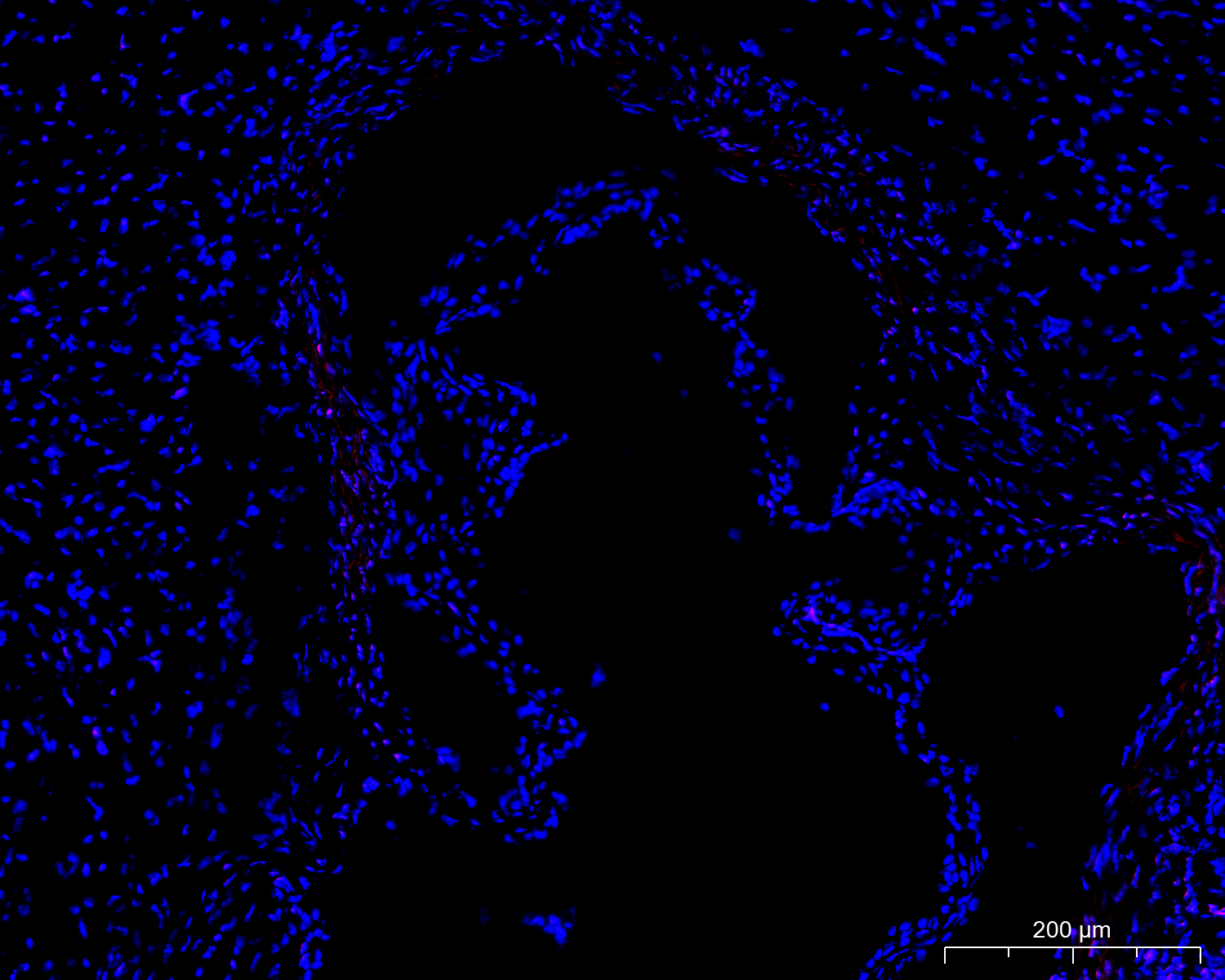

Supplement: S10 File — (ZIP) [file pone.0347758.s010.zip › 主动脉ROS/merge/control/7 ROS红_20.0x.tif]

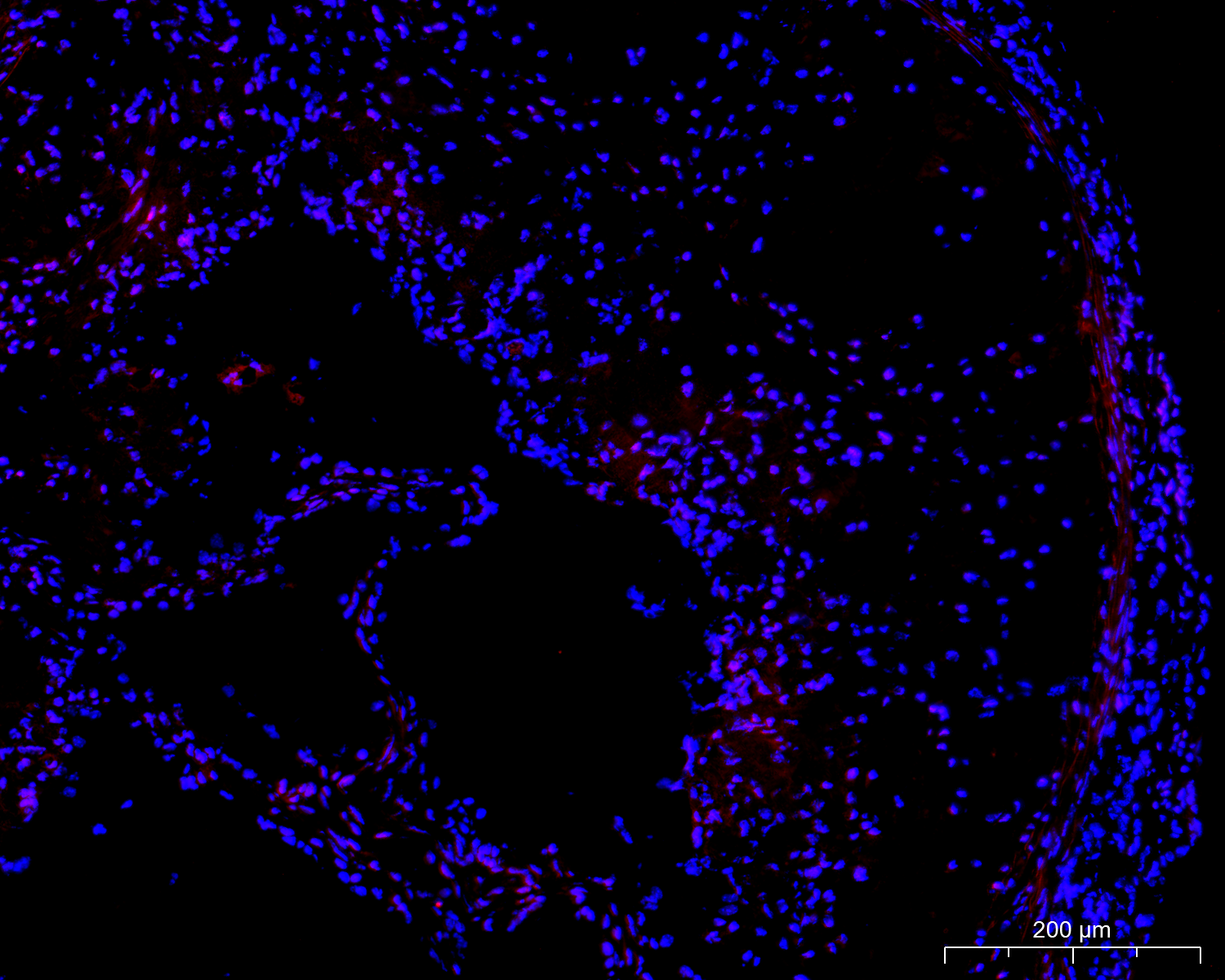

Supplement: S10 File — (ZIP) [file pone.0347758.s010.zip › 主动脉ROS/merge/PSB-H/100 ROS红_20.0x.tif]

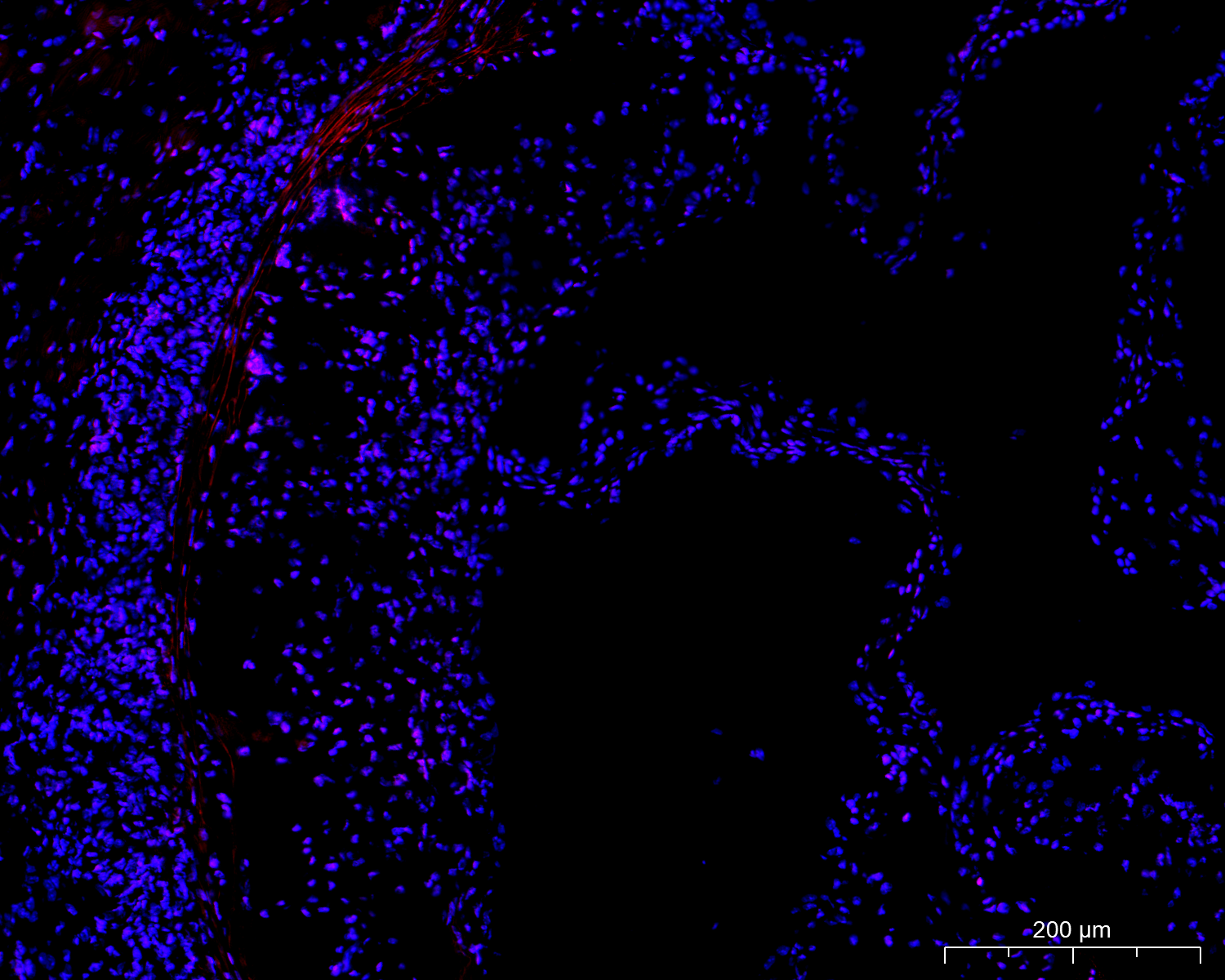

Supplement: S10 File — (ZIP) [file pone.0347758.s010.zip › 主动脉ROS/merge/PSB-H/93 ROS红_20.0x.tif]

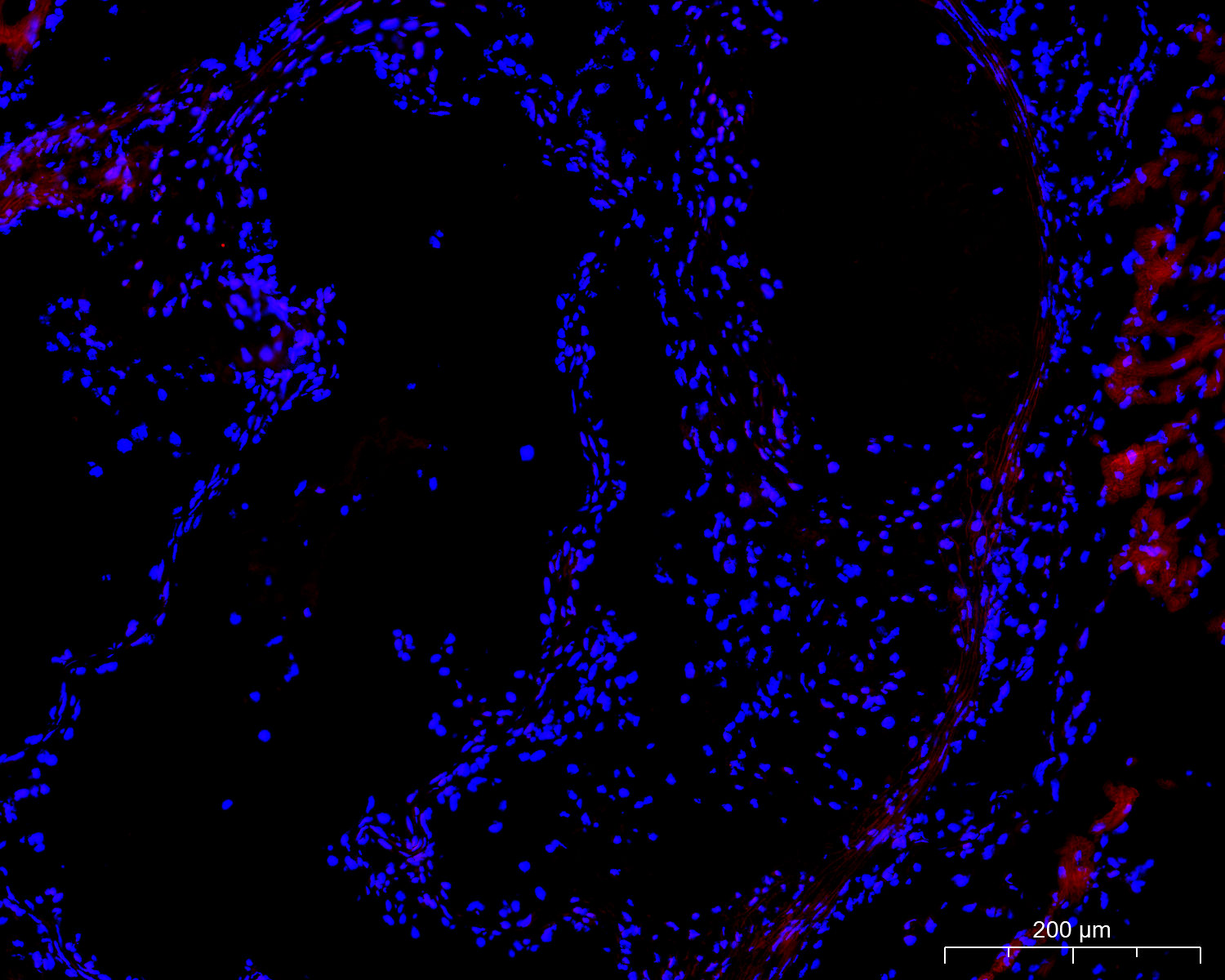

Supplement: S10 File — (ZIP) [file pone.0347758.s010.zip › 主动脉ROS/merge/PSB-H/98 ROS红_20.0x.tif]

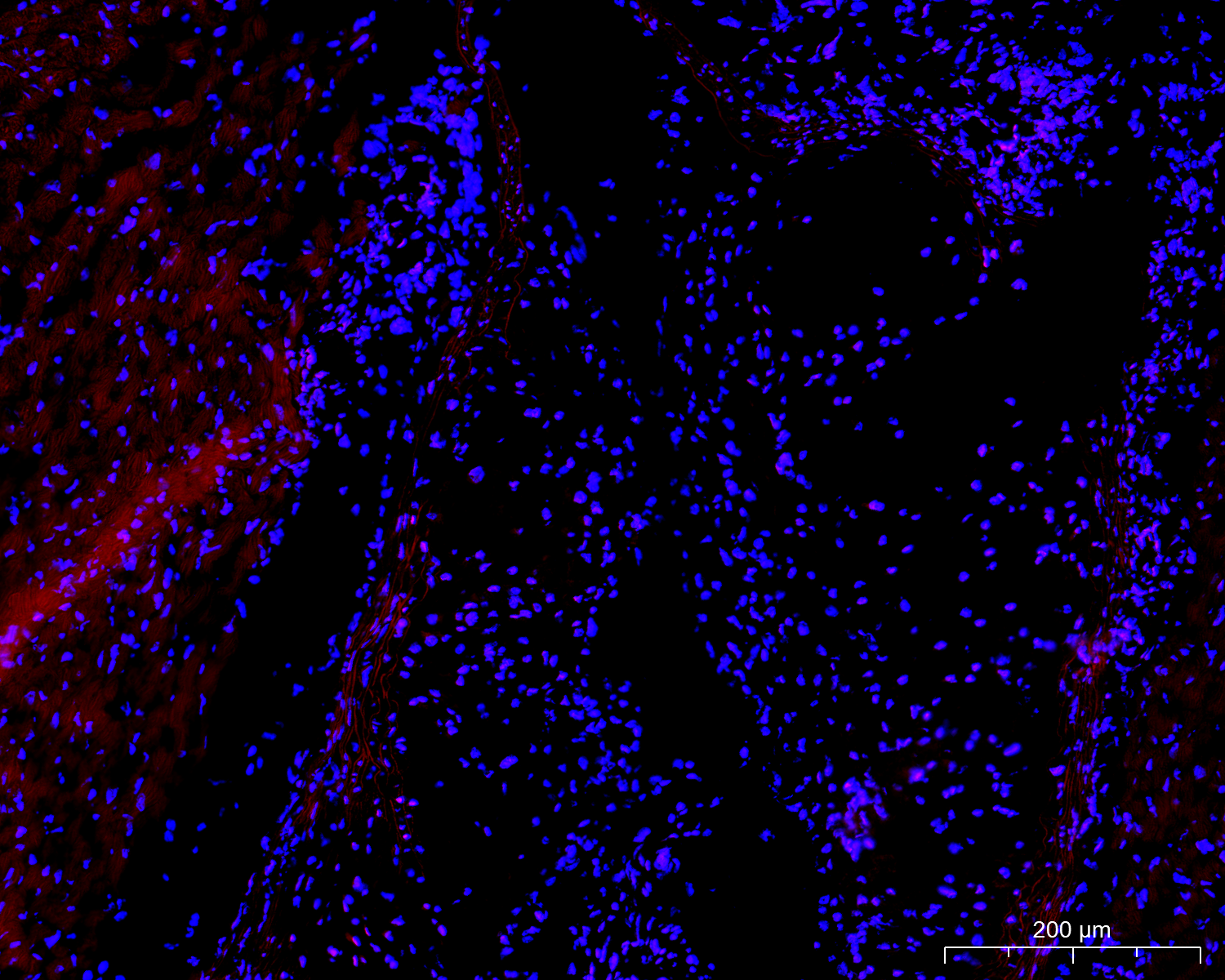

Supplement: S10 File — (ZIP) [file pone.0347758.s010.zip › 主动脉ROS/merge/PSB-H/A1 ROS红_20.0x.tif]

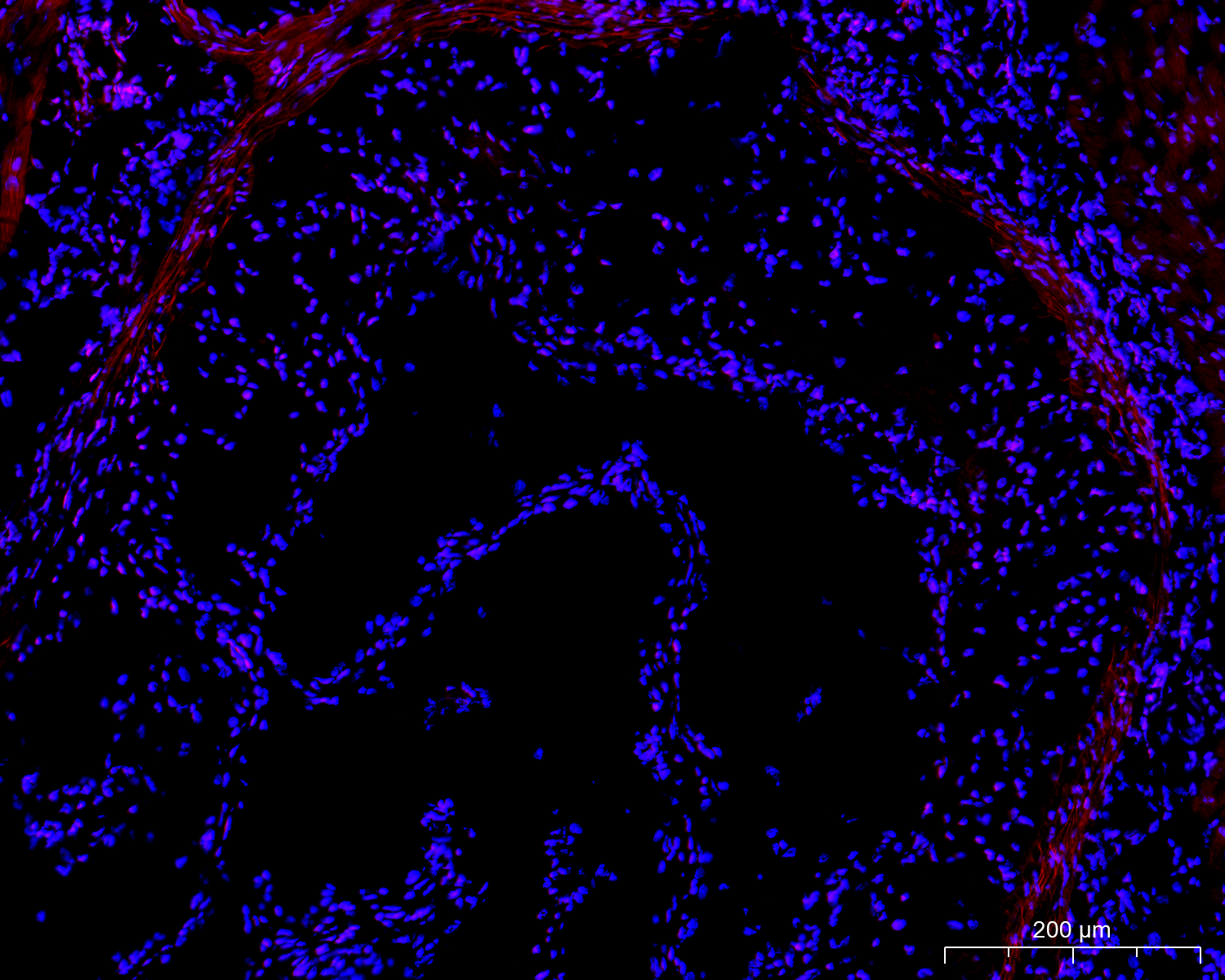

Supplement: S10 File — (ZIP) [file pone.0347758.s010.zip › 主动脉ROS/merge/PSB-L/74 ROS红_20.0x.tif]

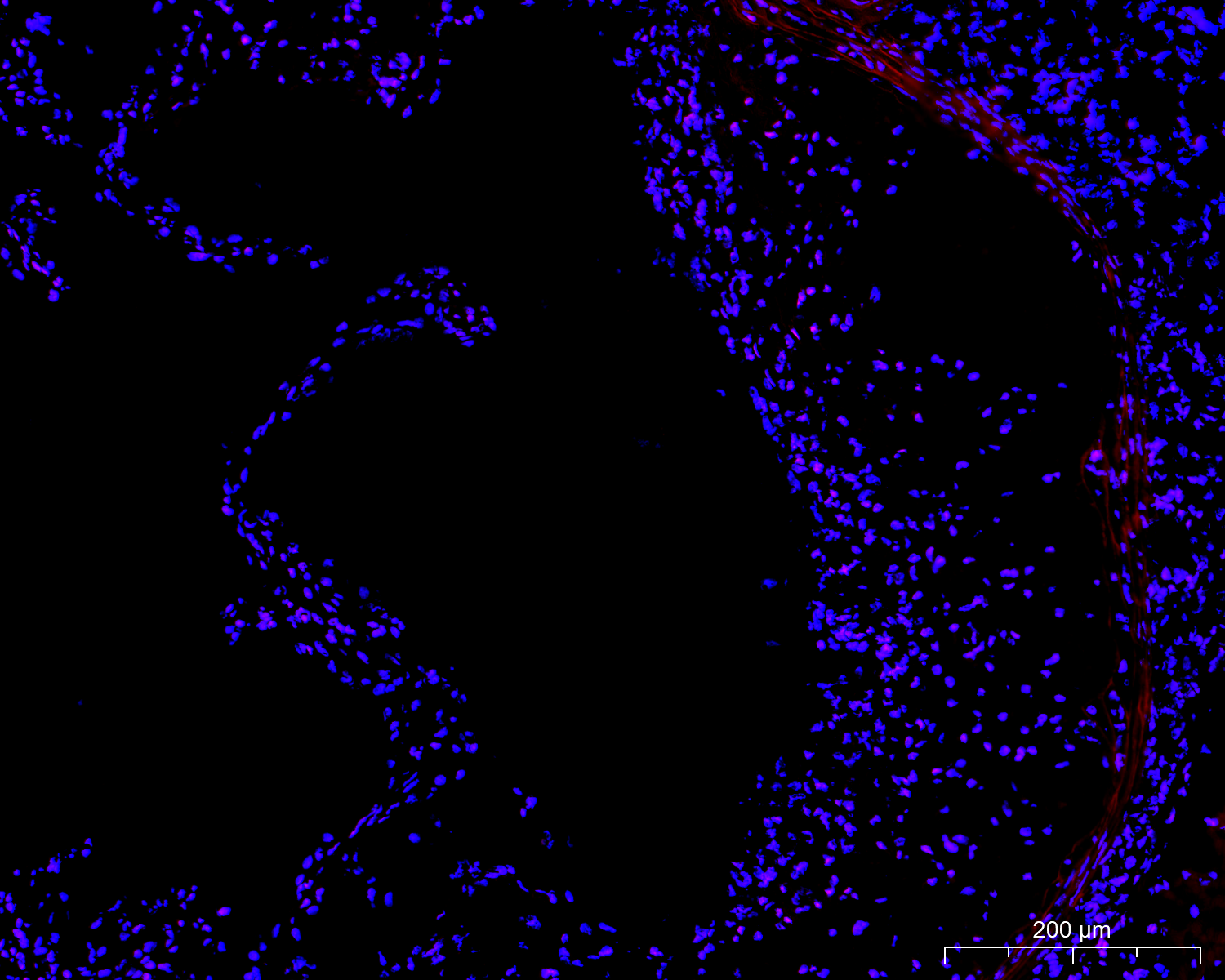

Supplement: S10 File — (ZIP) [file pone.0347758.s010.zip › 主动脉ROS/merge/PSB-L/77 ROS红_20.0x.tif]

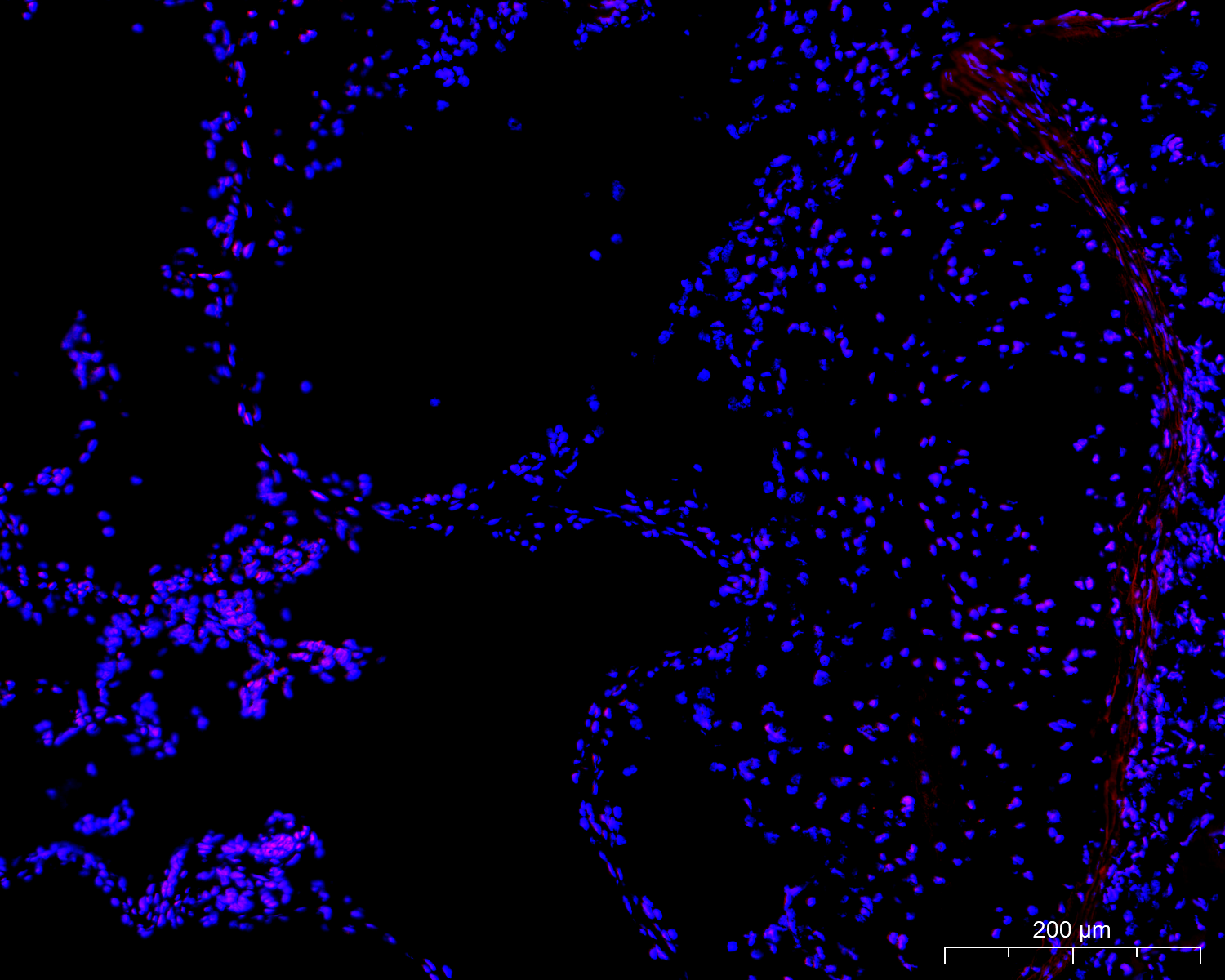

Supplement: S10 File — (ZIP) [file pone.0347758.s010.zip › 主动脉ROS/merge/PSB-L/80 ROS红_20.0x.tif]

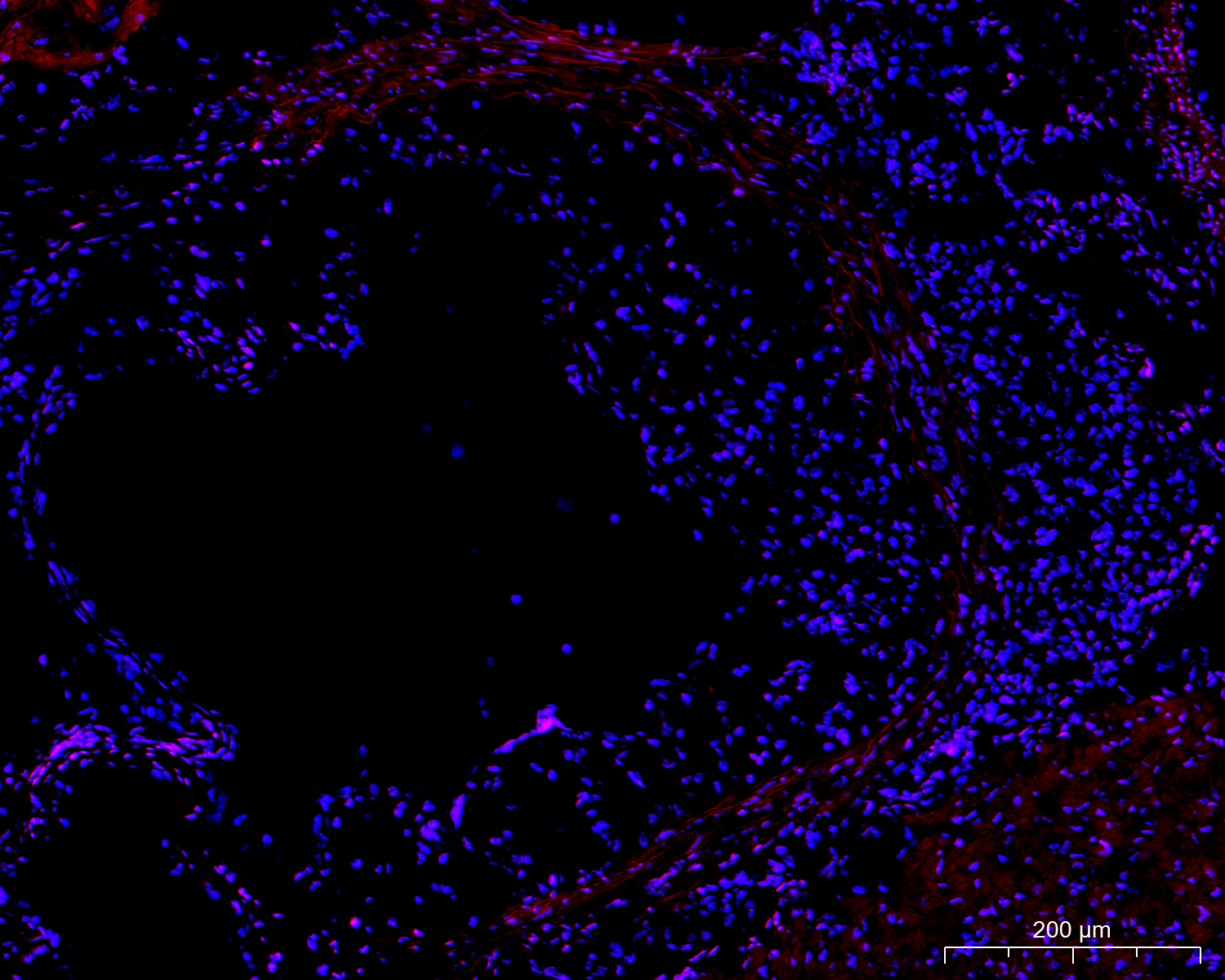

Supplement: S10 File — (ZIP) [file pone.0347758.s010.zip › 主动脉ROS/merge/PSB-L/82 ROS红_20.0x.tif]

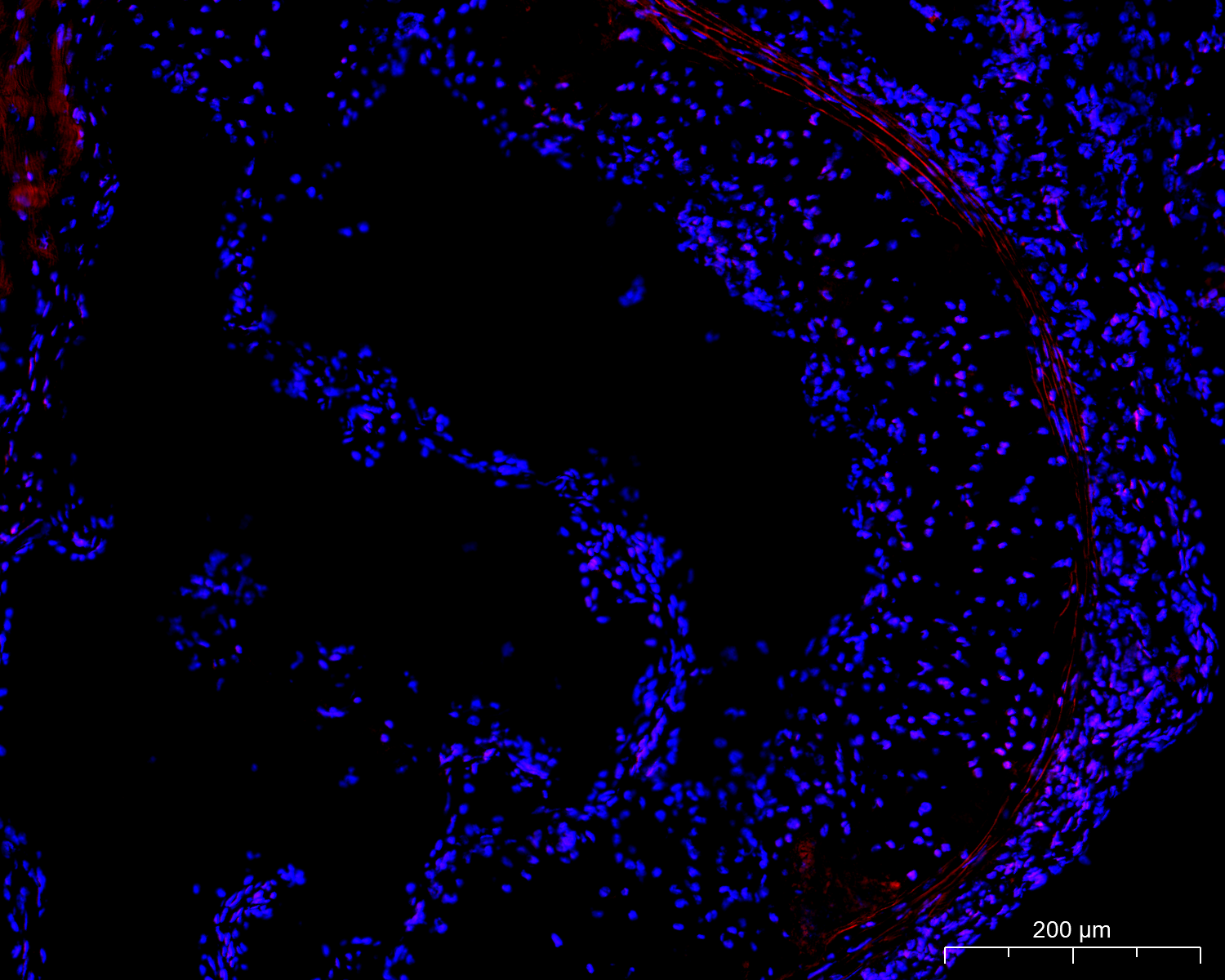

Supplement: S10 File — (ZIP) [file pone.0347758.s010.zip › 主动脉ROS/merge/PSB-M/85 ROS红_20.0x.tif]

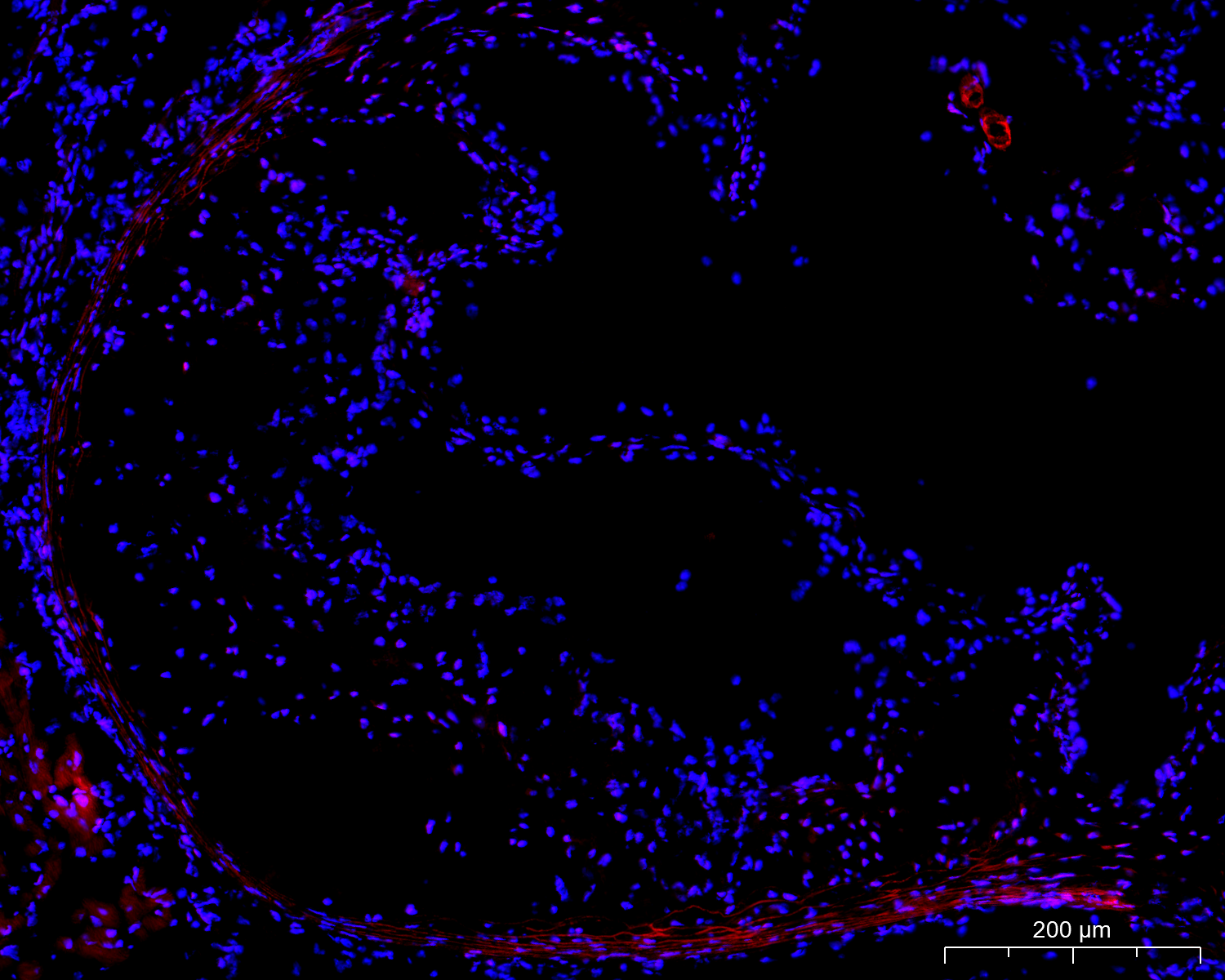

Supplement: S10 File — (ZIP) [file pone.0347758.s010.zip › 主动脉ROS/merge/PSB-M/89 ROS红_20.0x.tif]

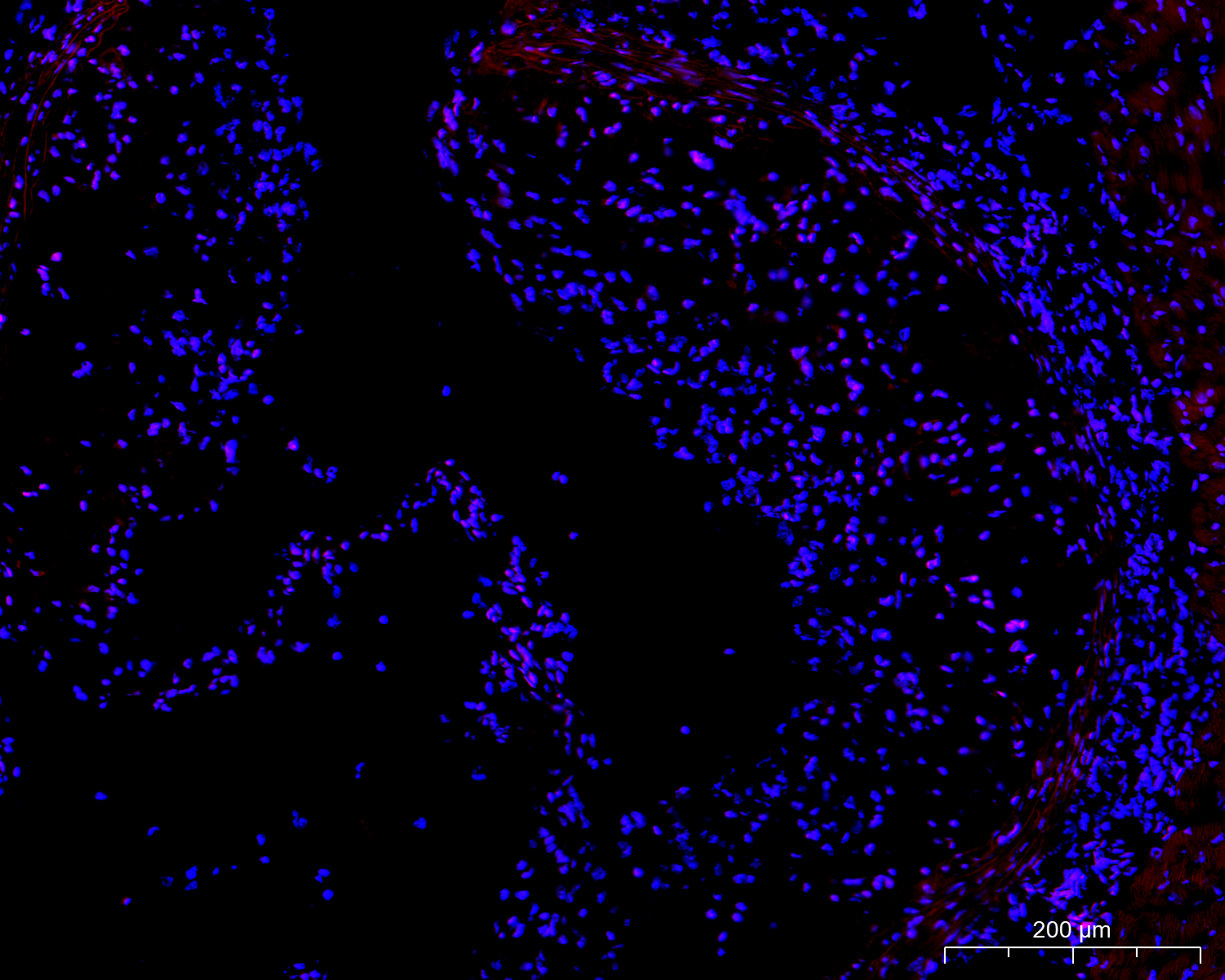

Supplement: S10 File — (ZIP) [file pone.0347758.s010.zip › 主动脉ROS/merge/PSB-M/90 ROS红_20.0x.tif]

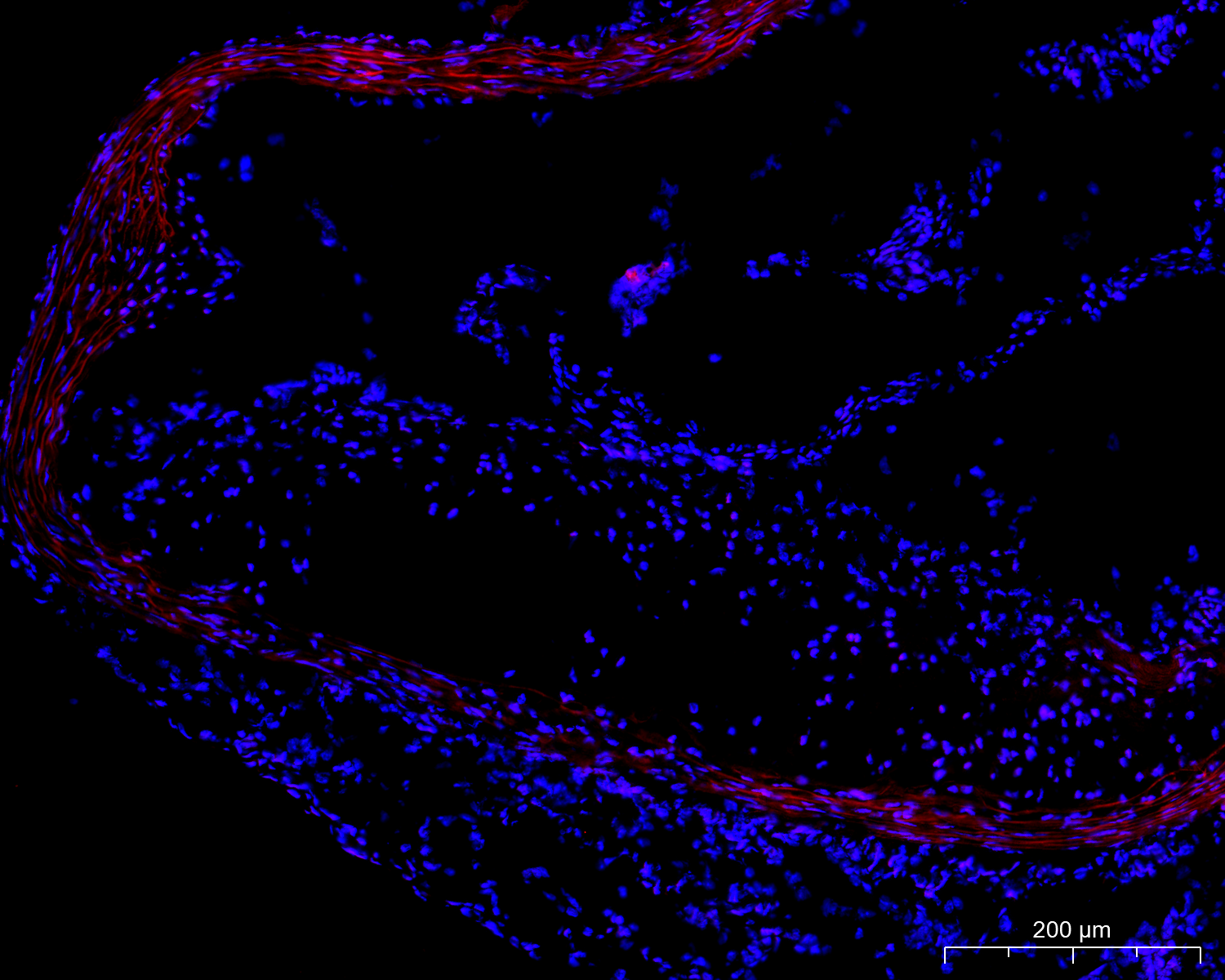

Supplement: S10 File — (ZIP) [file pone.0347758.s010.zip › 主动脉ROS/merge/PSB-M/92 ROS红_20.0x.tif]

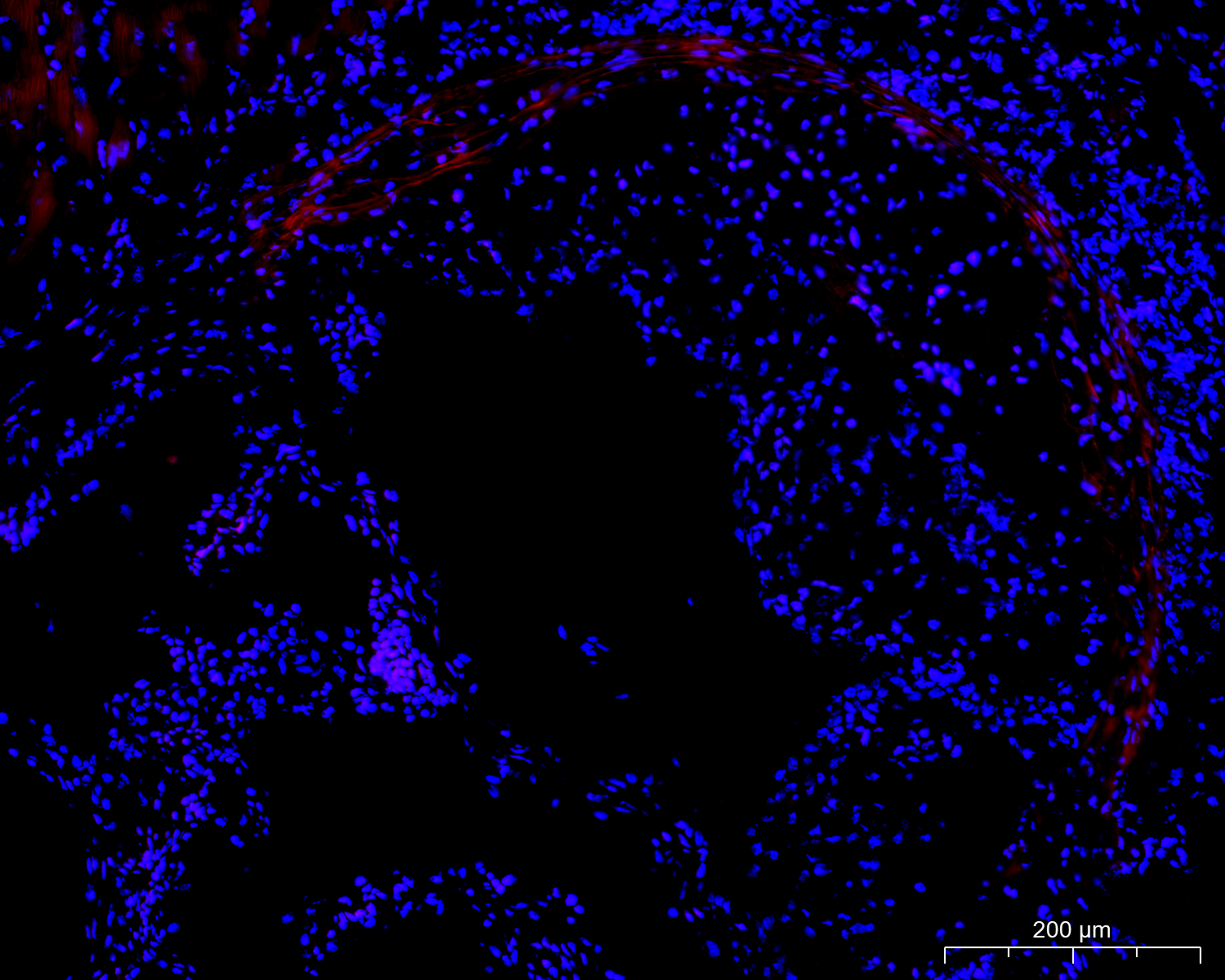

Supplement: S10 File — (ZIP) [file pone.0347758.s010.zip › 主动脉ROS/merge/statin/37 ROS红_20.0x.tif]

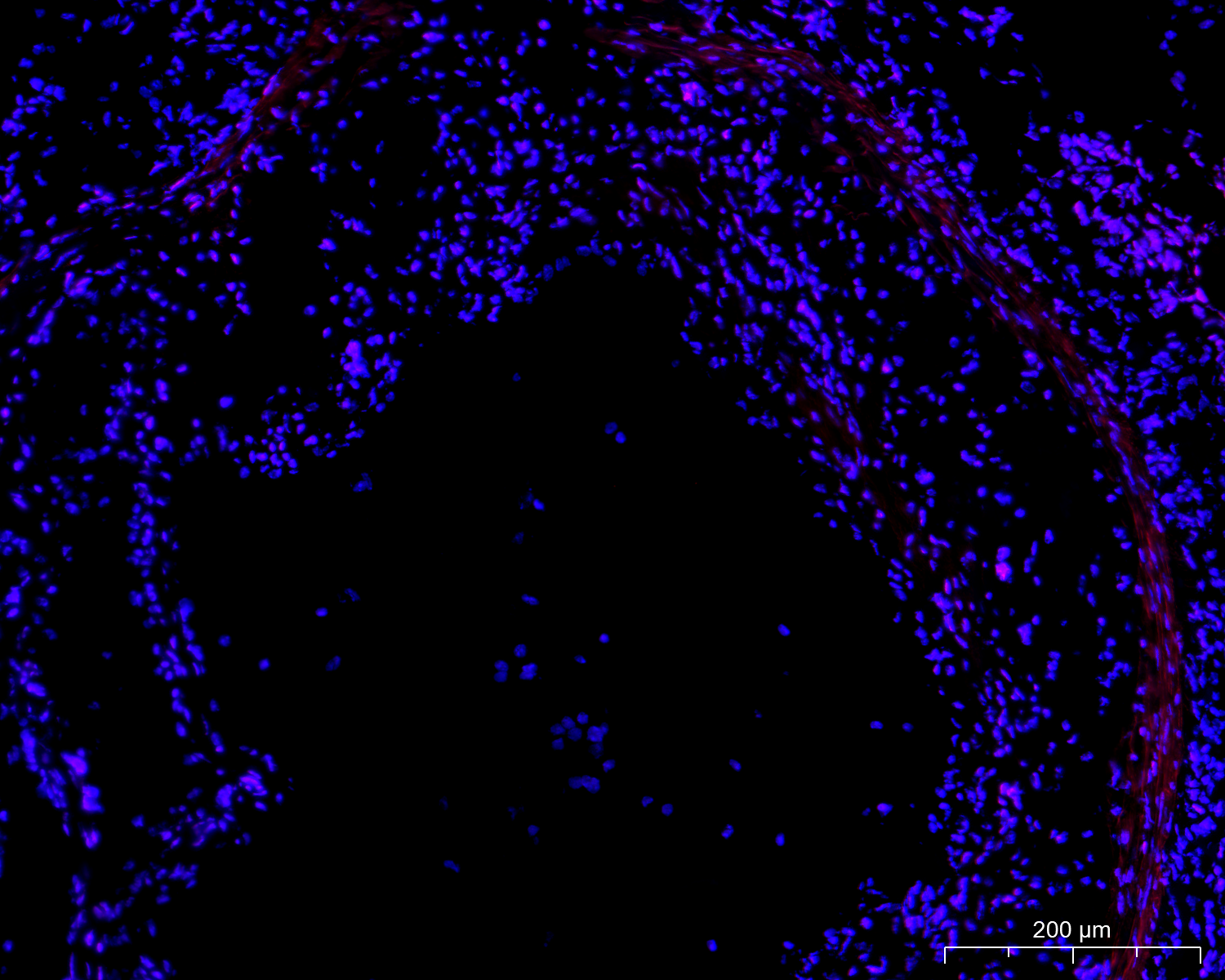

Supplement: S10 File — (ZIP) [file pone.0347758.s010.zip › 主动脉ROS/merge/statin/38 ROS红_20.0x.tif]

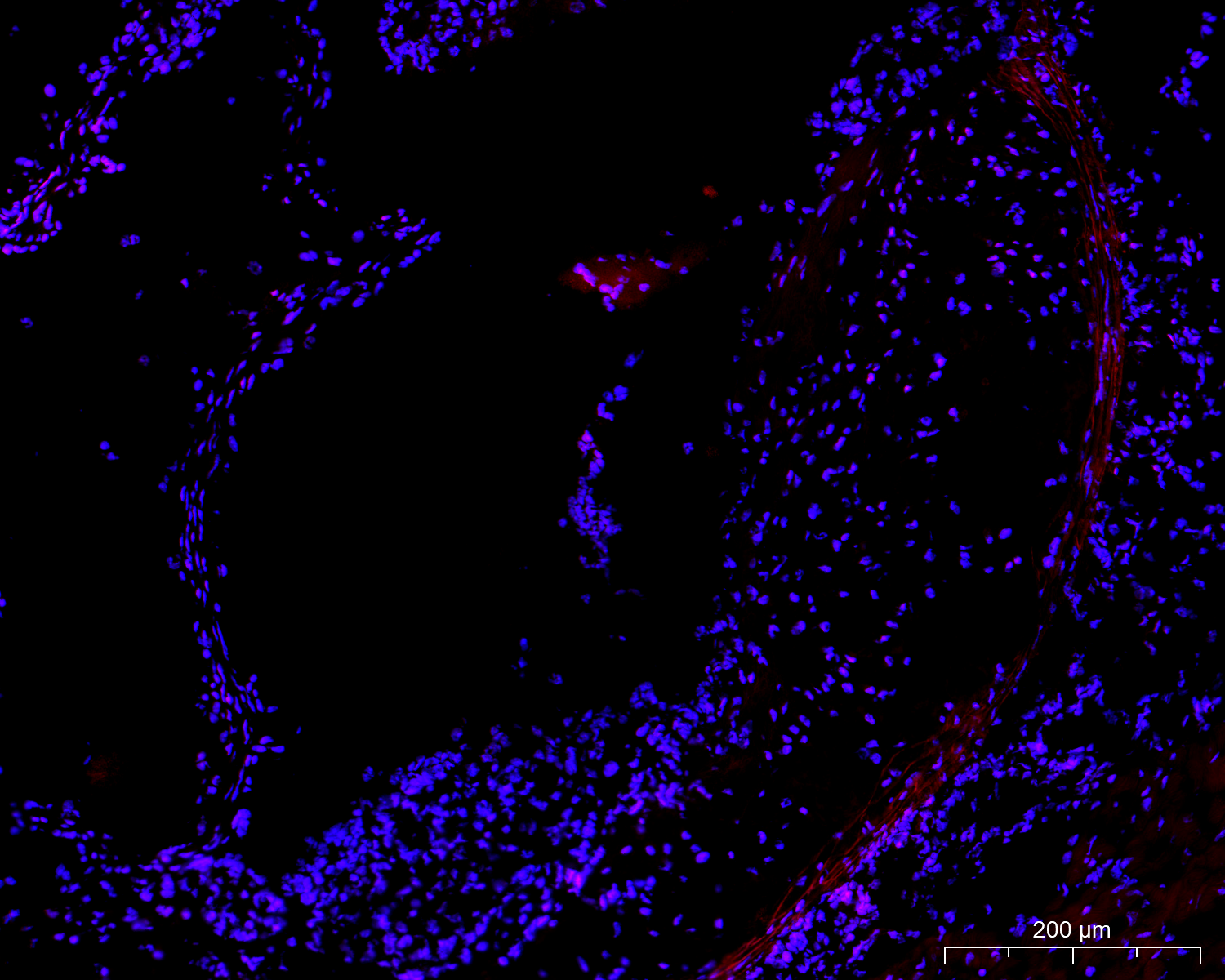

Supplement: S10 File — (ZIP) [file pone.0347758.s010.zip › 主动脉ROS/merge/statin/40 ROS红_20.0x.tif]

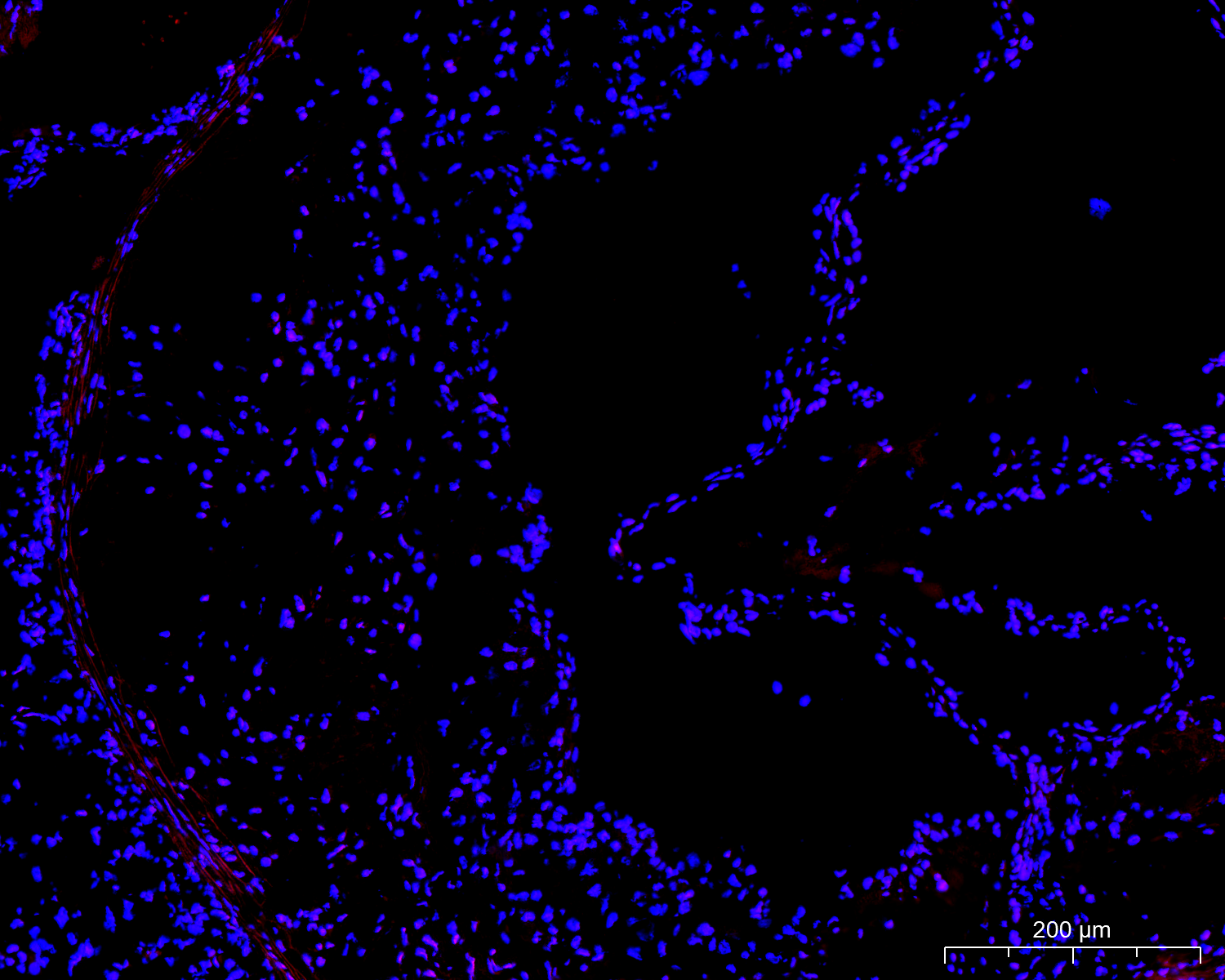

Supplement: S10 File — (ZIP) [file pone.0347758.s010.zip › 主动脉ROS/merge/statin/41 ROS红_20.0x.tif]

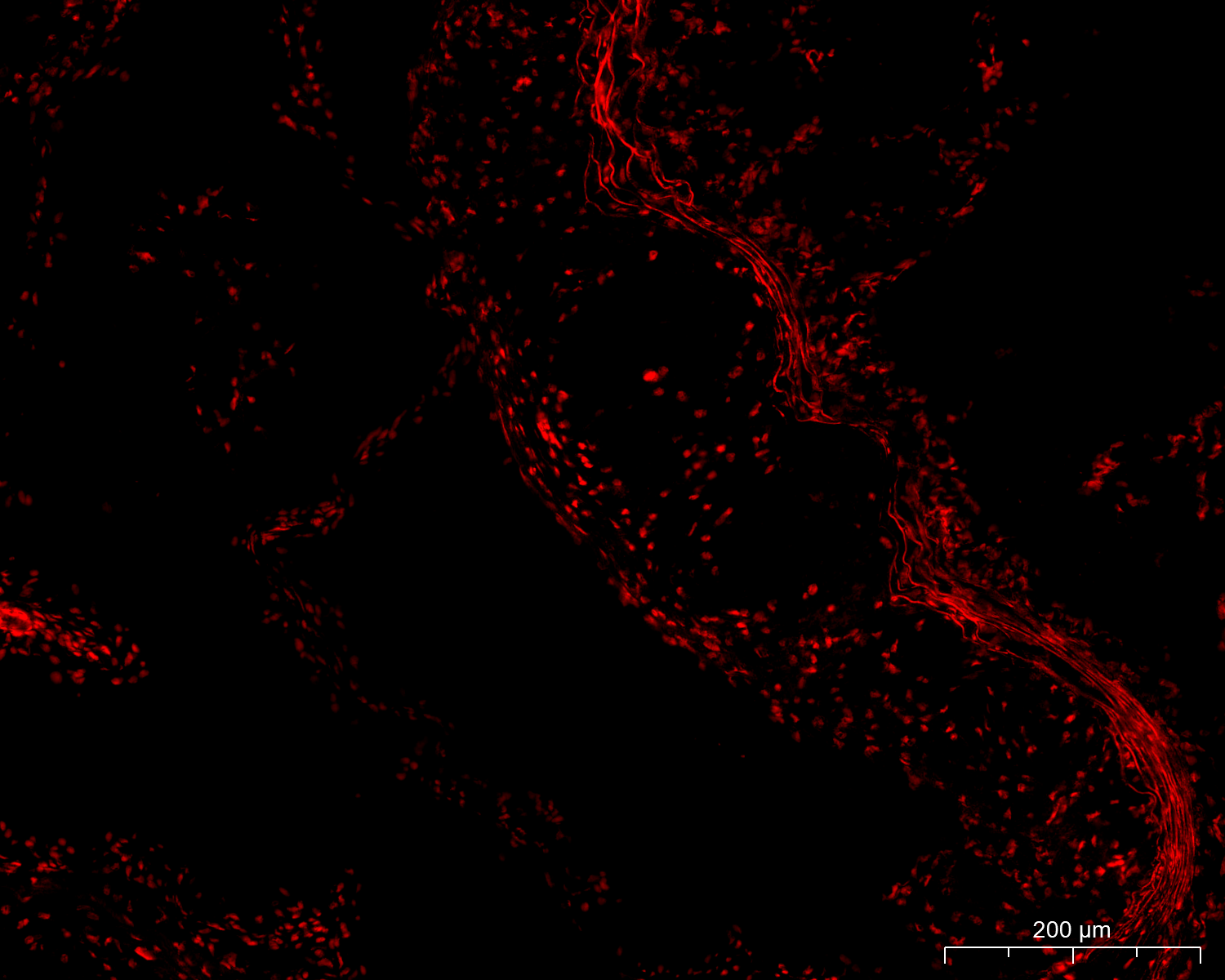

Supplement: S10 File — (ZIP) [file pone.0347758.s010.zip › 主动脉ROS/ROS/AS/23 ROS红_20.0x.tif]

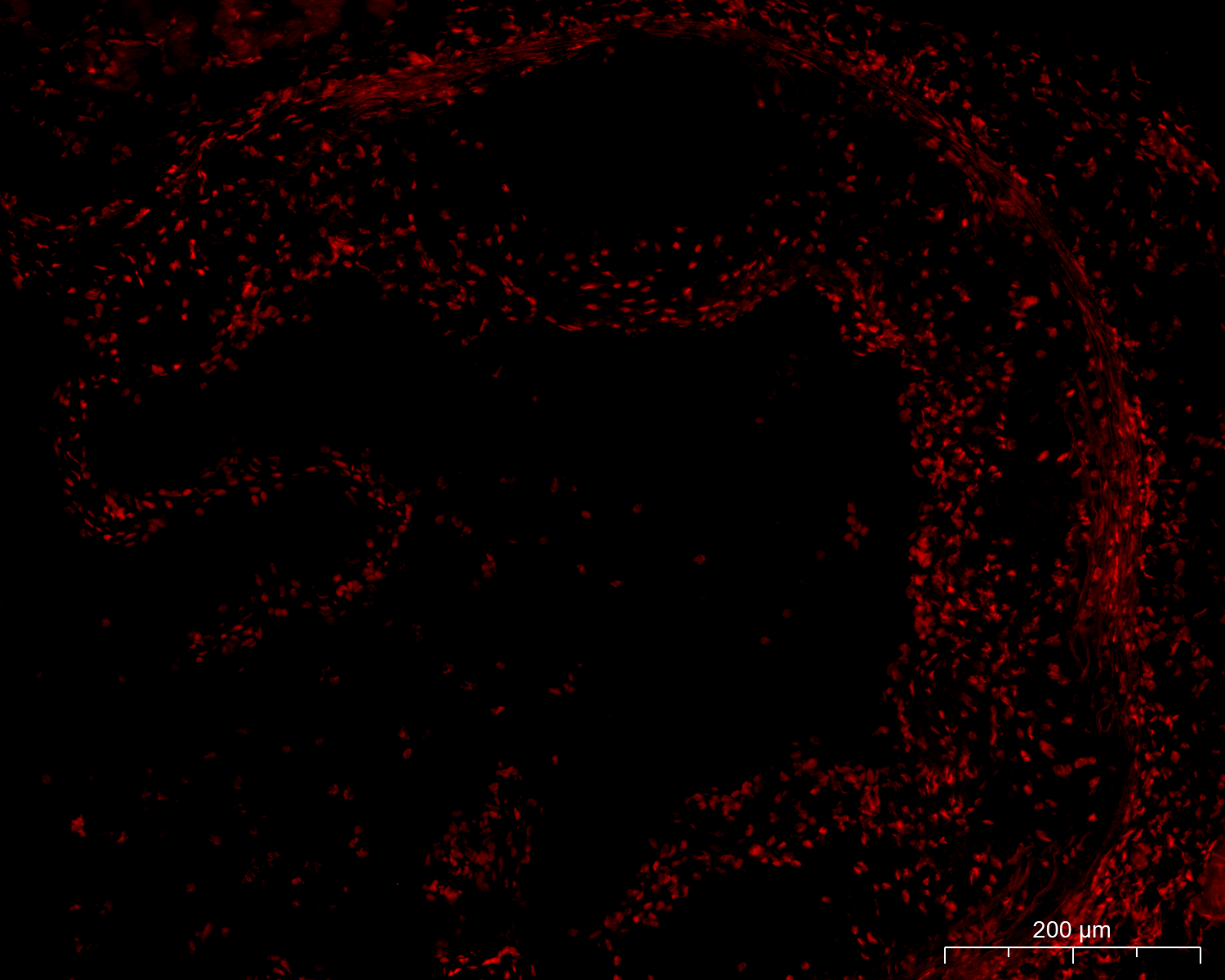

Supplement: S10 File — (ZIP) [file pone.0347758.s010.zip › 主动脉ROS/ROS/AS/27 ROS红_20.0x.tif]

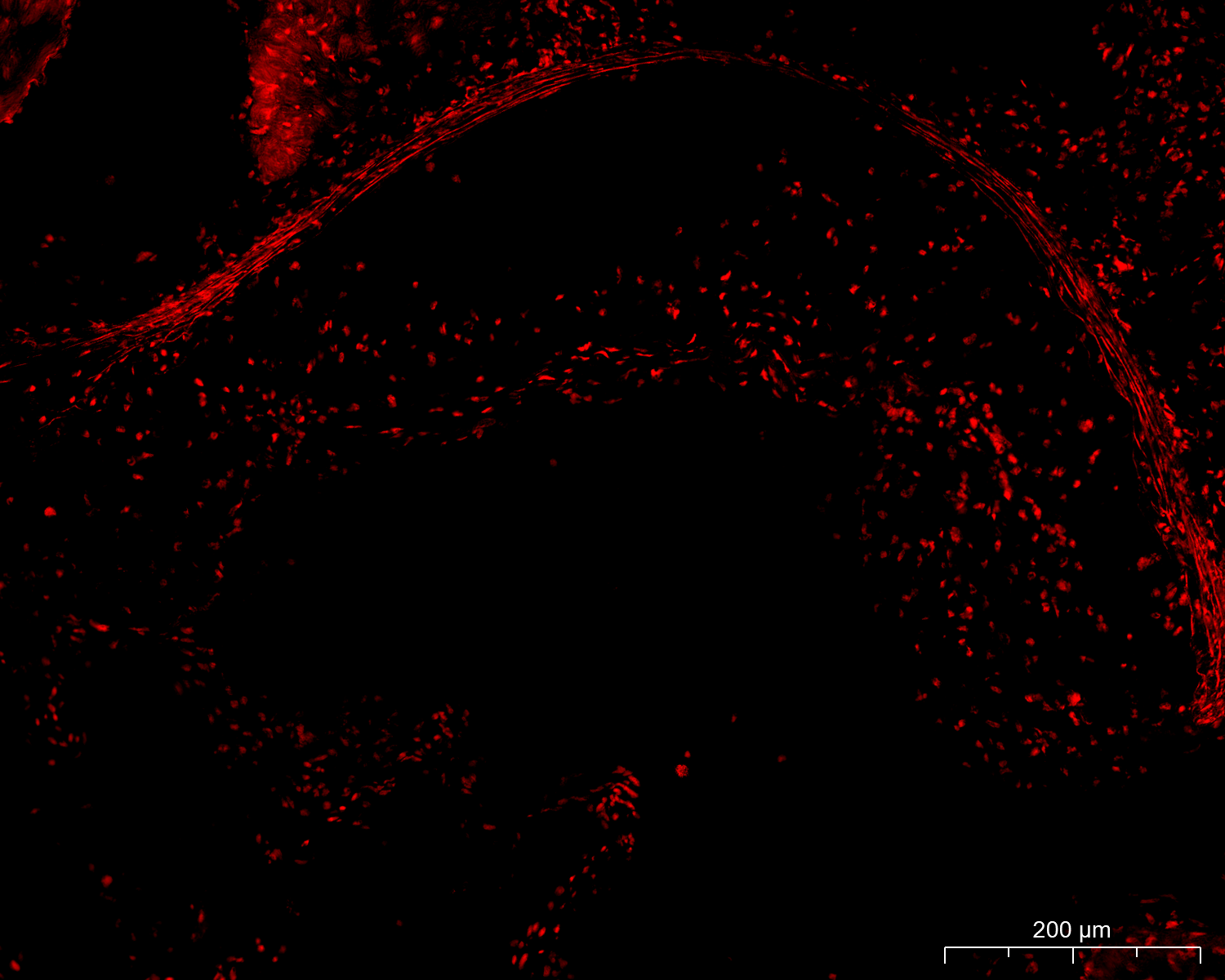

Supplement: S10 File — (ZIP) [file pone.0347758.s010.zip › 主动脉ROS/ROS/AS/28 ROS红_20.0x.tif]

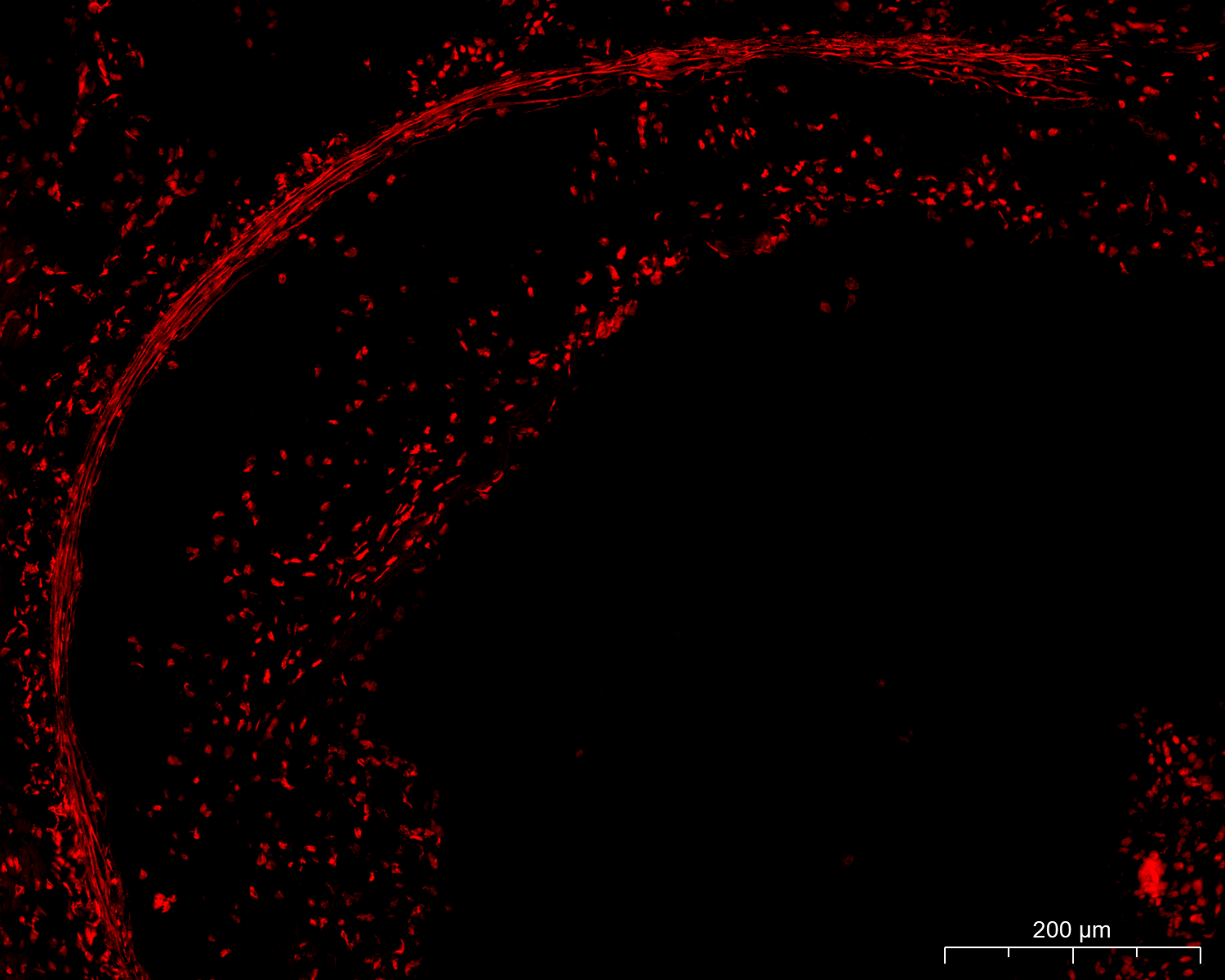

Supplement: S10 File — (ZIP) [file pone.0347758.s010.zip › 主动脉ROS/ROS/AS/31 ROS红_20.0x.tif]

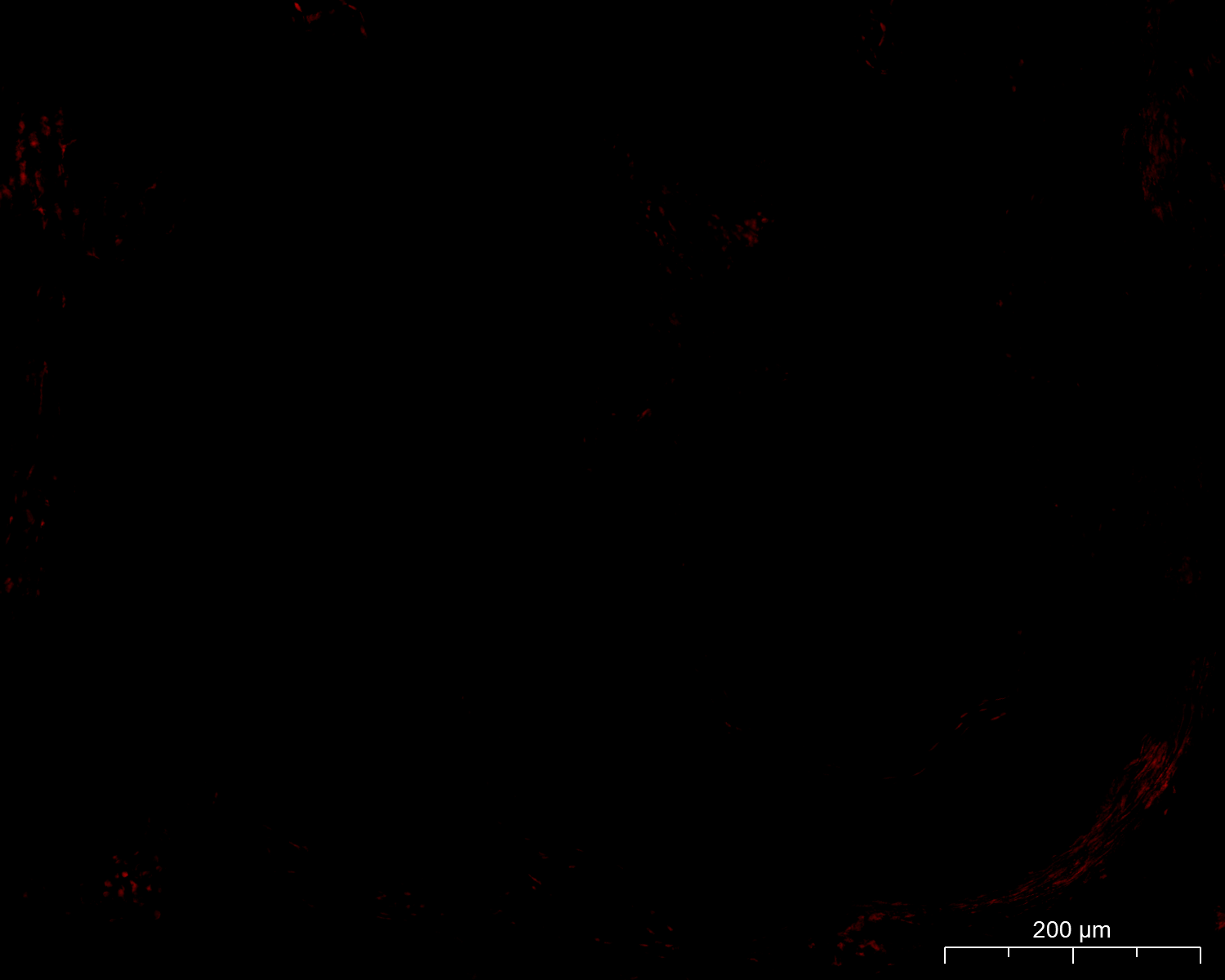

Supplement: S10 File — (ZIP) [file pone.0347758.s010.zip › 主动脉ROS/ROS/control/1 ROS红_20.0x.tif]

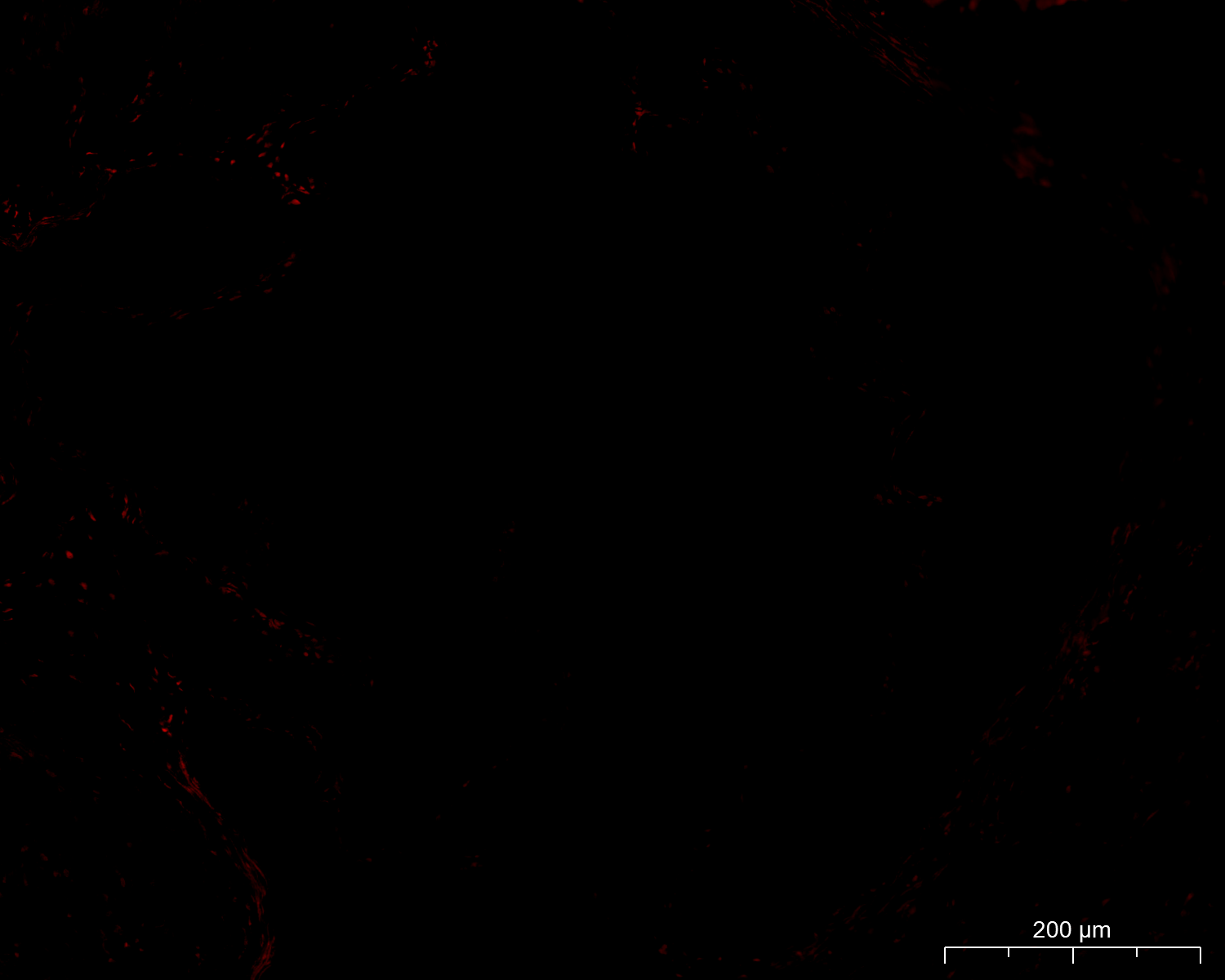

Supplement: S10 File — (ZIP) [file pone.0347758.s010.zip › 主动脉ROS/ROS/control/2 ROS红_20.0x.tif]

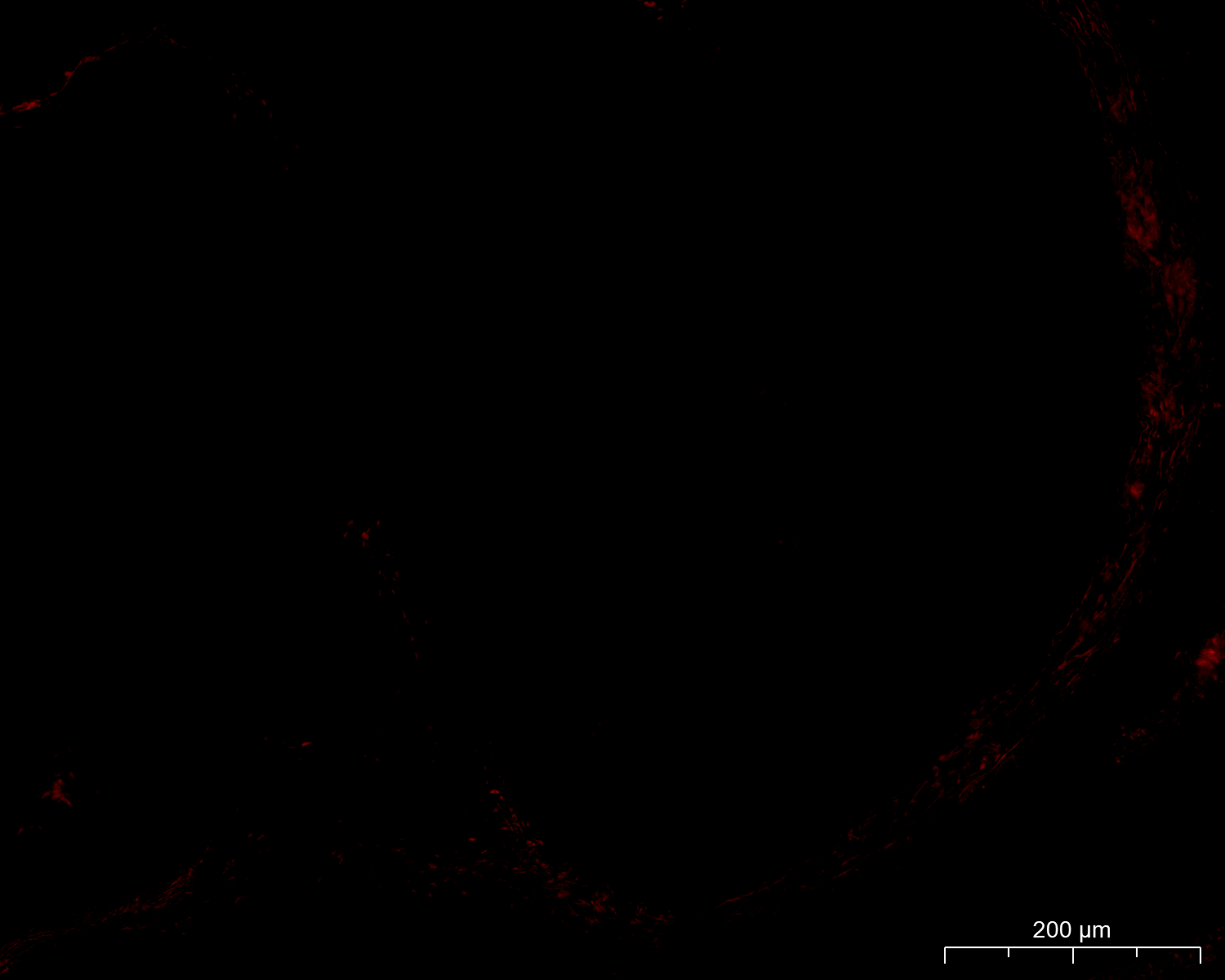

Supplement: S10 File — (ZIP) [file pone.0347758.s010.zip › 主动脉ROS/ROS/control/6 ROS红_20.0x.tif]

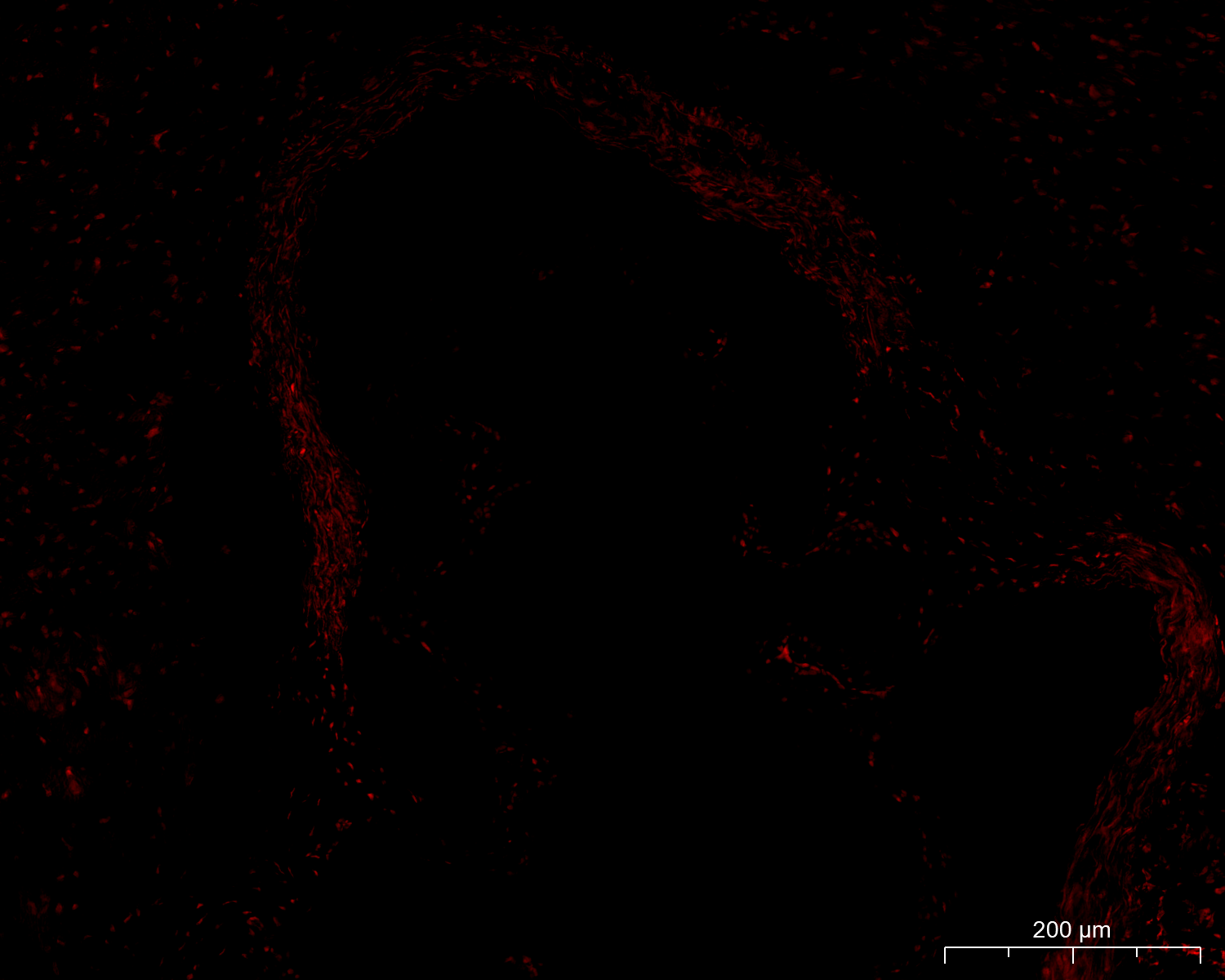

Supplement: S10 File — (ZIP) [file pone.0347758.s010.zip › 主动脉ROS/ROS/control/7 ROS红_20.0x.tif]

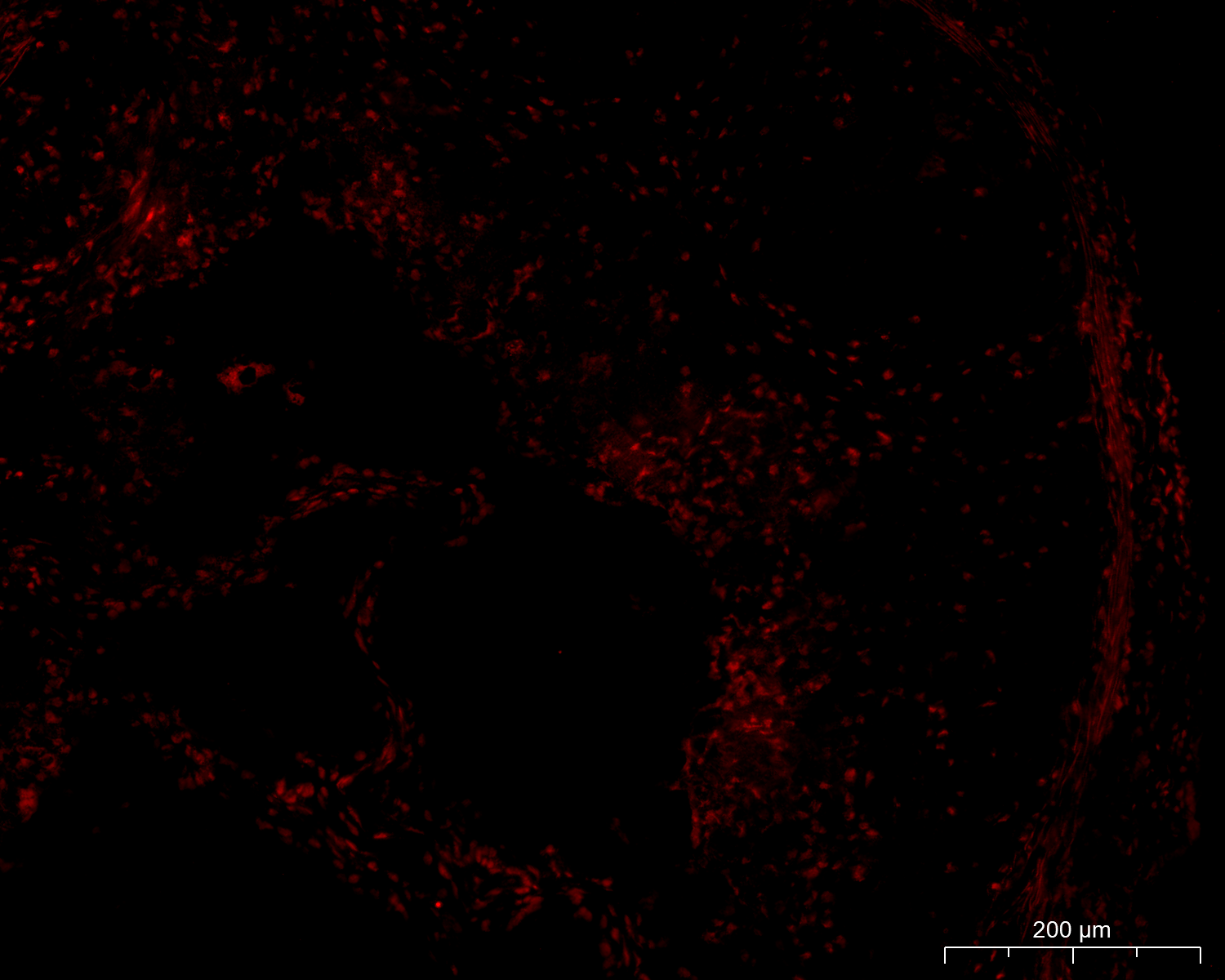

Supplement: S10 File — (ZIP) [file pone.0347758.s010.zip › 主动脉ROS/ROS/PSB-H/100 ROS红_20.0x.tif]

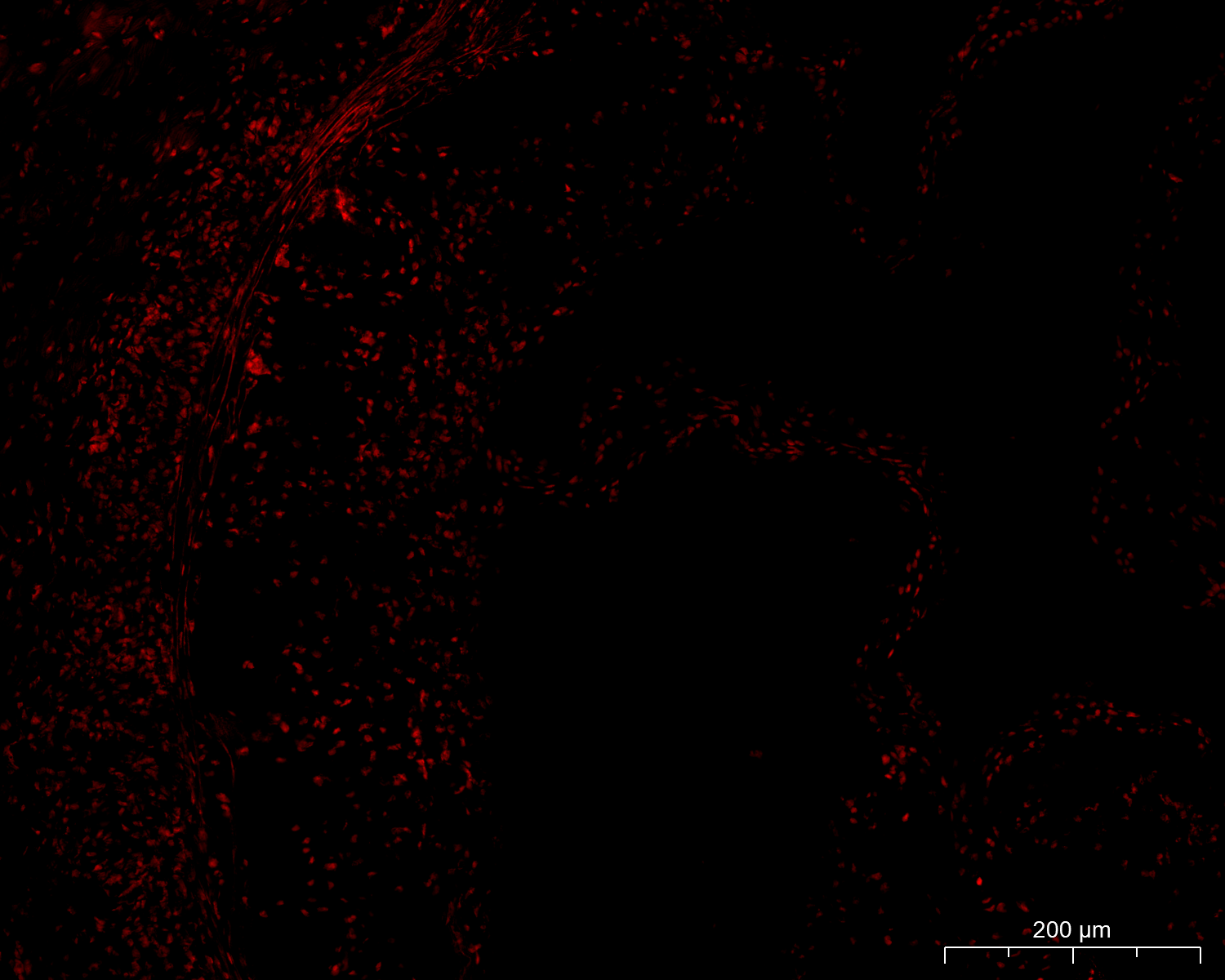

Supplement: S10 File — (ZIP) [file pone.0347758.s010.zip › 主动脉ROS/ROS/PSB-H/93 ROS红_20.0x.tif]

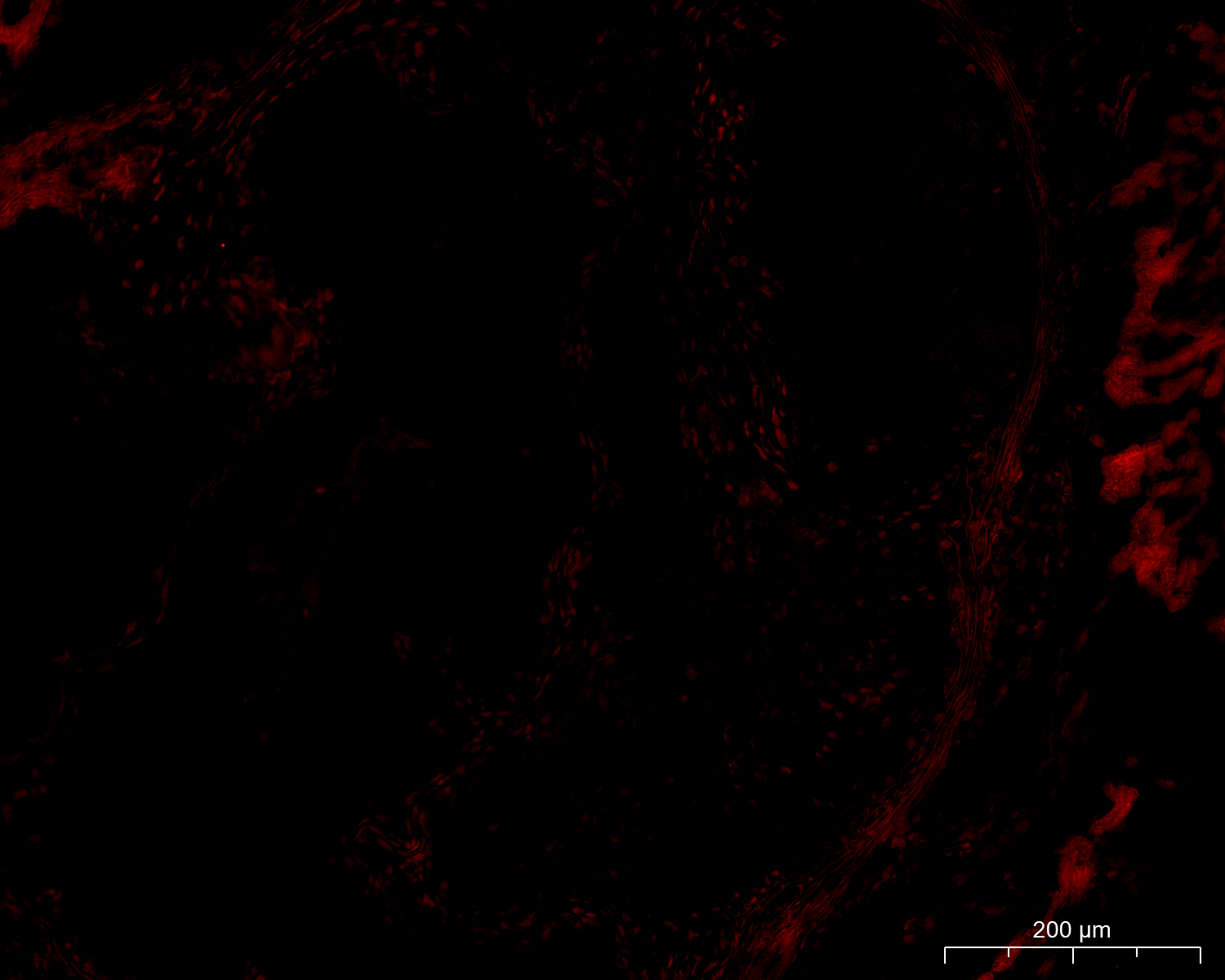

Supplement: S10 File — (ZIP) [file pone.0347758.s010.zip › 主动脉ROS/ROS/PSB-H/98 ROS红_20.0x.tif]

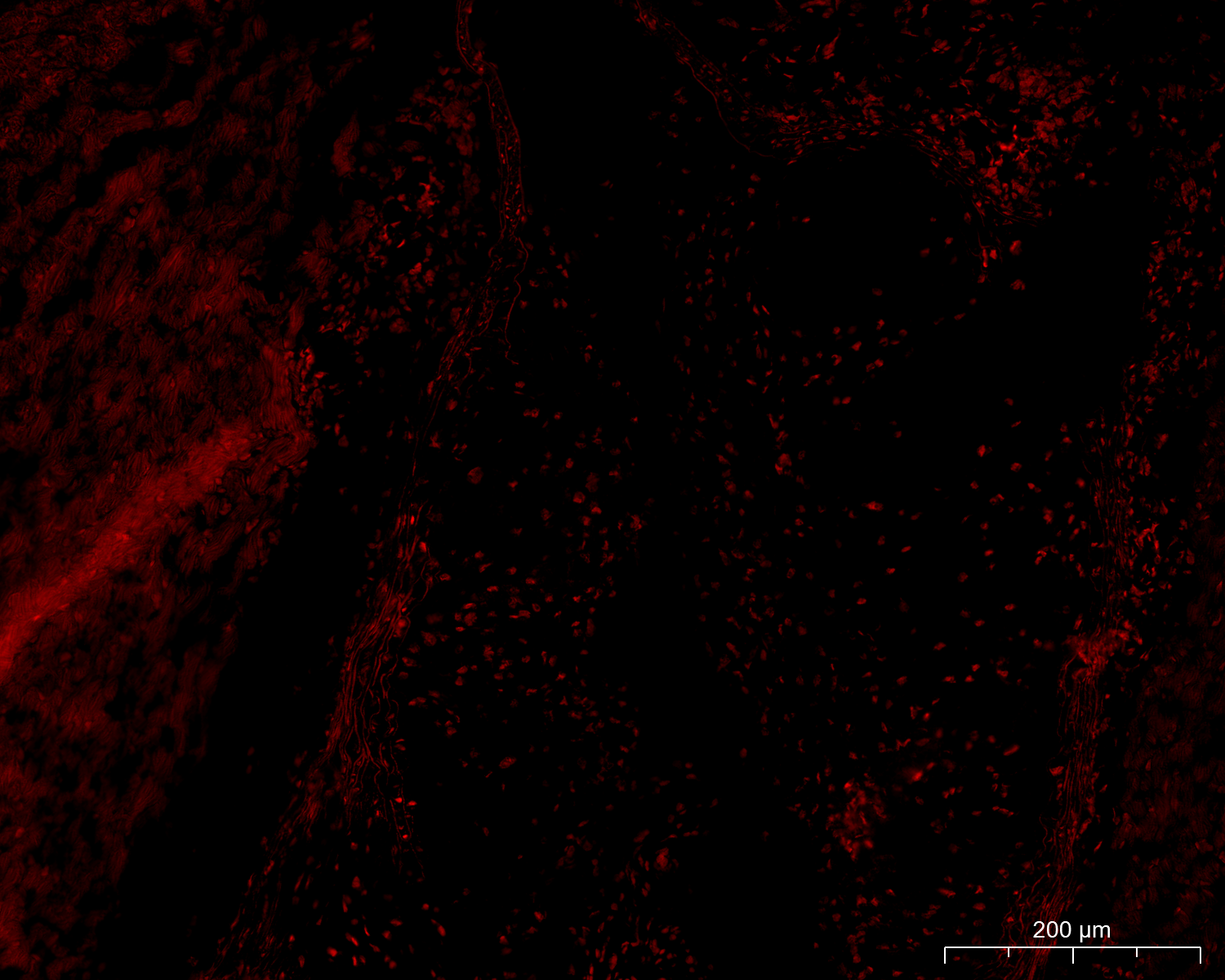

Supplement: S10 File — (ZIP) [file pone.0347758.s010.zip › 主动脉ROS/ROS/PSB-H/A1 ROS红_20.0x.tif]

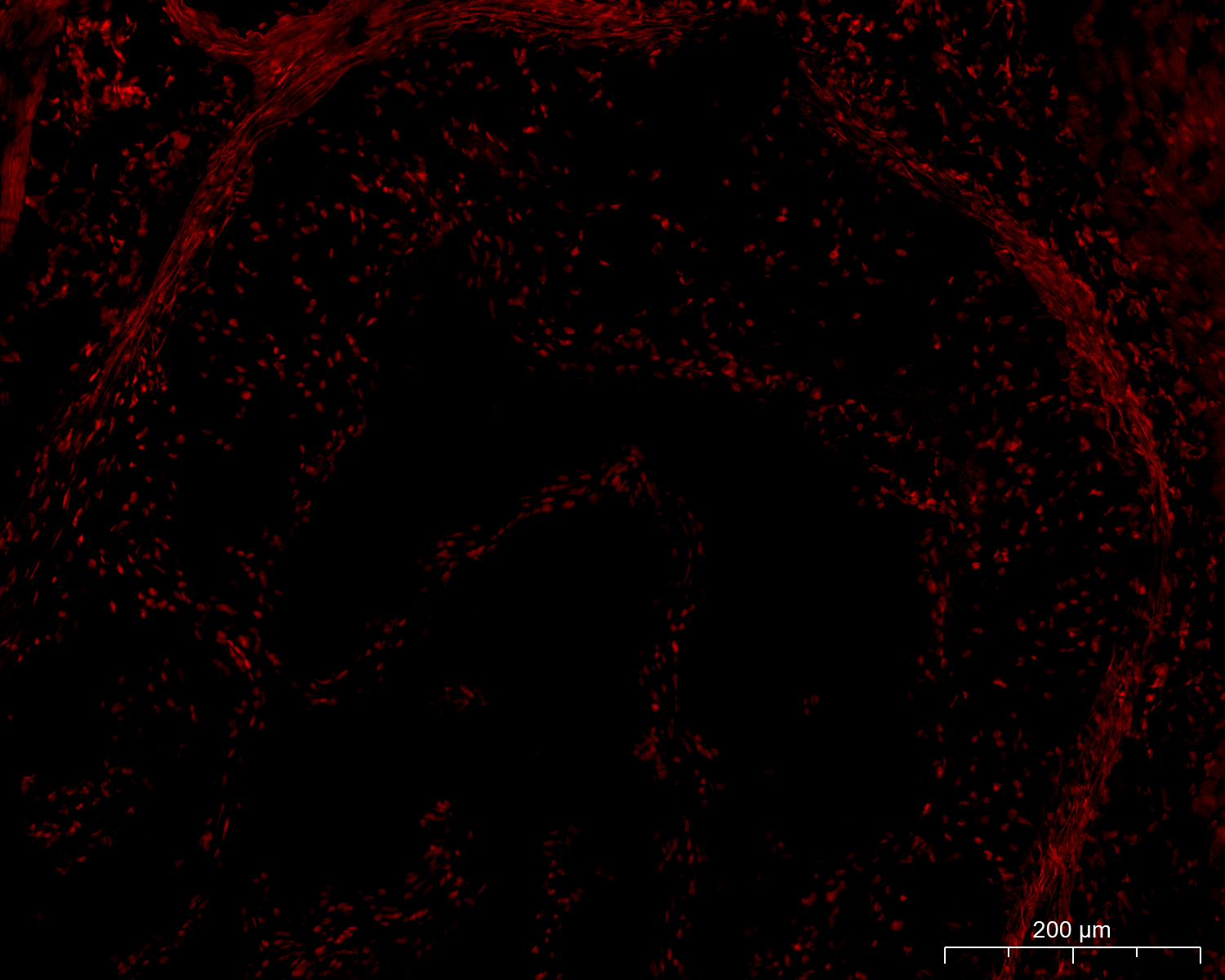

Supplement: S10 File — (ZIP) [file pone.0347758.s010.zip › 主动脉ROS/ROS/PSB-L/74 ROS红_20.0x.tif]

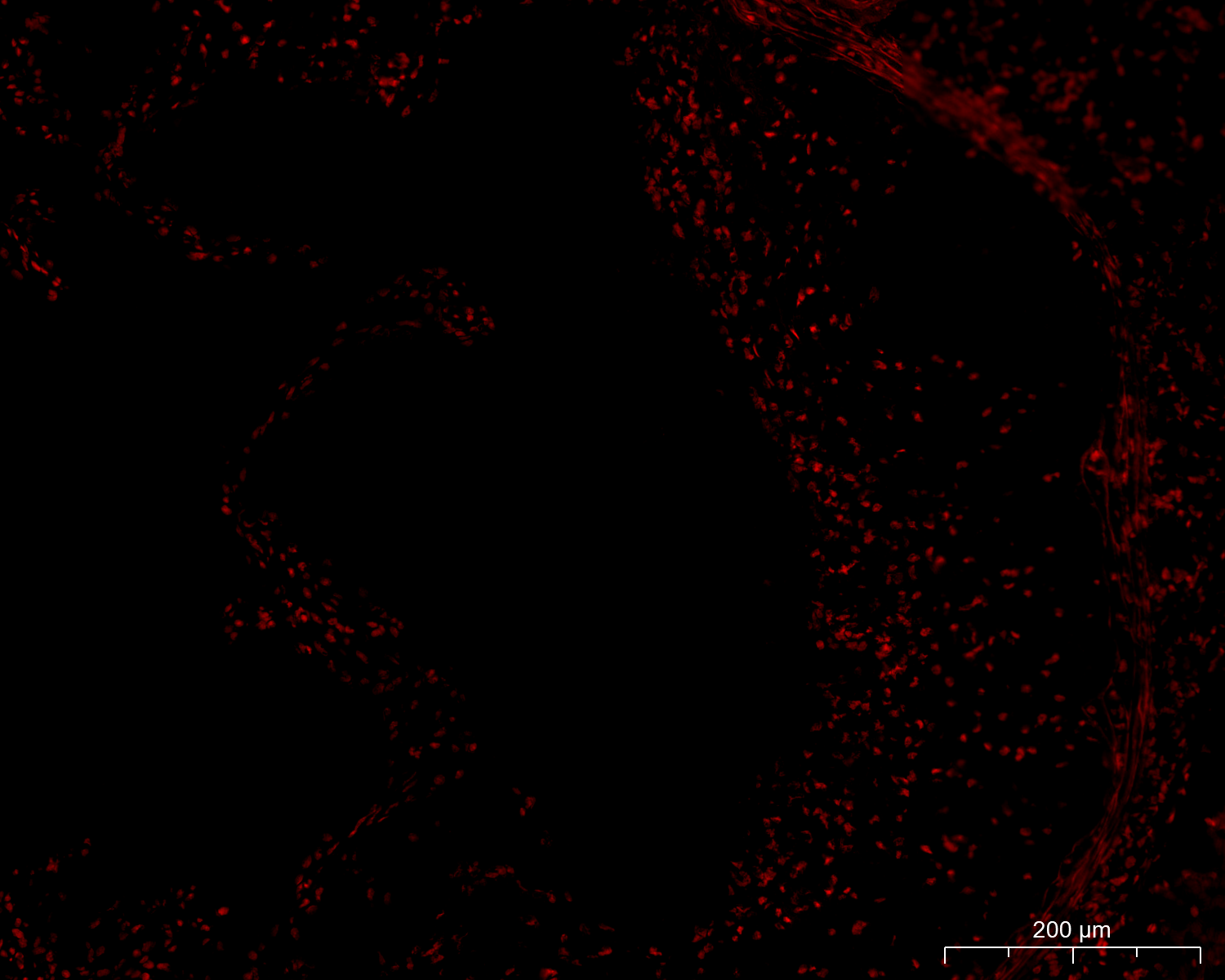

Supplement: S10 File — (ZIP) [file pone.0347758.s010.zip › 主动脉ROS/ROS/PSB-L/77 ROS红_20.0x.tif]

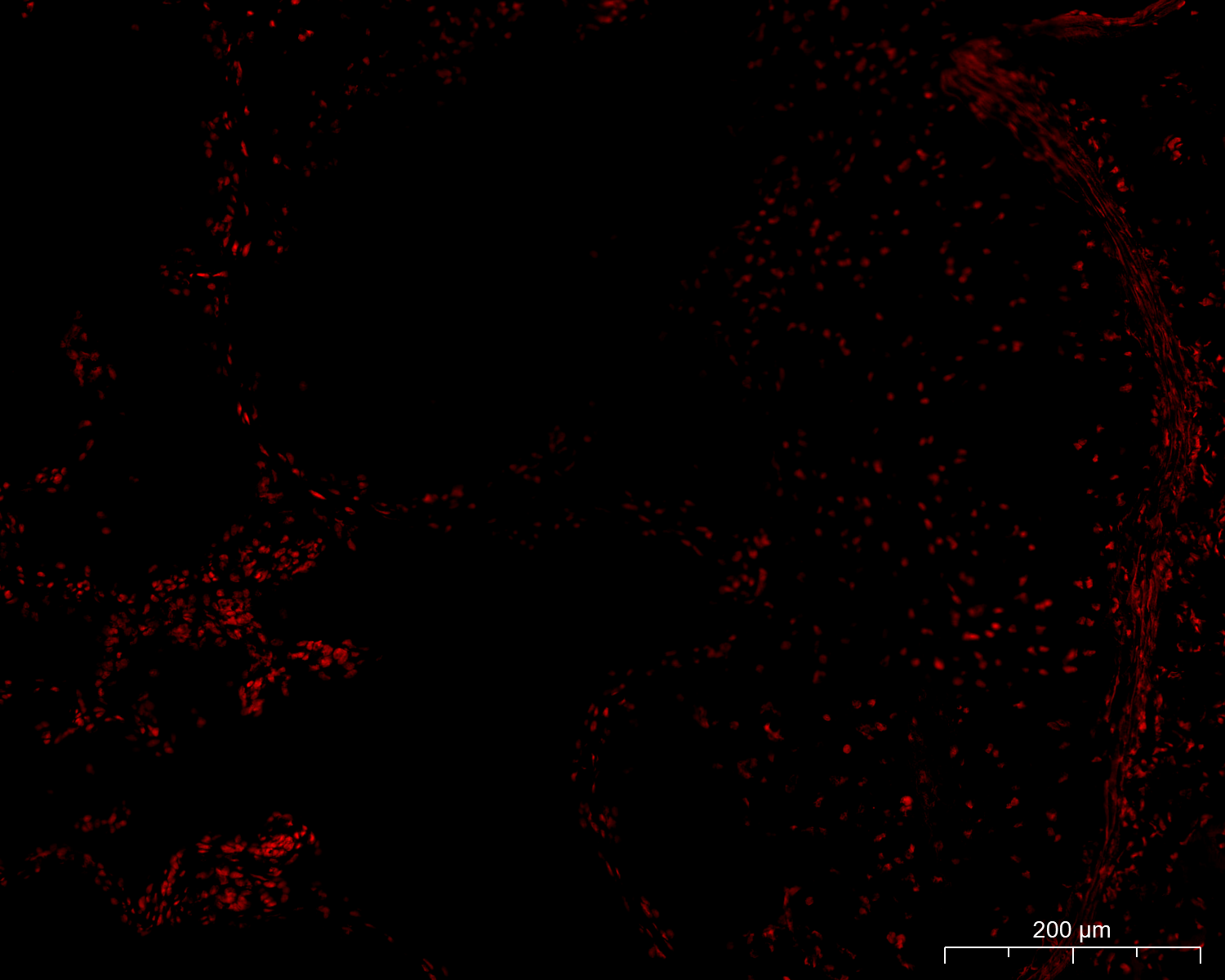

Supplement: S10 File — (ZIP) [file pone.0347758.s010.zip › 主动脉ROS/ROS/PSB-L/80 ROS红_20.0x.tif]

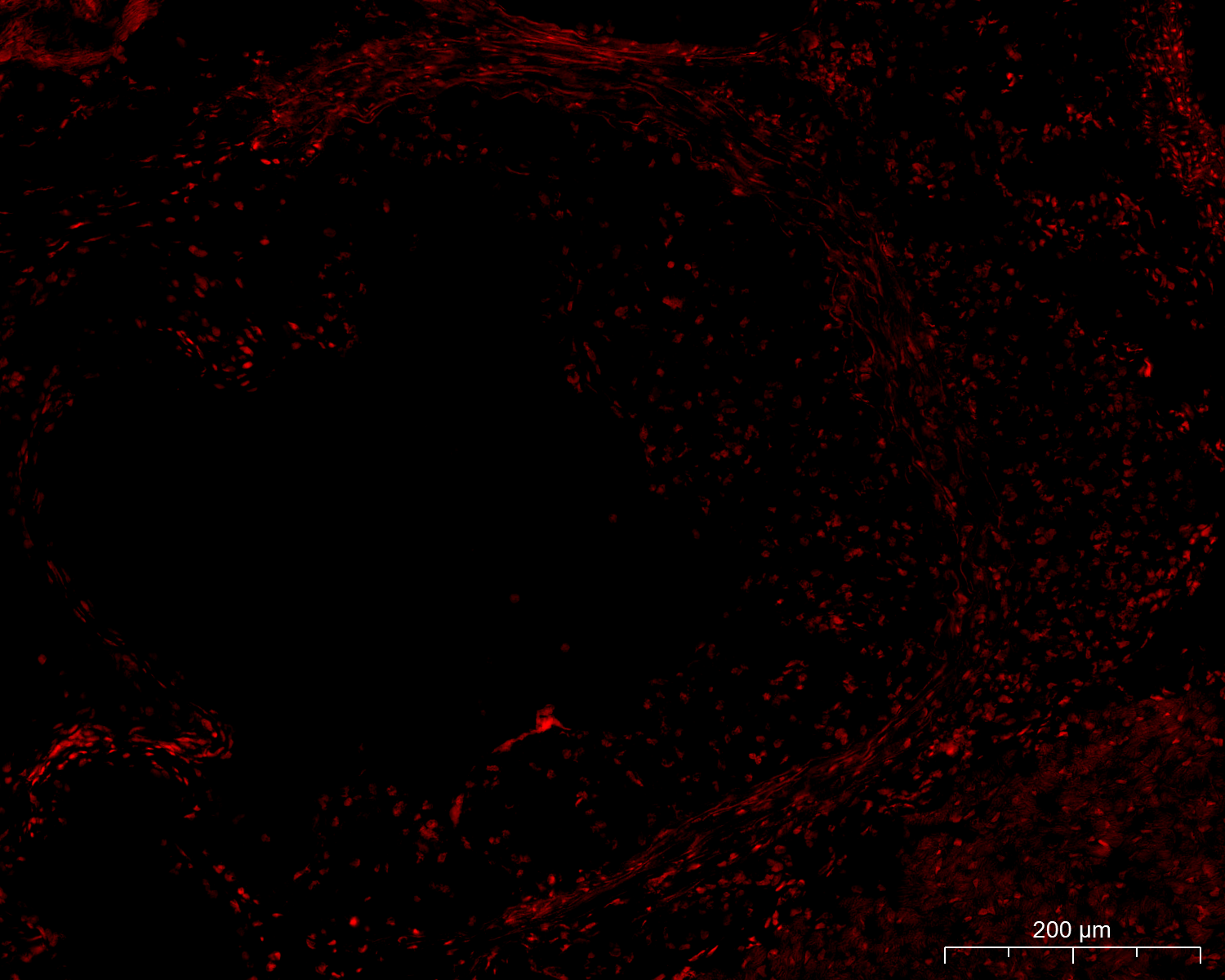

Supplement: S10 File — (ZIP) [file pone.0347758.s010.zip › 主动脉ROS/ROS/PSB-L/82 ROS红_20.0x.tif]

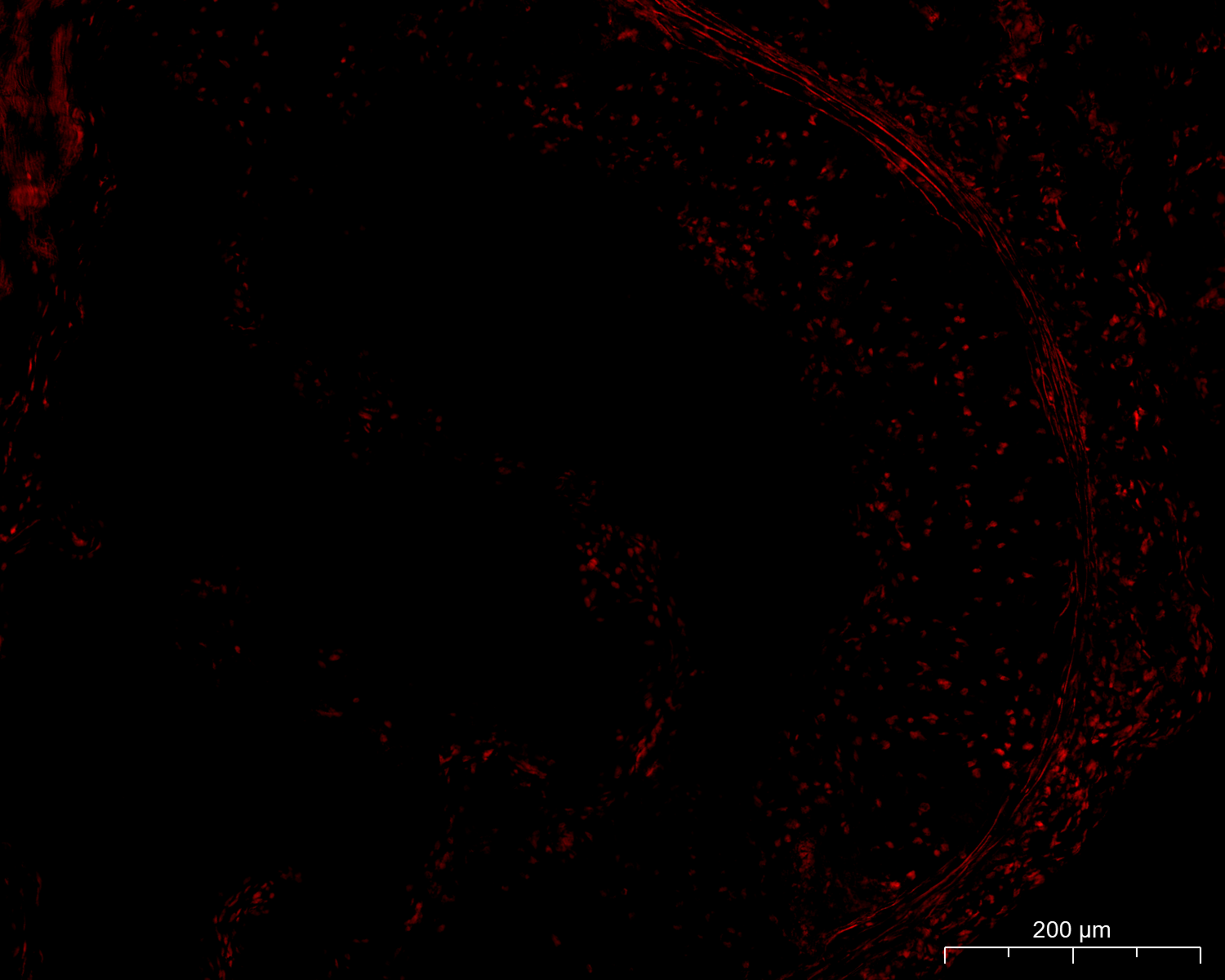

Supplement: S10 File — (ZIP) [file pone.0347758.s010.zip › 主动脉ROS/ROS/PSB-M/85 ROS红_20.0x.tif]

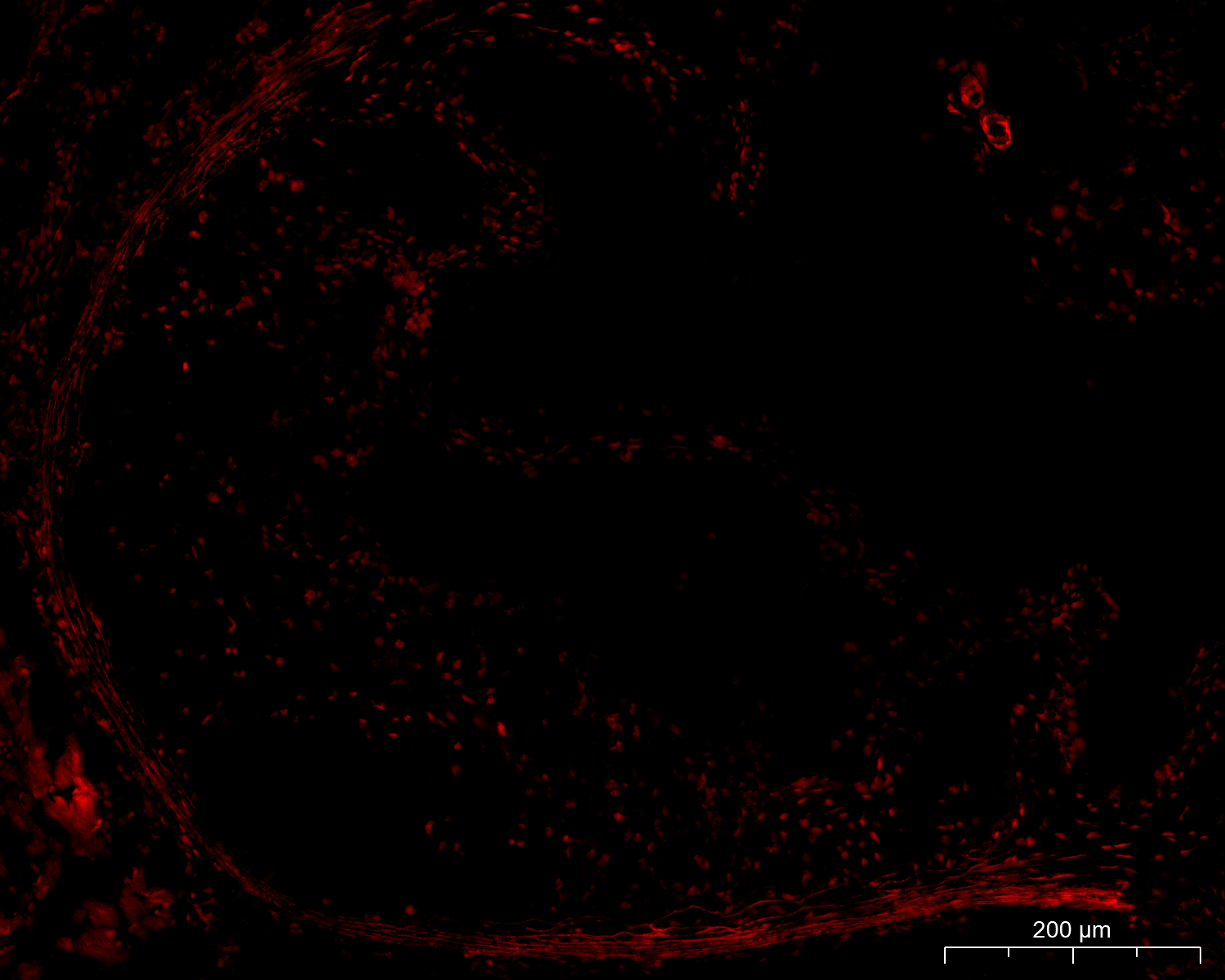

Supplement: S10 File — (ZIP) [file pone.0347758.s010.zip › 主动脉ROS/ROS/PSB-M/89 ROS红_20.0x.tif]

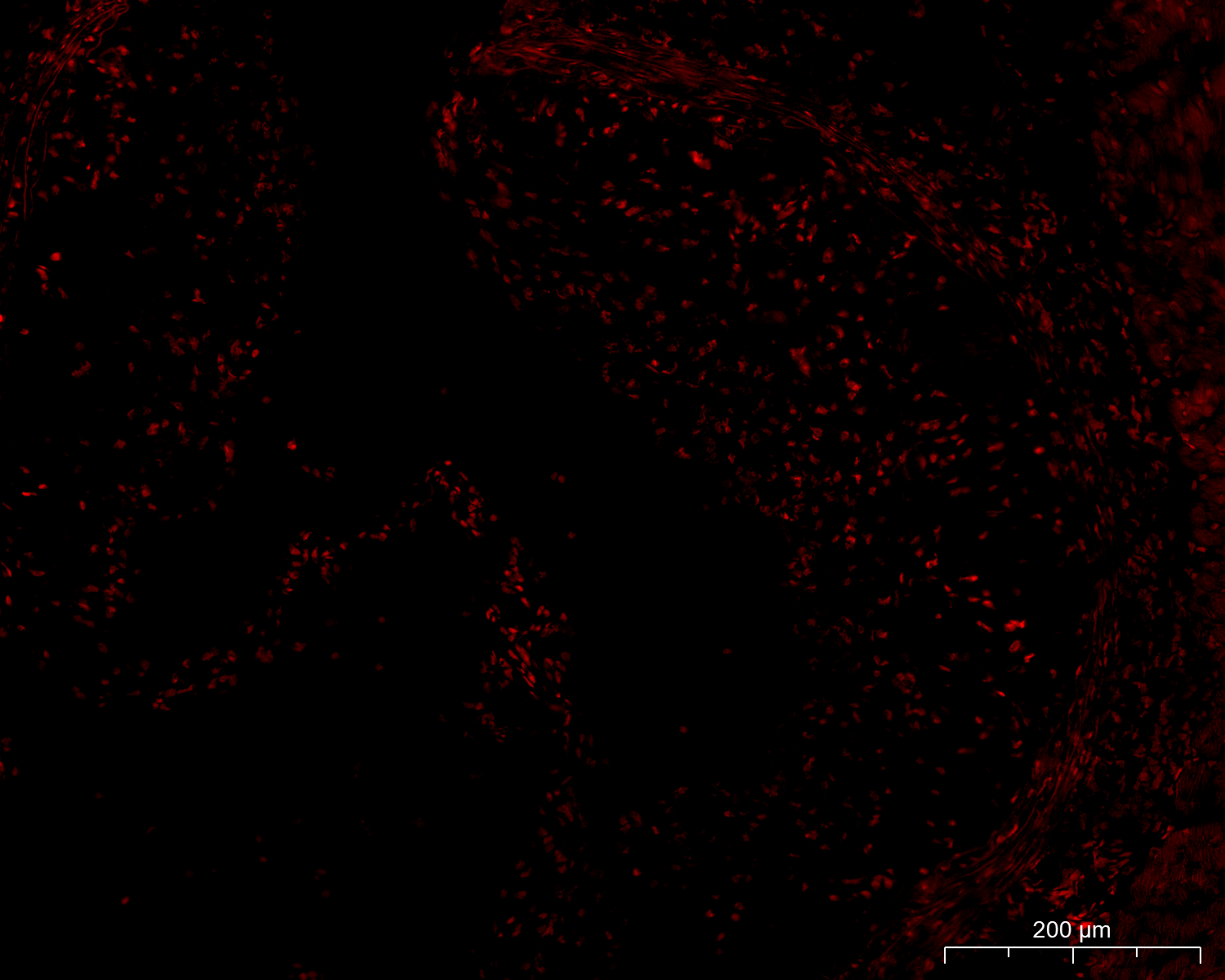

Supplement: S10 File — (ZIP) [file pone.0347758.s010.zip › 主动脉ROS/ROS/PSB-M/90 ROS红_20.0x.tif]

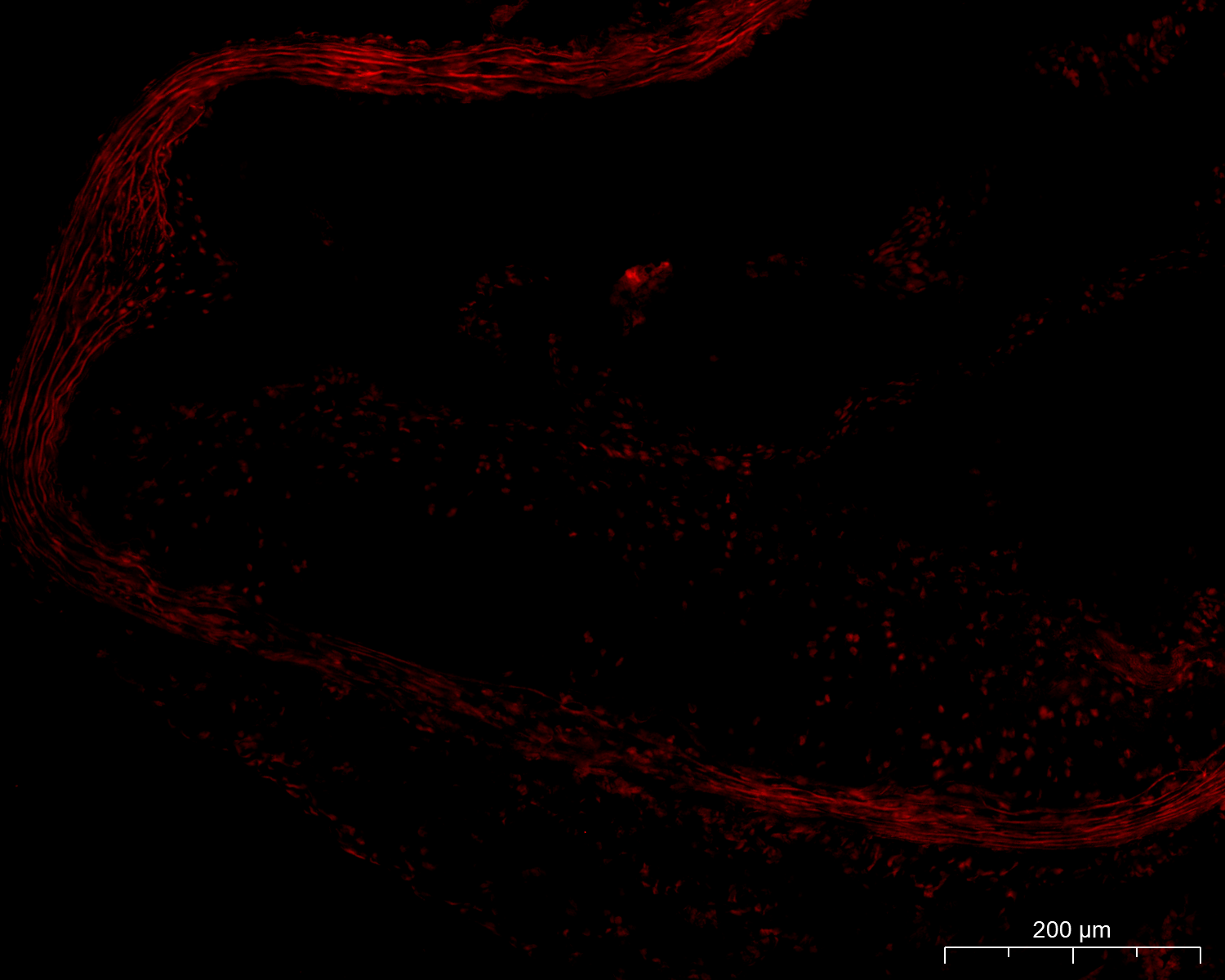

Supplement: S10 File — (ZIP) [file pone.0347758.s010.zip › 主动脉ROS/ROS/PSB-M/92 ROS红_20.0x.tif]

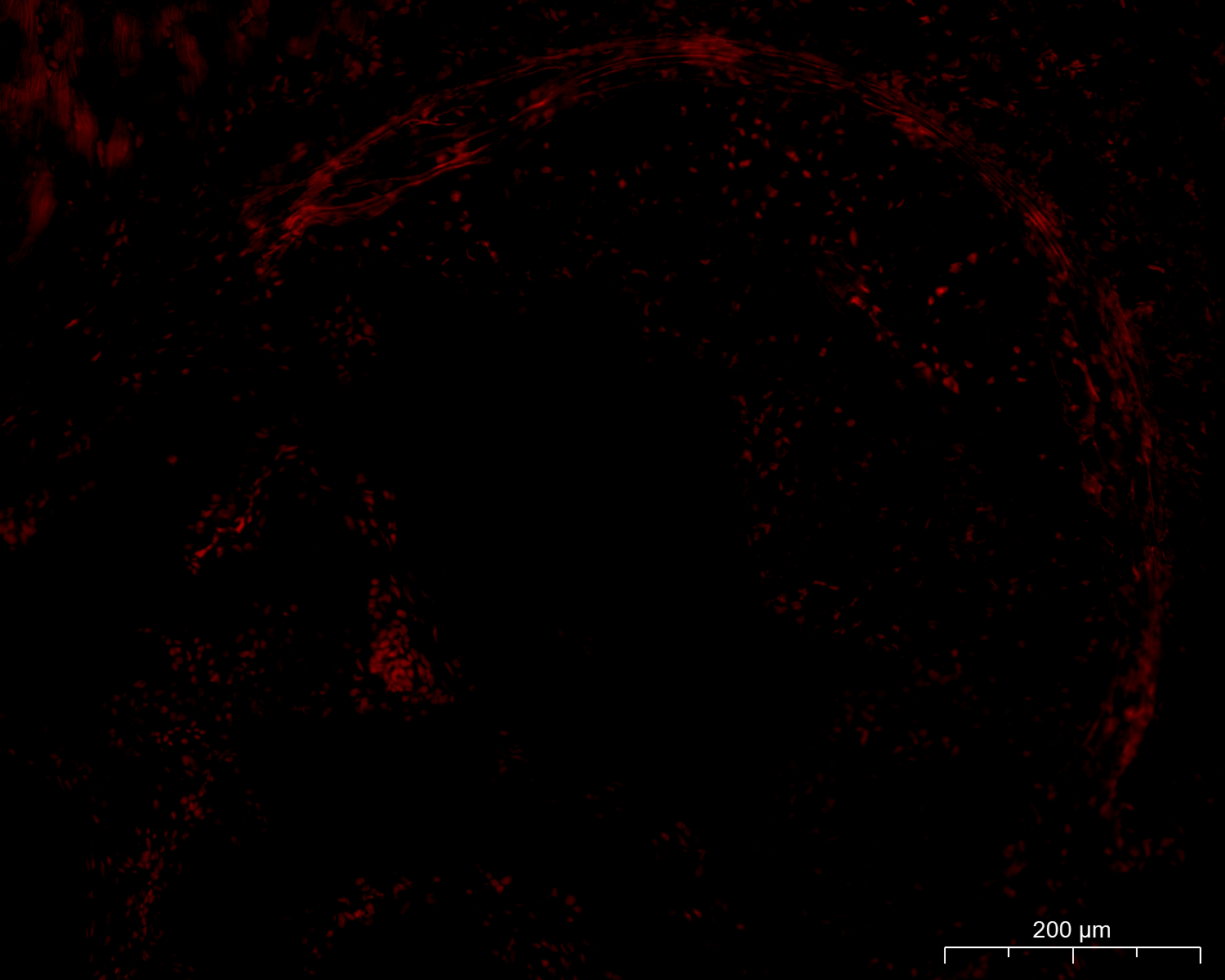

Supplement: S10 File — (ZIP) [file pone.0347758.s010.zip › 主动脉ROS/ROS/statin/37 ROS红_20.0x.tif]

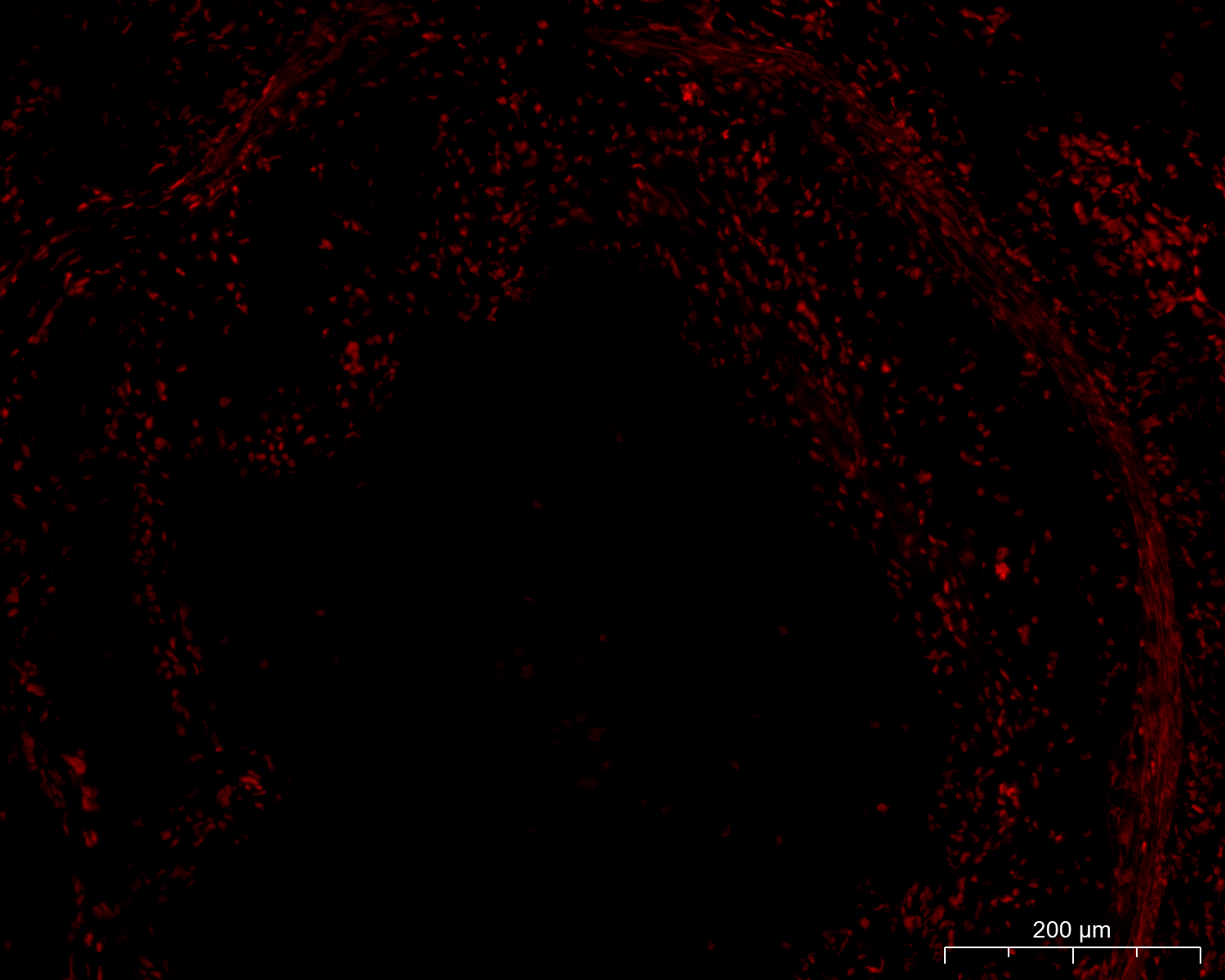

Supplement: S10 File — (ZIP) [file pone.0347758.s010.zip › 主动脉ROS/ROS/statin/38 ROS红_20.0x.tif]
