# Supplementary figures and images for: Polyphenol extract of Syzygium brachythyrsum mitigates atherosclerosis in high-fat diet induced ApoE-/- mice by regulating ROS/Keap1/Nrf2 pathway (part 4 of 4)
Source: PLoS One. 2026 May 5;21(5):e0347758. doi: 10.1371/journal.pone.0347758 (PMC13143111; doi:10.1371/journal.pone.0347758)

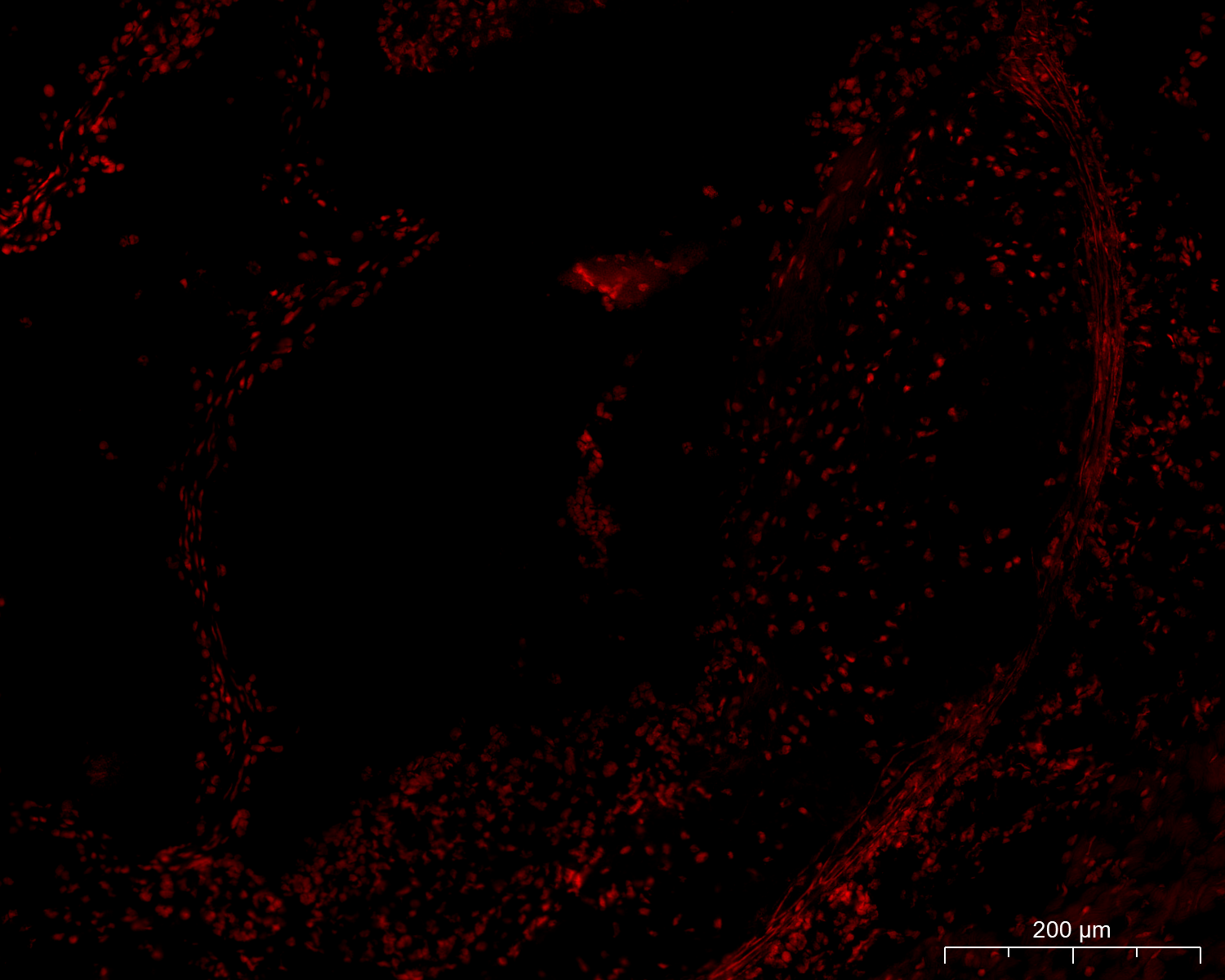

Supplement: S10 File — (ZIP) [file pone.0347758.s010.zip › 主动脉ROS/ROS/statin/40 ROS红_20.0x.tif]

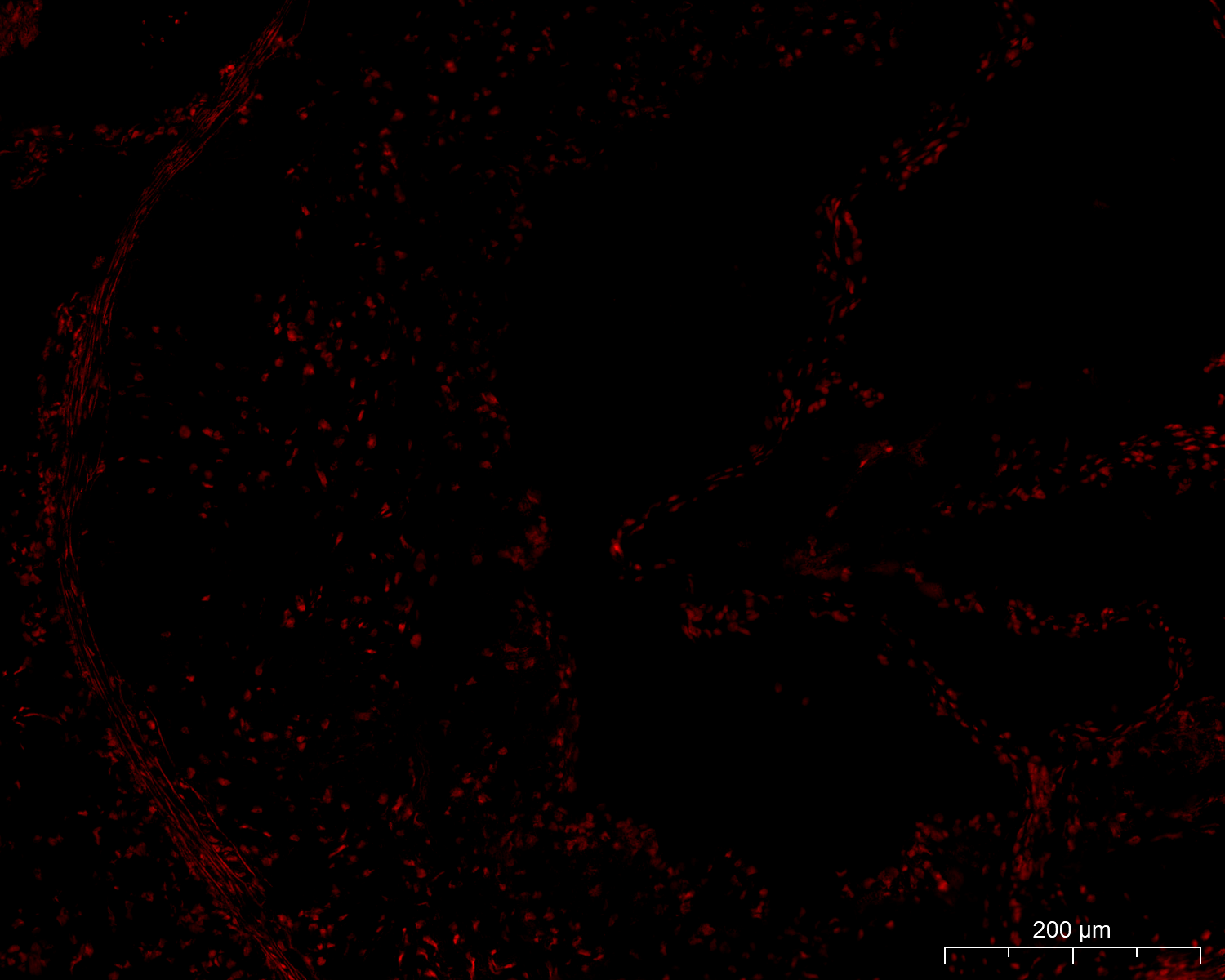

Supplement: S10 File — (ZIP) [file pone.0347758.s010.zip › 主动脉ROS/ROS/statin/41 ROS红_20.0x.tif]
